# A versatile glycosylation strategy *via* Au(iii) catalyzed activation of thioglycoside donors†

**DOI:** 10.1039/c6sc00633g

**Published:** 2016-03-08

**Authors:** Amol M. Vibhute, Arun Dhaka, Vignesh Athiyarath, Kana M. Sureshan

**Affiliations:** a School of Chemistry, Indian Institute of Science Education and Research Thiruvananthapuram KERALA-695016 India kms@iisertvm.ac.in http://kms514.wix.com/kmsgroup +91 4712597427

## Abstract

Among various methods of chemical glycosylations, glycosylation by activation of thioglycoside donors using a thiophilic promoter is an important strategy. Many promoters have been developed for the activation of thioglycosides. However, incompatibility with substrates having alkenes and the requirement of a stoichiometric amount of promoters, co-promoters and extreme temperatures are some of the limitations. We have developed an efficient methodology for glycosylation *via* the activation of thioglycoside donors using a catalytic amount of AuCl_3_ and without any co-promoter. The reaction is very fast, high-yielding and very facile at room temperature. The versatility of this method is evident from the facile glycosylation with both armed and disarmed donors and sterically demanding substrates (acceptors/donors) at ambient conditions, from the stability of the common protecting groups, and from the compatibility of alkene-containing substrates during the reaction.

## Abstract 2

We report a mild, versatile and high yielding glycosylation method *via* activation of thioglycoside donors using a Au(iii) catalyst and without using any co-promoter. Both armed and disarmed donors could be activated by this method and the anomeric selectivity could be tuned by changing the solvents.

## Graphical abstract

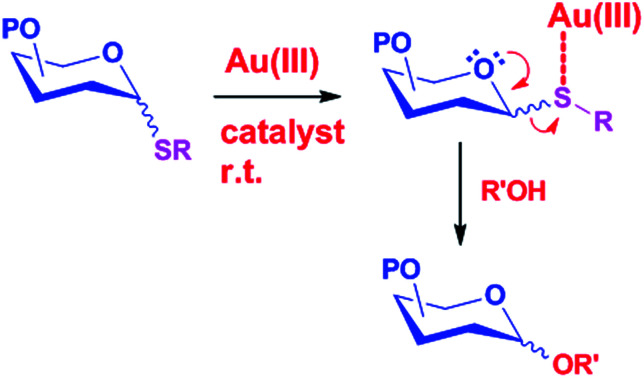

## Introduction

Various forms of carbohydrates play important biological roles and hence the chemical synthesis of glycoconjugates and oligosaccharides is an extremely important area of research.^1^ Chemical glycosylation involves transfer of the glycosyl moiety from an activated glycosyl donor to a hydroxyl group of the glycosyl acceptor, which can be another carbohydrate or another class of compounds.^2^ Several elegant methods of glycosylation have been developed using different donors.^1*k*,2*b*,3^ Among various glycosyl donors, thioglycosides have attracted a lot of attention from carbohydrate chemists because of their easy accessibility, stability, compatibility with various reaction conditions, orthogonality to other donors, *etc.*^4^ A major fraction of modern chemical glycosylations involve the use of thioglycosides as glycosyl donors. While thioglycosides are very stable, they can be easily activated for glycosylation by the use of electrophilic/thiophilic promoters. Varieties of reagents or combinations of reagents have been developed as promoters to activate thioglycoside donors.^2*b*,4*d*,*e*,5^ However, major drawbacks associated with this method are (i) the requirement of stoichiometric or excess amount of promoters which are toxic and expensive;^3*d*,6^ (ii) undesired side reactions arising from the highly electrophilic nature of the promoters, especially in substrates containing nucleophilic moieties;^4*e*,7^ (iii) the incompatibility of the activation conditions with substrates bearing alkenes;^8^ and (iv) the requirement of extremely low temperatures for the reaction. Development of novel and milder methods of thioglycoside activation that overcome these limitations is an agenda of utmost importance among chemists.^5*g*,6*a*^ Pohl *et al.* elegantly demonstrated the activation of thioglycoside donors using a sub-stoichiometric amount of Ph_3_Bi(OTf)_2_.^6*a*^ We herein report a novel method of thioglycoside activation using only a single additive in the catalytic amount that addresses these drawbacks.

**Scheme 1:**
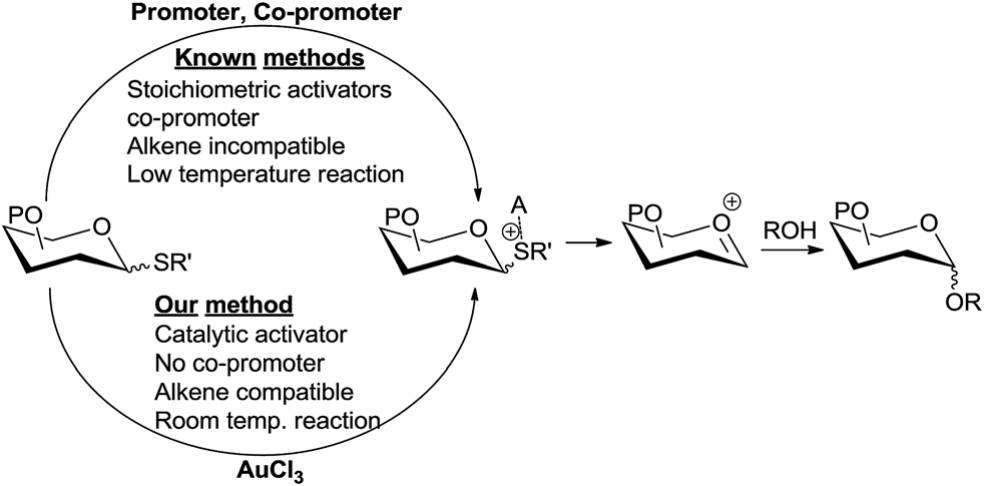
Thioglycoside activation strategies.

Yu *et al.*^9^ has elegantly demonstrated Au(i) catalysis in glycosylation by generating an oxocarbenium ion from the activation of a remote alkyne by Au(i). Further refinement has been done by Seeberger *et al.*, demonstrating that this method can be applied to flow chemistry.^10^ Hotha *et al.*^11^ used Au(iii) catalysts for the activation of propargyl glycosides. Later these authors have shown that even methyl glycosides can be activated by Au(iii),^12^ suggesting that alkyl glycosides, including propargyl glycosides, are presumably activated by the Lewis acidity of Au(iii). Vankar *et al.* have shown that AuCl_3_ in combination with phenylacetylene can be used to activate acetate^13^ and trichloroacetimidate^13*b*^ donors. Very recently, Schmidt *et al.* reported a mechanistically different glycosylation using trichloroacetimidate donors and a AuCl_3_ catalyst at −70 °C.^14^ However, glycosylations *via* the activation of these *O*-linked donors by Au(iii) are low yielding,^13*b*^ require extreme temperatures,^14^ longer reaction times,^15^ and a co-promoter such as phenylacetylene^13*a*^ or AgSbF_6_.^15^ We envisioned that the more Lewis basic sulphur in thioglycoside can be activated catalytically by Au(iii) under milder conditions (Scheme 1).

## Results and discussion

In order to investigate the feasibility of this proposal, a solution of the glycosyl donor, *p*-tolyl 2,3,4,6-tetra-*O*-benzyl-1-thio-β-d-glucopyranoside (D1), and methanol (A1) in dichloromethane was treated with 3 mol% of AuCl_3_. The donor was consumed completely in less than 10 minutes. To our satisfaction, methyl 2,3,4,6-tetra-*O*-benzyl-d-glucopyranosides were formed in good yield (60%) along with the hydrolysed product, 2,3,4,6-tetra-*O*-benzyl-d-glucopyranose. In order to avoid competing hydrolysis and thus to improve the yield of glycosylation, the reaction mixture was stirred with 4 Å molecular sieves prior to the addition of the catalyst. This way, the yield of the product could be improved to 91% (Table 1, entry 1). Similarly, glycosylation of benzyl alcohol (A2) and cyclohexanol (A3) with the donor D1 gave the corresponding glycosylated products in excellent yields (entries 2–3).

**Table 1:** Glycosylation of armed donors with various acceptors

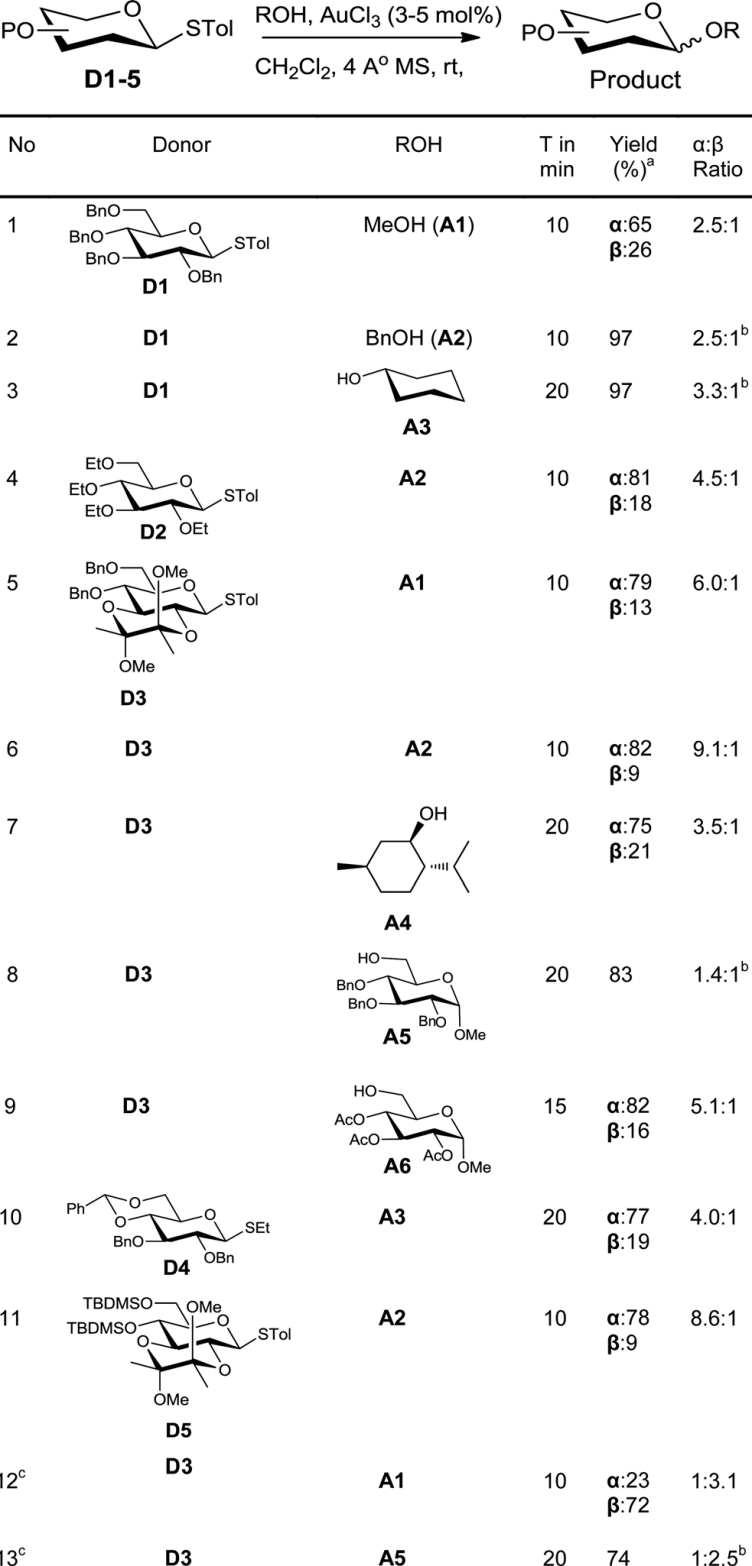

Change of protecting group on the donor had no effect on the reactivity, as was evident from the smooth glycosylation of the acceptor A2 with the donor, tetraethyl thioglucoside D2 (entry 4). The donor D3 having acid-labile BDA-protection also could be used for glycosylation of various acceptors (entries 5–9). Thus, simple acceptors A1 and A2, sterically crowded menthol (A4), and protected sugars A5 and A6 could be glycosylated using D3. Similarly, donor D4 having a benzylidene protecting group could also be used for glycosylation to get a glycosylated product in very good yield (entry 10). Silylated donor D5 also underwent glycosylation without desilylation (entry 11). Both thioalkyl and thioaryl glycosides behaved in a similar manner as was clear from the high yielding glycosylation using D4 as the donor (entry 10).

An ideal glycosylation strategy should be flexible to the choice of solvents in order to tune the α/β selectivity.^16^ Reactions in acetonitrile are known to produce predominantly β-anomers. In order to explore such tunability, we glycosylated donor D3 with acceptors A1 and A5 in acetonitrile. To our satisfaction, the reaction was smooth even in acetonitrile and the glycosylation proceeded with β-selectivity (entries 12–13) whereas α-selectivities were observed in DCM (entries 5 & 8).

Fascinated by these interesting results, we then moved on to find the generality of this glycosylation reaction by employing various other glycosyl donors (Table 2). The galactosyl donor D6 reacted with acceptors of different complexity to yield galactosylated products in very high yields (entries 14–15). For instance, galactosylation of the glucose derived acceptor A5 gave the corresponding disaccharides in high yield (entry 14). Mannosyl donor D7 could also be used for glycosylation as was clear from the efficient mannosylation of the acceptor A3 (entry 16).

**Table 2:** Glycosylation with complex donors and acceptors^*a*^

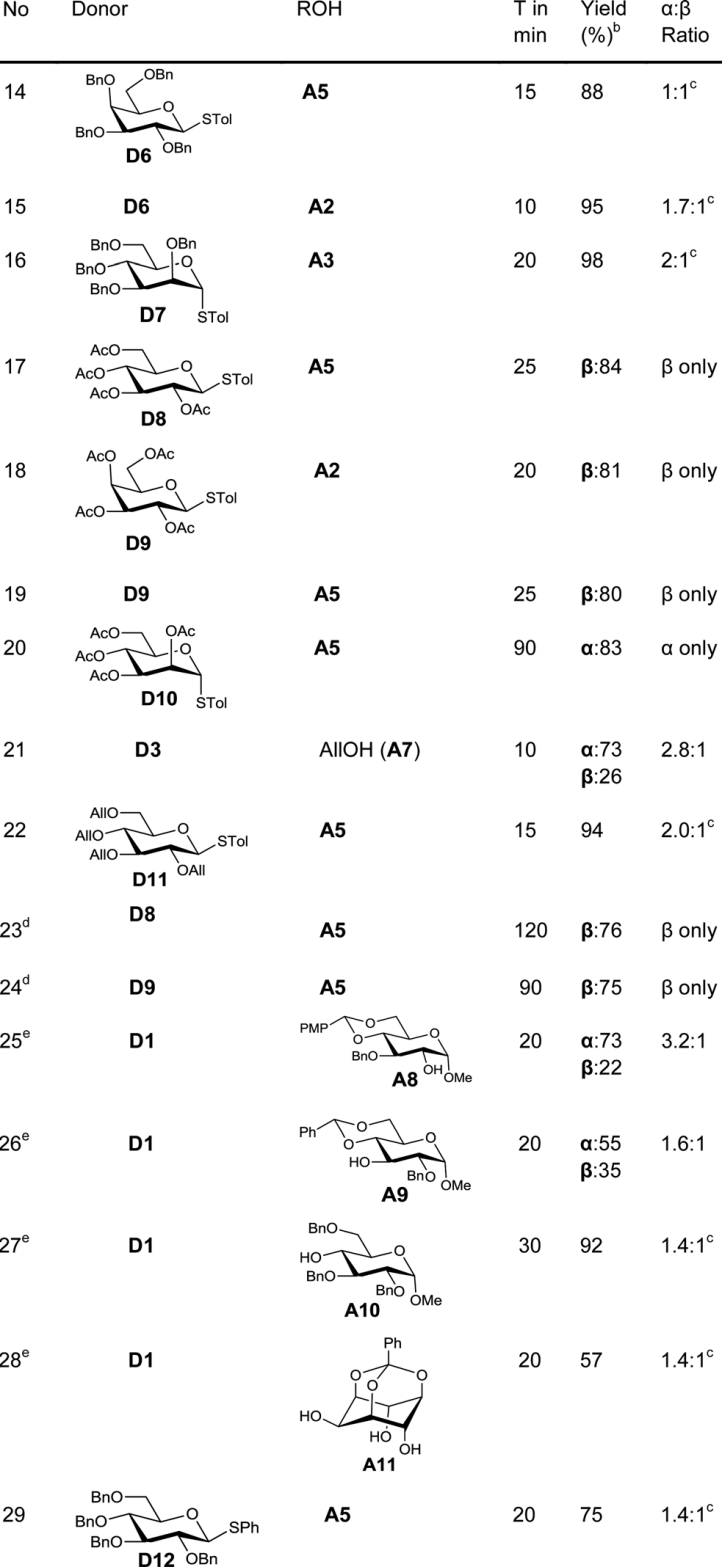

Glycosylation with disarmed donors is often very difficult, low yielding and requires harsh conditions.^17^ Methods for efficient glycosylation using disarmed donors are of high interest. In order to investigate the utility of our method for glycosylation using disarmed donors, we have made several disarmed donors of different monosaccharides. The glucosyl derivative A5 on glycosylation using *p*-tolyl 2,3,4,6-tetra-*O*-acetyl-β-d-glucoside (D8) as the donor gave the corresponding disaccharide (β-anomer exclusively) in 84% yield (entry 17). The disarmed galactosyl donor D9 could be used to galactosylate different acceptors (entries 18–19). For instance, galactosylation of the glucosyl acceptor A5 gave the β-disaccharide exclusively in good yield (entry 19). Similarly, the mannosylation of the acceptor A5 with the disarmed mannosyl donor D10, gave the corresponding α-disaccharide exclusively in excellent yield (entry 20). The rate of glycosylation using disarmed donors was slow as anticipated and took slightly longer to reach completion. It is interesting to note that our method is efficient for glycosylation using disarmed donors. It is worth noting that the previously reported Au(iii) mediated glycosylation does not work with disarmed donors.^11*a*^

One of the major issues associated with glycosylation using thioglycosides as donors is the requirement of activators that can react with the olefinic moiety and hence make the method unsuitable for reactions wherein the reaction partners (donor or acceptor) contain alkenes.^8,18^ Considering the frequent occurrence of unsaturations in glycosylated natural products,^2*d*^ methods to activate thioglycoside donors without affecting the olefinic motif are of great synthetic interest.^19^ As our method of activation requires only a catalytic amount of the activator, we explored the possibility of using this methodology in systems having olefinic motifs. In order to check the compatibility of the alkenyl motif in the acceptor, allyl alcohol (A7) was treated with the glucosyl donor D3 in the presence of 3 mol% of AuCl_3_. To our satisfaction, the allylglucosides could be isolated in quantitative yield (entry 21). This method is compatible with unsaturation in the donor too, as was evident from the efficient glycosylation of the acceptor A5 with donor D11 (entry 22).

In order to test the ability of other Au(iii) compounds to catalyze the glycosylation, we have used AuBr_3_ to carry out glycosylation of the acceptor A5 with the disarmed donors D8 and D9. These experiments suggested that AuBr_3_ is also able to catalyze the glycosylation reaction (entries 23–24). However, more catalyst (20 mol%) and a longer reaction time were essential for the reaction. This could be due to the comparatively weaker Lewis acidity of AuBr_3_. This low reactivity is beneficial in cases where the reactivity of the acceptor is low. For instance, the reaction of hindered acceptors A8–A10 with glycosyl donors in the presence of AuCl_3_ as the catalyst yields the corresponding glycosylated products in low yields along with the hydrolysed products arising from the competing hydrolysis of the donor. However, AuBr_3_ catalyzes the glycosylation reaction smoothly, yielding the corresponding disaccharides in very good yields (entries 25–27) and without any noticeable quantity of hydrolysed products.

Regioselective glycosylation of acceptors having multiple hydroxyl groups is an issue of great interest. *Myo*-inositol orthoesters having three secondary hydroxyl groups is one of the model acceptors to study the regioselectivity in glycosylations.^20^ In addition, the biological importance of glycosylinositols demands efficient methods for regioselective glycosylation.^21^ It is interesting to note that the reaction of orthobenzoate A11 and the donor D1 in the presence of AuBr_3_ gave the pseudodisaccharide with exclusive 2-*O*-glycosylation in moderate yield (entry 28). Though the yield is only moderate, it is interesting to note that not even traces of regioisomers could be observed in this case. However, glycosylation of *myo*-inositol orthoesters by previous methods resulted in the formation of a mixture of regioisomers.^20^ Due to the offensive smell of thiophenol, compared to thiotoluene, we have used thiotolyl glycosides in most of the cases. However, it is to be noted that the glycosylation with a phenyl thioglycoside as a donor also worked well as expected (entry 29).

**Scheme 2:**
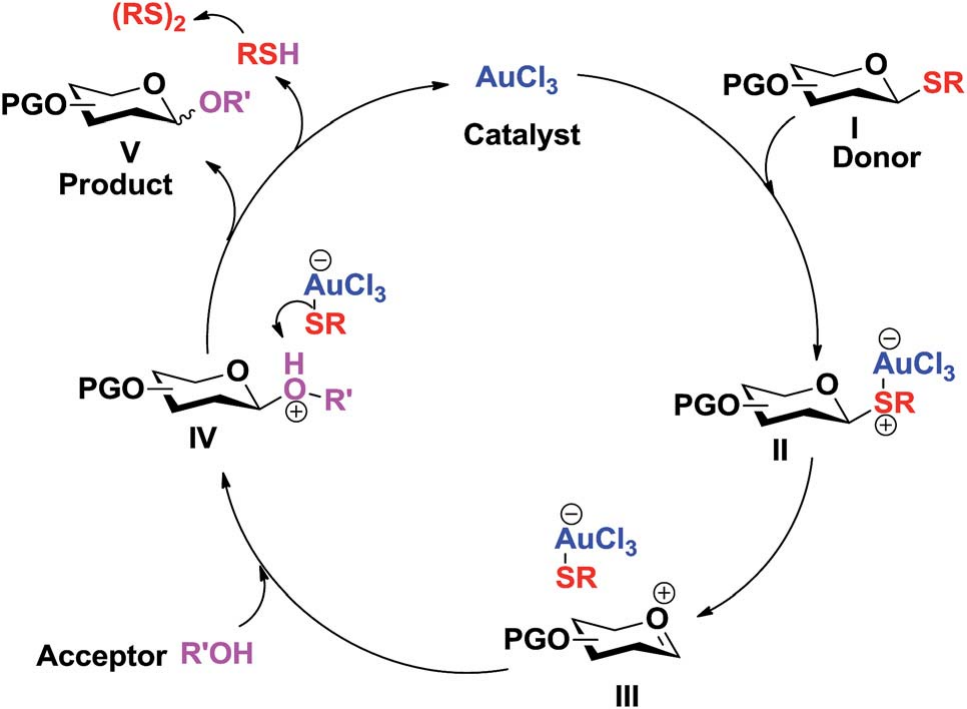
Plausible mechanism.

The following catalytic cycle can be conceived. The coordination of the nucleophilic sulphur to the Lewis acid (Au^3+^) results in the polarization and cleavage of the C–S bond, forming the oxocarbenium intermediate III. The nucleophilic attack of the acceptor alcohol (R'OH) to the oxocarbenium ion intermediate III gives the protonated glycoside IV. Dissociation of the weak anionic [AuCl_3_SR]^−^ complex, formed between the hard acid (Au^3+^) and soft base (RS^−^), regenerates the catalyst (AuCl_3_) and the thiolate, which abstracts the proton from the protonated glycoside IV to form the glycosylated product V and the thiol (RSH) which eventually forms disulfide. This catalytic cycle explains the sufficiency of low catalyst loading for this glycosylation (Scheme 2).

Apart from their use as bench-stable glycosyl donors, the thioalkyl/thioaryl groups serve as stable protecting groups for anomeric OH. For complex natural product/oligosaccharide synthesis, it is often essential to selectively deprotect the anomeric OH, leaving the other functional/protecting groups untouched, at a later stage of synthesis. Though several strategies are known for thioglycoside hydrolysis, they involve harsh conditions, the use of hazardous reagents, longer reaction times, the use of excess reagents and are incompatible with substrates having alkenes or other nucleophilic centers. The catalytic activation of thioglycosides discussed above suggested that they can be hydrolysed under mild conditions using water as the nucleophile. To explore such a possibility, thirteen different thioglycosides having different protecting/functional groups were subjected to hydrolysis in the presence of a catalytic amount of AuCl_3_ (Table S1; ESI†). To our satisfaction, all these glycosides could be deprotected in excellent yields.

## Conclusions

In conclusion, we have reported a mild, facile and high-yielding method for glycosylation using thioglycoside as a general donor. The reaction requires only 3 mol% of AuCl_3_ catalyst and no other promoters are required. The reaction is very fast and happens at room temperature. This method is compatible with wide varieties of acceptors and donors and gives excellent yields at very low catalyst loading. The method is versatile as (i) both armed and disarmed donors can be used for glycosylation; (ii) common protecting groups including acid sensitive protecting groups are stable under these conditions; (iii) this method can be used even when reaction partners have olefinic motifs and (iv) it allows flexibility in the choice of solvents to tune the α/β selectivity. This is the first report on the catalytic activation of thioglycoside donors at ambient conditions, without the use of any other co-promoter. In view of high yield, shorter reaction time, and catalytic activation by a minute amount of catalysts, this method might find application in oligosaccharide and glycoconjugate synthesis.

## Supplementary Material

SC-007-C6SC00633G-s001

SC-007-C6SC00633G-s002
