# Correction: A versatile glycosylation strategy *via* Au(iii) catalyzed activation of thioglycoside donors

**DOI:** 10.1039/c6sc90051h

**Published:** 2016-08-02

**Authors:** Amol M. Vibhute, Arun Dhaka, Vignesh Athiyarath, Kana M. Sureshan

**Affiliations:** a School of Chemistry , Indian Institute of Science Education and Research , Thiruvananthapuram , Kerala-695016 , India . Email: kms@iisertvm.ac.in ; Fax: +91 4712597427

## Abstract

Correction for ‘A versatile glycosylation strategy *via* Au(iii) catalyzed activation of thioglycoside donors’ by Amol M. Vibhute *et al.*, *Chem. Sci.*, 2016, **7**, 4259–4263.



## 

The authors wish to amend statements in the original article that concern the effectiveness of low quantities of AuX_3_ additive for thioglycoside activation. The use of 3–20 mol% AuX_3_ is reported in the original article. However, the authors have recently discovered that, at low AuX_3_ loading, the yield of glycosylation varied from trial-to-trial and depending on the source of AuX_3_. The yield also varied considerably depending on the time since the bottle of AuX_3_ had been opened, irrespective of its source. However, with higher AuX_3_ loading (0.8 equiv.), the reactions were reproducible in high yields across multiple attempts. As representative examples, the reactions in entries 8–10, 14, 17 and 26 were repeated with 0.8 equiv. of AuX_3_. In all of these cases, the yields of the glycosylation products were good (74–89%) with minor amounts of hydrolyzed products. For consistent and reproducible results, it is necessary to use 0.8 equiv. of AuX_3_.

Therefore, all references to AuX_3_ as a catalyst, or its use in catalytic amounts, should be disregarded throughout the original article and the following changes to the values presented in Tables 1 and 2 should be noted:

The reaction conditions given for the general reaction embedded in Table 1 should be corrected to the following: “ROH, AuCl_3_ (0.8 equiv.), CH_2_Cl_2_, 4 Å MS, rt”.

The footnote in Table 2 should be changed to the following: “^a^ Reaction condition: donor (1.0 equiv.), acceptor (1.0 equiv.), CH_2_Cl_2_, AuCl_3_, 4 Å MS, rt. ^b^ Isolated by chromatography. ^c^ Calculated using NMR spectroscopy. ^d/e^ AuBr_3_ (0.8 equiv. of AuX_3_ was used).”

The authors apologise for these errors and any consequent inconvenience to editors and readers.

The Royal Society of Chemistry apologises for these errors and any consequent inconvenience to authors and readers.

