# Supplementary material for: A versatile glycosylation strategy via Au(iii) catalyzed activation of thioglycoside donors
Source: Chem Sci. 2016 Mar 8;7(7):4259–63. doi: 10.1039/c6sc00633g (PMC6054025; doi:10.1039/c6sc00633g)

Electronic Supplementary Material (ESI) for Chemical Science.
This journal is © The Royal Society of Chemistry 2016

Electronic Supplementary Information (ESI) for

A Versatile Glycosylation Strategy via Au(III) Catalyzed Activation of Thioglycoside Donors

Amol M. Vibhute, Arun Dhaka, Vignesh Athiyarath and Kana M. Sureshan*

School of Chemistry, Indian Institute of Science Education and Research

Thiruvananthapuram, KERALA-695016, India, E-mail: kms@iisertvm.ac.in.

INDEX

1. Materials and methods	S2	20. Glycosylation of **D6** with **A5**	S18
2. Synthesis of **D11**	S3	21. Glycosylation of **D6** with **A2**	S19
3. Synthesis of **D2**	S4	22. Glycosylation of **D7** with **A3**	S20
4. Synthesis of **2**	S5	23. Glycosylation of **D8** with **A5**	S20
5. Synthesis of **D5**	S5	24. Glycosylation of **D9** with **A2**	S21
6. Synthesis of **D3**	S6	25. Glycosylation of **D9** with **A5**	S21
7. General procedure for glycosylation	S8	26. Glycosylation of **D10** with **A5**	S22
8. Glycosylation of **D1** with **A1**	S8	27. Glycosylation of **D3** with **A7**	S22
9. Glycosylation of **D1** with **A2**	S9	28. Glycosylation of **D11** with **A5**	S23
10. Glycosylation of **D1** with **A3**	S9	29. Glycosylation of **D8** with **A5**	S24
11. Glycosylation of **D2** with **A2**	S10	30. Glycosylation of **D9** with **A5**	S24
12. Glycosylation of **D3** with **A1**	S11	31. Glycosylation of **D1** with **A8**	S24
13. Glycosylation of **D3** with **A2**	S12	32. Glycosylation of **D1** with **A9**	S26
14. Glycosylation of **D3** with **A4**	S13	33. Glycosylation of **D1** with **A10**	S27
15. Glycosylation of **D3** with **A5**	S14	34. Glycosylation of **D1** with **A11**	S27
16. Glycosylation of **D3** with **A6**	S15	35. Structural assignment of **26**	S28
17. Glycosylation of **D4** with **A3**	S16	36. Hydrolysis of Thioglycosides	S30
18. Glycosylation of **D5** with **A2**	S17	37. General procedure for hydrolysis	S36
19. Glycosylation of **D3** with **A5**	S18	38. References	S41

(A) Materials and Methods:

All chemicals and solvents were purchased from commercial sources and used directly without further purification. $AuCl_3$ and $AuBr_3$ were purchased from Sigma-Aldrich. All the reactions were carried out under argon or nitrogen atmosphere employing oven dried glassware. Chromatograms were visualized under UV light and by dipping plates into either sulphuric acid in MeOH or anisaldehyde in ethanol, followed by heating. ^{1}H NMR, COSY, HMBC and HMQC spectra were recorded on a 500 MHz NMR spectrometer. Proton chemical shifts are reported in ppm (δ) relative to the internal standard tetramethylsilane (TMS, δ 0.0 ppm) or with the solvent reference relative to TMS employed as the internal standard ($CDCl_3$, δ 7.26 ppm; DMSO-d_6, δ 2.55 ppm). Data are reported as follows: chemical shift (multiplicity [singlet (s), doublet (d), doublet of doublet (dd), triplet (t), quartet (q), and multiplet (m)], coupling constants [Hz], integration and peak identification). All NMR signals were assigned on the basis of ^{1}H NMR, ^{13}C NMR, COSY and HMQC experiments. ^{13}C spectra were recorded with complete proton decoupling. Carbon chemical shifts are reported in ppm (δ) relative to TMS with the respective solvent resonance as the internal standard. All NMR data were collected at 25 °C. The concentration of the compounds for ^{1}H NMR was 5 mg per 0.5 mL and for ^{13}C NMR it was 5-20 mg per 0.5 mL. Melting points were determined using melting point apparatus and are uncorrected. Flash column chromatography was performed using 230-400 mesh silica gel.

X-ray intensity data measurements of freshly grown crystals of **D3** was carried out at 296 K on a Bruker-KAPPA APEX II CCD diffractometer with graphite-monochromatized (MoK = 0.71073Å) radiation. The X-ray generator was operated at 50 kV and 30 mA. Data were collected with scan width of 0.3° at different settings of φ (0°, 90° and 180°) keeping the sample to detector distance fixed at 40 mm and the detector position (2θ) fixed at 24°. The X-ray data collection was monitored by SMART program. All the data were corrected

for Lorentzian polarization and absorption effects using SAINT and SADABS programs. SHELX-97 was used for structure solution and full matrix least-squares refinement on *F*2. Molecular and packing diagrams were generated using Mercury-3.1. Geometrical calculations were performed using SHELXTL and PLATON.

(B) <u>Synthesis of glucopyranoside donors D2, D3, D5, and D11:</u>

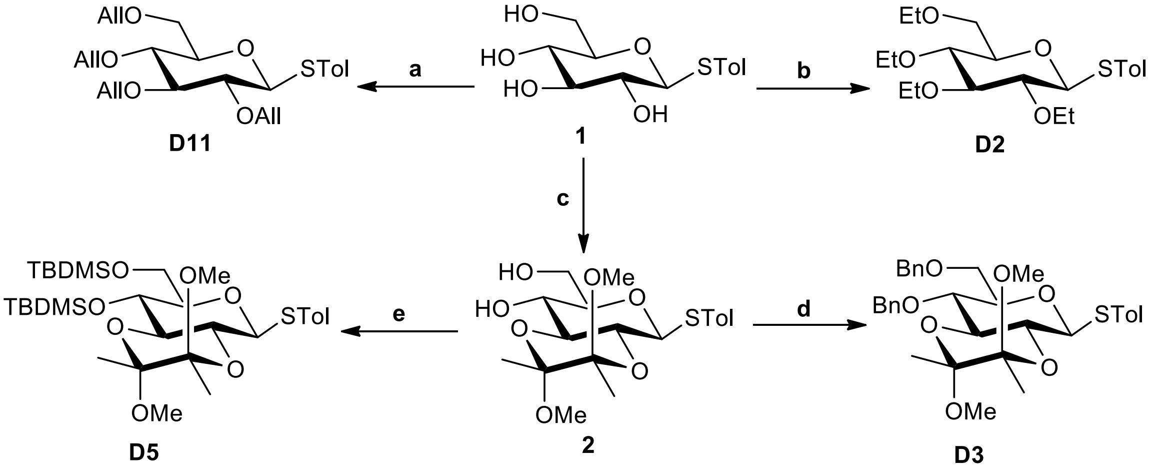

Scheme S1. Reagents and conditions: (a) AllBr, NaH, DMF, 0 °C-rt, 2 h, 93%; (b) EtI, NaH, DMF, 0 °C-rt, 1.5 h, 97%); (c) 2,3-butanedione, $CH(OMe)_3$, CSA, MeOH, 70 °C, 6 h, 45%; (d) BnBr, NaH, DMF, 0 °C-rt, 2 h, 98%; (e) TBDMSCl, imidazole, DMF, rt, 2 h, 95%

i. Synthesis of *p*-tolyl 2,3,4,6-tetra-*O*-allyl-1-thio-β-D-glucopyranoside (D11):

A solution of *p*-tolyl 1-thio-β-D-glucopyranoside (**1**)[1] (1.0 g, 3.5 mmol) in anhydrous DMF (20 mL) was cooled to 0 °C and sodium hydride (0.7 g, 17.5 mmol) was added in two portions. The mixture was stirred at 0 °C for 10 min. Allyl bromide (1.5 mL, 17.5 mmol) was then added slowly and the reaction mixture was stirred for 2 h, slowly warming to room temperature. When TLC showed complete conversion of the starting material, the reaction mixture was cooled to 0 °C and quenched by adding ice. The reaction mixture was extracted with ethyl acetate (2 x 20 mL) and washed with water (2 x 20 mL) followed by brine. The combined organic layer was dried over anhydrous sodium sulphate and concentrated under reduced pressure. The residue thus obtained was purified by flash column chromatography to afford donor **D11** (1.45 g, 93%) as a colourless gum. R_f = 0.33 (EtOAc-hexane, 1:4, v/v); ^{1}H

NMR (500 MHz, $CDCl_3$): δ 2.24 (s, 3H, Ar-CH_3), 3.15 (dd, J = 10.0, 9.0 Hz, 1H, H-2), 3.27-3.28 (m, 2H, H-4, H-5), 3.30-3.33 (m, 1H, H-3), 3.53-3.56 (m, 1H, H-6_A), 3.62-3.64 (m, 1H, H-6_B), 3.92-3.93 (m, 1H, CH_2-CH=CH_2), 3.96-3.97 (m, 1H, CH_2-CH=CH_2), 4.02-4.06 (m, 1H, CH_2-CH=CH_2), 4.12-4.15 (m, 1H, CH_2-CH=CH_2), 4.18-4.27 (m, 4H, CH_2-CH=CH_2), 4.39 (d, J = 12.0 Hz, 1H, H-1), 5.06-5.11 (m, 4H, CH=CH_2), 5.15-5.23 (m, 4H, CH=CH_2), 5.79-5.90 (m, 4H, C*H*=CH_2), 7.00 (d, J = 8.0 Hz, 2H, Ar-*H*), 7.37 (d, J = 8.0 Hz, 2H, Ar-*H*); ^{13}C NMR (125 MHz, $CDCl_3$): δ 21.1, 69.0, 72.3, 73.8, 74.1, 74.4, 77.4, 79.0, 80.3, 86.2, 87.7, 116.6, 116.8, 116.9, 117.2, 129.5, 129.9, 132.5, 134.8, 134.82, 135.0, 137.5. Anal. Calcd for $C_{25}H_{34}O_5S$: C, 67.24; H, 7.67; found: C, 67.40; H, 7.54.

ii. Synthesis of *p*-Tolyl 2,3,4,6-tetra-*O*-ethyl-1-thio-β-D-glucopyranoside (D2):

A solution of tetraol **1** (1.0 g, 3.5 mmol) in anhydrous DMF (20 mL) was cooled to 0 °C and sodium hydride (0.7 g, 17.5 mmol) was added in two portion. The mixture was stirred at room temperature for 10 min. Ethyl iodide (1.4 mL, 17.5 mmol) was then added slowly and the reaction was stirred at room temperature for 2 h. When TLC showed complete conversion of the starting material, the reaction mixture was cooled to 0 °C and quenched by adding ice. The reaction mixture was extracted with ethyl acetate (2 x 20 mL) and washed with water (2 x 20 mL) followed by brine. The combined organic layer was dried over anhydrous sodium sulphate and concentrated under reduced pressure. The residue thus obtained was purified by flash column chromatography to afford donor **D2** (1.35 g, 97%) as white solid. R_f = 0.25 (EtOAc-hexane, 1:4, v/v); Mp: 47-49 °C; ^{1}H NMR (500 MHz, $CDCl_3$): δ 1.09-1.17 (m, 12H, Ethl-CH_3), 2.24 (s, 3H, Ar-CH_3), 3.04 (t, J = 9.0 Hz, 1H, H-2), 3.14-3.16 (m, 1H, H-4), 3.19-3.23 (m, 2H, H-3, H-5), 3.40-3.42 (m, 1H, H-6_A), 3.46-3.52 (m, 2H, H-6_B, 0.5 x OCH_2), 3.54-3.62 (m, 2H, OCH_2), 3.66-3.71 (m, 1H, 0.5 x OCH_2), 3.73-3.78 (m, 4H, 2 x OCH_2), 4.36 (d, J = 10.0 Hz, 1H, H-1), 7.00 (d, J = 8.0 Hz, 2H, Ar-*H*), 7.37 (d, J = 8.0 Hz, 2H, Ar-*H*); ^{13}C NMR (125 MHz, $CDCl_3$): δ 15.2, 15.7, 15.8, 21.0, 66.8, 68.2, 68.8, 68.9, 69.5, 77.9, 79.2,

80.9, 86.6, 87.8, 129.4, 130.2, 132.3, 137.3. Anal. Calcd for $C_{21}H_{34}O_5$ S: C, 63.29; H, 8.60; found: C, 63.44; H, 8.77.

iii. Synthesis of diol 2:

To a solution of tetraol **1** (1.0 g, 3.5 mmol) in anhydrous MeOH (20 mL), trimethyl orthoformate (1 mL), 2,3-butanedione (0.35 mL, 3.9 mmol) and camphorsulfonic acid (0.043 g, 0.18 mmol) were added. The reaction mixture was refluxed for 4 h. When the TLC showed complete conversion of the starting material, the reaction mixture was cooled to room temperature and quenched by adding triethylamine (1 mL). The reaction mixture was concentrated under reduced pressure. The residue thus obtained was dissolved in ethyl acetate (20 mL) and washed with water (2 x 20 mL) followed by brine. The organic layer was dried over anhydrous sodium sulphate and concentrated under reduced pressure. The residue thus obtained was purified by flash column chromatography to afford desired diol **2** (0.63 g, 45%) along with its 3,4-regioisomer (R_f = 0.31; EtOAc-hexane, 1:3, v/v) as colourless foam. Data for diol **2**: R_f = 0.21 (EtOAc-hexane, 1:3, v/v); ^{1}H NMR (500 MHz, $CDCl_3$): δ 1.24 (s, 3H, CH_3), 1.26 (s, 3H, CH_3), 2.23 (s, 3H, Ar-CH_3), 3.15 (s, 3H, OCH_3), 3.20 (s, 3H, OCH_3), 3.28-3.31 (m, 1H, H-5), 3.49 (t, *J* = 9.0 Hz, 1H, H-5), 3.62-3.73 (m, 3H, H-3, H-4, H-6_A), 3.79 (dd, *J* = 12.0, 3.0 Hz, 1H, H-6_B), 4.67 (d, *J* = 10.0 Hz, 1H, H-1), 7.01 (d, *J* = 8.0 Hz, 2H), 7.31 (d, *J* = 8.0 Hz, 2H); ^{13}C NMR (125 MHz, $CDCl_3$): δ 17.5, 17.6, 21.0, 47.9, 48.2, 62.1, 67.4, 68.2, 74.3, 77.2, 80.0, 85.5, 99.6, 100.1, 129.3, 129.5, 129.6, 132.2, 133.4, 137.6. Anal. Calcd for $C_{19}H_{28}O_7S$: C, 56.98; H, 7.05; found: C, 57.22; H, 7.23.

iv. Synthesis of donor D5:

A solution of diol **2** (1.0 g, 2.5 mmol) in anhydrous DMF (20 mL) was cooled to 0 ^{o}C and imidazole (0.42 g, 6.2 mmol) followed by *tert*-butyldimethylsilyl chloride (0.9 g, 6.2 mmol) were added. The reaction was stirred at room temperature for 2 h. When the TLC showed complete conversion of the starting material, the reaction mixture was quenched by adding

ice. The reaction mixture was extracted with ethyl acetate (2 x 20 mL) and washed successively with aqueous sodium bicarbonate (20 mL), water and brine. The combined organic layer was dried over anhydrous sodium sulphate and concentrated under reduced pressure. The residue thus obtained was purified by flash column chromatography to afford donor **D5** (1.5 g, 95%) as a colourless gum. R_f = 0.51 (EtOAc-hexane, 1:9, v/v); ^{1}H NMR (500 MHz, $CDCl_3$): δ -0.03 (s, 3H, SiCH_3), -0.0002 (s, 3H, SiCH_3), 0.004 (s, 3H, SiCH_3), 0.07 (s, 3H, SiCH_3), 0.78 (s, 9H, ^{t}BuCH_3), 0.81 (s, 9H, ^{t}BuCH_3), 1.20 (s, 3H, CH_3), 1.23 (s, 3H, CH_3), 2.22 (s, 3H, Ar-CH_3), 3.12 (s, 3H, OCH_3), 3.17 (s, 3H, OCH_3), 3.19-3.20 (m, 1H, H-5), 3.47 (t, J = 9.0 Hz, 1H, H-2), 3.53-3.61 (m, 2H, H-3, H-4), 3.66 (dd, J = 11.0, 4.0 Hz, 1H, H-6_A), 3.75 (dd, J = 11.0, 2.0 Hz, 1H, H-6_B), 4.62 (d, J = 10.0 Hz, 1H, H-1), 6.96 (d, J = 8.0 Hz, 2H, Ar-H), 7.35 (d, J = 8.0 Hz, 2H, Ar-H); ^{13}C NMR (125 MHz, $CDCl_3$): δ -5.3, -5.1, -4.9, -3.5, 17.4, 17.6, 17.9, 18.4, 21.0, 25.8, 25.9, 25.93, 25.98, 47.9, 48.0, 62.1, 67.8, 68.3, 74.7, 76.7, 77.0, 77.2, 81.9, 85.4, 99.5, 99.9, 129.3, 129.4, 130.3, 131.9, 133.0, 137.0. Anal. Calcd for $C_{31}H_{56}O_7SSi_2$: C, 59.19; H, 8.97; found: C, 59.03; H, 9.11.

v. Synthesis of donor D3:

A solution of diol **2** (1.0 g, 2.5 mmol) in anhydrous DMF (20 mL) was cooled to 0 °C and sodium hydride (0.3 g, 7.5 mmol) was added in one portion. The mixture was stirred at room temperature for 10 min. Benzyl bromide (0.9 mL, 7.5 mmol) was then added slowly and the reaction mixture was stirred at room temperature for 2 h. When the TLC showed complete conversion of the starting material, the reaction mixture was cooled to 0 °C and quenched by adding ice. The reaction mixture was extracted with ethyl acetate (2 x 20 mL) and washed with water (2 x 20 mL) followed by brine. The combined organic layer was dried over anhydrous sodium sulphate and concentrated under reduced pressure. The residue thus obtained was purified by flash column chromatography to afford donor **D3** (1.42 g, 98%) as a white solid. R_f = 0.45 (EtOAc-hexane, 1:4, v/v); Mp: 110-112 °C; ^{1}H NMR (500 MHz,

$CDCl_3$): δ 1.27 (s, 3H, CH_3), 1.28 (s, 3H, CH_3), 2.20 (s, 3H, Ar-CH_3), 3.14 (s, 3H, OCH_3), 3.21 (s, 3H, OCH_3), 3.41-3.44 (m, 1H, H-5), 3.58-3.64 (m, 3H, H-2, H-4, H-6_A), 3.68 (d, *J* = 11.0 Hz, 1H, H-6_B), 3.83 (t, *J* = 9.0 Hz, 1H, H-3), 4.42 (d, *J* = 12.0 Hz, 1H, 0.5 x OCH_2Ph), 4.50 (d, *J* = 11.0 Hz, 2H, 1 x OCH_2Ph), 4.60 (d, *J* = 10.0 Hz, 1H, H-1), 4.84 (d, *J* = 11.0 Hz, 1H, 0.5 x OCH_2Ph), 6.93 (d, *J* = 8.0 Hz, 2H, Ar-*H*), 7.17-7.24 (m, 10H, Ar-*H*), 7.38 (d, *J* = 8.0 Hz, 2H, Ar-*H*); ^{13}C NMR (125 MHz, $CDCl_3$): δ 16.6, 16.7, 20.0, 46.8, 47.1, 67.1, 68.1, 72.3, 73.6, 73.9, 74.4, 78.4, 84.1, 98.5, 99.0, 126.4, 126.6, 126.7, 126.9, 127.2, 127.3, 128.4, 131.4, 136.4, 137.3, 137.39. Anal. Calcd for $C_{33}H_{40}O_7S$: C, 68.25; H, 6.94; found: C, 68.37; H, 6.78.

The donor **D3** was crystallized from the mixture of ethyl acetate: petroleum ether (1:4, v/v) by slow evaporation.

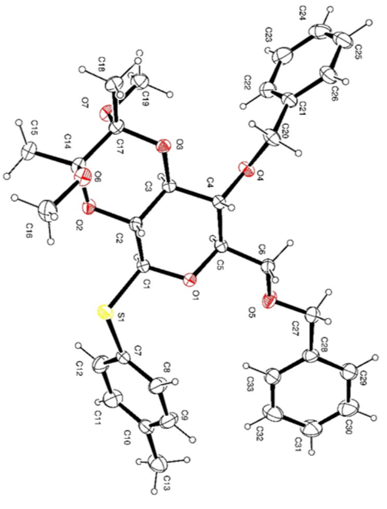

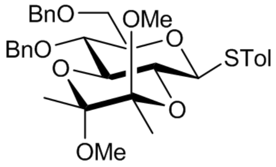

D3

Figure S1. ORTEP diagram with 30% probability ellipsoids and molecular structure of donor **D3**.

CCDC: 1430710, Refined formula: $C_{33}H_{40}O_7S$, Formula weight: M = 580.71, colourless needle, 0.20 x 0.15 x 0.10 mm^3, Monoclinic, space group: P2(1), Unit cell dimensions and volume: a = 11.0077(6), b = 9.7685(6), c = 14.9709(7) Å, V = 1910.3(14) Å^3 , No of formula units in the unit cell Z = 2, T = 296(2) K, 2θmax = 42.90°, Calculated density ρcalcd: (g cm^{-3}) = 1.230, F(000) = 620, Linear absorption coefficient μ: 0.149 mm^{-1}, 14426 reflections

collected, 6875 unique reflections (R_{int} = 0.1106), multi-scan absorption correction, T_{min} = 0.971, T_{max} = 0.985, number of parameters = 370, number of restraints = 1, GoF = 0.895, R1 = 0.0497, wR2 = 0.1087, R indices based on 3842 reflections with I >2\s(I) (refinement on F2). $\Delta\rho_{max}$ = 0.000, $\Delta\rho_{min}$ = 0.000 (eÅ^{-3}).

(C) General procedure for glycosylation:

A suspension of donor (0.1-0.5 mmol), acceptor (1.1-1.3 equiv) and 4 Å molecular sieves (0.3-0.5 g) in anhydrous dichloromethane (5 mL) was stirred at room temperature for 15 min under nitrogen atmosphere. $AuCl_3$ (3-5 mol%) was then added and the reaction mixture was then stirred at room temperature. The reaction was monitored by TLC. The reaction was quenched by adding aqueous $NaHCO_3$ solution, filtered through a Celite bed and washed with dichloromethane. The filtrate was then partitioned and the organic layer was washed successively with water and brine, dried over anhydrous sodium sulphate, filtered and concentrated under reduced pressure. The crude product thus obtained was purified by flash column chromatography using ethyl acetate and petroleum ether as eluents. All the new products were fully characterized by spectroscopic techniques and elemental analysis. In case of glycosylations with acid stable acceptors and donors the reaction mixture was partially evaporated and chromatographed directly without work up.

1. Glycosylation of donor D1[2] with acceptor A1:

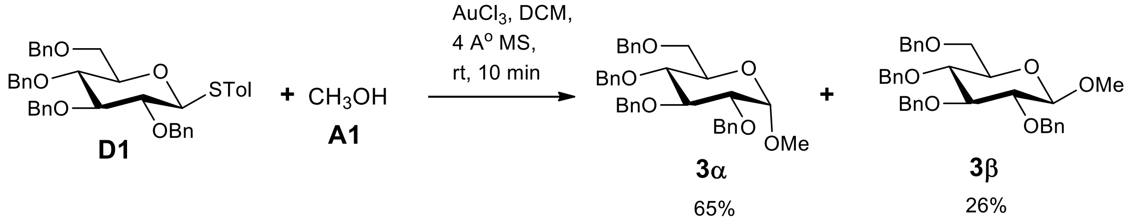

The treatment of donor **D1** (0.15 g, 0.23 mmol) with acceptor **A1** (0.047 mL, 1.16 mmol) in presence of 3 mol% of $AuCl_3$ gave 78 mg (65%) of methyl 2,3,4,6-tetra-*O*-benzyl-α-D-glucopyranoside (**3α**)[3] and 31 mg (26%) of methyl 2,3,4,6-tetra-*O*-benzyl-β-D-

glucopyranoside (**3β**)[4] as colourless gums. The spectral data is identical to that of reported data. **3α**: R_f = 0.41 (EtOAc-hexane, 1:5, v/v); ^{1}H NMR (500 MHz, $CDCl_3$): δ 3.30 (s, 3H, OCH_3), 3.48 (dd, *J* = 10.0, 3.5 Hz, 1H, H-2), 3.53-3.57 (m, 2H, H-4, H-6_A), 3.62-3.67 (m, 2H, H-5, H-6_B), 3.90 (t, *J* = 10.0, Hz, 1H, H-3), 4.38-4.42 (m, 2H, 1 x OCH_2Ph), 4.52 (d, *J* = 12.0, Hz, 1H, 0.5 x OCH_2Ph), 4.55 (d, *J* = 3.5 Hz, 1H, H-1), 4.58 (d, *J* = 12.0, Hz, 1H, 0.5 x OCH_2Ph), 4.70-4.76 (m, 3H, 1.5 x OCH_2Ph), 4.90 (d, *J* = 11.0 Hz, 1H, 0.5 x OCH_2Ph), 7.05-7.27 (m, 20H, Ar-*H*); **3β**: R_f = 0.43 (EtOAc-hexane, 1:5, v/v); ^{1}H NMR (500 MHz, $CDCl_3$): δ 3.34-3.40 (m, 2H, H-2, H-5), 3.50 (s, 3H, OCH_3), 3.52-3.57 (m, 2H, H-3, H-4) 3.59-3.63 (m, 1H, H-6_A), 3.66-3.69 (m, 1H, H-6_B), 4.23 (d, *J* = 7.8, Hz, 1H, H-1), 4.44-4.49 (m, 2H, 1 x OCH_2Ph), 4.54 (d, *J* = 12.0, Hz, 1H, 0.5 x OCH_2Ph), 4.63 (d, *J* = 11.0 Hz, 1H, 0.5 x OCH_2Ph), 4.70-4.75 (m, 2H, 1 x OCH_2Ph), 4.83-4.85 (m, 2H, 1 x OCH_2Ph), 7.08 (d, *J* = 6.0, Hz, 2H, Ar-H), 7.17-7.26 (m, 18H, Ar-H).

2. Glycosylation of donor D1 with acceptor A2:

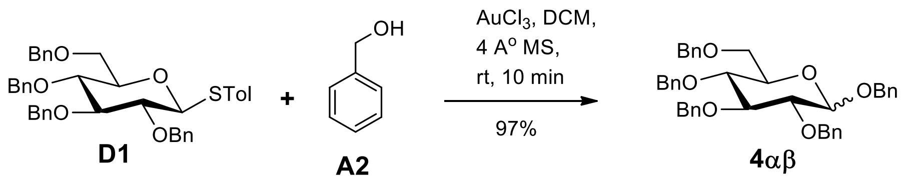

The treatment of donor **D1** (0.1 g, 0.15 mmol) with acceptor **A2** (0.048 mL, 0.46 mmol) in presence of 5 mol% of $AuCl_3$ gave 94 mg (97%, *α/β* = 2.5:1) of 1,2,3,4,6-penta-*O*-benzyl-D-glucopyranosides (**4αβ**)[5] as colourless gum. R_f = 0.44 (EtOAc-hexane, 1:5, v/v); ^{1}H NMR (500 MHz, $CDCl_3$): δ 3.49-3.53 (m, 0.4H), 3.58-3.62 (m, 2.5H), 3.67-3.71 (m, 1.8H), 3.72-3.75 (m, 1.4H), 3.78-3.87 (m, 1.5H), 4.08 (t, *J* = 9.0 Hz, 1H), 4.50-4.52 (m, 2H), 4.58-4.63 (m, 4H), 4.65-4.66 (m, 0.7H), 4.70-4.75 (m, 3H), 4.81-4.89 (m, 3.9H), 4.95-4.98 (m, 0.6H), 5.02 (t, *J* = 11.0, Hz, 1.6H), 7.05-7.27 (m, 35H, Ar-*H*).

3. Glycosylation of donor D1 with acceptor A3:

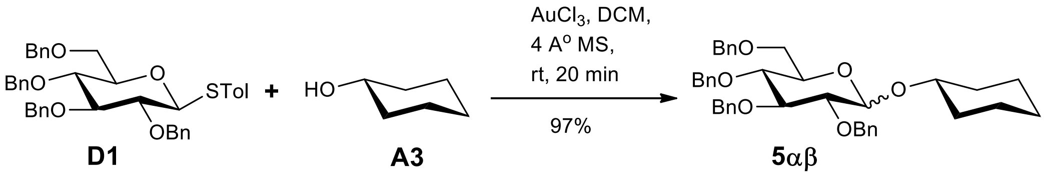

The treatment of donor **D1** (0.2 g, 0.31 mmol) with acceptor **A3** (0.066 mL, 0.62 mmol) in presence of 5 mol% of $AuCl_3$ gave 186 mg (97%, *α/β* = 3.3:1) of cyclohexyl 2,3,4,6-tetra-*O*-benzyl-D-glucopyranosides (**5αβ**)[5] as colourless gum. R_f = 0.43 (EtOAc-hexane, 1:5, v/v); ^{1}H NMR (500 MHz, $CDCl_3$): δ 1.22-1.33 (m, 4H, cyclohex-H), 1.36-1.47 (m, 1.3H, cyclohex-H), 1.44-1.52 (m, 1.5H, cyclohex-H), 1.55-1.60 (m, 1.5H, cyclohex-H), 1.75-1.81 (m, 2.6H, cyclohex-H), 1.87-1.98 (m, 2.2H, cyclohex-H), 2.02-2.06 (m, 0.3H, cyclohex-H), 3.46-3.50 (m, 0.7H), 3.56-3.61 (m, 2.2H), 3.64-3.70 (m, 2.6H), 3.73-3.78 (m, 1.6H), 3.90-3.93 (m, 1H), 4.03 (t, *J* = 9.0 Hz, 1H), 4.48-4.51 (m, 2H), 4.53-4.55 (m, 0.3H), 4.56-4.60 (m, 0.7H), 4.63-4.65 (m, 1.3H), 4.67-4.70 (m, 1H), 4.73-4.76 (m, 1H), 4.78-4.80 (m, 0.5H), 4.82-4.84 (m, 0.8H), 4.85-4.87 (m, 1.6H), 4.94-4.96 (m, 0.3H), 4.9 (d, *J* = 4.0 Hz, 1H), 5.02-5.04 (m, 1.2H), 7.16-7.39 (m, 26H, Ar-*H*).

4. Glycosylation of donor D2 with acceptor A2:

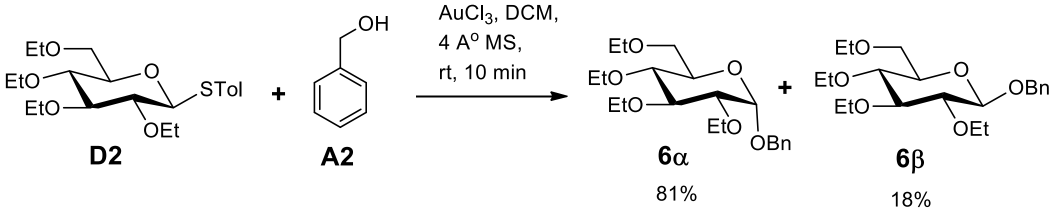

The treatment of donor **D2** (0.2 g, 0.5 mmol) with acceptor **A2** (0.26 mL, 2.5 mmol) in presence of 5 mol% of $AuCl_3$ gave 155 mg (81%) of benzyl 2,3,4,6-tetra-*O*-ethyl-α-D-glucopyranoside (**6α**) and 34 mg (18%) of benzyl 2,3,4,6-tetra-*O*-ethyl-β-D-glucopyranoside (**6β**) as colourless gums. **6α**: R_f = 0.40 (EtOAc-hexane, 1:5, v/v); ^{1}H NMR (500 MHz, $CDCl_3$): δ 1.09-1.15 (m, 12H, 4 x CH_3), 3.21 (dd, *J* = 9.6, 3.6 Hz, 1H, H-2), 3.27 (t, *J* = 9.6 Hz, 1H, H-3), 3.38-3.41 (m, 1H, 0.5 x OCH_2CH_3), 3.44-3.52 (m, 5H, H-5, H-6_A, 1.5 x OCH_2CH_3), 3.54-3.59 (m, 3H, H-4, H-6', 0.5 x OCH_2CH_3), 3.69-3.76 (m, 1H, 0.5 x

OCH_2CH_3), 3.77-3.83 (m, 2H, OCH_2CH_3), 4.52 (d, *J* = 12.4 Hz, 1H, 0.5 x OCH_2Ph), 4.3 (d, *J* = 12.4 Hz, 1H, 0.5 x OCH_2Ph), 4.85 (d, *J* = 3.6 Hz, 1H, H-1), 7.19-7.32 (m, 5H, Ar-H); ^{13}C NMR (125 MHz, $CDCl_3$): δ 15.1, 15.5, 15.7, 15.9, 66.6, 66.8, 68.2, 68.6, 68.8, 69.0, 70.4, 77.6, 80.2, 81.5, 95.8, 126.9, 127.5, 127.6, 128.23, 128.24, 137.4; **6β**: R_f = 0.42 (EtOAc-hexane, 1:5, v/v); ^1H NMR (500 MHz, $CDCl_3$): δ 1.10-1.15 (m, 12H, 4 x CH_3), 3.09 (t, *J* = 9.6 Hz, 1H, H-2), 3.17-3.22 (m, 3H, H-3, H-4, H-5), 3.43-3.48 (m, 1H, 0.5 x OCH_2CH_3), 3.51-3.59 (m, 3H, H-6$_A$, 1 x OCH_2CH_3), 3.62-3.64 (m, 2H, H-6$_B$, 0.5 x OCH_2CH_3), 3.68-3.72 (m, 1H, 0.5 x OCH_2CH_3), 3.73-3.85 (m, 3H, 1.5 x OCH_2CH_3), 4.27 (d, *J* = 7.7 Hz, 1H, H-1), 4.55 (d, *J* = 12.1 Hz, 1H, 0.5 x OCH_2Ph), 4.84 (d, *J* = 12.1 Hz, 1H, 0.5 x OCH_2Ph), 7.19-7.25 (m, 5H, Ar-H); ^{13}C NMR (125 MHz, $CDCl_3$): δ 15.1, 15.6, 15.7, 15.8, 66.9, 68.2, 68.3, 68.8, 69.5, 70.9, 75.0, 78.0, 82.1, 84.1, 102.5, 127.5, 127.6, 128.2, 137.7. Anal. Calcd for $C_{21}H_{34}O_6$: C, 65.94; H, 8.96; found: C, 66.12; H, 9.17.

5. Glycosylation of donor D3 with acceptor A1:

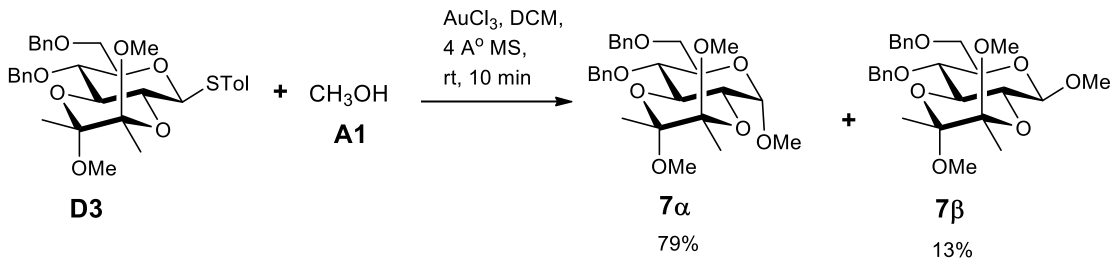

The treatment of donor **D3** (0.1 g, 0.17 mmol) with acceptor **A1** (0.069 mL, 1.7 mmol) in presence of 3 mol% of $AuCl_3$ gave 66 mg (79%) of methyl glycoside **7α** as a colourless gum and 11 mg (13%) of methyl glycoside **7β** as a white solid. **7α**: R_f = 0.35 (EtOAc-hexane, 1:4, v/v); ^1H NMR (500 MHz, $CDCl_3$): δ 1.27 (s, 3H, CH_3), 1.28 (s, 3H, CH_3), 3.20 (s, 3H, OCH_3), 3.22 (s, 3H, OCH_3), 3.32 (s, 3H, OCH_3), 3.58-3.59 (m, 1H, H-5), 3.66-3.69 (m, 3H, H-4, H-6$_A$, H-6$_B$), 3.75 (dd, *J* = 10.0, 3.5 Hz, 1H, H-2), 4.13 (dd, *J* = 10.0, 8.0 Hz, 1H, H-3), 4.40-4.43 (m, 2H, 1 x OCH_2Ph), 4.56 (d, *J* = 12.0 Hz, 1H, 0.5 x OCH_2Ph), 4.70 (d, *J* = 3.5, 1H, H-1), 4.84 (d, *J* = 12.0 Hz, 1H, 0.5 x OCH_2Ph), 7.12-7.27 (m, 10H, Ar-H); ^{13}C NMR

(125 MHz, $CDCl_3$): δ 17.7, 18.0, 47.8, 47.9, 54.9, 68.3, 70.5, 70.6, 73.4, 74.8, 75.0, 77.2, 97.9, 99.3, 99.8, 127.6, 127.8, 127.9, 128.3, 128.34, 138.1, 138.5; **7β**: R_f = 0.37 (EtOAc-hexane, 1:4, v/v); Mp: 130-132 °C; ^{1}H NMR (500 MHz, $CDCl_3$): δ 1.26 (s, 3H, CH_3), 1.29 (s, 3H, CH_3), 3.20 (s, 3H, OCH_3), 3.23 (s, 3H, OCH_3), 3.41-3.48 (m, 4H, H-5, OCH_3), 3.50 (dd, J = 8.0, 2.0 Hz, 1H, H-2), 3.58-3.68 (m, 3H, H-4, H-6_A, H-6_B), 3.81 (t, J = 10.0 Hz, 1H, H-3), 4.32 (d, J = 8.0, 1H, H-1), 4.44-4.47 (m, 2H, 1 x OCH_2Ph), 4.54 (d, J = 12.0 Hz, 1H, 0.5 x OCH_2Ph), 4.84 (d, J = 12.0 Hz, 1H, 0.5 x OCH_2Ph), 7.14-7.26 (m, 10H, Ar-H); ^{13}C NMR (125 MHz, $CDCl_3$): δ 17.6, 17.8, 47.9, 48.0, 56.8, 68.9, 69.4, 73.4, 73.7, 74.9, 74.94, 75.6, 77.2, 99.42, 99.48, 101.3, 127.5, 127.7, 128.0, 128.3, 138.2, 138.3. Anal. Calcd for $C_{27}H_{36}O_8$: C, 66.38; H, 7.43; found: C, 66.19; H, 7.23.

6. Glycosylation of donor D3 with acceptor A2:

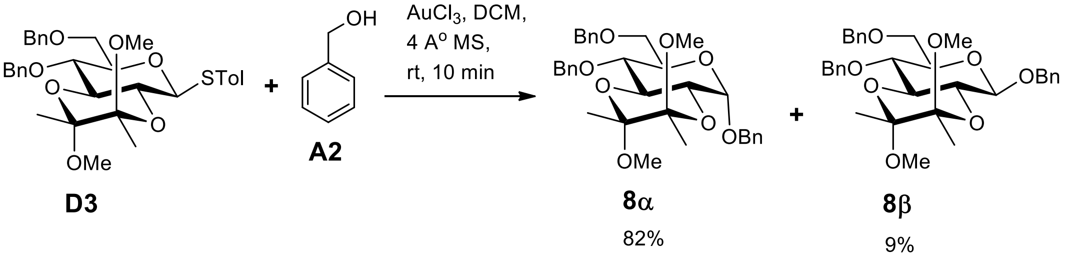

The treatment of donor **D3** (0.2 g, 0.34 mmol) with acceptor **A2** (0.18 mL, 1.72 mmol) in presence of 3 mol% of $AuCl_3$ gave 161 mg (82%) of benzyl glycoside **8α** and 18 mg (9%) of benzyl glycoside **8β** were obtained as colourless gum. **8α**: R_f = 0.36 (EtOAc-hexane, 1:4, v/v); ^{1}H NMR (500 MHz, $CDCl_3$): δ 1.27 (s, 6H, 2CH_3), 3.17 (s, 3H, OCH_3), 3.22 (s, 3H, OCH_3), 3.38 (dd, J = 10.0, 1.5 Hz, 1H, H-6_A), 3.59 (dd, J = 10.0, 3.5 Hz, 1H, H-6_B), 3.62-3.69 (m, 1H, H-5), 3.70 (t, J = 10.0, Hz, 1H, H-4), 3.75 (dd, J = 10.0, 3.5 Hz, 1H, H-2), 4.17 (dd, J = 10.0, 9.0 Hz, 1H, H-3), 4.37 (d, J = 12.0, Hz, 1H, 0.5 x OCH_2Ph), 4.40 (d, J = 11.0 Hz, 1H, 0.5 x OCH_2Ph), 4.53 (d, J = 12.0 Hz, 1H, 0.5 x OCH_2Ph), 4.61-4.62 (m, 2H, 1 x OCH_2Ph), 4.82 (d, J = 11.0 Hz, 1H, 0.5 x OCH_2Ph), 4.88 (d, J = 3.5, 1H, H-1), 7.11-7.31 (m, 15H, Ar-H); ^{13}C NMR (125 MHz, $CDCl_3$): δ 17.6, 18.0, 47.8, 47.9, 68.2, 68.3, 69.8, 70.6,

71.0, 73.4, 74.9, 75.1, 96.6, 99.3, 99.8, 126.98, 127.5, 127.6, 127.64, 127.8, 128.0, 128.1, 128.30, 128.32, 128.5, 137.5, 138.1, 138.5; **8β**: R_f = 0.38 (EtOAc-hexane, 1:4, v/v); ^{1}H NMR (500 MHz, $CDCl_3$): δ 1.36 (s, 3H, CH_3), 1.38 (s, 3H, CH_3), 3.31 (s, 3H, OCH_3), 3.33 (s, 3H, OCH_3), 3.51-3.54 (m, 1H, H-5), 3.67-3.73 (m, 3H, H-2, H-4, H-6_A), 3.76 (dd, *J* = 10.0, 3.5 Hz, 1H, H-6_B), 3.91 (t, *J* = 10.0, Hz, 1H, H-3), 4.55-4.58 (m, 2H, H-1, 0.5 x OCH_2Ph), 4.60-4.65 (m, 2H, 1 x OCH_2Ph), 4.69 (d, *J* = 12.0, Hz, 1H, 0.5 x OCH_2Ph), 4.95 (dd, *J* = 11.0 10.0 Hz, 2H, 1 x OCH_2Ph), 7.24-7.41 (m, 15H, Ar-H); ^{13}C NMR (125 MHz, $CDCl_3$): δ 17.6, 17.8, 47.8, 47.9, 69.5, 69.0, 70.7, 73.4, 73.7, 74.8, 75.6, 96.46, 99.47, 99.9, 127.3, 127.41, 127.48, 127.5, 127.7, 128.0, 128.1, 128.30, 137.8, 138.32, 138.35. Anal. Calcd for $C_{33}H_{40}O_8$: C, 70.19; H, 7.14; found: C, 70.46; H, 6.98.

7. **Glycosylation of donor D3 with acceptor A4:**

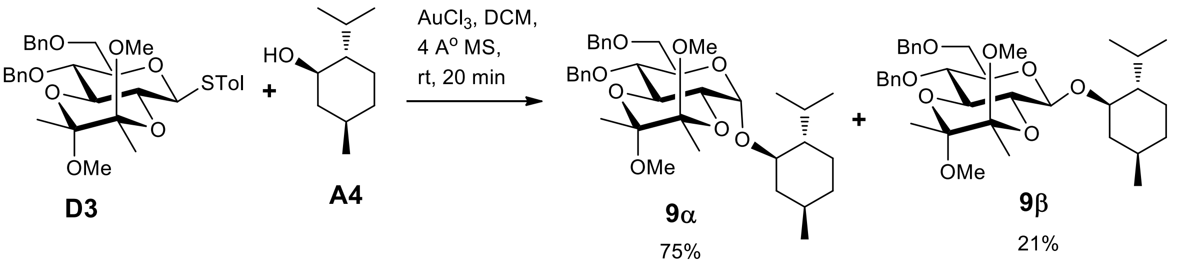

The treatment of donor **D3** (0.1 g, 0.17 mmol) with acceptor **A4** (0.032 g, 0.20 mmol) in presence of 3 mol% of $AuCl_3$ gave 79 mg (75%) of menthyl glycoside **9α** and 22 mg (21%) of menthyl glycoside **9β** as colourless gum. **9α**: R_f = 0.37 (EtOAc-hexane, 1:4, v/v); ^{1}H NMR (500 MHz, $CDCl_3$): δ 0.70 (d, *J* = 7.0 Hz, 3H, mnth-CH_3), 0.77 (d, *J* = 7.0 Hz, 4H, mnth-C*H* and mnth-CH_3), 0.80 (d, *J* = 7.0 Hz, 3H, mnth-CH_3), 0.90-0.92 (m, 2H, mnth-CH_2), 1.16 (s, 3H, BDA-CH_3), 1.18 (s, 2H, mnth-CH_2), 1.25 (s, 3H, BDA-CH_3), 1.50-1.52 (m, 2H, mnth-CH_2), 2.01-2.10 (m, 1H, mnth-C*H*), 2.20-2.30 (m, 1H, mnth-C*H*), 3.16 (s, 3H, OCH_3), 3.18 (s, 3H, OCH_3), 3.20-3.21 (m, 1H, mnth-C*H*), 3.56 (dd, *J* = 10.0, 1.0 Hz, 1H, H-5), 3.60-3.69 (m, 3H, H-2, H-4, H-6_A), 3.86 (d, *J* = 10.0 Hz, 1H, H-6_B), 4.14 (t, *J* = 10.0 Hz, 1H, H-3), 4.39 (t, *J* = 11.0 Hz, 2H, 1 x OCH_2Ph), 4.57 (d, 1H, *J* = 12.0 Hz, 0.5 x OCH_2Ph), 4.79 (d, *J* = 3.0

Hz, 1H, H-1), 4.83 (d, J = 12.0 Hz, 1H, 0.5 x OCH_2Ph), 7.11-7.26 (m, 10H, Ar-H); ^{13}C NMR (125 MHz, $CDCl_3$): δ 16.8, 17.4, 18.0, 20.4, 22.2, 24.0, 25.5, 31.5, 34.4, 42.7, 47.6, 47.7, 48.6, 68.6, 68.7, 70.4, 71.1, 73.3, 74.8, 75.2, 81.8, 98.6, 99.2, 99.3, 127.5, 127.57, 127.8, 127.9, 128.2, 138.2, 138.6; **9β**: R_f = 0.39 (EtOAc-hexane, 1:4, v/v); ^{1}H NMR (500 MHz, $CDCl_3$): δ 0.72 (d, J = 7.0 Hz, 3H, mnth-CH_3), 0.80-0.82 (m, 7H, mnth-CH_3), 0.83-0.85 (m, 2H, mnth-CH_2), 1.17-1.18 (m, 2H, mnth-CH_2), 1.23 (s, 3H, BDA-CH_3), 1.27 (s, 3H, BDA-CH_3), 1.54 (d, J = 12.0 Hz, 2H, mnth-CH_2), 2.02 (d, J = 12.0 Hz, 1H, mnth-CH), 2.21-2.23 (m, 1H, mnth-CH), 3.20 (s, 3H, OCH_3), 3.22 (s, 3H, OCH_3), 3.36-3.45 (m, 3H, H-2, H-5, mnth-CH), 3.55 (t, J = 10.0 Hz, 1H, H-4), 3.61-3.62 (m, 2H, H-6$_A$, H-6$_B$), 3.79 (t, J = 10.0 Hz, 1H, H-3), 4.45-4.46 (m, 2H, H-1, 0.5 x OCH_2Ph), 4.51-4.54 (m, 2H, 1 x OCH_2Ph), 4.84 (d, 1H, J = 11.0 Hz, 0.5 x OCH_2Ph), 7.18-7.25 (m, 10H, Ar-H); ^{13}C NMR (125 MHz, $CDCl_3$): δ 16.1, 17.6, 20.9, 22.2, 23.4, 25.4, 31.5, 34.4, 40.7, 47.7, 47.8, 69.5, 69.54, 73.5, 74.0, 74.8, 74.9, 75.5, 78.0, 98.2, 99.4, 99.43, 127.3, 127.5, 127.6, 128.0, 128.2, 128.3, 138.4, 138.5. Anal. Calcd for $C_{36}H_{52}O_8$: C, 70.56; H, 8.55; found: C, 70.29; H, 8.71.

8. Glycosylation of donor D3 with acceptor A5[6]:

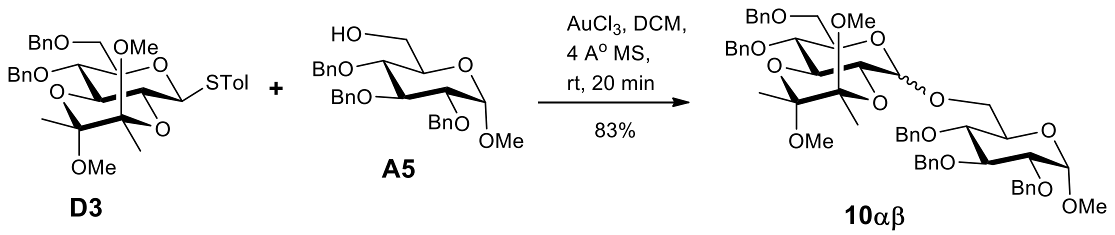

The treatment of donor **D3** (0.1 g, 0.17 mmol) with acceptor **A5** (0.088 g, 0.18 mmol) in presence of 3 mol% of $AuCl_3$ gave 132 mg (83%, *α/β* = 1.4:1) of disaccharide **10αβ** as colourless gum. R_f = 0.34 (EtOAc-hexane, 1:3, v/v); ^{1}H NMR (500 MHz, $CDCl_3$): δ 1.13-1.26 (m, 14.5H), 3.11-3.12 (m, 4.8H), 3.19-3.20 (m, 4.9H), 3.28-3.30 (m, 4.8H), 3.40-3.46 (m, 2.8H), 3.48-3.61 (m, 5.6H), 3.62-3.80 (m, 9.3H), 3.89 (t, J = 9.0 Hz, 1.6H), 4.02-4.05 (m, 1H), 4.15 (t, J = 10.0 Hz, 1H), 4.34-4.42 (m, 2.5H), 4.43-4.48 (m, 2.3H), 4.50-4.54 (m, 2.6H), 4.56-4.61 (m, 2.6H), 4.66-4.71 (m, 2.5H), 4.73-4.83 (m, 4.8H), 4.86-4.90 (m, 1.6H),

4.92 (d, J = 3.0 Hz, 0.8H), 7.09-7.23 (m, 43H, Ar-H); ^{13}C NMR (125 MHz, $CDCl_3$): δ 17.6, 17.8, 17.9, 47.6, 47.8, 47.87, 54.9, 55.0, 68.3, 68.4, 69.1, 70.3, 70.38, 71.0, 73.1, 73.3, 73.7, 74.7, 74.9, 75.0, 75.5, 77.9, 79.8, 82.2, 97.6, 127.4, 127.5, 127.53, 127.56, 127.6, 127.65, 127.7, 127.8, 127.86, 127.9, 128.0, 128.03, 128.09, 128.1, 128.2, 128.3, 128.34, 128.38, 128.4, 138.1, 138.2, 138.21, 138.22, 138.3. Anal. Calcd for $C_{54}H_{64}O_{13}$: C, 70.42; H, 7.00; found: C, 70.17; H, 7.28.

9. Glycosylation of donor D3 with acceptor A6[7]:

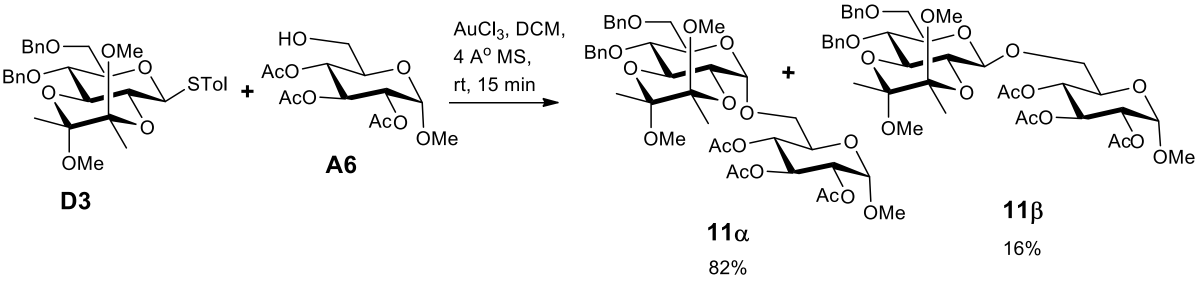

The treatment of donor **D3** (0.2 g, 0.34 mmol) with acceptor **A6** (0.11 g, 0.34 mmol) in presence of 3 mol% of $AuCl_3$ gave 218 mg (82%) of disaccharide **11α** and 43 mg (16%) of disaccharide **11β** as colourless gum. **11α**: R_f = 0.21 (EtOAc-hexane, 1:3, v/v); ^{1}H NMR (500 MHz, $CDCl_3$): δ 1.22 (s, 3H, CH_3), 1.24 (s, 3H, CH_3), 1.92 (s, 3H, COCH_3), 1.94 (s, 3H, COCH_3), 1.99 (s, 3H, COCH_3), 3.15 (s, 3H, OCH_3), 3.18 (s, 3H, OCH_3), 3.31 (s, 3H, OCH_3), 3.49 (dd, J = 11.0, 2.0 Hz, 1H), 3.54 (dd, J = 11.0, 2.0 Hz, 1H), 3.64-3.67 (m, 2H), 3.69-3.73 (m, 2H), 3.81 (d, J = 10.0, Hz 1H), 3.98 (dt, J = 10.0, 7.0, 2.0 Hz, 1H), 4.10 (t, J = 10.0 Hz, 1H), 4.37-4.41 (m, 2H), 4.54 (d, J = 12.0 Hz, 1H, 0.5 x OCH_2Ph), 4.76-4.86 (m, 5H), 5.38 (t, J = 10.0 Hz, 1H), 7.11 (d, J = 7.0 Hz, 2H, Ar-H), 7.17-7.25 (m, 8H, Ar-H); ^{13}C NMR (125 MHz, $CDCl_3$): δ 17.6, 17.9, 20.6, 20.7, 47.6, 47.9, 55.0, 66.5, 67.9, 68.2, 68.3, 69.7, 70.2, 70.3, 70.9, 71.0, 73.4, 74.7, 74.8, 77.2, 96.1, 96.8, 99.2, 99.7, 127.5, 127.6, 127.7, 127.8, 128.2, 128.3, 138.0, 138.7, 169.8, 170.0, 170.1; **11β**: R_f = 0.23 (EtOAc-hexane, 1:3, v/v); ^{1}H NMR (500 MHz, $CDCl_3$): δ 1.19 (s, 3H, CH_3), 1.27 (s, 3H, CH_3), 1.91 (s, 3H, COCH_3), 1.92 (s, 3H, COCH_3), 1.99 (s, 3H, COCH_3), 3.20 (s, 3H, OCH_3), 3.21 (s, 3H, OCH_3), 3.32 (s, 3H,

OCH_3), 3.41 (dd, *J* = 9.0, 4.0 Hz, 1H), 3.49 (t, *J* = 8.0 Hz, 1H), 3.55-3.66 (m, 4H), 3.76-3.80 (m, 1H), 3.84 (d, *J* = 11.0 Hz 1H), 3.94 (t, *J* = 9.0 Hz, 1H), 4.40-4.45 (m, 3H), 4.52 (d, *J* = 12.0 Hz, 1H), 4.79-4.90 (m, 4H), 5.37 (t, *J* = 10.0 Hz, 1H), 7.13-7.25 (m, 10H, Ar-*H*); ^{13}C NMR (125 MHz, $CDCl_3$): δ 17.4, 17.8, 20.6, 20.7, 20.73, 47.8, 54.9, 68.4, 68.46, 68.9, 69.1, 69.3, 70.4, 70.9, 73.4, 73.6, 74.6, 74.8, 75.6, 96.2, 99.3, 99.4, 100.9, 127.5, 127.7, 128.0, 128.3, 138.2, 138.29, 169.5, 170.1. Anal. Calcd for $C_{39}H_{52}O_{16}$: C, 60.30; H, 6.75; found: C, 60.51; H, 6.94.

10. Glycosylation of donor D4[8] with acceptor A3:

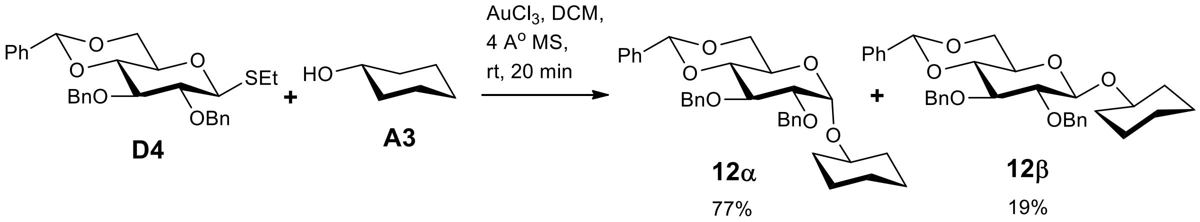

Treatment of donor **D4** (0.2 g, 0.40 mmol) with acceptor **A3** (0.064 mL, 0.60 mmol) in presence of 3 mol% of $AuCl_3$ gave 166 mg (77%) of cyclohexyl 2,3-di-*O*-benzyl-4,6-*O*-benzylidene-α-D-glucopyranoside (**12α**) and 41 mg (19%) of cyclohexyl 2,3-di-*O*-benzyl 4,6-*O*-benzylidene-β-D-glucopyranoside (**12β**) as colourless gum. **12α**: R_f = 0.35 (EtOAc-hexane, 1:4, v/v); ^{1}H NMR (500 MHz, $CDCl_3$): δ 1.14-1.22 (m, 6H, cyclohex-H), 1.70-1.72 (m, 2H, cyclohex-H), 1.80-1.82 (m, 2H, cyclohex-H), 3.44-3.49 (m, 2H, H-2, cyclohex-H), 3.53 (t, *J* = 10.0 Hz, 1H, H-4), 4.62 (t, *J* = 10.0 Hz, 1H, H-6_A), 3.88 (td, *J* = 10.0, 5.0 Hz, 1H, H-5), 3.99 (t, *J* = 10.0 Hz, 1H, H-3), 4.18 (dd, *J* = 10.0, 5.0 Hz, 1H, H-6_B), 4.62 (d, 1H, *J* = 11.0 Hz, 0.5 x OCH_2Ph), 4.73 (d, 1H, *J* = 12.0 Hz, 0.5 x OCH_2Ph), 4.77 (d, 1H, *J* = 11.0 Hz, 0.5 x OCH_2Ph), 4.84-4.86 (m, 2H, H-1, 0.5 x OCH_2Ph), 5.48 (s, 1H, benzylidene-*H*), 7.19-7.43 (m, 15H, Ar-*H*); ^{13}C NMR (125 MHz, $CDCl_3$): δ 24.1, 24.3, 25.5, 29.6, 31.5, 33.4, 62.4, 69.1, 73.3, 75.3, 76.0, 78.6, 79.3, 82.4, 96.0, 101.1, 125.9, 127.5, 127.7, 127.94, 127.98, 128.2, 128.26, 128.3, 128.8, 137.4, 138.3, 138.9; **12β**: R_f = 0.37 (EtOAc-hexane, 1:4, v/v); ^{1}H NMR (500 MHz, $CDCl_3$): δ 1.17-1.19 (m, 4H, cyclohex-H), 1.21-1.22 (m, 2H, cyclohex-H), 1.68

(d, J = 5.0 Hz, 2H, cyclohex-H), 1.88 (d, J = 11.0 Hz, 2H, cyclohex-H), 3.32 (td, J = 10.0, 5.0 Hz, 1H, H-5), 3.38 (t, J = 8.0 Hz, 1H, H-2), 3.59-3.68 (m, 3H, H-3, H-6_A, cyclohex-H), 3.72 (t, J = 10.0 Hz, 1H, H-4), 4.26 (dd, J = 10.0, 5.0 Hz, 1H, H-6_B), 4.54 (d, J = 8.0 Hz, 1H, H-1), 4.68 (d, J = 11.0 Hz, 1H, 0.5 x OCH_2Ph), 4.71 (d, J = 11.0 Hz, 1H, 0.5 x OCH_2Ph), 4.82 (d, J = 11.0 Hz, 1H, 0.5 x OCH_2Ph), 4.86 (d, J = 11.0 Hz, 1H, 0.5 x OCH_2Ph), 5.48 (s, 1H, benzylidene-*H*), 7.18-7.42 (m, 15H, Ar-*H*); ^{13}C NMR (125 MHz, $CDCl_3$): δ 23.9, 24.0, 25.5, 31.9, 33.7, 66.0, 68.8, 75.0, 75.3, 76.7, 78.1, 81.0, 81.4, 82.1, 101.0, 102.3, 126.0, 127.5, 127.6, 127.9, 128.1, 128.2, 128.25, 128.3, 128.8, 137.3, 138.4, 138.6. Anal. Calcd for $C_{33}H_{38}O_6$: C, 74.69; H, 7.22; found: C, 74.85; H, 7.02.

11. Glycosylation of donor D5 with acceptor A2:

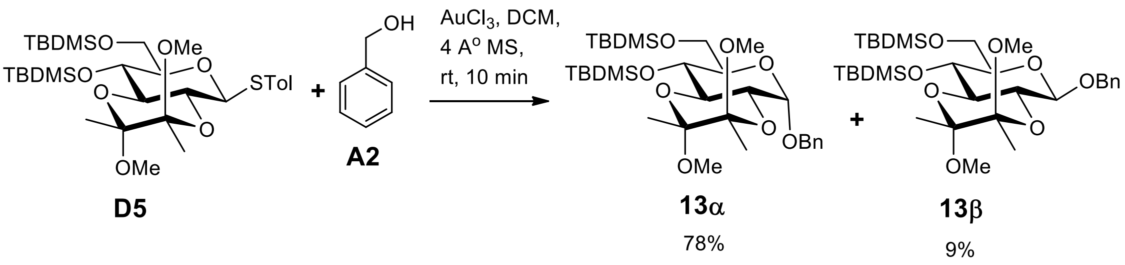

The treatment of donor **D5** (0.2 g, 0.31 mmol) with acceptor **A2** (0.05 mL, 0.47 mmol) in presence of 3 mol% of $AuCl_3$ gave 148 mg (78%) of benzyl glycoside **13α** and 17 mg (9%) of benzyl glycoside **13β** were obtained as colourless gum. **13α**: R_f = 0.45 (EtOAc-hexane, 1:9, v/v); ^{1}H NMR (500 MHz, $CDCl_3$): δ -0.004 (s, 3H, SiCH_3), -0.0002 (s, 3H, SiCH_3), 0.02 (s, 3H, SiCH_3), 0.11 (s, 3H, SiCH_3), 0.84 (s, 18H, ^{t}BuCH_3), 1.21 (s, 3H, CH_3), 1.22 (s, 3H, CH_3), 3.16 (s, 3H, OCH_3), 3.21 (s, 3H, OCH_3), 3.50-3.63 (m, 5H, H-2, H-4, H-5, H-6_A, H-6_B), 3.91 (t, J = 9.0 Hz, 1H, H-3), 4.61 (d, J = 12.0 Hz, 1H, 0.5 x OCH_2Ph), 4.69 (d, J = 12.0 Hz, 1H, 0.5 x OCH_2Ph), 4.77 (s, 1H, H-1), 7.28-7.36 (m, 5H, Ar-*H*), ^{13}C NMR (125 MHz, $CDCl_3$): δ -5.3, -5.1, -4.9, -3.5, 17.6, 17.68, 18.0, 18.4, 25.9, 25.94, 29.7, 47.8, 47.85, 62.1, 68.5, 68.7, 68.73, 69.7, 95.4, 99.3, 99.6, 127.4, 128.1, 137.6. Anal. Calcd for $C_{31}H_{56}O_8Si_2$: C, 60.75; H, 9.21; found: C, 61.05; H, 8.95.

12. Glycosylation of donor D3 with acceptor A1:

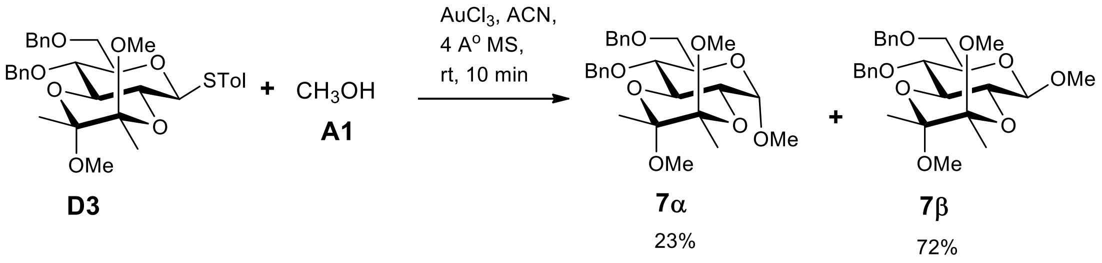

The treatment of donor **D3** (0.1 g, 0.17 mmol) with acceptor **A1** (0.069 mL, 1.7 mmol) in presence of 3 mol% of $AuCl_3$ gave 19 mg (23%) of methyl glycoside **7α** as colourless gum and 61 mg (72%) of methyl glycoside **7β** as white solid.

13. Glycosylation of donor D3 with acceptor A5:

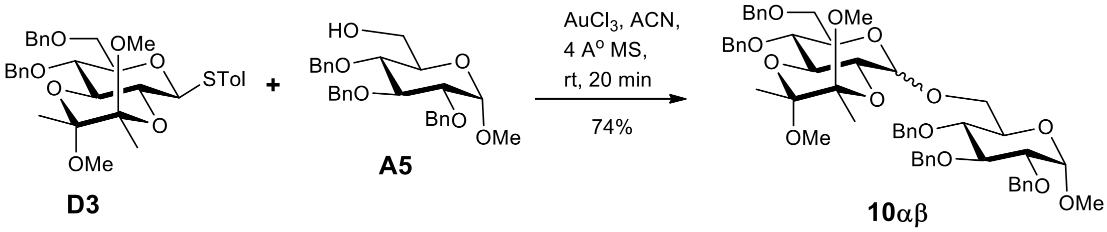

The treatment of donor **D3** (0.2 g, 0.34 mmol) with acceptor **A5** (0.17 g, 0.37 mmol) in presence of 3 mol% of $AuCl_3$ gave 234 mg (74%, *α/β* = 1:2.5) of disaccharide **10αβ** as colourless gum. ^{1}H NMR (500 MHz, $CDCl_3$): δ 1.13-1.26 (m, 12.3H), 3.11-3.12 (m, 2.5H), 3.19-3.20 (m, 5.9H), 3.24-3.32 (m, 3.9H), 3.42-3.46 (m, 2.6H), 3.48-3.60 (m, 5H), 3.64-3.70 (m, 4.8H), 3.76-3.80 (m, 1.4H), 3.89 (t, *J* = 9.0 Hz, 1.3H), 4.03 (d, *J* = 9.0 Hz, 1H), 4.15 (t, *J* = 9.7 Hz, 0.4H), 4.34-4.40 (m, 1.9H), 4.44-4.48 (m, 2.8H), 4.50-4.61 (m, 3.8H), 4.66-4.71 (m, 2.4H), 4.74-4.90 (m, 5.3H), 4.92 (d, *J* = 3.0 Hz, 0.3H), 7.14-7.23 (m, 35H, Ar-*H*);

14. Glycosylation of donor D6[9] with acceptor A5:

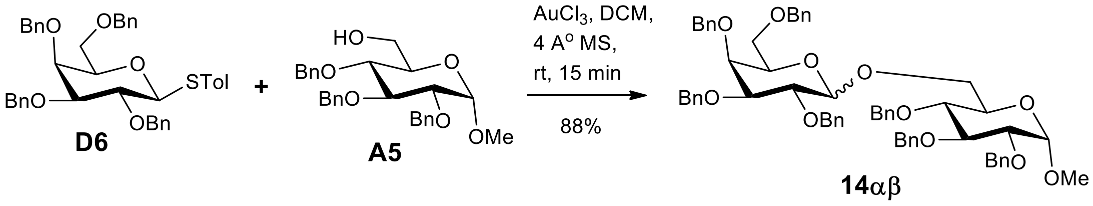

The treatment of donor **D6** (0.1 g, 0.15 mmol) with acceptor **A5** (0.085 g, 0.18 mmol) in presence of 5 mol% of $AuCl_3$ gave 134 mg (88%, *α/β* = 2.0:1) of disaccharide **14αβ** as colourless gum. R_f = 0.38 (EtOAc-hexane, 1:4, v/v); ^{1}H NMR (500 MHz, DMSO-d_6): δ 3.24-3.38 (m, 8.5H), 3.59-3.68 (m, 7.2H), 3.73-3.77 (m, 3H), 3.83-3.85 (m, 2H), 3.88-3.89 (m, 1.2H), 3.98-4.00 (m, 2.5H), 4.36-4.42 (m, 2H), 4.43-4.46 (m, 3.4H), 4.49 (s, 3H), 4.50 (s, 3H), 4.54-4.56 (m, 4H), 4.63-4.73 (m, 10H), 4.76-4.84 (m, 7H), 5.01 (d, *J* = 3.0 Hz, 1H), 5.22 (t, *J* = 6.0 Hz, 3.5H), 7.21-7.37 (m, 70H, Ar-*H*); ^{13}C NMR (125 MHz, DMSO-d_6): δ 54.8, 54.9, 63.3, 69.1, 69.4, 70.1, 71.6, 71.9, 72.7, 72.8, 73.0, 74.4, 74.5, 74.8, 75.3, 76.5, 77.6, 77.8, 81.6, 97.3, 126.8, 127.1, 127.6, 127.7, 127.8, 127.88, 127.9, 127.98, 128.0, 128.1, 128.18, 128.4, 128.5, 128.6, 128.68, 128.7, 138.6, 138.8, 139.0, 139.2, 139.3, 142.8. Anal. Calcd for $C_{62}H_{66}O_{11}$: C, 75.43; H, 6.74; found: C, 75.61; H, 6.93.

15. Glycosylation of donor D6 with acceptor A2:

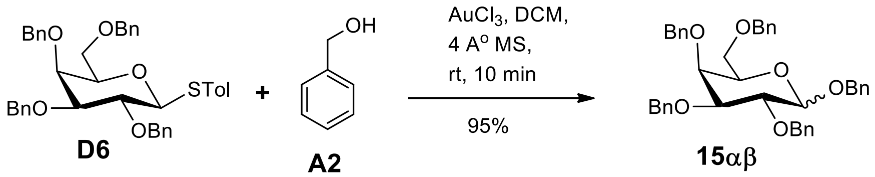

The treatment of donor **D6** (0.1 g, 0.15 mmol) with acceptor **A2** (0.08 mL, 0.77 mmol) in presence of 5 mol% of $AuCl_3$ gave 92 mg (95%, *α/β* = 1.7:1) of 1,2,3,4,6-penta-*O*-benzyl-D-galactopyranosides (**15αβ**)[10] as colourless gum. R_f = 0.41 (EtOAc-hexane, 1:4, v/v); ^{1}H NMR (500 MHz, DMSO-d_6): δ 3.52-3.61 (m, 4.9H), 3.67-3.70 (m, 0.8H), 3.73-3.78 (m, 0.8H), 3.85 (dd, *J* = 10.0, 3.0 Hz, 1.1H), 3.92 (dd, *J* = 10.0, 2.0 Hz, 1.1H), 3.95-3.98 (m, 1.5H), 4.07 (s, 1H), 4.44-4.51 (m, 5.9H), 4.58-4.61 (m, 3.2H), 4.63-4.80 (m, 5.3H), 5.01 (d, *J* = 3.0 Hz, 1H), 7.23-7.39 (m, 40H, Ar-*H*); ^{13}C NMR (125 MHz, DMSO-d_6): δ 68.9, 69.3, 69.6, 70.4, 72.0, 72.8, 73.0, 74.5, 75.4, 76.1, 78.4, 79.3, 81.7, 96.4, 102.4, 127.7, 127.75, 127.8, 127.88, 127.9, 127.95, 128.0, 128.1, 128.2, 128.4, 128.6, 128.66, 128.7, 130.1, 130.3, 131.4, 136.4, 138.2, 138.6, 139.2, 139.24. Anal. Calcd for $C_{41}H_{42}O_6$: C, 78.07; H, 6.71; found: C, 78.21; H, 6.89.

16. Glycosylation of donor D7[11] with acceptor A3:

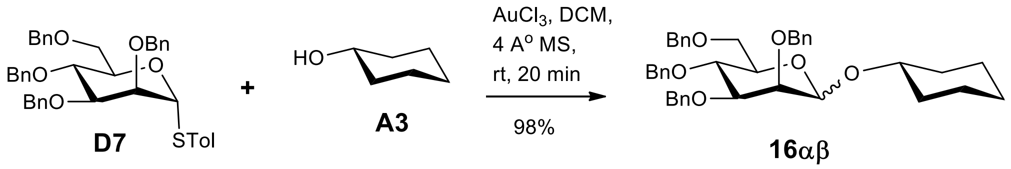

The treatment of donor **D7** (0.2 g, 0.31 mmol) with acceptor **A3** (0.05 mL, 0.46 mmol) in presence of 5 mol% of $AuCl_3$ gave 189 mg (98%, *α/β* = 2:1) of cyclohexyl 2,3,4,6-tetra-*O*-benzyl-D-mannopyranoside (**16αβ**)[12] as colourless gum. R_f = 0.40 (EtOAc-hexane, 1:4, v/v); ^{1}H NMR (500 MHz, $CDCl_3$): δ 1.12-1.28 (m, 7.1H), 1.43-1.44 (m, 2H), 1.68-1.73 (m, 6H), 3.37 (t, *J* = 7.5 Hz, 0.9H), 3.43 (dd, *J* = 9.0, 2.0 Hz, 0.9H), 3.48-3.50 (m, 0.6H), 3.64-3.66 (m, 2.8H), 3.70-3.78 (m, 3.8H), 3.85-3.92 (m, 1H), 4.34-4.36 (m, 1H), 4.41-4.48 (m, 3.6H), 4.50-4.60 (m, 3.1H), 4.61-4.70 (m, 1.4H), 4.79-4.85 (m, 2.2H), 4.92-4.93 (m, 1.3H), 4.34-4.36 (m, 1H), 7.09-7.42 (m, 30H, Ar-*H*); ^{13}C NMR (125 MHz, $CDCl_3$): δ 23.7, 23.8, 25.6, 25.7, 31.5, 33.5, 69.9, 71.3, 71.7, 72.1, 72.5, 73.2, 73.4, 73.7, 74.0, 75.0, 75.1, 75.19, 75.2, 75.9, 80.0, 82.6, 97.0, 99.5, 127.3, 127.39, 127.4, 127.5, 127.59, 127.6, 127.66, 127.69, 127.7, 127.8, 128.0, 128.07, 128.2, 128.28, 128.3, 128.4, 138.2, 138.4, 138.6, 138.9.

17. Glycosylation of donor D8[13] with acceptor A5:

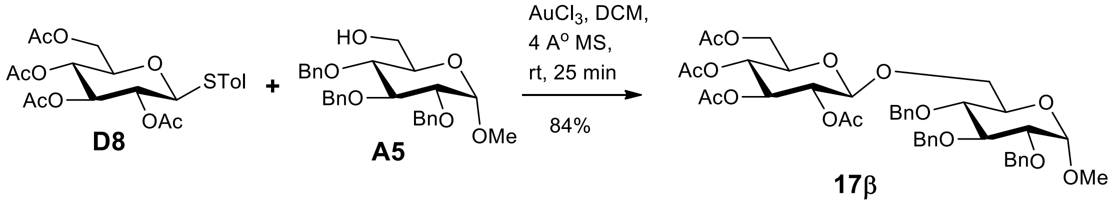

The treatment of donor **D8** (0.1 g, 0.22 mmol) with acceptor **A5** (0.11 g, 0.24 mmol) in presence of 5 mol% of $AuCl_3$ gave 143 mg (84%) of disaccharide **17β**[14] as colourless gum. The spectral data and elemental analysis is identical to that of reported data. ^{1}H NMR (500 MHz, $CDCl_3$): δ 1.85 (s, 3H, COCH_3), 1.89 (s, 3H, COCH_3), 1.91 (s, 3H, COCH_3), 1.94 (s, 3H, COCH_3), 3.25 (s, 3H, OCH_3), 3.32 (t, *J* = 9.0 Hz, 1H), 3.43 (d, *J* = 8.0 Hz, 1H), 3.53-3.59 (m, 2H), 3.65-3.67 (m, 1H), 3.86 (t, *J* = 9.0 Hz, 1H), 3.95 (d, *J* = 11.0 Hz, 1H), 4.01 (d, *J*

= 12.0 Hz, 1H), 4.12 (dd, *J* = 12.0, 4.0 Hz, 1H), 4.41-4.43 (m, 3H), 4.54 (d, *J* = 12.0 Hz, 1H), 4.67-4.70 (m, 2H), 4.75 (d, *J* = 11.0 Hz, 1H), 4.87 (d, *J* = 11.0 Hz, 1H), 4.92-4.99 (m, 2H), 5.07 (t, *J* = 10.0 Hz, 1H), 7.14-7.26 (m, 15H, Ar-*H*).

18. Glycosylation of donor D9[15] with acceptor A2:

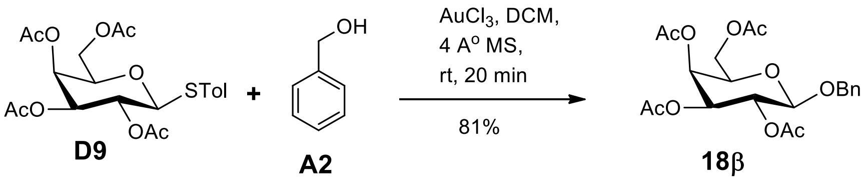

The treatment of donor **D9** (0.1 g, 0.22 mmol) with acceptor **A2** (0.11 mL, 1.1 mmol) in presence of 5 mol% of $AuCl_3$ gave 78 mg (81%) of benzyl 2,3,4,6-tetra-*O*-acetyl-β-D-galactopyranoside (**18β**)[16] as colourless gum. The spectral data and elemental analysis is identical to that of reported data. ^{1}H NMR (500 MHz, $CDCl_3$): δ 1.90 (s, 3H, COC*H*$_3$), 1.94 (s, 3H, COC*H*$_3$), 1.99 (s, 3H, COC*H*$_3$), 2.09 (s, 3H, COC*H*$_3$), 3.81 (t, *J* = 6.5 Hz, 1H, H-5), 4.08 (dd, *J* = 11.0, 7.0 Hz, 1H, H-6$_A$), 4.14 (dd, *J* = 11.0, 7.0 Hz, 1H, H-6$_B$), 4.44 (d, *J* = 8.0 Hz, 1H, H-1), 4.56 (d, *J* = 12.0 Hz, 1H, 0.5 x OC*H*$_2$Ph), 4.84 *J* = 12.0 Hz, 1H, 0.5 x OC*H*$_2$Ph), 4.91 (dd, *J* = 10.0, 3.0 Hz, 1H, H-3), 5.20 (dd, *J* = 10.0, 8.0 Hz, 1H, H-2), 5.32 (d, *J* = 2.0 Hz, 1H, H-4), 7.19-7.28 (m, 5H, Ar-*H*).

19. Glycosylation of donor D9 with acceptor A5:

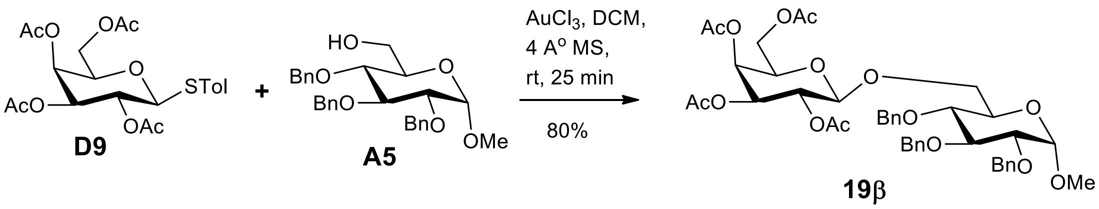

The treatment of donor **D9** (0.1 g, 0.22 mmol) with acceptor **A5** (0.11 g, 0.24 mmol) in presence of 5 mol% of $AuCl_3$ gave 139 mg (80%) of disaccharide **19β**[17] as colourless gum. ^{1}H NMR (500 MHz, $CDCl_3$): δ 1.86 (s, 3H, COC*H*$_3$), 1.87 (s, 3H, COC*H*$_3$), 1.91 (s, 3H, COC*H*$_3$), 2.02 (s, 3H, COC*H*$_3$), 3.26 (s, 3H, OC*H*$_3$), 3.31 (t, *J* = 9.0 Hz, 1H), 3.41 (dd, *J* = 10.0, 3.0 Hz, 1H), 3.56 (dd, *J* = 11.0, 5.0 Hz, 1H), 3.67 (dd, *J* = 10.0, 3.0 Hz, 1H), 3.75 (t, *J* =

7.0 Hz, 1H), 3.87 (t, J = 9.0 Hz, 1H), 3.97-4.05 (m, 3H), 4.37 (d, J = 8.0 Hz, 1H), 4.43 (d, J = 11.0 Hz, 1H), 4.48 (d, J = 3.0 Hz, 1H), 4.54 (d, J = 12.0 Hz, 1H), 4.67-4.70 (m, 2H), 4.76 (d, J = 11.0 Hz, 1H), 4.87-4.89 (m, 2H), 5.14-5.17 (m, 1H), 5.26 (d, J = 2.0 Hz, 1H), 7.14-7.25 (m, 15H, Ar-*H*)

20. Glycosylation of donor D10[18] with acceptor A5:

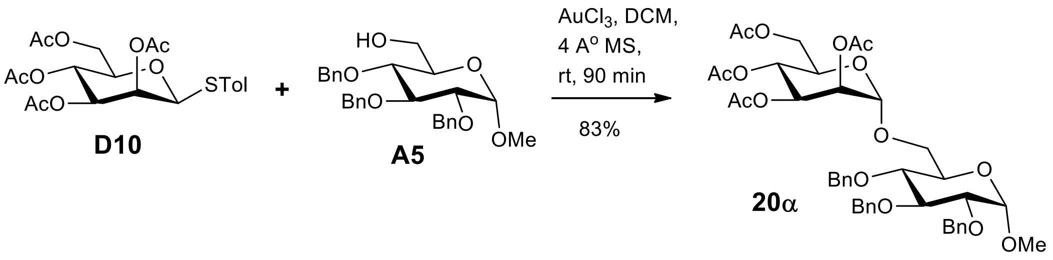

The treatment of donor **D10** (0.2 g, 0.44 mmol) with acceptor **A5** (0.22 g, 0.48 mmol) in presence of 5 mol% of $AuCl_3$ gave 290 mg (83%) of disaccharide **20α**[19] as colourless gum. The spectral data and elemental analysis is identical to that of the reported data. ^{1}H NMR (500 MHz, $CDCl_3$): δ 1.88 (s, 3H, COCH_3), 1.91 (s, 3H, COCH_3), 1.93 (s, 3H, COCH_3), 2.04 (s, 3H, COCH_3), 3.28 (s, 3H, OCH_3), 3.36 (t, J = 9.0 Hz, 1H), 3.45 (dd, J = 9.0, 3.0 Hz, 1H), 3.57 (d, J = 10.0 Hz, 1H), 3.67-3.69 (m, 2H), 3.84-3.86 (m, 1H), 3.88-3.94 (m, 2H), 4.05 (dd, J = 12.0, 5.0 Hz, 1H), 4.50 (d, J = 11.0 Hz, 2H), 4.58 (d, J = 12.0 Hz, 1H), 4.68-4.71 (m, 2H), 4.77 (s, 1H), 4.85-4.90 (m, 2H), 5.13-5.21 (m, 3H), 7.16-7.28 (m, 15H, Ar-*H*)

21. Glycosylation of donor D3 with acceptor A7:

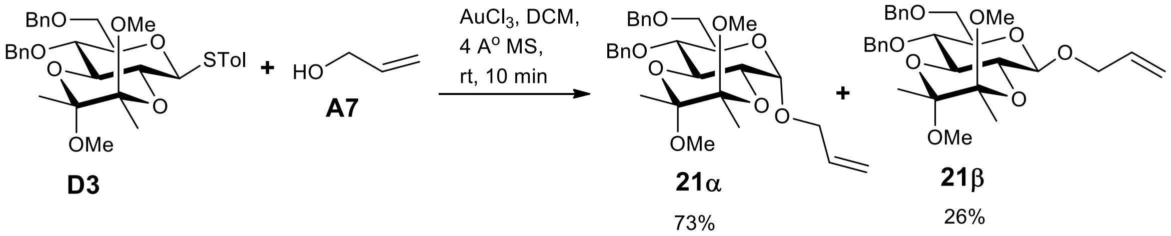

The treatment of donor **D3** (0.1 g, 0.17 mmol) with acceptor **A7** (0.017 mL, 0.25 mmol) in presence of 3 mol% of $AuCl_3$ gave 64 mg (73%) of allyl glycoside **21α** and 23 mg (26%) of allyl glycoside **21β** as colourless gum. **21α**: R_f = 0.36 (EtOAc-hexane, 1:4, v/v); ^{1}H NMR

(500 MHz, $CDCl_3$): δ 1.26 (s, 3H, CH_3), 1.27 (s, 3H, CH_3), 3.19 (s, 3H, OCH_3), 3.22 (s, 3H, OCH_3), 3.56 (d, *J* = 10.0 Hz, 1H, H-6_A), 3.66-3.76 (m, 4H, H-2, H-4, H-5, H-6_B), 4.01-4.11 (m, 2H, CH_2CH=CH_2), 4.15 (t, *J* = 9.0 Hz, 1H, H-3), 4.38-4.42 (m, 2H, OCH_2Ph), 4.56 (d, 1H, *J* = 12.0 Hz, 0.5 x OCH_2Ph), 4.85-5.08 (m, 2H, OCH_2Ph), 5.10 (d, 1H, *J* = 10.0 Hz, 0.5 x CH_2CH=CH_2), 5.21 (d, 1H, *J* = 17.0 Hz, 0.5 x CH_2CH=CH_2), 5.82-5.90 (m, 1H, CH_2C*H*=CH_2), 7.11-7.25 (m, 10H, Ar-*H*); ^{13}C NMR (125 MHz, $CDCl_3$): δ 17.6, 18.0, 47.8, 47.9, 68.2, 68.3, 68.4, 70.6, 70.8, 73.4, 74.9, 75.0, 95.8, 99.3, 99.8, 117.9, 127.64, 127.66, 127.8, 127.9, 128.32, 128.34, 128.4, 128.7, 129.3, 134.0, 138.1, 138.5; **21β**: R_f = 0.38 (EtOAc-hexane, 1:4, v/v); ^{1}H NMR (500 MHz, $CDCl_3$): δ 1.25 (s, 3H, CH_3), 1.28 (s, 3H, CH_3), 3.21 (s, 3H, OCH_3), 3.23 (s, 3H, OCH_3), 3.42 (dd, *J* = 10.0, 5.0 Hz, 1H, H-5), 3.52-3.67 (m, 4H, H-2, H-4, H-6_A, H-6_B), 3.81 (t, *J* = 10.0 Hz, 1H, H-3), 4.07 (dd, *J* = 13.0, 5.0 Hz, 1H, 1 x CH_2CH=CH_2), 4.30 (dd, *J* = 13.0, 5.0 Hz, 1H, 1 x CH_2CH=CH_2), 4.43-4.47 (m, 3H, H-1, 1 x OCH_2Ph), 4.53 (d, 1H, *J* = 12.0 Hz, 0.5 x OCH_2Ph), 4.84 (d, 1H, *J* = 12.0 Hz, 0.5 x OCH_2Ph), 5.08 (d, 1H, *J* = 10.0 Hz, 0.5 x CH_2CH=CH_2), 5.25 (d, 1H, *J* = 17.0 Hz, 0.5 x CH_2CH=CH_2), 5.82-5.87 (m, 1H, CH_2C*H*=CH_2), 7.14-7.26 (m, 10H, Ar-*H*); ^{13}C NMR (125 MHz, $CDCl_3$): δ 17.6, 17.8, 47.8, 47.9, 69.0, 69.4, 69.9, 73.4, 73.8, 74.8, 75.5, 99.4, 99.6, 116.8, 127.5, 127.7, 128.0, 128.3, 134.1, 138.30, 138.33. Anal. Calcd for $C_{29}H_{38}O_8$: C, 67.69; H, 7.44; found: C, 67.80; H, 7.63.

22. Glycosylation of donor D11 with acceptor A5:

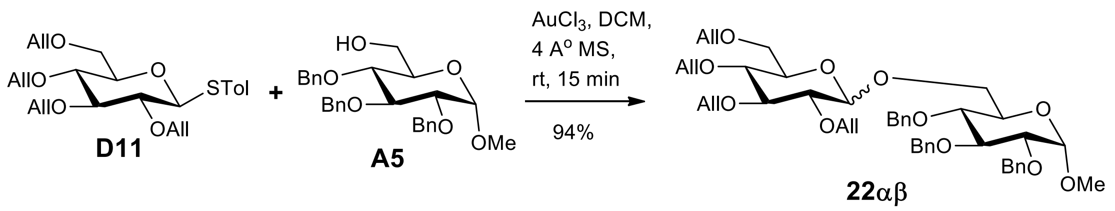

The treatment of donor **D11** (0.2 g, 0.44 mmol) with acceptor **A5** (0.22 g, 0.49 mmol) in presence of 5 mol% of $AuCl_3$ gave 330 mg (94%, *α/β* = 2.0:1) of disaccharide **22αβ** as colourless gum. R_f = 0.29 (EtOAc-hexane, 1:4, v/v); ^{1}H NMR (500 MHz, $CDCl_3$): δ 3.15 (t, *J*

= 7.5 Hz, 0.6H), 3.23-3.26 (m, 2.1H), 3.27-3.28 (m, 4.9H), 3.32-3.34 (m, 1.4H), 3.41-3.46 (m, 2.9H), 3.49-3.53 (m, 2.7H), 3.59-3.63 (m, 3.5H), 3.66-3.69 (m, 3.4H), 3.88-3.96 (m, 4.8H), 3.99-4.00 (m, 2.5H), 4.02-4.03 (m, 1.6H), 4.08-4.17 (m, 3H), 4.20-4.25 (m, 3.7H), 4.47 (d, *J* = 3.5 Hz, 1H), 4.53-4.59 (m, 3.7H), 4.68-4.75 (m, 3H), 4.80-4.83 (m, 1.4H), 4.88-4.90 (m, 2.4H), 5.01-5.09 (m, 6.2H), 5.13-5.19 (m, 6.3H), 5.78-5.85 (m, 6.1H), 7.18-7.28 (m, 23H, Ar-*H*). Anal. Calcd for $C_{46}H_{58}O_{11}$: C, 70.21; H, 7.43; found: C, 70.45; H, 7.61.

23. Glycosylation of donor D8 with acceptor A5 using $AuBr_3$:

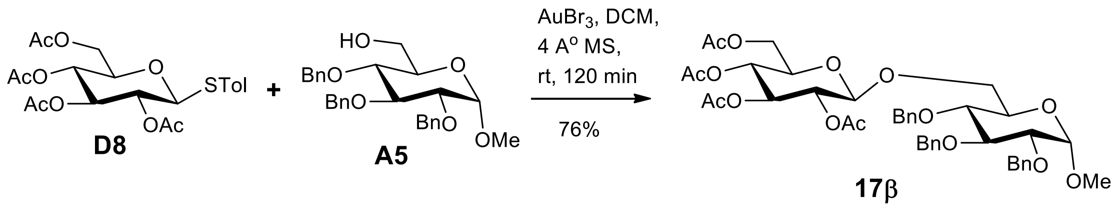

The treatment of donor **D8** (0.1 g, 0.22 mmol) with acceptor **A5** (0.11 g, 0.24 mmol) in presence of 20 mol% of $AuBr_3$ gave 130 mg (76%) of disaccharide **17β** as a colourless gum.

24. Glycosylation of donor D9 with acceptor A5:

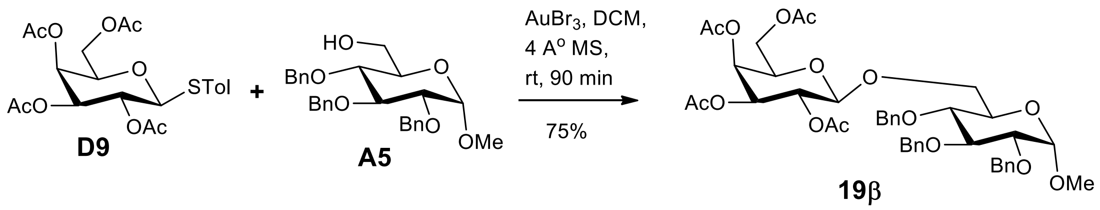

The treatment of donor **D9** (0.1 g, 0.22 mmol) with acceptor **A5** (0.11 g, 0.24 mmol) in presence of 20 mol% of $AuBr_3$ gave 131 mg (75%) of disaccharide **19β** as colourless gum.

25. Glycosylation of donor D1 with acceptor A8[20]:

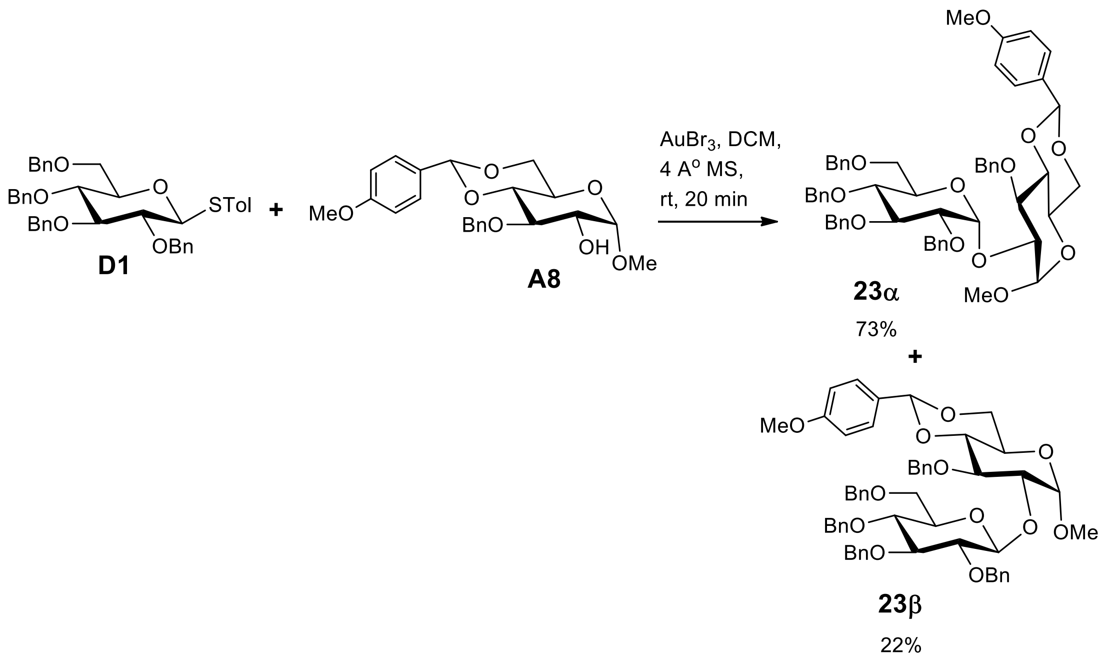

The treatment of donor **D1** (0.05 g, 0.077 mmol) with acceptor **A8** (0.04 g, 0.10 mmol) in presence of 3 mol% of $AuBr_3$ gave 52 mg (73%) of disaccharide **23α** and 16 mg (22%) of disaccharide **23β** as colourless gum. **23α**: R_f = 0.31 (EtOAc-hexane, 1:3, v/v); ^{1}H NMR (500 MHz, $CDCl_3$): δ 3.30 (dd, *J* = 11.0, 2.0 Hz, 1H), 3.38 (s, 3H, OCH_3), 3.40 (d, *J* = 3.0, Hz, 1H), 3.49-3.53 (m, 2H), 3.57-3.66 (m, 2H), 3.74 (s, 3H, OCH_3), 3.75-3.79 (m, 2H), 3.99-4.06 (m, 3H), 4.19-4.22 (m, 2H), 4.37 (d, *J* = 11.0, Hz, 1H), 4.44 (d, *J* = 12.0, Hz, 1H), 4.61-4.67 (m, 2H), 4.71-4.74 (m, 2H), 4.76-4.80 (m, 3H), 4.84 (d, *J* = 3.0 Hz, 1H), 4.93 (d, *J* = 11.0 Hz, 1H), 5.44 (s, 1H, benzylidene-*H*), 6.93-7.28 (m, 29H, Ar-*H*); ^{13}C NMR (125 MHz, $CDCl_3$): δ 55.0, 55.3, 62.3, 68.0, 68.9, 69.9, 73.0, 73.2, 74.32, 74.9, 75.6, 75.7, 77.7, 79.1, 82.1, 82.3, 94.4, 97.2, 101.2, 113.6, 127.2, 127.4, 127.5, 127.6, 127.7, 127.8, 127.9, 127.98, 128.0, 128.2, 128.28., 128.3, 128.4, 128.7, 137.9, 138.0, 138.4, 138.6; **23β**: R_f = 0.33 (EtOAc-hexane, 1:3, v/v); ^{1}H NMR (500 MHz, $CDCl_3$): δ 3.32-3.35 (m, 1H), 3.36 (s, 3H, OCH_3), 3.43-3.58 (m, 6H), 3.66 (t, *J* = 10.0 Hz, 1H), 3.73 (s, 3H, OCH_3), 3.77 (dd, *J* = 10.0, 4.0 Hz, 1H), 3.80-3.82 (m, 1H), 4.01 (t, *J* = 9.0 Hz, 1H), 4.22 (dd, *J* = 10.0, 5.0 Hz, 1H), 4.41-4.51 (m, 4H), 4.57 (d, *J* = 11.0, Hz, 1H), 4.67-4.73 (m, 5H), 4.83-4.88 (m, 2H), 4.97 (d, *J* = 11.0, Hz, 1H), 5.44 (s, 1H), 6.81 (d, *J* = 8.0 Hz, 2H, Ar-*H*), 7.07-7.26 (m, 25H, Ar-*H*), 7.31 (d, *J* =

8.0 Hz, 2H, Ar-*H*),; ^{13}C NMR (125 MHz, $CDCl_3$): δ 55.3, 55.38, 62.2, 69.0, 69.1, 73.4, 74.5, 74.6, 74.9, 75.6, 77.7, 78.1, 78.5, 81.9, 82.6, 84.7, 100.4, 101.3, 104.3, 113.5, 127.3, 127.36, 127.4, 127.5, 127.6, 127.7, 127.8, 127.9, 128.0, 128.05, 128.09, 128.1, 128.18, 128.3, 128.39, 128.4, 129.9, 138.0, 138.1, 138.15. Anal. Calcd for $C_{56}H_{60}O_{12}$: C, 72.71; H, 6.54; found: C, 72.88; H, 6.70.

26. Glycosylation of donor D1 with acceptor A9[21]:

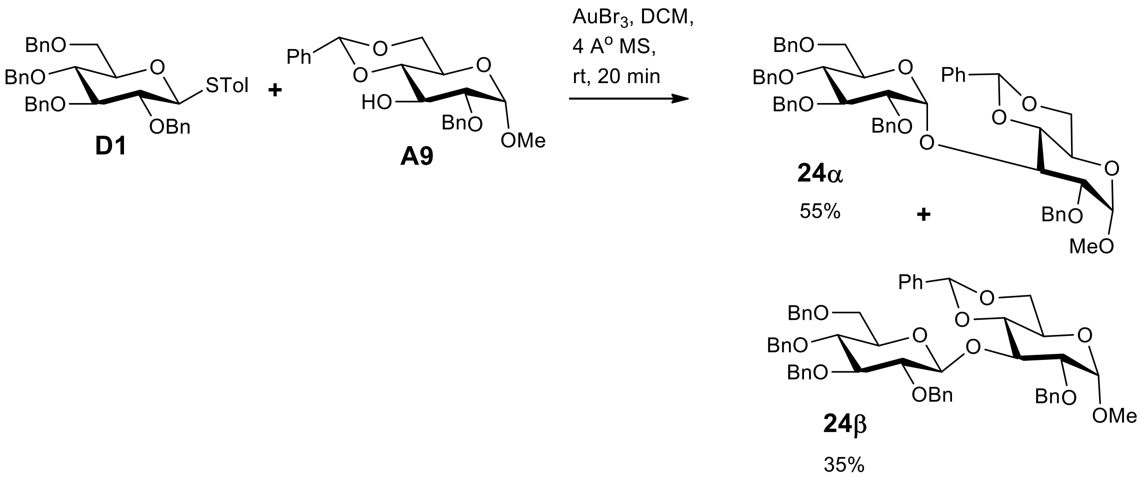

The treatment of donor **D1** (0.05 g, 0.077 mmol) with acceptor **A9** (0.04 g, 0.10 mmol) in presence of 3 mol% of $AuBr_3$ gave 38 mg (55%) of disaccharide **24α** and 24 mg (35%) of disaccharide **24β** as colourless gum. **24α**: R_f = 0.32 (EtOAc-hexane, 1:3, v/v); ^{1}H NMR (500 MHz, $CDCl_3$): δ 3.33 (s, 3H, OCH_3), 3.39-3.44 (m, 3H), 3.52-3.65 (m, 3H), 3.70 (t, *J* = 9.0, Hz, 1H), 3.77-3.82 (m, 1H), 3.89 (t, *J* = 9.0, Hz, 1H), 4.11-4.17 (m, 2H), 4.20-4.36 (m, 4H), 4.46-4.51 (m, 3H), 4.58 (d, *J* = 11.0 Hz, 1H, 0.5 x OCH_2Ph), 4.63 (s, 1H), 4.72 (d, *J* = 11.0 Hz, 2H, 1 x OCH_2Ph), 4.91 (d, *J* = 11.0 Hz, 1H, 0.5 x OCH_2Ph), 5.38 (s, 1H), 5.51 (s, 1H), 6.85 (d, *J* = 7.0 Hz, 2H, Ar-*H*), 7.01-7.30 (m, 28H, Ar-*H*); ^{13}C NMR (125 MHz, $CDCl_3$): δ 55.3, 61.8, 68.1, 69.2, 69.8, 71.1, 72.7, 73.3, 73.4, 74.7, 75.5, 77.5, 78.0, 78.8, 81.6, 82.9, 96.1, 98.5, 102.1, 126.4, 127.3, 127.36, 127.4, 127.5, 127.6, 127.8, 127.9, 128.1, 128.2, 128.28, 128.4, 128.46, 128.7, 129.3, 137.0, 137.4, 137.8, 138.0, 138.9; **24β**: R_f = 0.34 (EtOAc-hexane, 1:3, v/v); ^{1}H NMR (500 MHz, $CDCl_3$): δ 3.16-3.18 (m, 1H), 3.27 (s, 3H,

OCH_3), 3.42 (t, J = 8.0, Hz, 1H), 3.47-3.49 (m, 2H), 3.50-3.52 (m, 2H), 3.55-3.61 (m, 3H), 3.62-3.67 (m, 1H), 3.72-3.79 (m, 1H), 4.14 (dd, J = 10.0, 5.0 Hz, 1H), 4.28 (t, J = 9.0 Hz, 1H), 4.38-4.41 (m, 3H), 4.44-4.46 (m, 1H), 4.63-4.67 (m, 2H), 4.69-4.72 (m, 2H), 4.81-4.85 (m, 2H, 1 x OCH_2Ph), 4.98 (d, J = 11.0 Hz, 1H, 0.5 x OCH_2Ph), 5.39 (s, 1H), 7.14-7.27 (m, 30H, Ar-H); ^{13}C NMR (125 MHz, $CDCl_3$): δ 55.3, 62.1, 68.6, 69.0, 73.5, 73.7, 74.7, 74.8, 74.9, 75.5, 75.7, 77.9, 80.3, 80.4, 82.9, 84.9, 98.7, 101.5, 102.4, 126.1, 127.4, 127.5, 127.6, 127.8, 127.9, 128.0, 128.1, 128.2, 128.3, 12835., 128.8, 137.4, 138.2, 138.4, 138.9. Anal. Calcd for $C_{55}H_{58}O_{11}$: C, 73.81; H, 6.53; found: C, 74.03; H, 6.35.

27. Glycosylation of donor D1 with acceptor A10[22]:

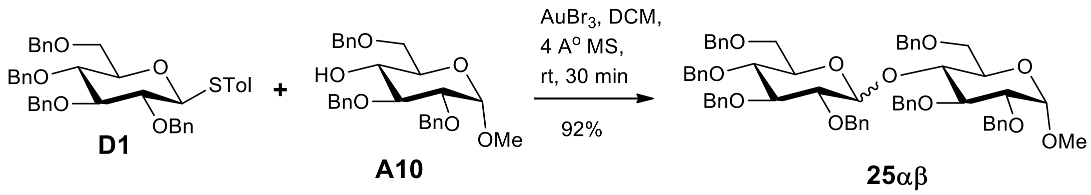

The treatment of donor **D1** (0.05 g, 0.077 mmol) with acceptor **A10** (0.04 g, 0.096 mmol) in presence of 3 mol% of $AuBr_3$ gave 70 mg (92%, α/β = 1.4:1) of disaccharide **25αβ**[23] as colourless gum. ^{1}H NMR (500 MHz, $CDCl_3$): δ 3.28-3.33 (m, 5.9H), 3.40-3.61 (m, 12.1H), 3.74-3.83 (m, 3.7H), 3.96-4.09 (m, 2.4H), 4.19-4.67 (m, 18.2H), 4.67-5.14 (m, 10.1H), 5.60 (s, 0.6H), 5.61 (s, 0.4H), 7.03-7.32 (m, 60H, Ar-H);

28. Glycosylation of donor D1 with acceptor A11[24]:

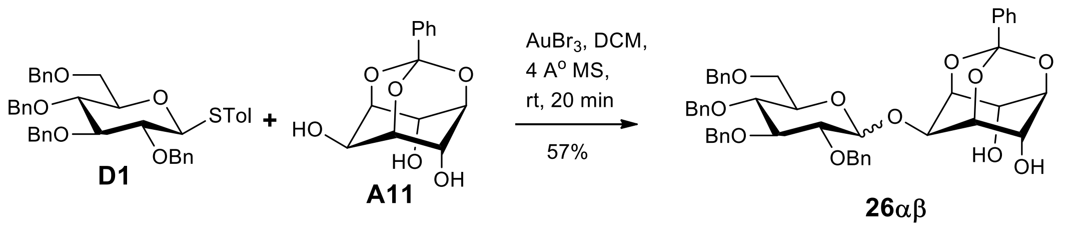

The treatment of donor **D1** (0.2 g, 0.31 mmol) with acceptor **A11** (0.099 g, 0.37 mmol) in presence of 3 mol% of $AuBr_3$ gave 136 mg (57%, α/β = 1.4:1) of pseudodisaccharide **26αβ** as colourless gum. R_f = 0.19 (EtOAc-hexane, 1:2, v/v); ^{1}H NMR (500 MHz, $CDCl_3$): δ 3.40-

3.47 (m, 3.1H), 3.49-3.56 (m, 3.4H), 3.58-3.71 (m, 3.1H), 3.73-3.92 (m, 1.6H), 4.04-4.06 (m, 2.5H), 4.26-4.35 (m, 2.3H), 4.40-4.48 (m, 6.7H), 4.55-4.62 (m, 5H), 4.65-4.76 (m, 5H), 4.79-4.92 (m, 1.5H), 5.01-5.03 (m, 1.6H), 7.09-7.23 (m, 42H, Ar-*H*), 7.56 (bs, 2H, Ar-*H*); ^{13}C NMR (125 MHz, $CDCl_3$): δ 67.2, 68.1, 68.4, 68.5, 69.5, 71.7, 71.76, 71.8, 72.4, 73.1, 73.4, 73.6, 73.7, 73.8, 74.0, 74.6, 74.68, 76.9, 76.8, 79.1, 83.5, 95.5, 101.2, 106.1, 106.13, 124.4, 124.44, 126.5, 126.6, 126.65, 126.7, 126.8, 126.9, 126.94, 126.96, 127.0, 127.2, 127.3, 127.36, 127.4, 127.5, 128.3, 136.0, 137.2. Anal. Calcd for $C_{47}H_{48}O_{11}$: C, 71.56; H, 6.13; found: C, 71.34; H, 6.30.

29. Glycosylation of donor D12[25] with acceptor A5:

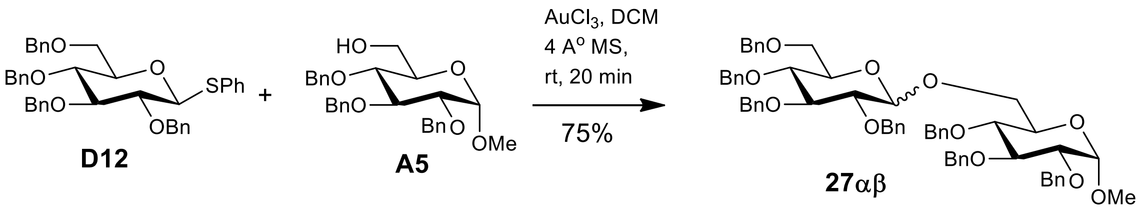

The treatment of donor **D12** (0.047 g, 0.074 mmol) with acceptor **A5** (0.038 g, 0.081 mmol) in presence of 5 mol% of $AuCl_3$ gave 55 mg (75%, *α/β* = 1.4:1) of pseudodisaccharide **27αβ**[26] as colourless gum. R_f = 0.4 (EtOAc-hexane, 1:4, v/v); ^{1}H NMR (500 MHz, $CDCl_3$): δ 3.25 (s, 2H), 3.27 (s, 3H), 3.32-3.39 (m, 2H), 3.41-3.49 (m, 5H), 3.51-3.59 (m, 6H), 3.61-3.65 (m, 2H), 3.68-3.78 (m, 4H), 3.88-3.91 (m, 3H), 4.09-4.12 (m, 1H), 4.26-4.28 (m, 1H), 4.34-4.42 (m, 4H), 4.46-4.50 (m, 5H), 4.52-4.62 (m, 5H), 4.64-4.70 (m, 5H), 4.71-4.75 (m, 5H), 4.82-4.89 (m, 6H), 7.05-7.25 (m, 60H, Ar-*H*).

D) <u>Structural assignment of pseudo-disaccharide 26:</u>

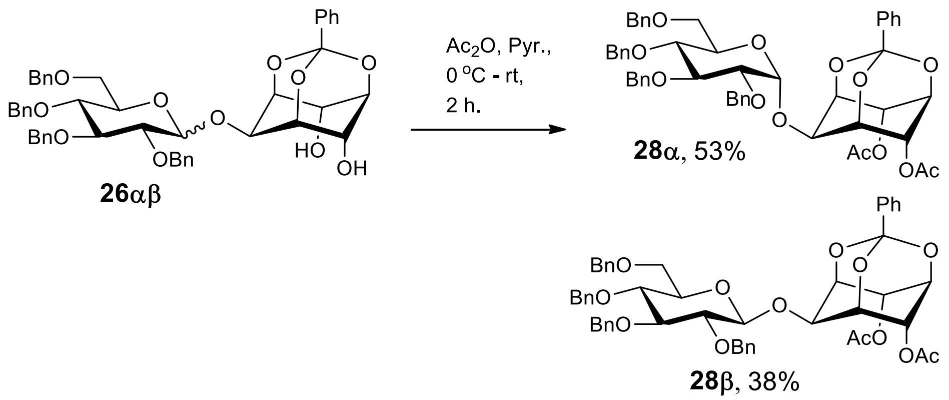

A solution of pseudo-disaccharide **28αβ** (0.02 g, 0.025 mmol) in anhydrous pyridine (2 mL) was cooled to 0 °C and to this solution acetic anhydride (0.014 mL, 0.126 mmol) was added drop-wise. Then catalytic amount of DMAP (1 mg) was added and the reaction mixture was stirred at room temperature for 2 h. When TLC showed complete conversion of the starting material, the reaction mixture was cooled to 0 °C and quenched by adding ice. The reaction mixture was extracted with ethyl acetate (2 x 10 mL) and washed with water (2 x 10 mL) followed by brine. The combined organic layer was dried over anhydrous sodium sulphate, filtered and concentrated under reduced pressure. The residue thus obtained was purified by flash column chromatography to afford **28α** (11.6 mg, 53%) and **28β** (8.3 mg, 38%) as colourless gum. **28α**: R_f = 0.33 (EtOAc-hexane, 1:2, v/v); ^{1}H NMR (500 MHz, $CDCl_3$): δ 1.96 (s, 3H, $COCH_3$), 2.00 (s, 3H, $COCH_3$), 3.51-3.61 (m, 4H, H-2', H-4', H-5', H-6$_A$'), 3.88 (bs, 1H, H-5), 3.99 (d, J = 9.0, Hz, 1H, H-6$_B$'), 4.04 (t, J = 9.0, Hz, 1H, H-3'), 4.35 (d, J = 11.0, Hz, 1H, 0.5 x OCH_2Ph), 4.39 (d, J = 11.0, Hz, 1H, 0.5 x OCH_2Ph), 4.48-4.50 (m, 2H, 0.5 x OCH_2Ph), 4.55-4.57 (m, 2H, H-1, H-3), 4.61 (bs, 1H, H-2), 4.73-4.77 (m, 3H, 1.5 x OCH_2Ph), 4.89 (d, J = 11.0, Hz, 1H, 0.5 x OCH_2Ph), 4.94 (d, J = 3.0, Hz, 1H, H-1'), 5.48 (bs, 1H, H-4/6), 5.55 (bs, 1H, H-4/6), 7.06 (d, J = 7.0, Hz, 1H, Ar-H), 7.26-7.30 (m, 21H, Ar-H), 7.58 (d, J = 8.0, Hz, 1H, Ar-H); ^{13}C NMR (125 MHz, $CDCl_3$): δ 20.6 ($COCH_3$), 20.7 ($COCH_3$), 66.7, 66.9, 68.0, 68.6, 70.2, 70.8, 71.4, 73.1, 73.4, 74.8, 75.6, 77.6, 80.3, 81.6, 97.2, 107.9, 125.4, 127.6, 127.7, 127.8, 127.85, 127.9, 128.0, 128.3, 128.36, 128.4, 129.6,

137.8, 138.3, 138.8, 169.0 (*C*O), 169.1 (*C*O); **28β**: R_f = 0.35 (EtOAc-hexane, 1:2, v/v); ^{1}H NMR (500 MHz, $CDCl_3$): δ 1.90 (s, 3H, COC*H*$_3$), 1.95 (s, 3H, COC*H*$_3$), 3.46-3.48 (m, 2H, H-5', H-6$_A$'), 3.51 (t, *J* = 8.0, Hz, 1H, H-2'), 3.55-3.63 (m, 3H, H-3', H-4', H-6$_B$'), 4.13 (bs, 1H, H-5), 4.44-4.53 (m, 4H, 2 x OC*H*$_2$Ph), 4.59-4.67 (m, 4H, H-1, H-2, H-3, H-1'), 4.71-4.77 (m, 2H, 1 x OC*H*$_2$Ph), 4.89 (d, *J* = 11.0, Hz, 1H, 0.5 x OC*H*$_2$Ph), 4.97 (d, *J* = 11.0, Hz, 1H, 0.5 x OC*H*$_2$Ph), 5.55 (bs, 2H, H-4, H-6), 7.10-7.22 (m, 23H, Ar-*H*), 7.58 (d, *J* = 8.0, Hz, 1H, Ar-*H*); ^{13}C NMR (125 MHz, $CDCl_3$): δ 20.6 (CO*C*H$_3$), 20.68 (CO*C*H$_3$), 66.3, 67.0, 68.0, 68.08, 69.1, 69.9, 72.0, 73.4, 74.8, 75.0, 75.07, 75.7, 77.7, 81.7, 84.5, 102.4, 108.0, 125.4, 127.6, 127.7, 127.8, 127.9, 128.3, 129.6, 138.0, 138.09, 138.1, 169.1 (*C*O), 169.2 (*C*O); Anal. Calcd for $C_{51}H_{52}O_{13}$: C, 70.17; H, 6.00; found: C, 70.33; H, 6.28.

E) <u>Hydrolysis of thioglycosides:</u>

Table S1. Hydrolysis of thioglycosides by catalytic amount of gold (III) chloride trihydrate.

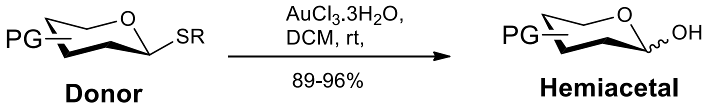

Entry	Donor	Hemiacetal	Time (min)	Yield (%)[a]	α/β Ratio [b]
1	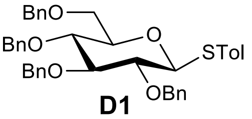 **D1**	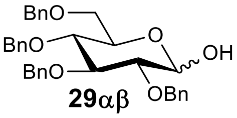 **29αβ**	60	93	1:1
2	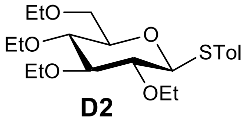 **D2**	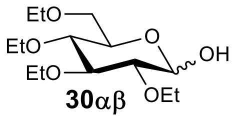 **30αβ**	30	90	1:1
3	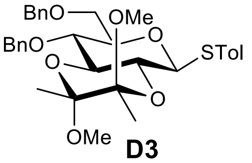 **D3**	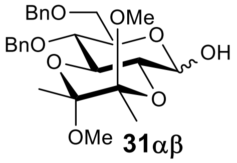 **31αβ**	30	92	1:0.6
4	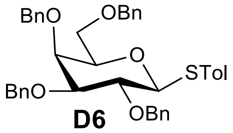 **D6**	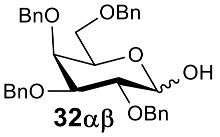 **32αβ**	30	92	1:0.6
5	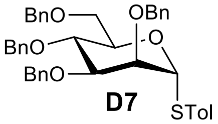 **D7**	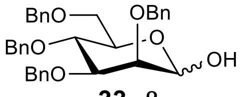 **33αβ**	90	92	1:0.14

6	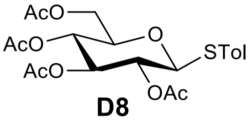 **D8**	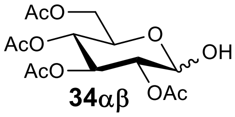 **34αβ**	120	95	1:0.4
7	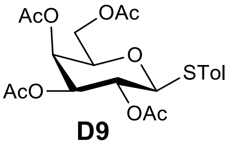 **D9**	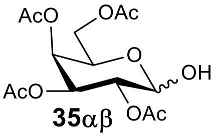 **35αβ**	105	90	1:0.3
8	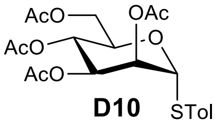 **D10**	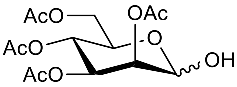 **36αβ**	120	89	1:0.1
9	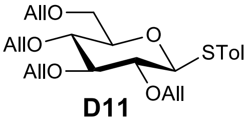 **D11**	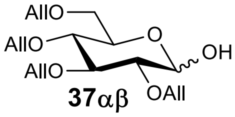 **37αβ**	30	89	1:0.9
10	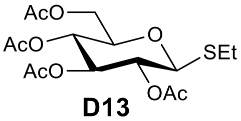 **D13**	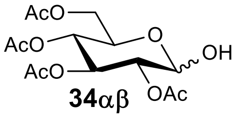 **34αβ**	120	96	1:0.4
11	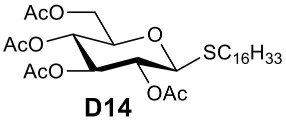 **D14**	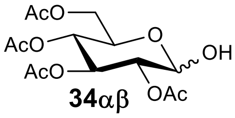 **34αβ**	150	91	1:0.4
12	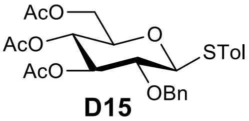 **D15**	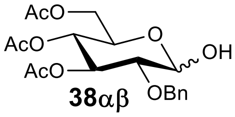 **38αβ**	90	95	1:0.4
13	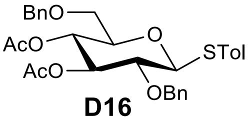 **D16**	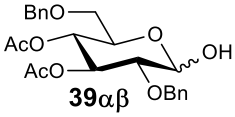 **39αβ**	30	92	1:0.5

[a] Isolated by chromatography. [b] Calculated based on ^{1}H NMR.

F) <u>Synthesis of donor D14:</u>

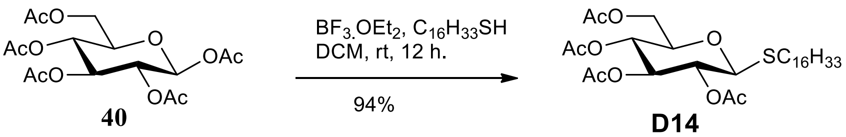

40 → **D14**

A mixture of penta-acetate **40** (5 g, 12.82 mmol) and hexadecanethiol (4.3 mL, 14.10 mmol) in anhydrous dichloromethane (30 mL) was cooled to 0 ^{o}C under nitrogen atmosphere. To this cooled solution, $BF_3.Et_2O$ (4.0 mL, 32.05 mmol) was added drop-wise over 20 min. The reaction mixture was stirred at room temperature for 12 h (solution turned violet brown). After completion of the reaction (TLC), the mixture was cooled to 0 ^{o}C and quenched with saturated aqueous sodium bicarbonate solution. The reaction mixture was then diluted with dichloromethane (50 mL), partitioned and separated. The aqueous layer was further extracted

with dichloromethane (30 mL) and the combined organic layer was washed with water and brine. The organic layer was dried over anhydrous sodium sulphate, filtered and evaporated under reduced pressure. The thick residue thus obtained was purified by flash column chromatography by using 25% ethyl acetate in n-hexane as eluent to get pure hexadecyl thioglycoside **D14** (7.1 g, 94%) as white solid. MP: 85-87 °C; ^{1}H NMR (CDCl3, 500 MHz): δ 0.81 (t, J = 7 Hz, 3H, Alkyl-CH_3), 1.18-1.20 (m, 21H, Alkyl-CH_2), 1.28-1.29 (m, 2H, Alkyl-CH_2), 1.50-1.52 (m, 5H, Alkyl-CH_2), 1.93 (s, 3H, COCH_3), 1.95 (s, 3H, COCH_3), 1.98 (s, 3H, COCH_3), 2.01 (s, 3H, COCH_3), 2.56-2.62 (m, 2H, SCH_2), 3.61-3.65 (m, 1H, H-5), 4.06 (dd, J = 12 and 7 Hz, 1H, H-6$_A$), 4.17 (dd, J = 12 and 5 Hz, 1H, H-6$_B$), 4.40 (d, J = 10 Hz, 1H, H-1), 4.96 (t, J = 10 Hz, 1H, H-2), 5.01 (t, J = 10 Hz, 1H, H-4), 5.15 (t, J = 9 Hz, 1H, H-3); ^{13}C NMR (CDCl$_3$, 125 MHz): δ 14.0, 20.5, 20.59, 20.7, 22.6, 28.8, 29.1, 29.3, 29.5, 29.59, 29.6, 29.68, 30.0, 31.9, 62.2, 68.3, 69.4, 73.9, 75.8, 83.6, 169.3, 169.4, 170.2, 170.6, Anal. Calcd for $C_{30}H_{52}O_9S$: C, 61.20; H, 8.90; found: C, 61.10; H, 8.93.

G) <u>Synthesis of donor D15:</u>

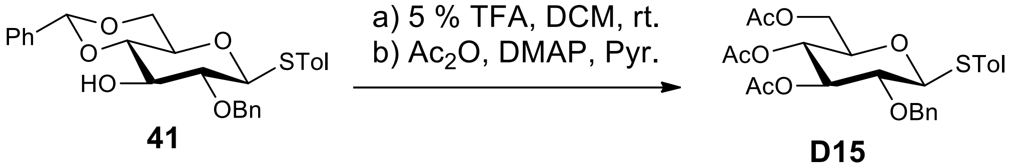

To a solution of thioglycoside **41**[27] (0.5 g, 1.08 mmol) in wet DCM (5 mL) was added a solution of trifluoracetic acid in DCM (1.5 mL; 1/20, v/v). The reaction mixture was stirred at room temperature. When the TLC showed complete consumption of starting material, the reaction was neutralised with triethylamine (0.7 mL) and concentrated under reduced pressure. The residue thus obtained was co-evaporated with toluene (2 x 5 mL) and was dissolved in pyridine (2 mL). Acetic anhydride (0.46 mL, 4.9 mmol) followed by DMAP (0.036 g, 0.29 mmol) were added and the reaction mixture was stirred for 5 hours at room temperature. At completion (TLC), the reaction mixture was concentrated under reduced pressure. The residue thus obtained was purified by column chromatography (EtOAc-hexane, 1:4, v/v) to afford **D15** (0.5 g, 93%) as a white solid. R_f = 0.3 (EtOAc-hexane, 1:4, v/v); Mp : 99-103 °C; ^{1}H NMR (500 MHz, CDCl$_3$): δ 1.81 (s, 3H, COCH_3), 1.93 (s, 3H, COCH_3), 2.01

(s, 3H, COCH_3), 2.28 (s, 3H, CH_3), 3.44 (t, J = 9.45 Hz, 1H, H-2), 3.58-3.61 (m, 1H, H-5), 4.06 (dd, J = 2.0 and 12.0 Hz, 1H, H-6'), 4.17 (dd, J = 5.0 and 12.0 Hz, 1H, H-6), 4.49 (d, J = 11.0 Hz, 1H, 0.5 x CH_2Ph), 4.55 (d, J = 10.0 Hz, 1H, H-1), 4.8 (d, J = 11.0 Hz, 1H, 0.5 x CH_2Ph), 4.89 (t, J = 10.0 Hz, 1H, H-4), 5.13 (t, J = 9.0 Hz, 1H, H-3), 7.05 (d, J = 8.0 Hz, 2H, Ar-H), 7.21-7.23 (m, 3H, Ar-H), 7.26-7.29 (m, 2H, Ar-H), 7.39 (d, J = 8.0 Hz, 2H, Ar-H); ^{13}C NMR (125 MHz, $CDCl_3$): δ 20.64, 20.69, 20.75, 21.16, 62.4, 68.6, 75.1, 75.5, 75.6, 78.3, 87.8, 127.9, 128.1, 128.5, 128.8, 129.8, 133.2, 137.6, 138.4, 169.8, 170.0, 170.6. Anal. Calcd for $C_{26}H_{30}O_8S$: C, 62.14; H, 6.02; found C, 61.98; H, 5.73.

H) **Synthesis of donor D16:**

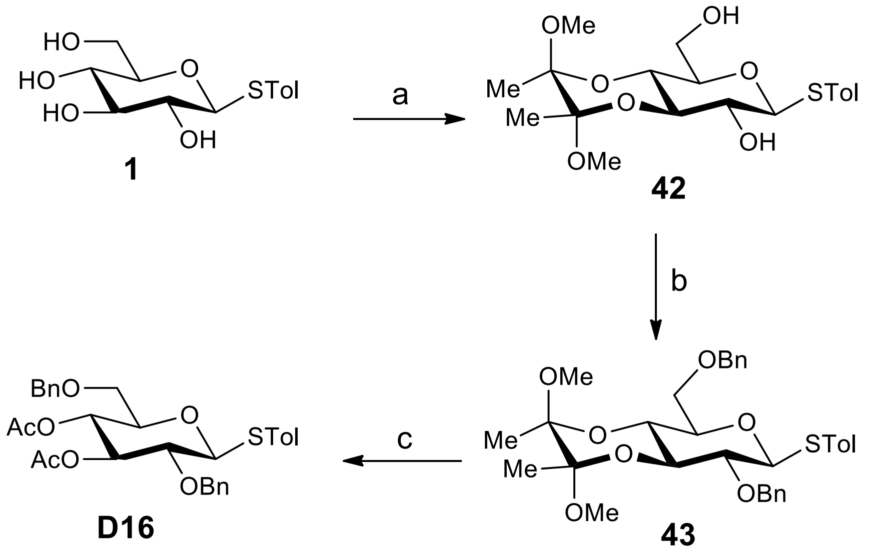

Scheme S2. a) 2,3-butanedione, $CH(OCH_3)_3$, CSA, MeOH, reflux, 3 h, 43 %; b) BnBr, NaH, DMF, 0 °C-rt, 1h, 95 %; c) i. HOAc/H_2O/$(HOCH_2)_2$, 14/6/3, v/v/v, reflux, 1.5 h; ii. Ac_2O, Pyr/DMAP, rt, 2 h, 88 % (after 2 steps).

a) Synthesis of diol 42.

To a solution of tetraol **1**[1] (1 g, 3.5 mmol) in anhydrous MeOH (20 mL), trimethyl orthoformate (1.4 mL, 12.8 mmol), 2,3-butanedione (0.33 mL, 3.7 mmol) and camphorsulfonic acid (0.09 g, 0.39 mmol) were added and the reaction mixture was refluxed for 5 h. When TLC showed complete conversion of the starting material, the reaction mixture

was cooled to room temperature and quenched by adding triethylamine (0.5 mL). The reaction mixture was concentrated under reduced pressure. The crude oil thus obtained was diluted with ethyl acetate (20 mL) and washed with water (2 x 20 mL) followed by brine. The organic layer was collected and dried over anhydrous Na_2SO_4 and concentrated under reduced pressure. The residue thus obtained was purified by column chromatography (EtOAc-hexane, 2:3, v/v) to afford the diol **42** (0.51 g, 1.5 mmol, 43 %) as a white foam. R_f = 0.31 (EtOAc-hexane, 1:3, v/v). ^{1}H NMR (500 MHz, $CDCl_3$): δ 1.21 (s, 3H, C*H*3), 1.25 (s, 3H, C*H*3), 2.27 (s, 3H, C*H*3), 3.16 (s, 3H, OC*H*3), 3.23 (s, 3H, OC*H*3), 3.38 (t, *J* = 9.0 Hz, 1H, H-2), 3.47-3.52 (m, 1H, H-5), 3.54-3.56 (m, 1H, H-4), 3.62-3.70 (m, 2H, H-5 and H-6'), 3.78-3.84 (m, 1H, H-6), 4.43 (d, *J* = 9.0 Hz, 1H, H-1), 7.05 (d, *J* = 8.0 Hz, 2H, Ar-*H*), 7.34 (d, *J* = 8.0 Hz, 2H, Ar-*H*). ^{13}C NMR (125 MHz, $CDCl_3$): δ 17.6, 17.7, 21.18, 29.26, 48.0, 61.4, 65.5, 69.2, 73.3, 78.02, 88.3, 99.6, 99.8, 126.9, 129.9, 133.9, 138.95. Anal. Calcd for $C_{19}H_{28}O_7S$: C, 56.98; H, 7.05; found C, 56.81; H, 7.05.

b) *p*-Tolyl 2,6-di-*O*-benzyl-3,4-di-*O*-(2',3'-dimethoxybutane-2',3'-diyl)-1-thio-β-D-glucopyranoside (43).

A solution of diol **42** (0.5 g, 1.25 mmol) in anhydrous DMF (10 mL) was cooled to 0 °C and sodium hydride (60%, 0.175 g, 4.4 mmol) was added in one portion. The reaction mixture was stirred at room temperature for 15 min. Benzyl bromide (0.34 mL, 2.9 mmol) was then added slowly and stirring was continued at room temperature for 2 h. When TLC showed complete consumption of starting material, the reaction mixture was cooled to 0 °C and the excess of sodium hydride was quenched by addition of MeOH (1 mL). The reaction mixture was diluted with EtOAc (25 mL) and H_2O (10 mL). The layers were collected separately and the DMF/H_2O mixture was extracted with EtOAc (4 x 10 mL). The combined organic layer was washed with water (2 x 10 mL), followed by brine and dried over anhydrous Na_2SO_4. The resulting solution was concentrated under reduced pressure. The residue thus obtained

was purified by column chromatography (EtOAc/hexane, 1:9, v/v) to afford compound **43** as a white solid (0.69 g, 1.19 mmol, 95 %); R_f = 0.45 (EtOAc-hexane, 1:9, v/v); Mp : 95-97 °C; ^{1}H NMR (500 MHz, $CDCl_3$): δ 1.21 (s, 3H, CH_3), 1.26 (s, 3H, CH_3), 2.22 (s, 3H, CH_3), 3.12 (s, 3H, OCH_3), 3.19 (s, 3H, OCH_3), 3.37 (t, *J* = 9.0 Hz, 1H, H-2), 3.53-3.56 (m, 1H, H-6'), 3.63-3.69 (m, 2H, H-4 and H-5), 3.73 (dd, *J* = 1.5 and 11.0 Hz, 1H, H-6), 3.79 (t, *J* = 9.5 Hz, 1H, H-3), 4.47-4.50 (m, 2H, 0.5 x CH_2Ph and H-1), 4.54 (d, *J* = 11.0 Hz, 1H, 0.5 x CH_2Ph), 4.65 (d, *J* = 11.0 Hz, 1H, 0.5 x CH_2Ph), 4.74 (d, *J* = 11.0 Hz, 1H, 0.5 x CH_2Ph), 6.92 (d, *J* = 8.0 Hz, 2H, Ar-*H*), 7.21-7.23 (m, 3H, Ar-*H*), 7.27-7.29 (m, 5H, Ar-*H*), 7.37-7.40 (m, 4H, Ar-*H*), . ^{13}C NMR (125 MHz, $CDCl_3$): δ 17.7, 17.9, 21.1, 47.9, 48.1, 65.7, 68.4, 73.4, 74.9, 75.4, 77.7, 87.2, 99.6, 99.7, 127.4, 127.7, 128.17, 128.25, 128.32, 128.7, 129.6, 133.6, 137.9, 138.47, 138.5 Anal. Calcd for $C_{33}H_{40}O_7S$: C, 68.25; H, 6.94; found C, 68.03; H, 7.15.

c) Synthesis of donor D16.

Compound **43** (0.6 g, 1.03 mmol) was refluxed in a mixture of acetic acid/H_2O/ethylene glycol (15 mL, 14/6/3, v/v/v) for 2 h. When TLC showed complete consumption of the starting material, the reaction mixture was cooled to 0 °C and quenched with aqueous $NaHCO_3$ solution (10 %, 40 mL). The resulting suspension was extracted with EtOAc (3 x 25 mL). The combined organic layer was washed with water (2 x 10 mL), followed by brine, dried over anhydrous $NaSO_4$ and concentrated under reduced pressure. The crude product thus obtained was dissolved in a mixture of acetic anhydride (0.3 mL, 3.2 mmol), pyridine (3 mL), DMAP (0.025 g, 0.2 mmol) and stirred for 16 hrs at room temperature. When TLC showed complete disappearance of the starting material, the mixture was quenched with ice and concentrated under reduced pressure. The residue thus obtained was purified by column chromatography (EtOAc/hexane, 1:9, v/v) to afford donor **D16** as a white solid (0.5 g, 88 % for 2 steps). R_f = 0.4 (EtOAc-hexane, 1:4, v/v); Mp : 100-103 °C; ^{1}H NMR (500 MHz, $CDCl_3$): δ 1.81 (s, 3H, COCH_3), 1.84 (s, 3H, COCH_3), 2.24 (s, 3H, CH_3), 3.44 (t, *J* = 9.5 Hz,

1H, H-2), 3.47-3.55 (m, 2H, H-6 and H-6'), 3.56-3.58 (m, 1H, H-5), 4.41 (d, *J* = 12.0 Hz, 1H, 0.5 x C*H*$_2$Ph), 4.47 (d, *J* = 12.0 Hz, 1H, 0.5 x C*H*$_2$Ph), 4.48 (d, *J* = 11.0 Hz, 1H, 0.5 x C*H*$_2$Ph), 4.58 (d, *J* = 10.0 Hz, 1H, H-1), 4.79 (d, J = 11.0 Hz, 1H, 0.5 x C*H*$_2$Ph), 4.91 (t, *J* = 10.0 Hz, 1H, H-4), 5.13 (t, *J* = 9.0 Hz, 1H, H-3), 6.98 (d, *J* = 8.0 Hz, 2H, Ar-*H*), 7.28-7.21 (m, 10H, Ar-*H*), 7.39 (d, *J* = 8.0 Hz, 2H, Ar-*H*). ^{13}C NMR (125 MHz, $CDCl_3$): δ 20.67, 20.73, 21.13, 69.0, 69.33, 73.5, 75.1, 75.9, 78.4, 87.7, 127.7, 127.8, 127.9, 128.1, 128.3, 128.4, 129.1, 129.8, 132.9, 137.7, 137.9, 138.1, 169.9, 170.1. Anal. Calcd for $C_{31}H_{34}O_7S$: C, 67.62; H, 6.22; found C, 67.73; H, 6.04.

I) General procedure for hydrolysis:

To a solution of thioglycosides (50/100 mg) in wet dichloromethane (5 mL), gold (III) chloride trihydrate (3-10 mol%) was added at room temperature. The resulting pale yellow solution was continued to stir at room temperature until completion of the starting material (monitored by TLC, Table S1). After completion of the reaction, the reaction mixture was quenched with saturated sodium bicarbonate solution until it becomes neutral as indicated by a pH paper. The reaction mixture was extracted with dichloromethane (2 x 10 mL) and washed with water followed by brine. The organic layer was dried over anhydrous sodium sulphate, filtered and concentrated under reduced pressure. The residue thus obtained was purified by flash column chromatography, using ethyl acetate and petroleum ether as eluents. All the new products were fully characterized by spectroscopic techniques and elemental analysis.

1. Hydrolysis of donor D1.

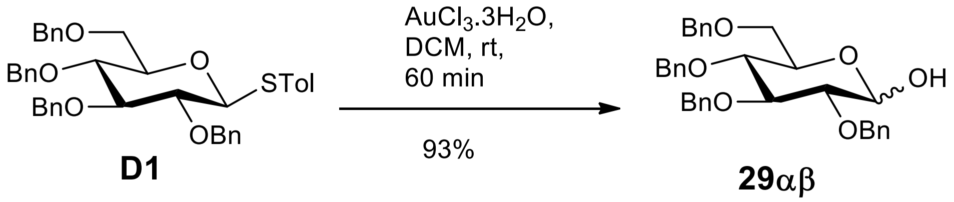

The donor **D1** (0.05 g, 0.08 mmol) on treatment with 3 mol% of $AuCl_3.3H_2O$ (1 mg) in wet DCM (5 mL) gave 39 mg (93 %) of the hydrolyzed product **29αβ**. The spectral data for the product was similar to that of previously reported data.[28]

2. Hydrolysis of donor D2.

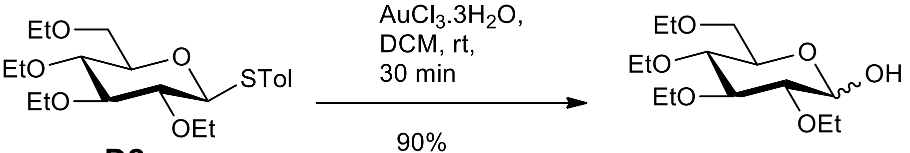

D2 **30αβ**

The donor **D2** (0.1 g, 0.25 mmol) on treatment with 3 mol% of $AuCl_3.3H_2O$ (3 mg) in wet DCM (10 mL) gave 66 mg (90 %) of the hydrolyzed product **30αβ** as a white foam. R_f = 0.35 (EtOAc-hexane, 1:2, v/v). ^{1}H NMR (500 MHz, $CDCl_3$): δ 1.17-1.09 (m, 24H, $CH_2CH_{3(\alpha\beta)}$), 2.97 (dd, J = 8.0 and 9.0 Hz, 1H, H-2_β), 3.12 (t, J = 9.0 Hz, 1H), 3.17-3.21 (m, 1H), 3.22-3.24 (m, 2H), 3.26 (bs, 1H, OH_α), 3.29-3.33 (m, 1H), 3.38-3.43 (m, 1H), 3.43-3.46 (m, 1H), 3.47-3.49 (m, 1H), 3.49-3.50 (m, 1H), 3.50-3.52 (m, 2H), 3.52-3.56 (m, 4H), 3.56-3.57 (m, 0.5H), 3.57-3.59 (m, 1H), 3.69-3.62 (m, 1H), 3.64-3.72 (m, 1H), 3.64-3.66 (m, 1H), 3.66-3.71 (m, 2H), 3.71-3.75 (m, 2H), 3.75-3.77 (m, 1H), 3.77-3.78 (m, 1H), 3.78-3.81 (m, 2H), 3.81-3.87 (m, 2H), 3.88-4.00 (s, 1H, OH_β), 4.51 (d, J = 7.5 Hz, 1H, H-1_β), 5.21 (d, J = 3.0 Hz, 1H, H-1_α). ^{13}C NMR (125 MHz, $CDCl_3$): δ 15.0, 15.03, 15.65, 15.74, 15.76, 15.9, 66.8, 66.9, 68.2, 68.7, 69.2, 70.3, 74.7, 77.7, 78.1, 80.4, 81.4, 83.1, 84.6, 91.4, 97.2. Anal. Calcd for $C_{14}H_{28}O_6$: C, 57.51; H, 9.65; found C, 57.65; H, 9.74.

3. Hydrolysis of donor D3.

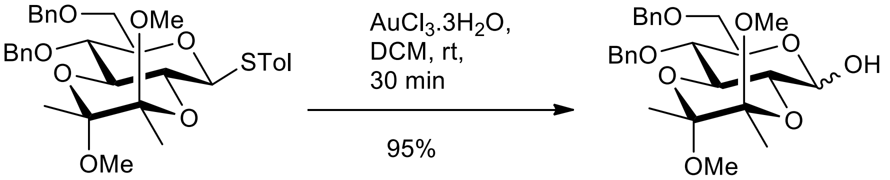

D3 **31αβ**

The donor **D3** (0.1 g, 0.17 mmol) on treatment with 3 mol % $AuCl_3.3H_2O$ (2 mg) in wet DCM (10 mL) gave 75 mg (95 %) of the hydrolyzed product **31αβ** as a white foam. R_f = 0.31 (EtOAc-hexane, 1:4, v/v). ^{1}H NMR (500 MHz, $CDCl_3$): δ 1.15-1.30 (m, 12H, C*H*$_3$ (of α and β isomers)), 3.14 (bs, 1H, $OH_α$), 3.17-3.26 (m, 12H, OC*H*$_3$ (of α and β isomers)), 3.41-3.51 (m, 2H, H-$2_β$ and H-$5_β$), 3.54-3.59 (m, 3H, H-$4_β$, H-$6_β$ and H-$6'_β$), 3.61-3.67 (m, 3H, H-$4_α$, H-$6_α$ and H-$6'_α$), 3.73 (dd, 1H, *J* = 3.0 and 10.0 Hz, 1H, H-$2_α$), 3.80 (t, 1H, *J* = 10.0 Hz, H-$3_β$), 3.93- 3.98 (m, 1H, H-$5_α$), 4.17 (t, *J* = 10.0 Hz, H-$3_α$), 4.39-4.44 (m, 4H, 2 x 0.5 x C*H*$_2$Ph (of α isomer) and 2 x 0.5 x C*H*$_2$Ph (of β isomer)), 4.50-4.54 (m, 2H, 0.5 x C*H*$_2$Ph (of α isomer) and 0.5 x C*H*$_2$Ph (of β isomer)), 4.70 (s, 1H, H-$1_β$), 4.83 (d, *J* = 11.0 Hz, 1H, 0.5 x C*H*$_2$Ph), 4,84 (d, *J* = 11.0 Hz, 1H, 0.5 x C*H*$_2$Ph), 5.18 (s, 1H, H-$1_α$), 7.13-7.14 (m, 4H, Ar-*H*), 7.18-7.24 (m, 16H, Ar-*H*); ^{13}C NMR (125 MHz, $CDCl_3$): δ 17.5, 17.6, 17.8, 17.9, 47.8, 47.9, 47.99, 68.5, 68.6, 68.9, 70.05, 70.6, 71.05, 73.4, 73.49, 73.5, 74.7, 74.8, 75.0, 75.5, 91.3, 94.6, 99.4, 99.5, 99.9, 127.6, 127.65, 127.7, 127.9, 127.92, 127.99, 128.3, 128.35, 137.9, 138.04, 138.2, 138.5. Anal. Calcd for $C_{26}H_{34}O_8$: C, 65.81; H, 7.22; found C, 65.92; H, 7.49.

4. Hydrolysis of donor D6.

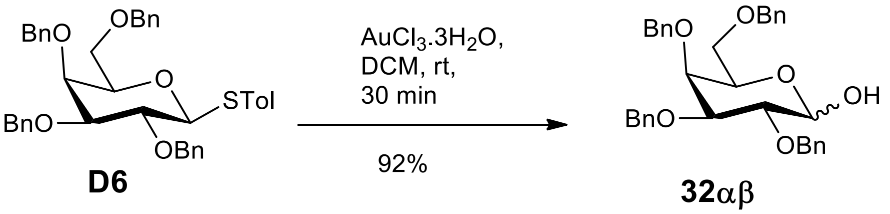

The donor **D6** (0.1 g, 0.15 mmol) on treatment with 3 mol% of $AuCl_3.3H_2O$ (2 mg) in wet DCM (10 mL) gave 77 mg (92 %) of the hydrolyzed product **32αβ**. The spectral data for the product was similar to that of previously reported data.[29]

5. Hydrolysis of donor D7.

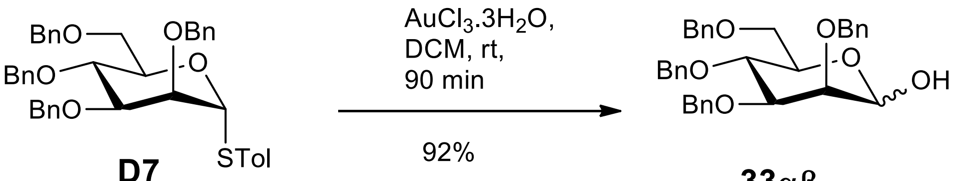

D7 → **33αβ**

The donor **D7** (0.1 g, 0.16 mmol) on treatment with 3 mol% of $AuCl_3.3H_2O$ (2 mg) in wet DCM (10 mL) gave 77 mg (92 %) of the hydrolyzed product **33αβ**. The spectral data for the product was similar to that of previously reported data.[30]

6. Hydrolysis of donor D8.

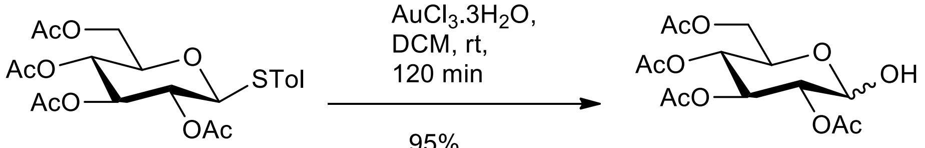

D8 → **34αβ**

The donor **D8** (0.1 g, 0.22 mmol) on treatment with 10 mol% of $AuCl_3.3H_2O$ (8 mg) in wet DCM (10 mL) gave 73 mg (95 %) of the hydrolyzed product **34αβ**. The spectral data for the product was similar to that of previously reported data.[31]

7. Hydrolysis of donor D9.

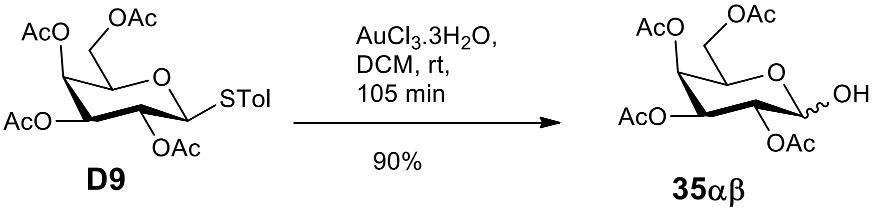

D9 → **35αβ**

The donor **D9** (0.1 g, 0.22 mmol) on treatment with 10 mol% of $AuCl_3.3H_2O$ (8 mg) in wet DCM (10 mL) gave 69 mg (90 %) of the hydrolyzed product **35αβ**. The spectral data for the product was similar to that of previously reported data.[31]

8. Hydrolysis of donor D10.

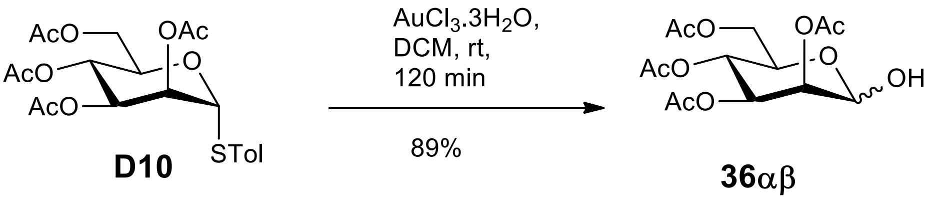

The donor **D10** (0.1 g, 0.22 mmol) on treatment with 10 mol% of $AuCl_3.3H_2O$ (8 mg) in wet DCM (10 mL) gave 68 mg (89 %) of the hydrolyzed product **36αβ**. The spectral data for the product was similar to that of previously reported data.[31]

9. Hydrolysis of donor D11.

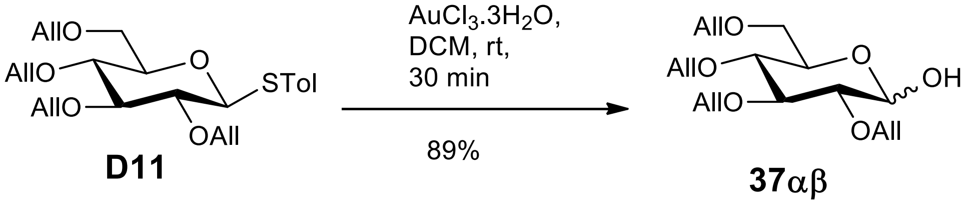

The donor **D11** (0.05 g, 0.11 mmol) on treatment with 3 mol% of $AuCl_3.3H_2O$ (1 mg) in wet DCM (5 mL) gave 34 mg (89 %) of the hydrolyzed product **37αβ**. The spectral data for the product was similar to that of previously reported data.[32]

10. Hydrolysis of donor D13.

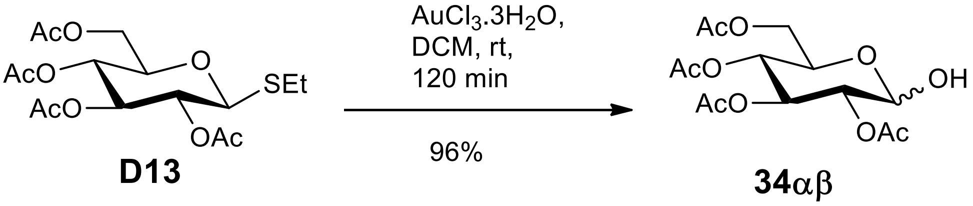

The donor **D13**[33] (0.1 g, 0.26 mmol) on treatment with 10 mol% of $AuCl_3.3H_2O$ (9 mg) in wet DCM (10 mL) gave 85 mg (96 %) of the hydrolyzed product **34αβ**. The spectral data for the product was similar to that of previously reported data.[31]

11. Hydrolysis of donor D14.

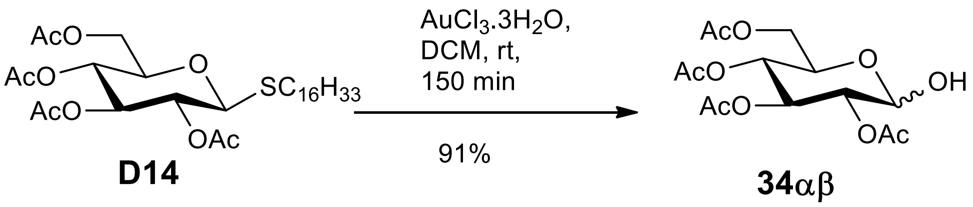

The donor **D14** (0.1 g, 0.17 mmol) on treatment with 10 mol% of $AuCl_3.3H_2O$ (7 mg) in wet DCM (10 mL) gave 54 mg (91 %) of the hydrolyzed product **34αβ**. The spectral data for the product was similar to that of previously reported data.[31]

12. Hydrolysis of donor D15.

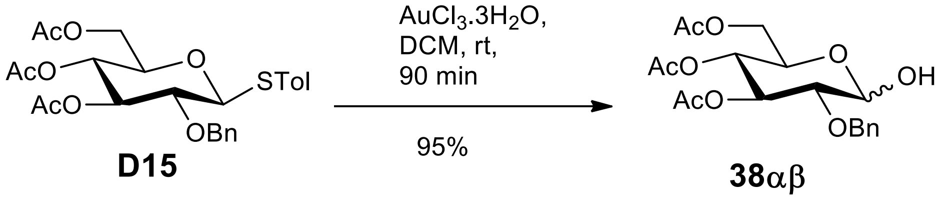

D15 **38αβ**

The donor **D15** (0.1 g, 0.19 mmol) on treatment with 3 mol% of $AuCl_3.3H_2O$ (2 mg) in wet DCM (10 mL) gave 75 mg (95 %) of the hydrolyzed product **38αβ**. The spectral data for the product was similar to that of previously reported data.[34]

13. Hydrolysis of donor D16.

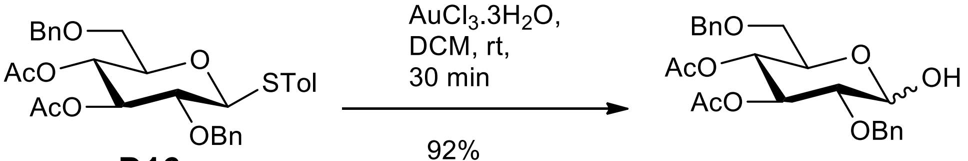

D16 **39αβ**

The donor **D16** (0.1 g, 0.18 mmol) on treatment with 3 mol% of $AuCl_3.3H_2O$ (2 mg) in wet DCM (10 mL) gave 74 mg (92 %) the hydrolyzed product **39αβ**. The spectral data for the product was similar to that of previously reported data.[35]

J) References:

(1) S. K. Veleti, J. J. Lindenberger, S. Thanna, D. R. Ronning, S. J. Sucheck, *J. Org. Chem.* 2014, **79**, 9444.

(2) (a) R. R. France, N. V. Rees, J. D. Wadhawan, A. J. Fairbanks, R. G. Compton, *Org. Biomol. Chem.* 2004, **2**, 2188. (b) A. P. Dieskau, B. Plietker, *Org. Lett.* 2011, **13**, 5544.

(3) T. Nokami, A. Shibuya, H. Tsuyama, S. Suga, A. A. Bowers, D. Crich, J. –i. Yoshida, *J. Am. Chem. Soc.* 2007, **129**, 10922.

(4) S. Buda, M. Nawoj, P. Golebiowska, K. Dyduch, A. Michalak, J. Mlynarski, *J. Org. Chem.* 2015, **80**, 770.

(5) Y. Geng, A. Kumar, H. M. Faidallah, H. A. Albar, I. A. Mhkalid, R. R. Schmidt, *Angew. Chem.* 2013, **125**, 10273; *Angew. Chem. Int. Ed.* 2013, **52**, 10089.

(6) T. K. Pradhan, C. C. Lin, K. -K. T. Mong, *Org. Lett*. 2014, **16**, 1474.

(7) (a) P. L. Garegg, J. -L. Maloisel, S. Oscarson, *Synthesis* 1995, 409. (b) S. Malik, K. P. R. Kartha, *Synlett* 2009, **9**, 1809.

(8) D. Crich, A. U. Vinod, *J. Org. Chem*. 2005, 70, 1291.

(9) S. Hsieh, M. Jan, L. N. Patkar, C. Chen, C. Lin, *Carbohydr. Res*. 2005, **340**, 49.

(10) A. L. Mattson, A. K. Michel, M. J. Cloninger, *Carbohydr. Res.* 2012, **347**, 142.

(11) X. -S. Ye, C. -H. Wong, *J. Org. Chem.* 2000, **65**, 2410.

(12) K. Toshima, H. Nagai, K. -i. Kasumi, K. Kawahara, S. Matsumura, *Tetrahedron* 2004, **60**, 5331.

(13) S. S. Weng, Y. D. Lin, C. T. Chen, *Org. Lett*. 2006, **8**, 5633.

(14) A. P. Dieskau, B. Plietker, *Org. Lett.* 2011, **13**, 5544.

(15) C. Chao, M. Chen, S. Lin, K. T. Mong, *Carbohydr. Res*. 2008, **343**, 957.

(16) Z. Pakulski, *Synthesis* 2003, **13**, 2074.

(17) H. Kondo, S. Aoki, Y. Ichikawa, R. L. Halcomb, H. Ritzen, C. -H. Wong, *J. Org. Chem.* 1994, **59**, 864.

(18) Z. Zhang, I. R. Ollman, X. -S. Ye, R. Wischnat, T. Baasov, C.-H. Wong, *J. Am. Chem. Soc*. 1999, **121**, 734.

(19) S. Manabe, Y. Ito, *J. Org. Chem.* 2013, **78**, 4568.

(20) A. M. Riley, D. J. Jenkins, B. V. L. Potter, *J. Am. Chem. Soc.* 1995, **117**, 3300.

(21) V. M. Dhurandhare, G. P. Mishra, S. Lama, C. –C. Wang, *Org. Biomol. Chem.* 2015, **13**, 9457.

(22) M. P. DeNinno, J. B. Etienne, K. C. Duplantier, *Tetrahedron Lett.* 1995, **36**, 669.

(23) S. Hosono, W. -S. Kim, H. Sasai, M. Shibasaki, *J. Org. Chem.* 1995, **60**, 4.

(24) G. Bhosekar, C. Murali, R. G. Gonnade, M. S. Shashidhar, M. M. Bhadbhade, *Cryst. Growth Des.* 2005, **5**, 1977.

(25) I. Damager, C. E. Olsen, B. L. Moeller, M. S. Motawia, *Carbohydr. Res.* 1999, **320**, 19.

(26) A-H. A. Chu, A. Minciunescu, V. Montanari, K. Kumar and C. S. Bennett, *Org. Lett.* 2014, **16**, 1780.

(27) C. C. Wang, S. S. Kulkarni, J. -C Lee, S. Y. Luo, S. –C. Hung, *Nat. Protoc.* 2008, **3**, 97.

(28) S. R. Koppolu, R. Niddana, R. Balamurugan. *Org. Biomol. Chem.*, 2015, **13**, 5094.

(29) P. C. B. Page, Y. Chan, J. Liddle, M. R. J. Elsegood, *Tetrahedron* 2014, **70**, 7283.

(30) M. Matwiejuk, J. Thiem, *Eur. J. Org. Chem.* 2011, 5860.

(31) S. M. Andersen, M. Heuckendorff, H. H. Jensen, *Org. Lett.* 2015, **17**, 944.

(32) P. Wei, D. Zhang, Z. Gao, W. Cai, W. Xu, L. Tang, G. Zhao, *Synth. Commun.* 2015, **45**, 1457.

(33) B. Mukhopadhyay, K. P. R. Kartha, D. A. Russell, R. A. Field. *J. Org. Chem.* 2004, **69**, 7758.

(34) N. J. Davis, S. L. Flitsch, *J. Chem. Soc. Perkin Trans. I*, 1994, 359.

(35) R. D. Marwood, D. J. Jenkins, V. Correa, C. W. Taylor, B. V. L. Potter, *J. Med. Chem.* 2000, **43**, 4278.

Electronic Supplementary Information (ESI) for

A Versatile Glycosylation Strategy via Au(III) Catalyzed Activation of Thioglycoside Donors

Amol M. Vibhute, Arun Dhaka, Vignesh Athiyarath and Kana M. Sureshan*

School of Chemistry, Indian Institute of Science Education and Research Thiruvananthapuram, KERALA-695016, India, E-mail: kms@iisertvm.ac.in.

INDEX

1. NMR data of **D2**	S3-S7	21. NMR data of **11β**	S88-S92
2. NMR data of **D3**	S8-S12	22. NMR data of **12α**	S93-S97
3. NMR data of **D5**	S13-S15	23. NMR data of **12β**	S98-S102
4. NMR data of **D11**	S16-S20	24. NMR data of **13α**	S103-S107
5. NMR data of **2**	S21-S25	25. NMR data of **14αβ**	S108-S111
6. NMR data of **3α**	S26-S27	26. NMR data of **15αβ**	S112-S115
7. NMR data of **3β**	S28-S29	27. NMR data of **16αβ**	S116-S121
8. NMR data of **4αβ**	S30-S31	28. NMR data of **17β**	S122-S123
9. NMR data of **5αβ**	S32-S33	29. NMR data of **18β**	S124
10. NMR data of **6α**	S34-S38	30. NMR data of **19β**	S125
11. NMR data of **6β**	S39-S43	31. NMR data of **20α**	S126-S127
12. NMR data of **7α**	S44-S48	32. NMR data of **21α**	S128-S133
13. NMR data of **7β**	S49-S53	33. NMR data of **21β**	S134-S138
14. NMR data of **8α**	S54-S59	34. NMR data of **22αβ**	S139-S144
15. NMR data of **8β**	S60-S64	35. NMR data of **23α**	S145-S149
16. NMR data of **9α**	S65-S69	36. NMR data of **23β**	S150-S154
17. NMR data of **9β**	S70-S74	37. NMR data of **24α**	S155-S159
18. NMR data of **10αβ** (α major)	S75-S80	38. NMR data of **24β**	S160-S164
19. NMR data of **10αβ** (β major)	S81-S82	39. NMR data of **25αβ**	S165-S166
20. NMR data of **11α**	S83-S87	40. NMR data of **26αβ**	S167-S172

41. NMR data of **D12**	S173-S175	53. NMR data of **32αβ**	S214
42. NMR data of **27αβ**	S176-S177	54. NMR data of **33αβ**	S215
43. NMR data of **28α**	S178-S183	55. NMR data of **34αβ**	S216
44. NMR data of **28β**	S184-S189	56. NMR data of **35αβ**	S217
45. NMR data of **D14**	S190-S194	57. NMR data of **36αβ**	S218
46. NMR data of **D15**	S195-S197	58. NMR data of **37αβ**	S219
47. NMR data of **42**	S198-S200	59. NMR data of **38αβ**	S220
48. NMR data of **43**	S201-S203	60. NMR data of **39αβ**	S221
49. NMR data of **D16**	S204-S206	61. NMR data of **PhSSPh**	S222
50. NMR data of **29αβ**	S207	62. NMR data of **PhSH**	S223
51. NMR data of **30αβ**	S208-S210	63. NMR data of **TolSSTol**	S224
52. NMR data of **31αβ**	S211-S213	64. NMR data of ***p*-TolSH**	S225

^{1}H NMR of D2 in $CDCl_3$

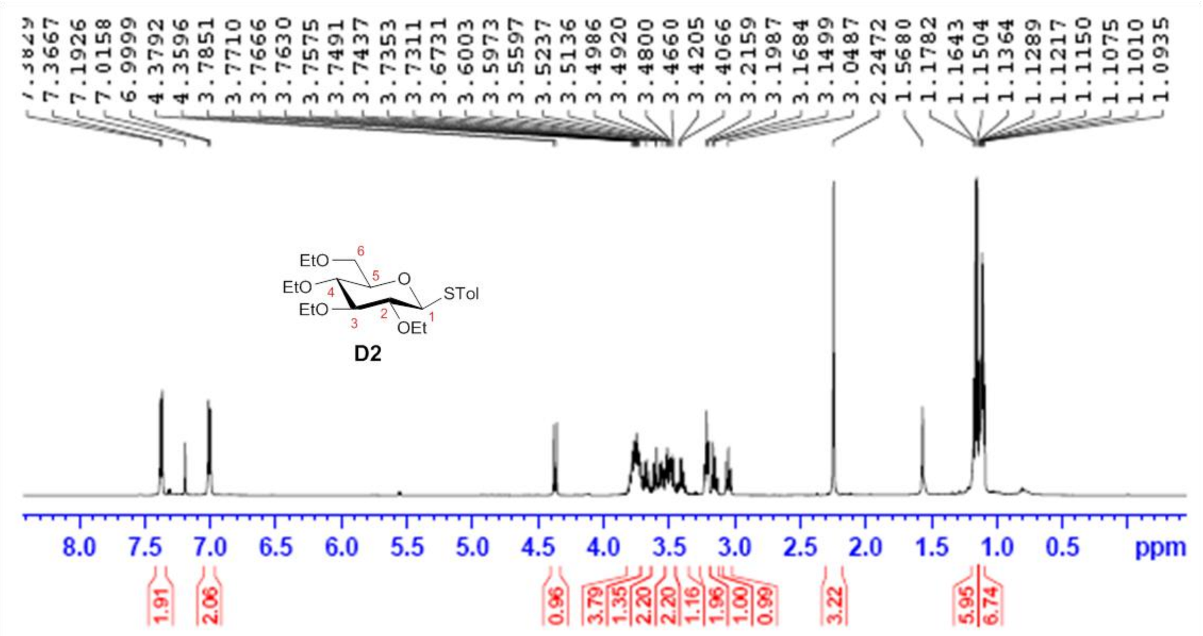

zoom

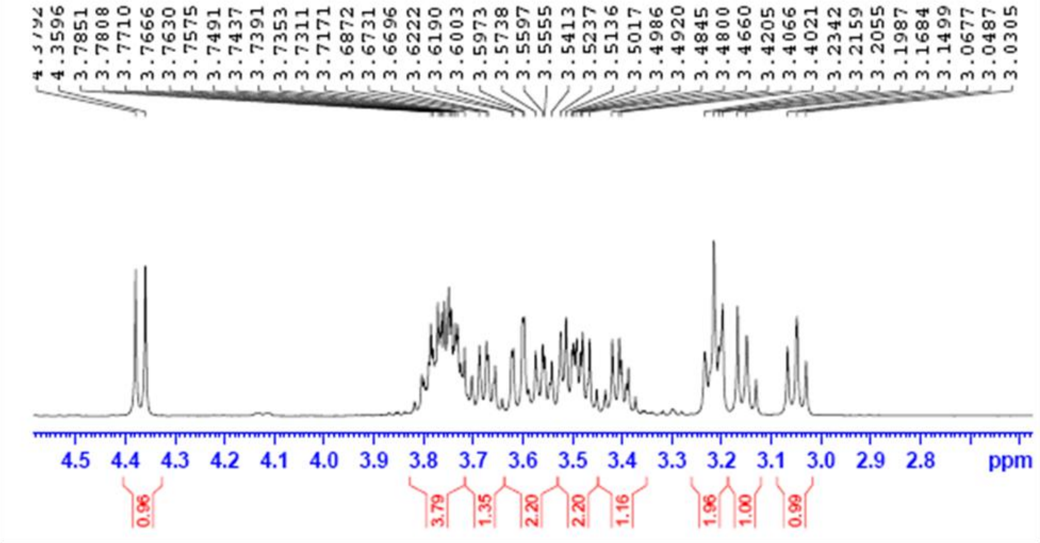

COSY NMR of D2 in $CDCl_3$

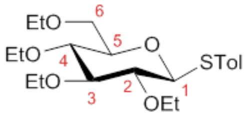

D2

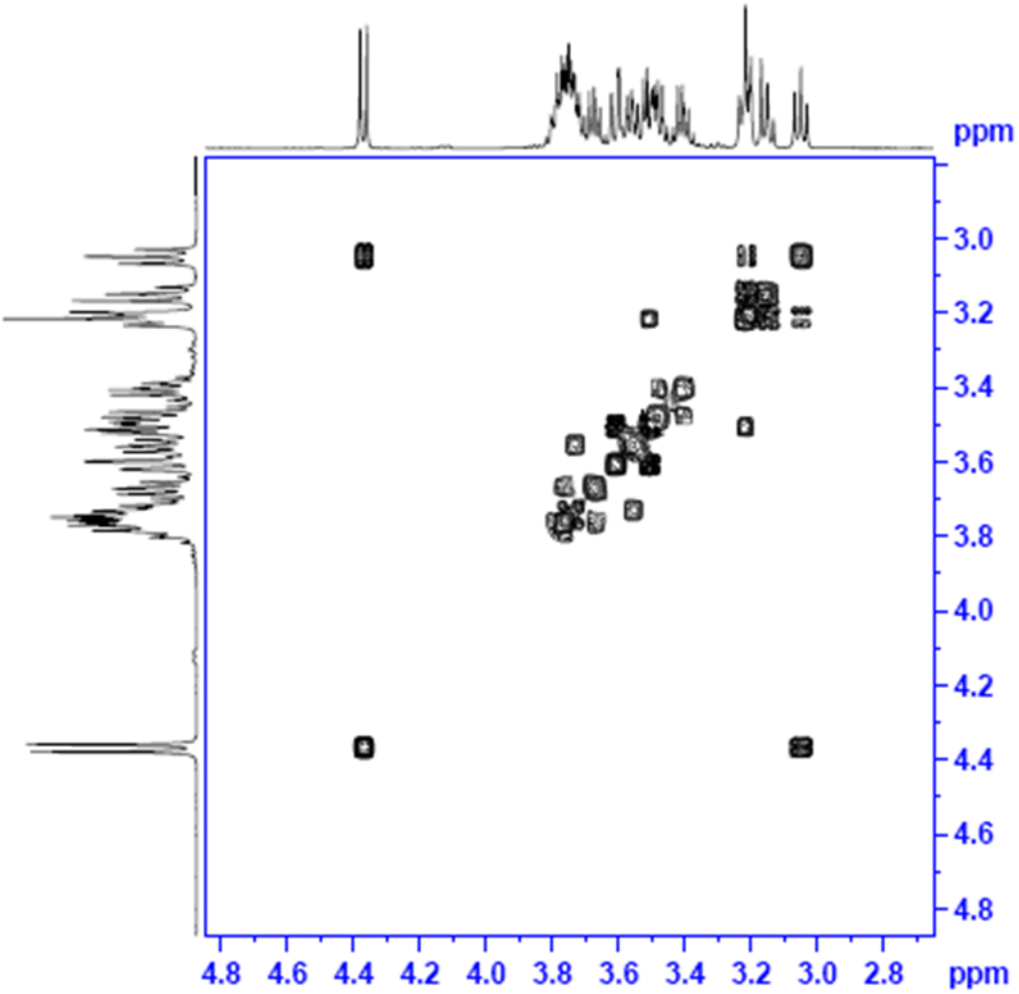

^{13}C NMR of D2 in $CDCl_3$

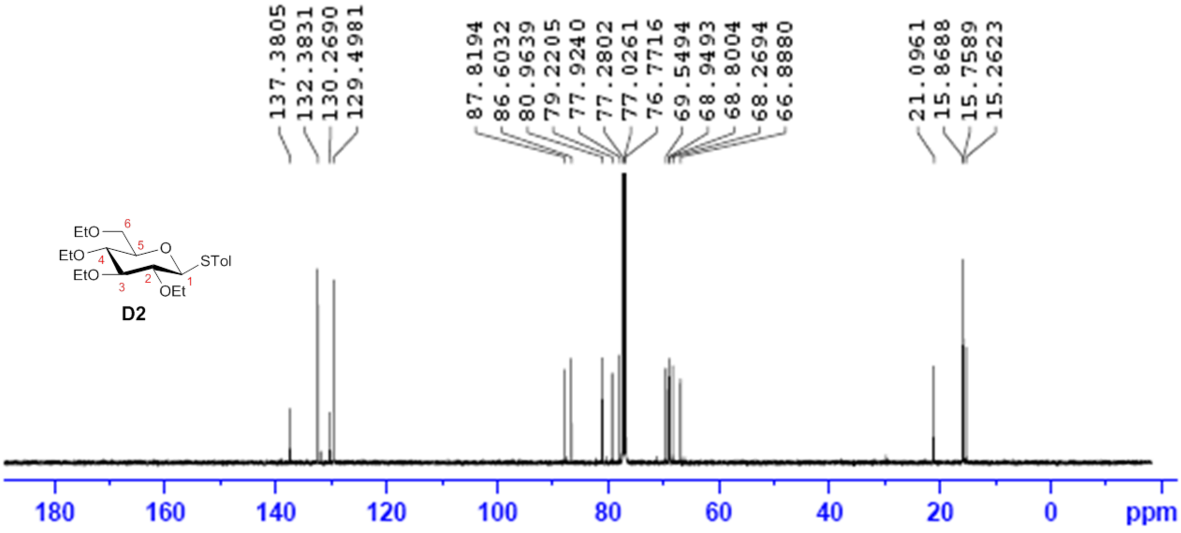

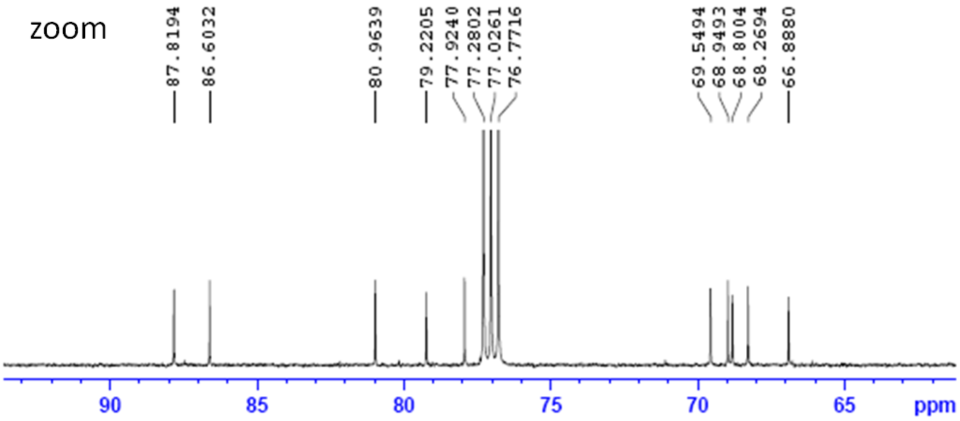

DEPT NMR of D2 in CDCl$_3$

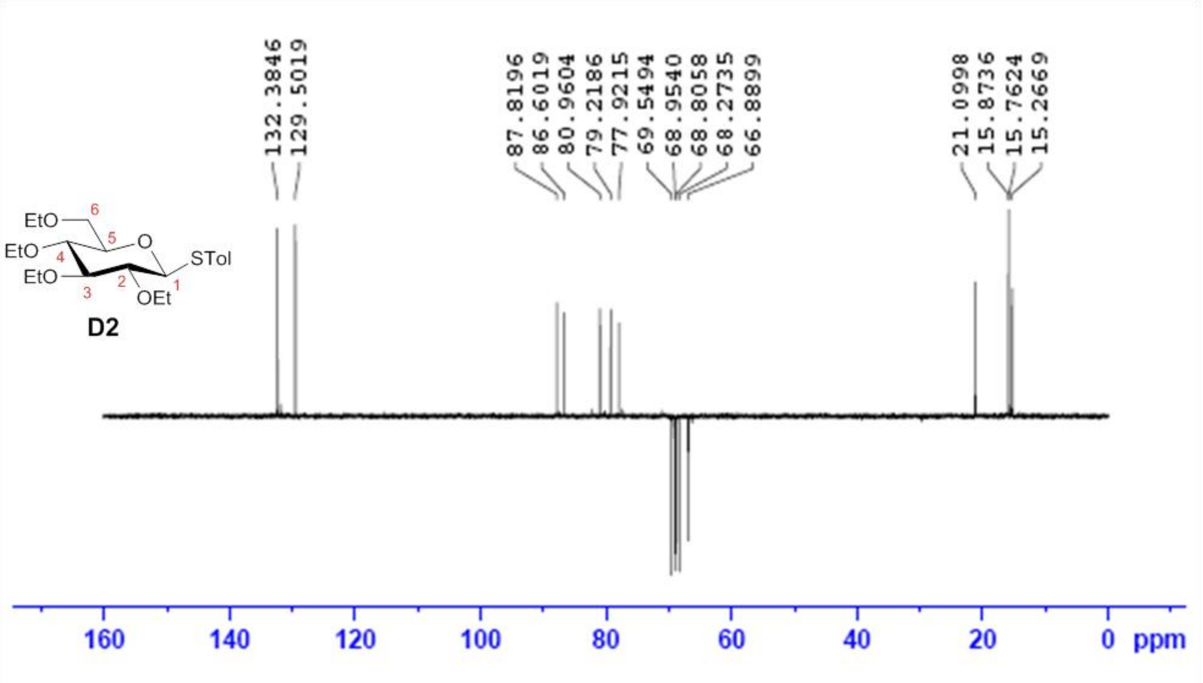

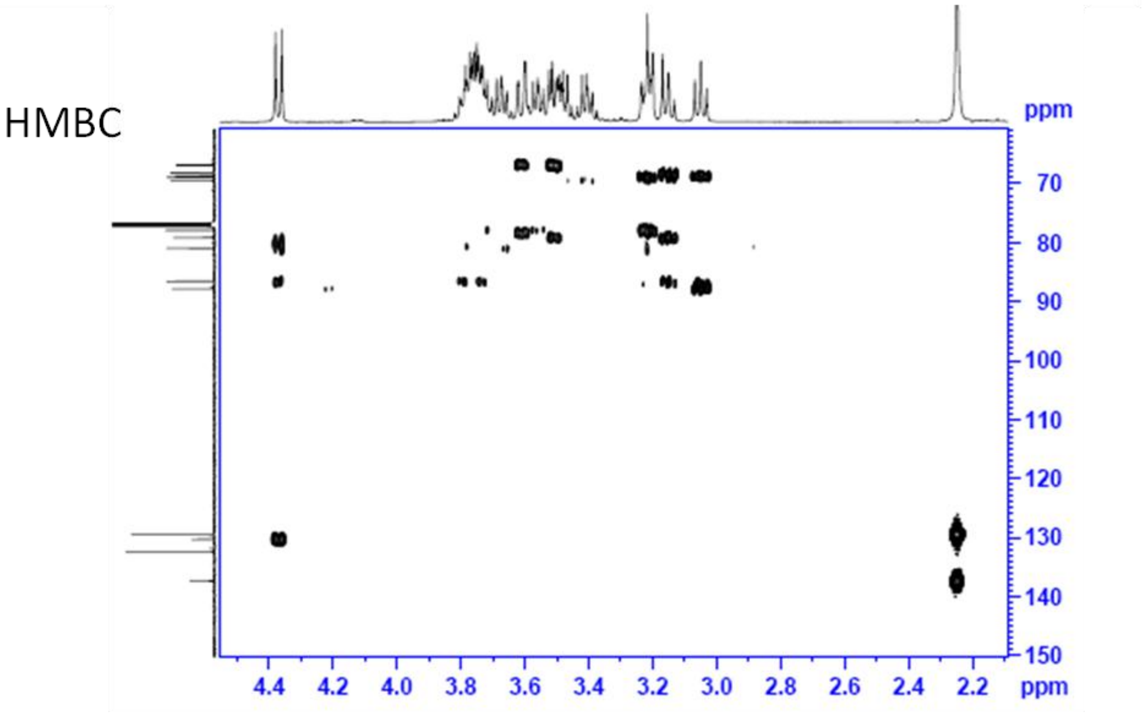

HMQC NMR of D2 in $CDCl_3$

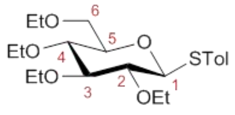

D2

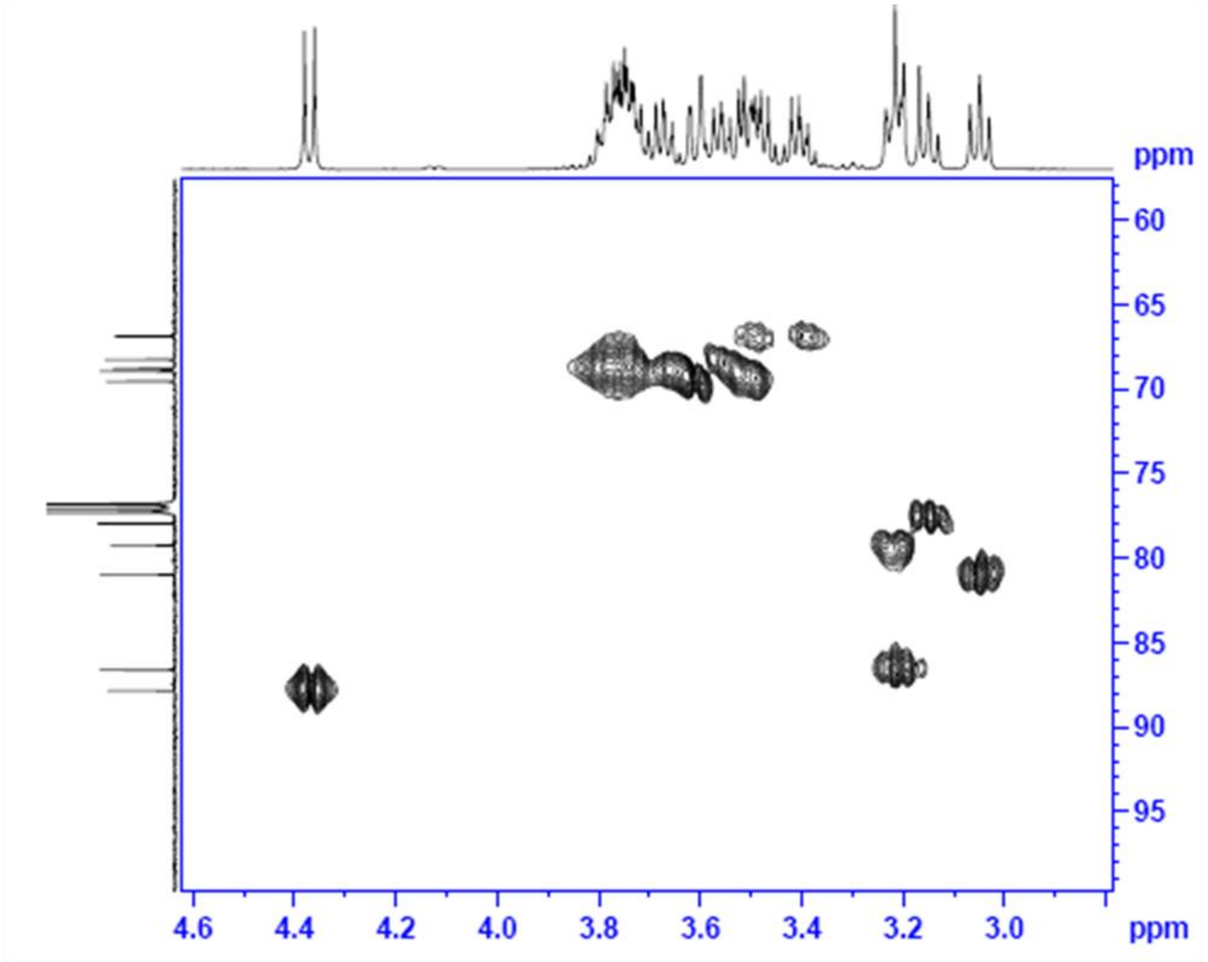

^{1}H NMR of D3 in $CDCl_3$

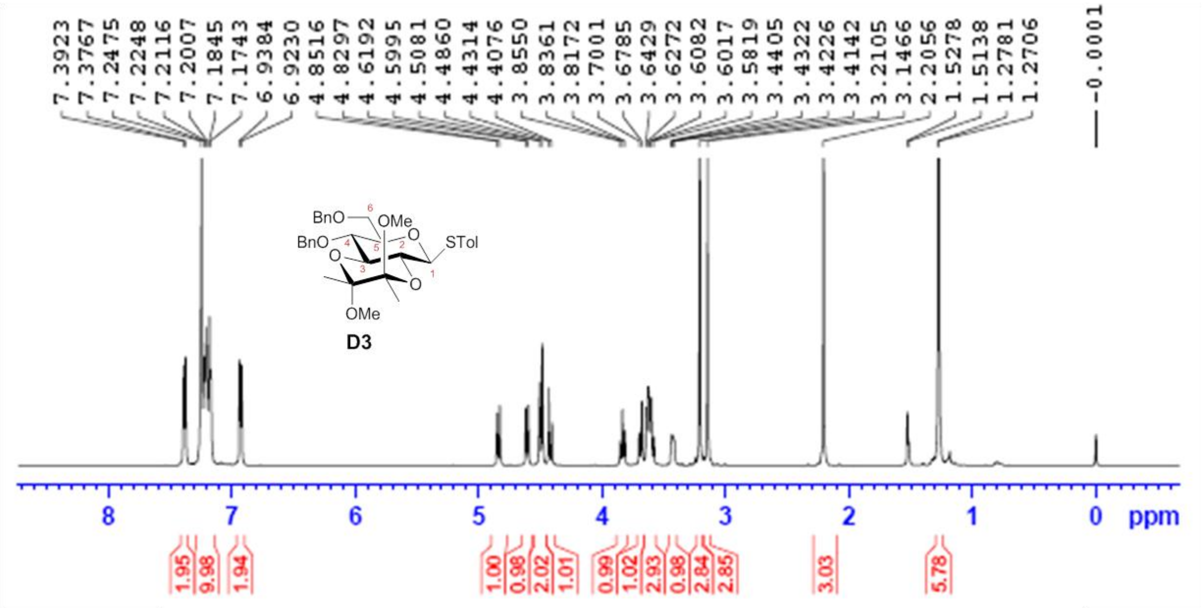

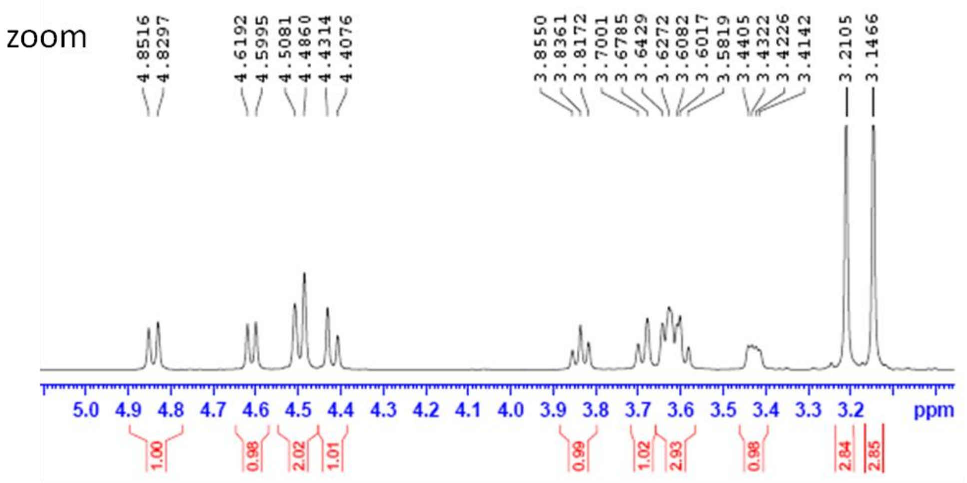

COSY NMR of D3 in $CDCl_3$

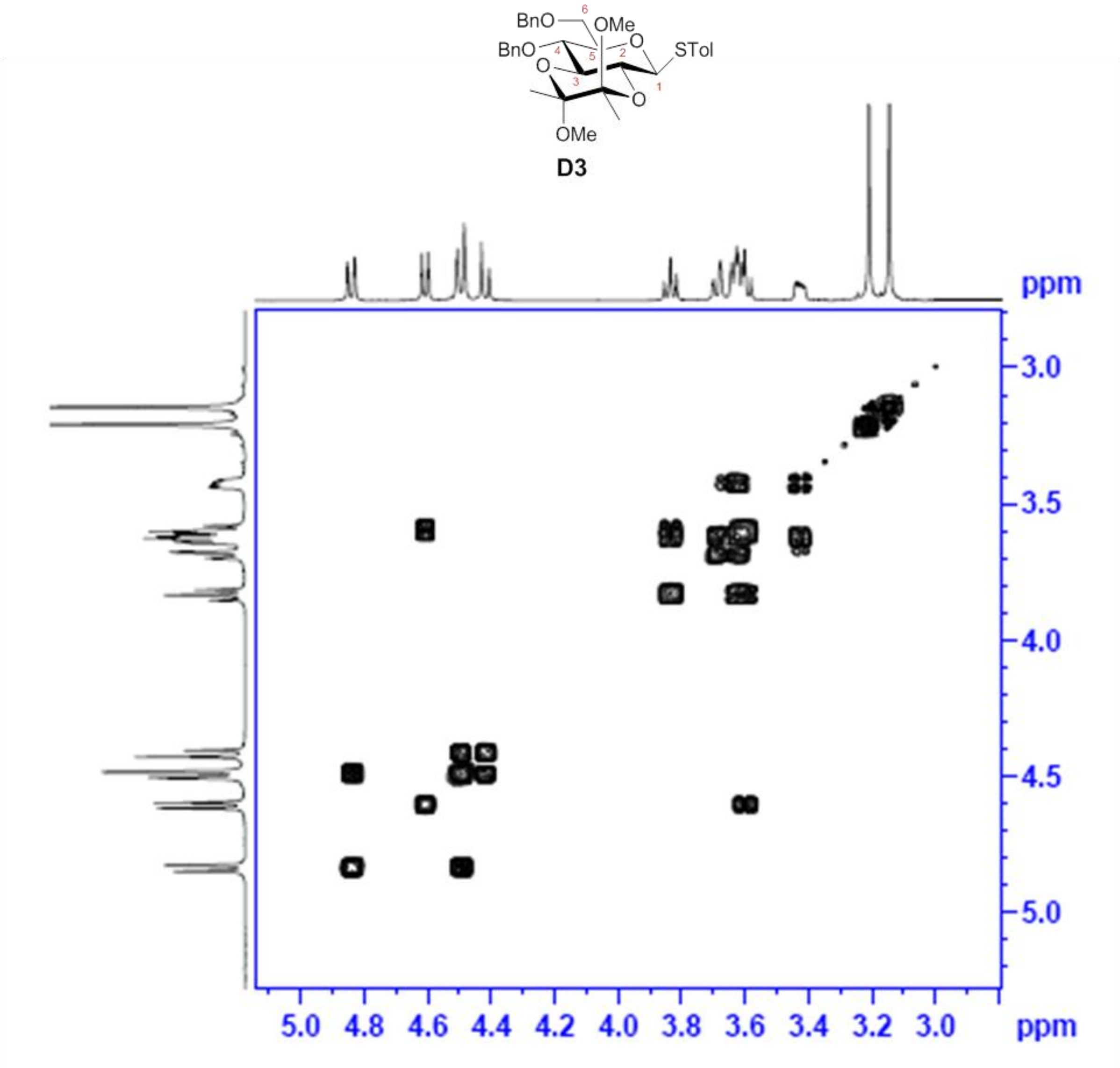

^{13}C NMR of D3 in $CDCl_3$

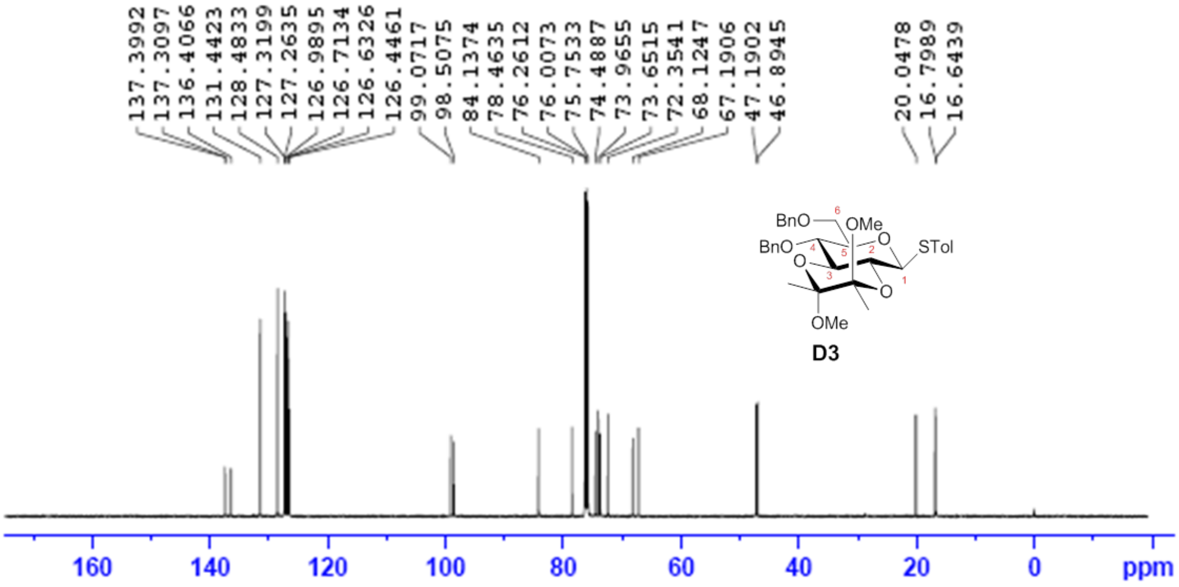

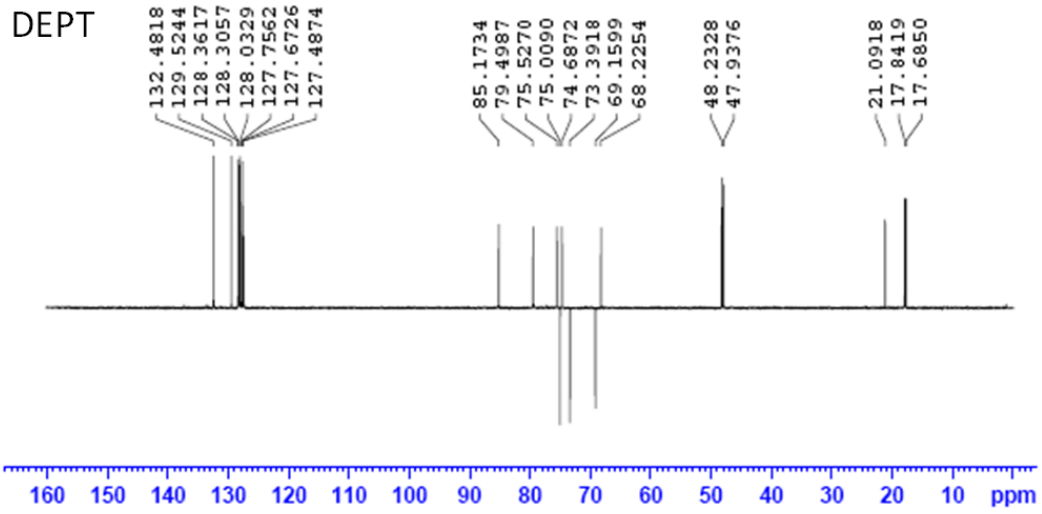

HMBC NMR of D3 in $CDCl_3$

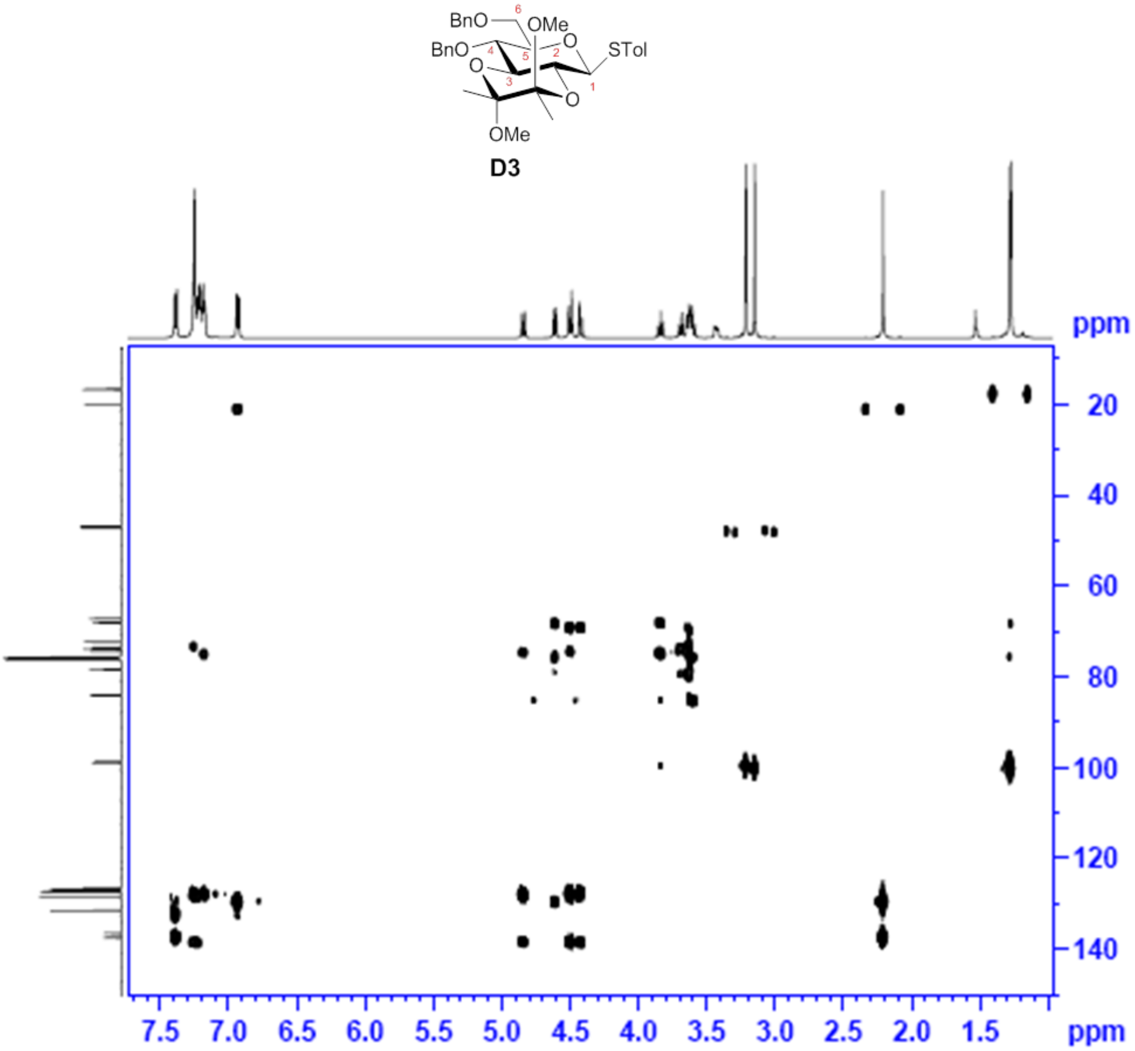

HMQC NMR of D3 in $CDCl_3$

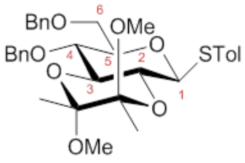

D3

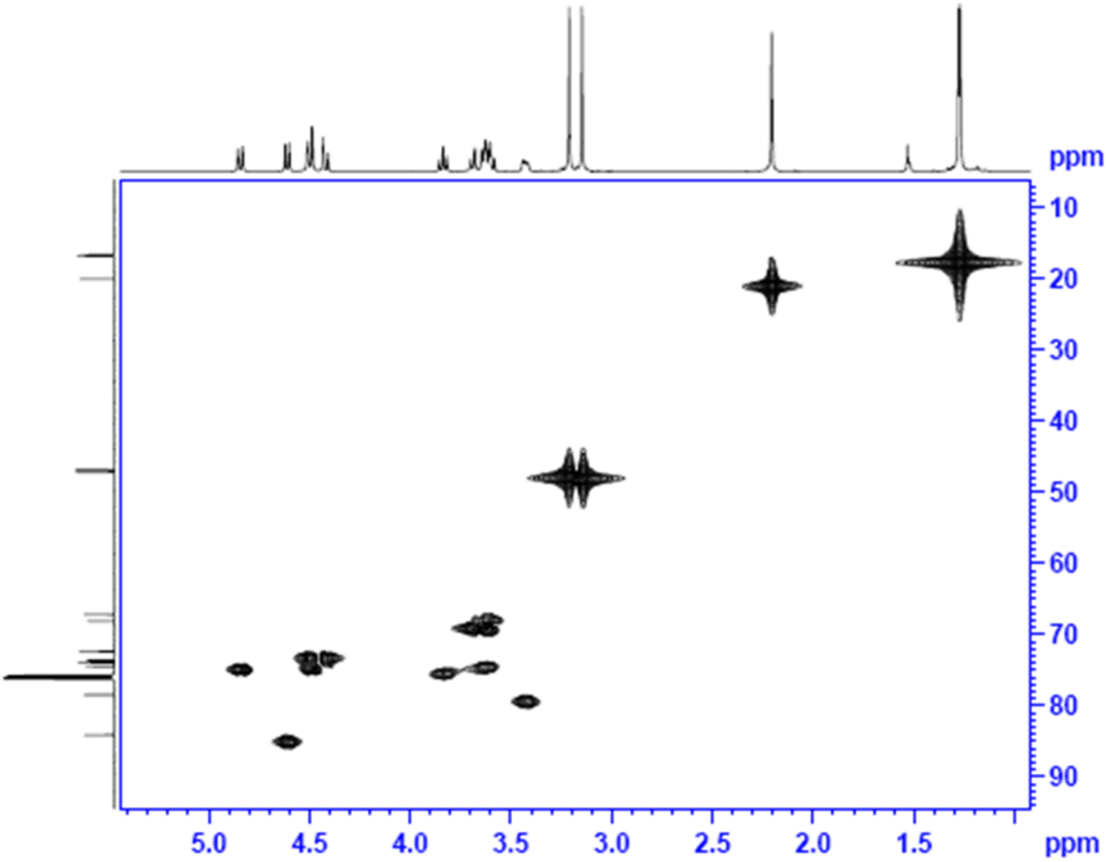

^{1}H NMR of D5 in $CDCl_3$

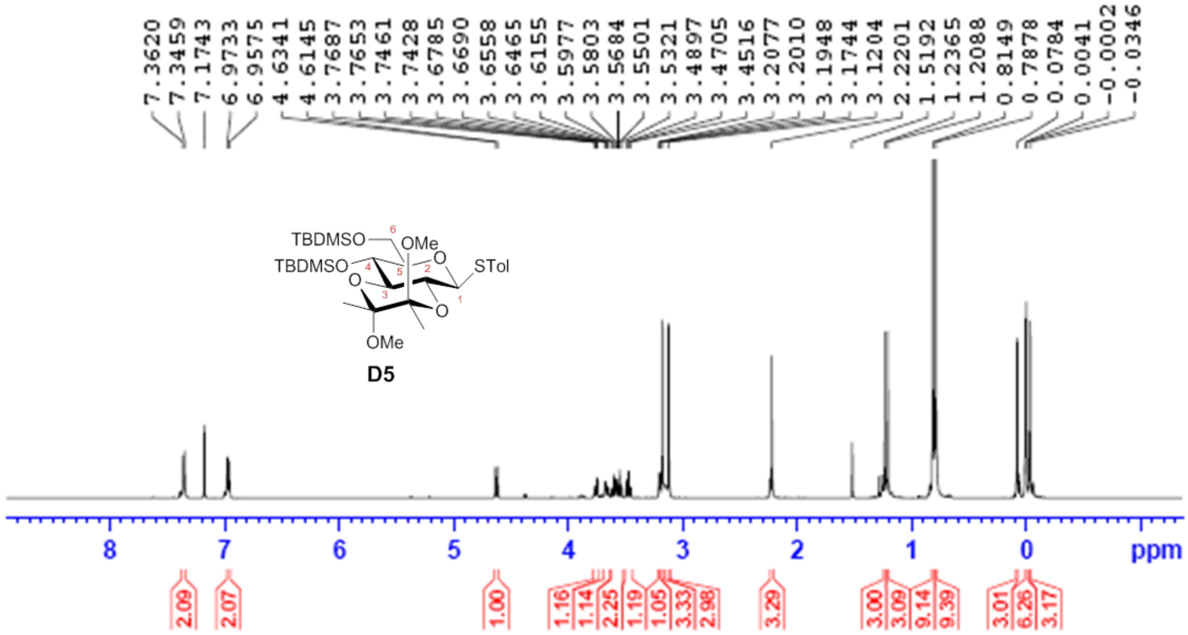

zoom

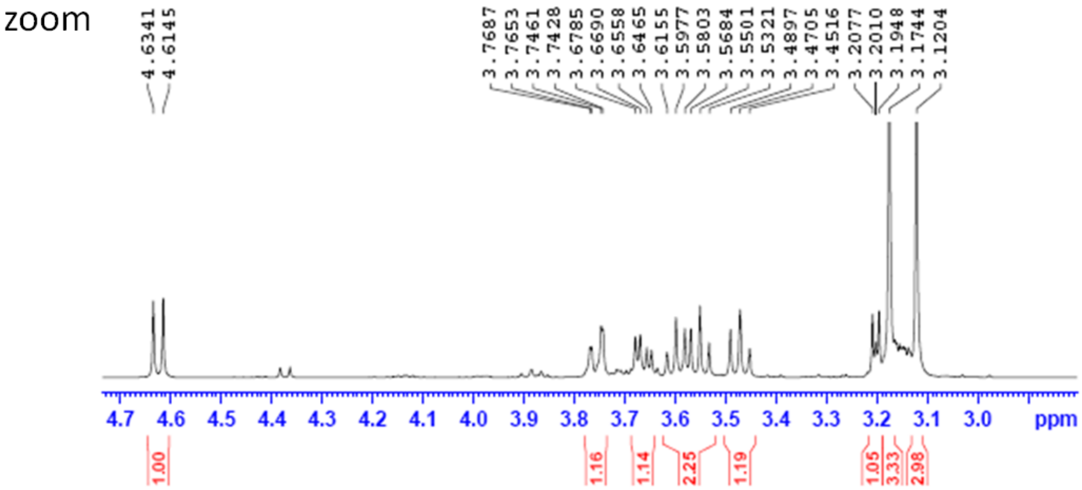

COSY NMR of D5 in $CDCl_3$

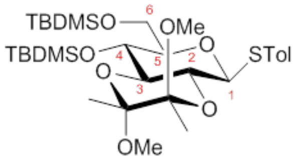

D5

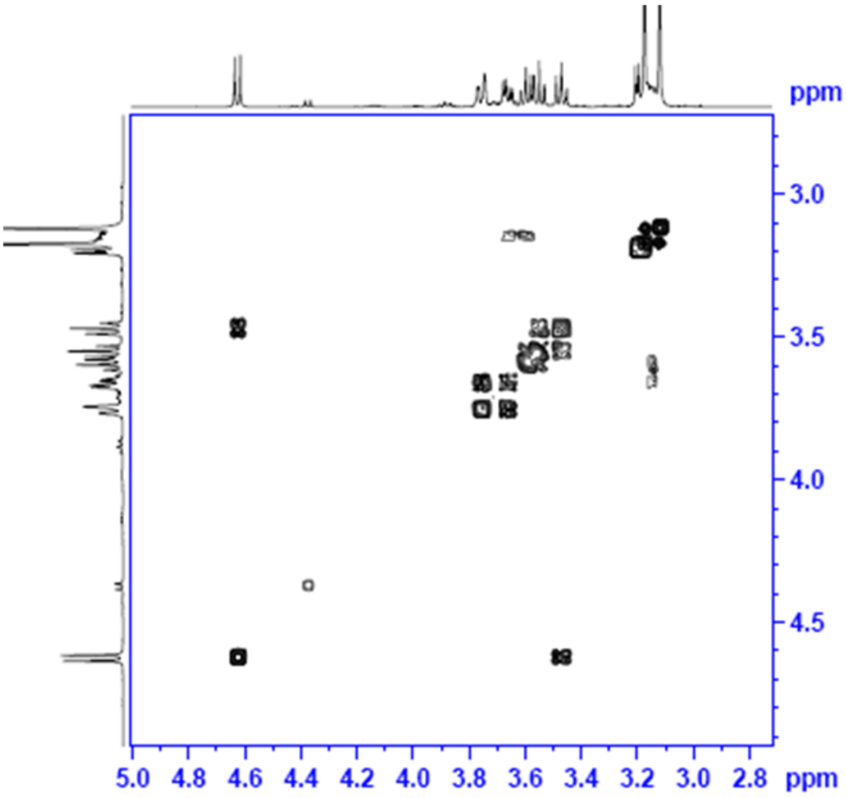

^{13}C NMR of D5 in $CDCl_3$

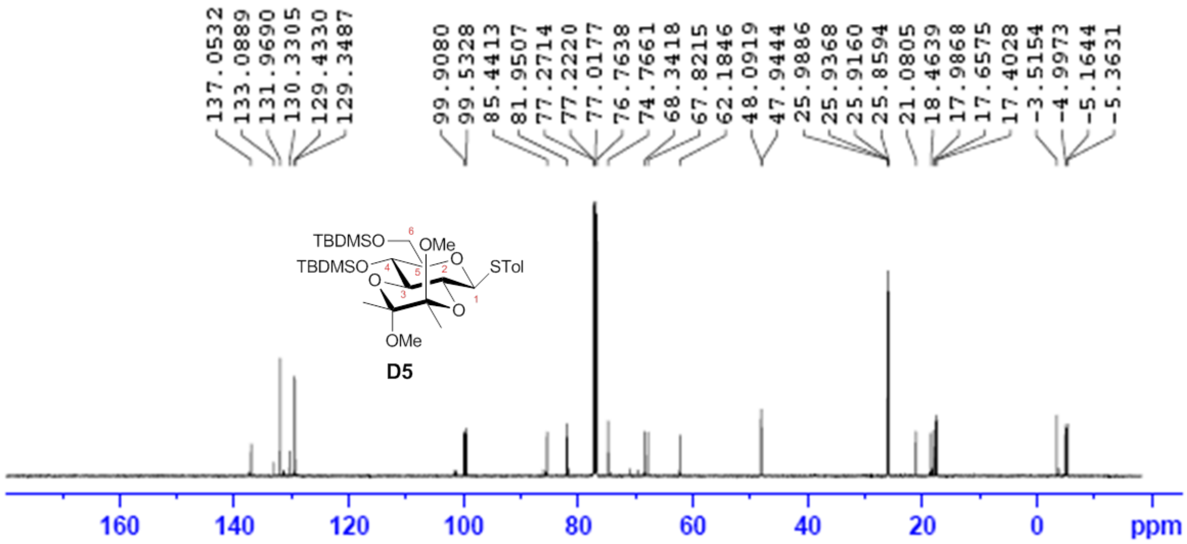

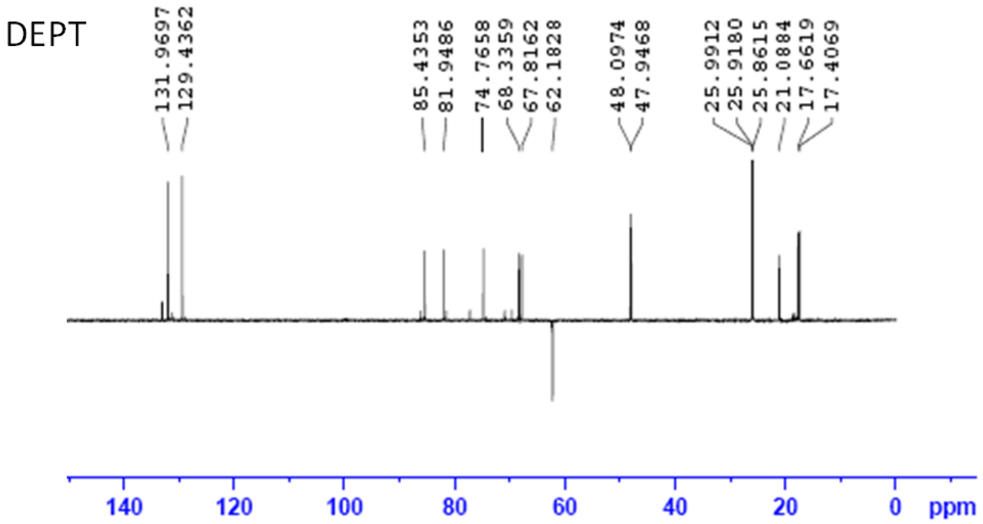

^{1}H NMR of D11 in $CDCl_3$

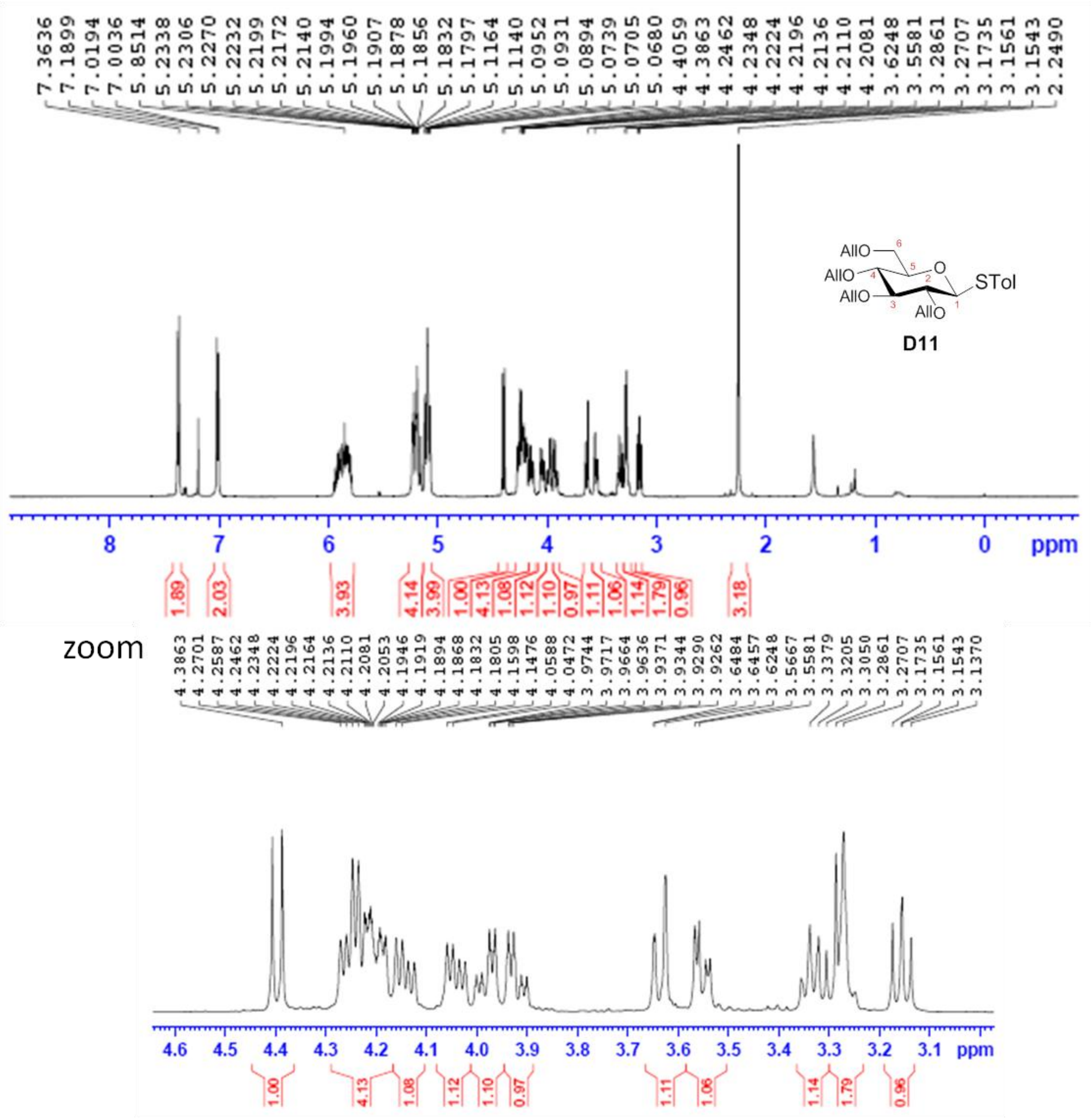

COSY NMR of D11 in $CDCl_3$

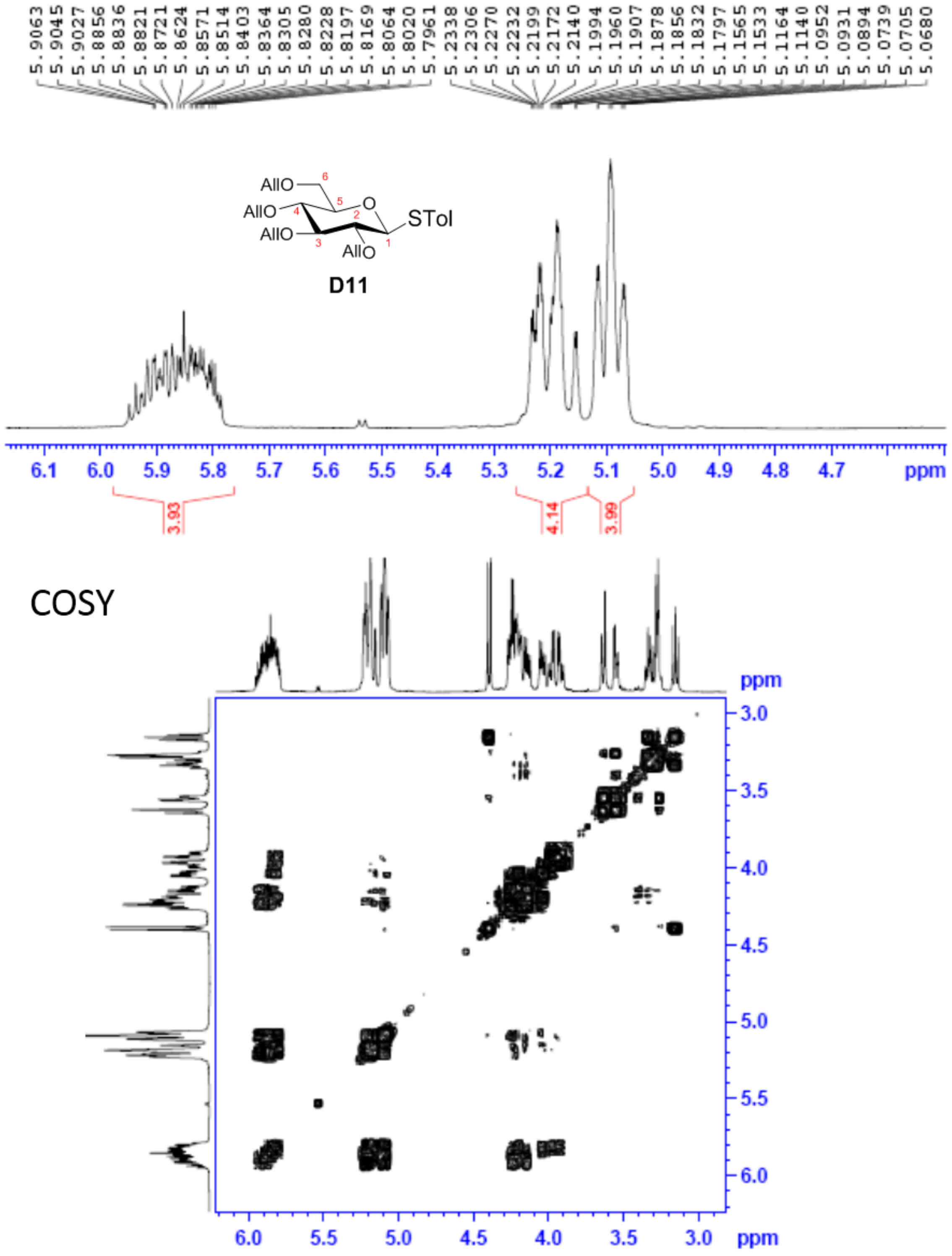

^{13}C NMR of D11 in $CDCl_3$

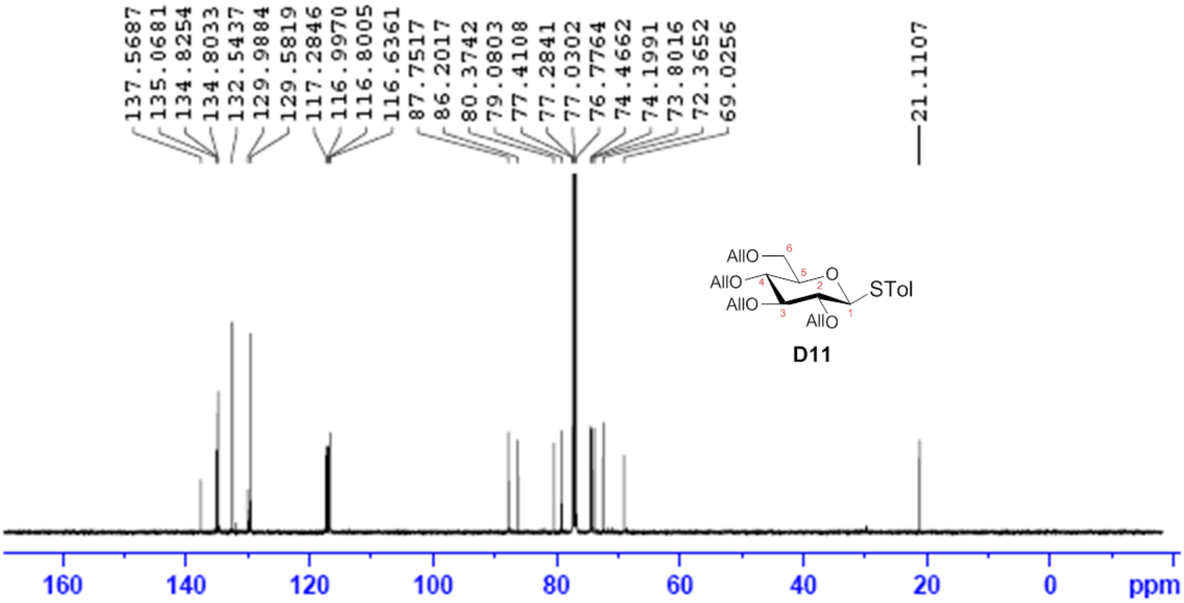

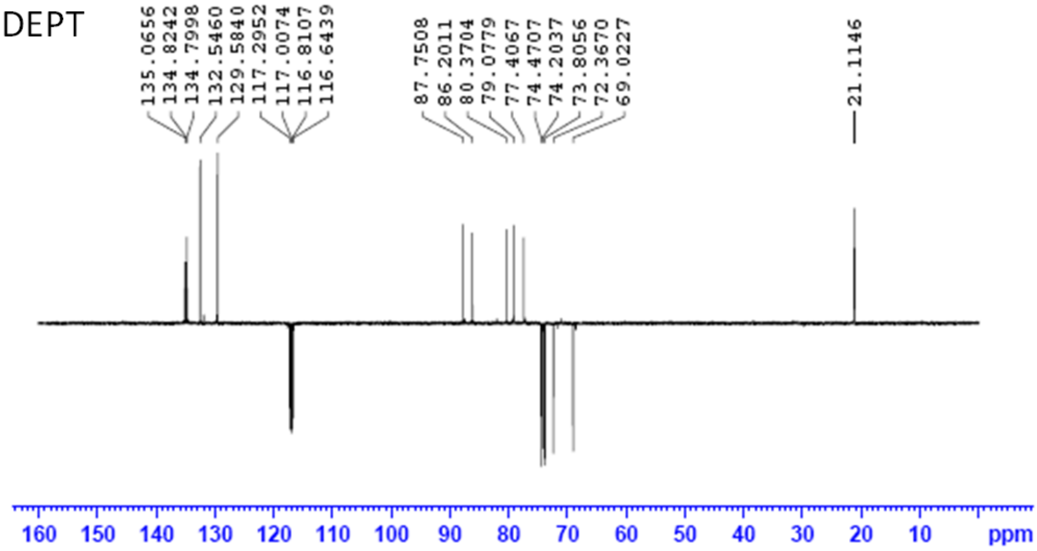

HMBC NMR of D11 in $CDCl_3$

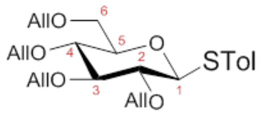

D11

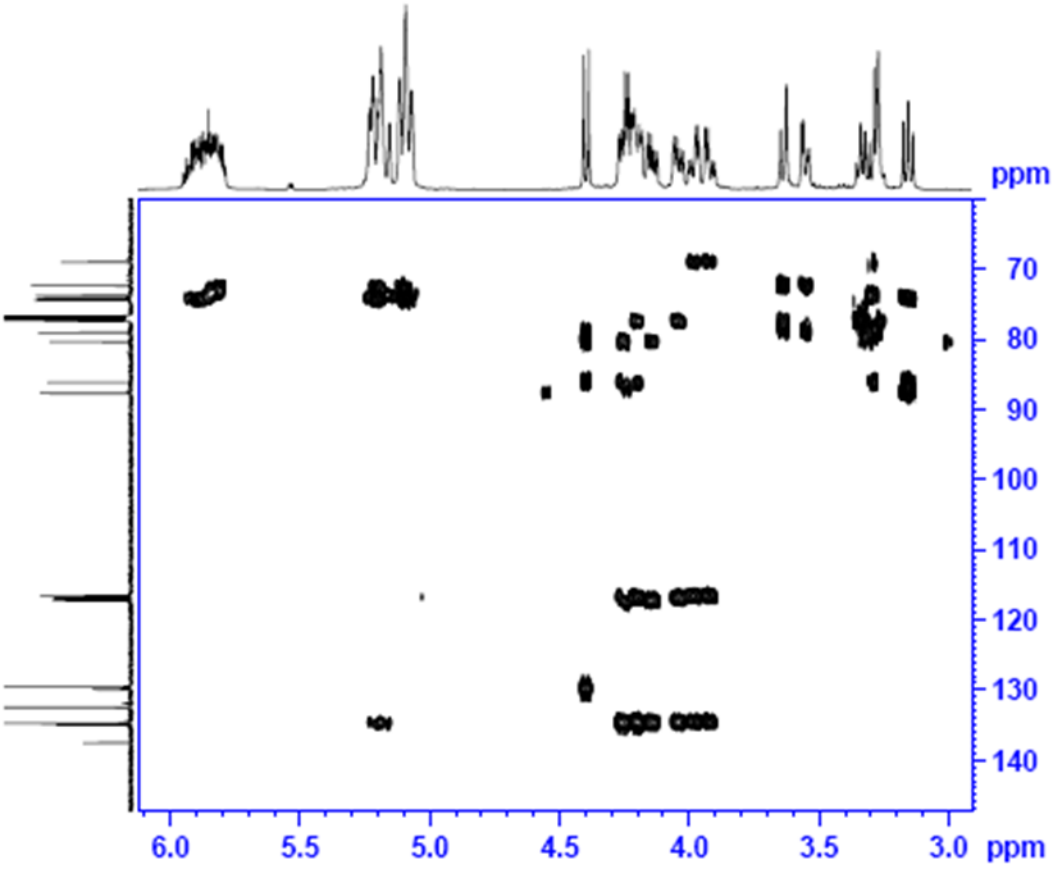

HMQC NMR of D11 in $CDCl_3$

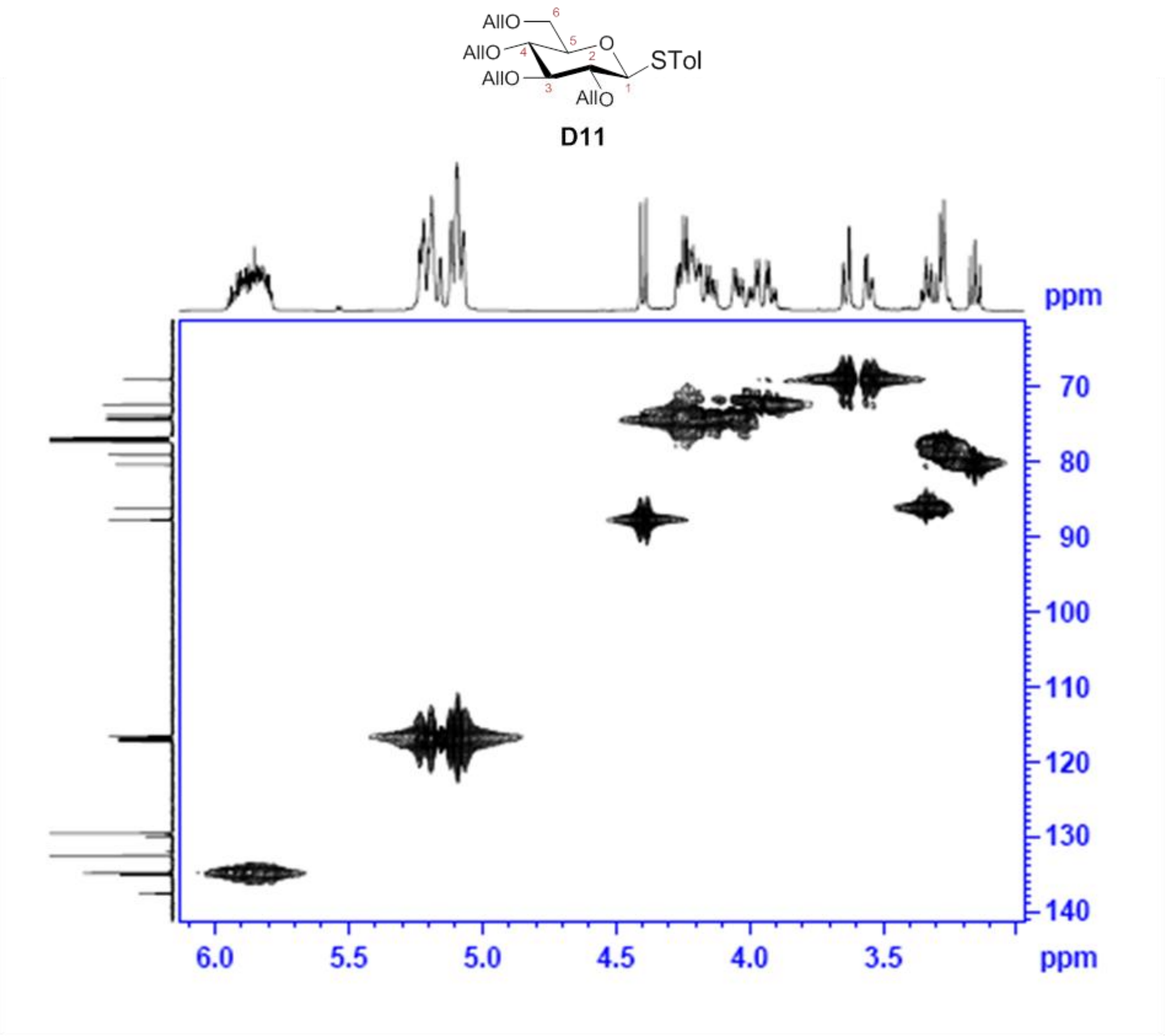

^{1}H NMR of 2 in $CDCl_3$

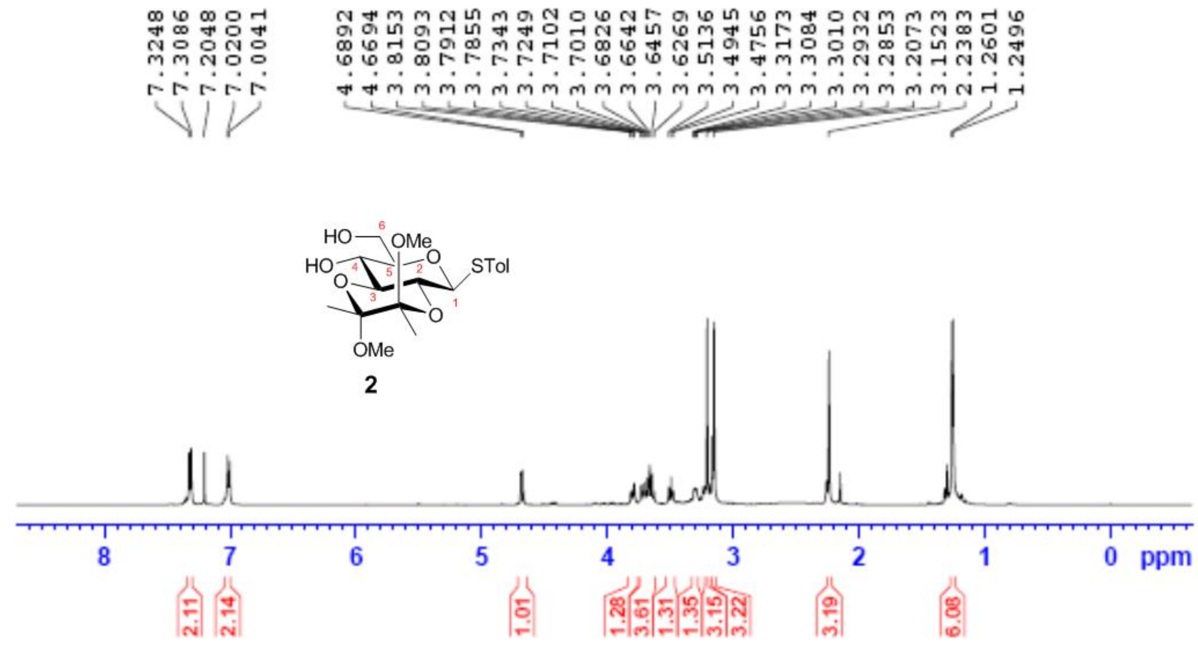

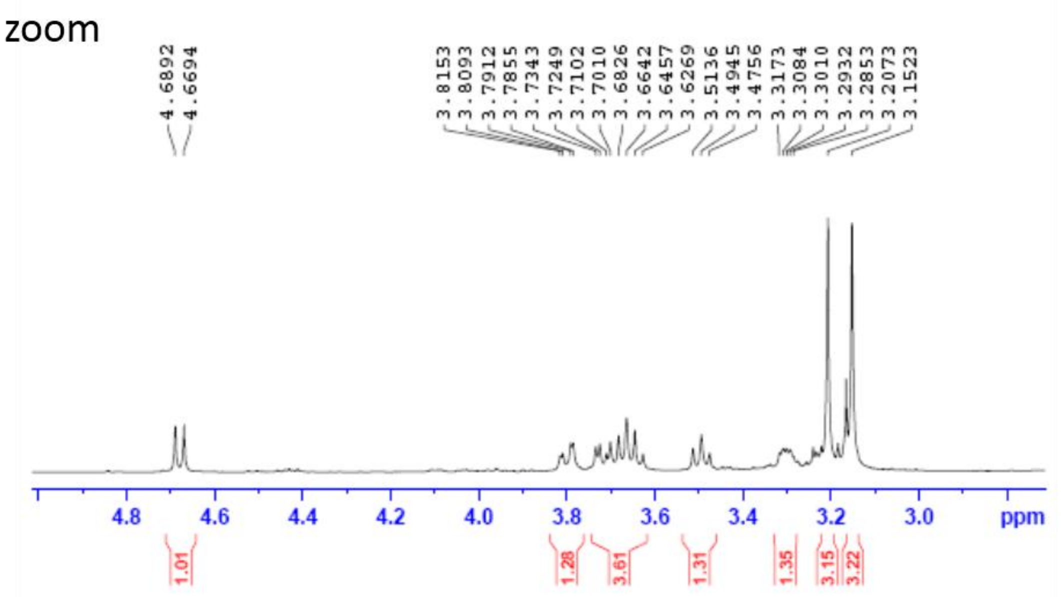

COSY NMR of 2 in $CDCl_3$

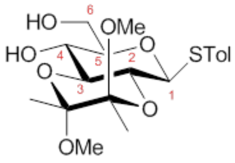

2

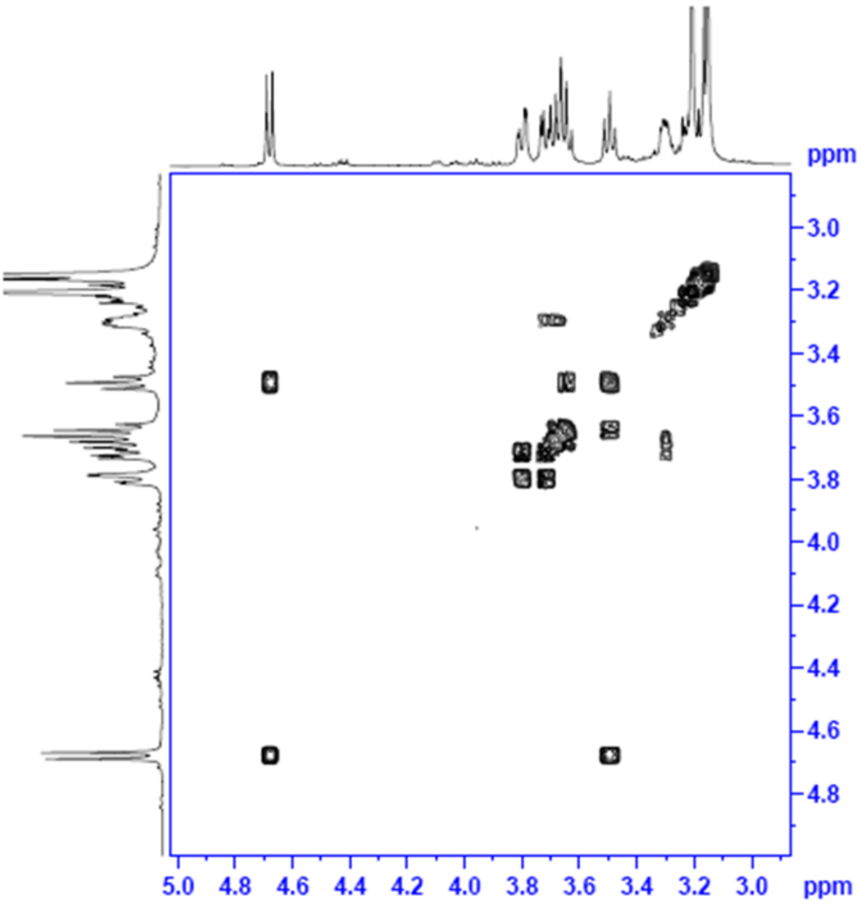

^{13}C NMR of 2 in $CDCl_3$

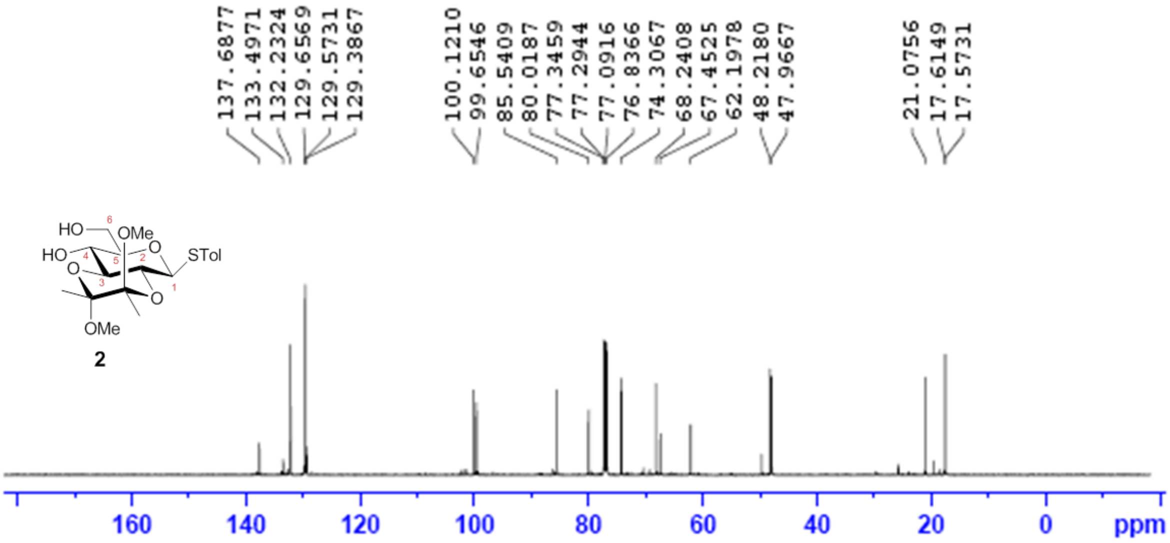

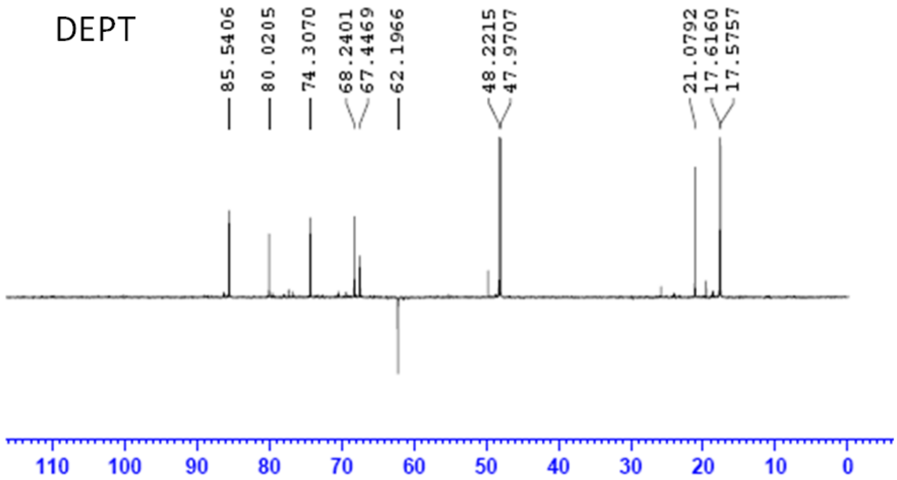

HMBC NMR of 2 in $CDCl_3$

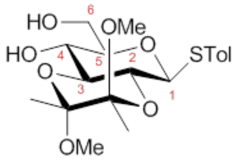

2

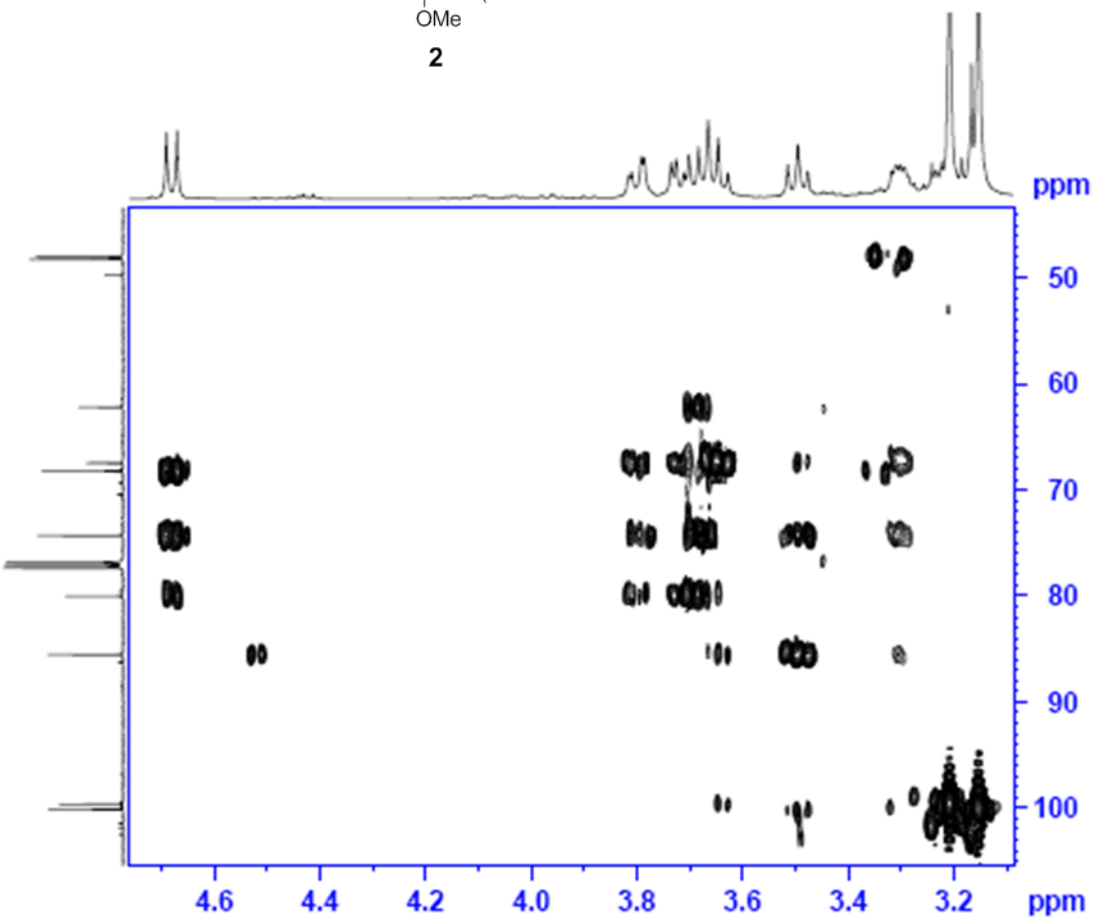

HMQC NMR of 2 in $CDCl_3$

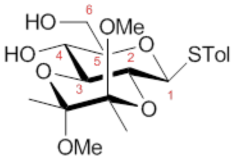

2

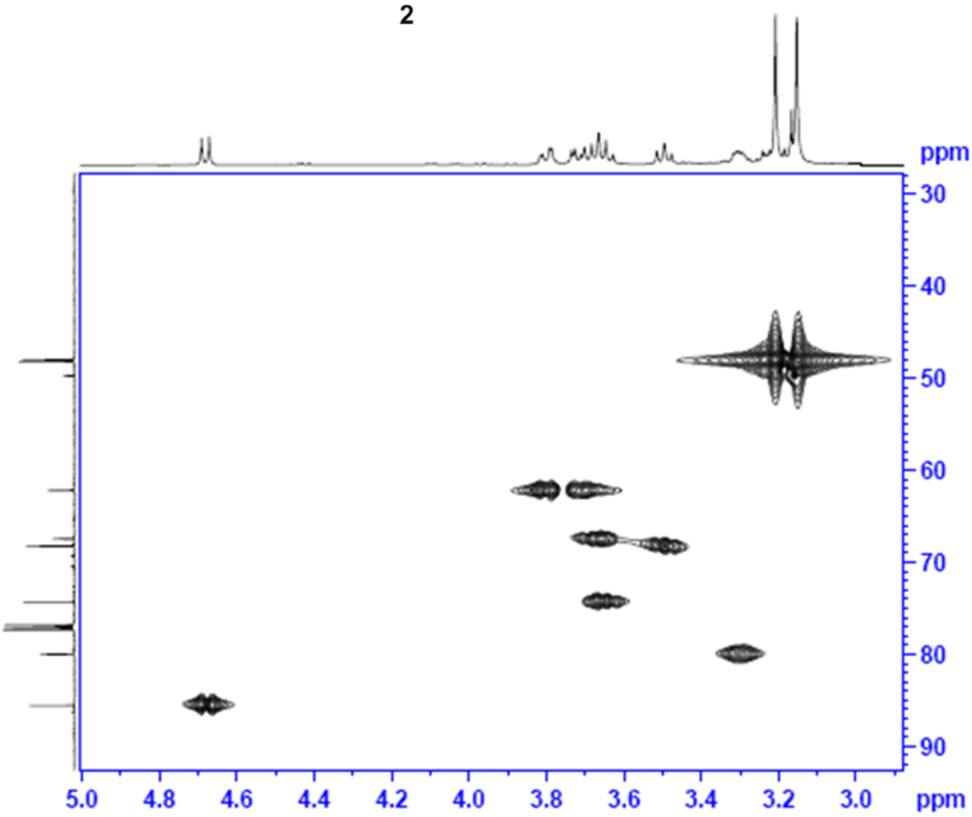

^{1}H NMR of 3α in CDCl$_3$

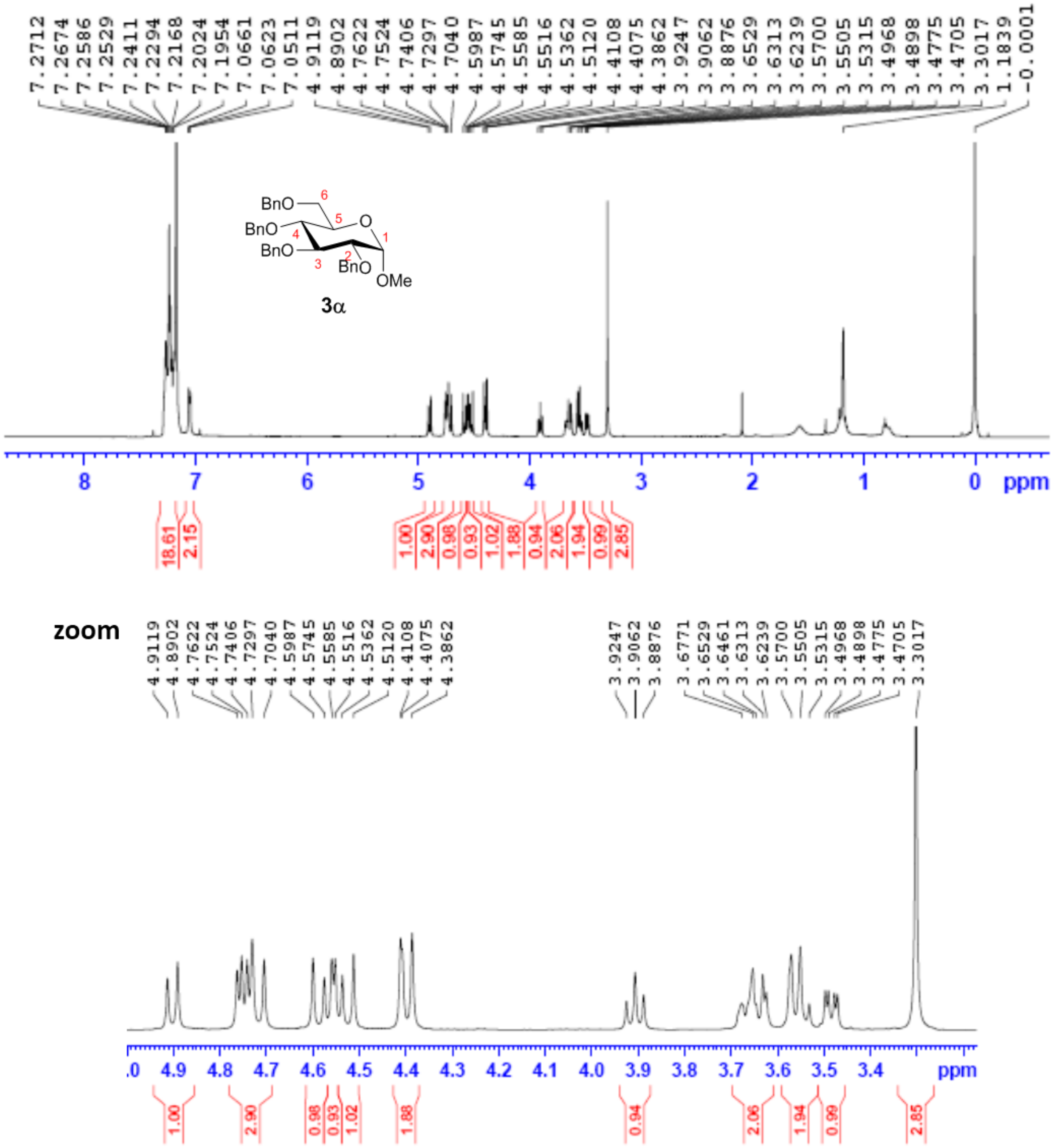

COSY of 3α in $CDCl_3$

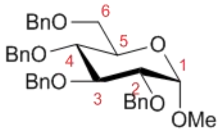

3α

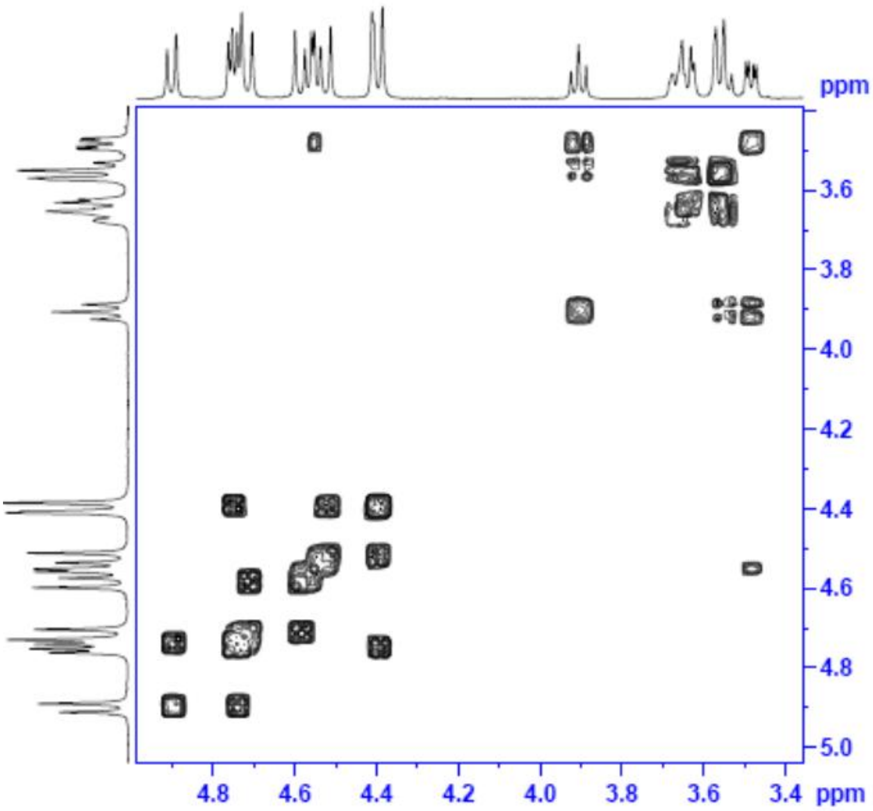

^{1}H NMR of 3β in $CDCl_3$

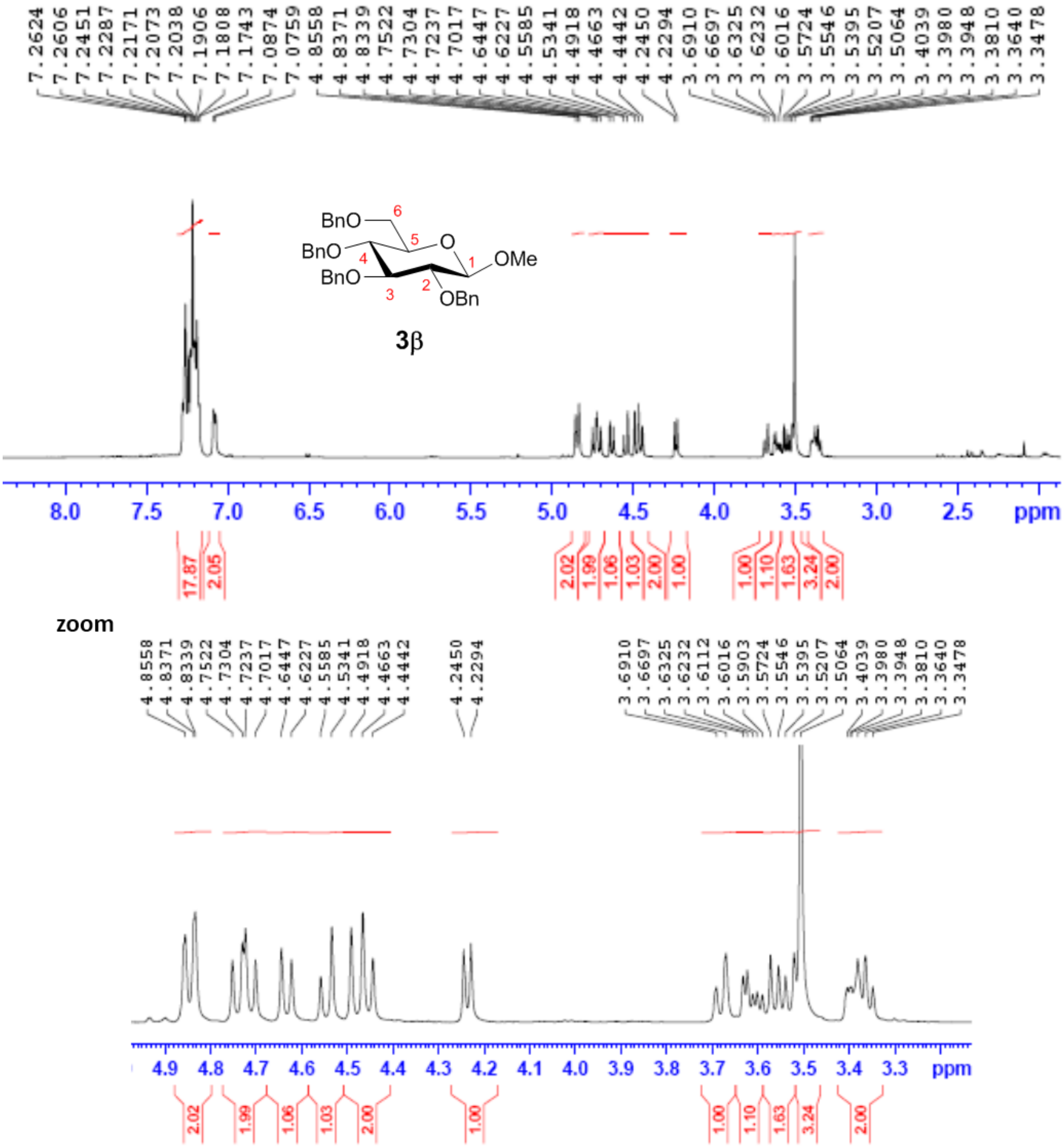

COSY of 3β in $CDCl_3$

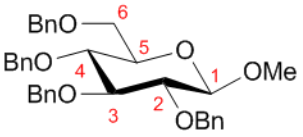

3β

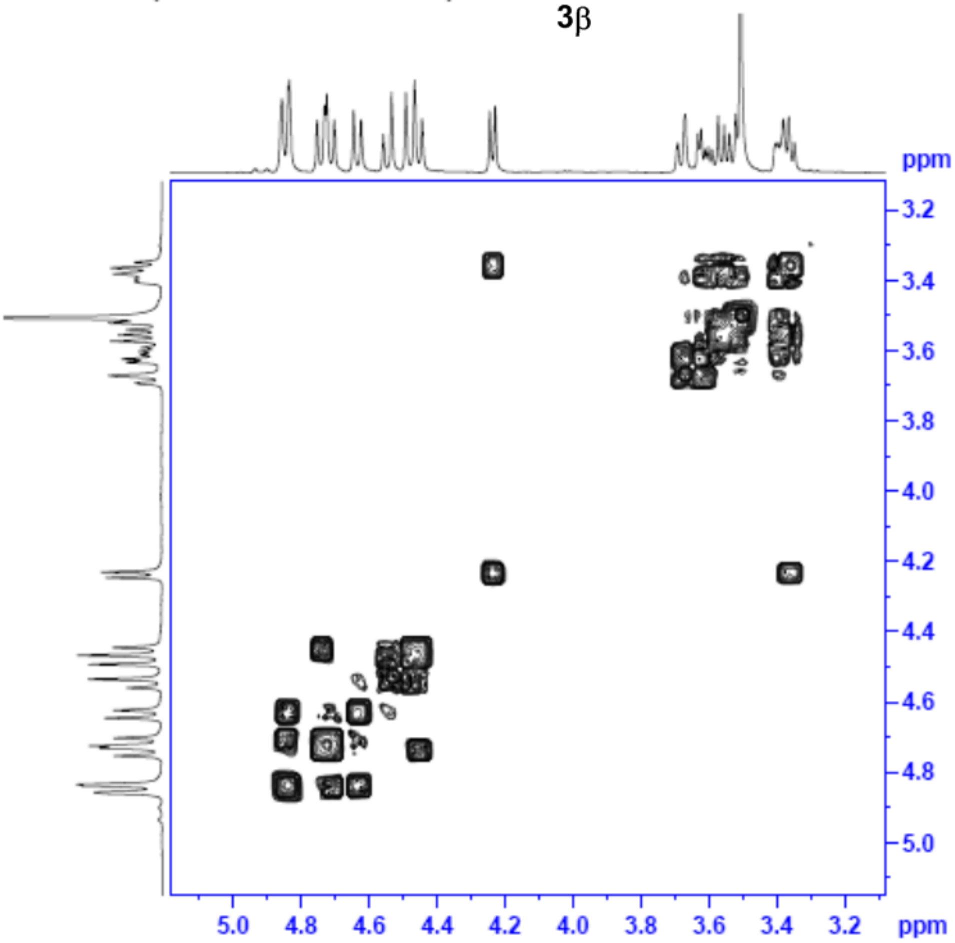

^1H NMR of 4αβ in $CDCl_3$

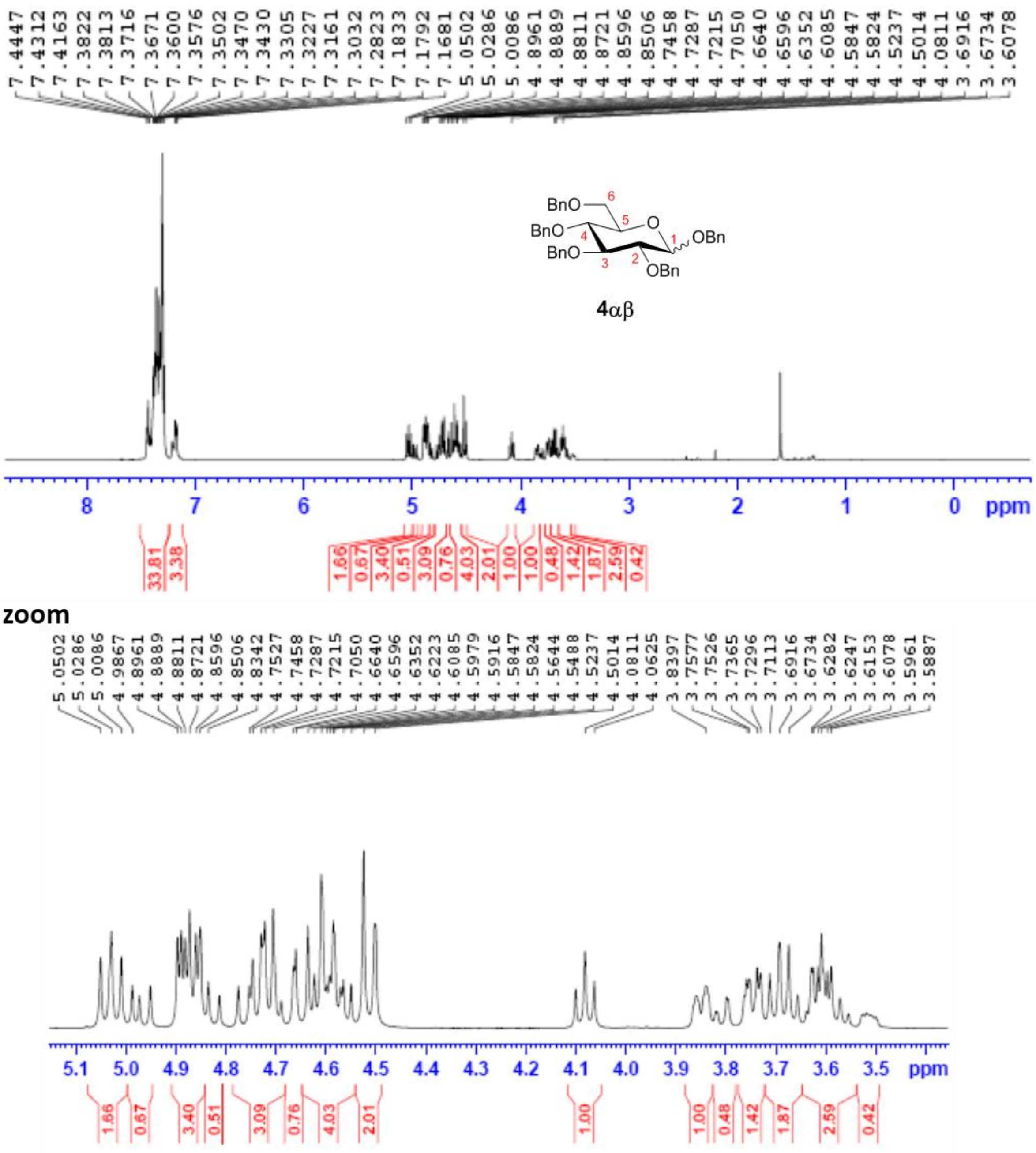

COSY of 4αβ in $CDCl_3$

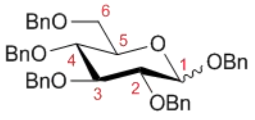

4αβ

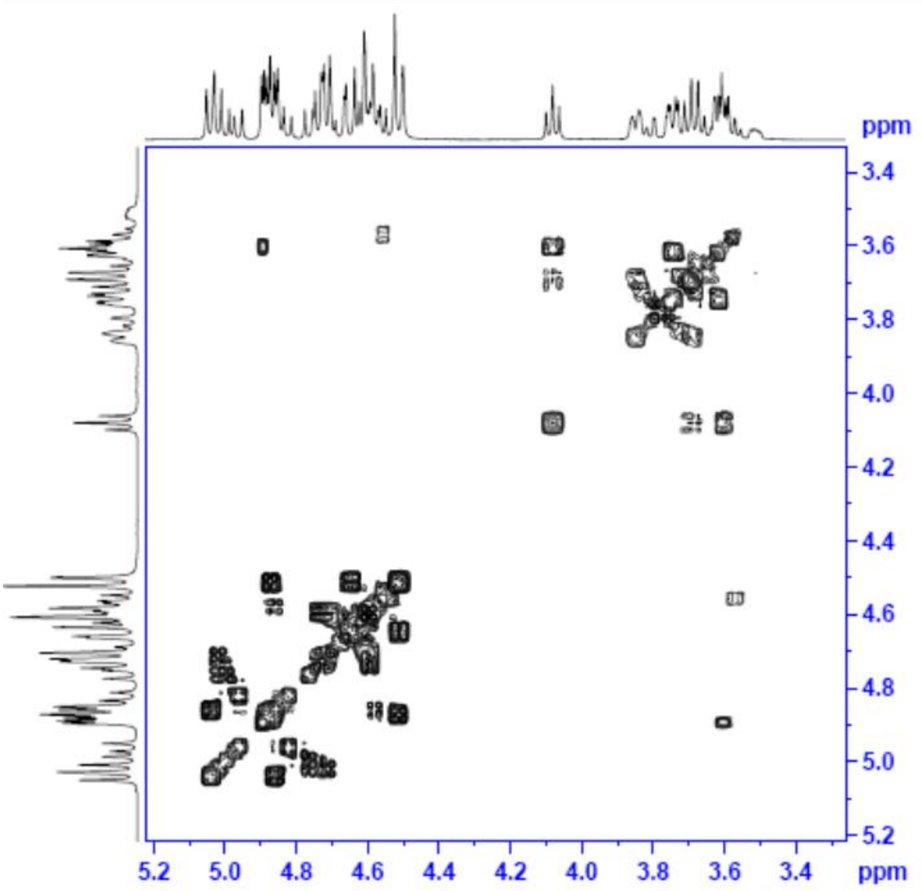

^{1}H NMR of 5αβ in CDCl$_3$

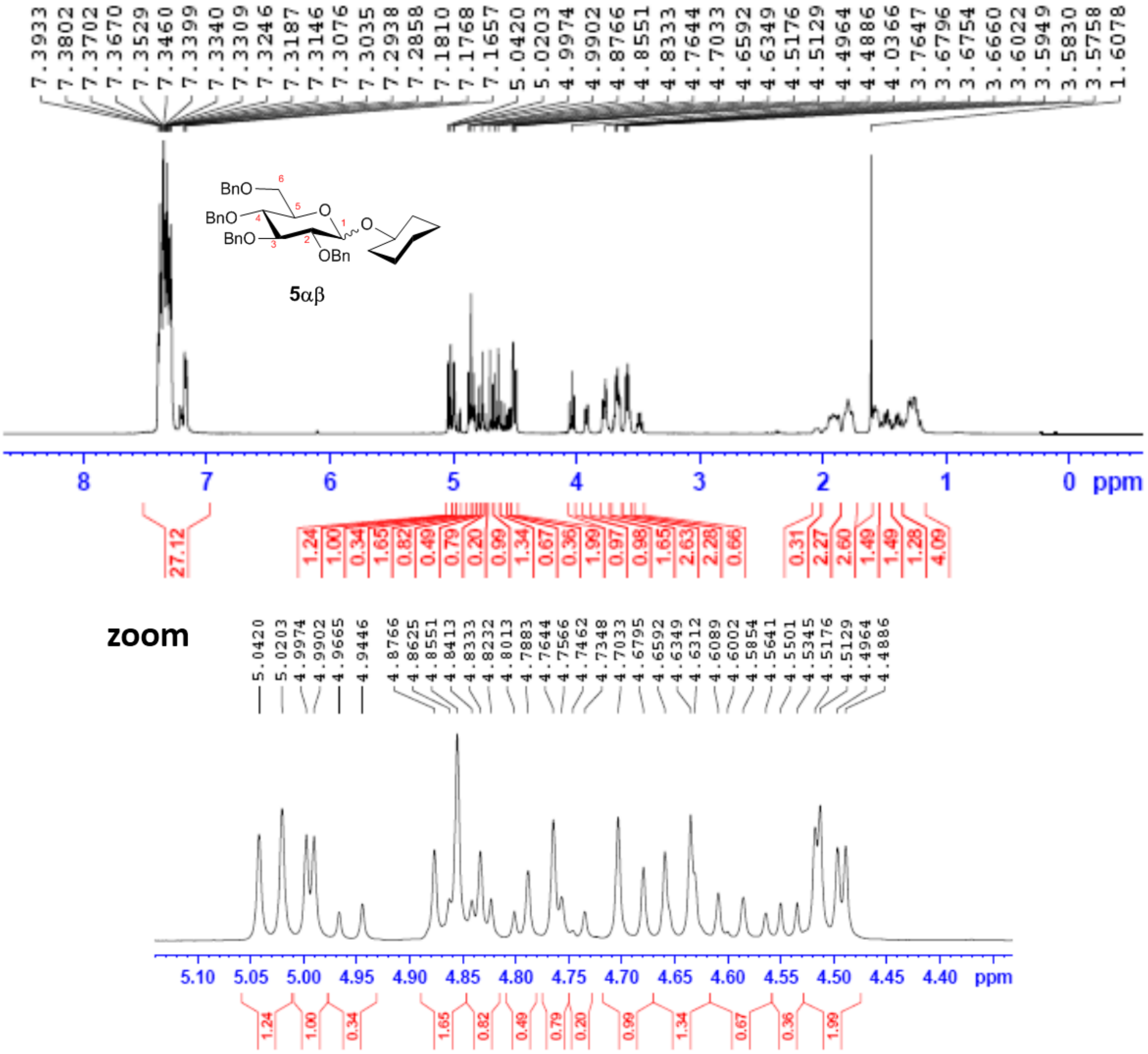

COSY of 5αβ in $CDCl_3$

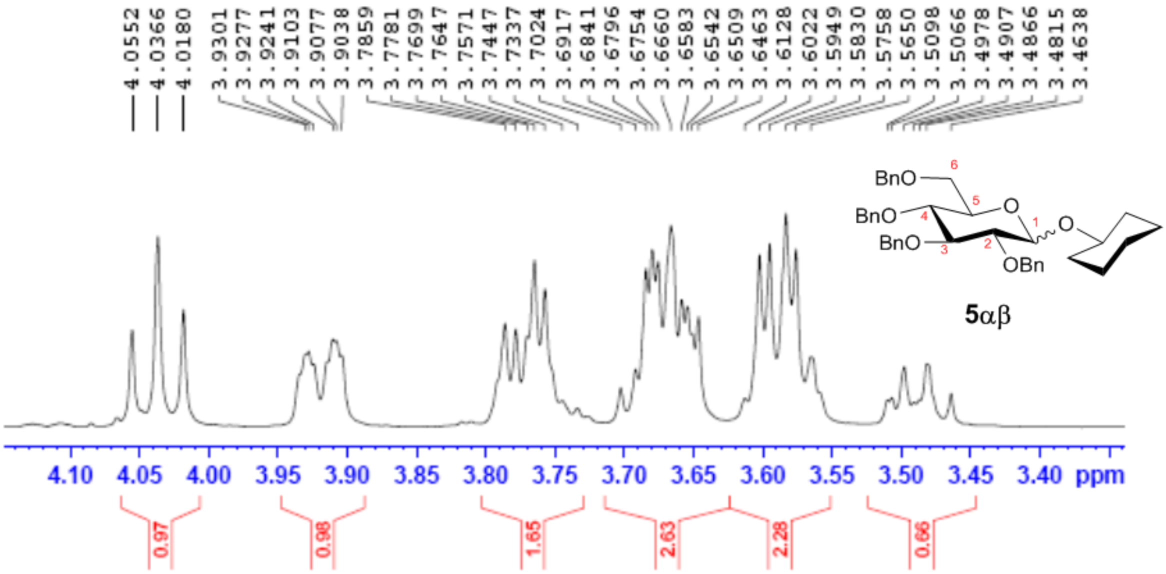

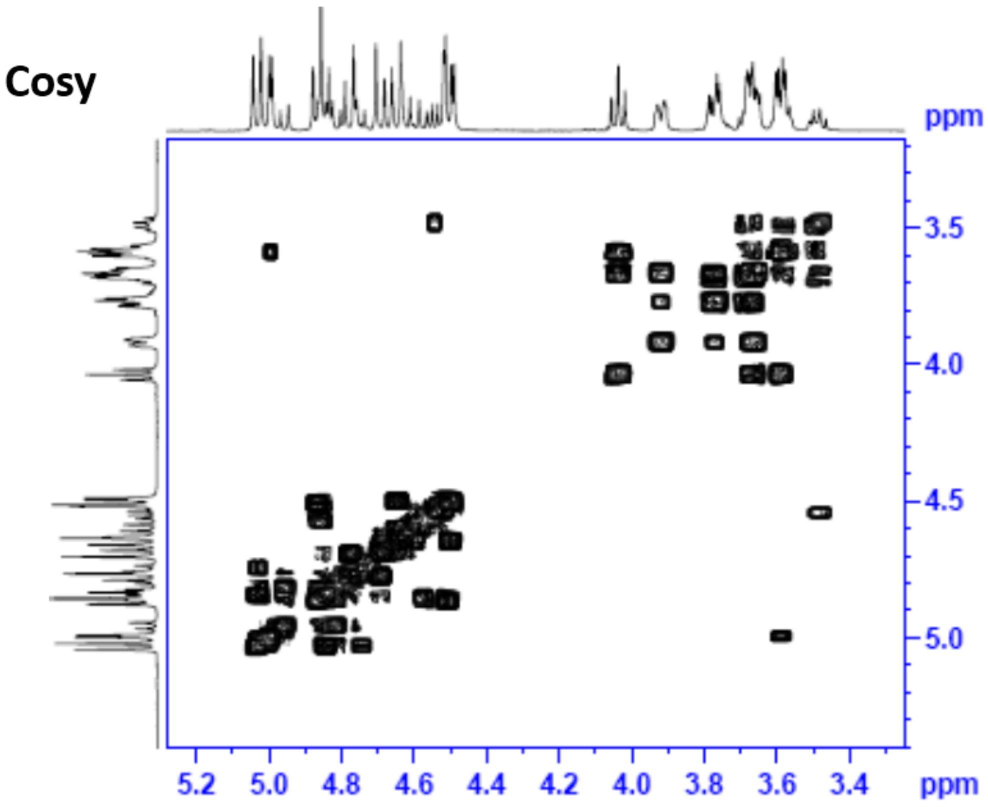

^{1}H NMR of 6α in $CDCl_3$

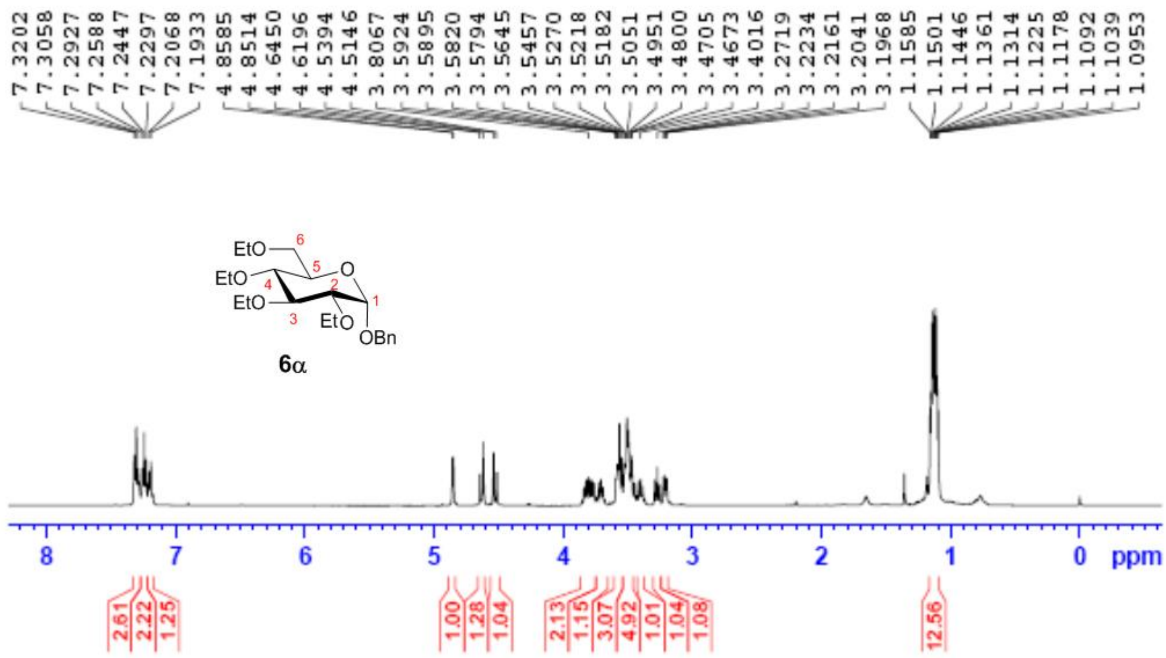

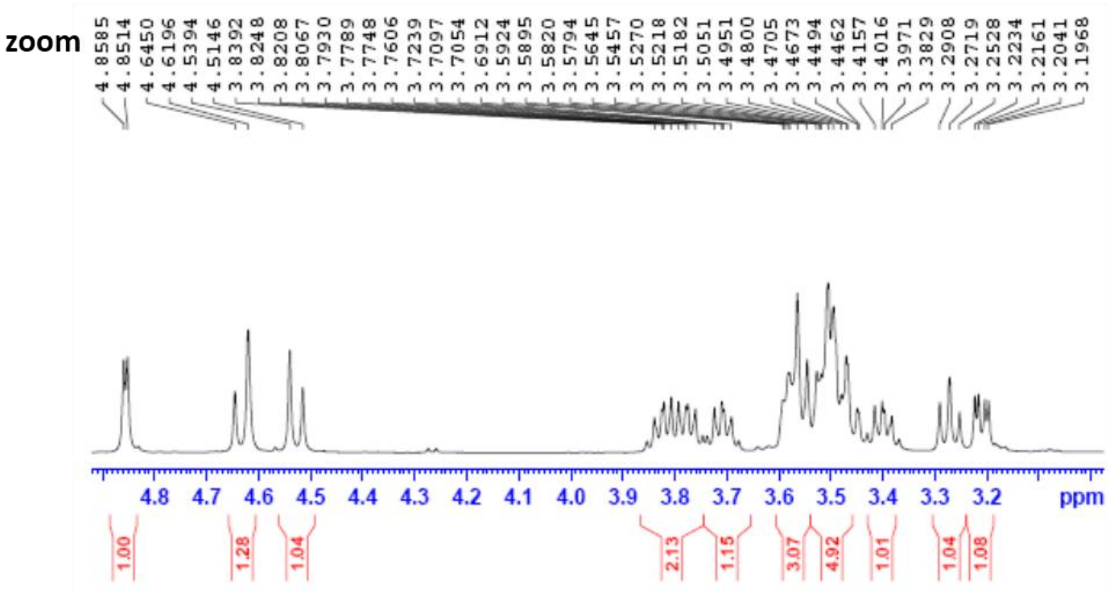

COSY of 6α in $CDCl_3$

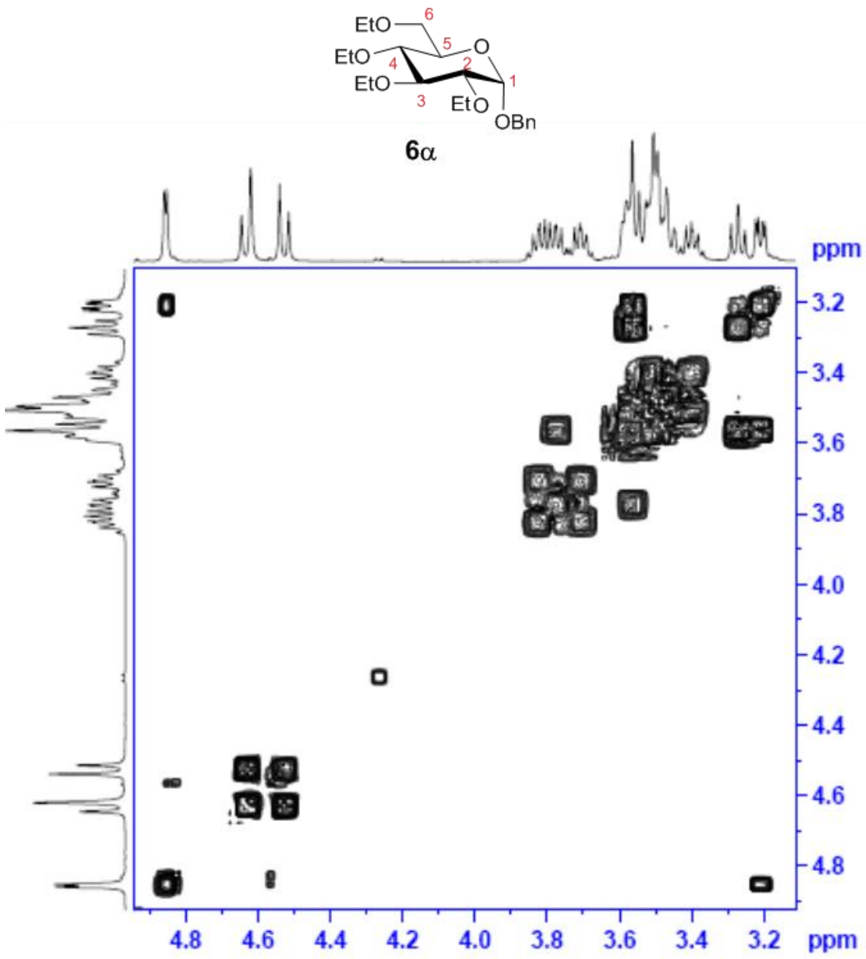

^{13}C NMR of 6α in $CDCl_3$

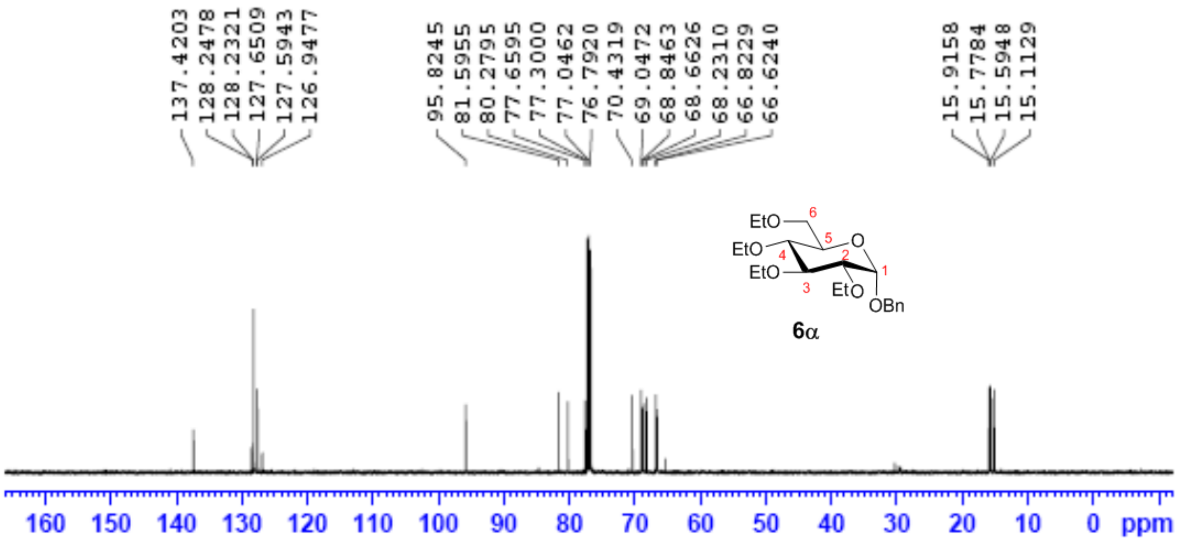

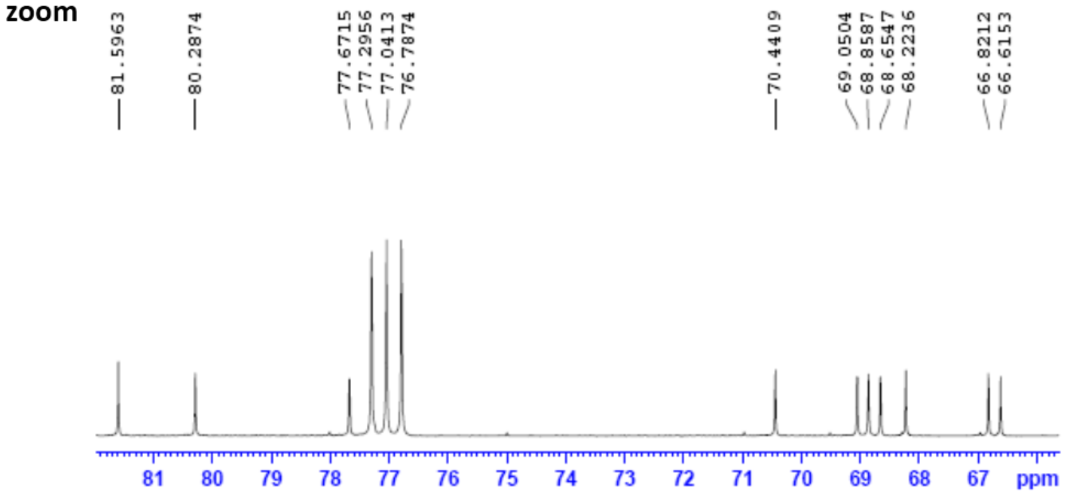

DEPT NMR of 6α in $CDCl_3$

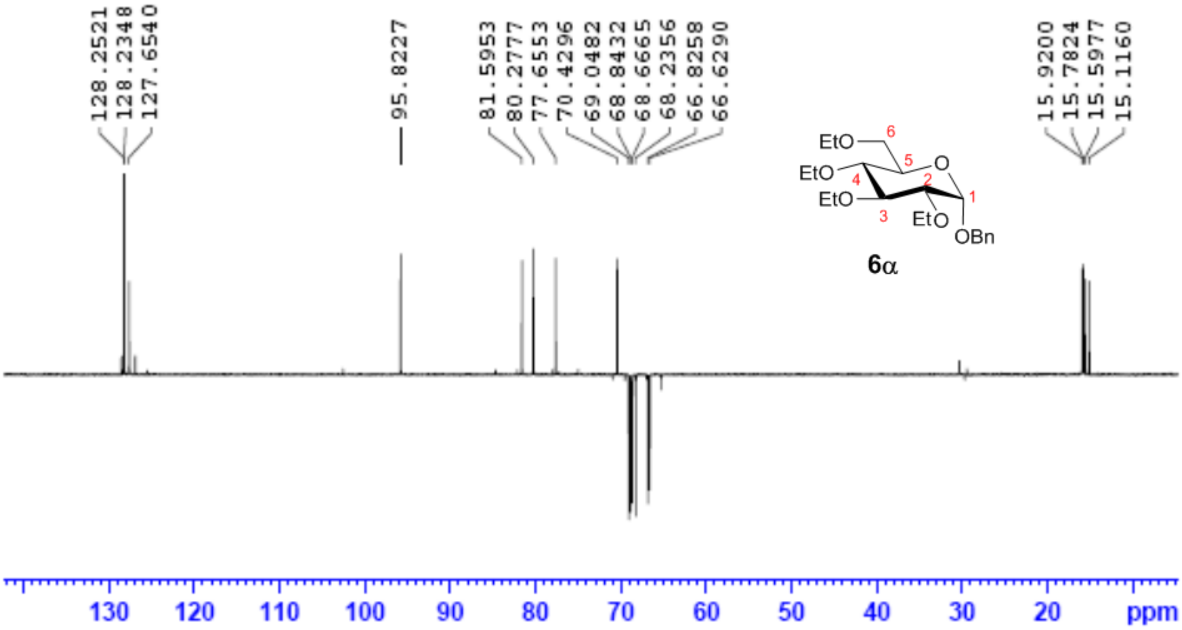

HMBC

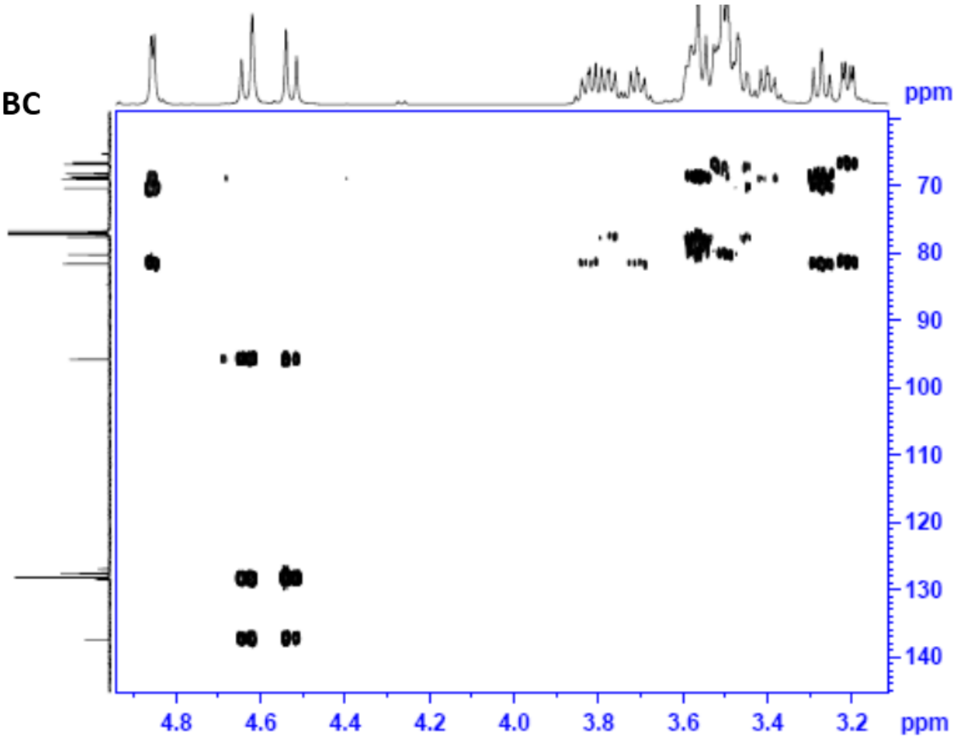

HMQC NMR of 6α in $CDCl_3$

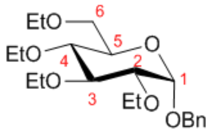

6α

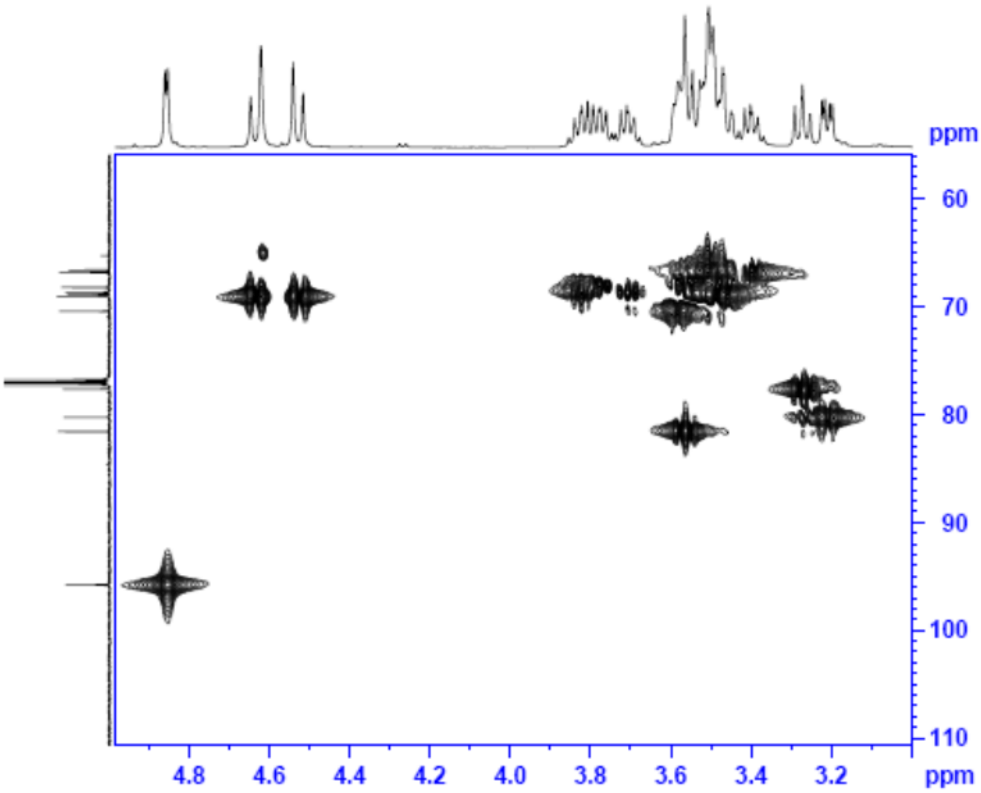

^{1}H NMR of 6β in $CDCl_3$

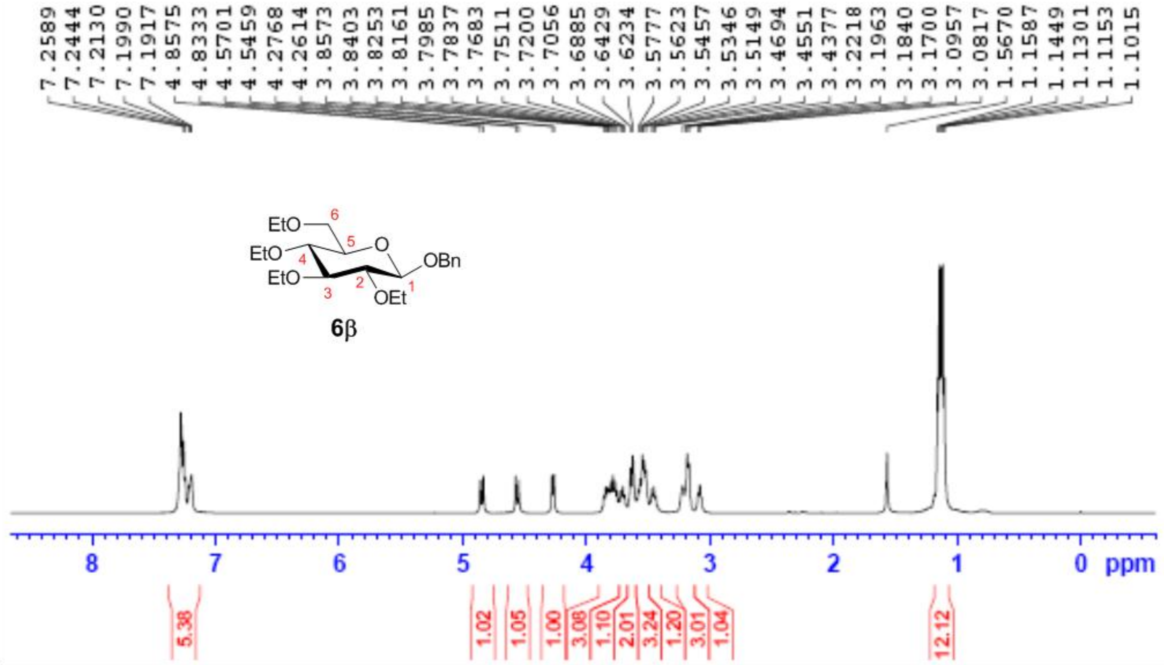

zoom

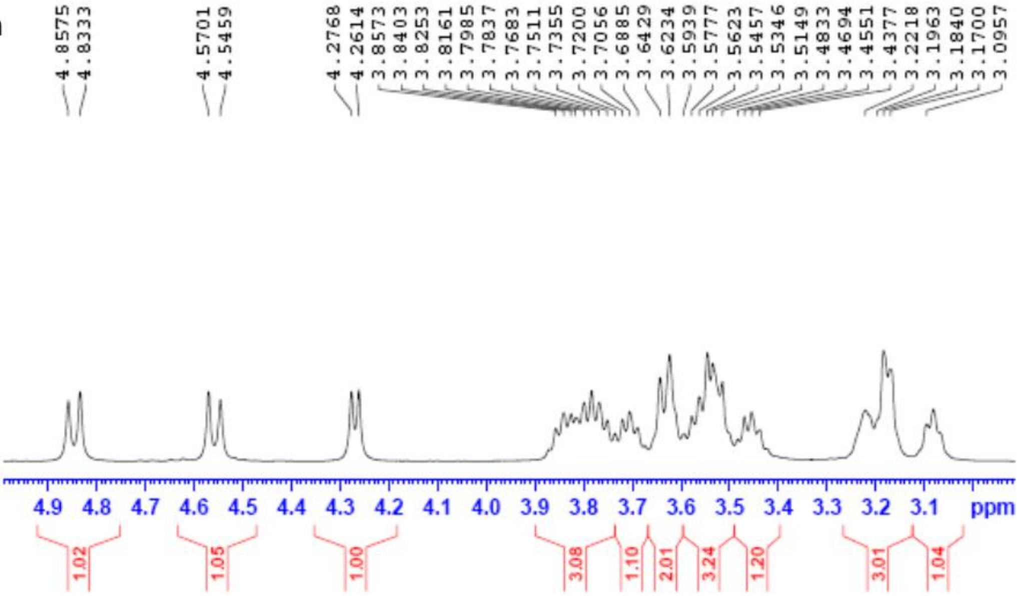

COSY NMR of 6β in $CDCl_3$

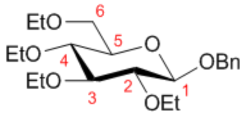

6β

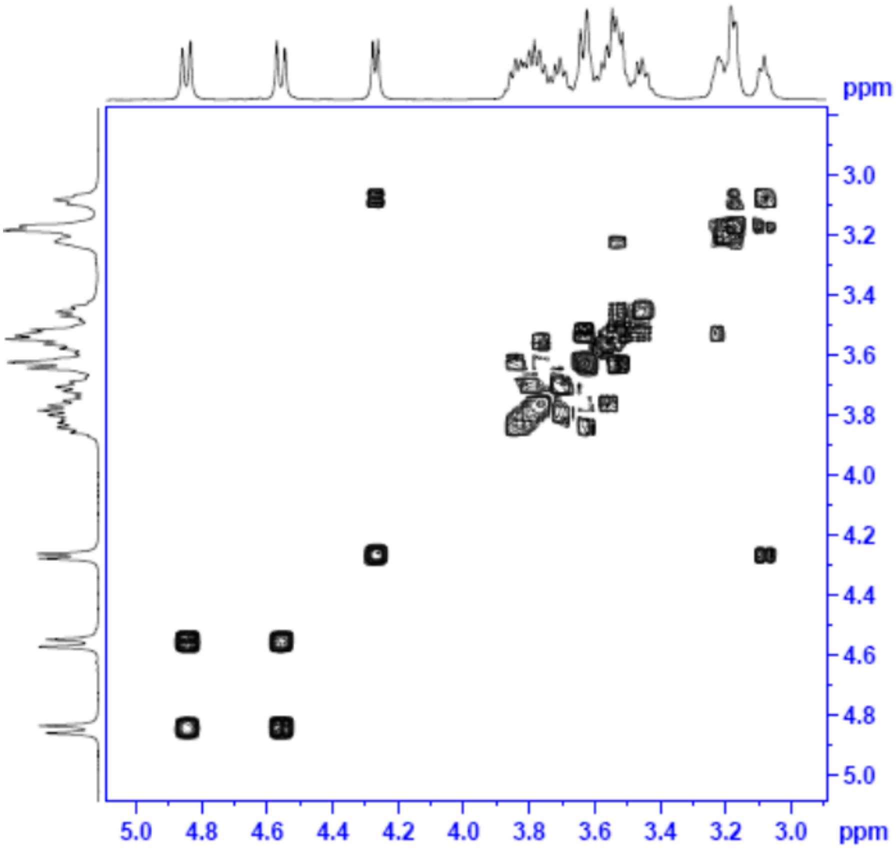

^{13}C NMR of 6β in $CDCl_3$

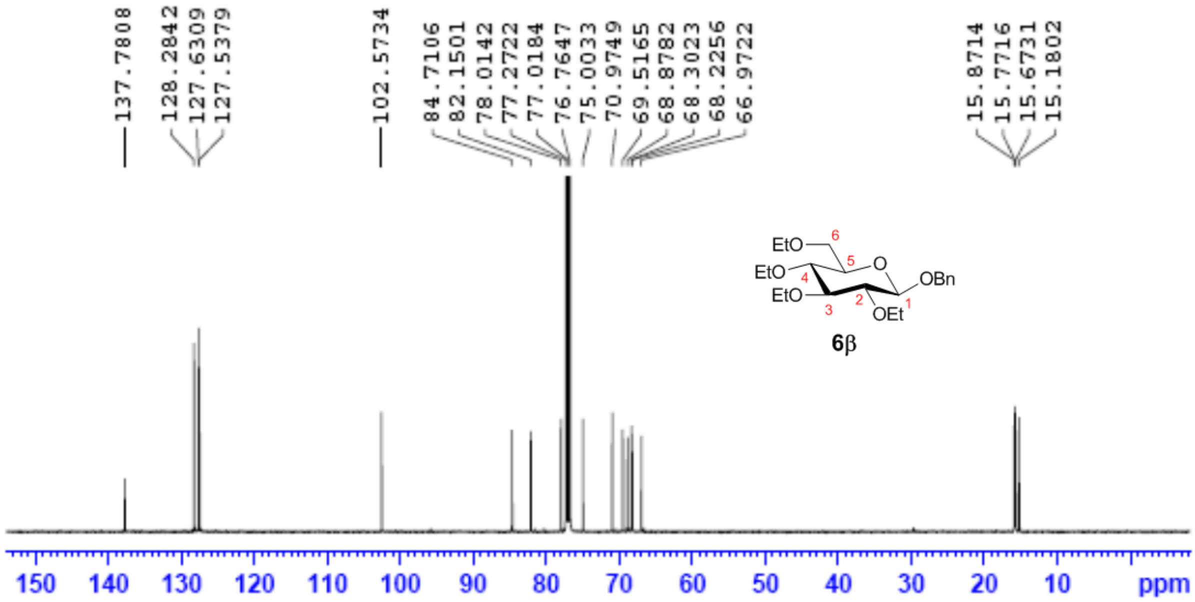

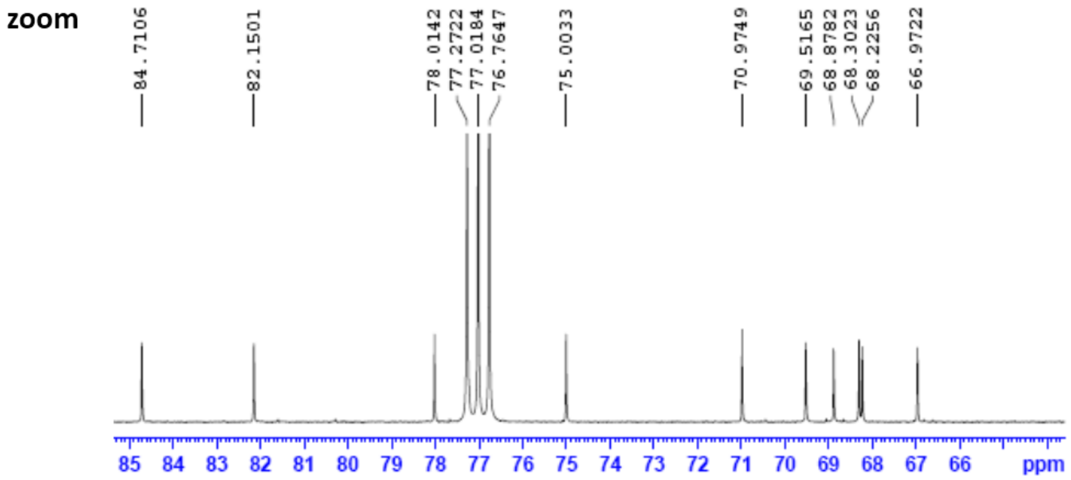

DEPT NMR of 6β in $CDCl_3$

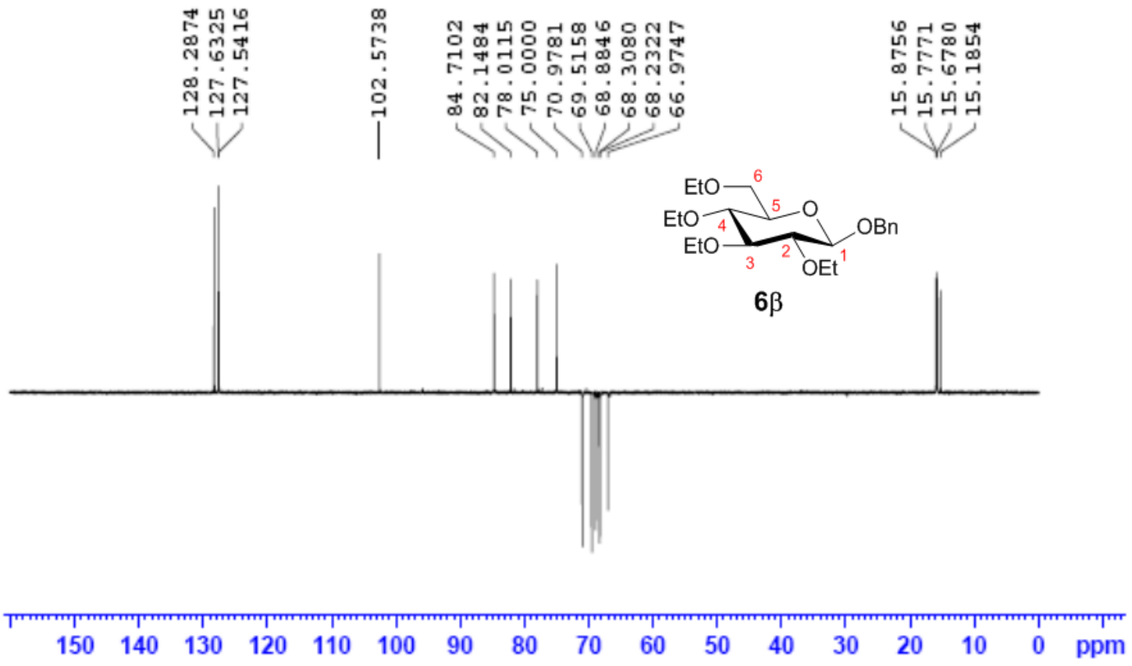

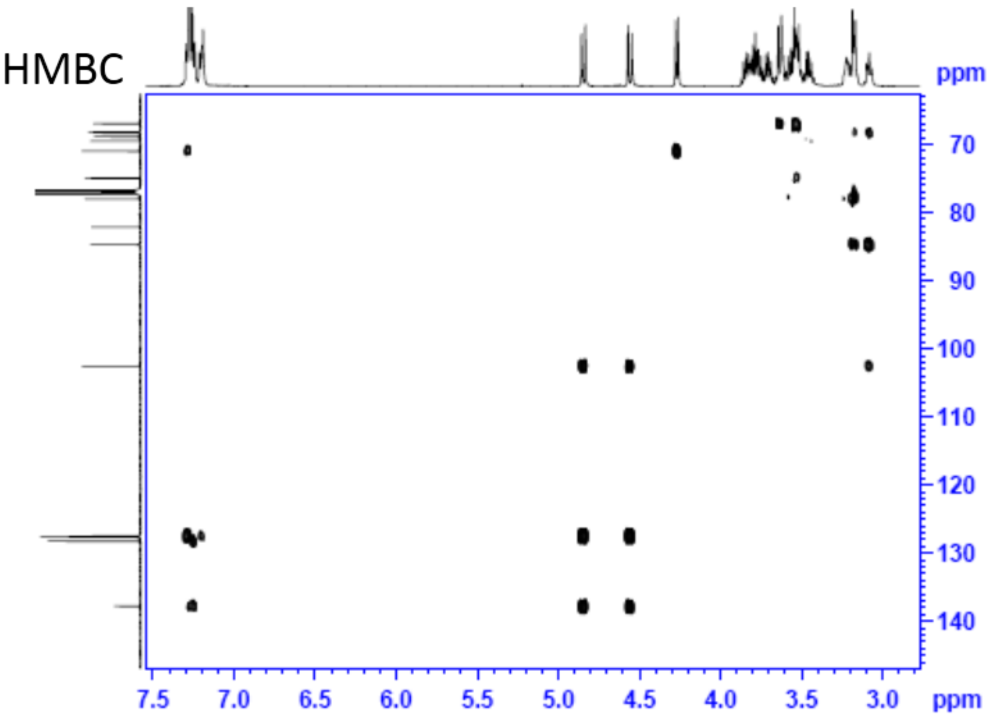

HMQC NMR of 6β in $CDCl_3$

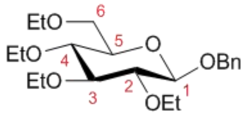

6β

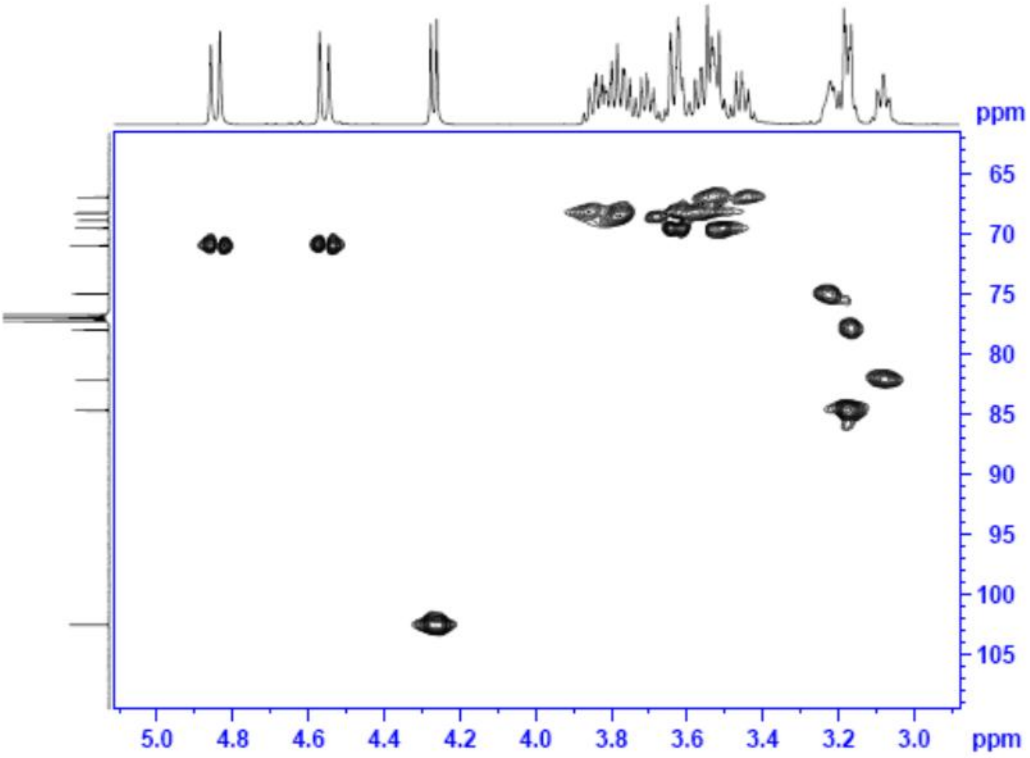

^{1}H NMR of 7α in $CDCl_3$

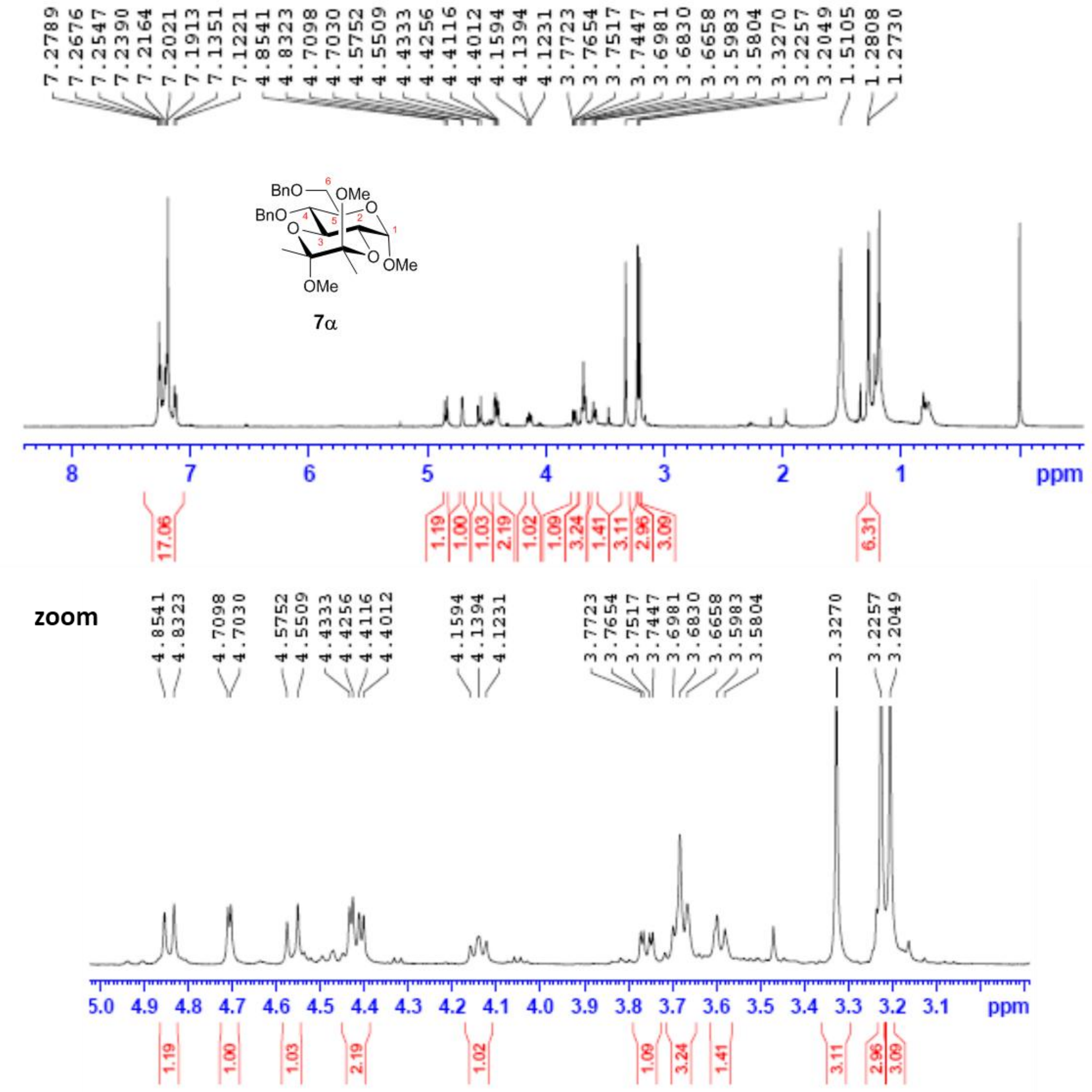

COSY NMR of 7α in $CDCl_3$

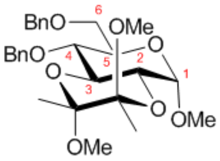

7α

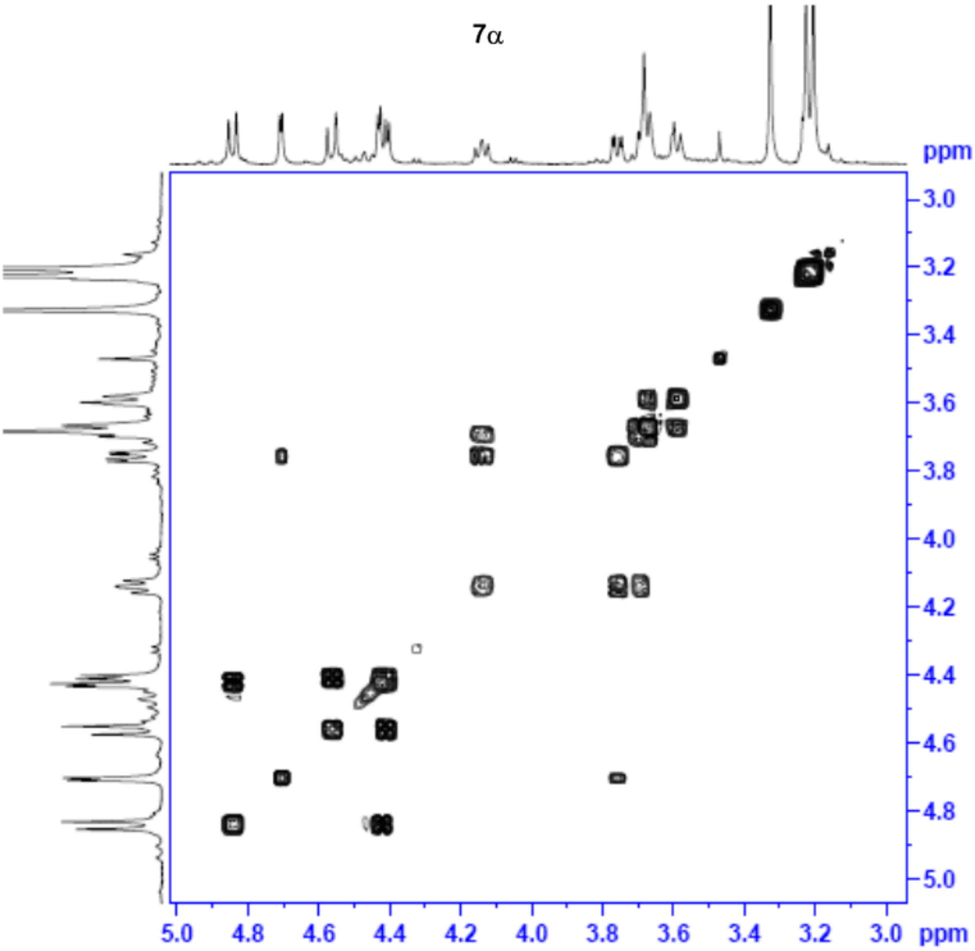

^{13}C NMR of 7α in $CDCl_3$

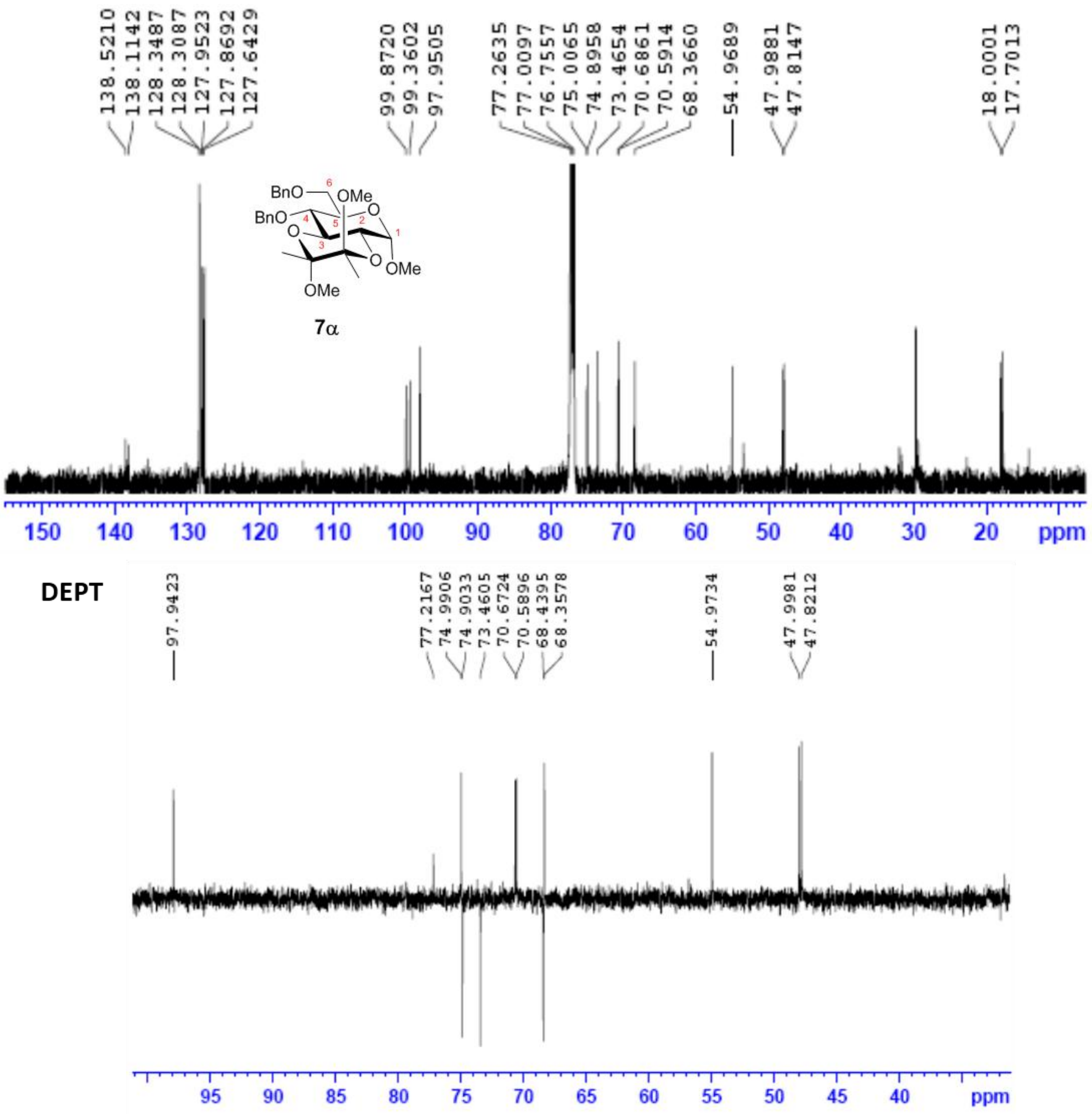

HMBC NMR of 7α in $CDCl_3$

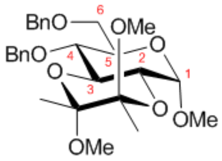

7α

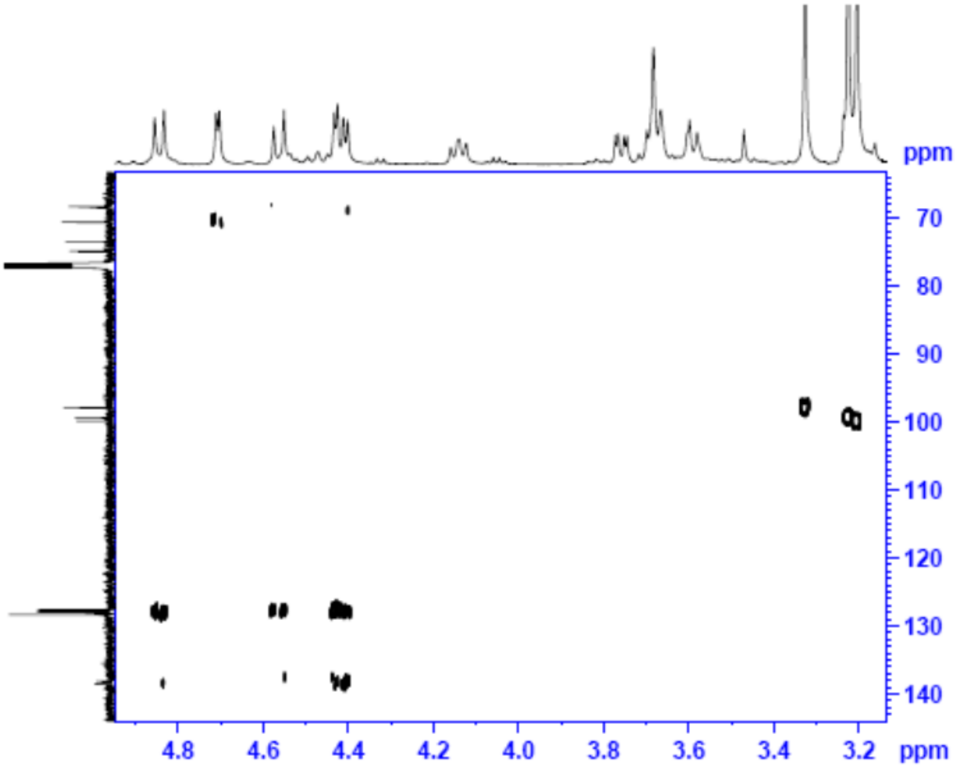

HMQC NMR of 7α in $CDCl_3$

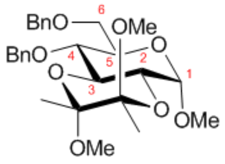

7α

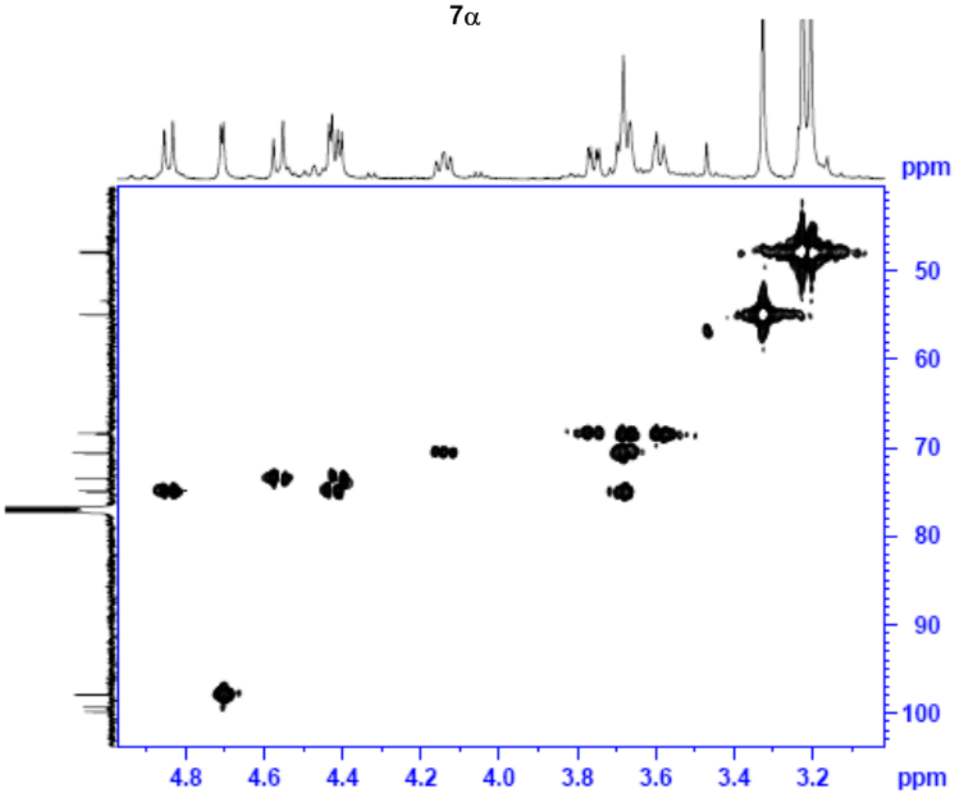

^{1}H NMR of 7β in $CDCl_3$

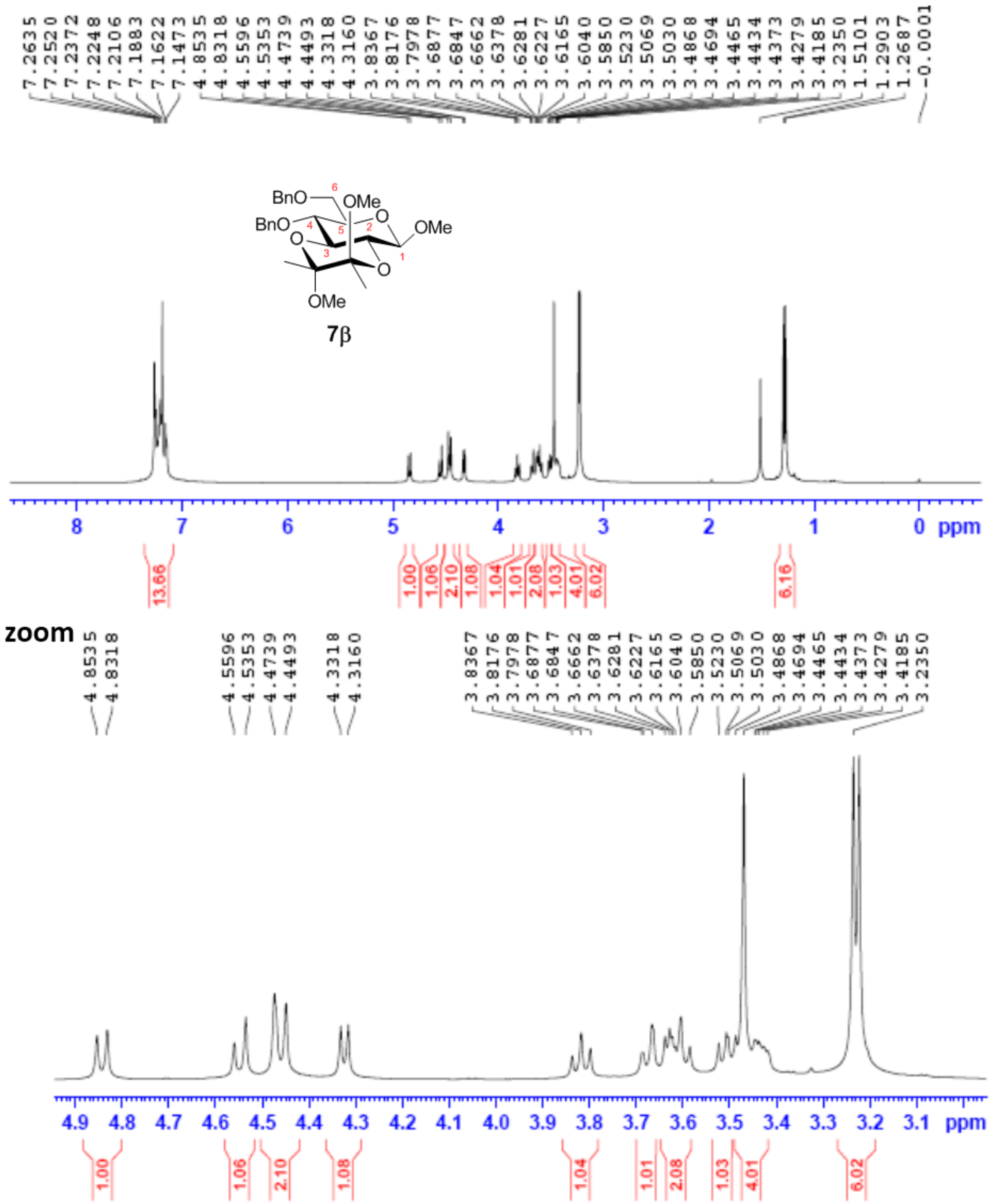

COSY NMR of 7β in $CDCl_3$

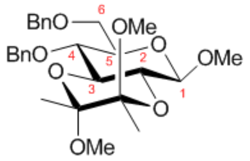

7β

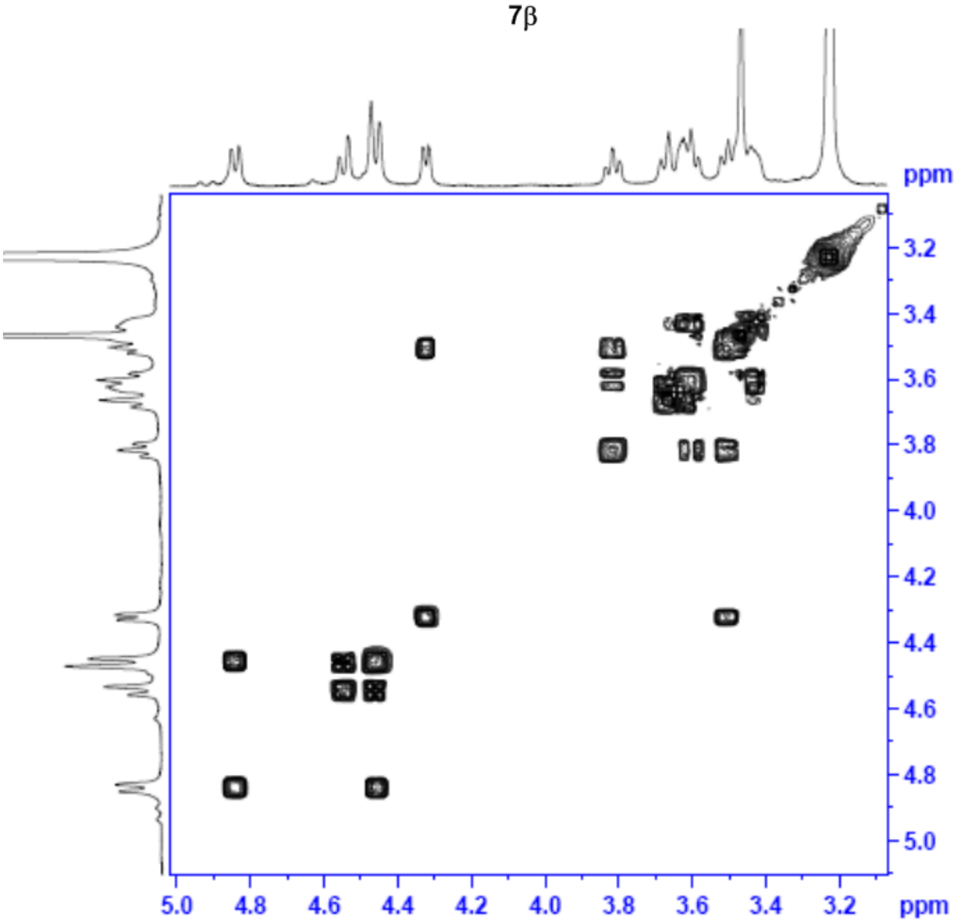

^{13}C NMR of 7β in $CDCl_3$

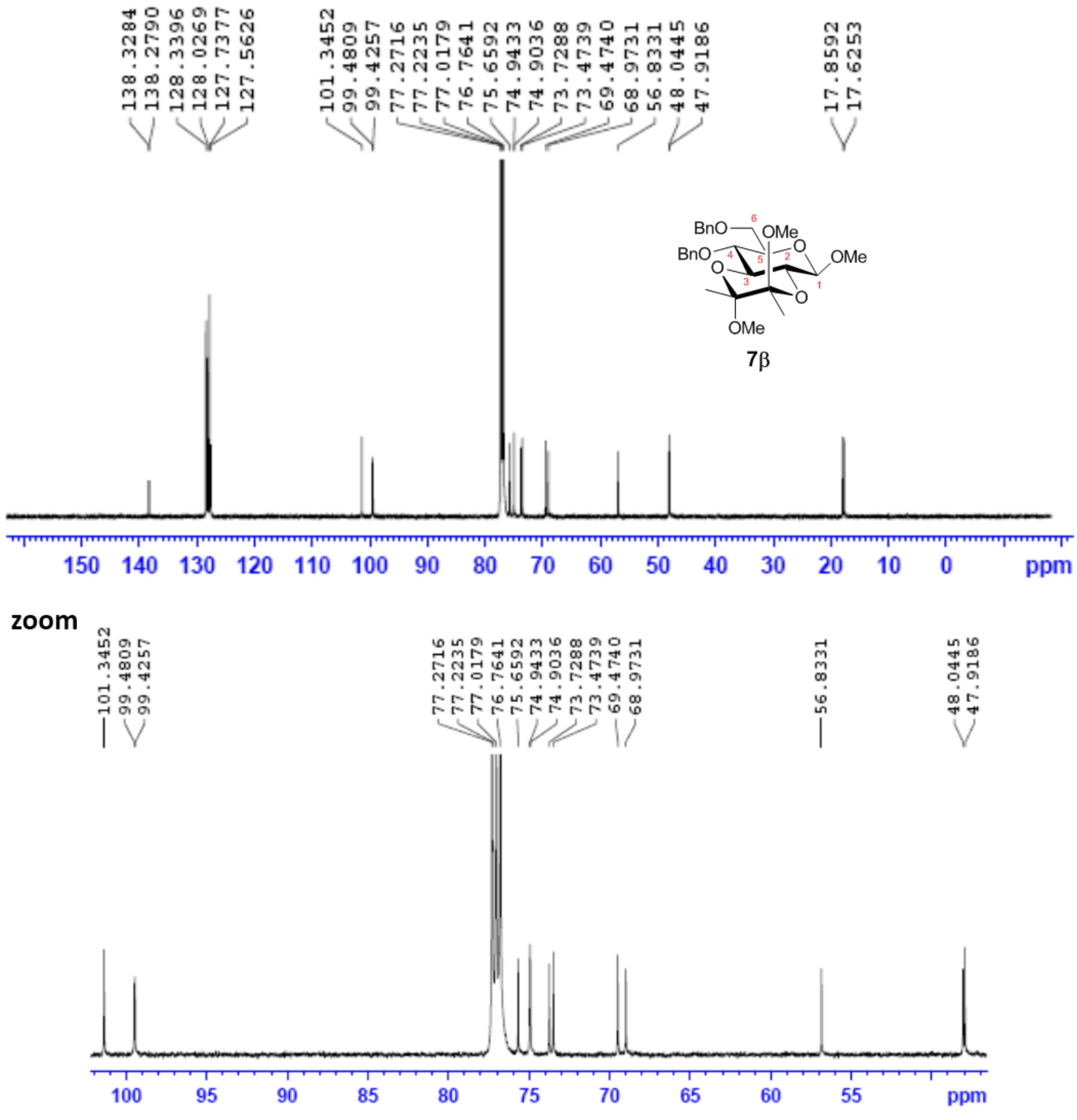

DEPT NMR of 7β in CDCl₃

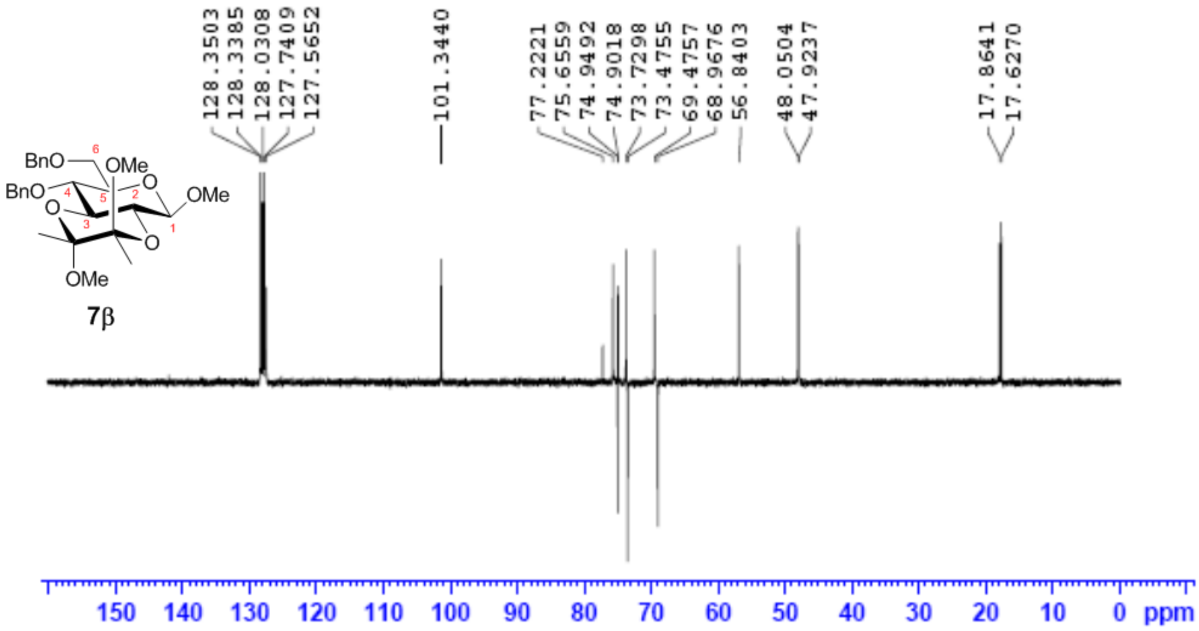

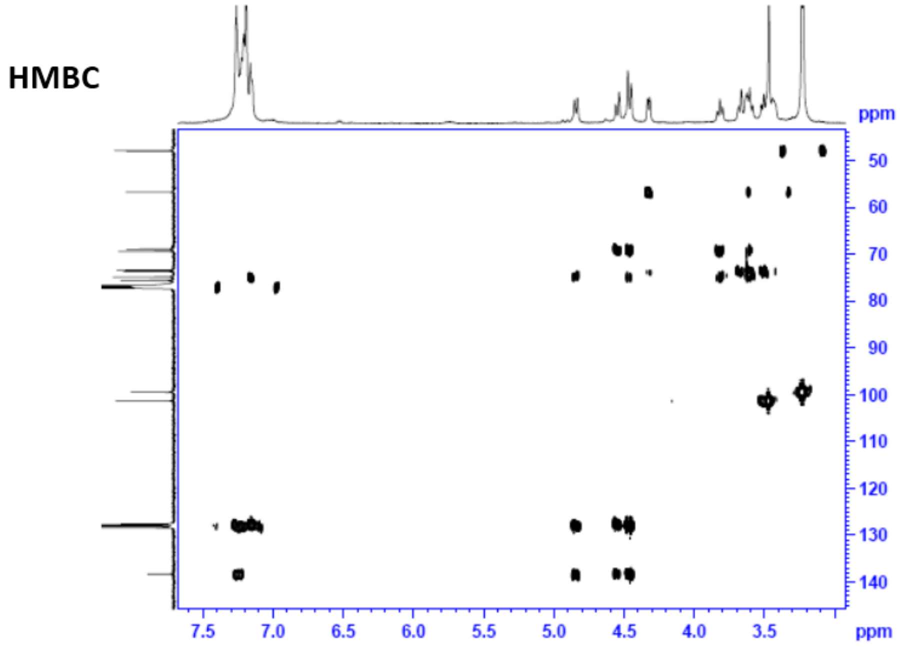

HMQC NMR of 7β in $CDCl_3$

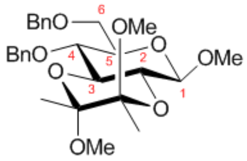

7β

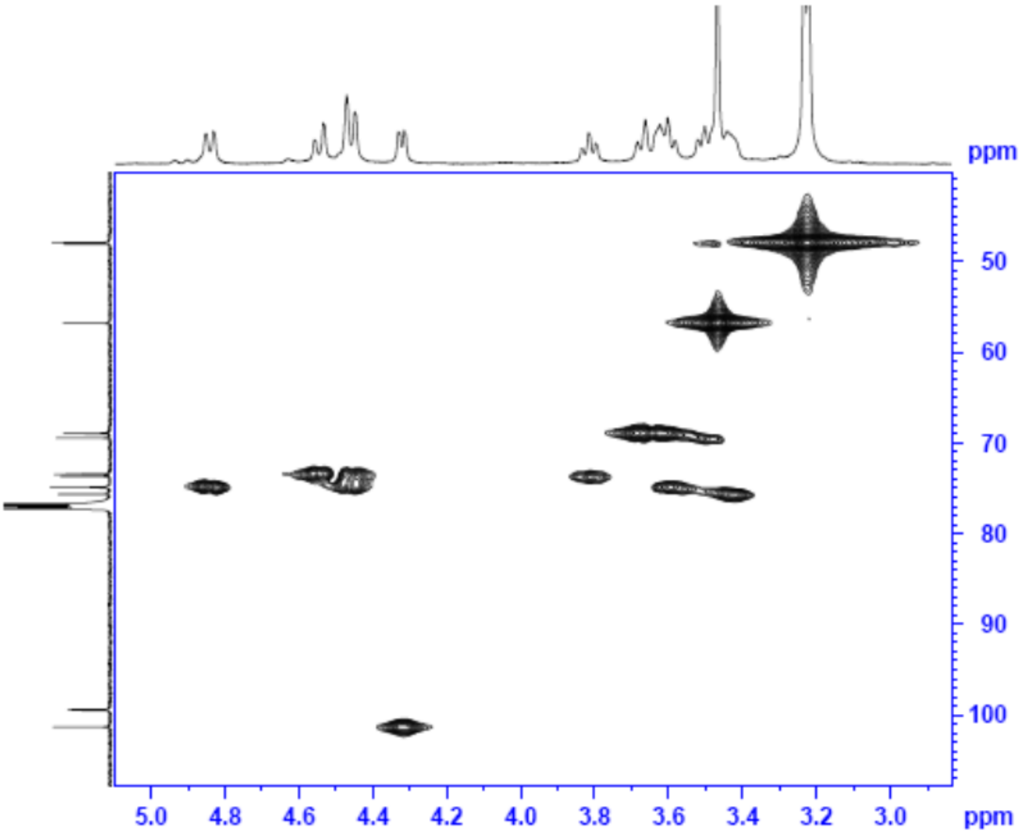

^{1}H NMR of 8α in $CDCl_3$

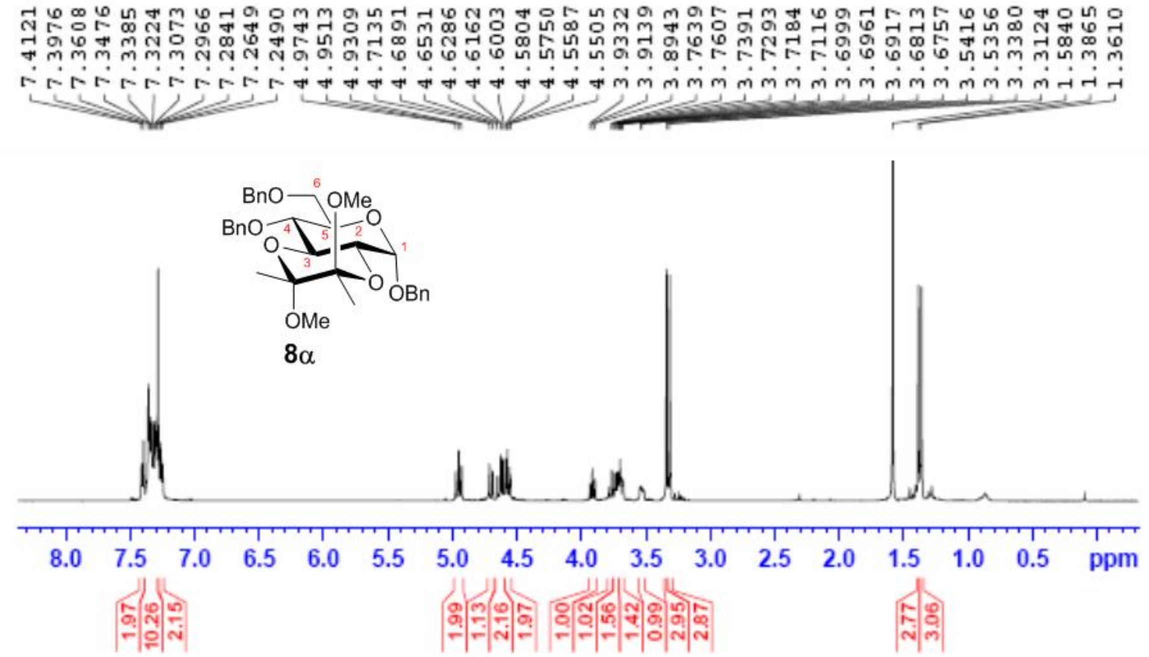

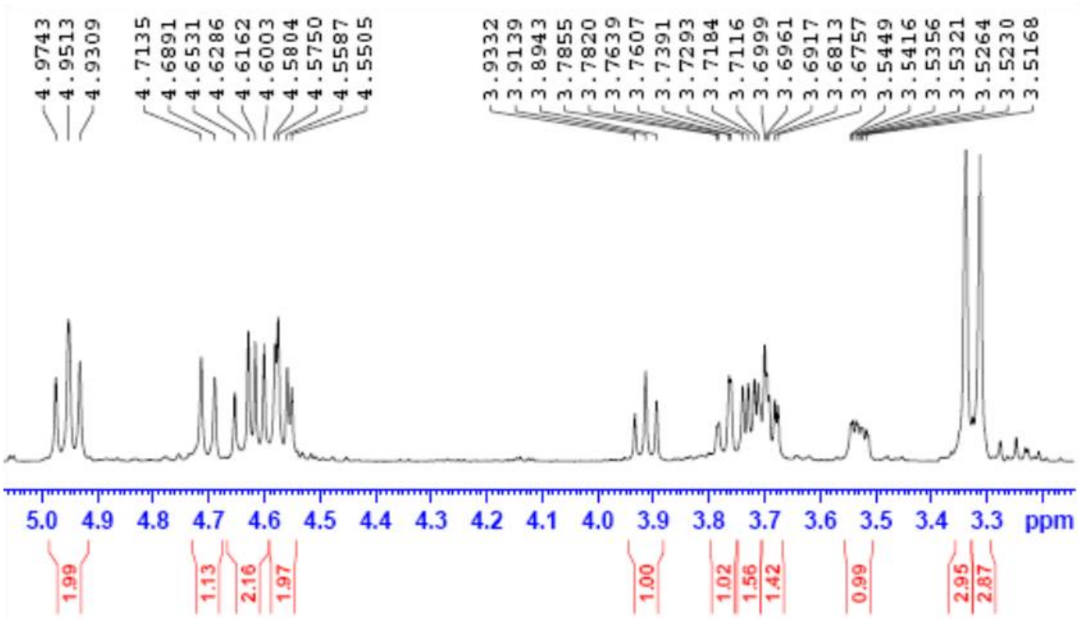

COSY NMR of 8α in CDCl₃

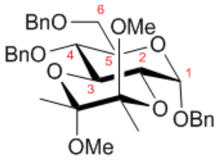

8α

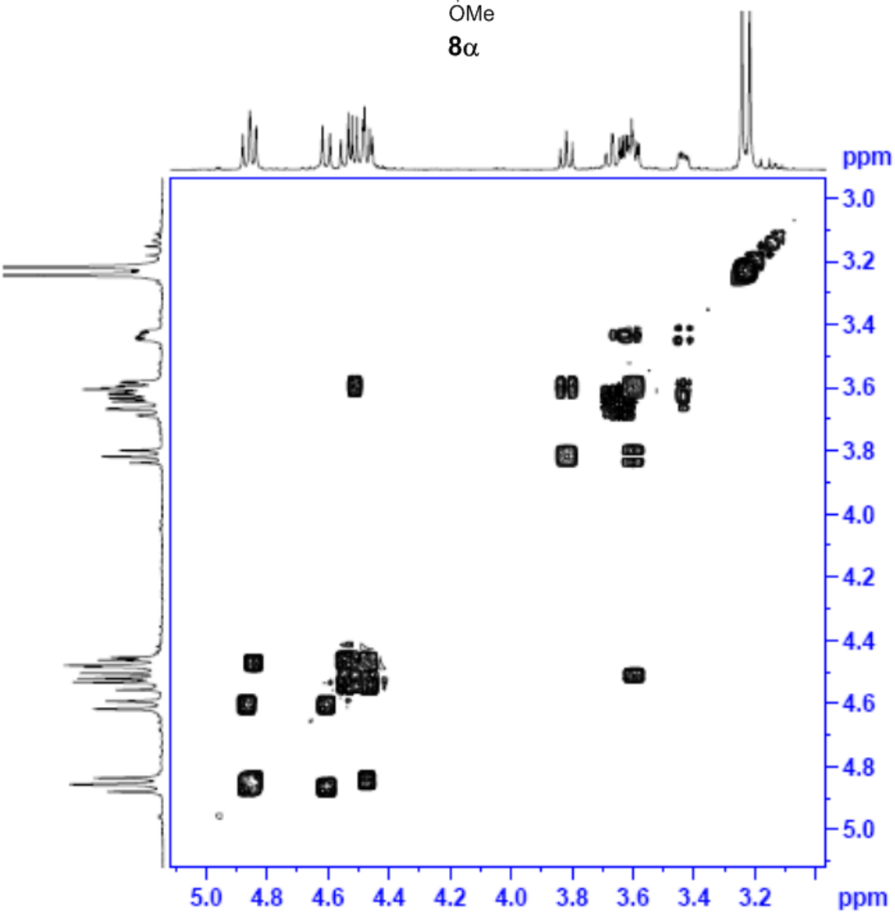

^{13}C NMR of 8α in $CDCl_3$

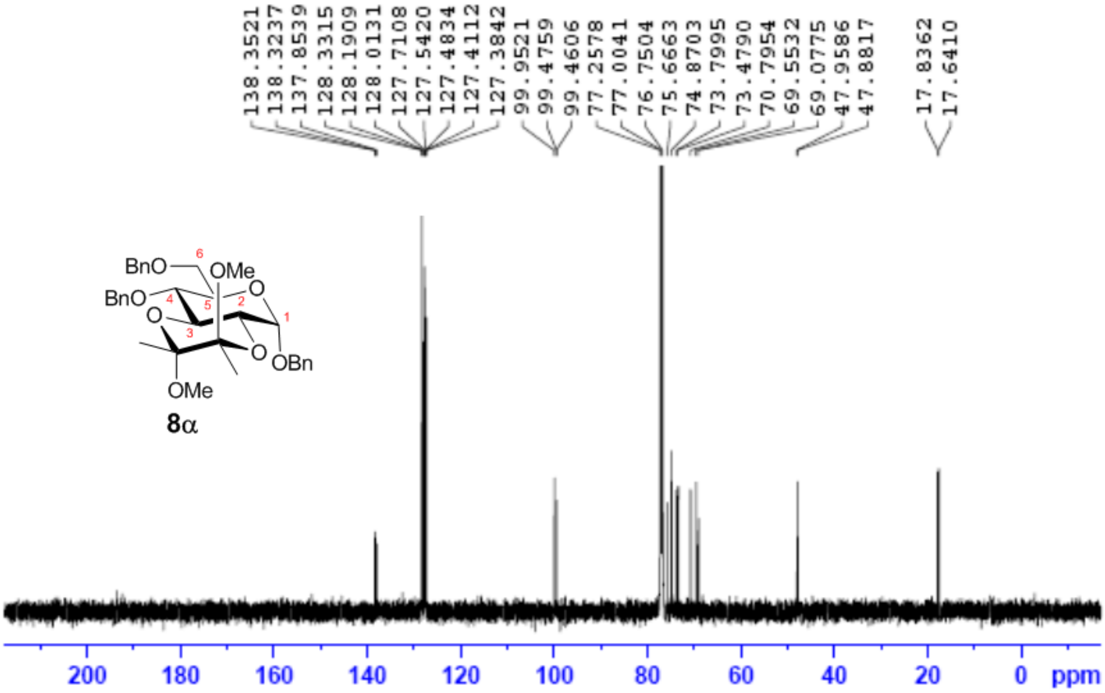

zoom

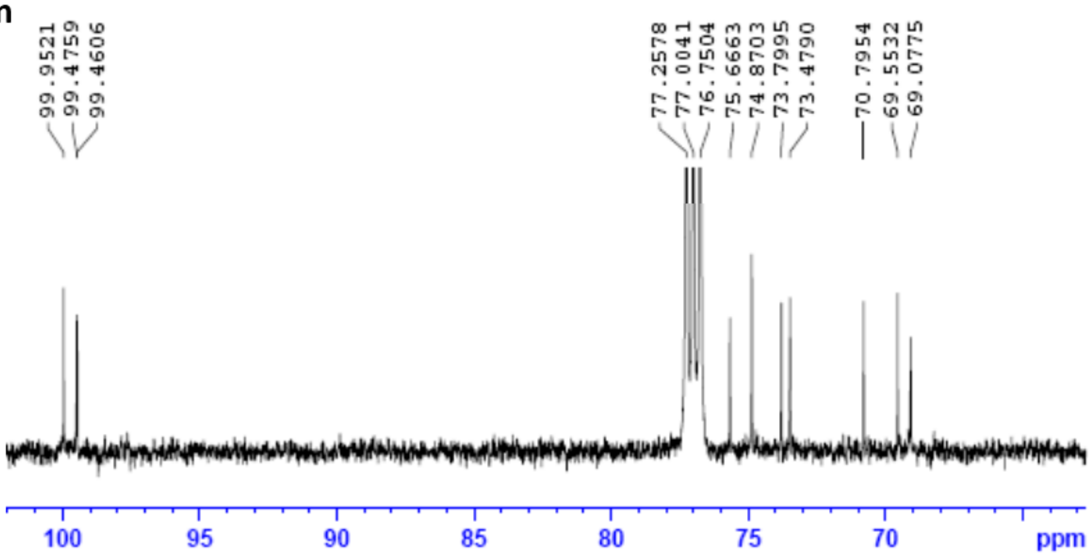

DEPT NMR of 8α in $CDCl_3$

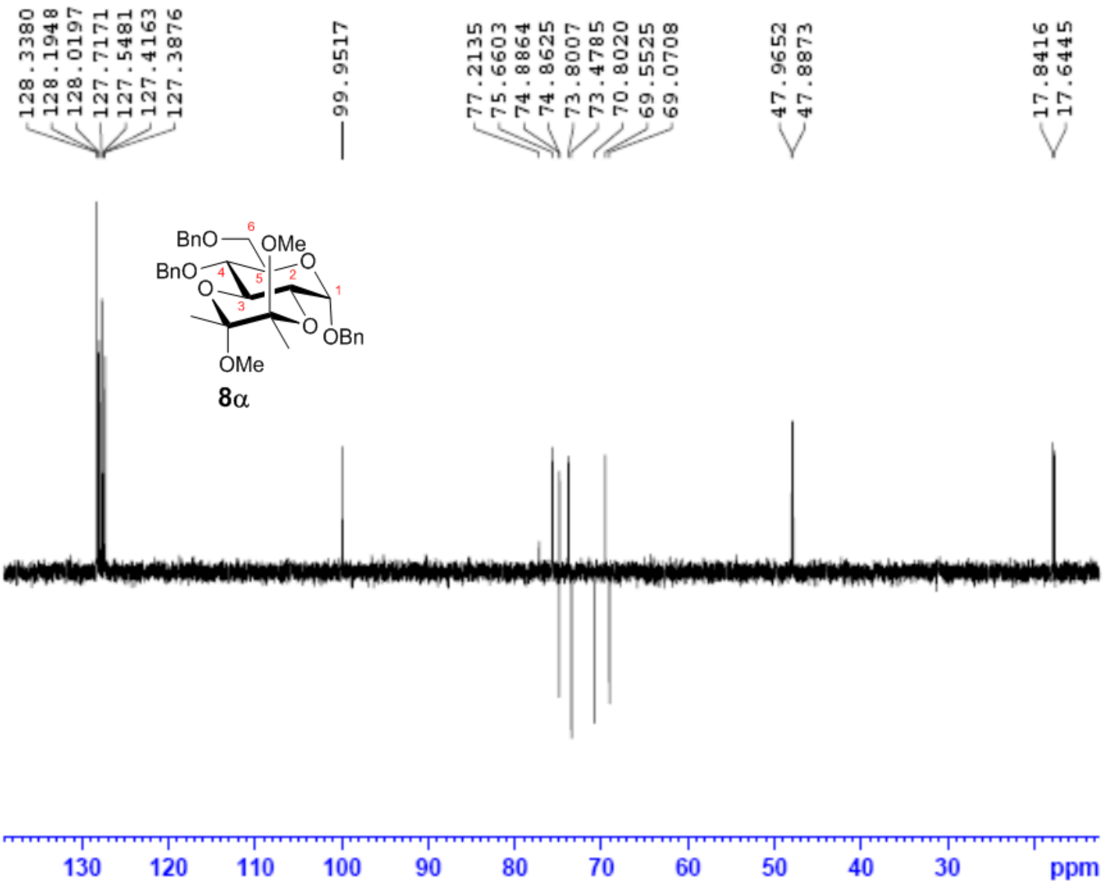

HMBC NMR of 8α in $CDCl_3$

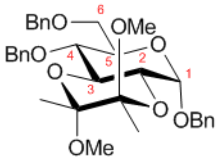

8α

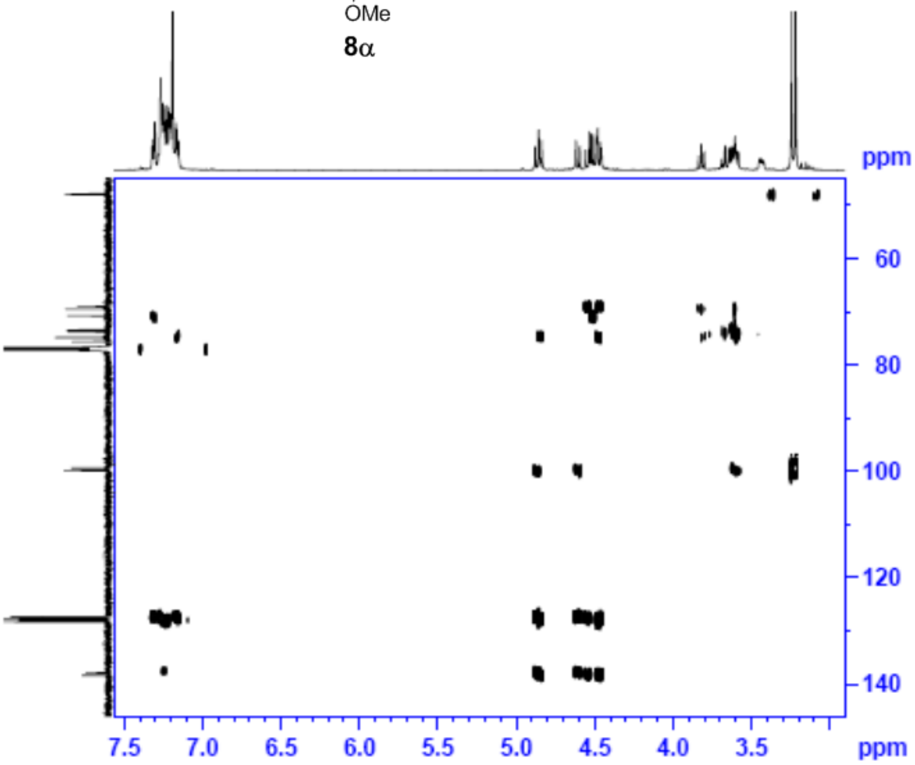

HMQC NMR of 8α in $CDCl_3$

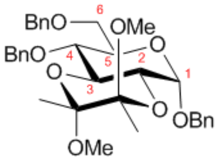

8α

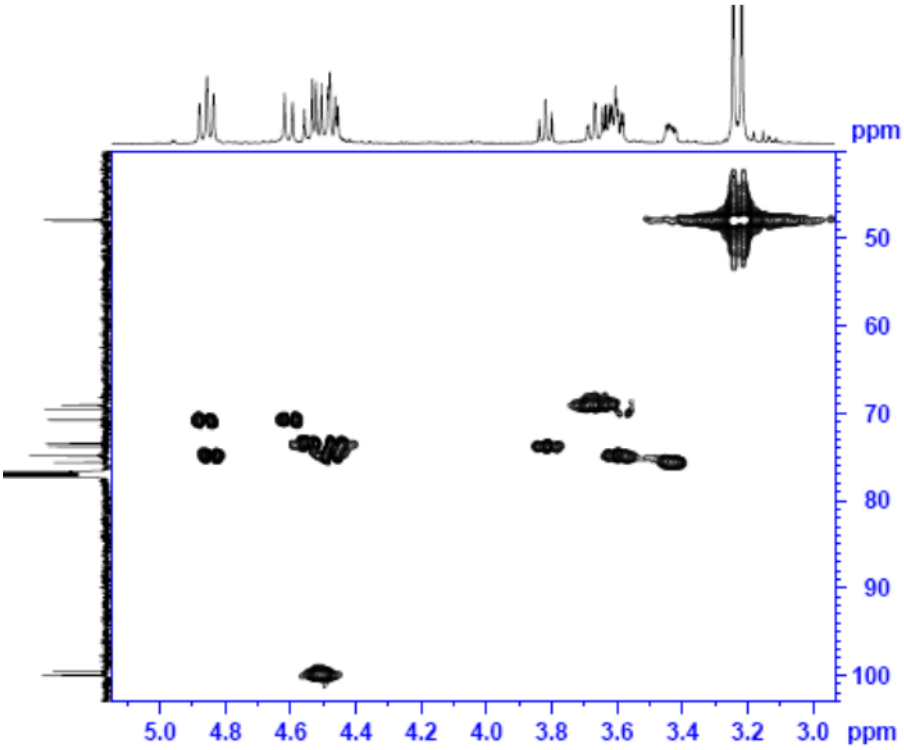

^{1}H NMR of 8β in $CDCl_3$

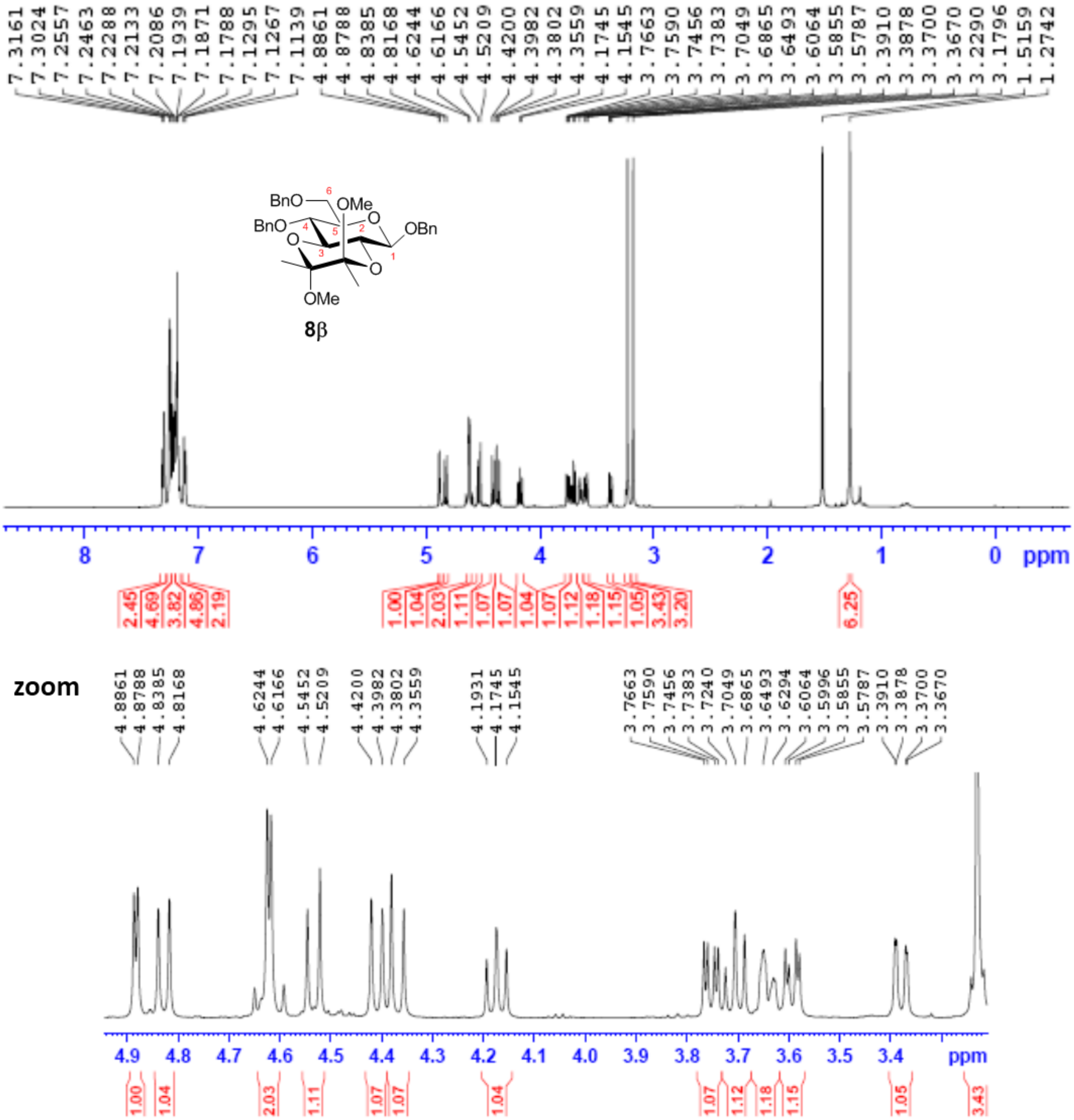

COSY NMR of 8β in $CDCl_3$

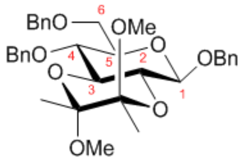

8β

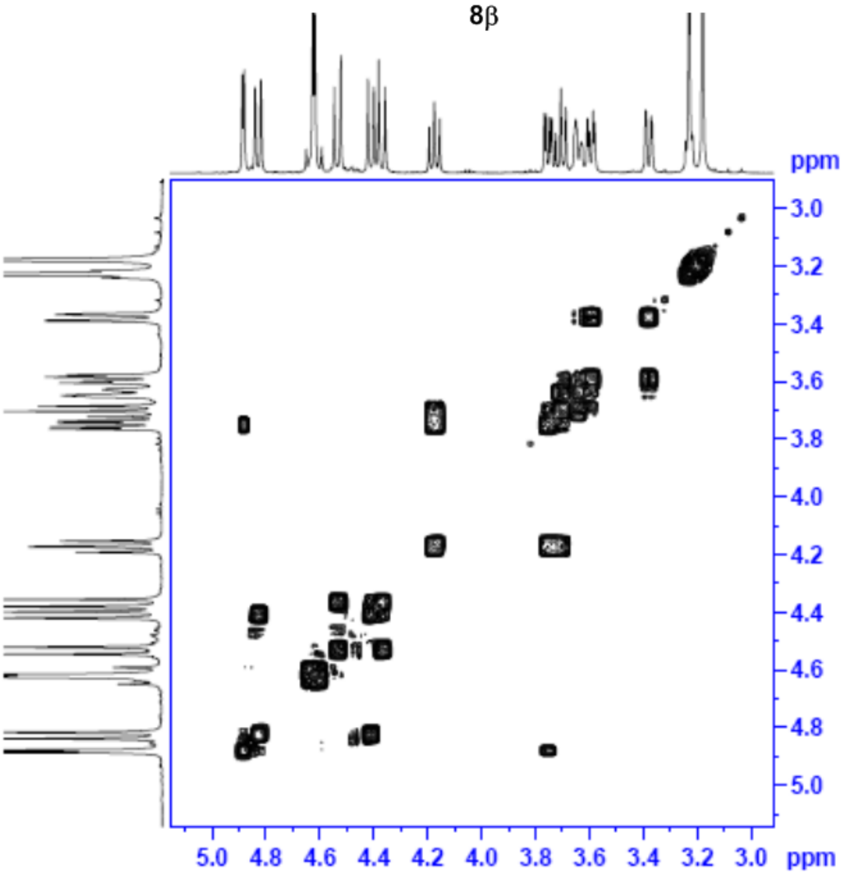

^{13}C NMR of 8β in $CDCl_3$

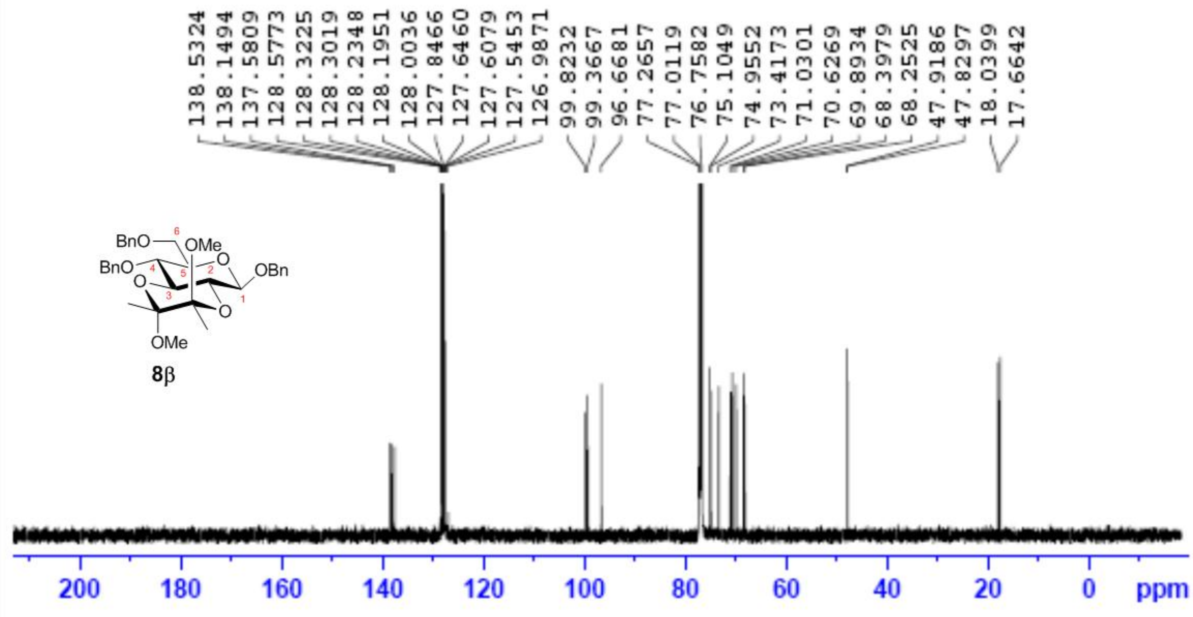

zoom

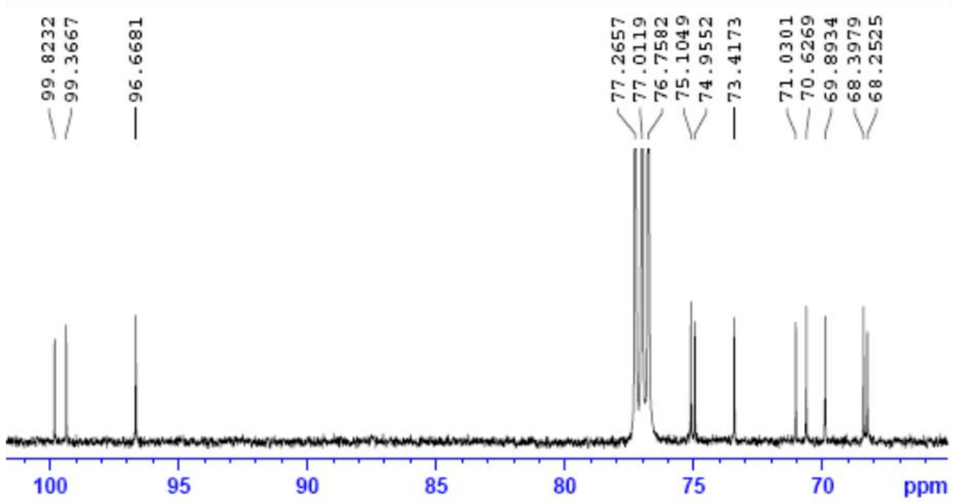

DEPT NMR of 8β in CDCl$_3$

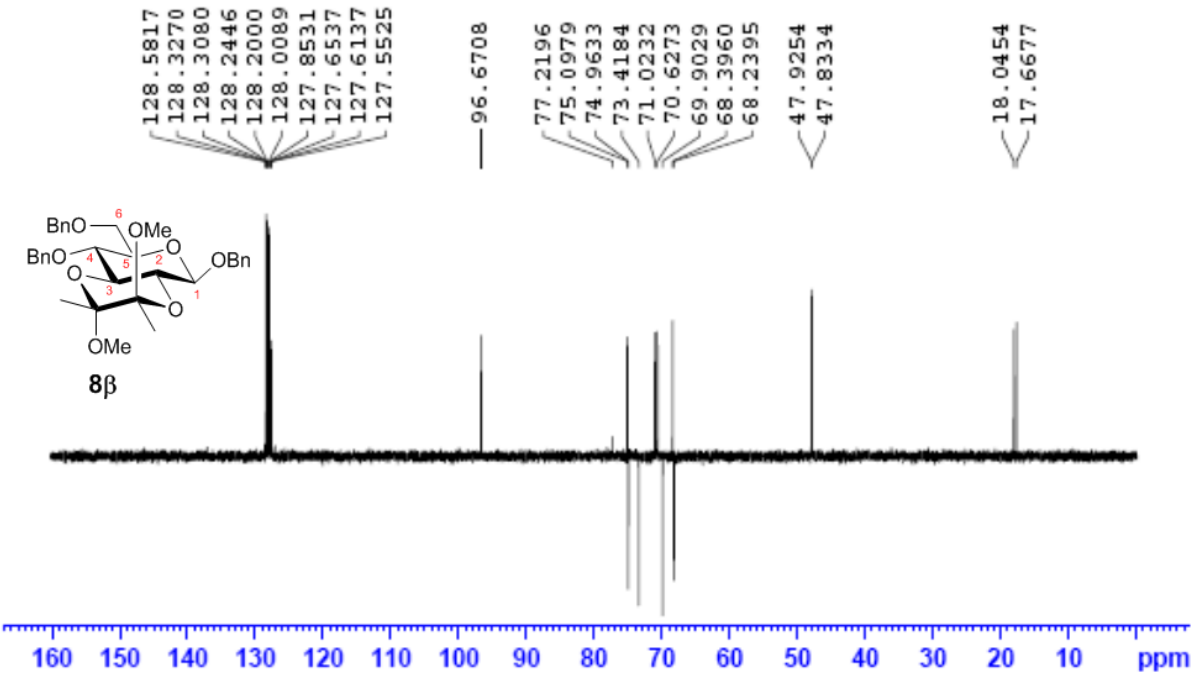

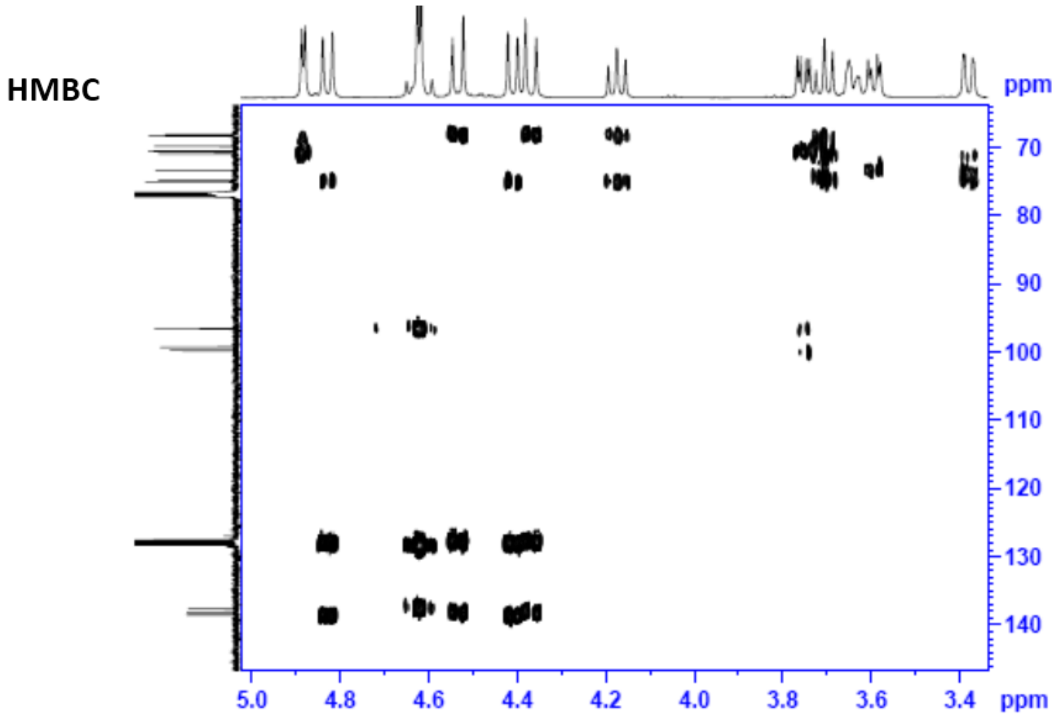

HMQC NMR of 8β in $CDCl_3$

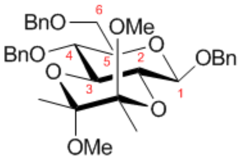

8β

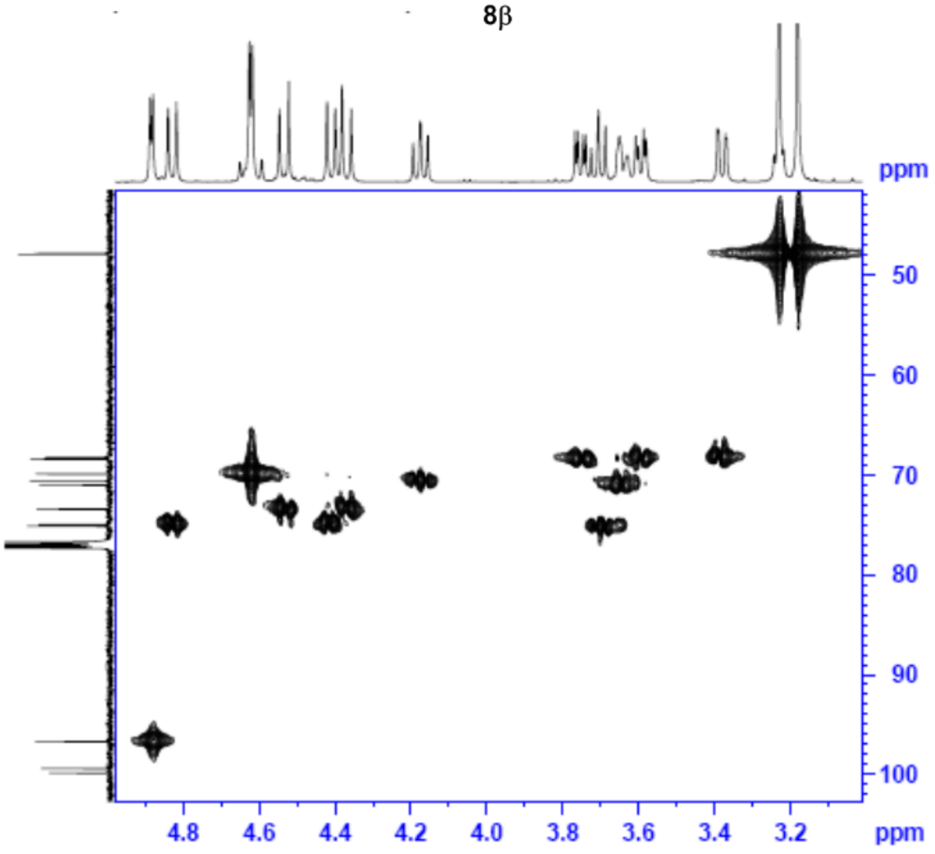

^{1}H NMR of 9α in $CDCl_3$

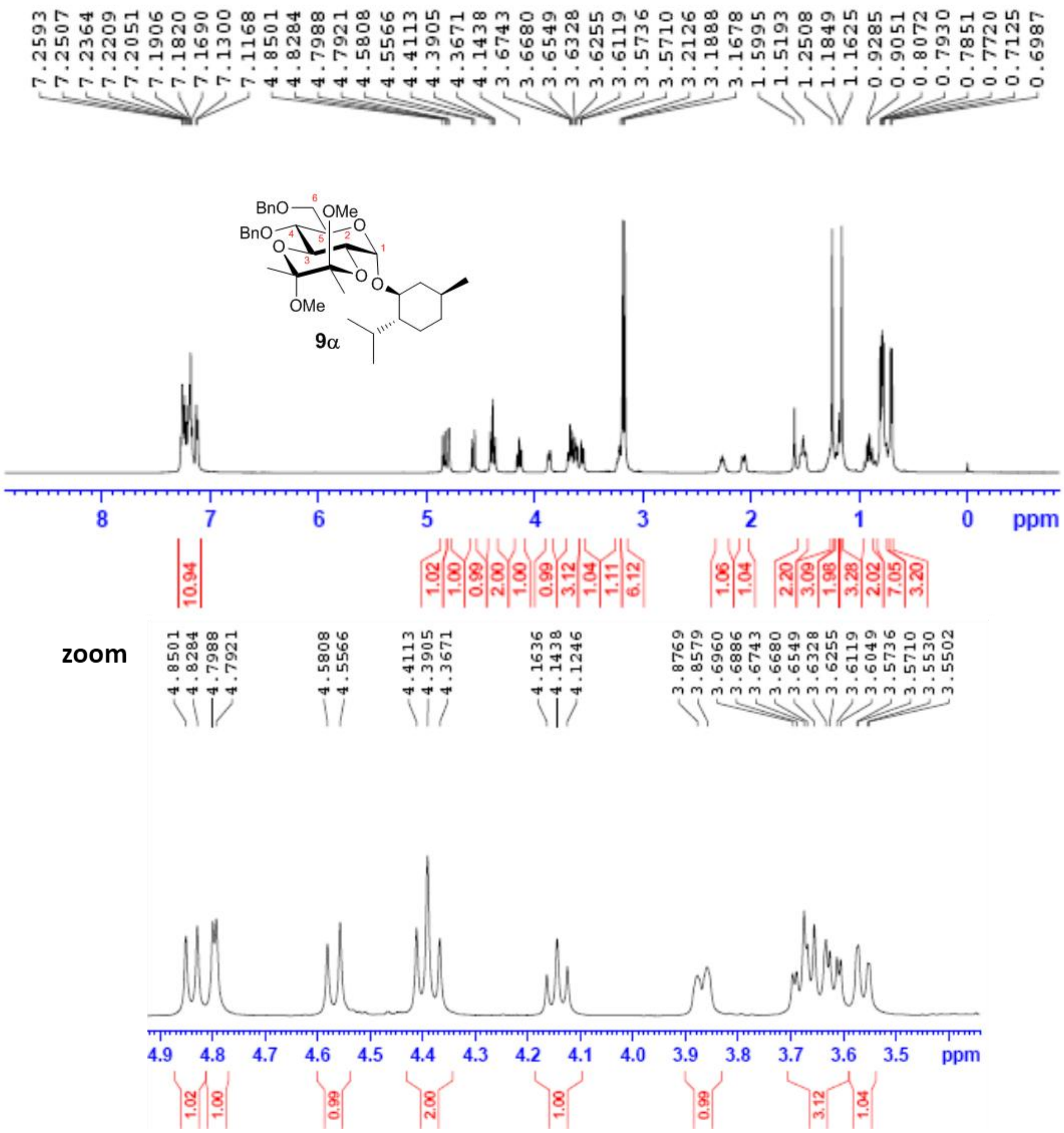

COSY NMR of 9α in $CDCl_3$

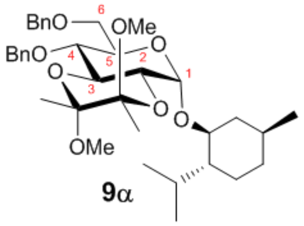

9α

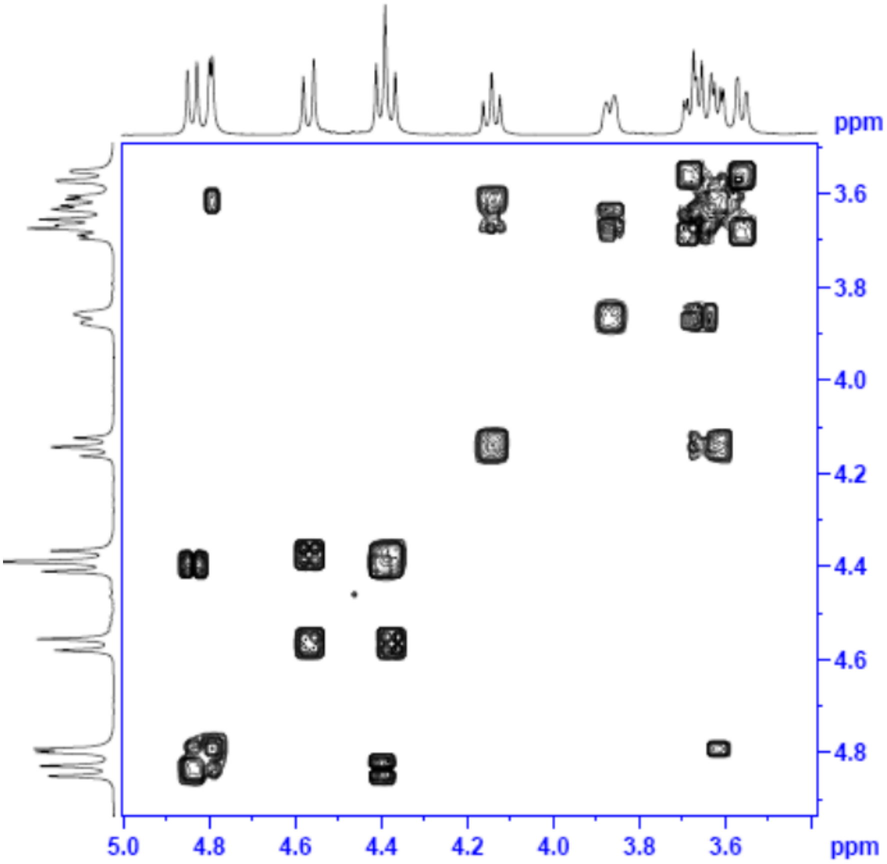

^{13}C NMR of 9α in $CDCl_3$

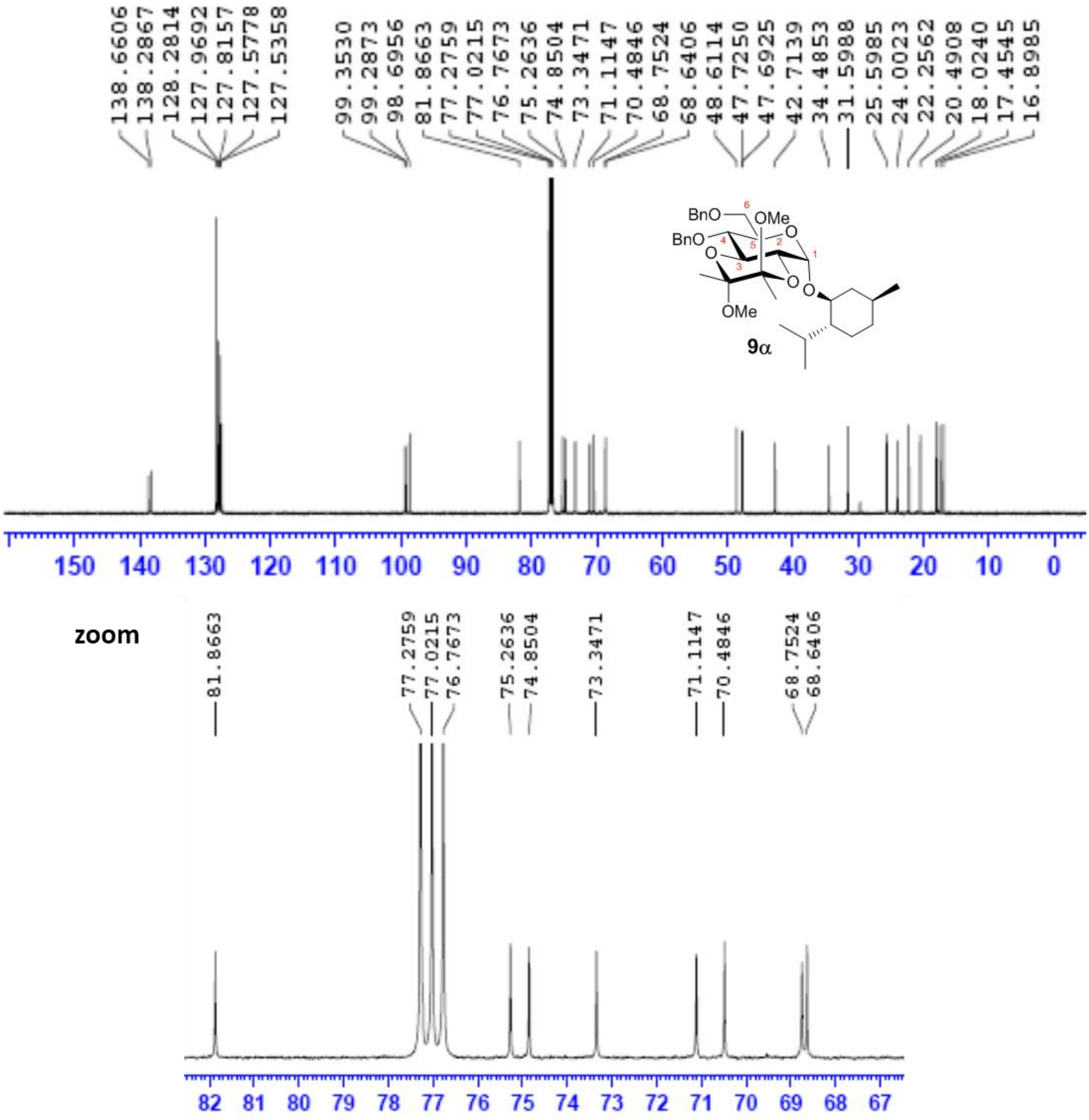

DEPT NMR of 9α in $CDCl_3$

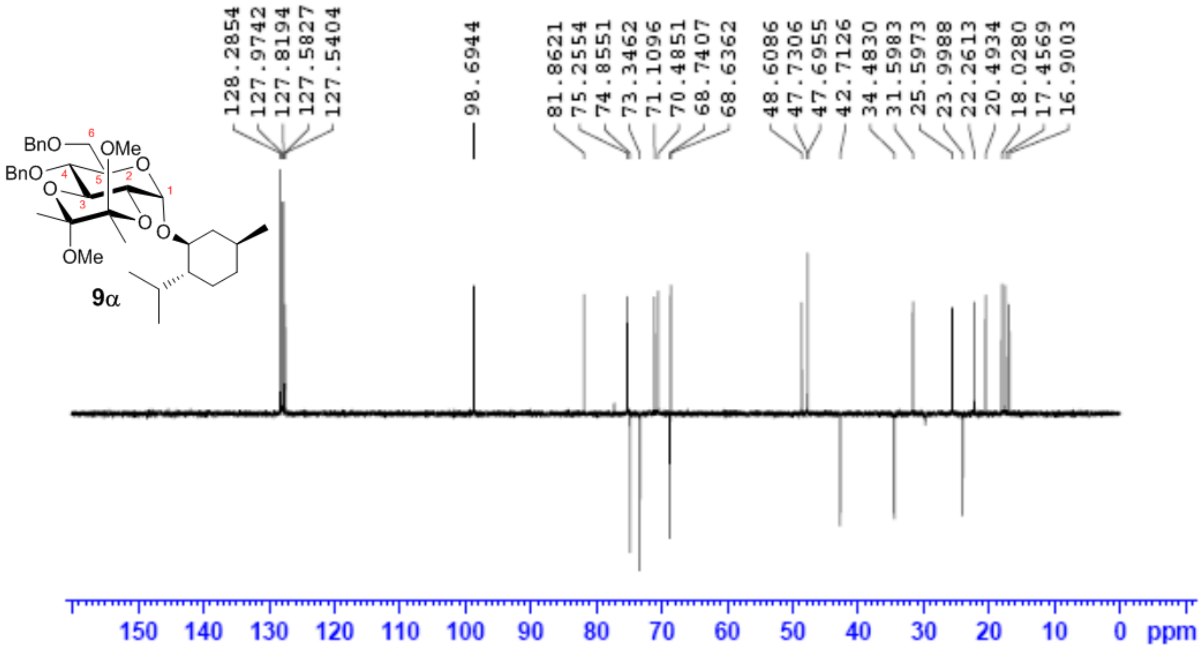

HMBC

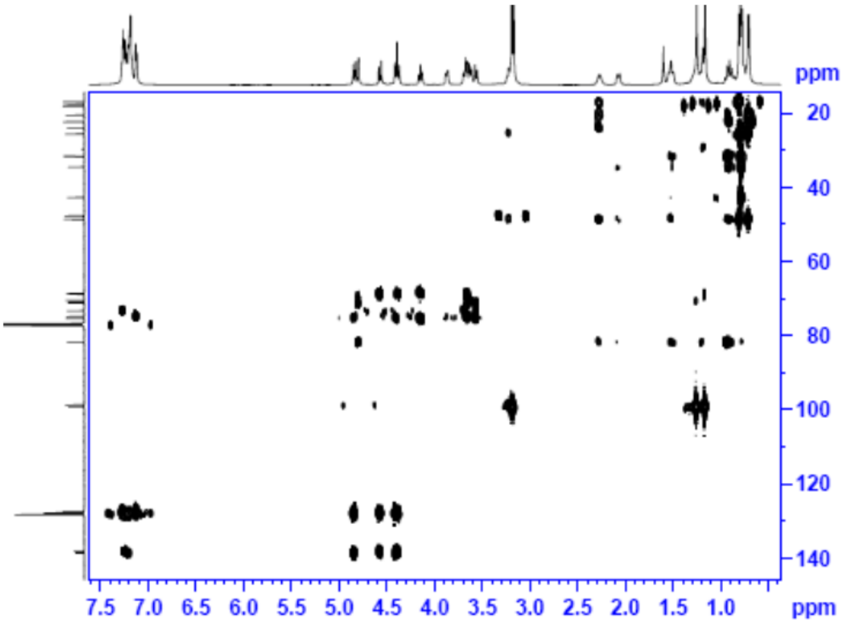

HMQC NMR of 9α in $CDCl_3$

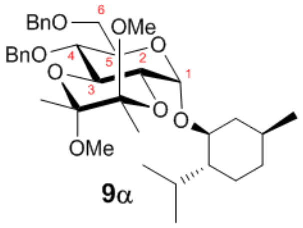

9α

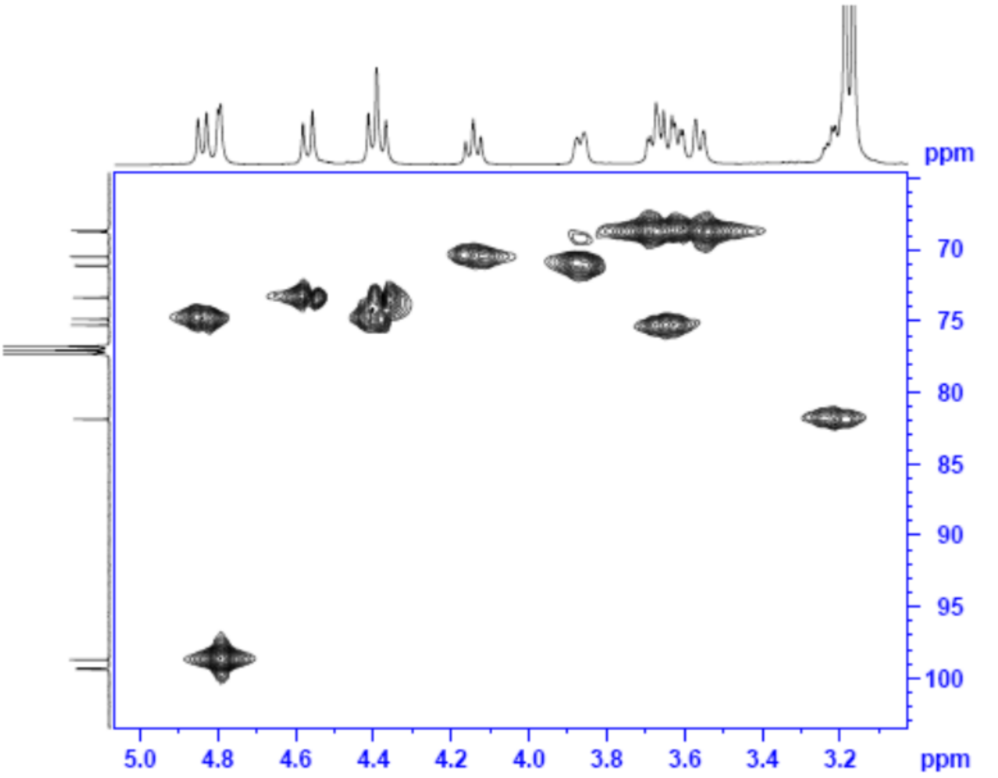

^{1}H NMR of 9β in $CDCl_3$

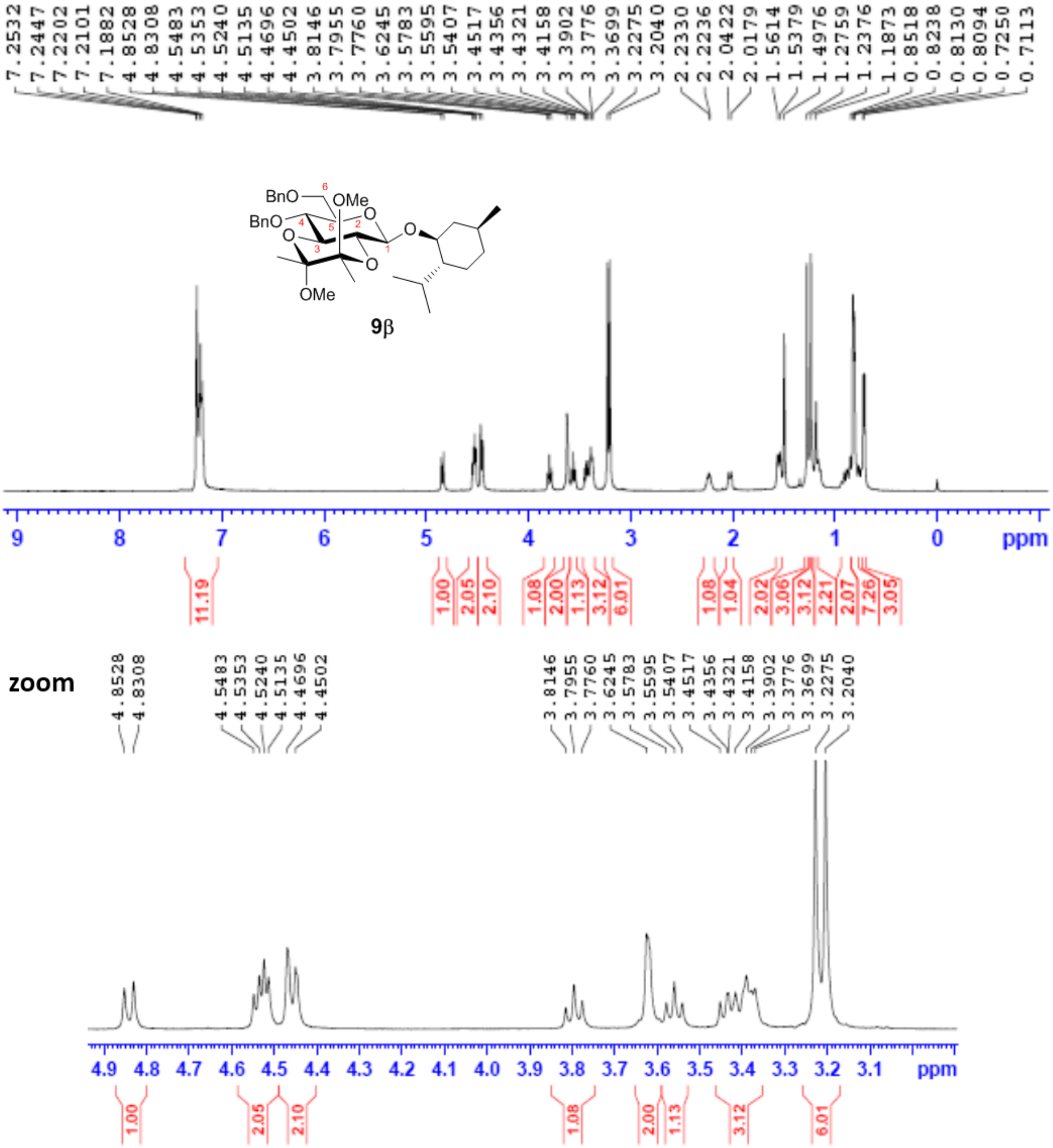

COSY NMR of 9β in $CDCl_3$

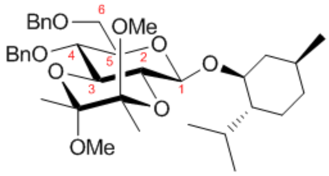

9β

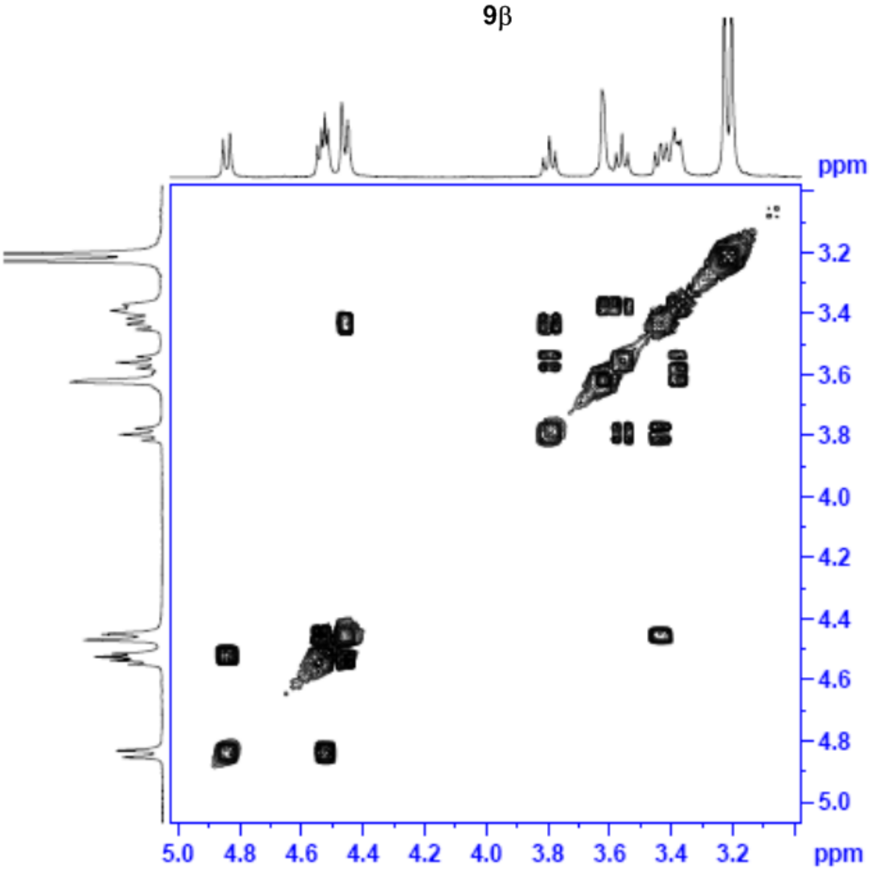

^{13}C NMR of 9β in $CDCl_3$

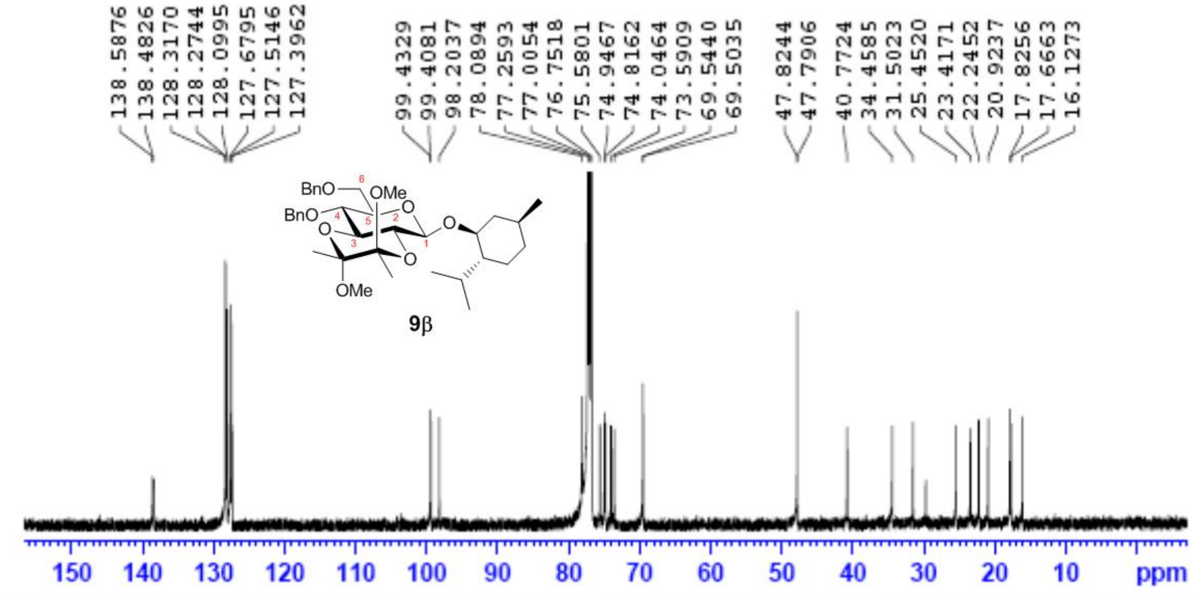

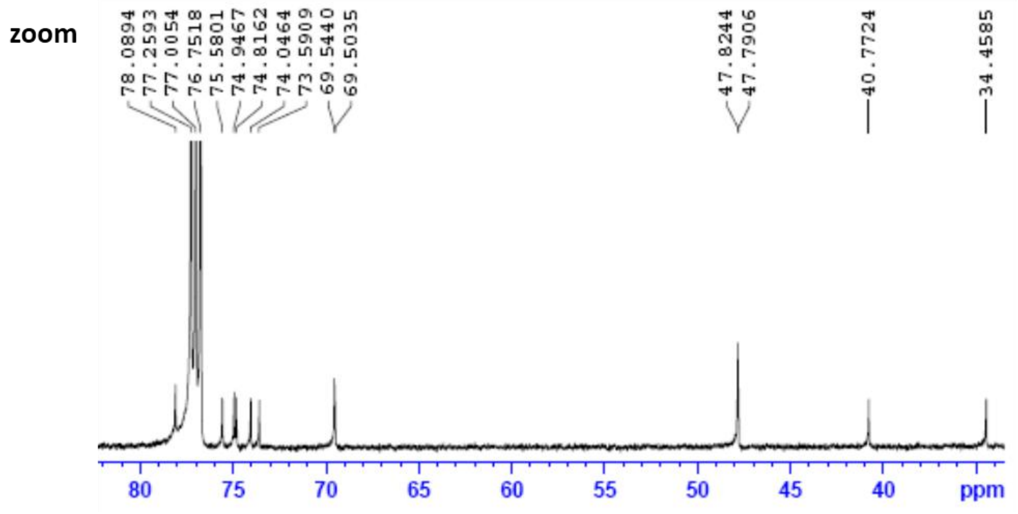

DEPT NMR of 9β in $CDCl_3$

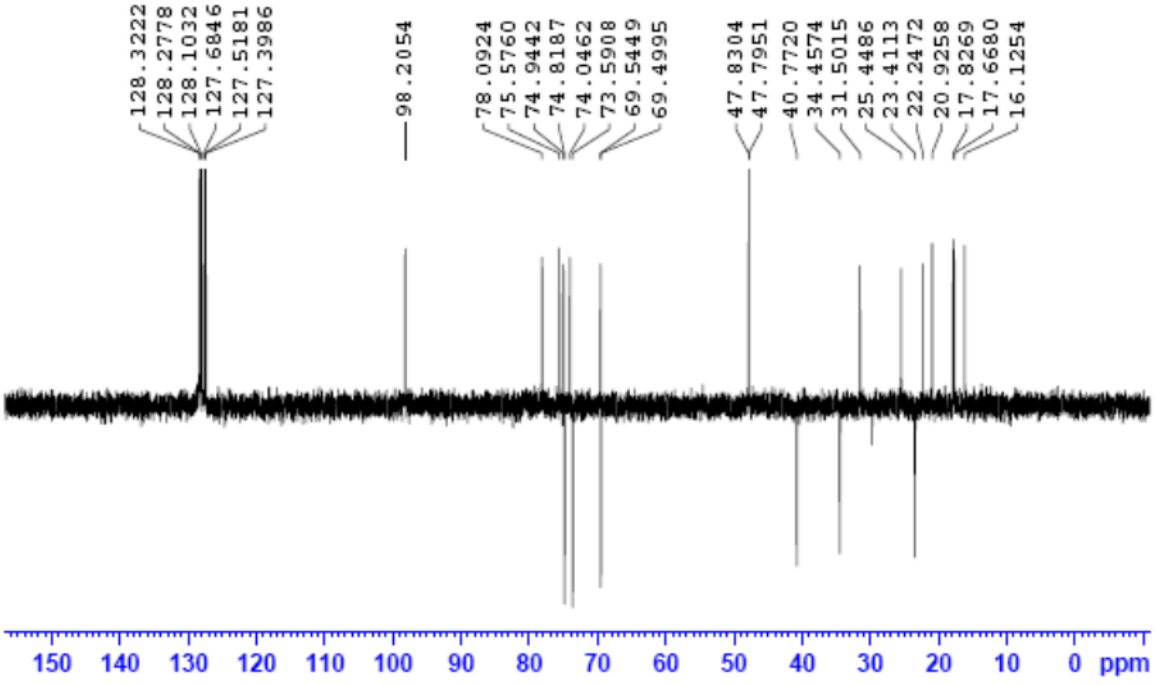

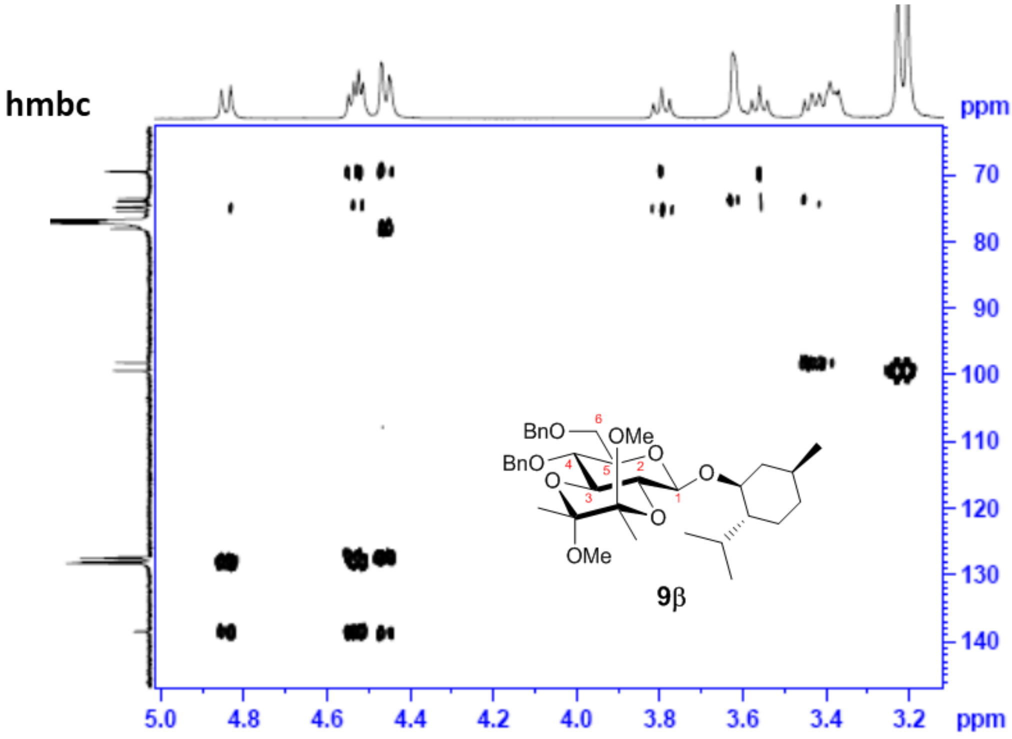

HMQC NMR of 9β in $CDCl_3$

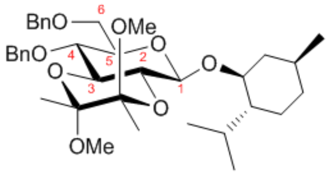

9β

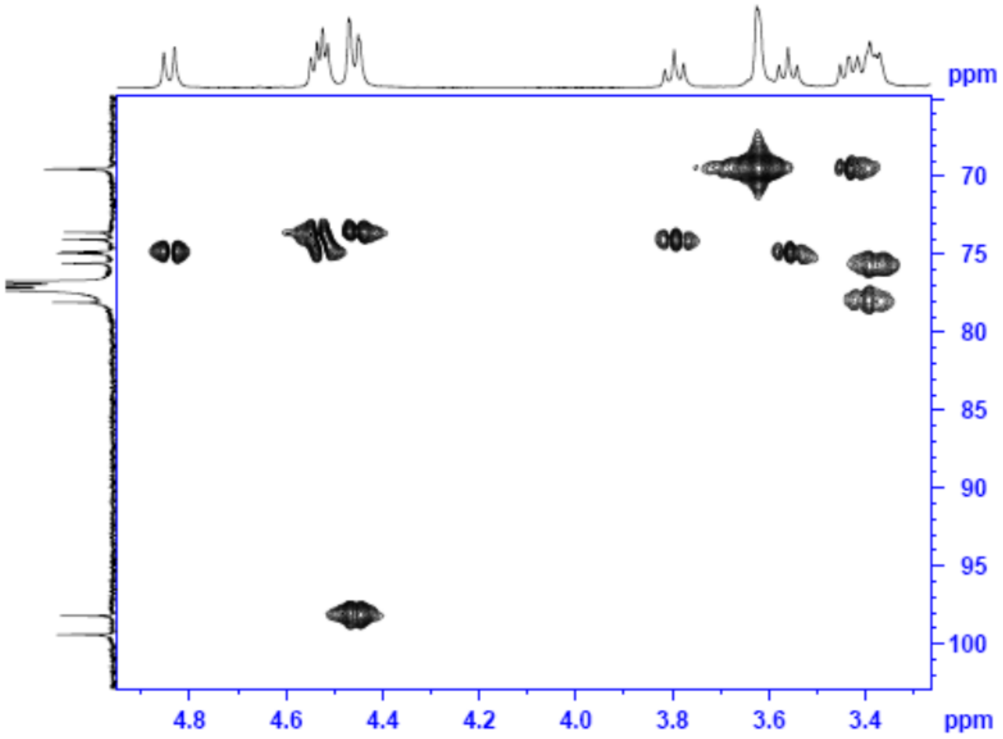

^{1}H NMR of 10αβ (α major) in CDCl$_3$

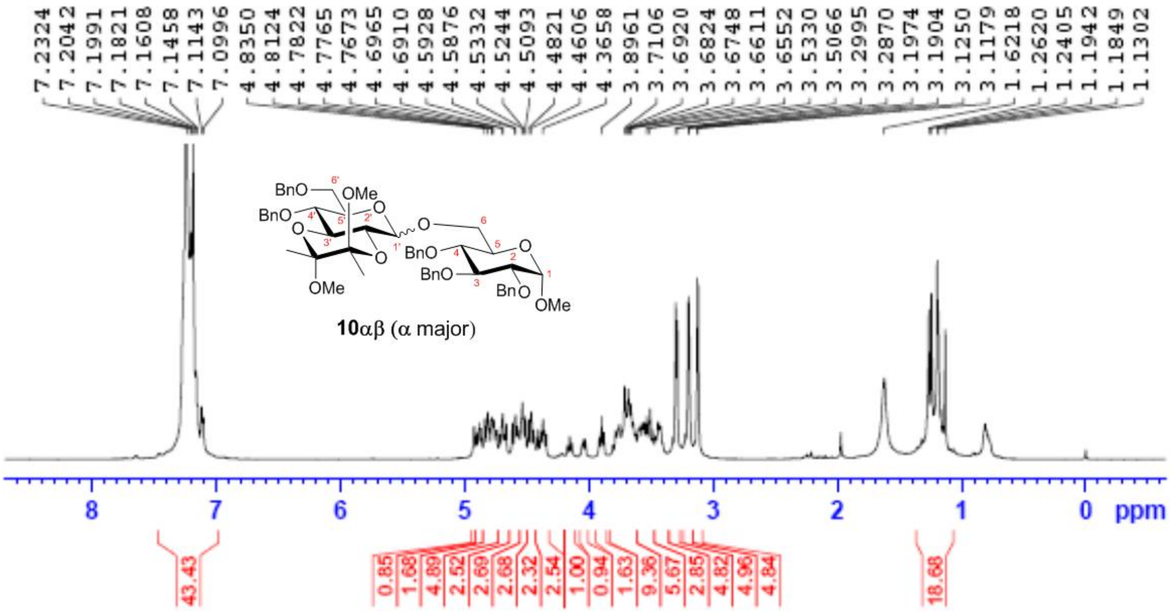

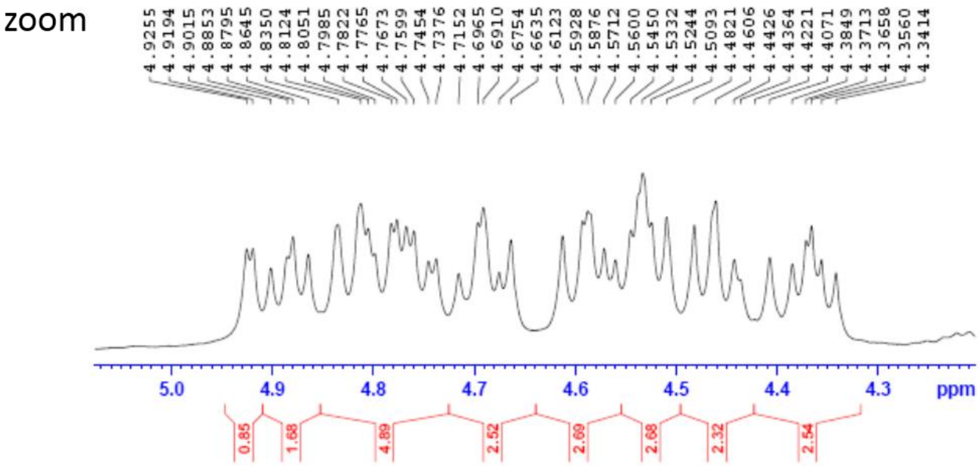

COSY NMR of 10αβ (α major) in CDCl$_3$

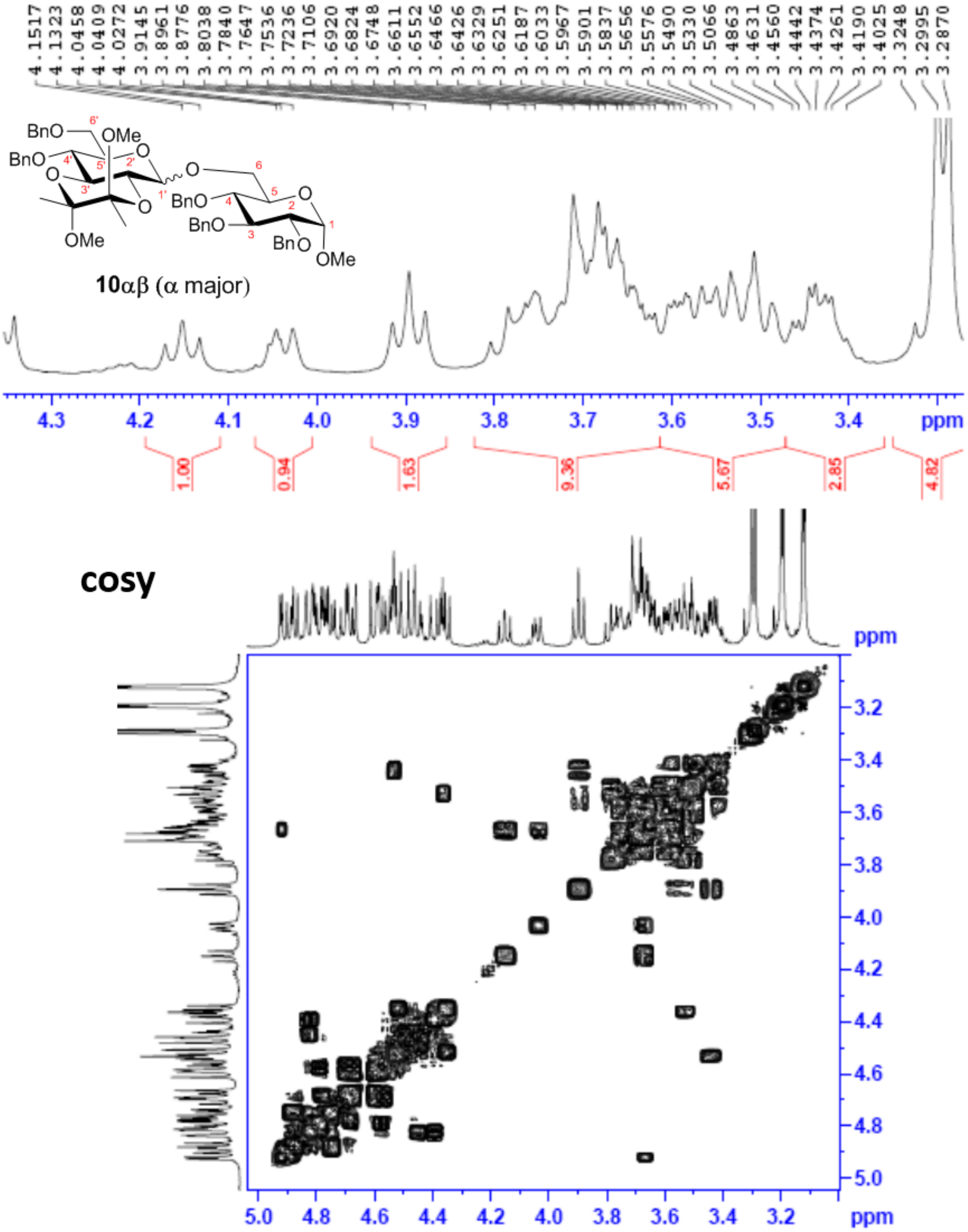

^{13}C NMR of 10αβ (α major) in $CDCl_3$

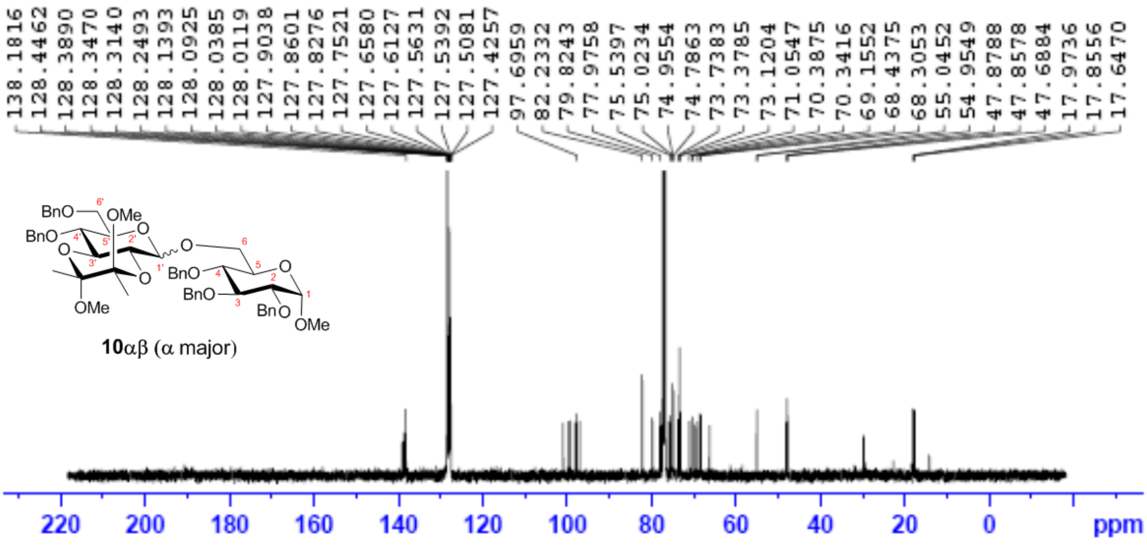

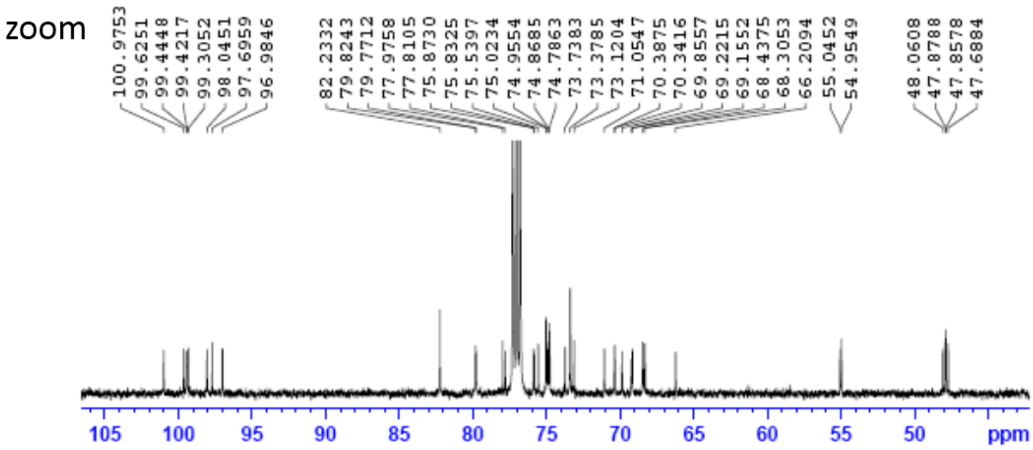

DEPT NMR of 10αβ (α major) in CDCl$_3$

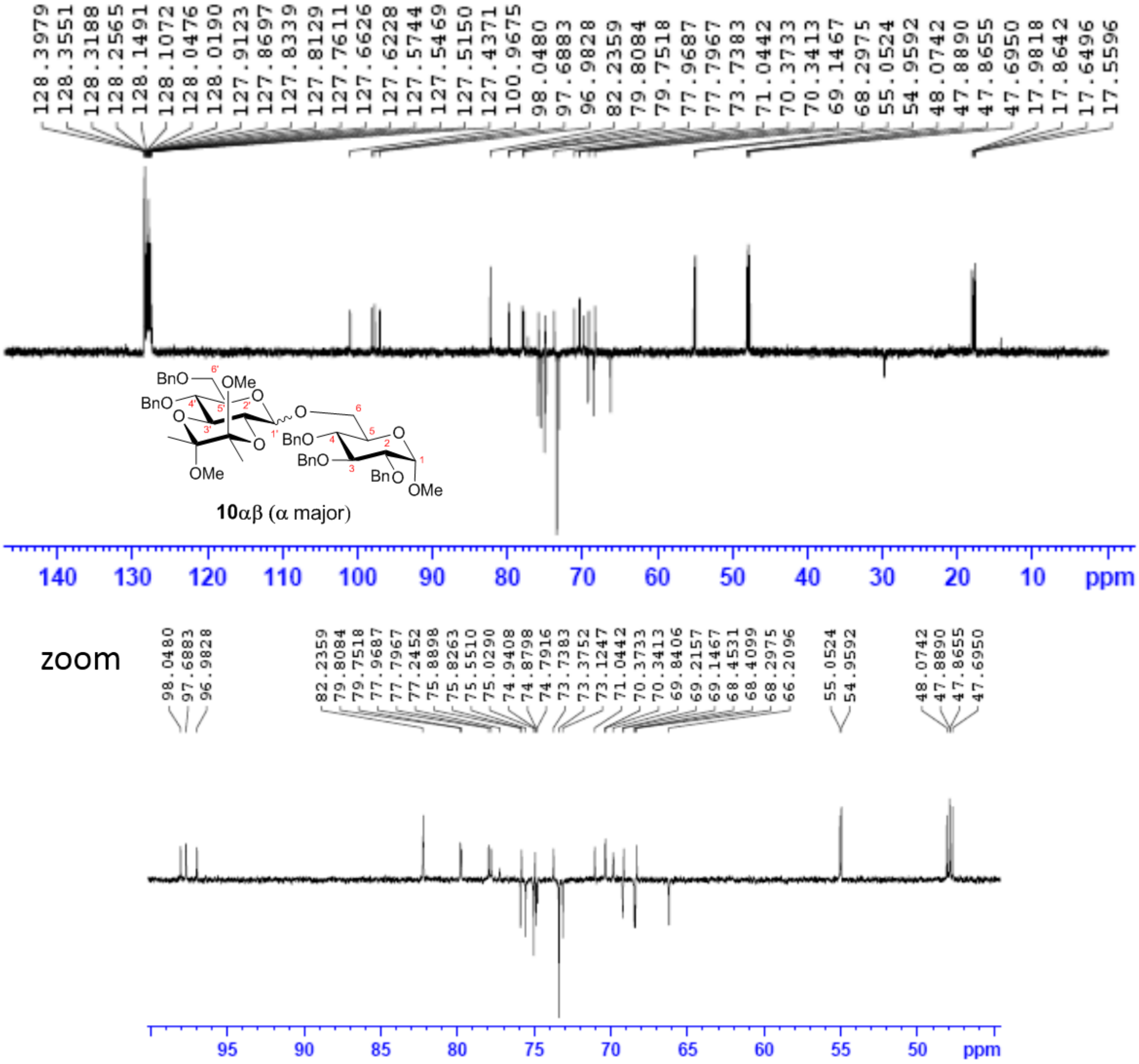

HMBC NMR of 10αβ (α major) in $CDCl_3$

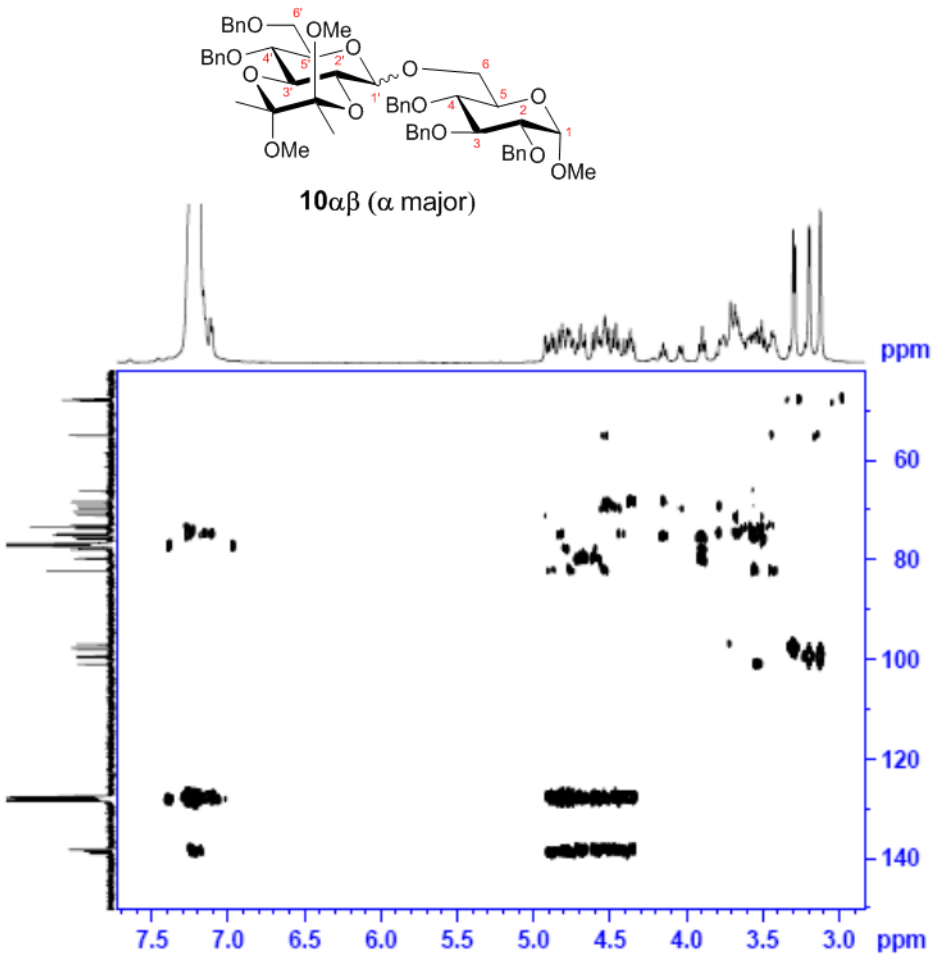

HMQC NMR of 10αβ (α major) in $CDCl_3$

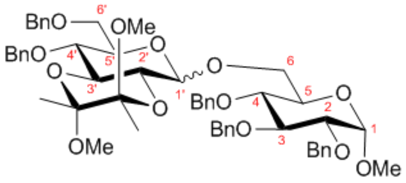

10αβ (α major)

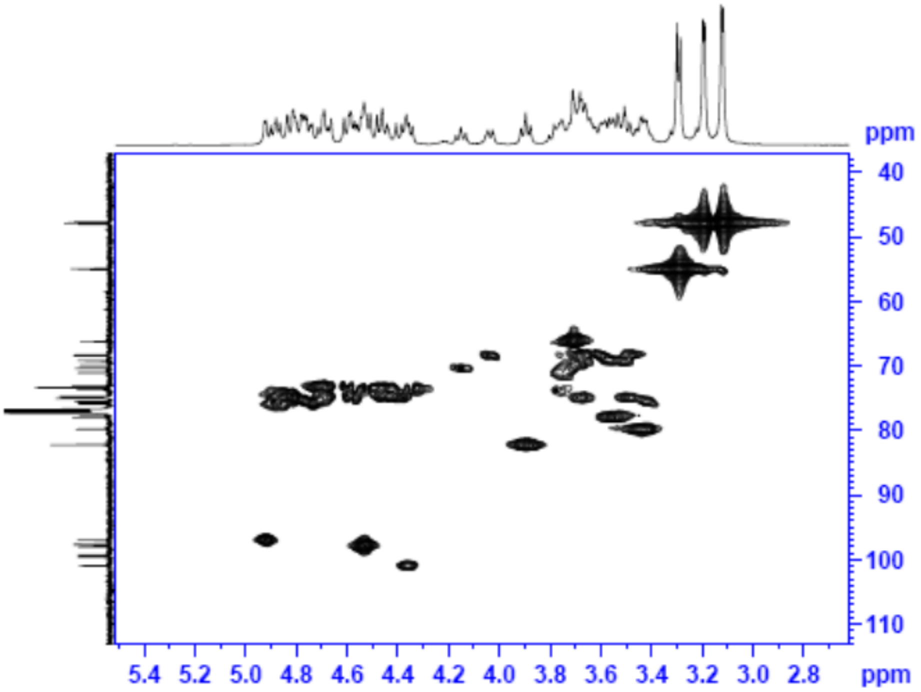

^{1}H NMR of 10αβ (β major) in $CDCl_3$

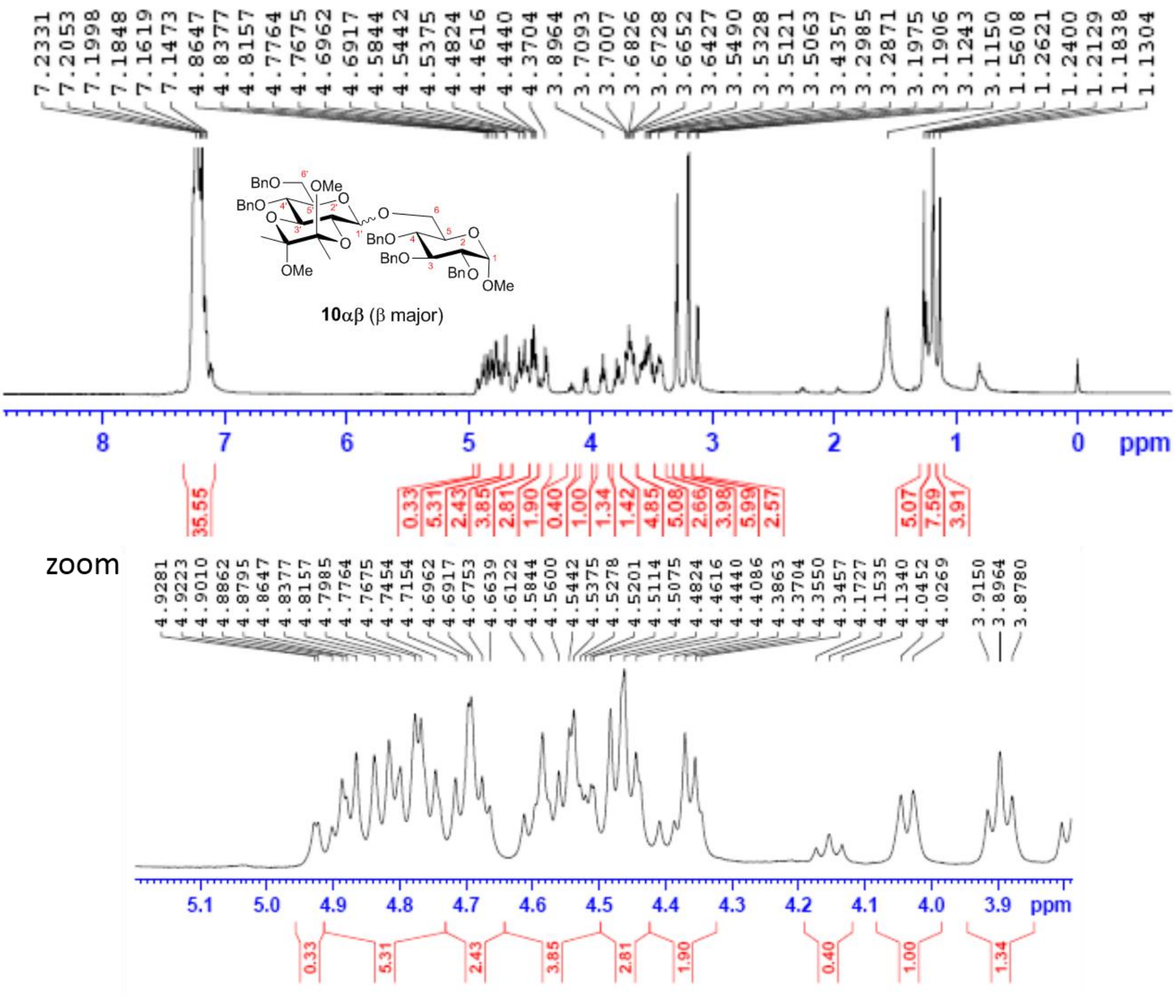

COSY NMR of 10αβ (β major) in $CDCl_3$

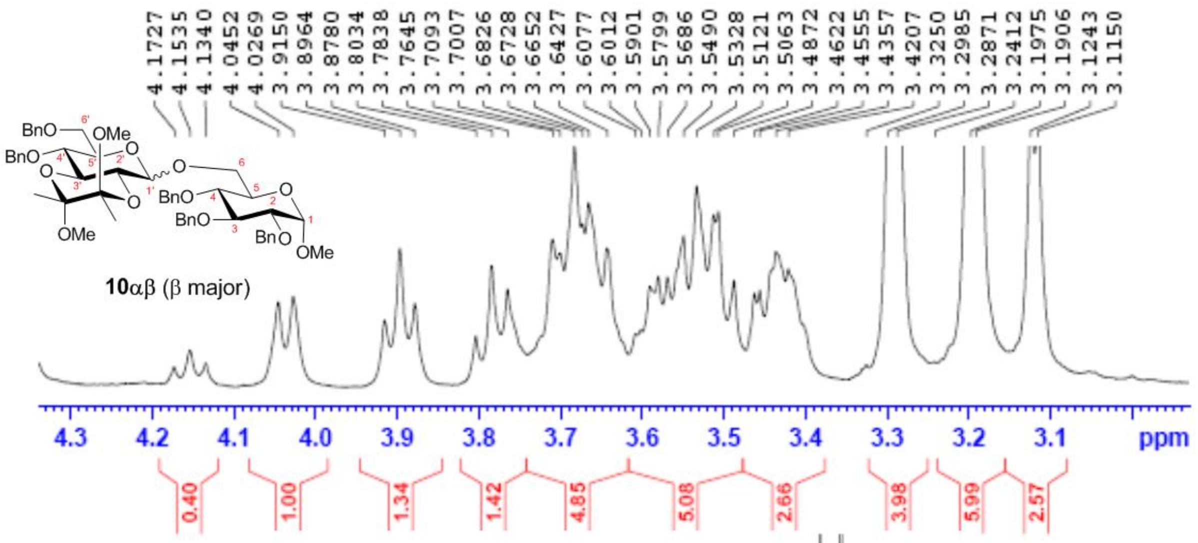

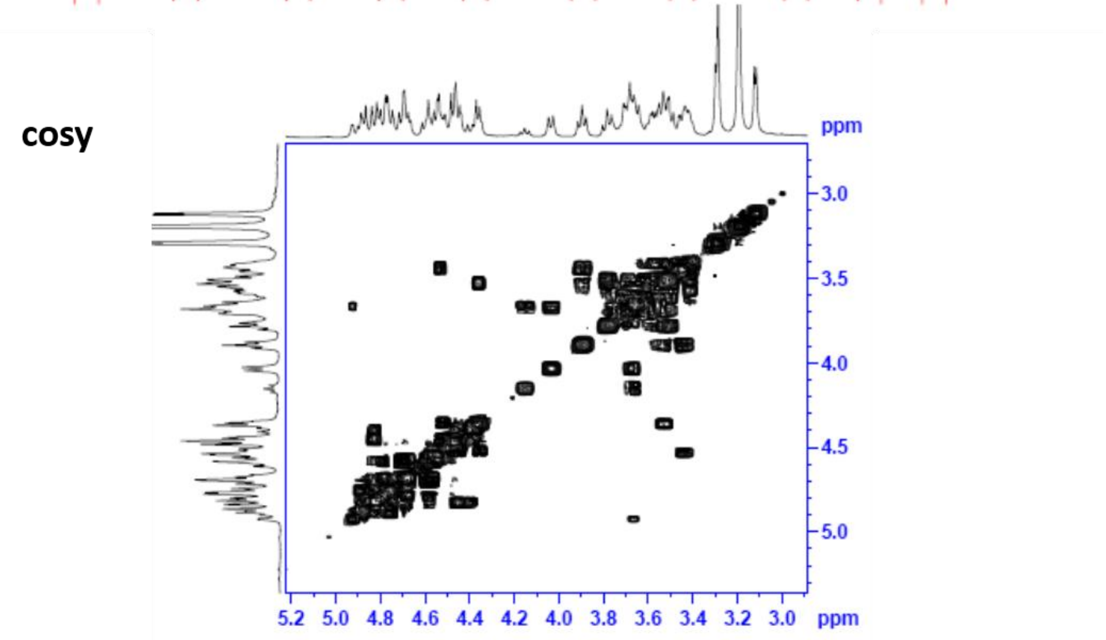

^{1}H NMR of 11α in $CDCl_3$

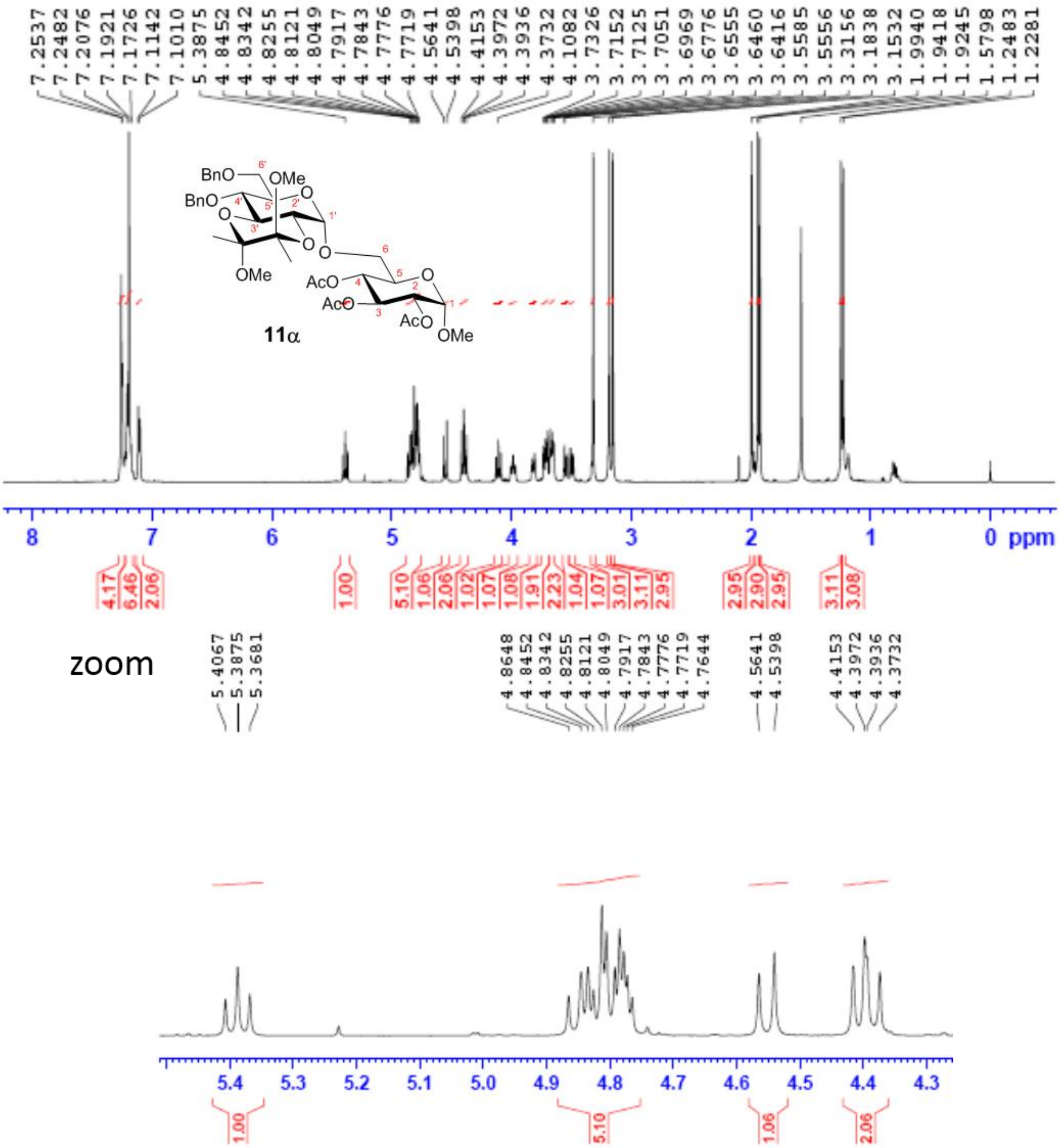

COSY NMR of 11α in CDCl₃

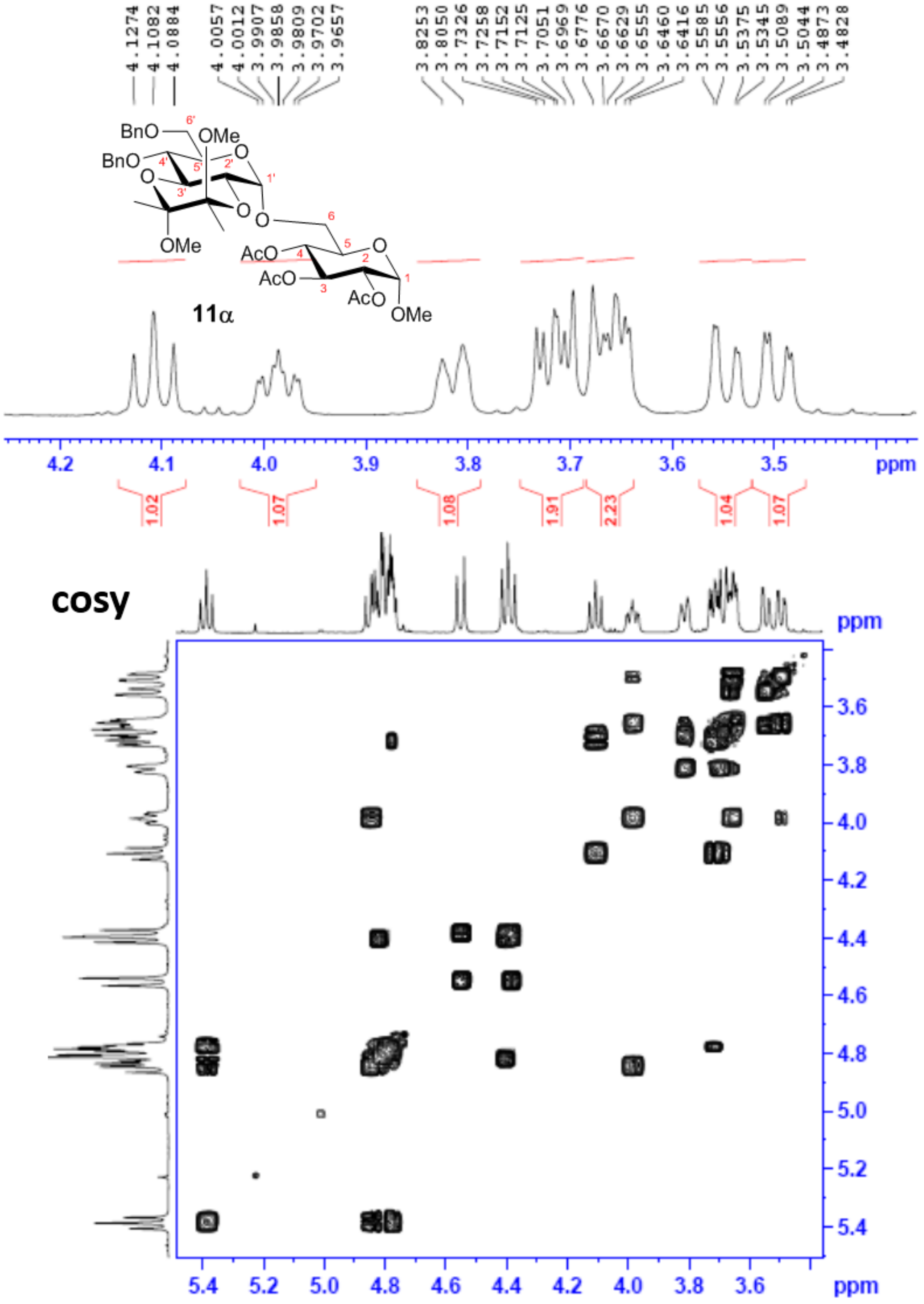

^{13}C NMR of 11*α* in $CDCl_3$

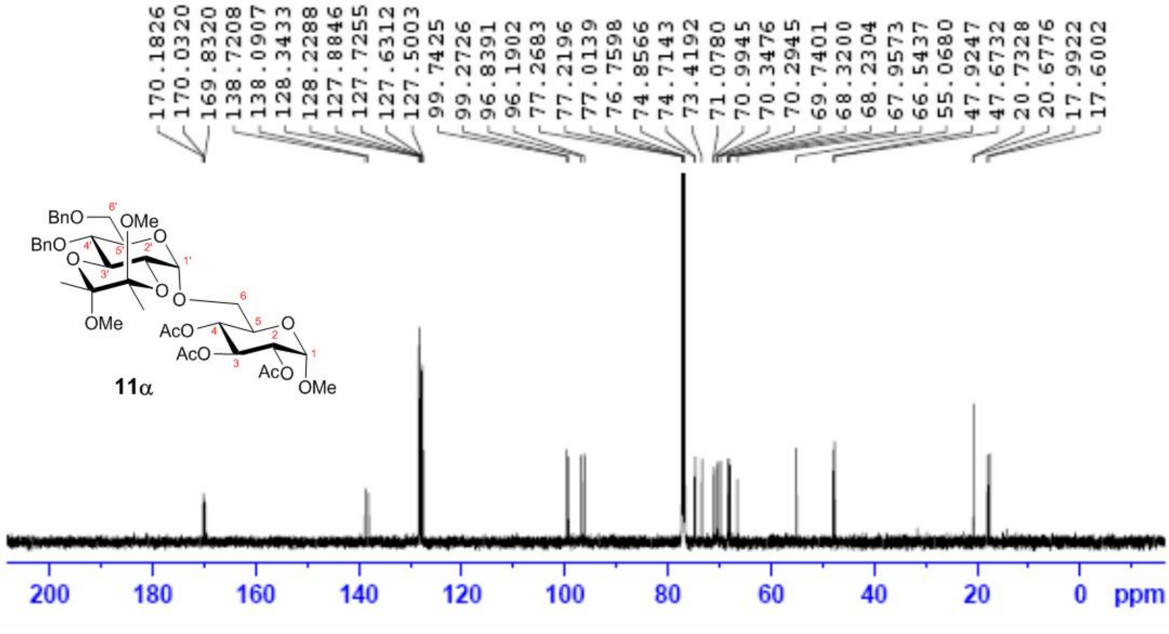

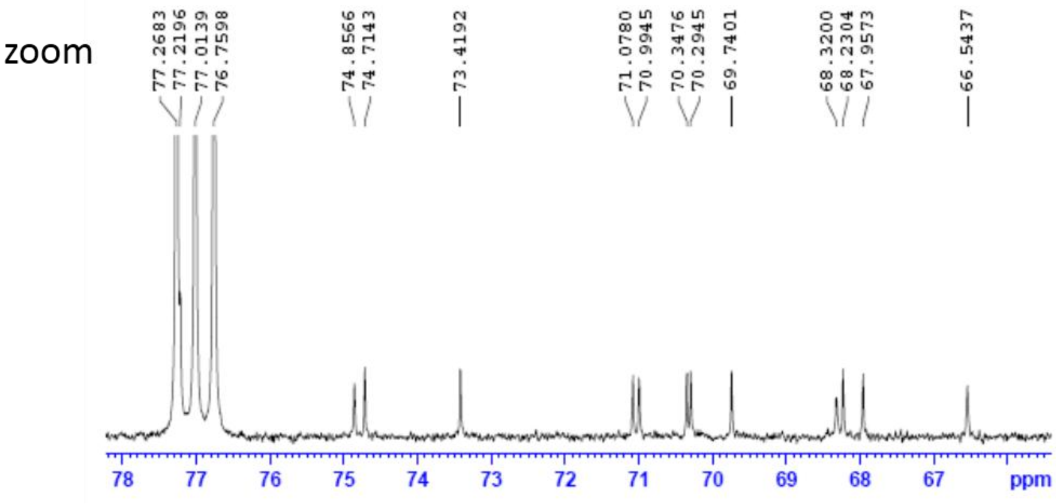

DEPT NMR of 11α in $CDCl_3$

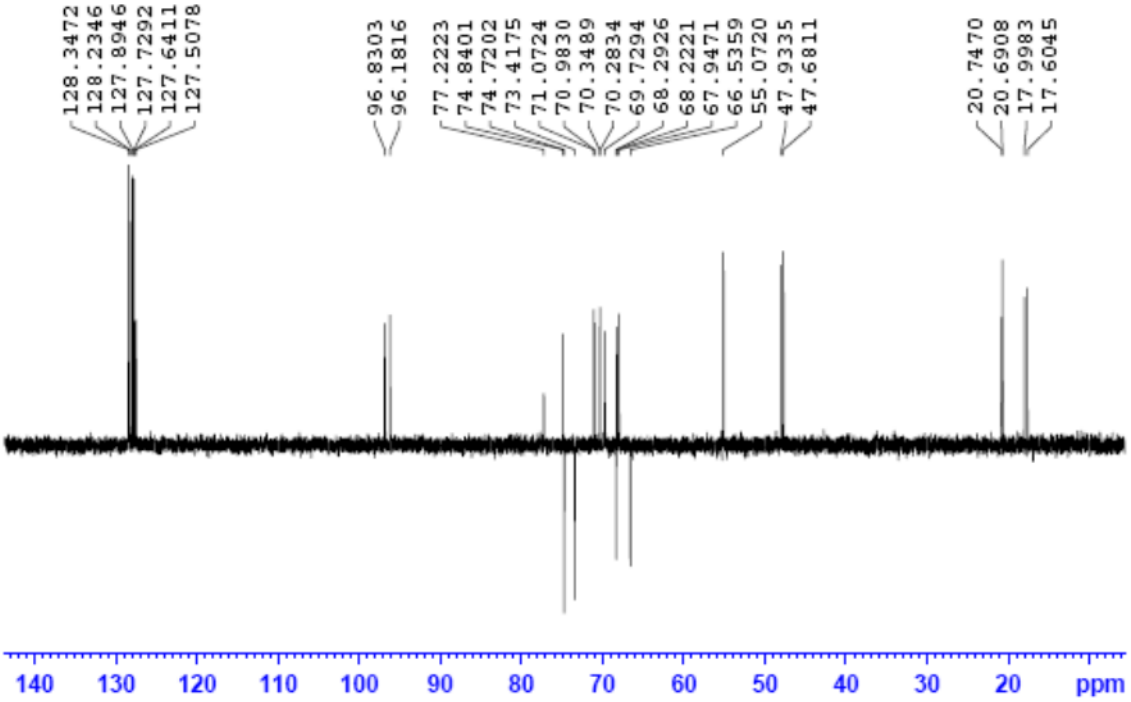

HMBC

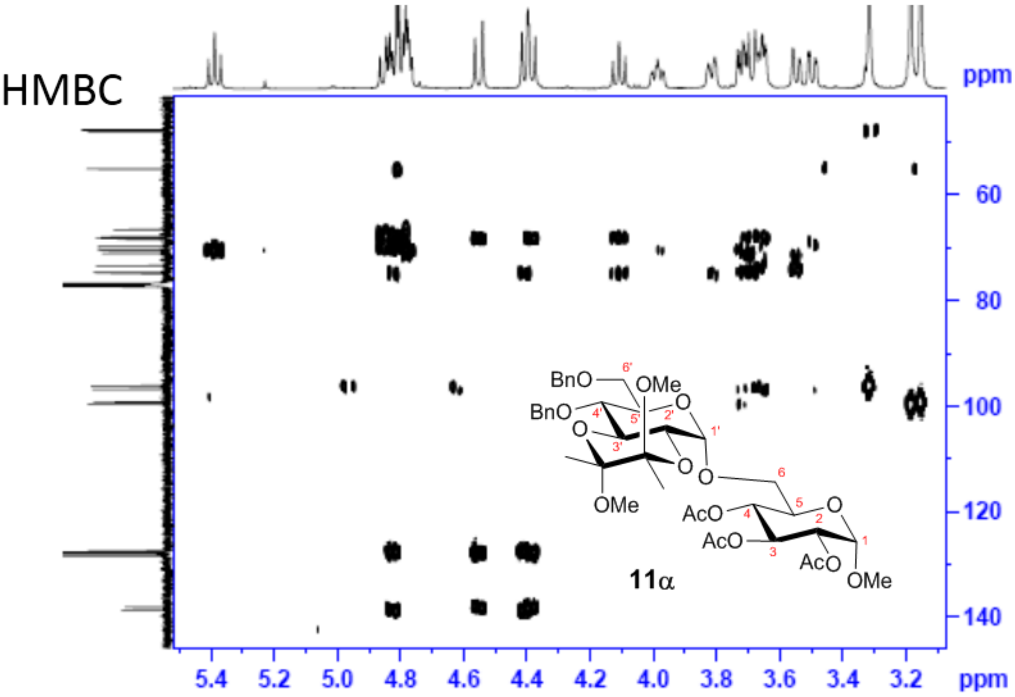

HMQC NMR of 11α in $CDCl_3$

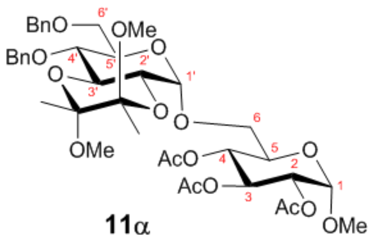

11α

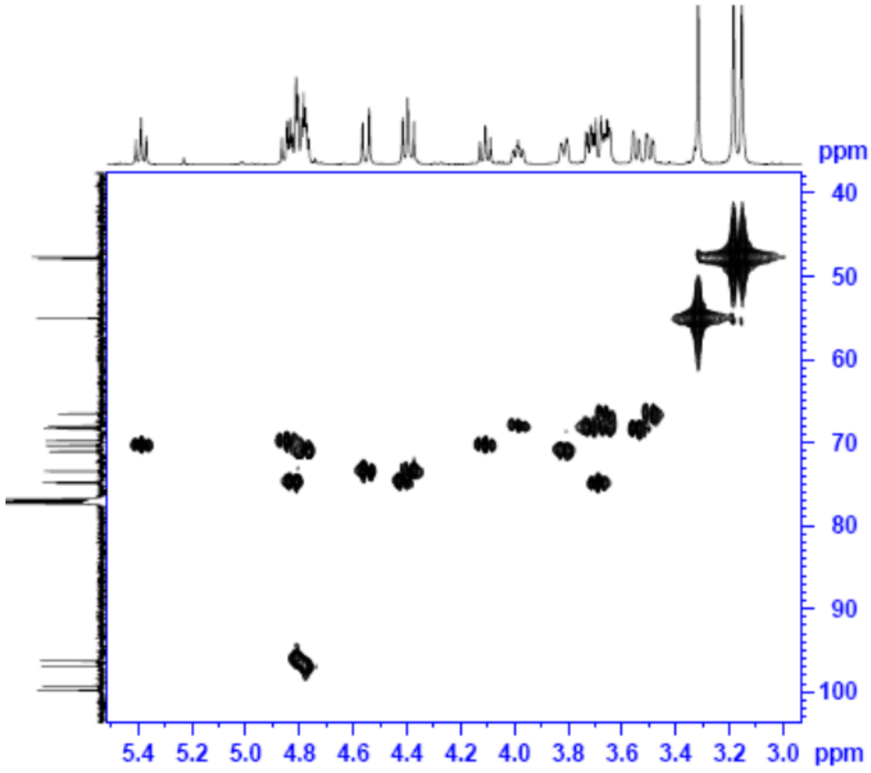

^{1}H NMR of 11β in $CDCl_3$

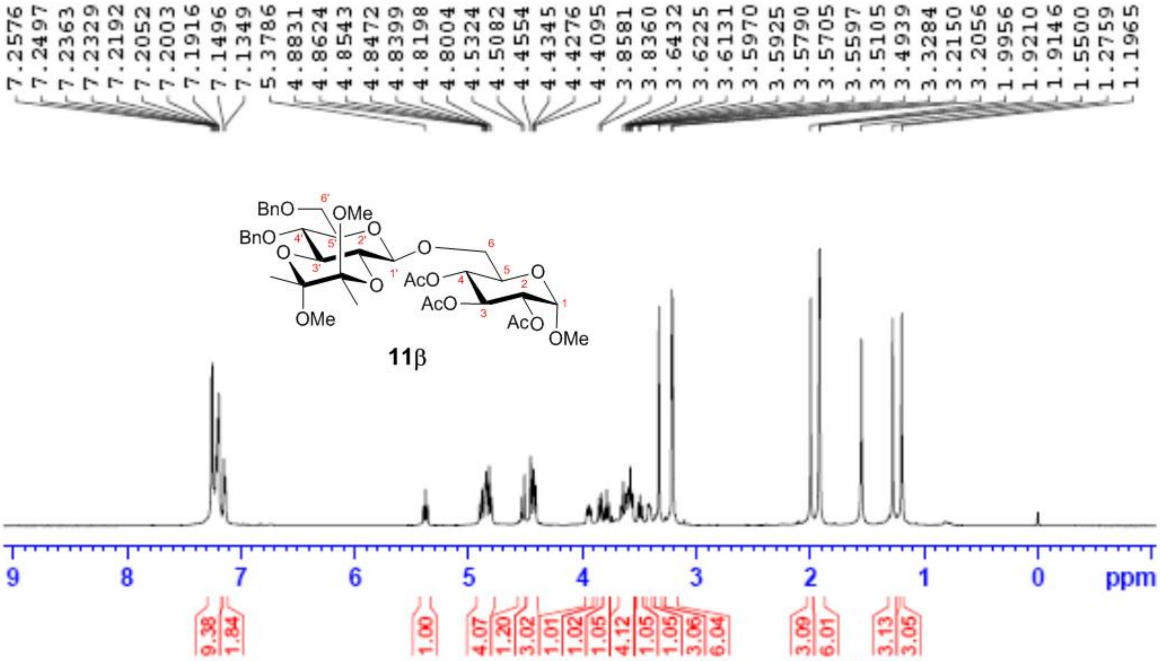

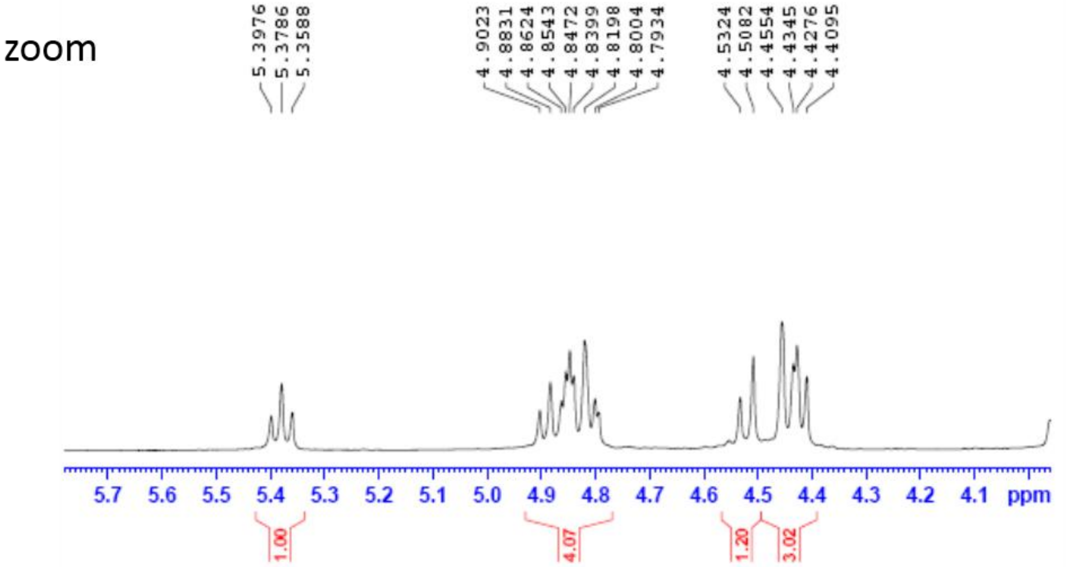

^{1}COSY NMR of 11β in $CDCl_3$

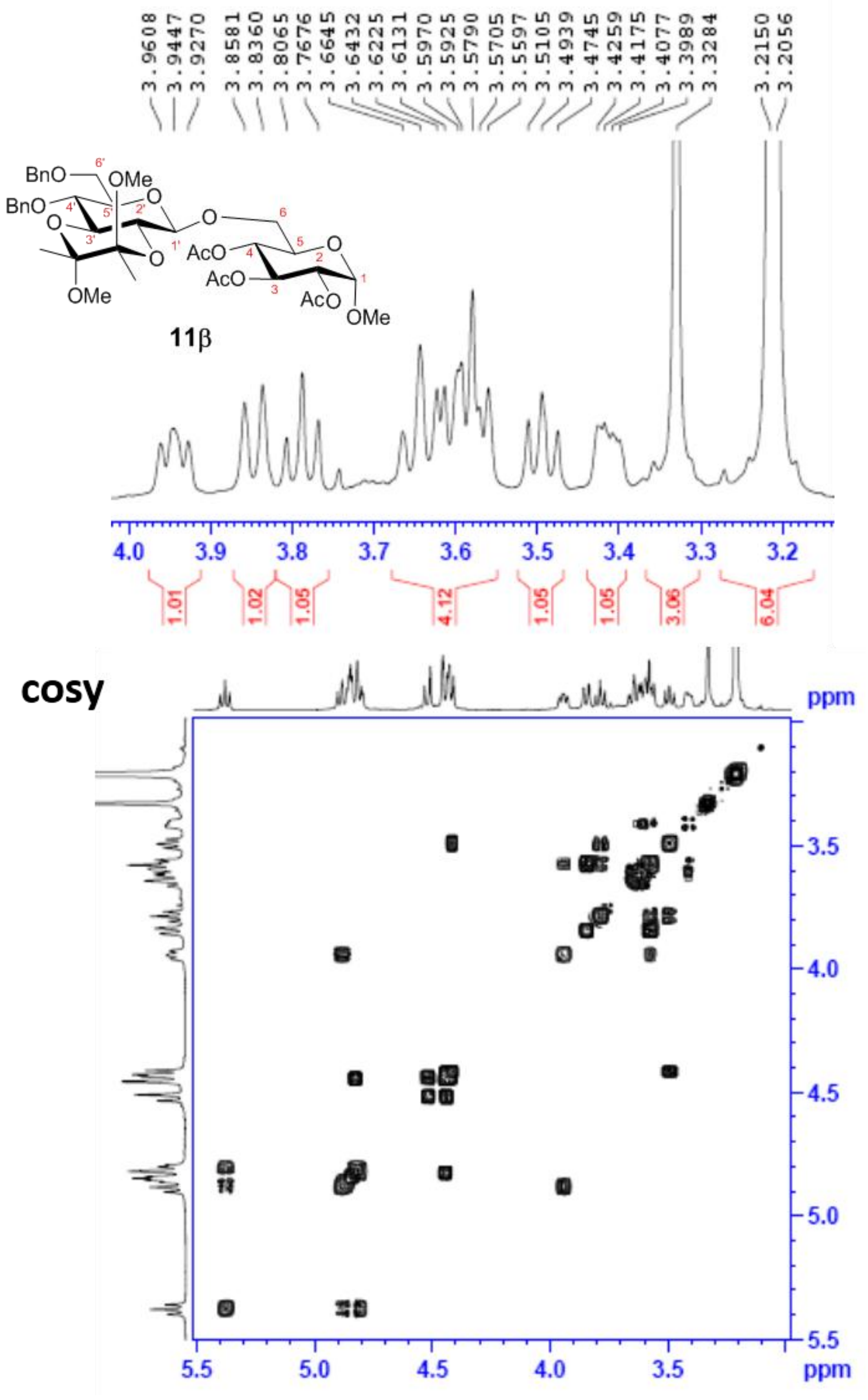

^{13}C NMR of 11β in CDCl$_3$

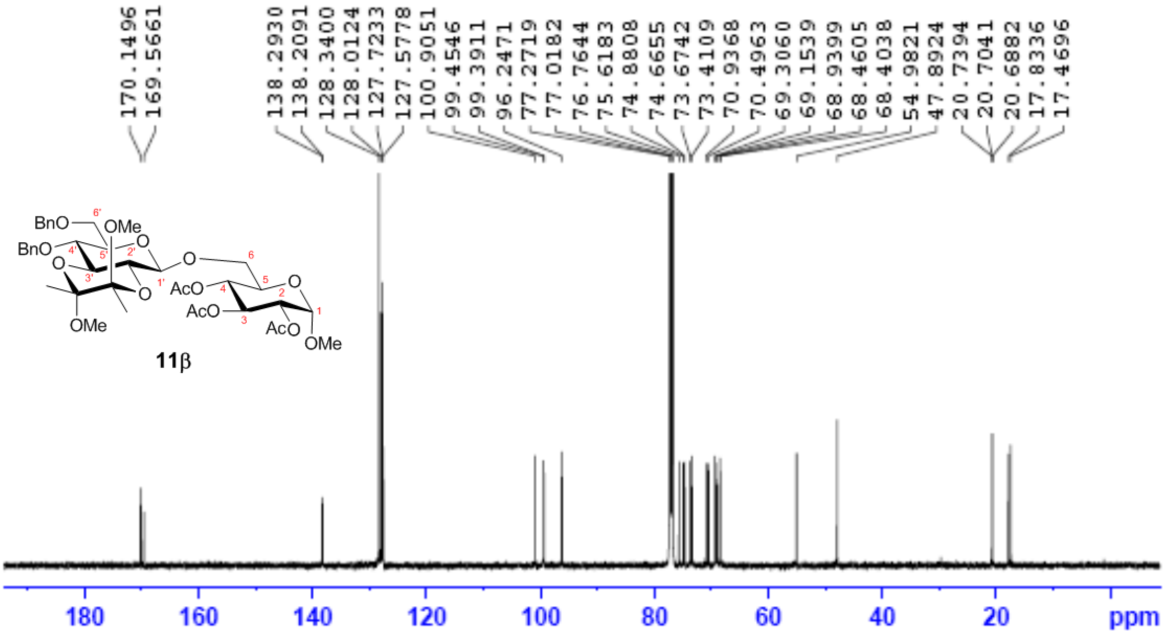

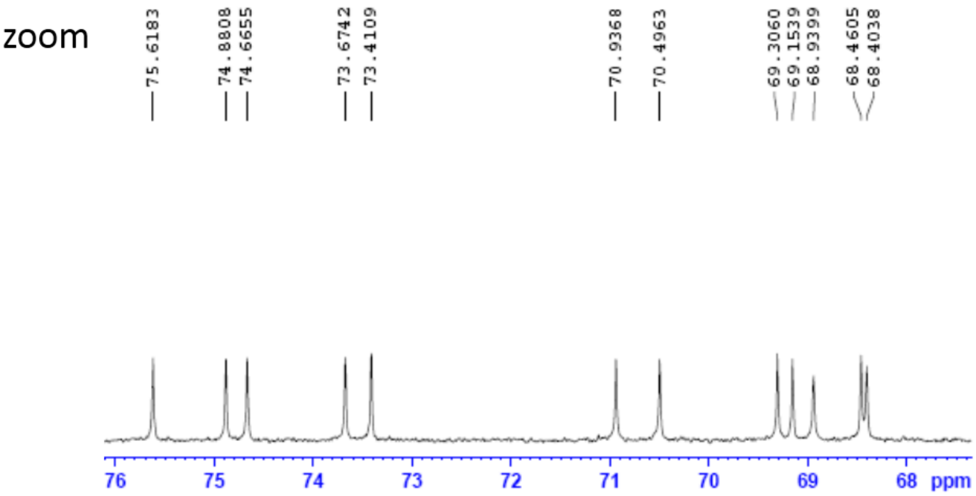

DEPT NMR of 11β in $CDCl_3$

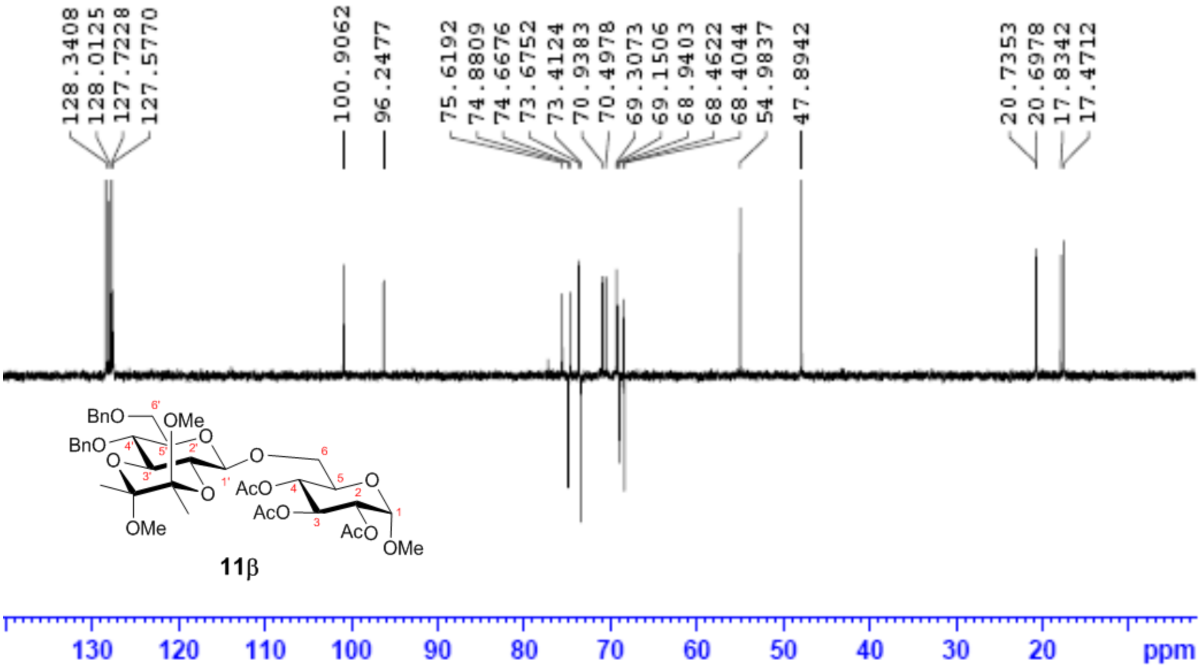

HMBC

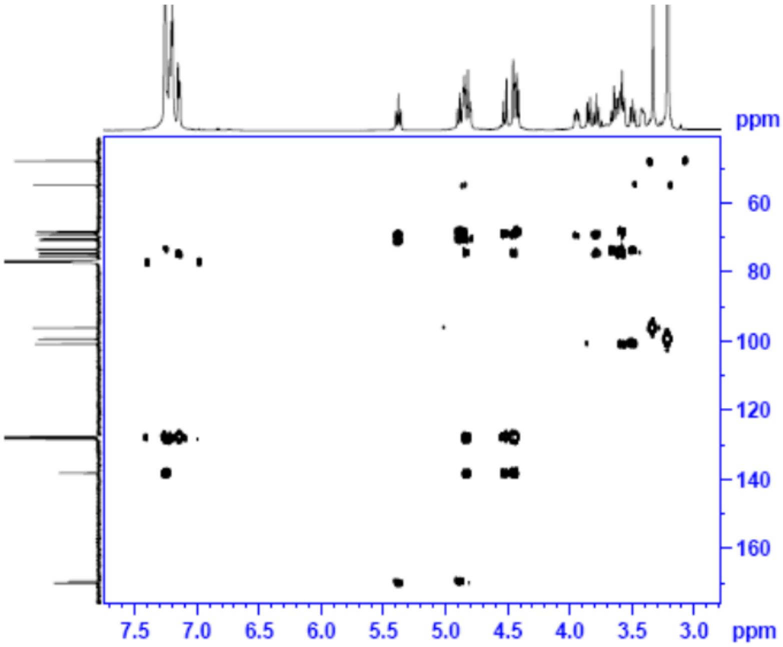

HMQC NMR of 11β in $CDCl_3$

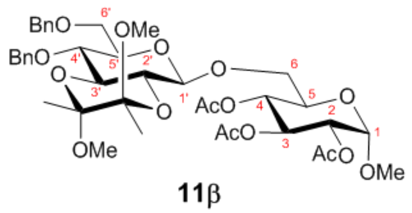

11β

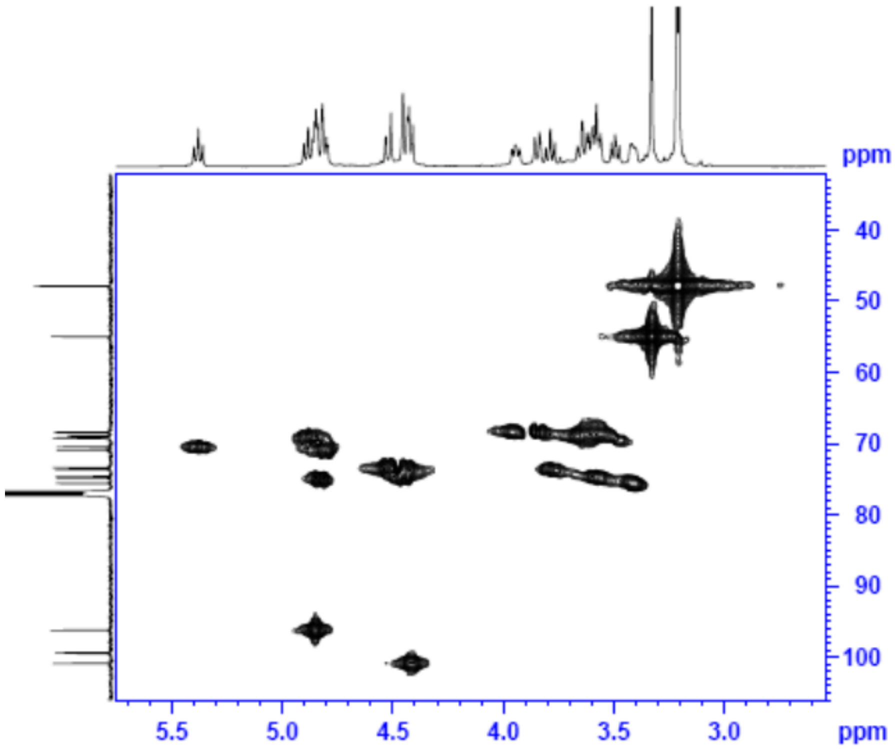

^{1}H NMR of 12α in $CDCl_3$

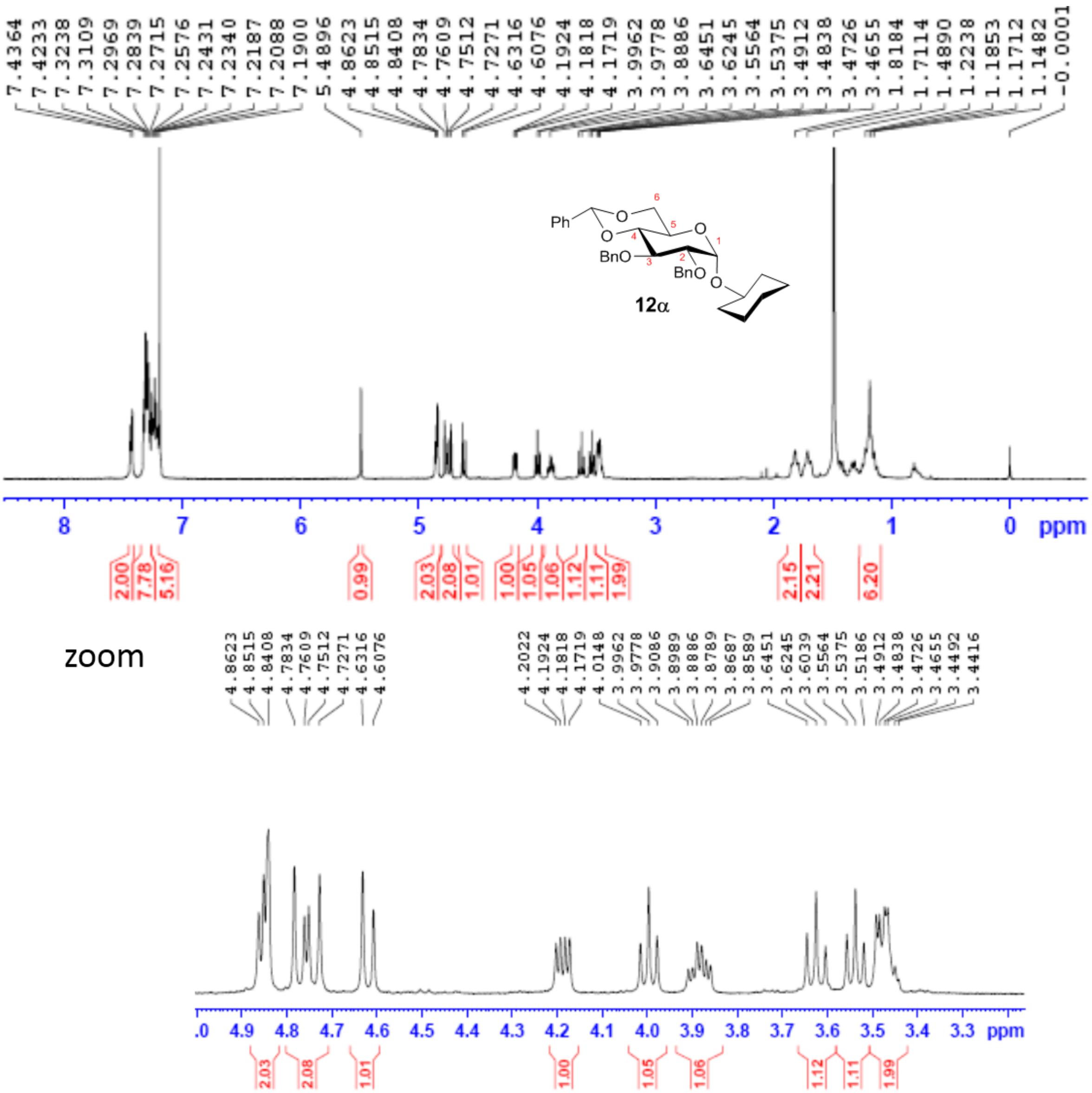

COSY NMR of 12α in $CDCl_3$

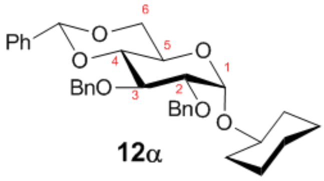

12α

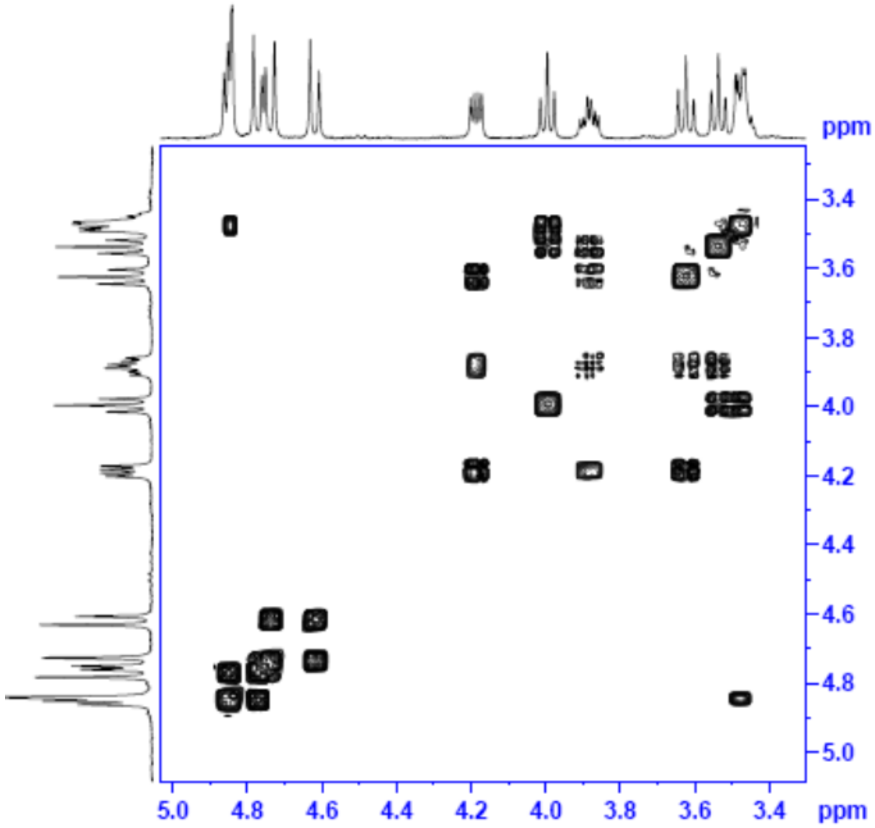

^{13}C NMR of 12*α* in CDCl$_3$

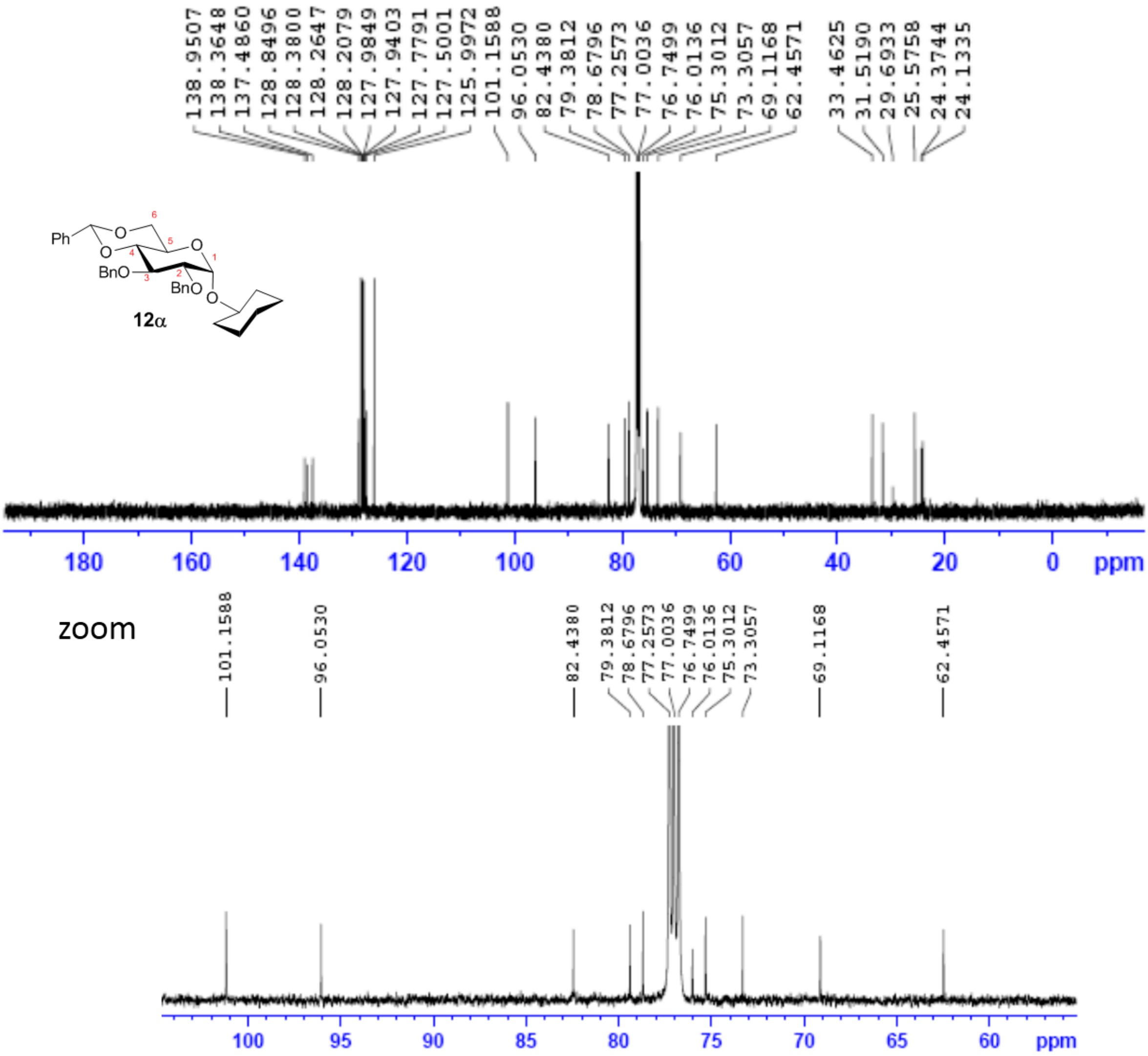

DEPT NMR of 12α in $CDCl_3$

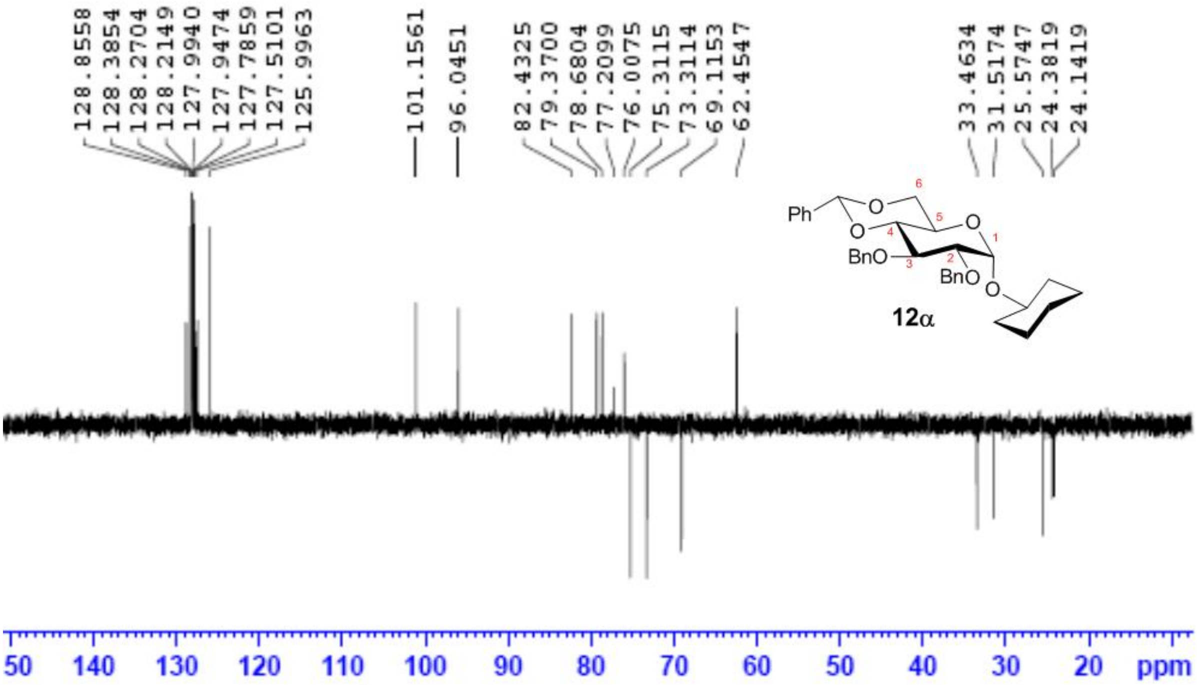

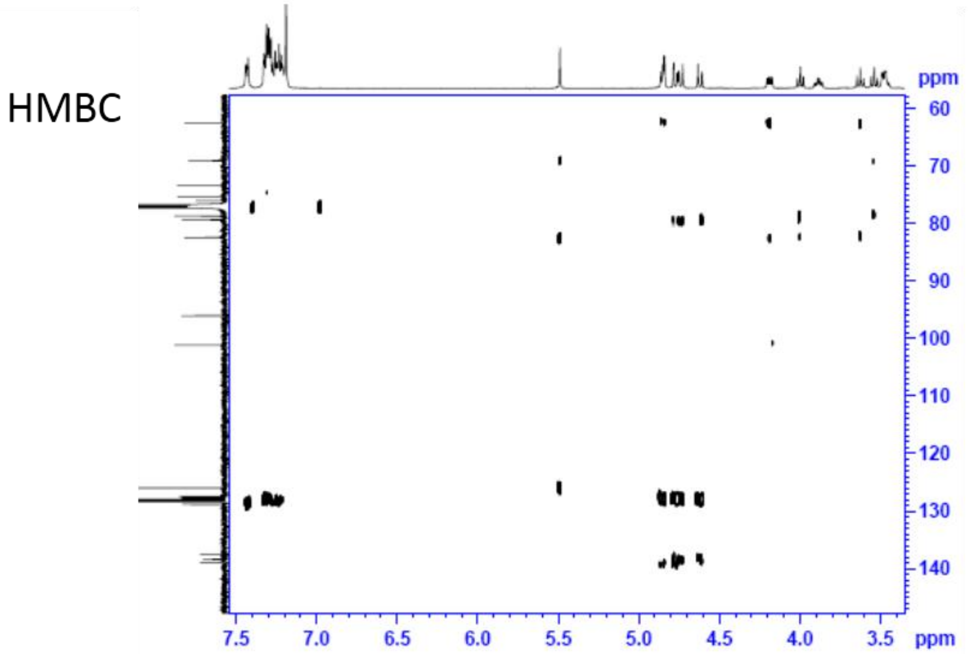

HMQC NMR of 12α in $CDCl_3$

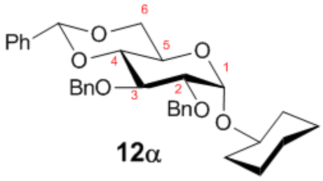

12α

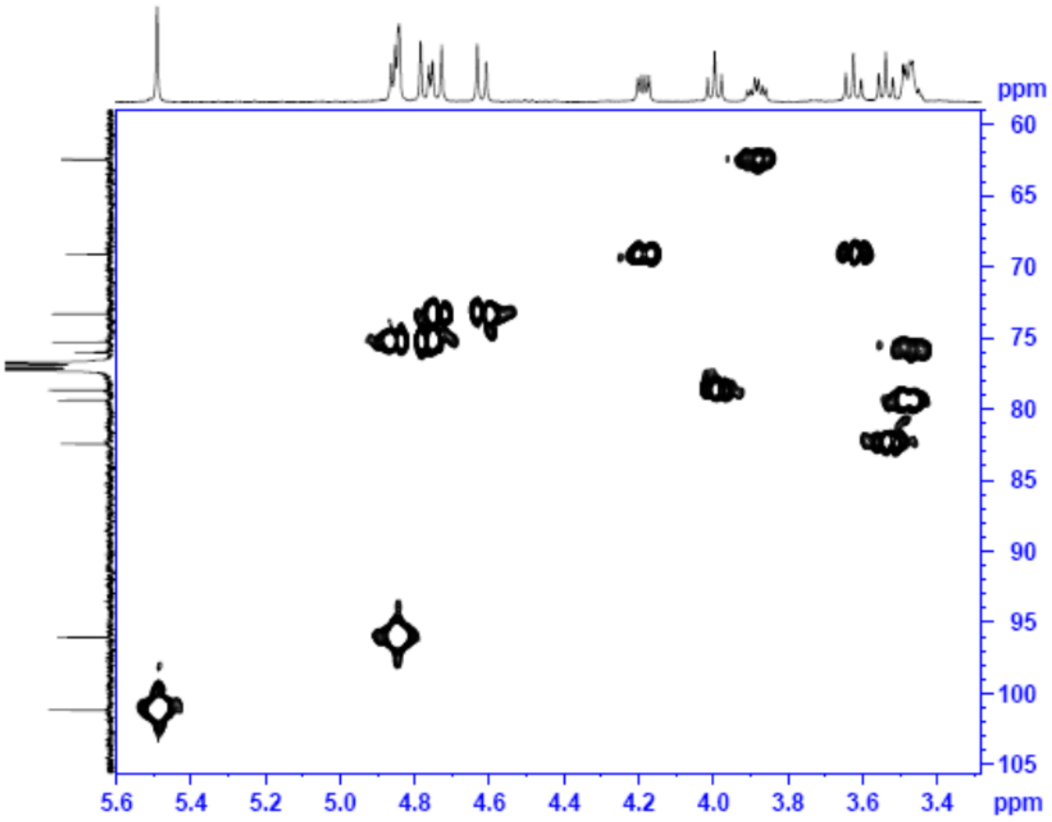

^{1}H NMR of 12β in $CDCl_3$

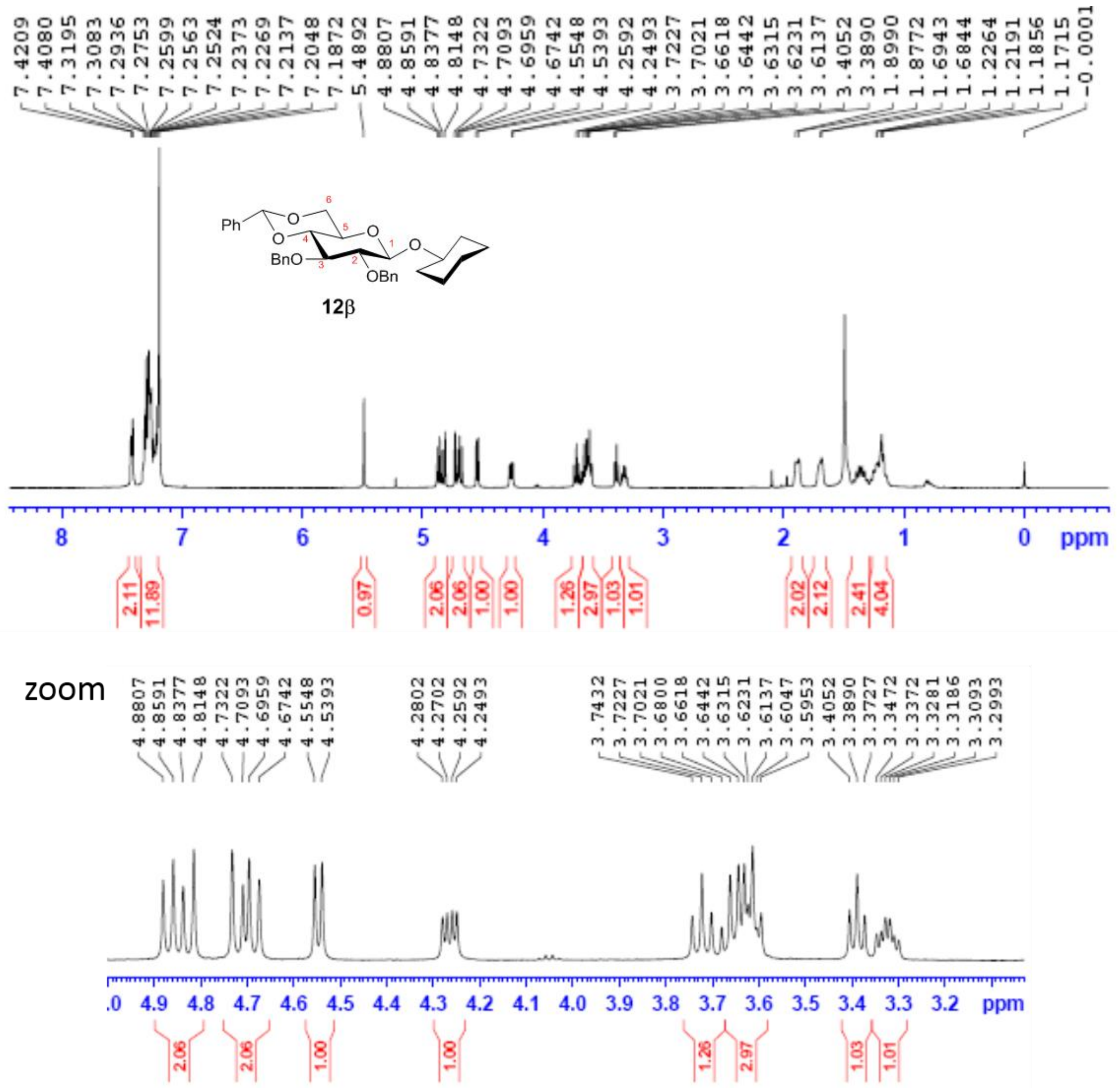

COSY NMR of 12β in $CDCl_3$

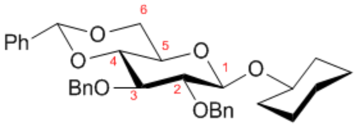

12β

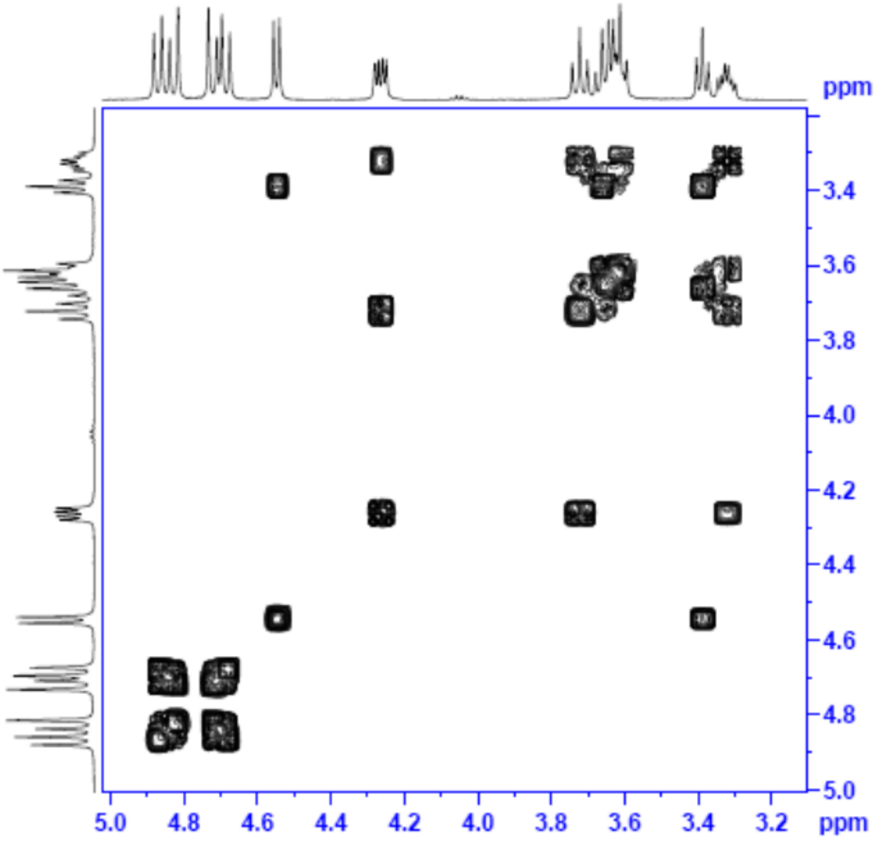

^{13}C NMR of 12β in $CDCl_3$

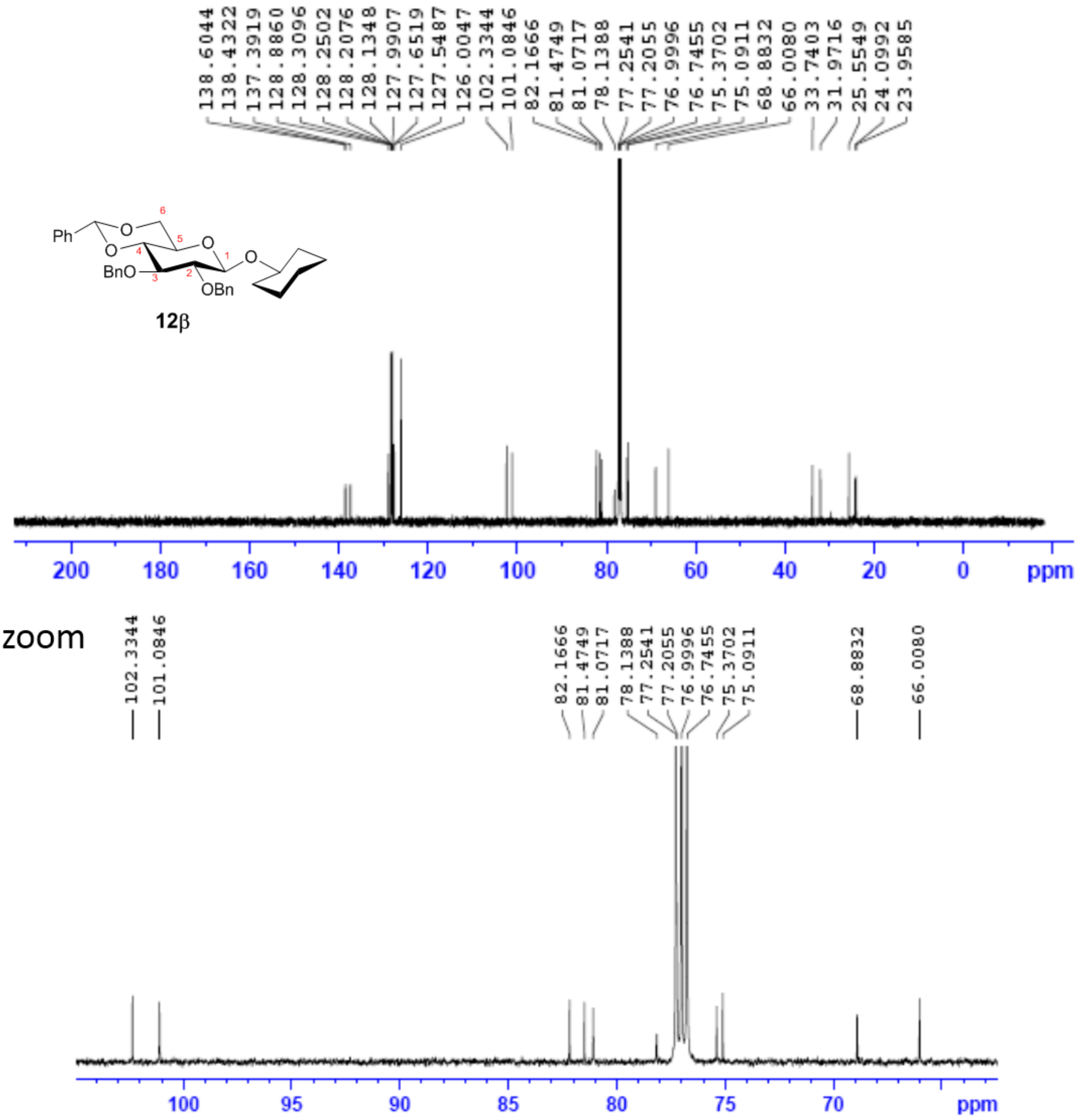

DEPT NMR of 12β in $CDCl_3$

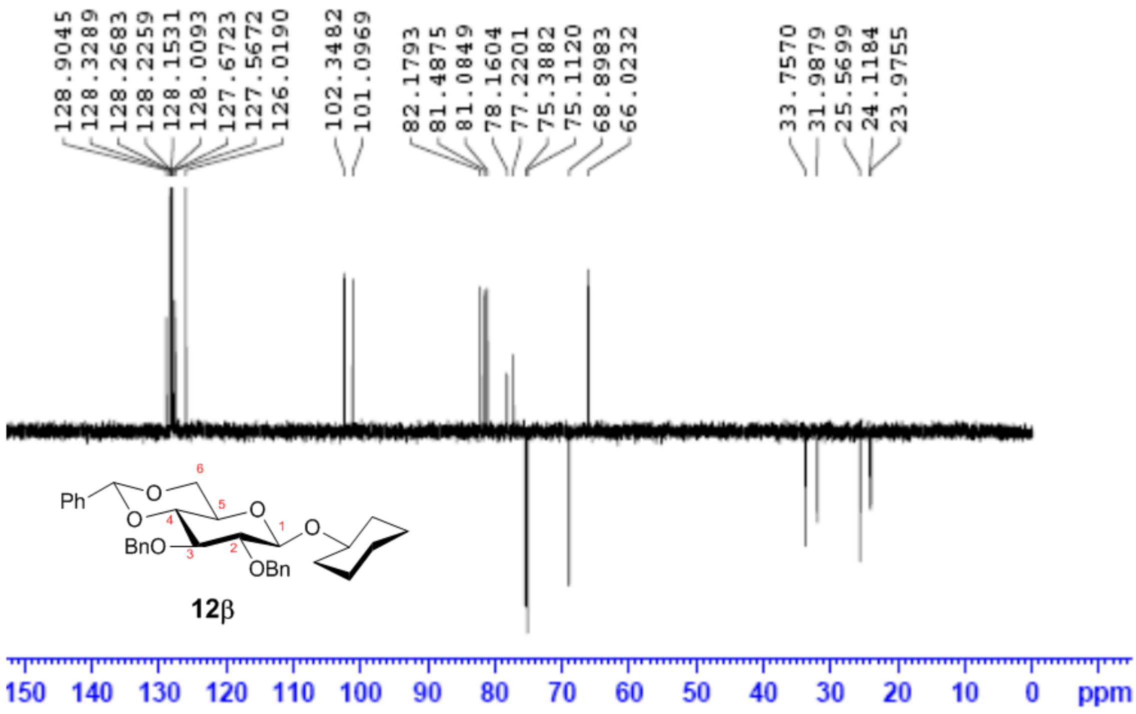

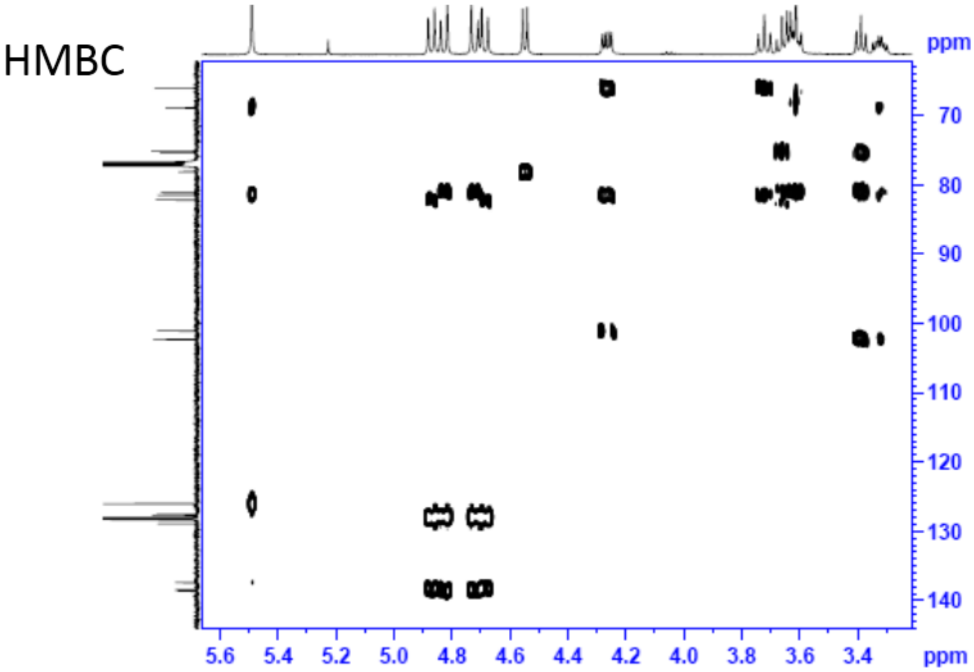

HMQC NMR of 12β in $CDCl_3$

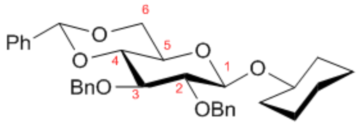

12β

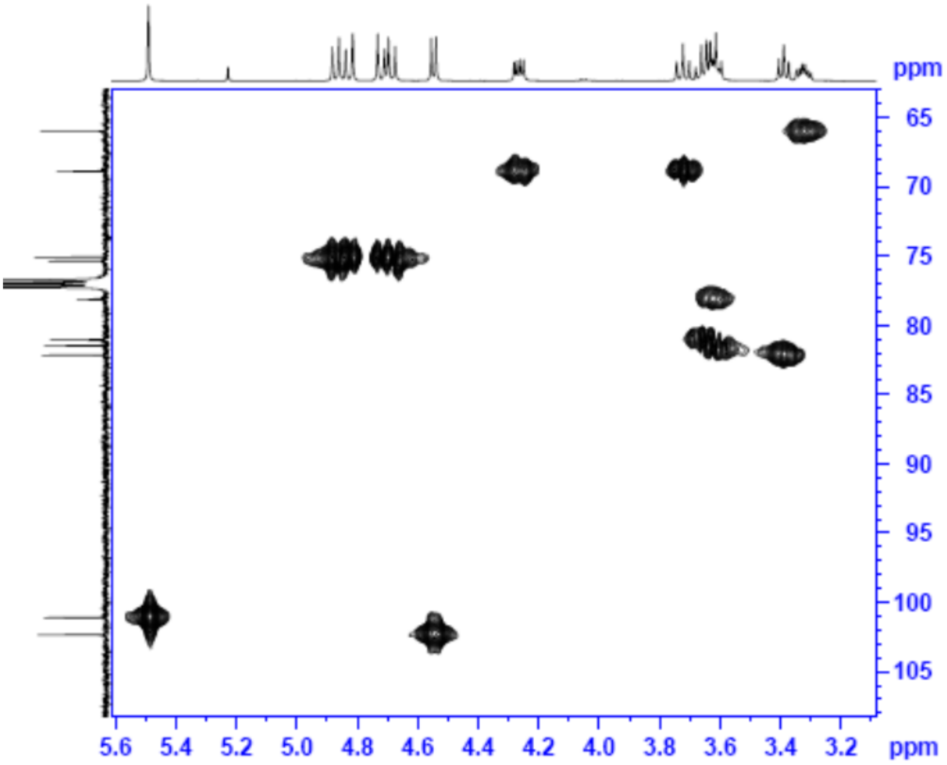

^{1}H NMR of 13α in $CDCl_3$

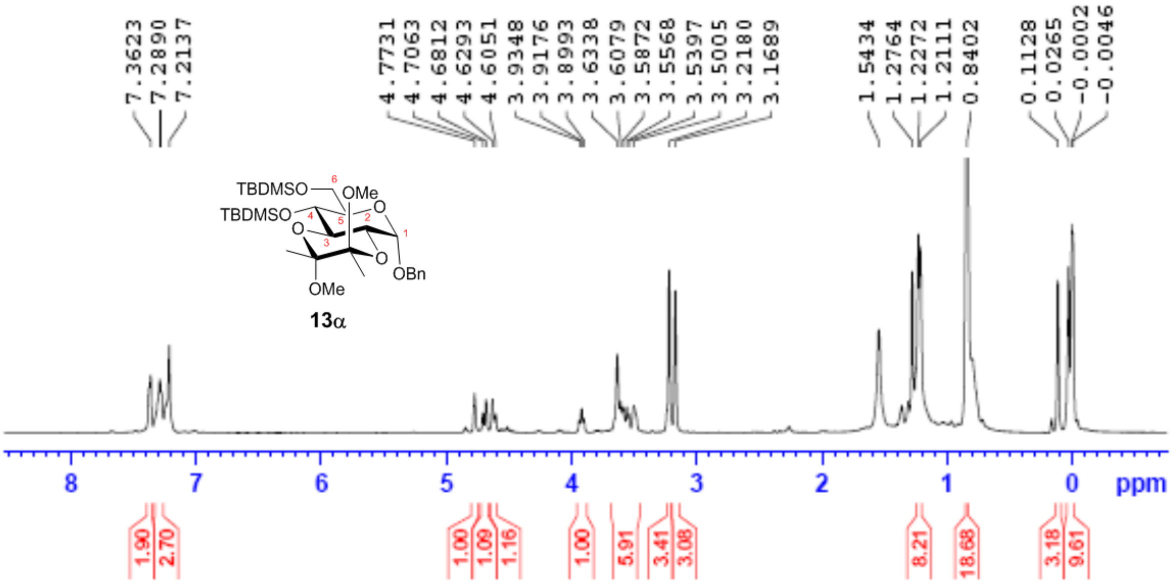

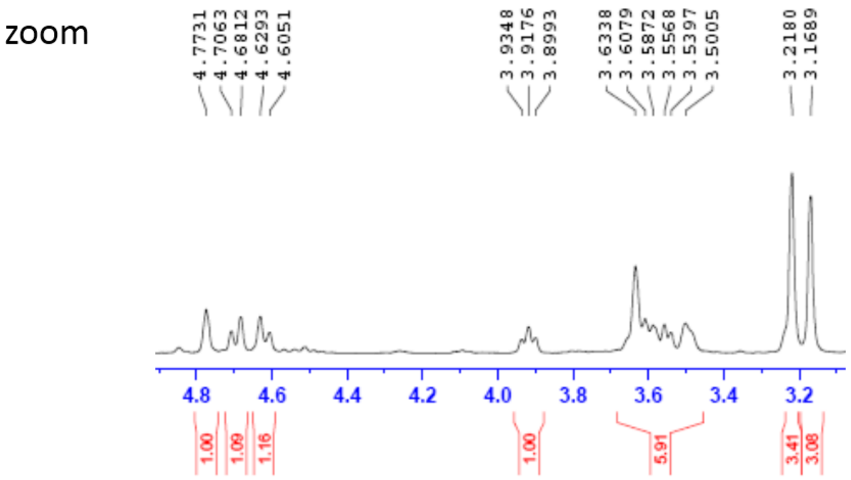

COSY NMR of 13α in $CDCl_3$

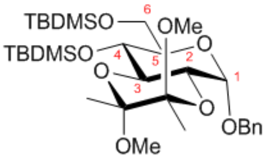

13α

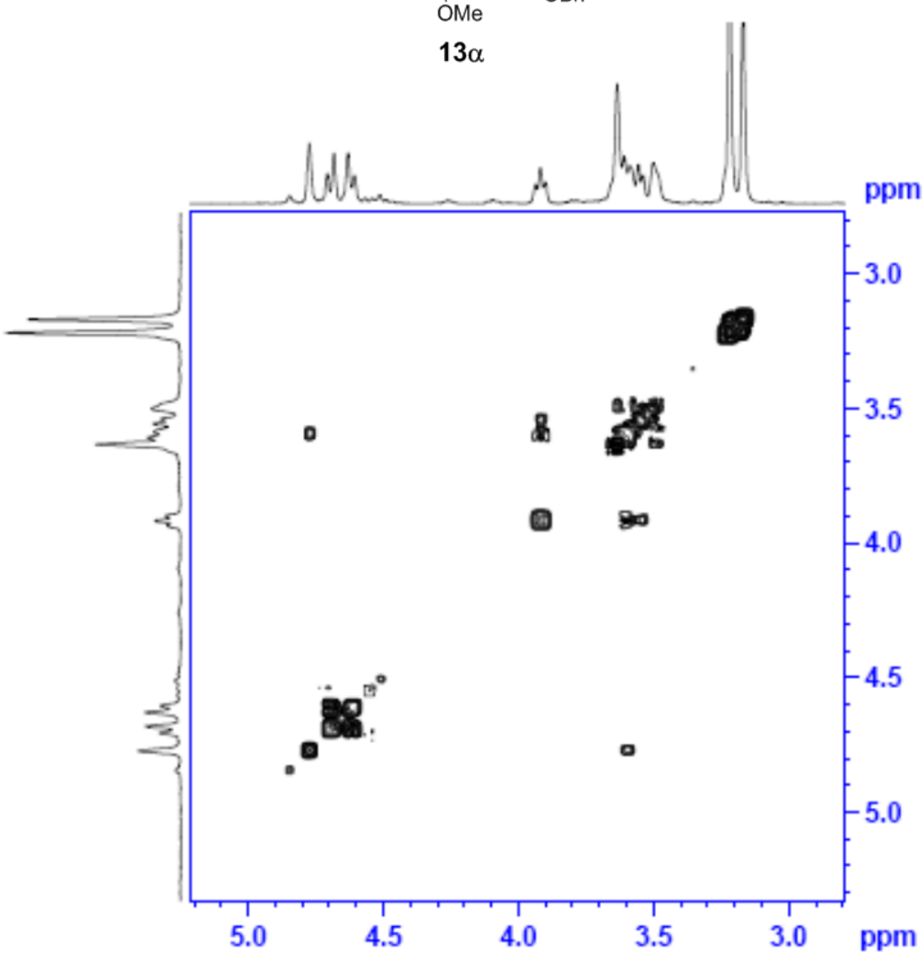

^{13}C NMR of 13α in $CDCl_3$

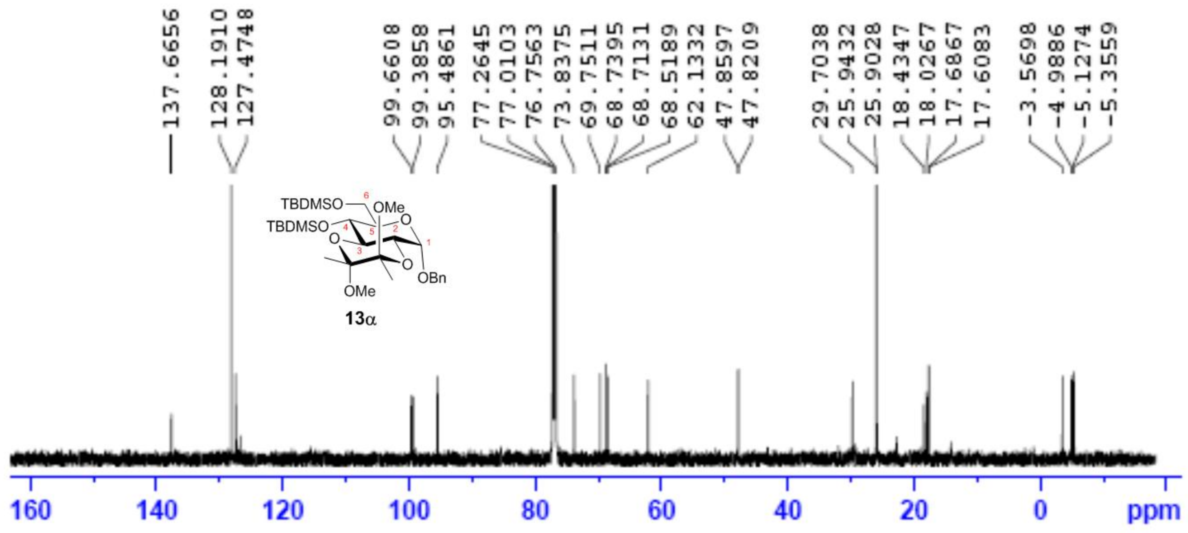

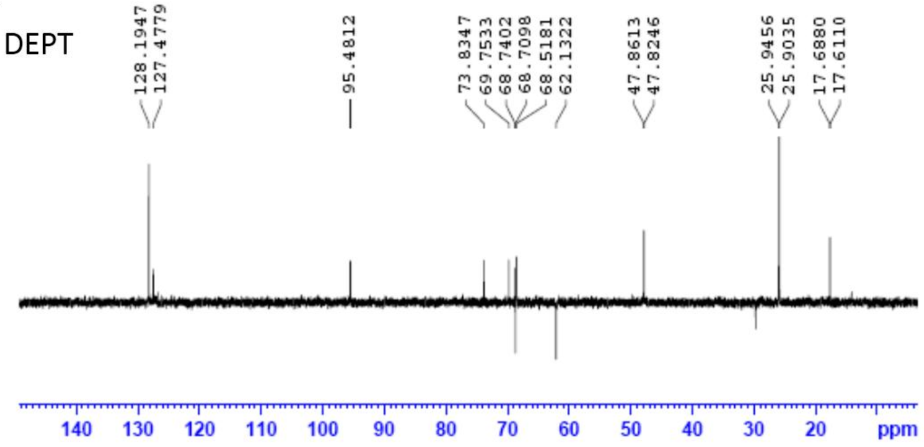

HMBC NMR of 13α in $CDCl_3$

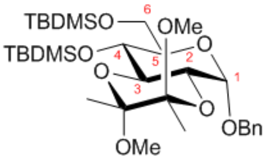

13α

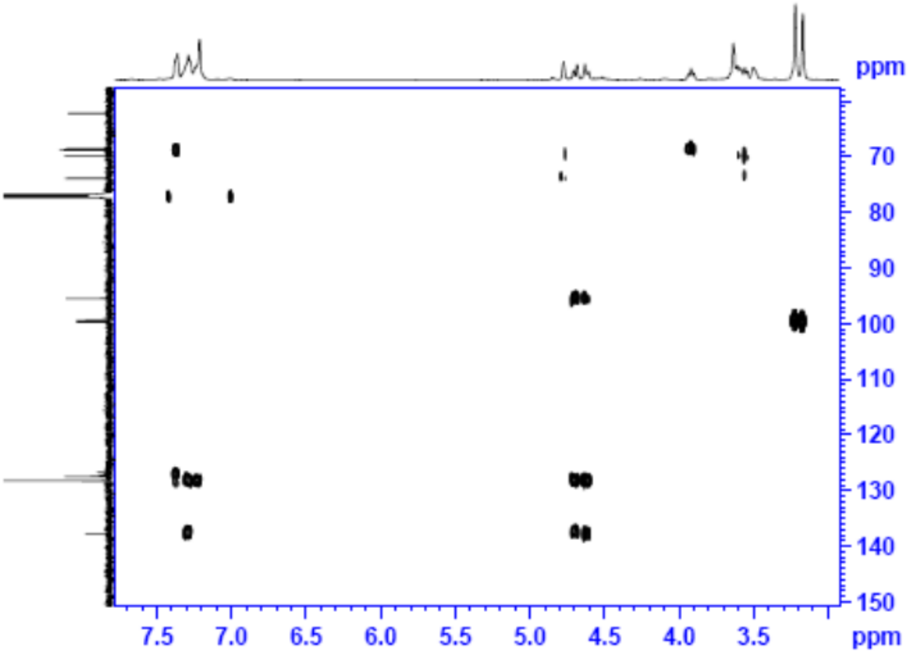

HMQC NMR of 13α in $CDCl_3$

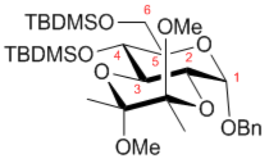

13α

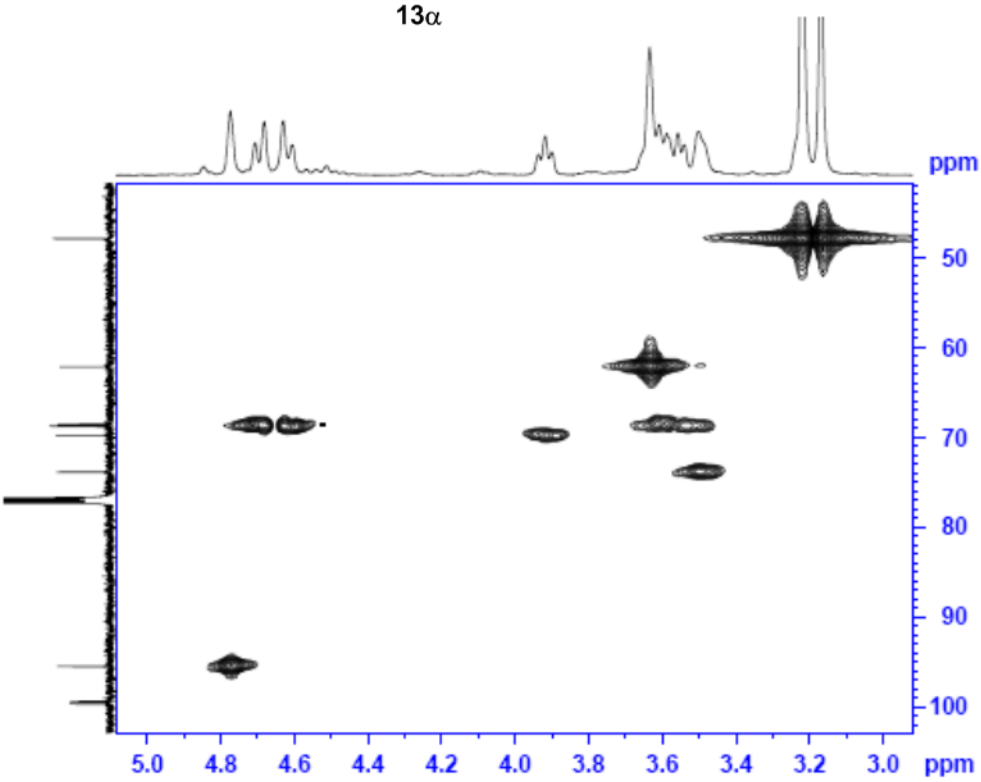

^{1}H NMR of 14αβ in DMSO-d$_6$

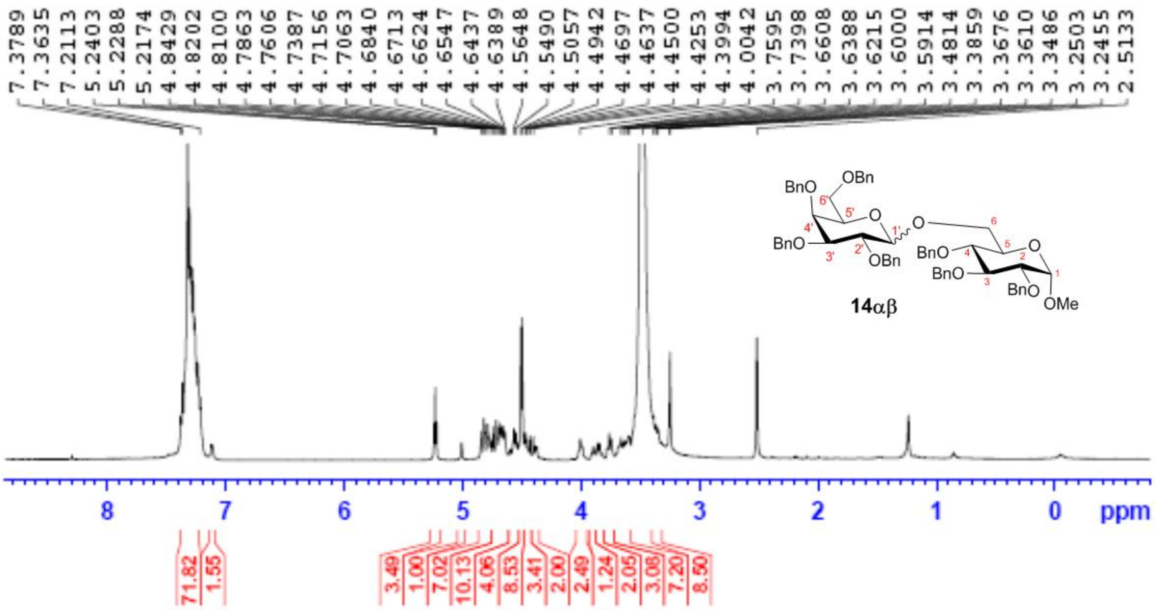

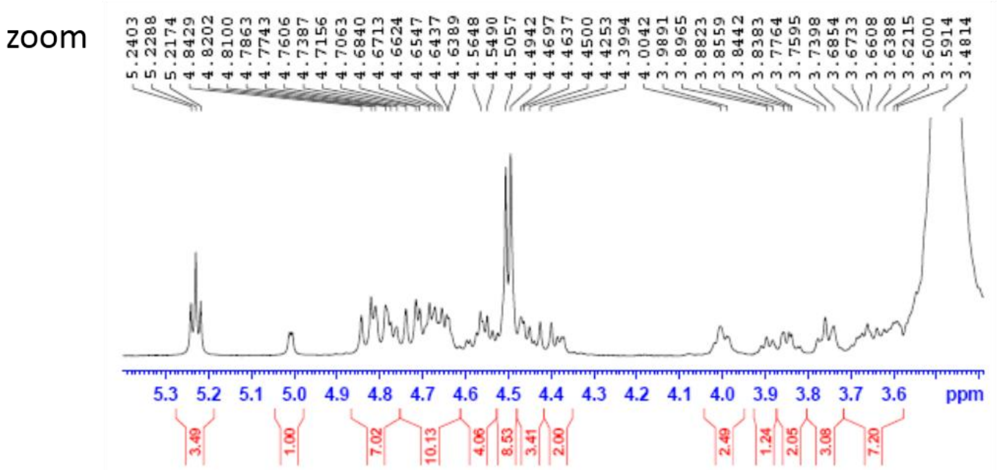

COSY NMR of 14αβ in DMSO-d_6

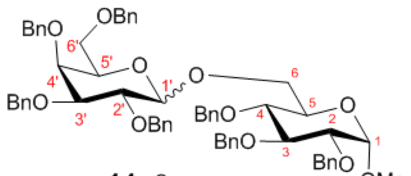

14αβ

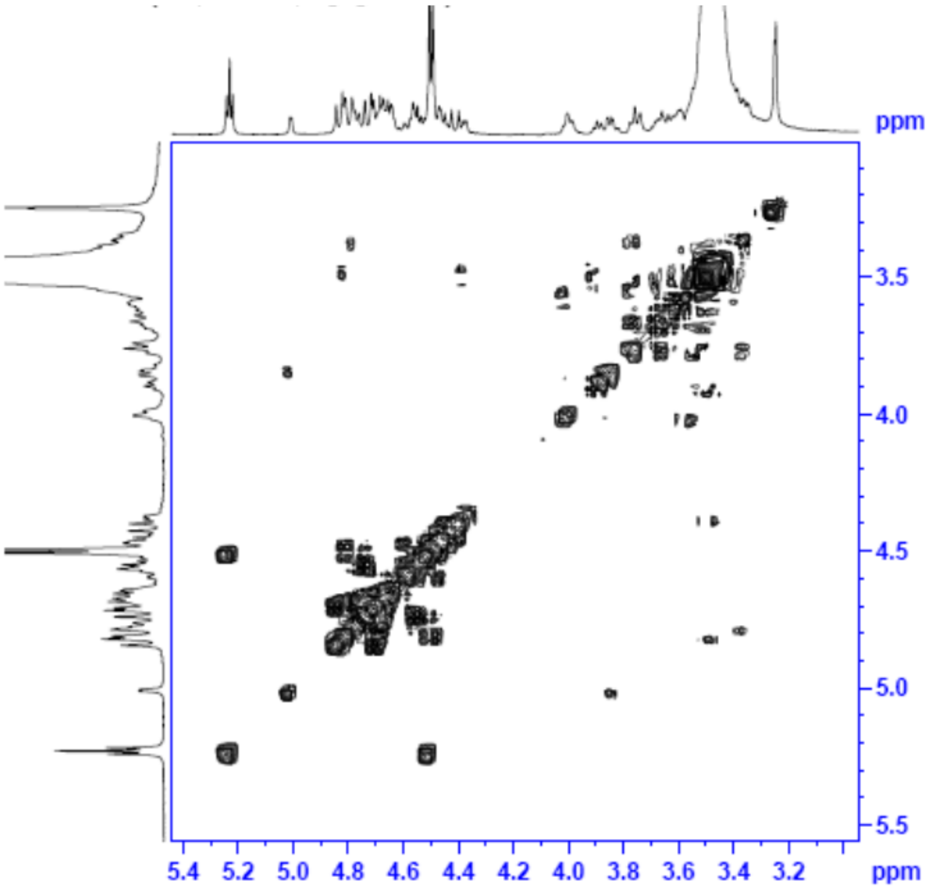

^{13}C NMR of 14αβ in DMSO-d$_6$

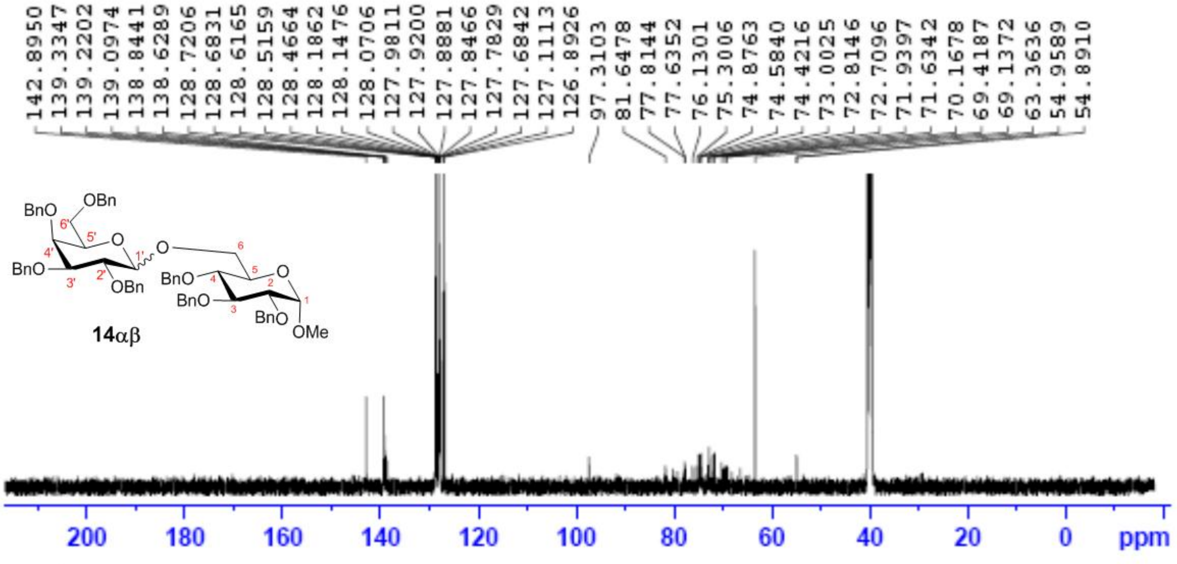

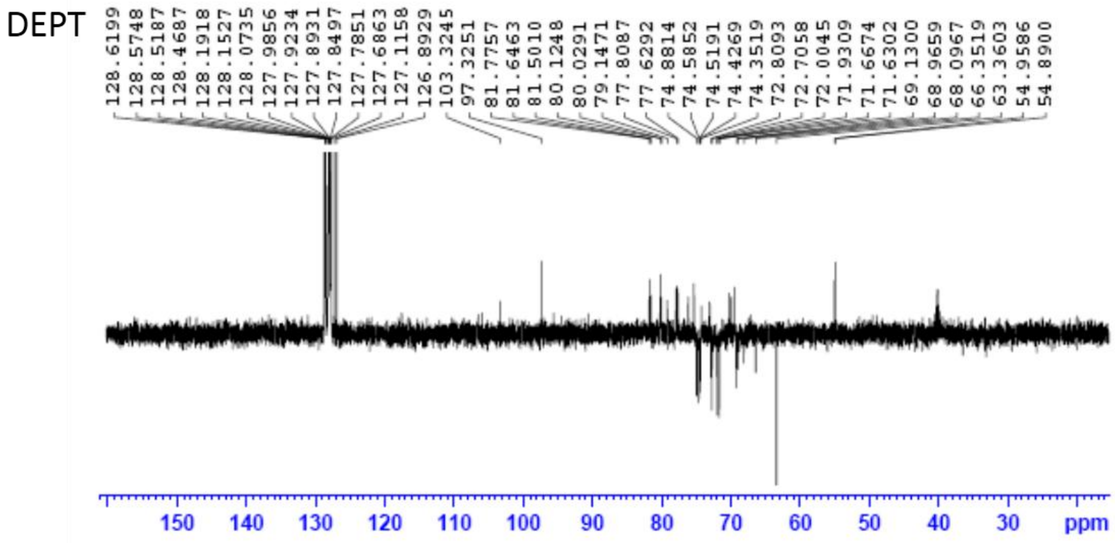

HMQC NMR of 14αβ in DMSO-d_6

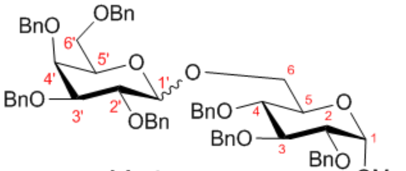

14αβ

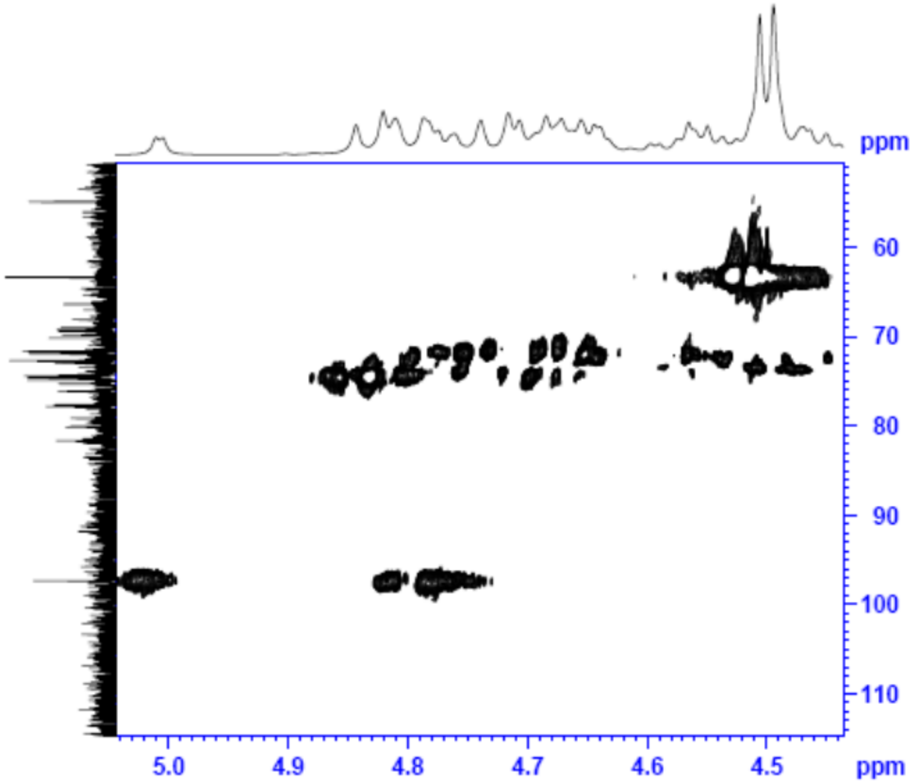

^{1}H NMR of 15αβ in DMSO-d$_6$

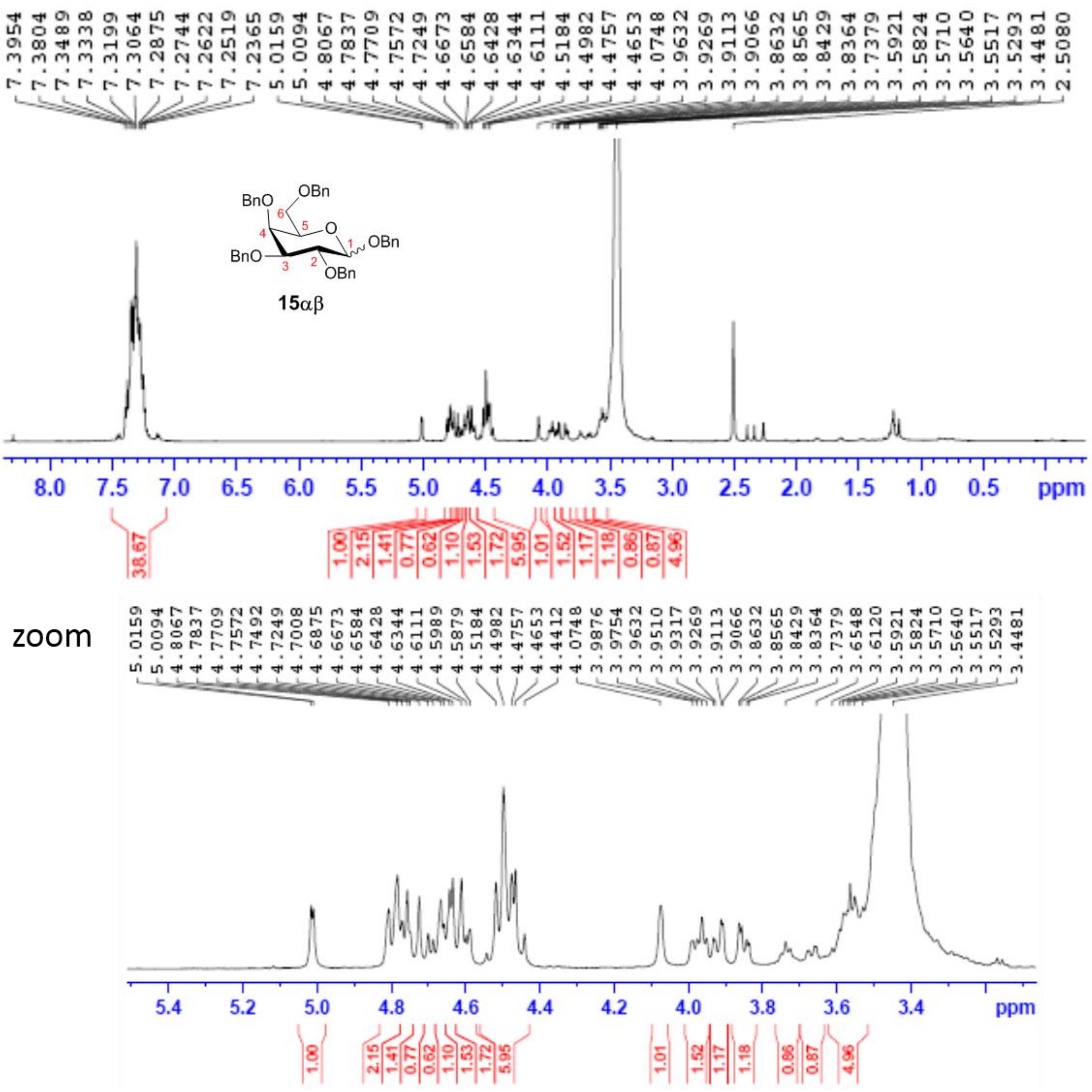

COSY NMR of 15αβ in DMSO-d$_6$

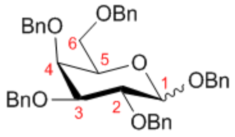

15αβ

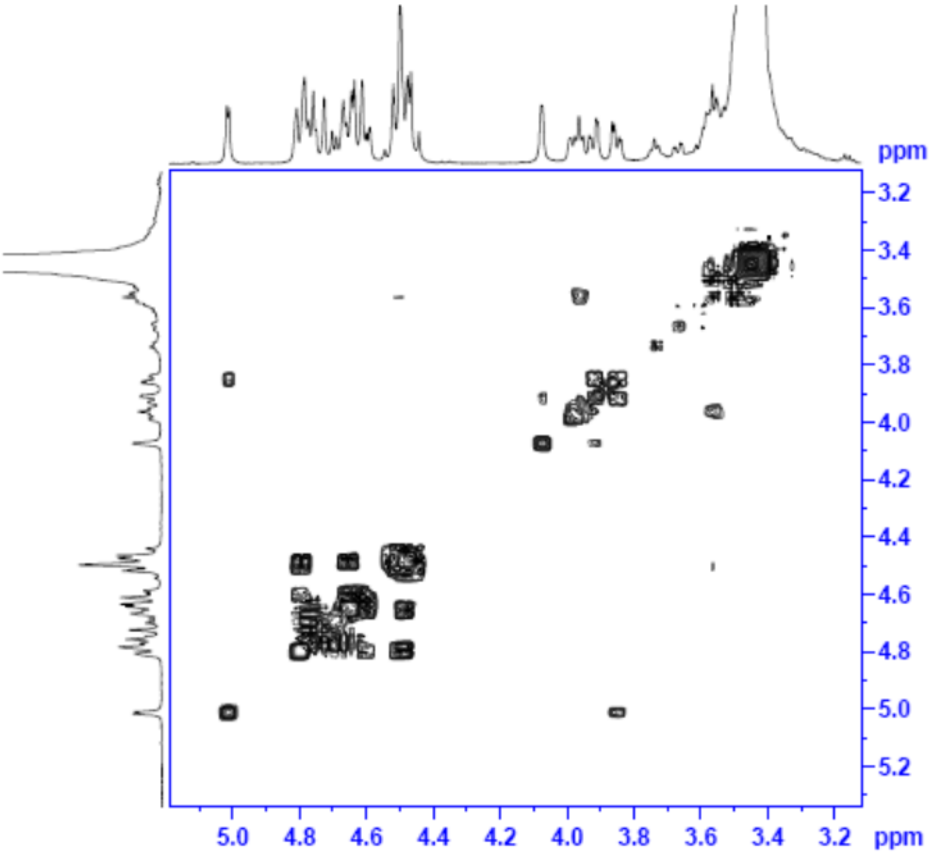

^{13}C NMR of 15αβ in DMSO-d$_6$

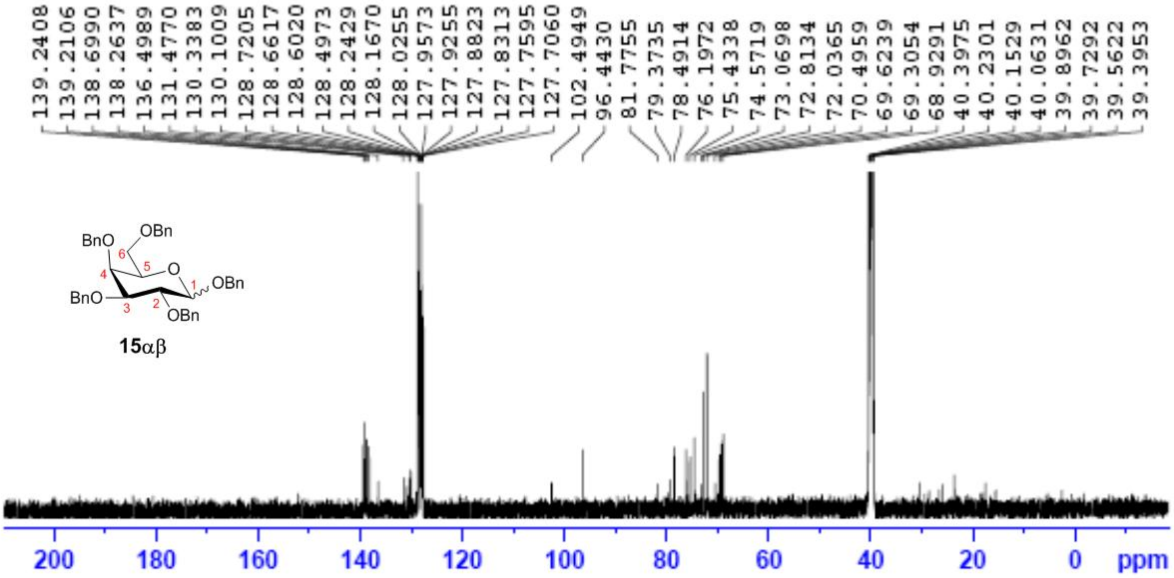

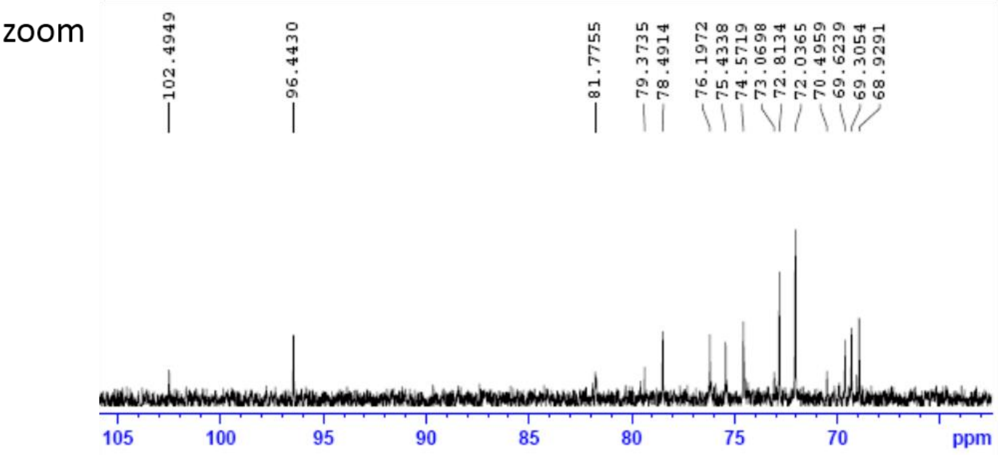

DEPT NMR of 15αβ in DMSO-d_6

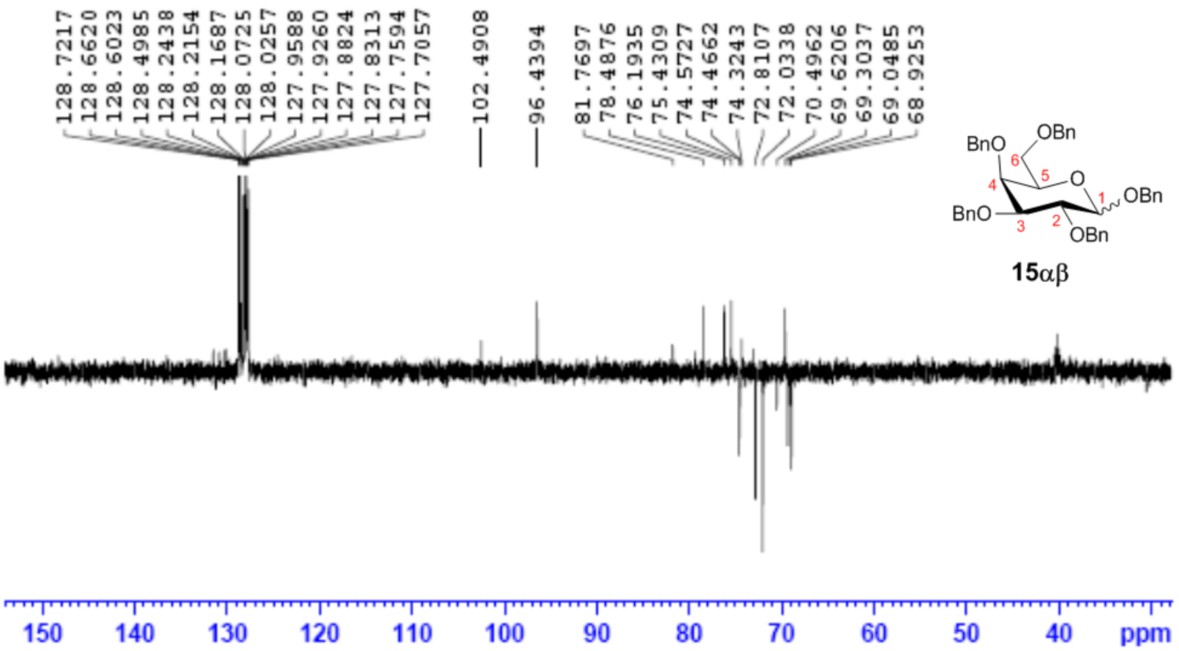

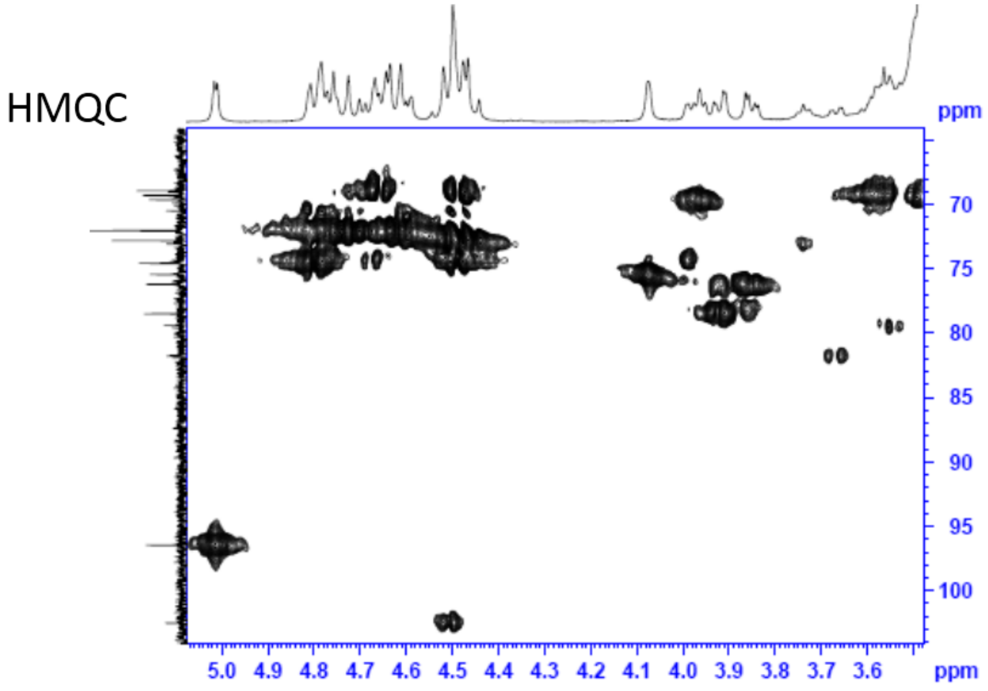

^{1}H NMR of 16αβ in $CDCl_3$

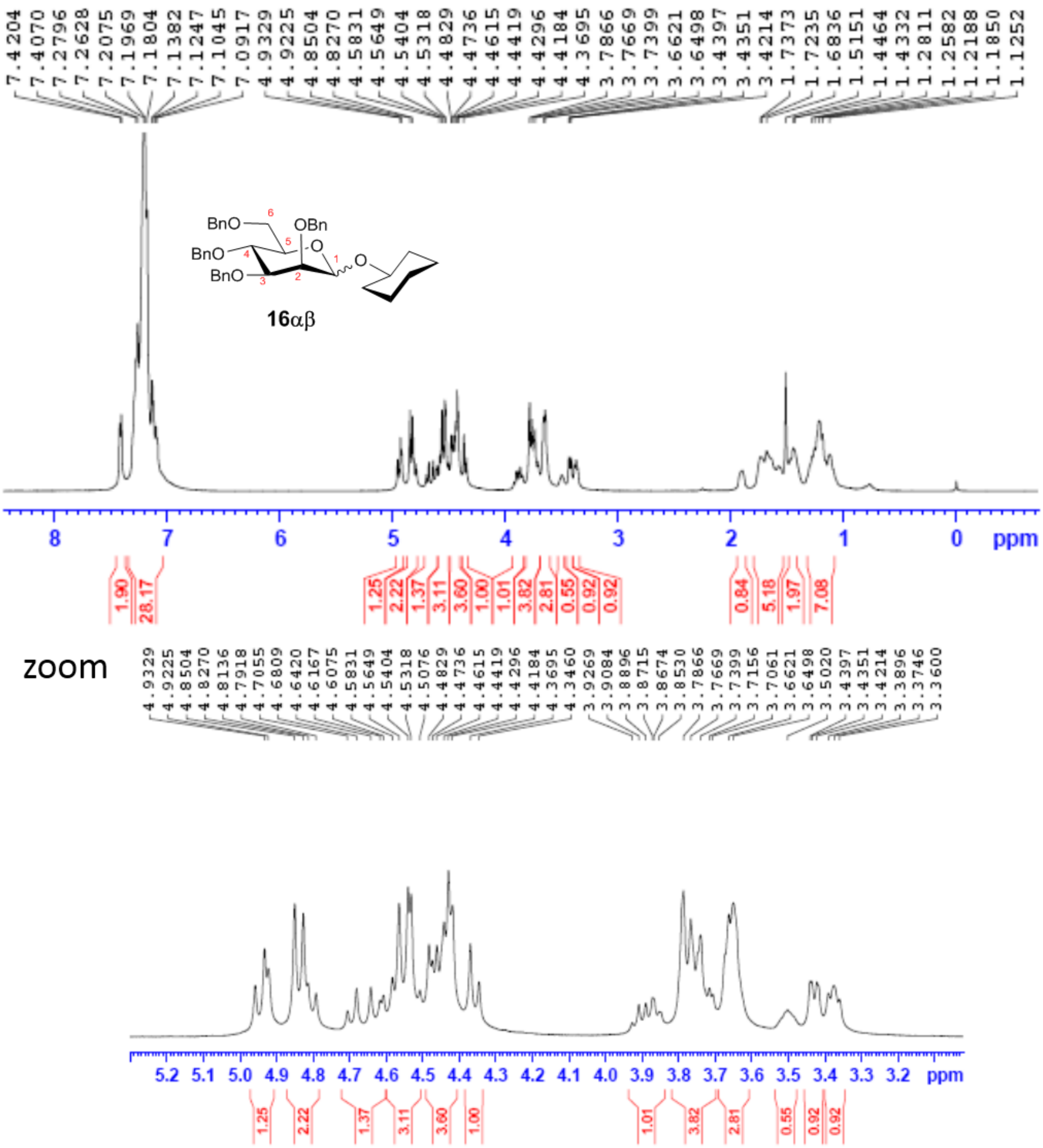

COSY NMR of 16αβ in $CDCl_3$

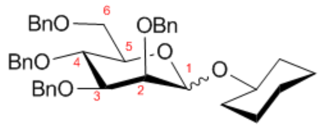

16αβ

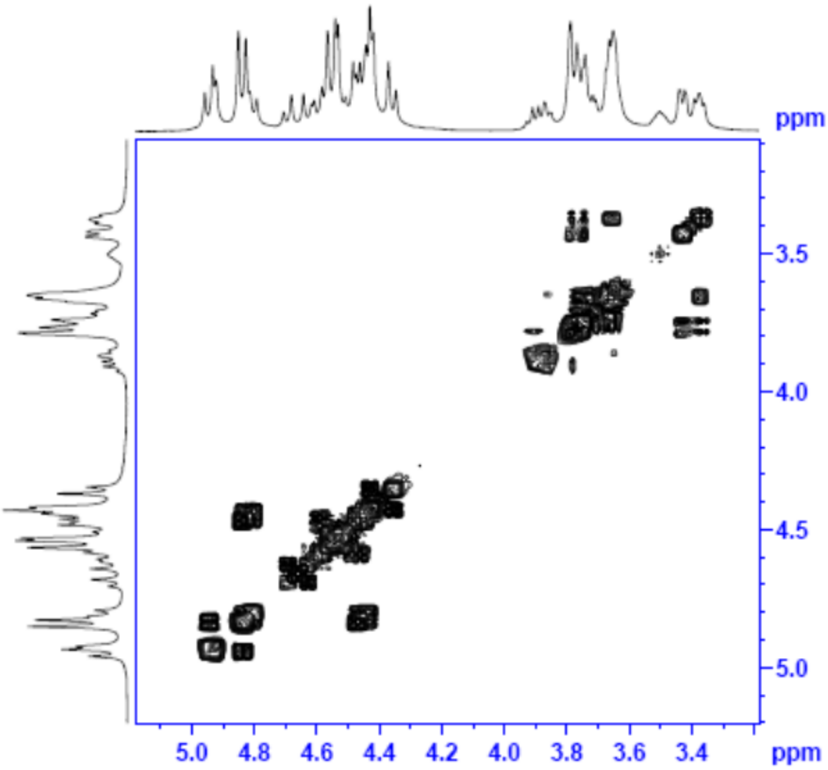

^{13}C NMR of 16αβ in $CDCl_3$

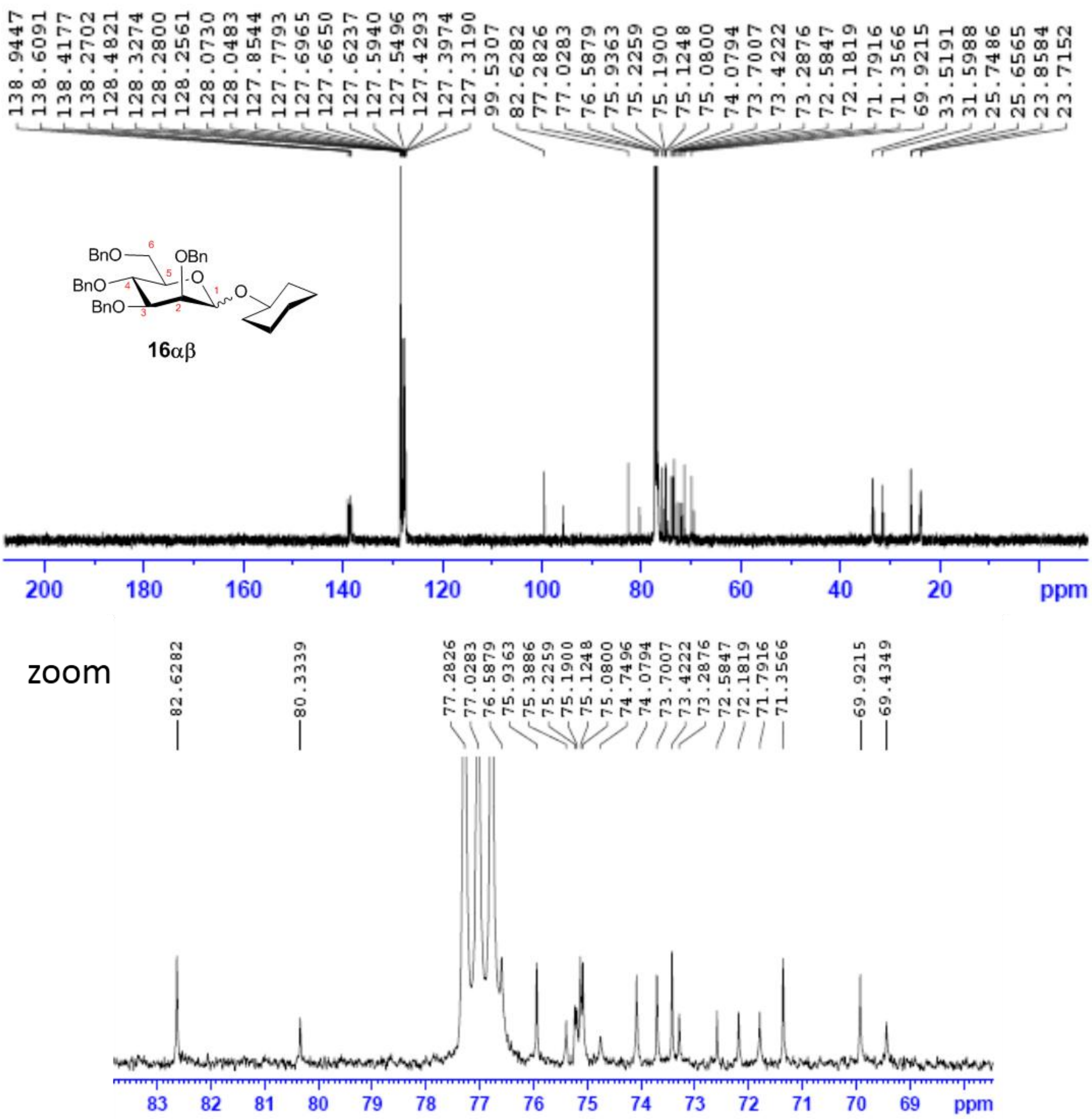

DEPT NMR of 16αβ in $CDCl_3$

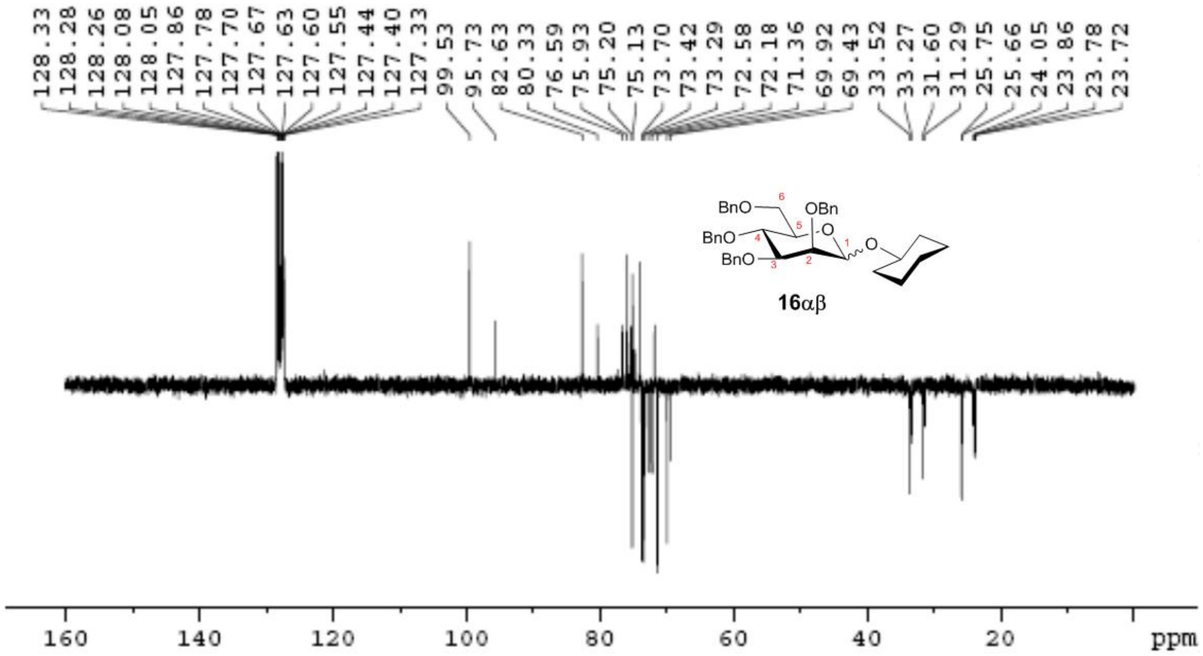

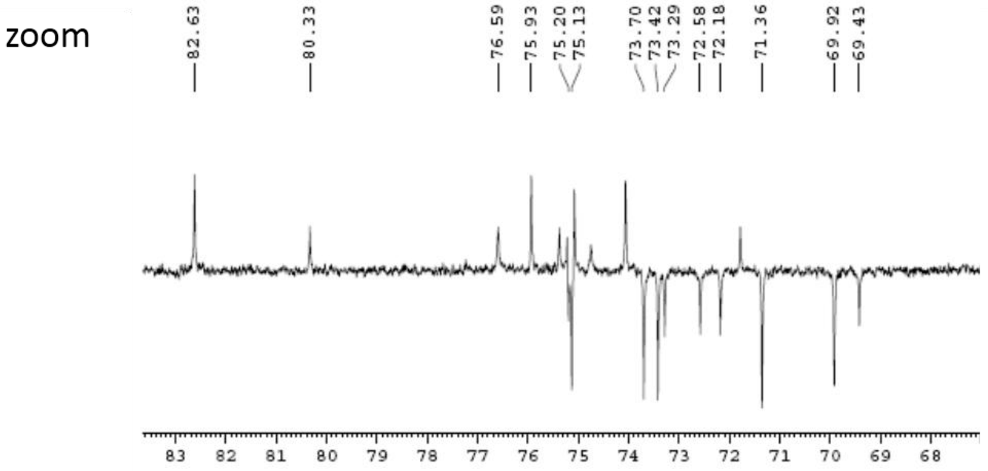

HMBC NMR of 16αβ in $CDCl_3$

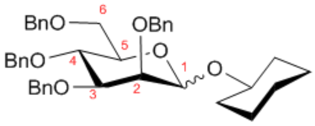

16αβ

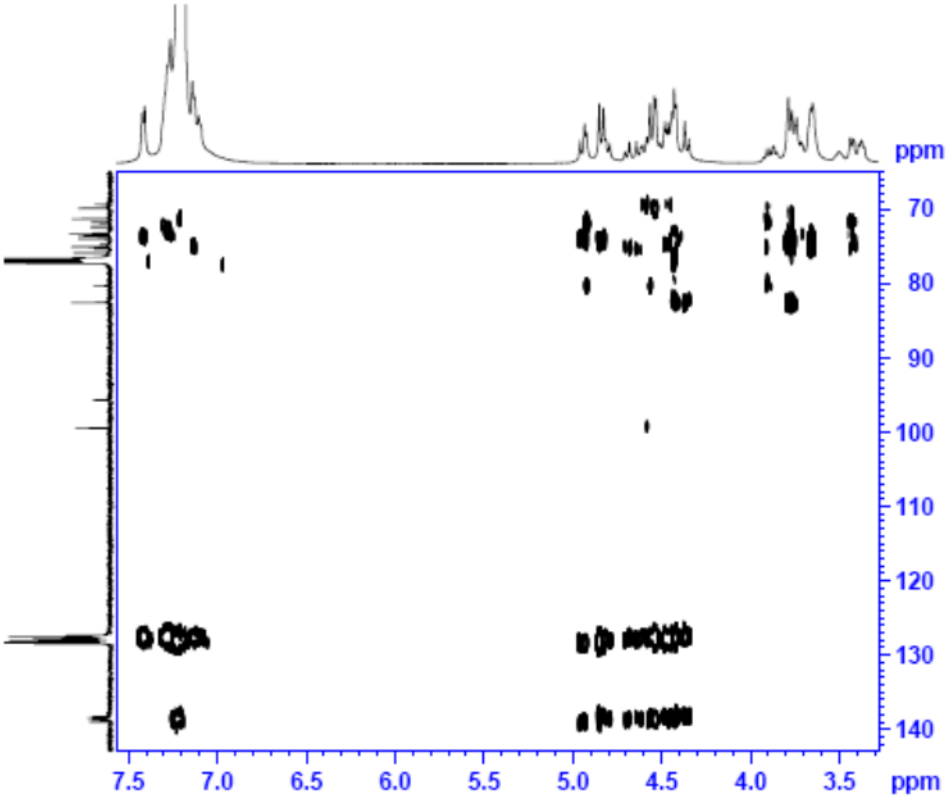

HMQC NMR of 16αβ in $CDCl_3$

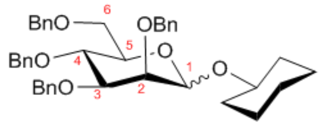

16αβ

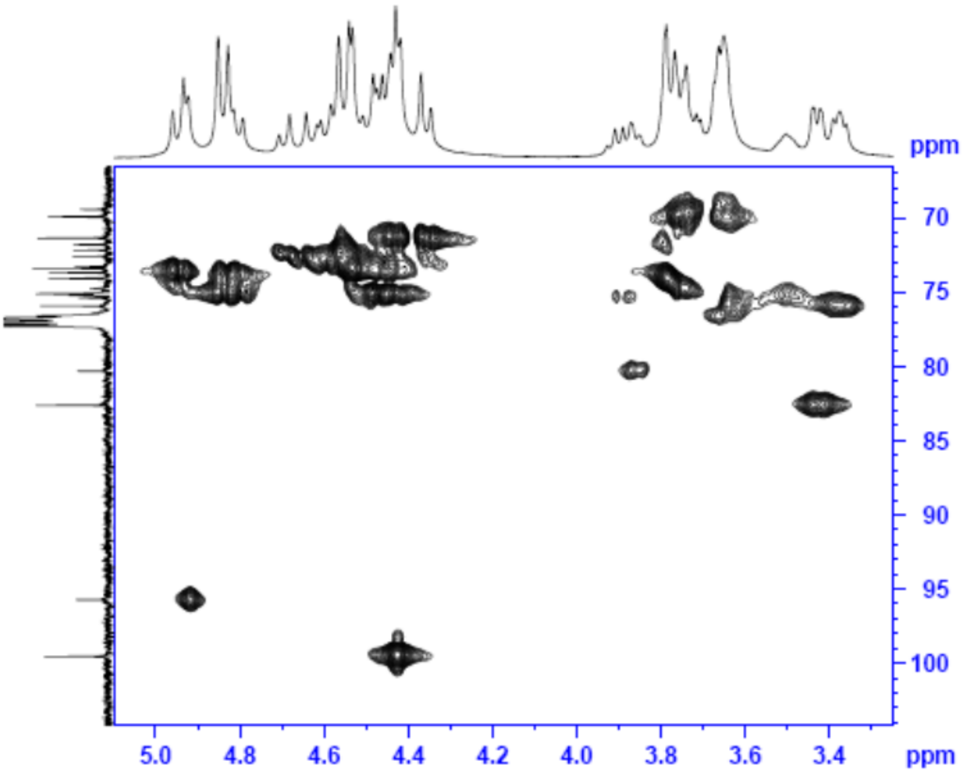

^{1}H NMR of 17β in $CDCl_3$

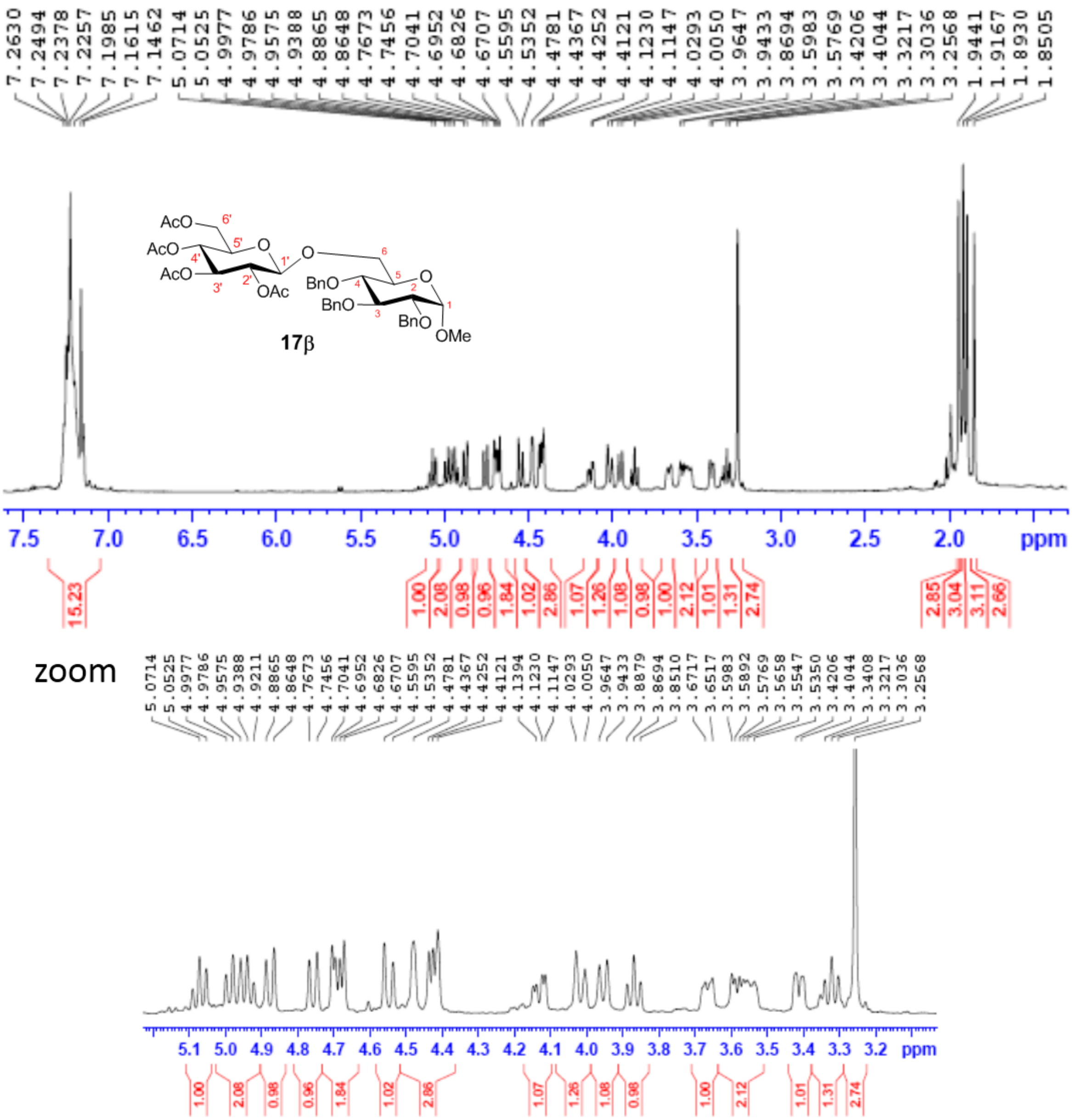

COSY NMR of 17β in $CDCl_3$

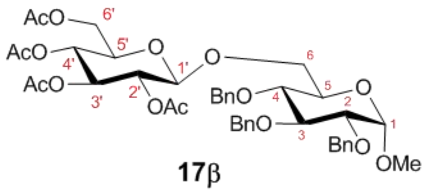

17β

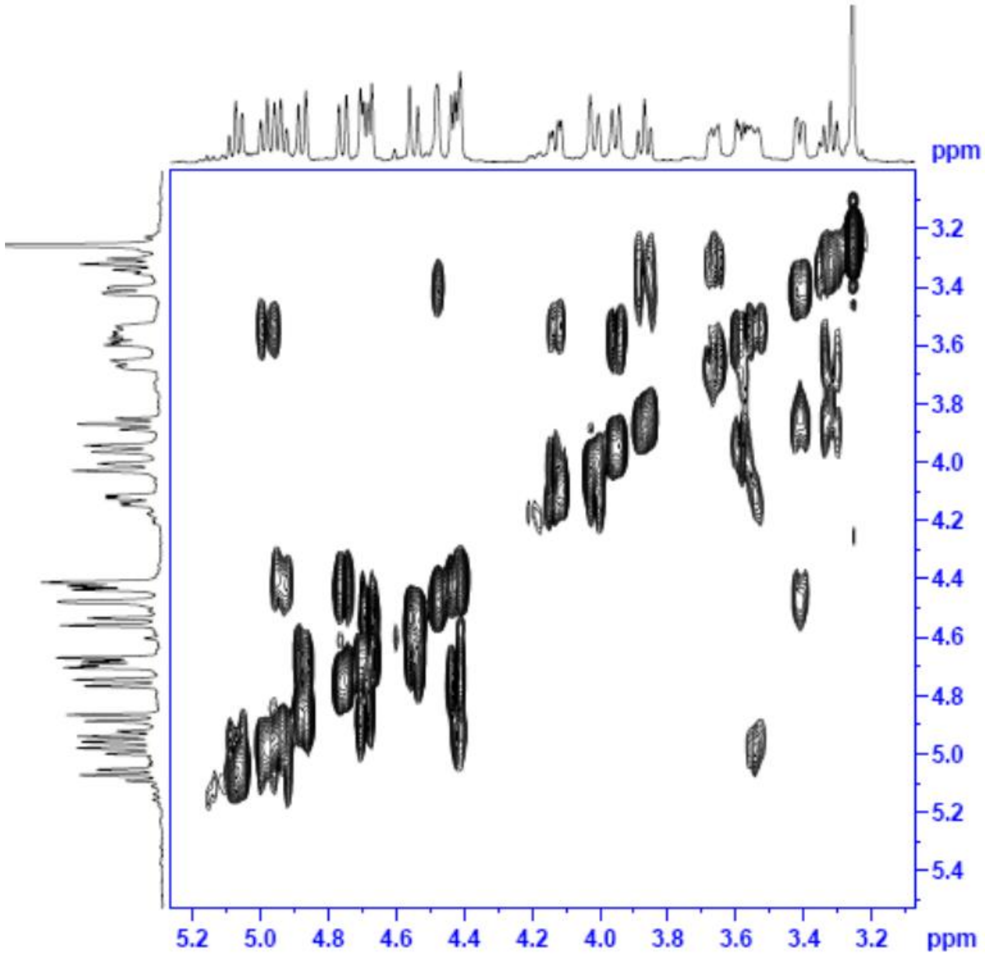

^{1}H NMR of 18β in $CDCl_3$

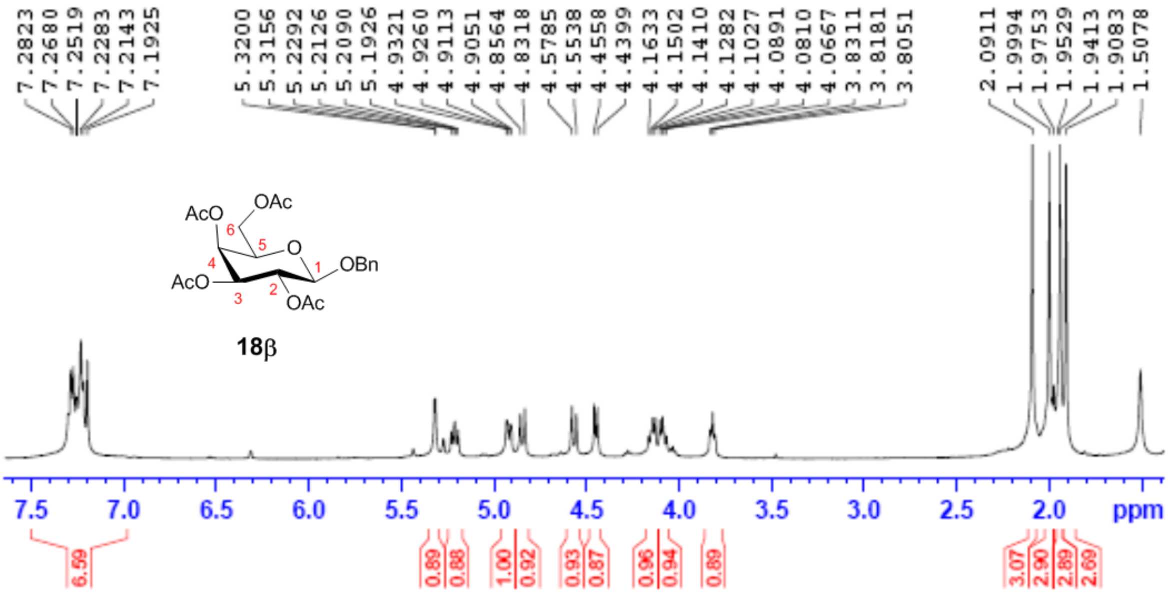

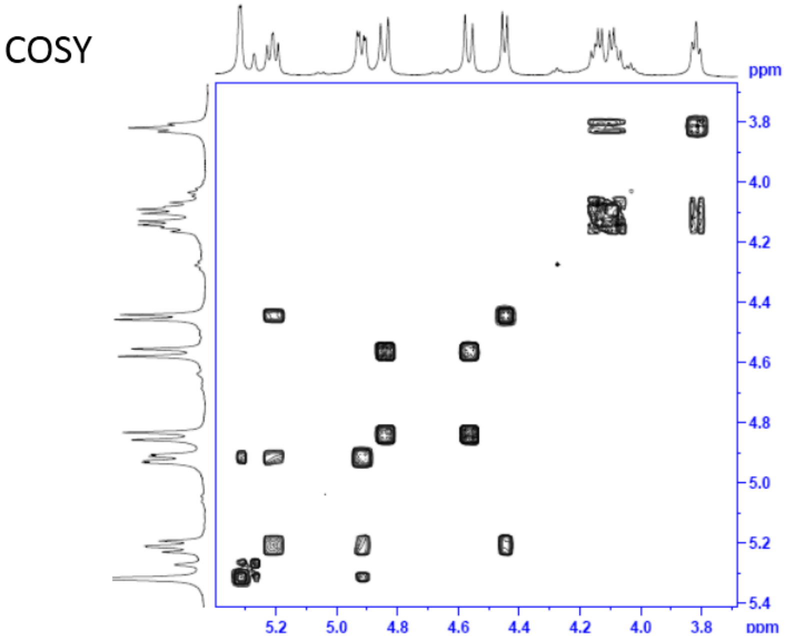

^{1}H NMR of 19β in $CDCl_3$

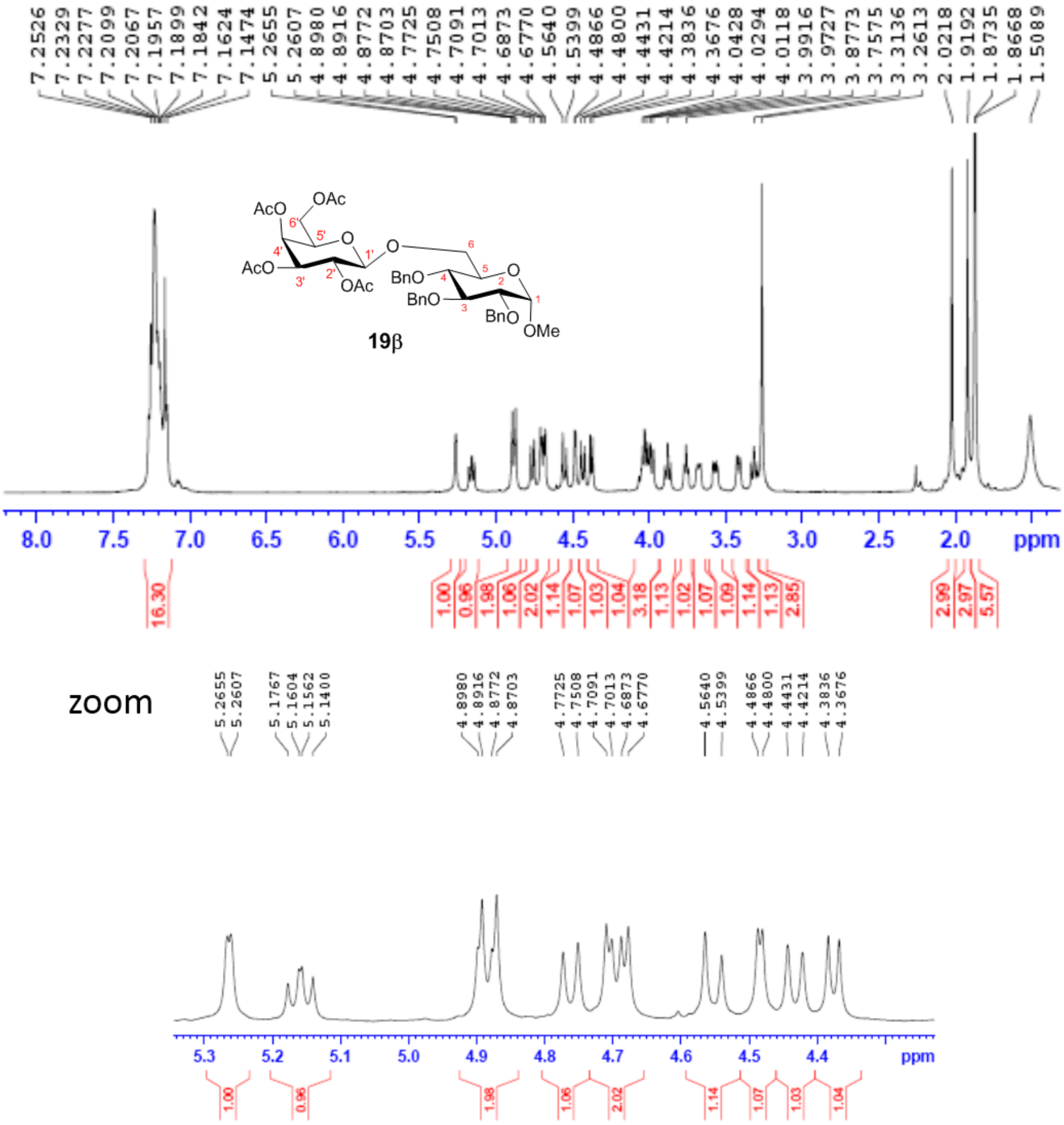

^{1}H NMR of 20α in $CDCl_3$

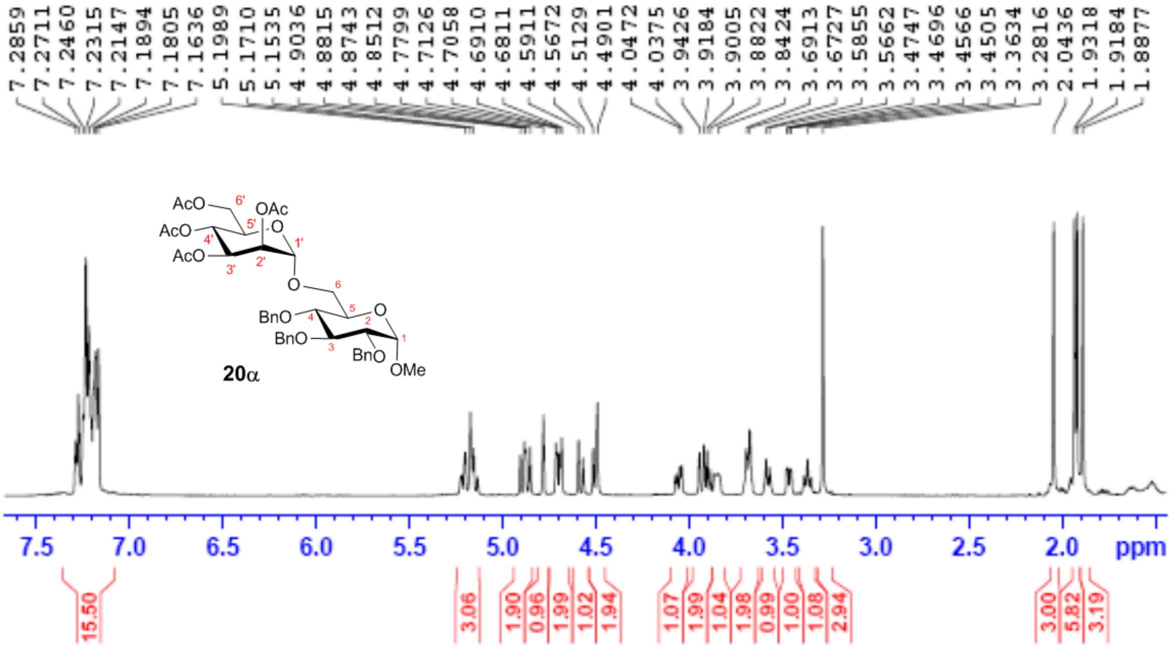

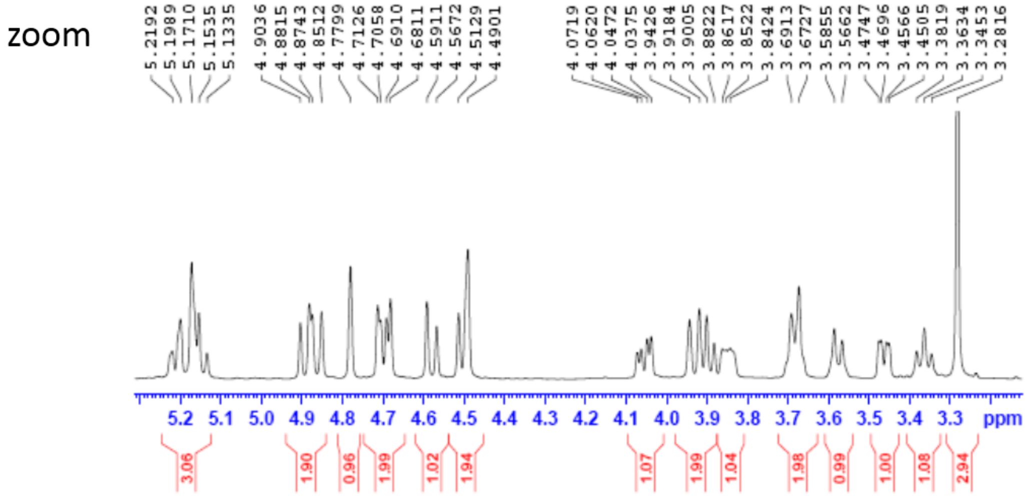

COSY NMR of 20α in $CDCl_3$

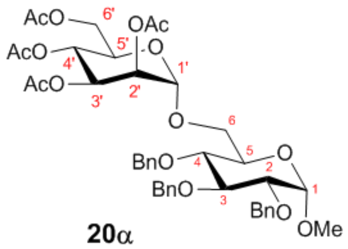

20α

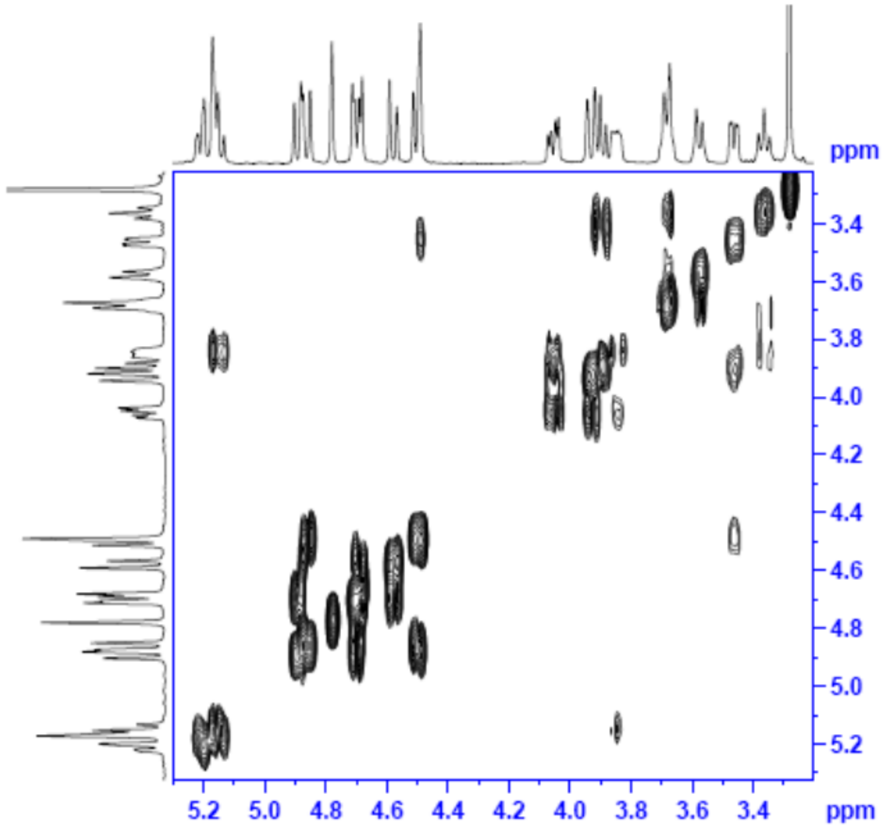

^{1}H NMR of 21α in $CDCl_3$

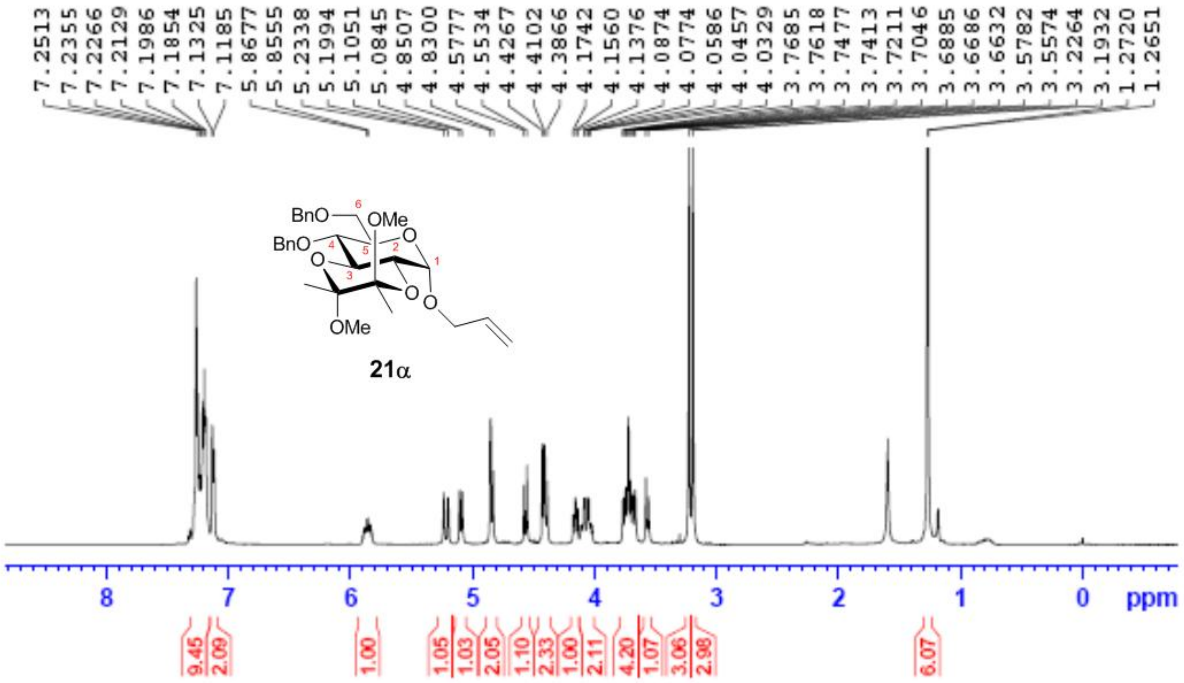

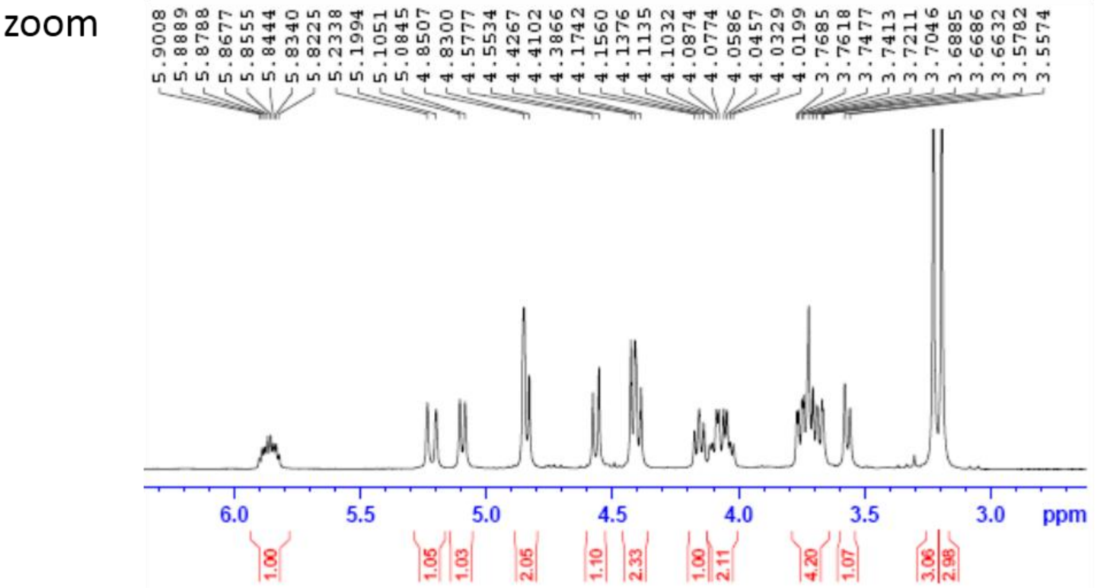

COSY NMR of 21α in $CDCl_3$

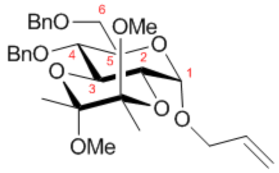

21α

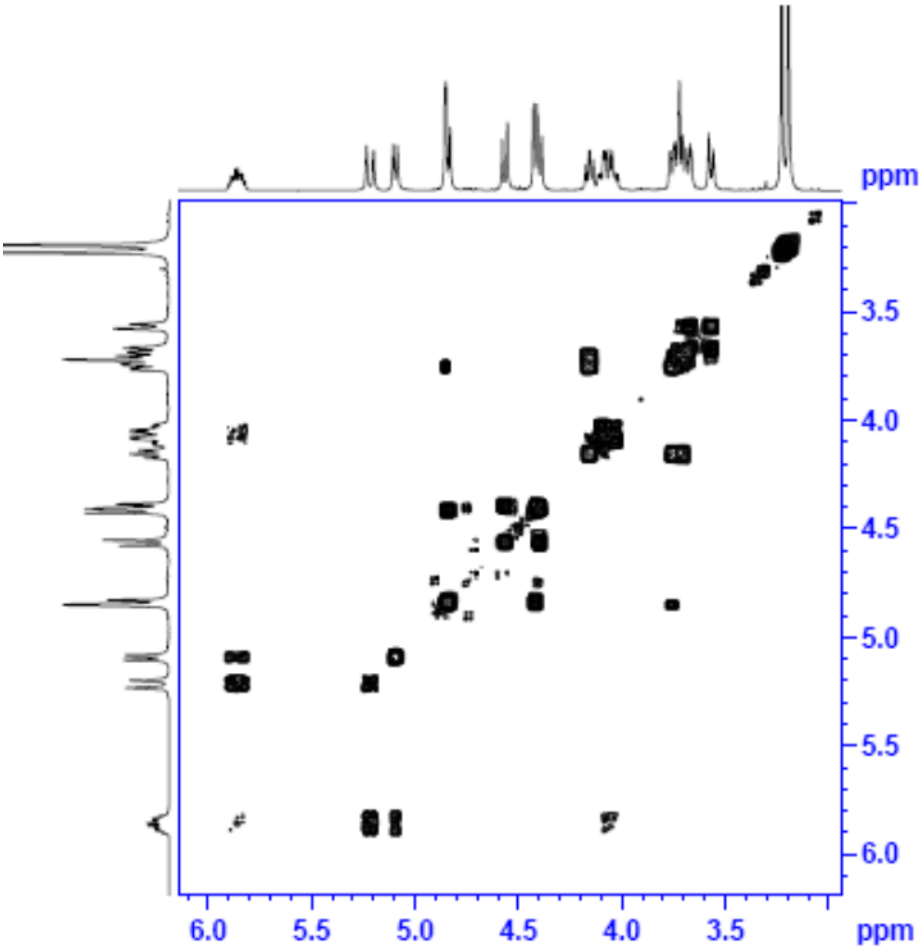

^{13}C NMR of 21α in $CDCl_3$

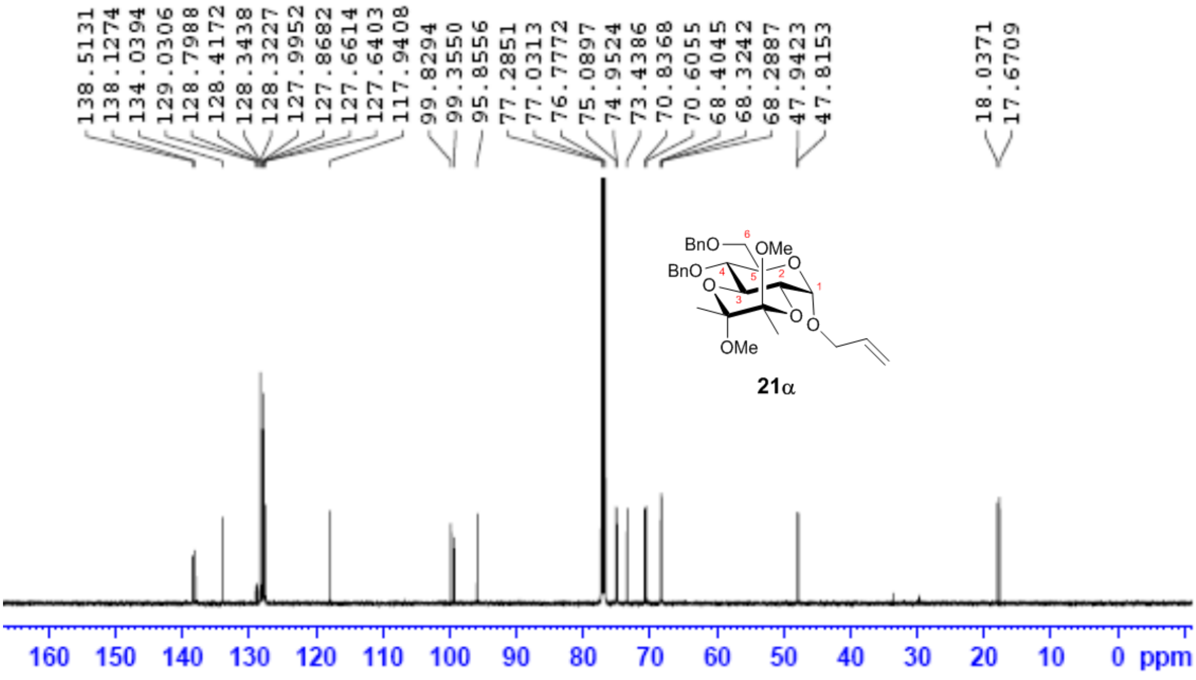

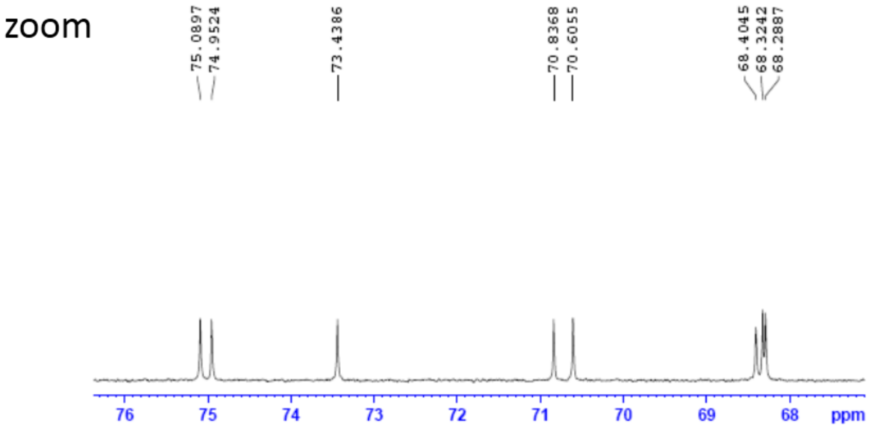

DEPT NMR of 21α in $CDCl_3$

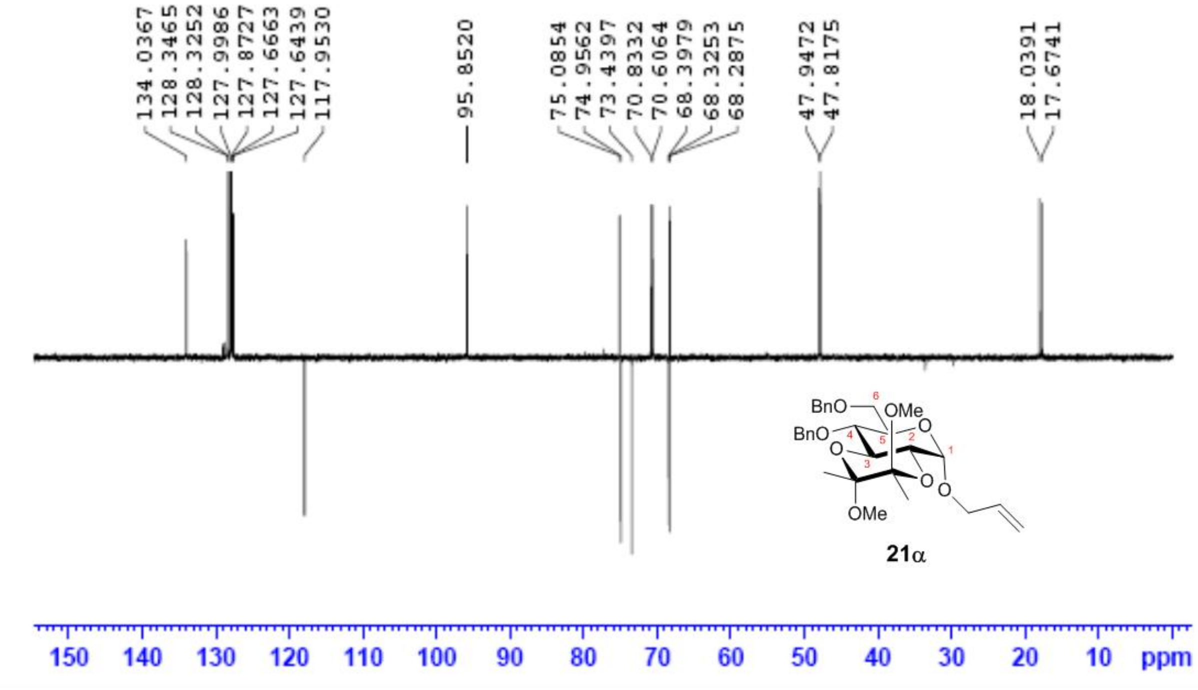

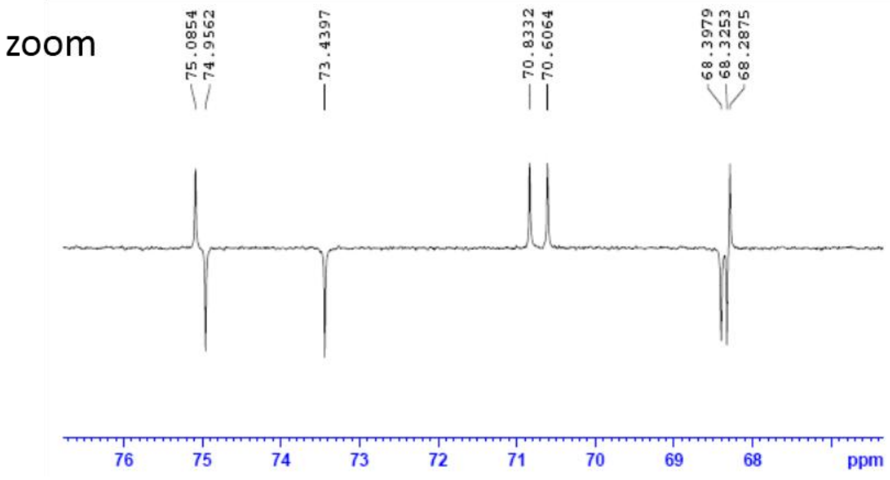

HMBC NMR of 21α in $CDCl_3$

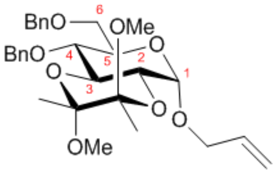

21α

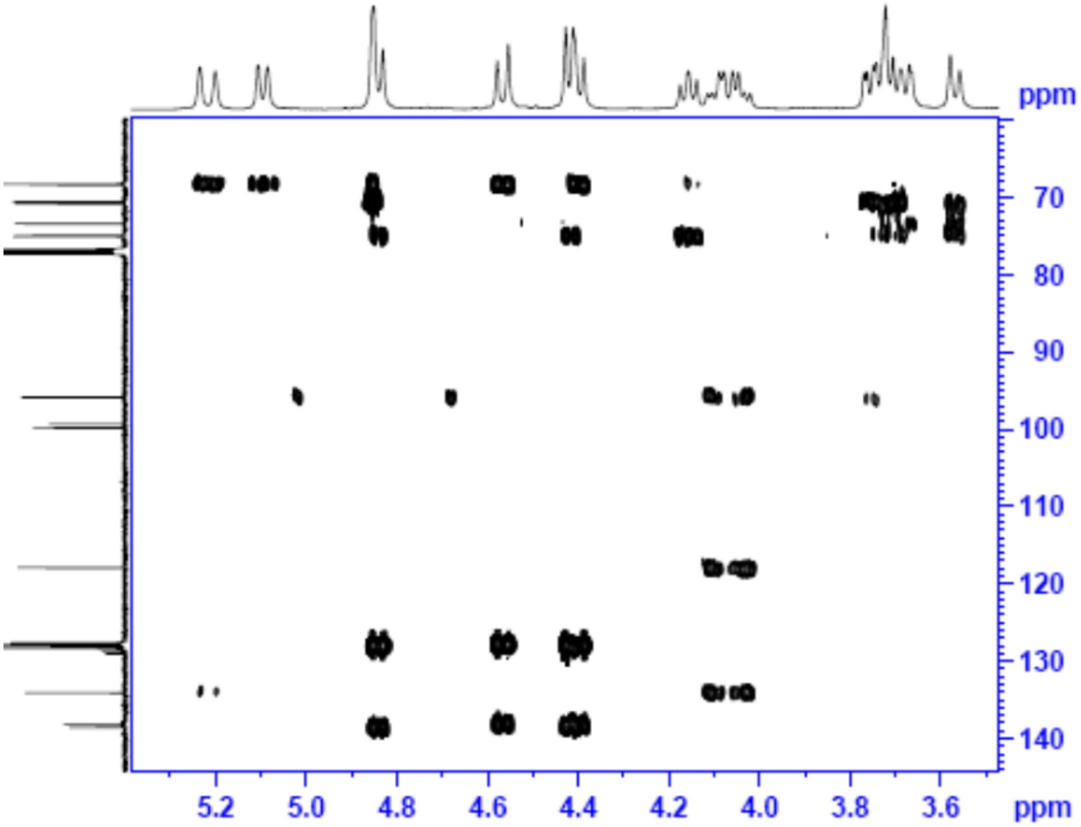

HMQC NMR of 21α in $CDCl_3$

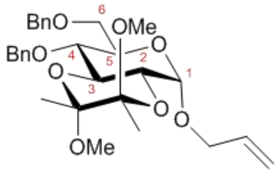

21α

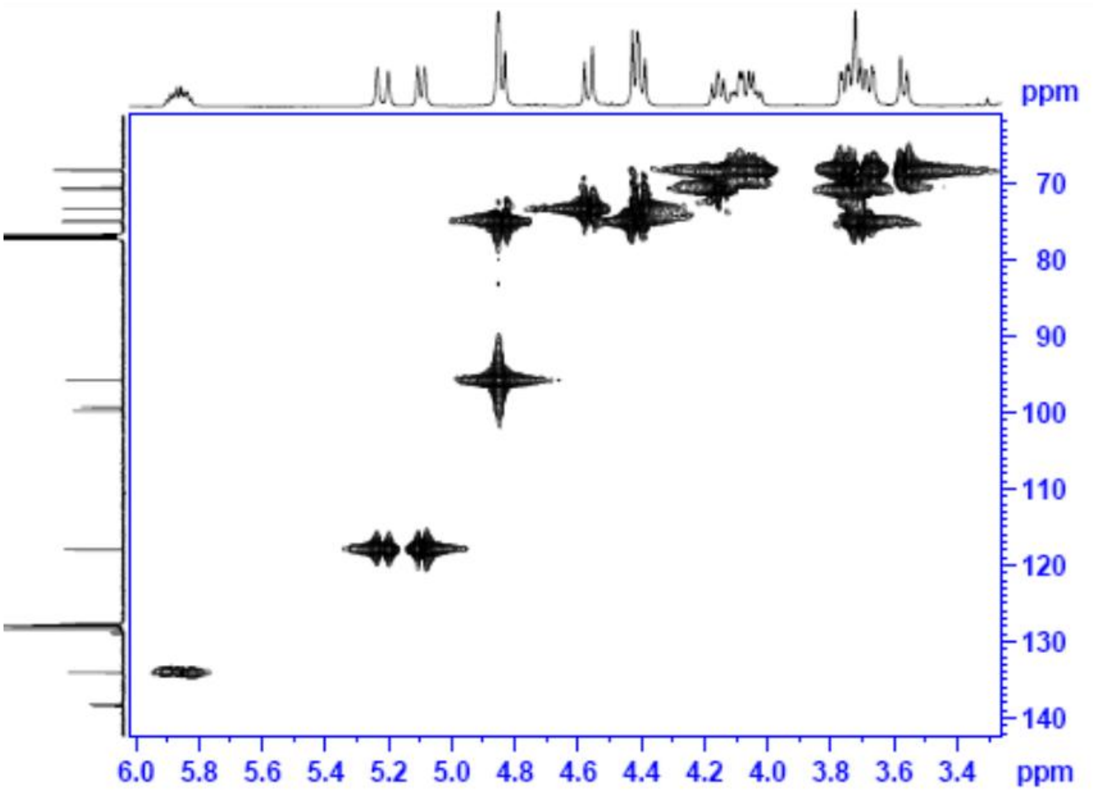

^{1}H NMR of 21β in $CDCl_3$

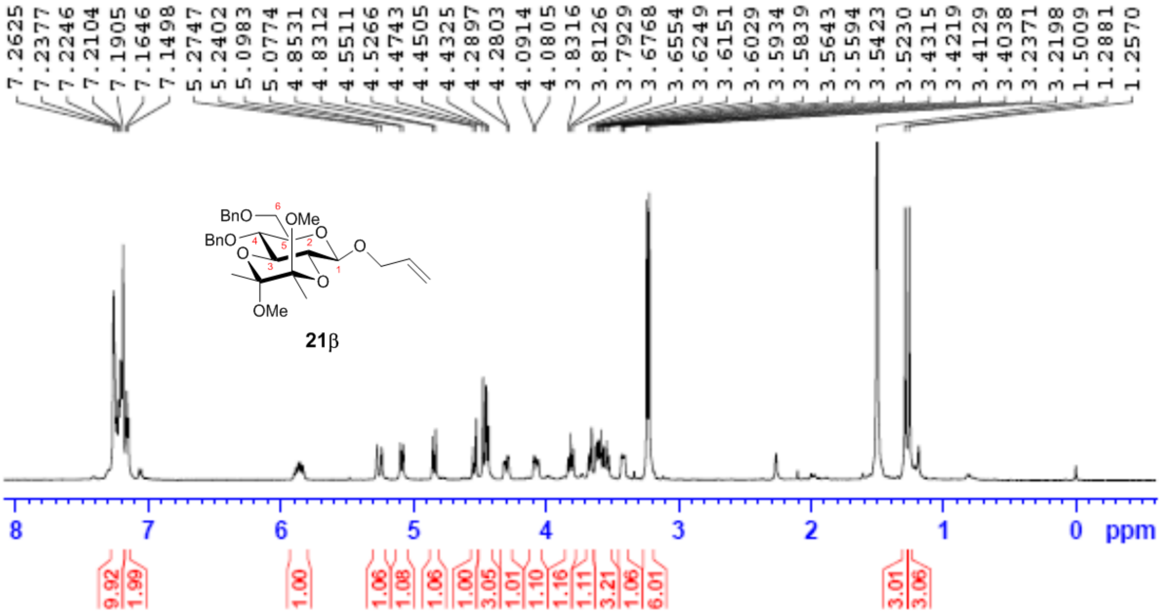

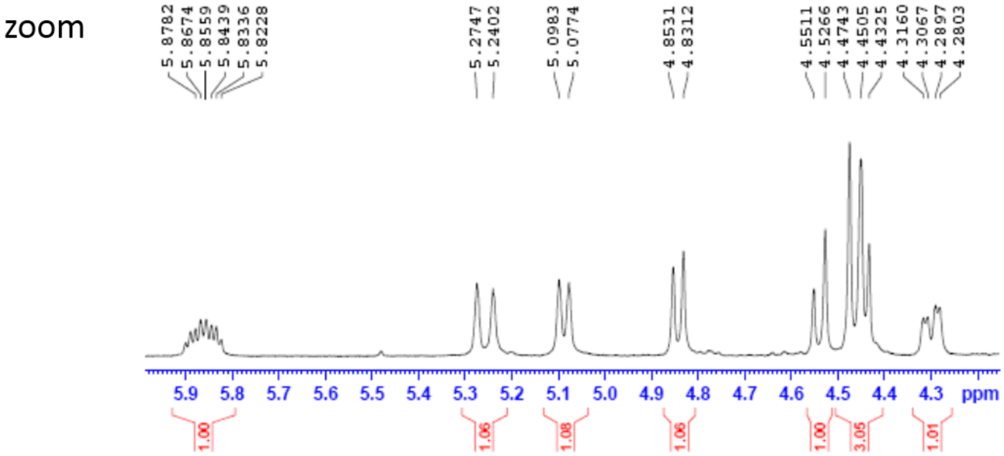

COSY NMR of 21β in $CDCl_3$

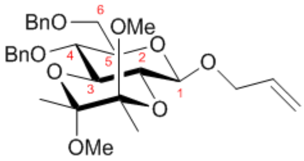

21β

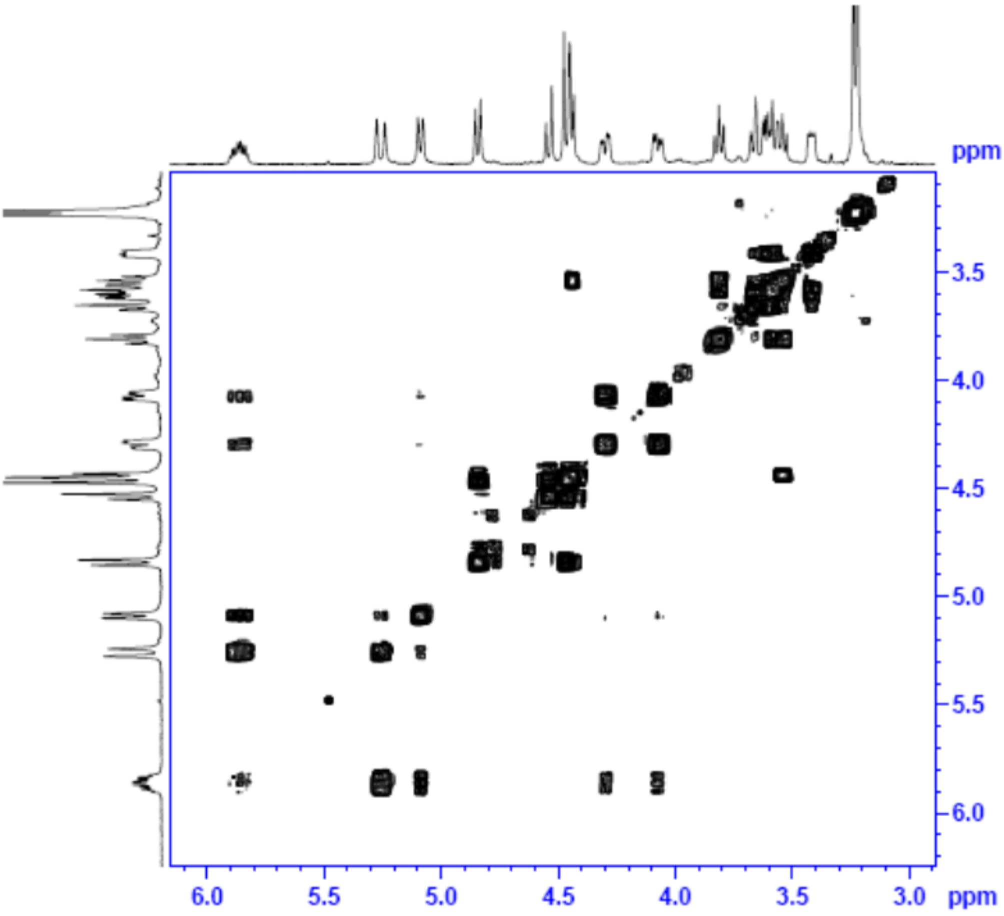

^{13}C NMR of 21β in $CDCl_3$

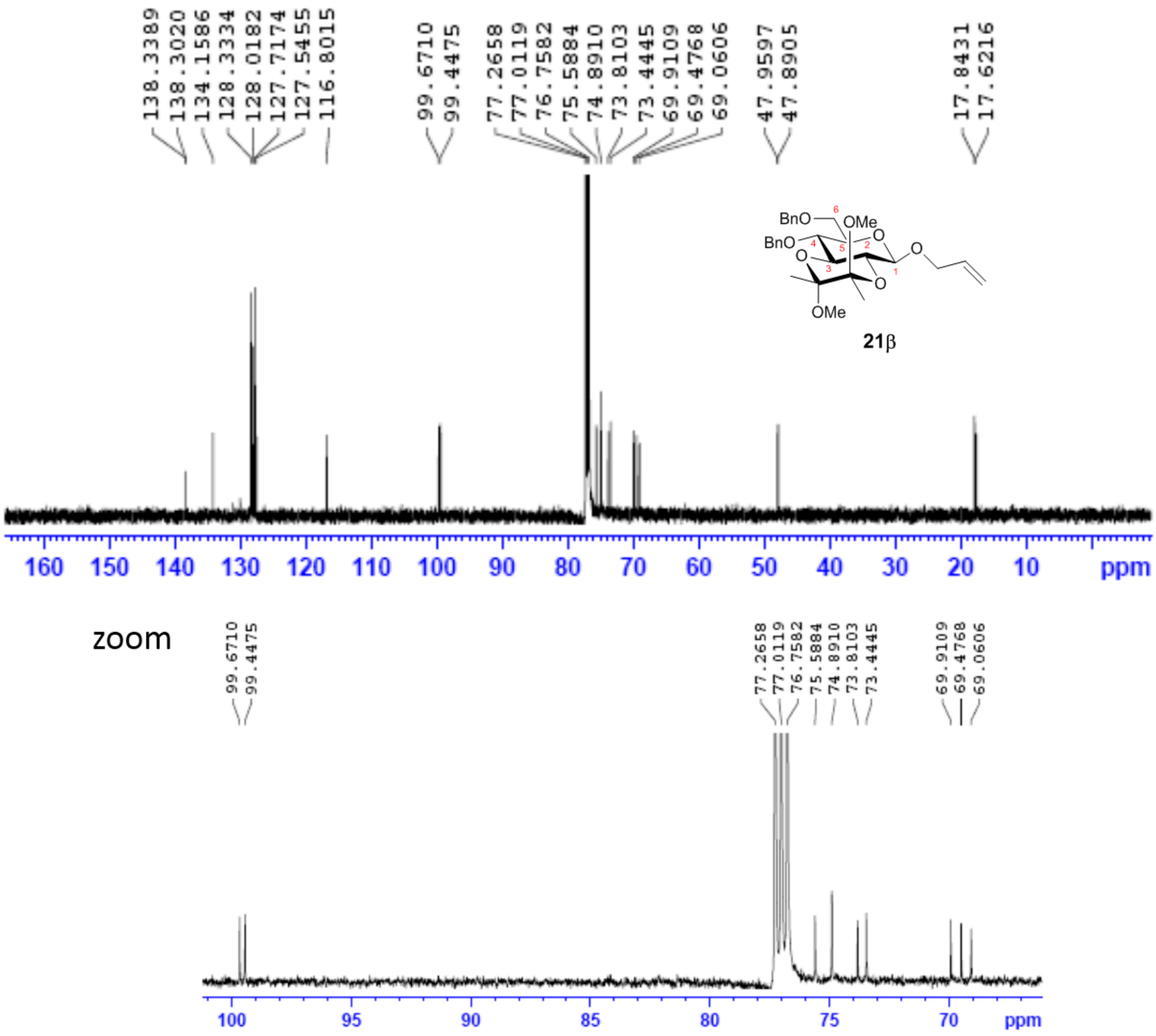

DEPT NMR of 21β in $CDCl_3$

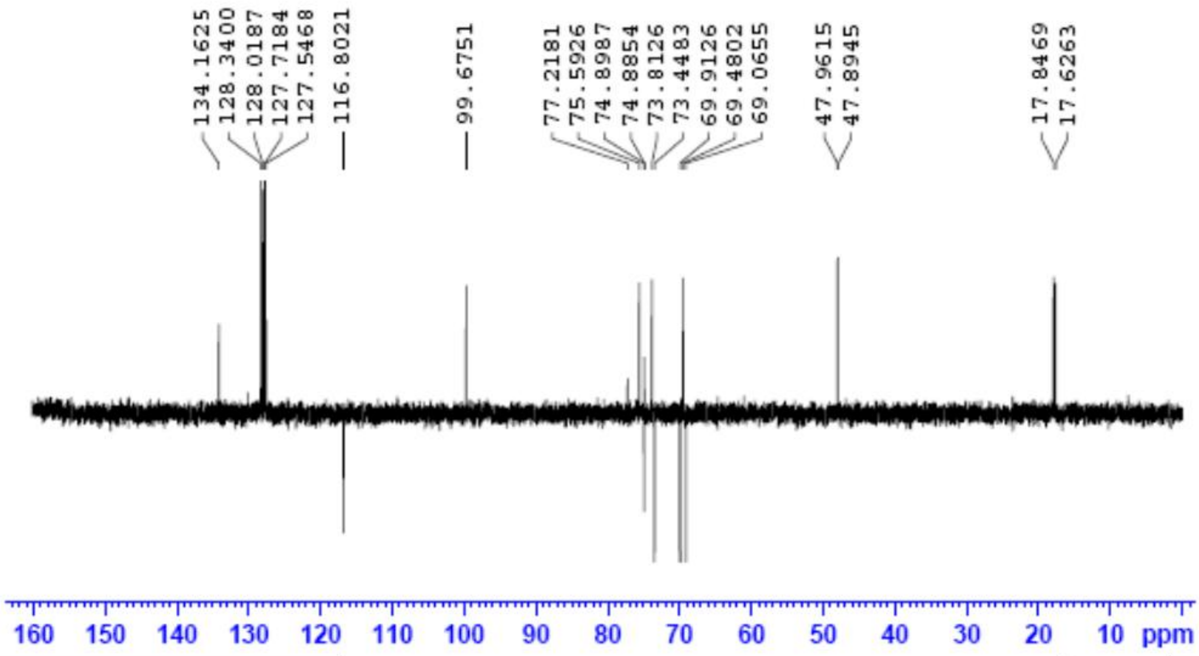

HMBC

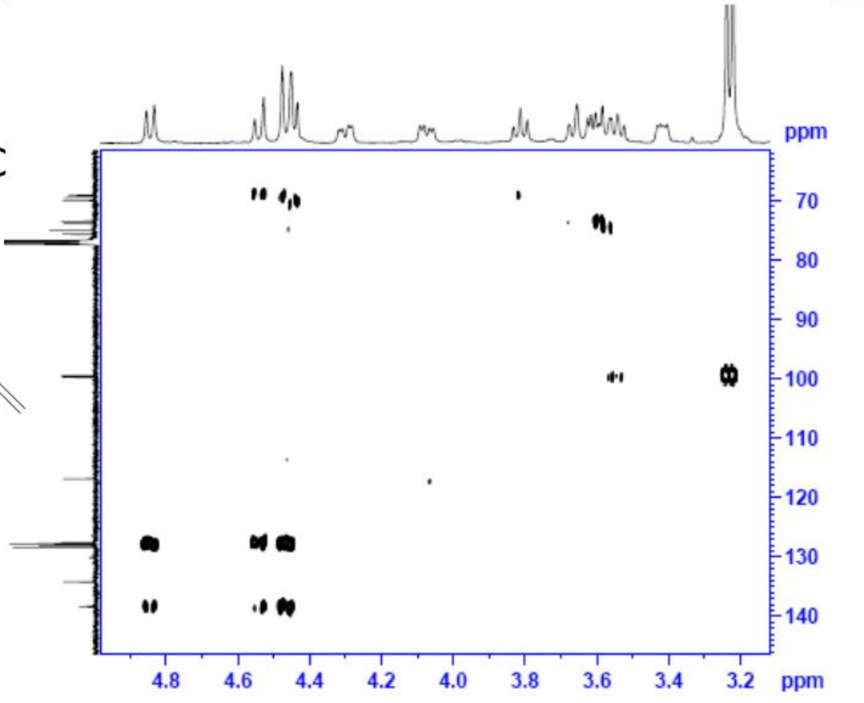

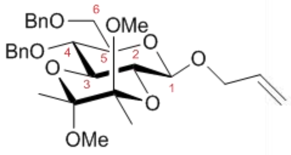

21β

HMQC NMR of 21β in $CDCl_3$

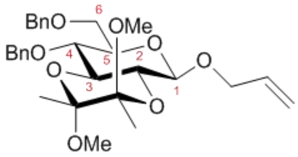

21β

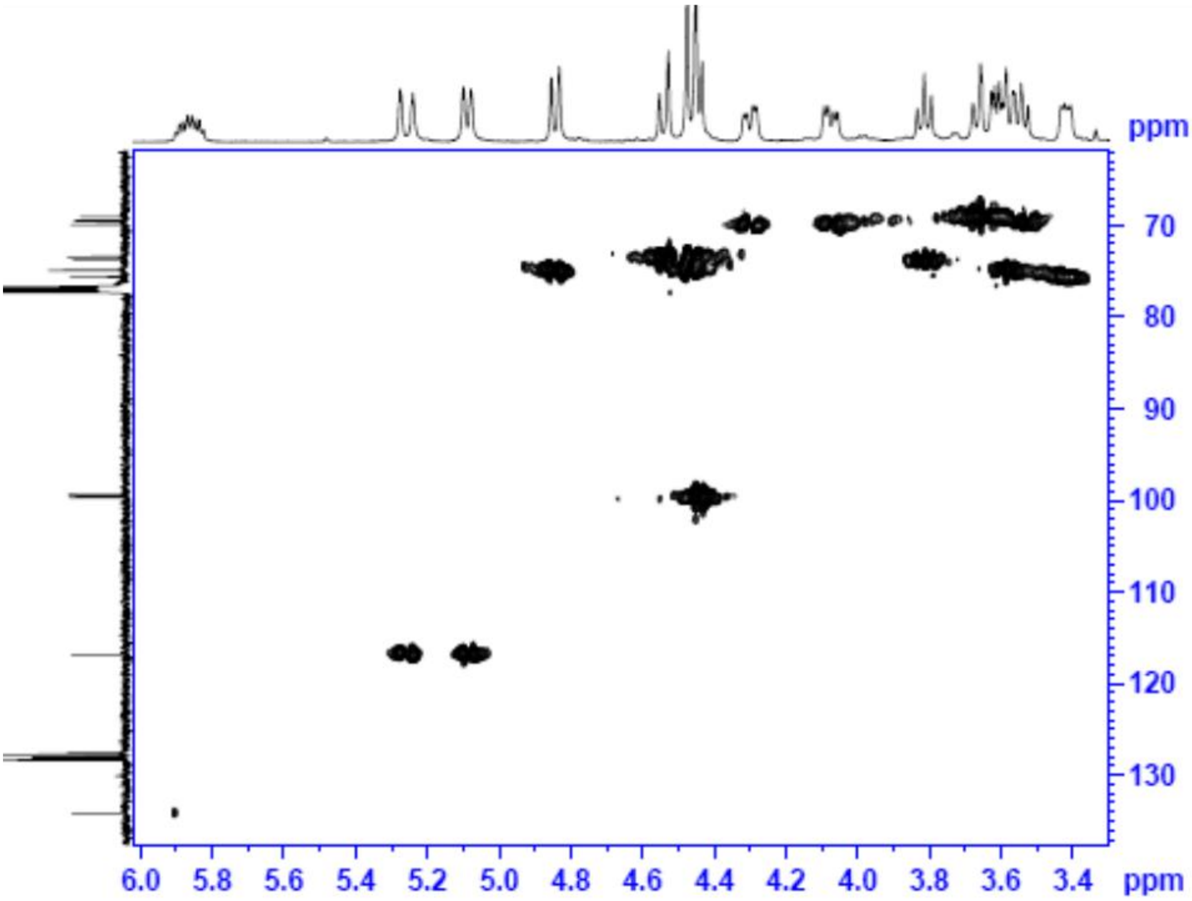

^{1}H NMR of 22αβ in $CDCl_3$

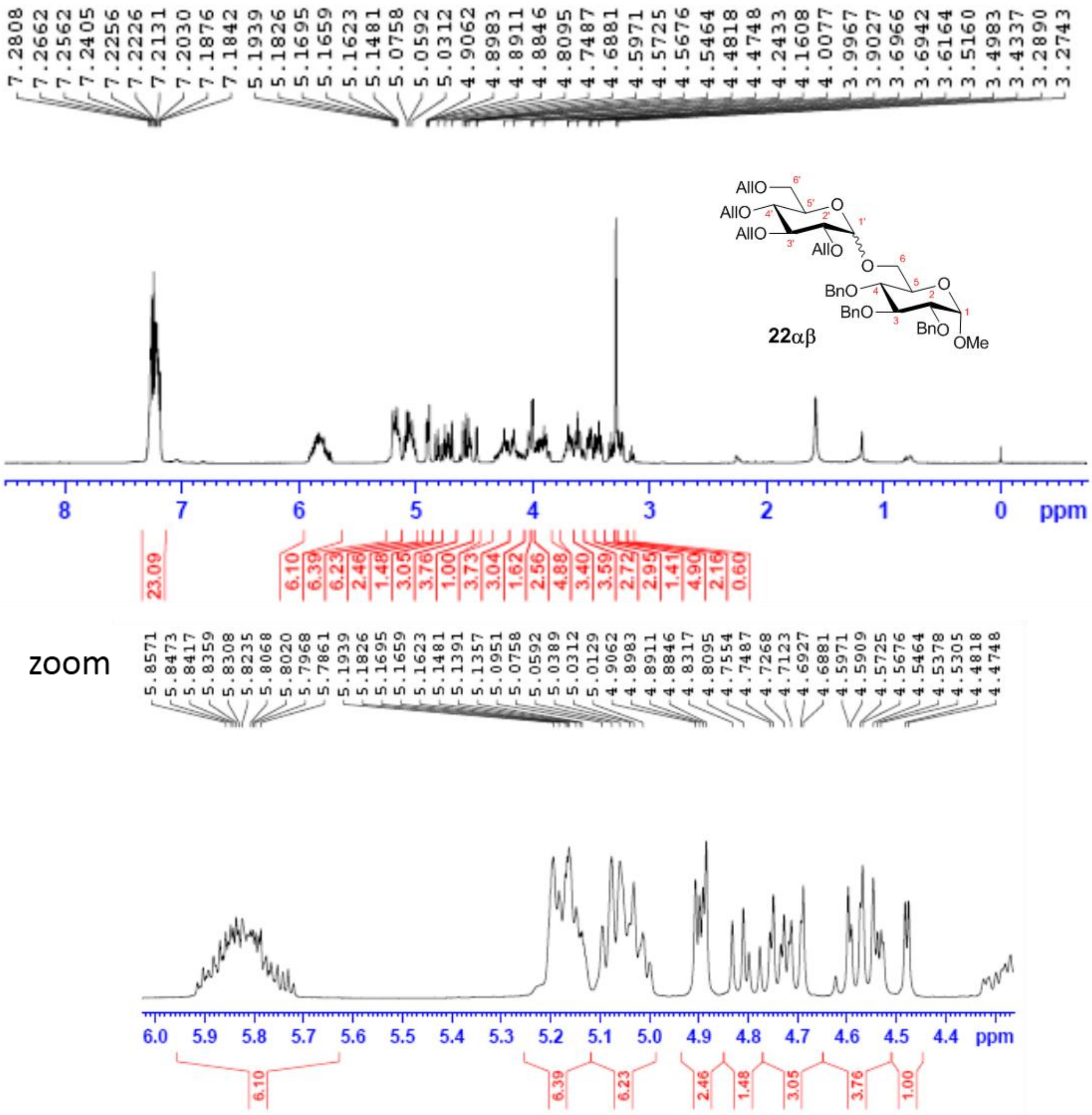

COSY NMR of 22αβ in $CDCl_3$

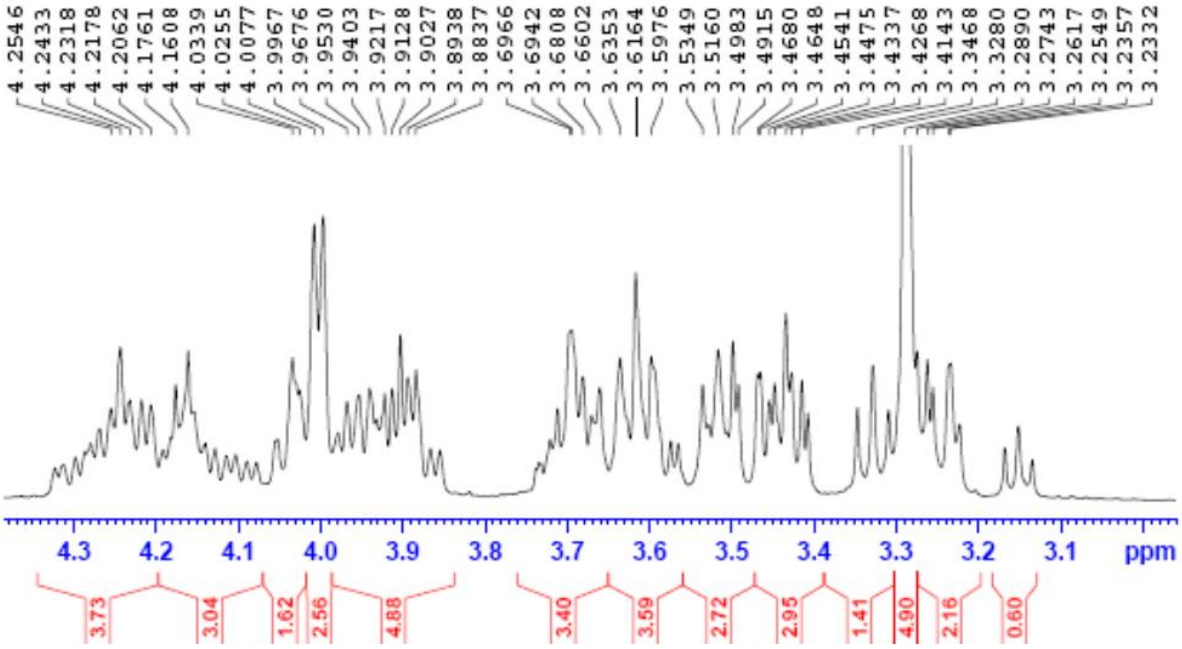

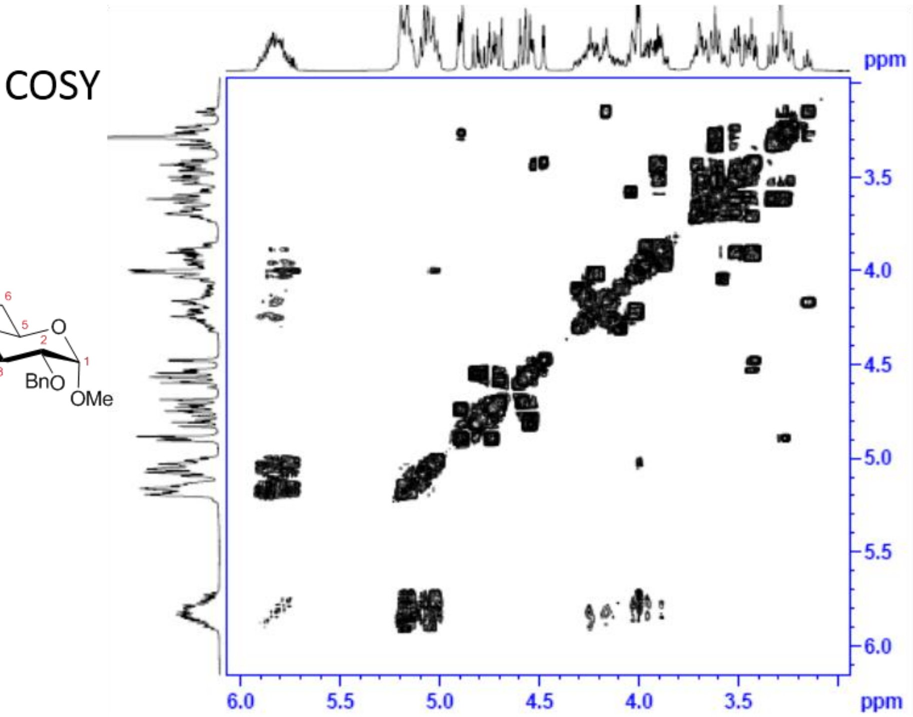

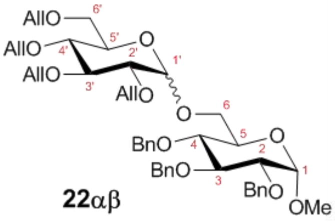

22αβ

^{13}C NMR of 22αβ in $CDCl_3$

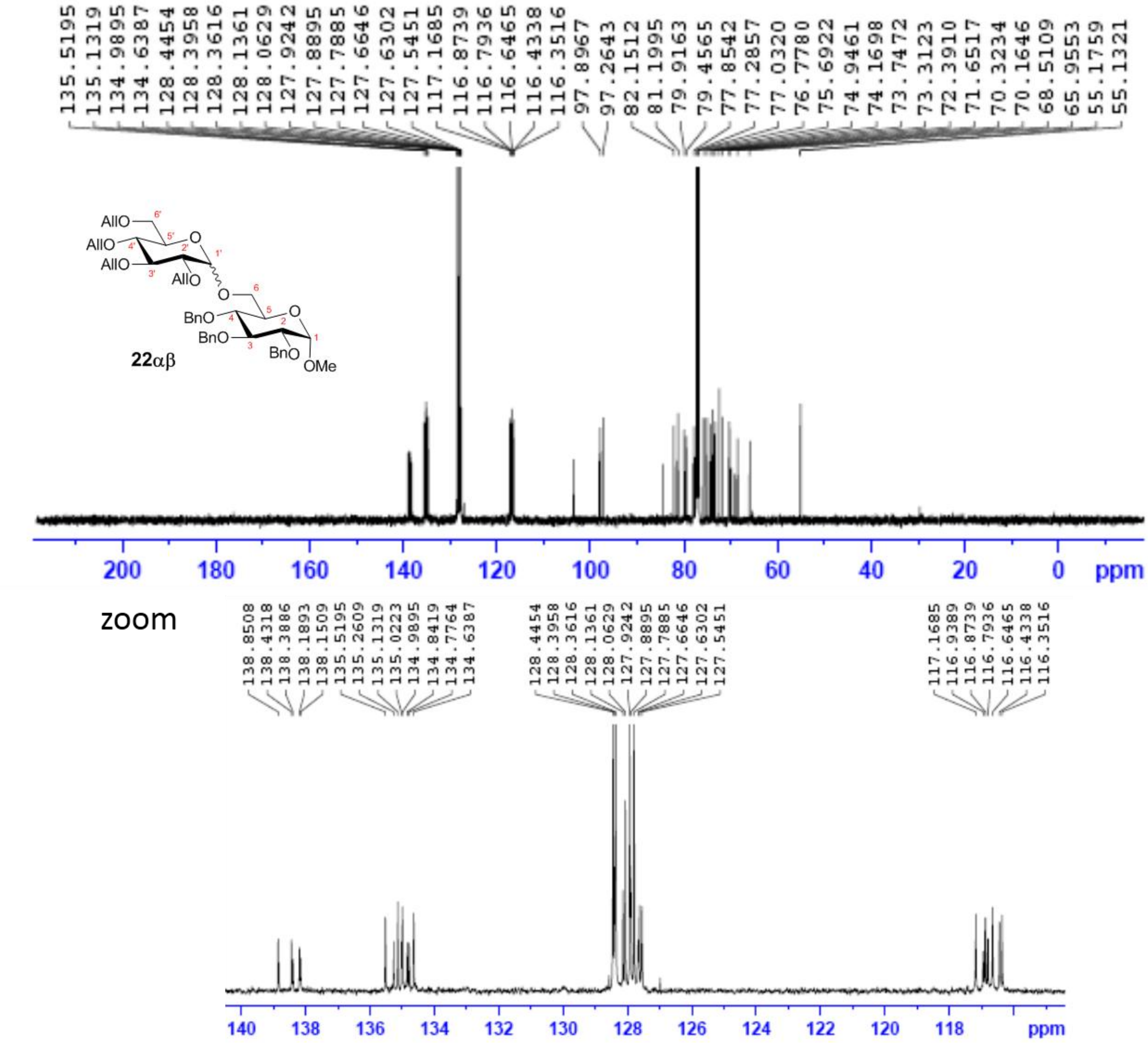

DEPT NMR of 22αβ in $CDCl_3$

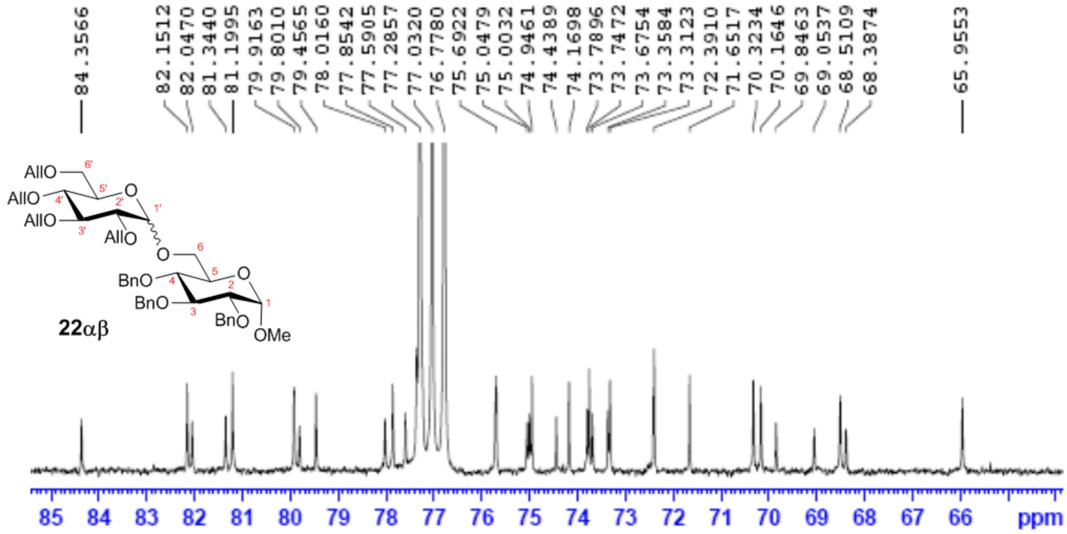

DEPT

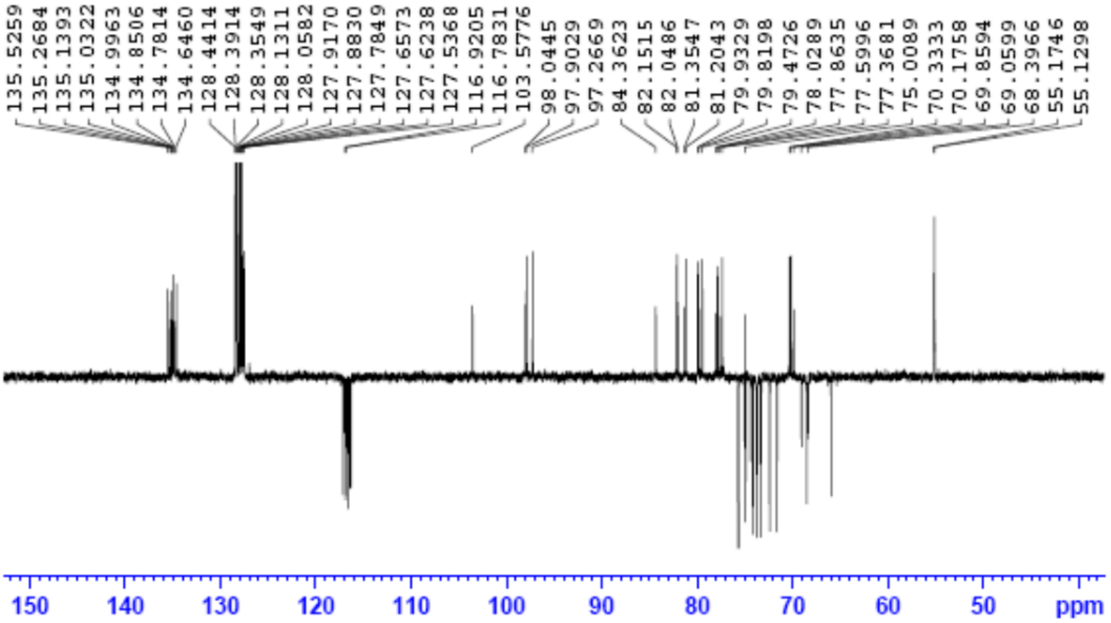

HMBC NMR of 22αβ in $CDCl_3$

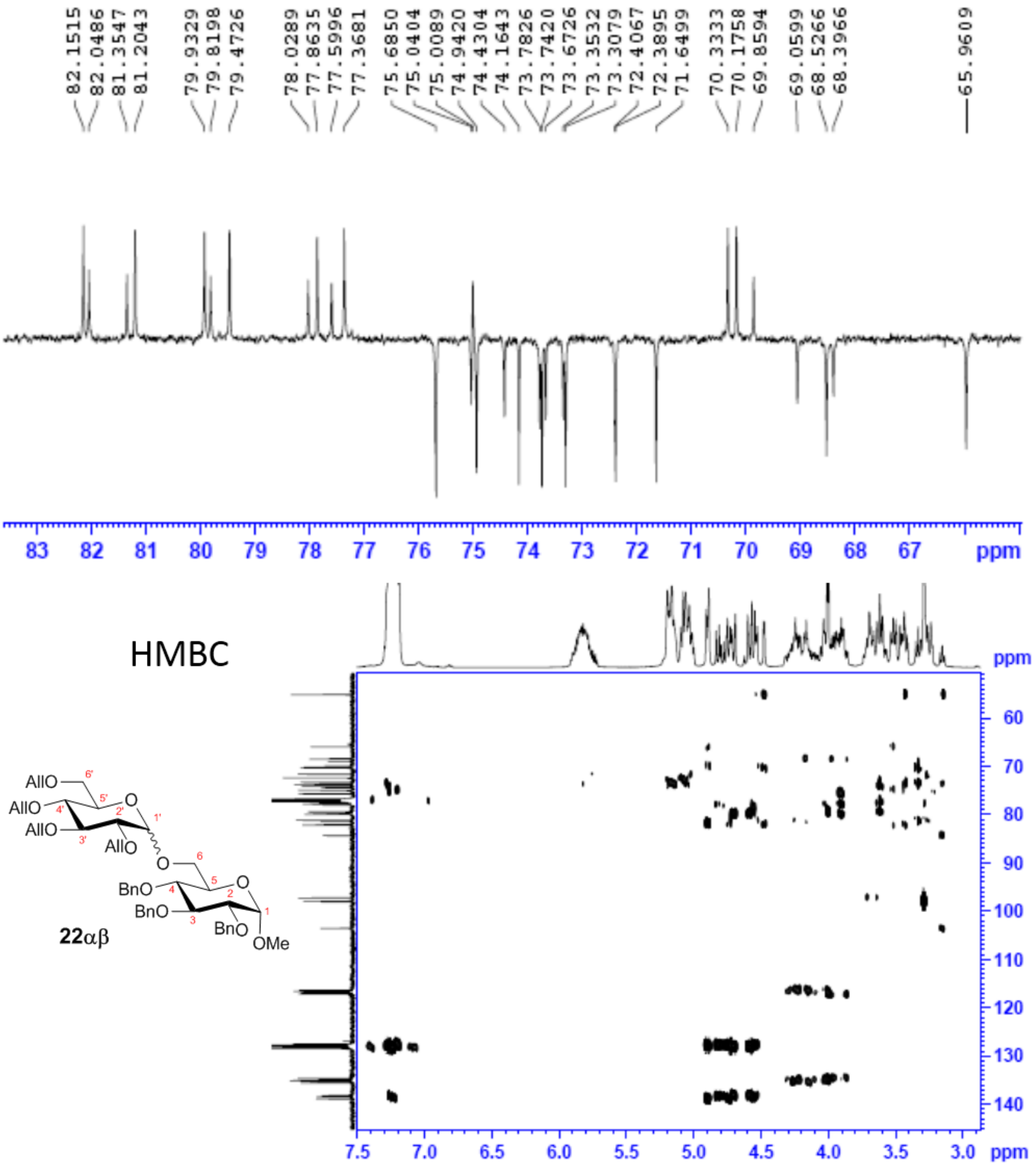

22αβ

HMQC NMR of 22αβ in $CDCl_3$

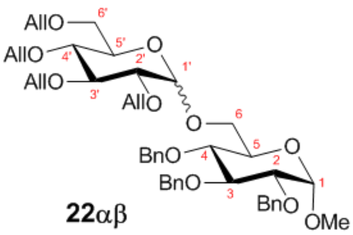

22αβ

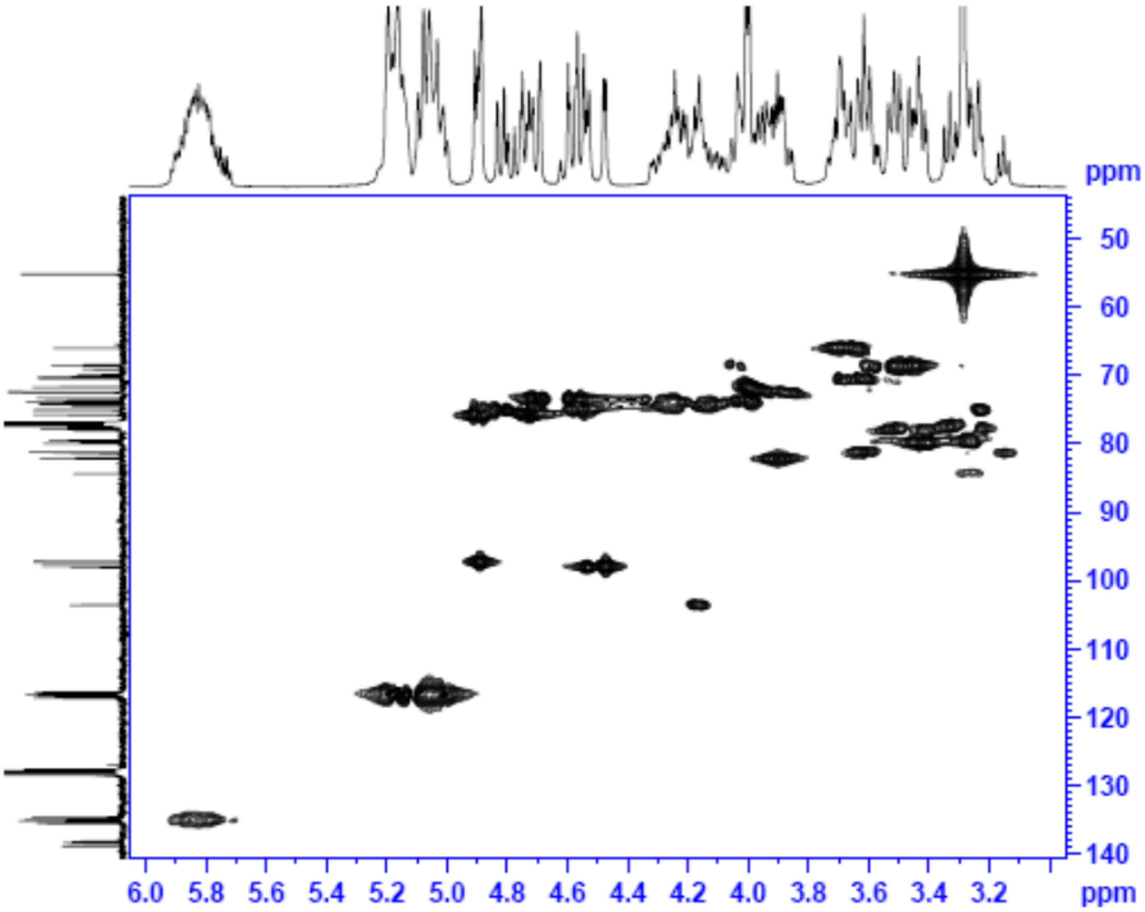

^{1}H NMR of 23α in $CDCl_3$

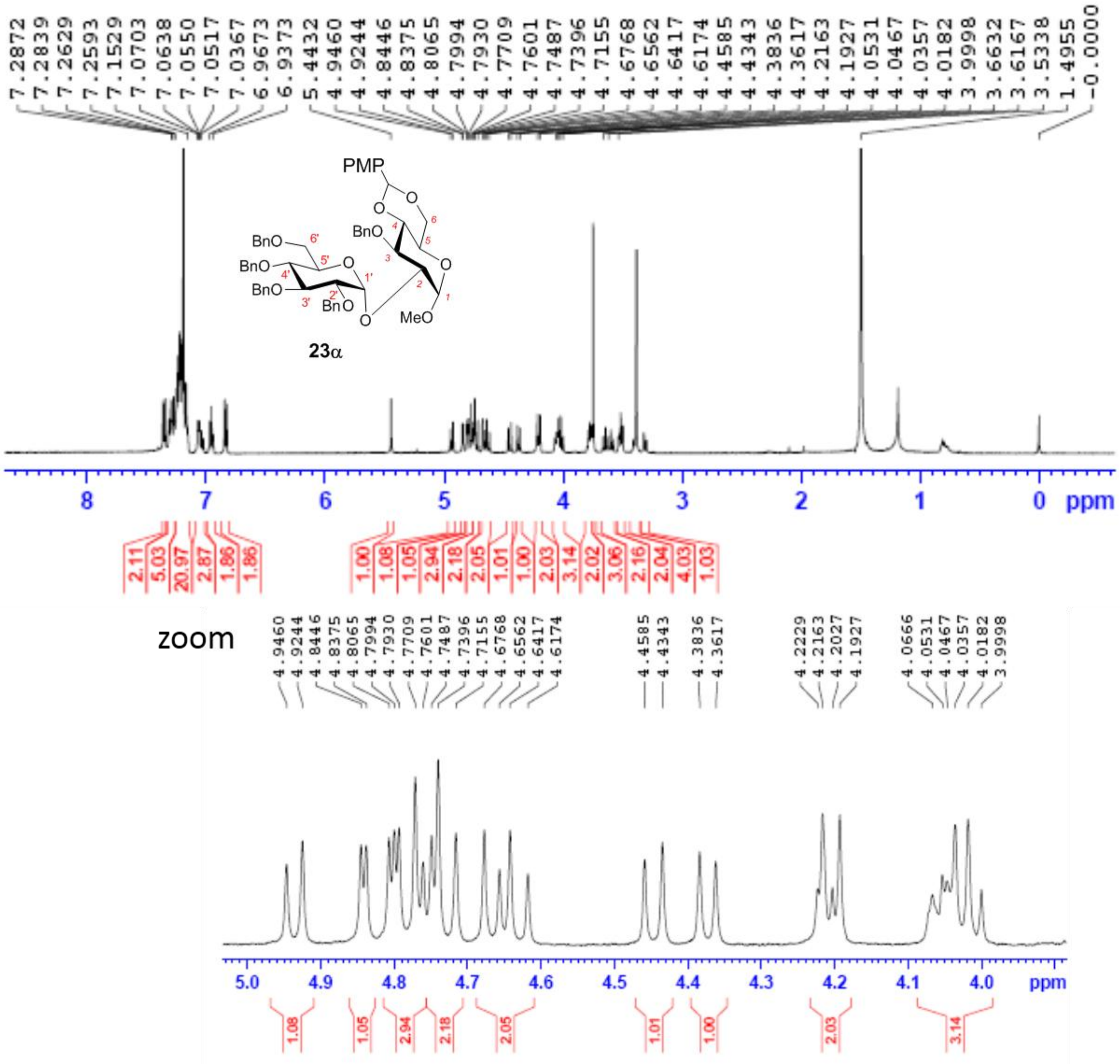

COSY NMR of 23α in $CDCl_3$

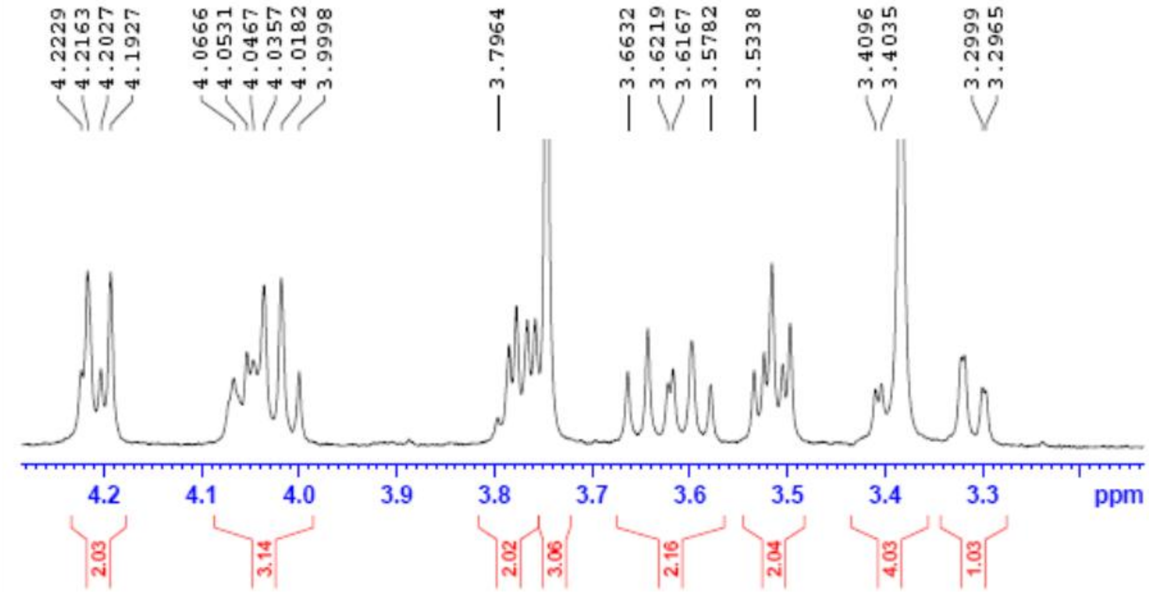

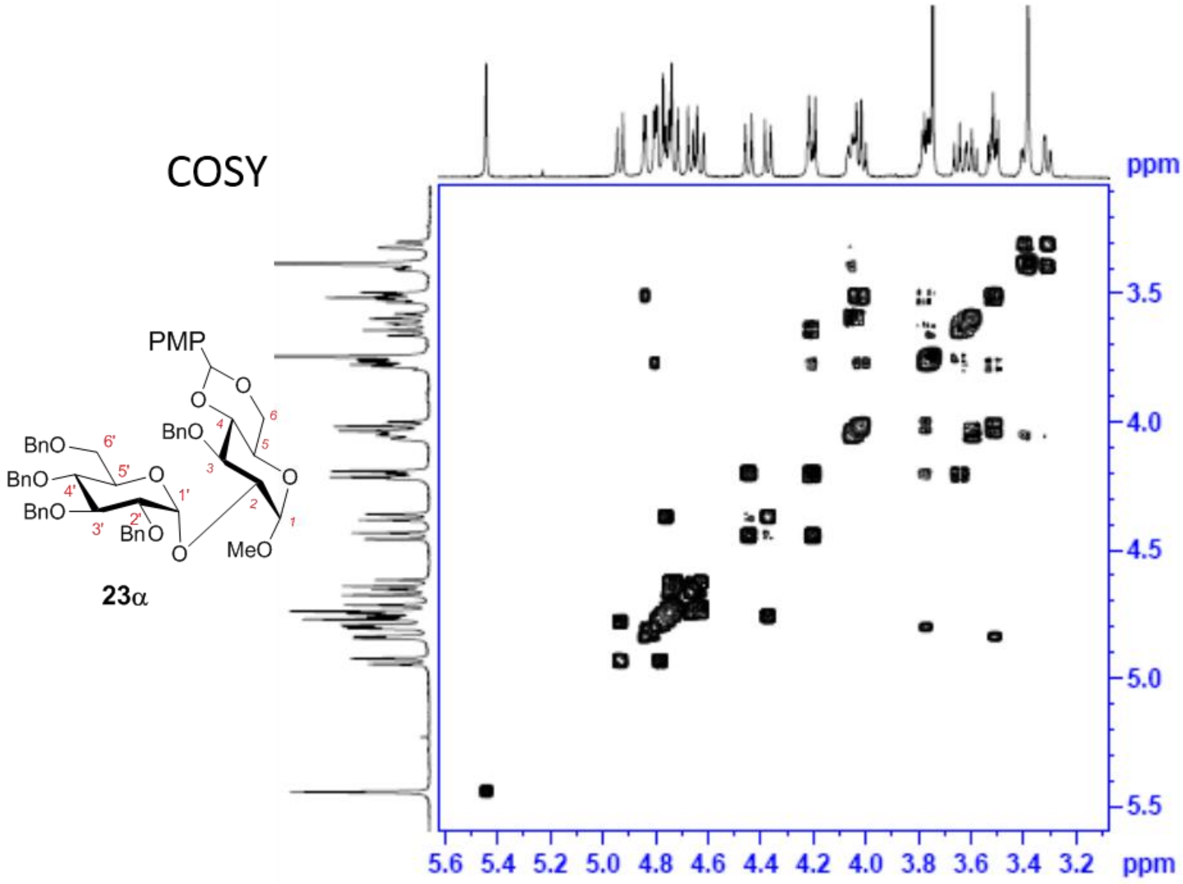

^{13}C NMR of 23α in CDCl$_3$

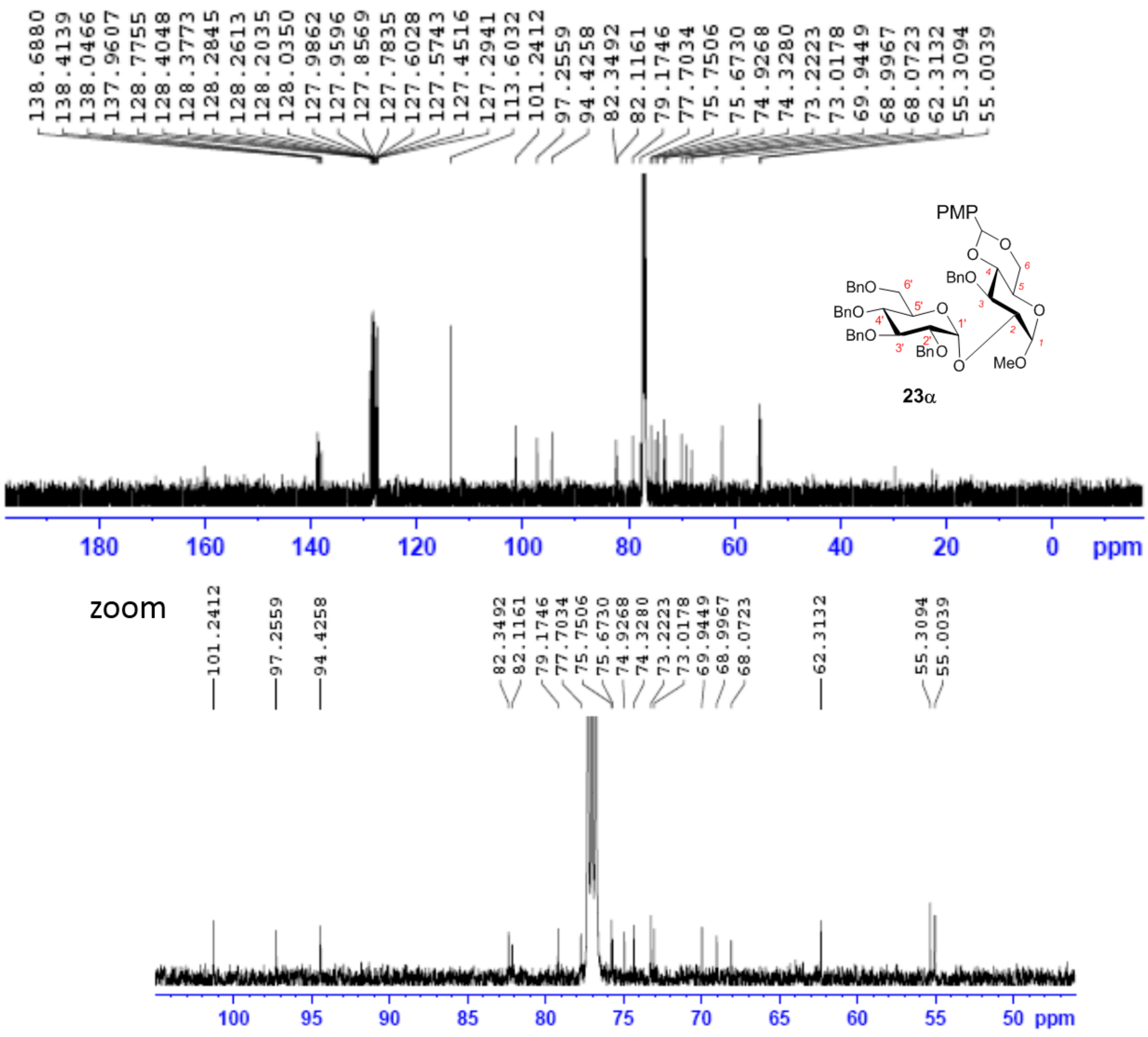

DEPT NMR of 23α in CDCl$_3$

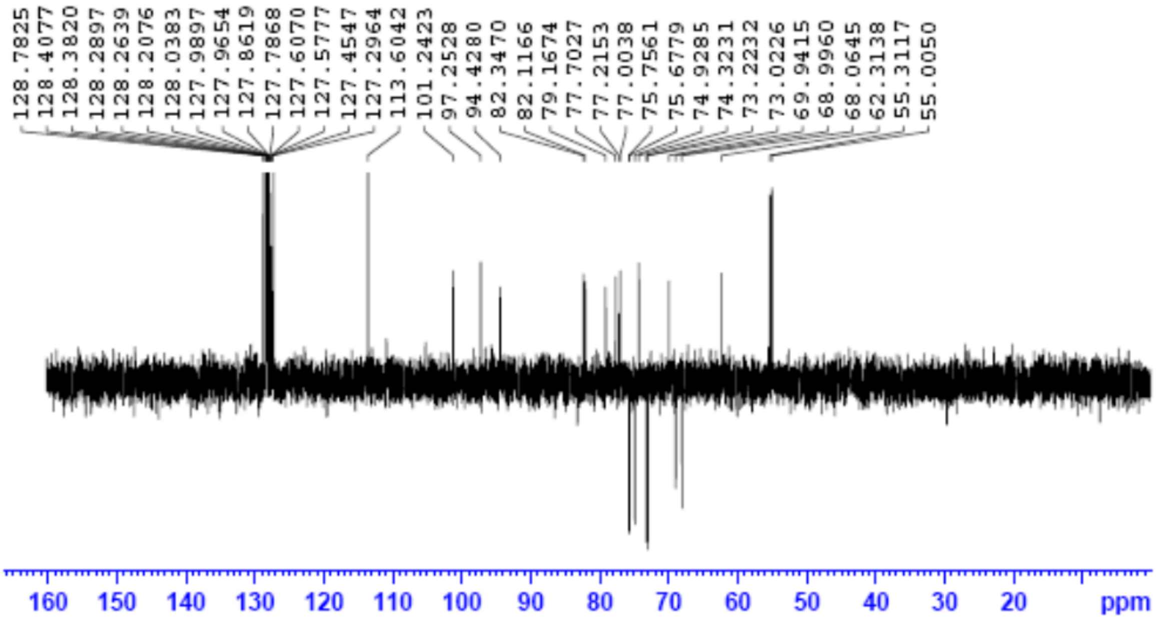

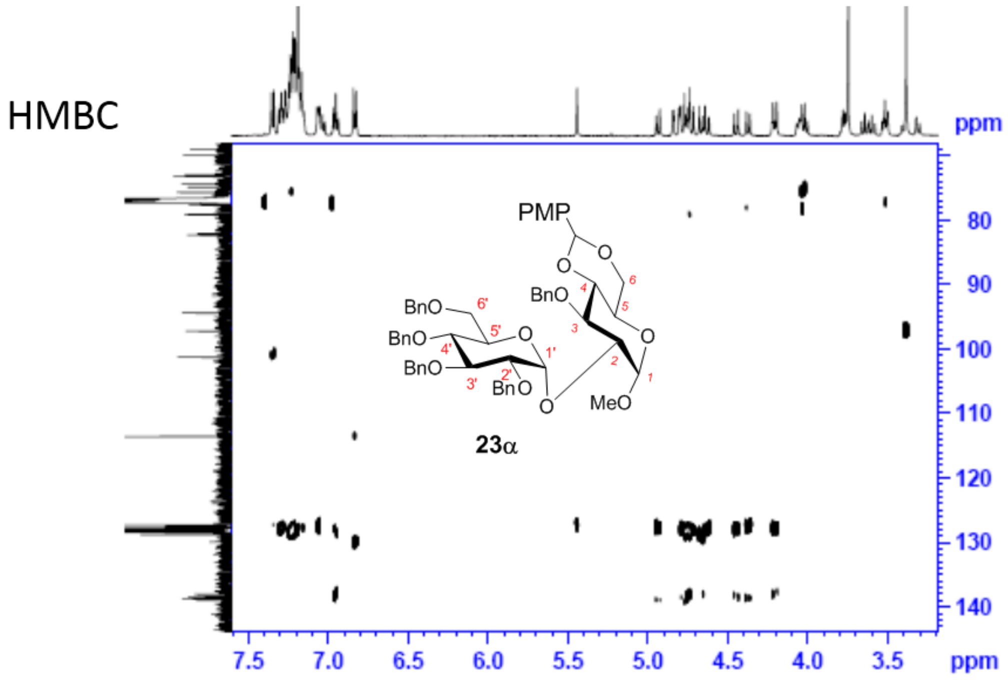

HMQC NMR of 23α in $CDCl_3$

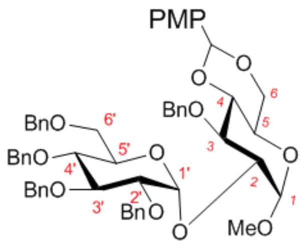

23α

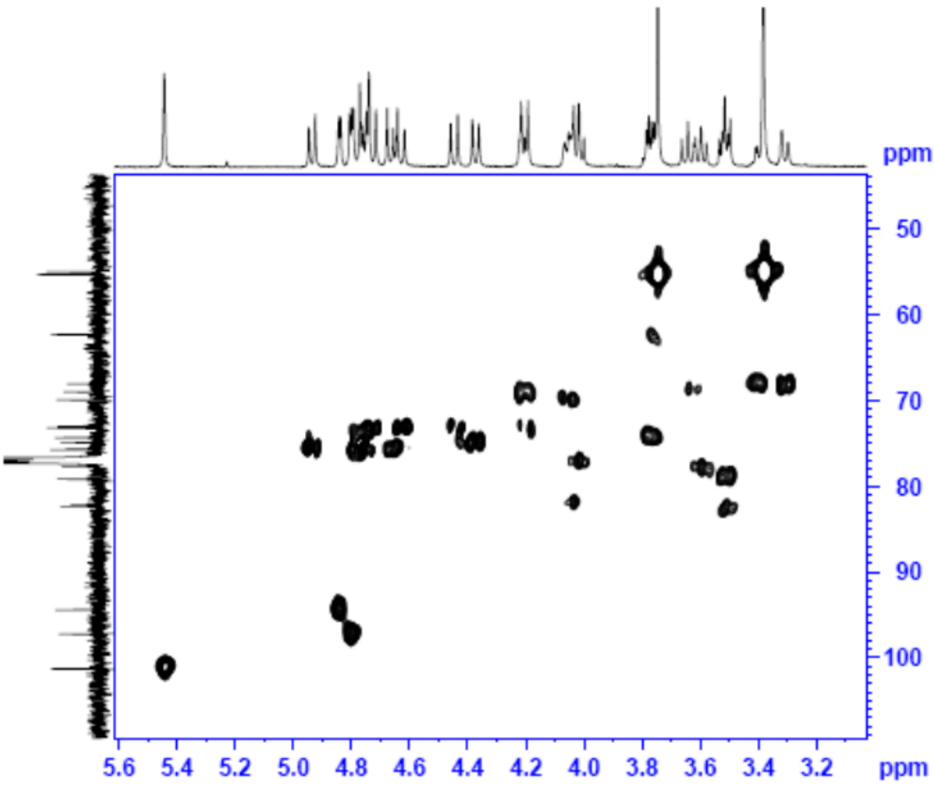

^{1}H NMR of 23β in $CDCl_3$

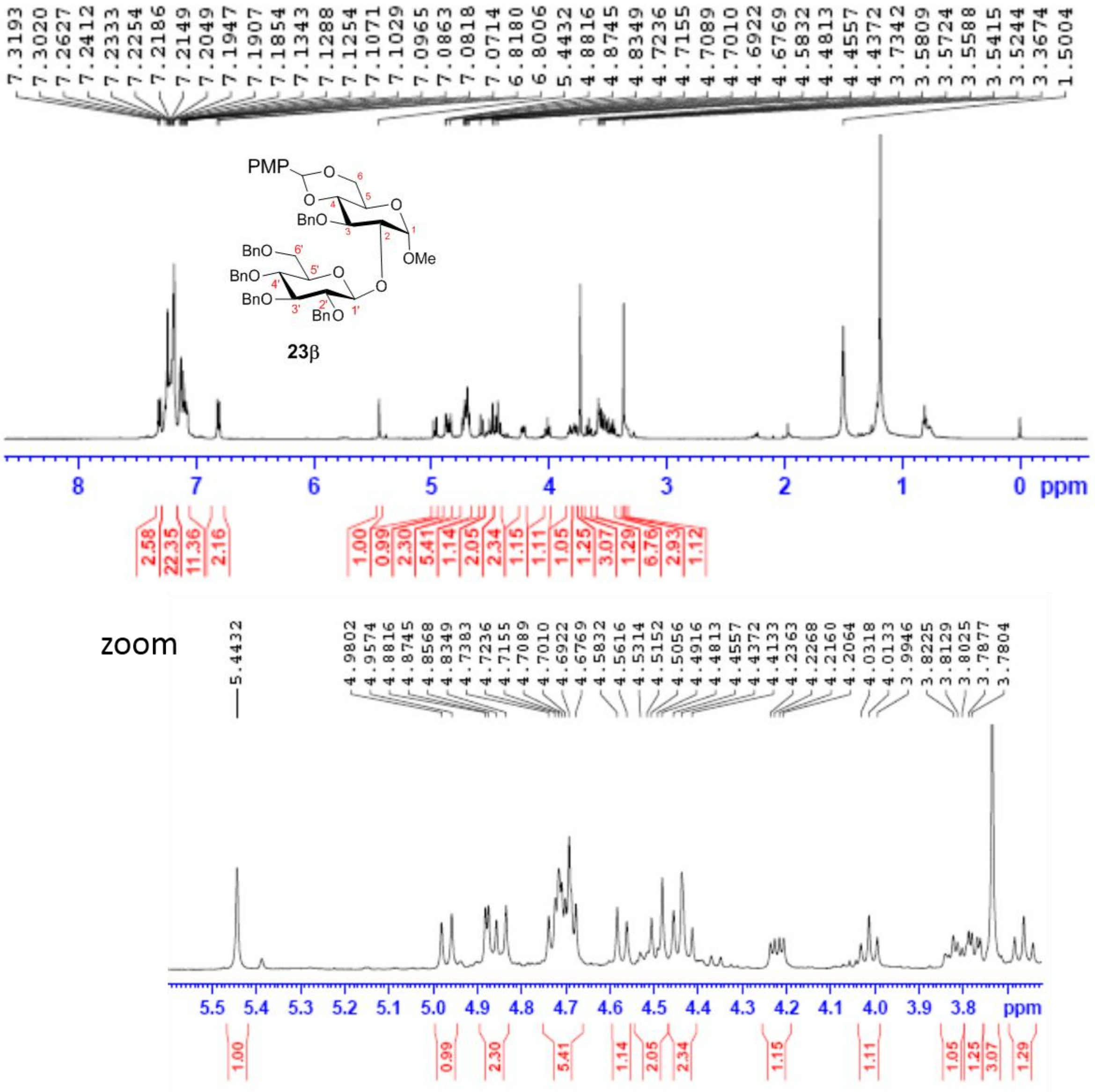

COSY NMR of 23β in $CDCl_3$

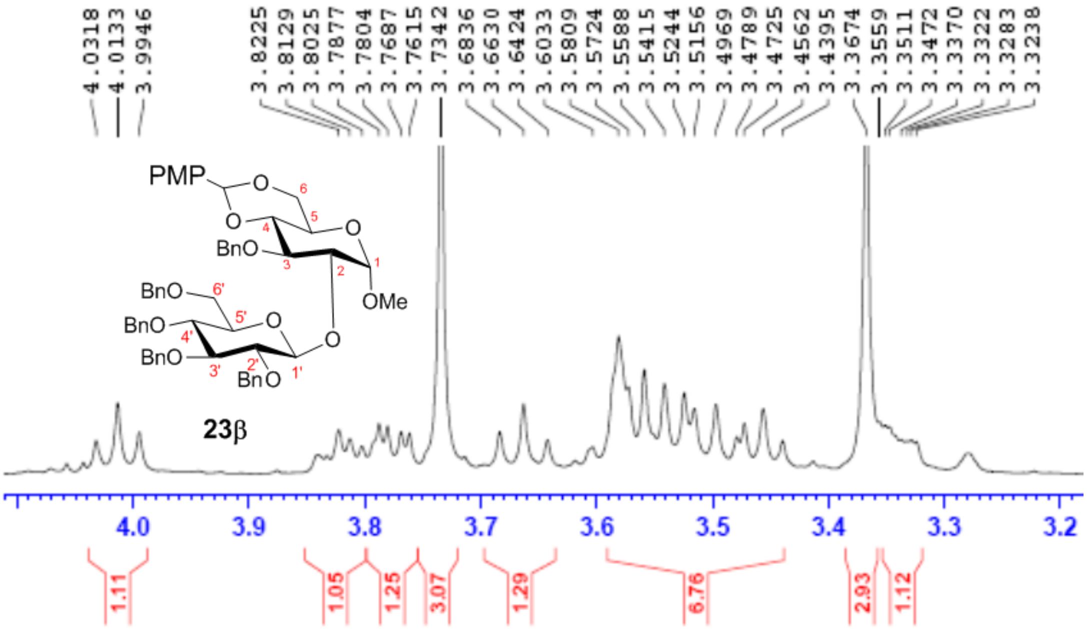

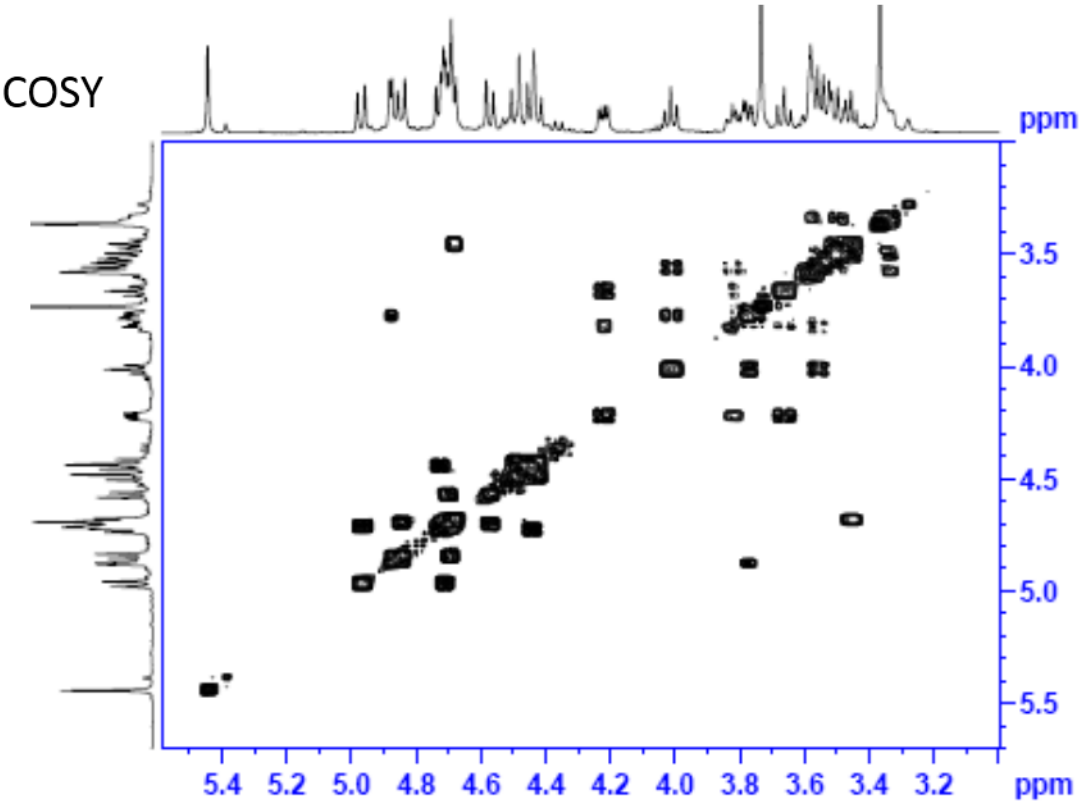

^{13}C NMR of 23β in CDCl$_3$

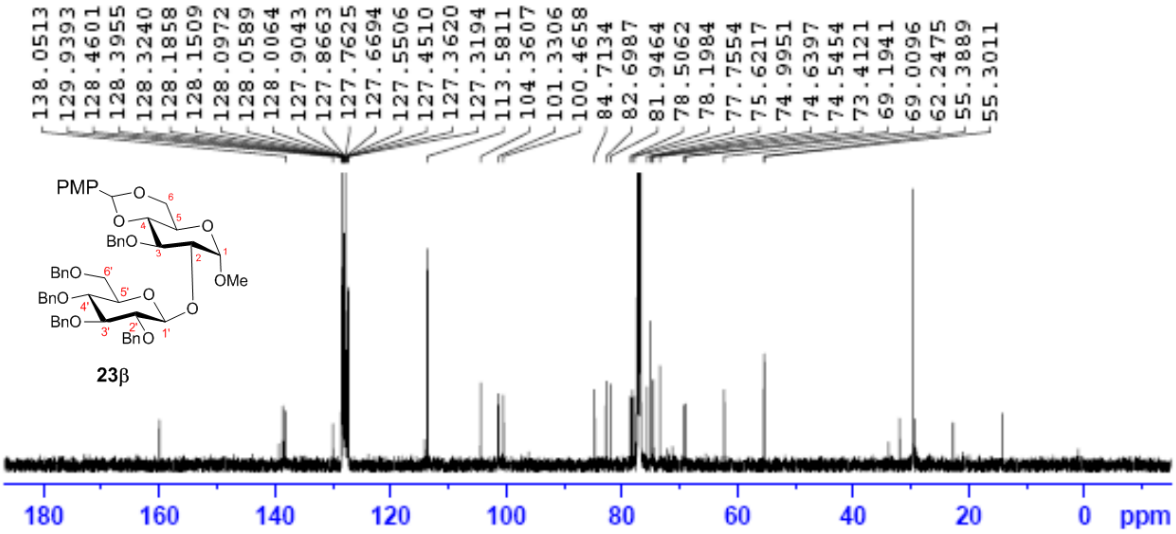

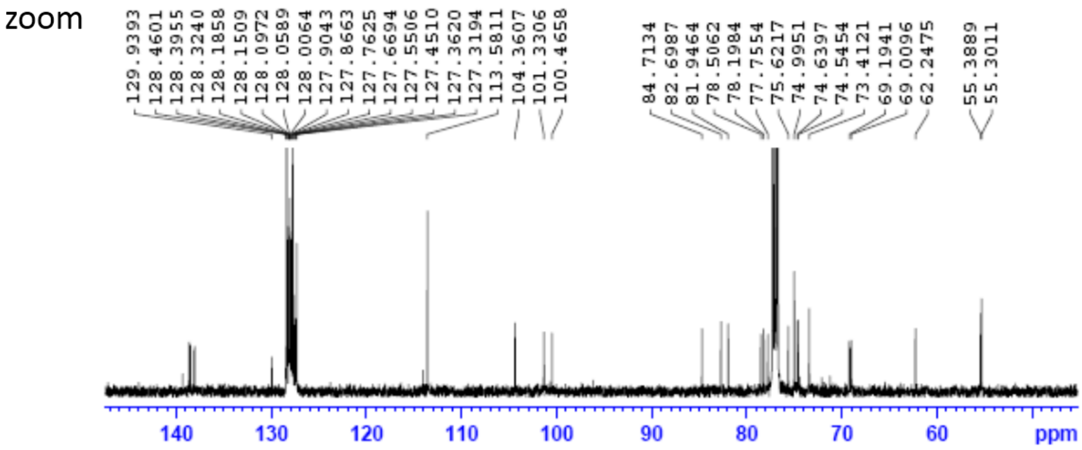

DEPT NMR of 23β in CDCl$_3$

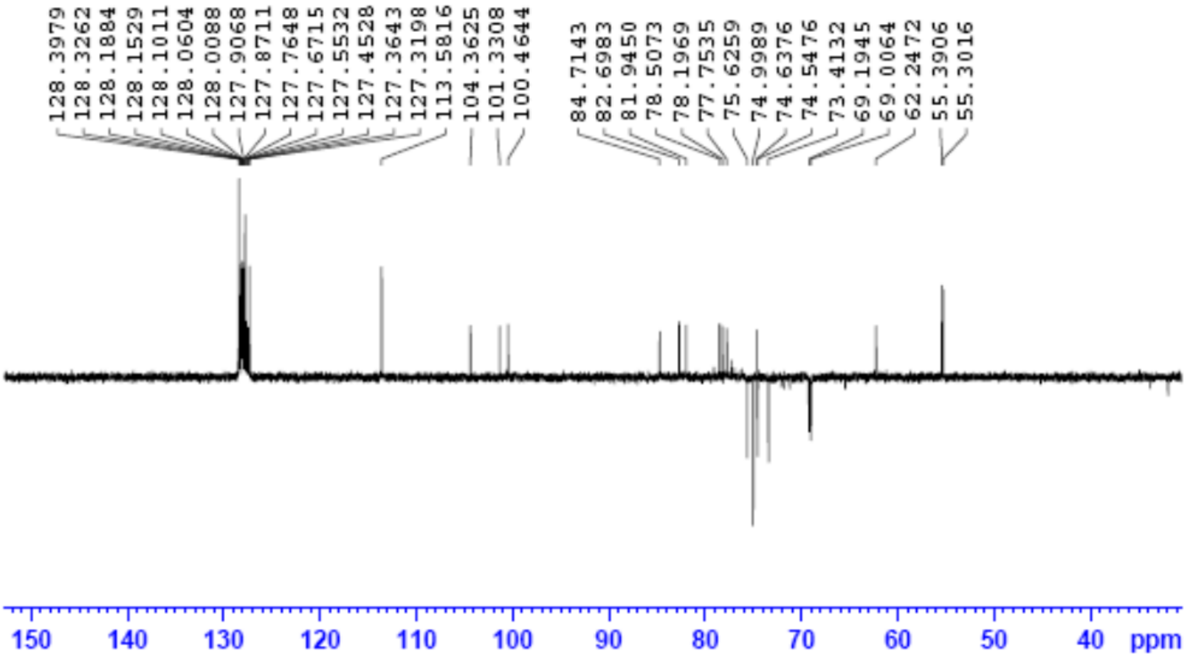

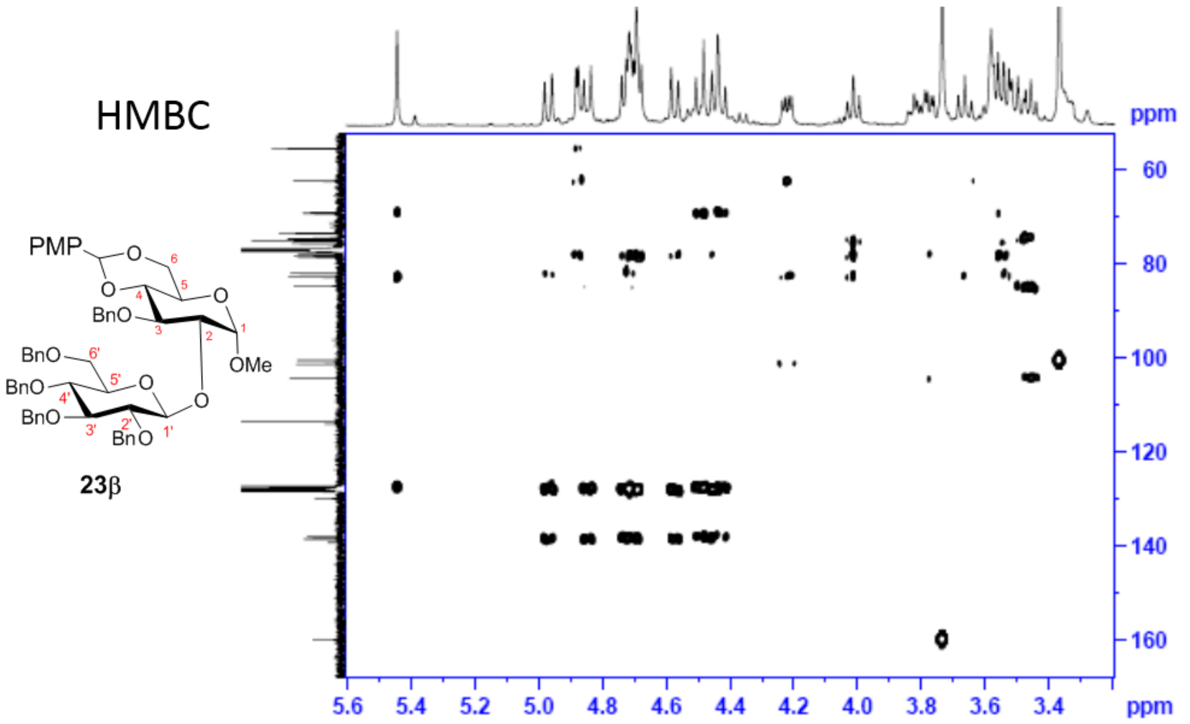

HMQC NMR of 23β in $CDCl_3$

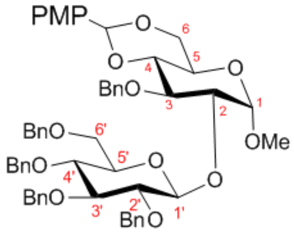

23β

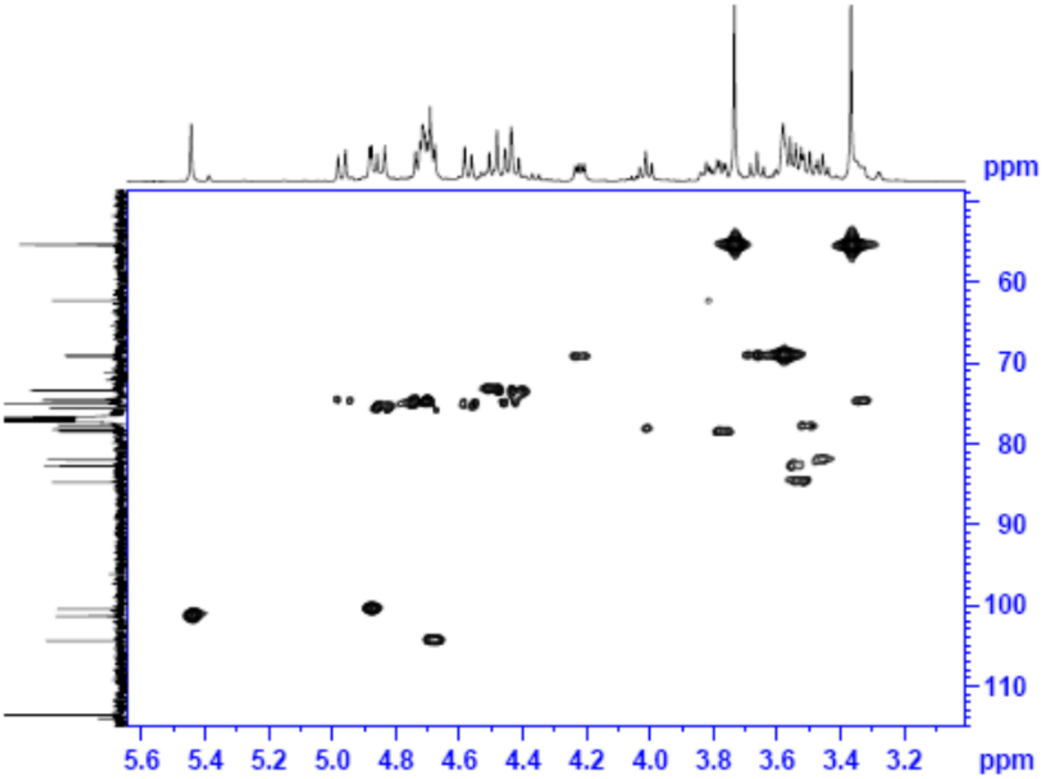

^{1}H NMR of 24α in $CDCl_3$

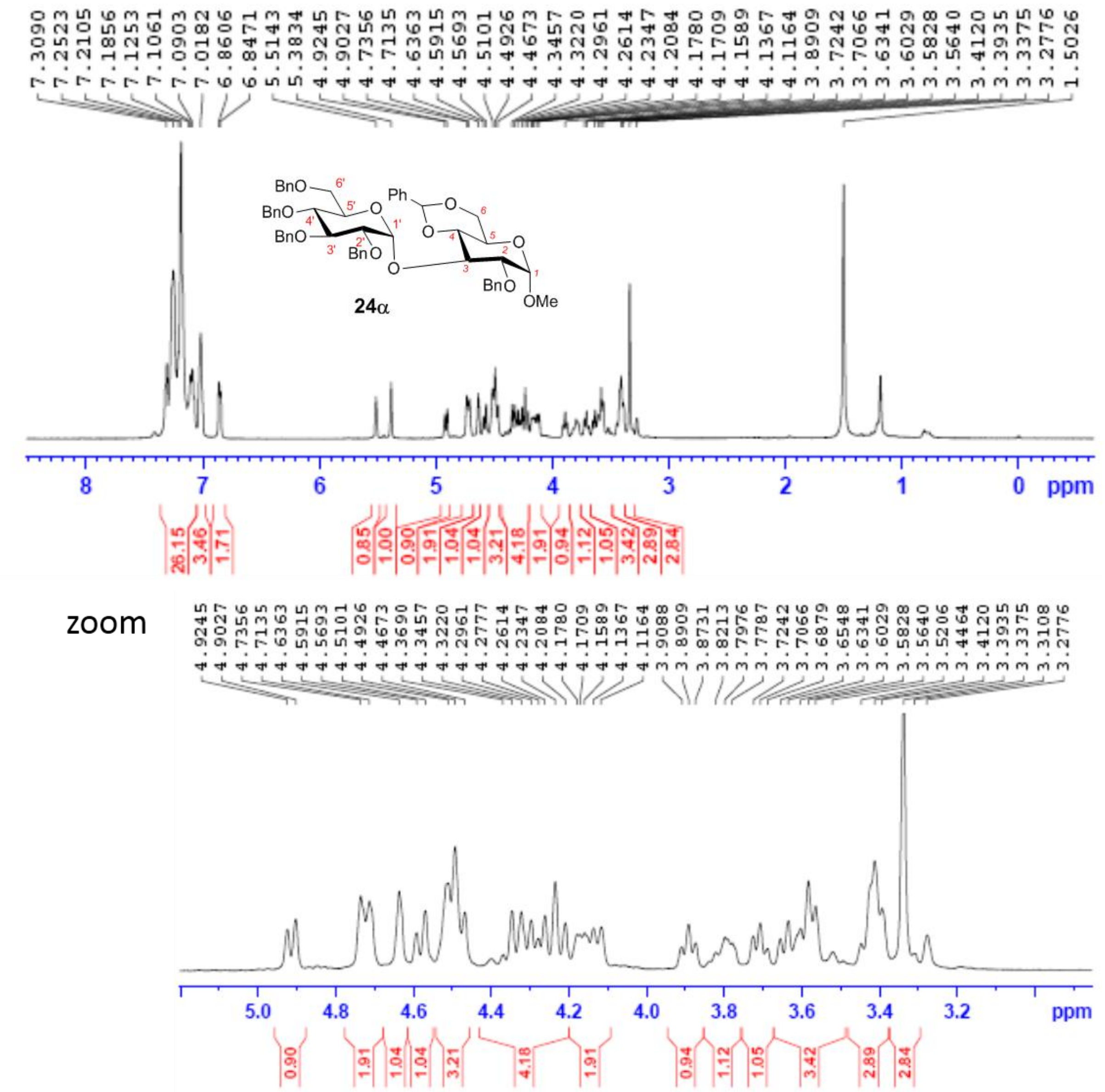

COSY NMR of 24α in CDCl3

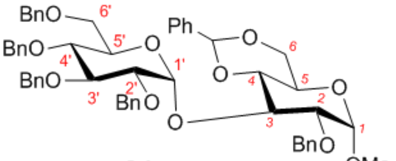

24α

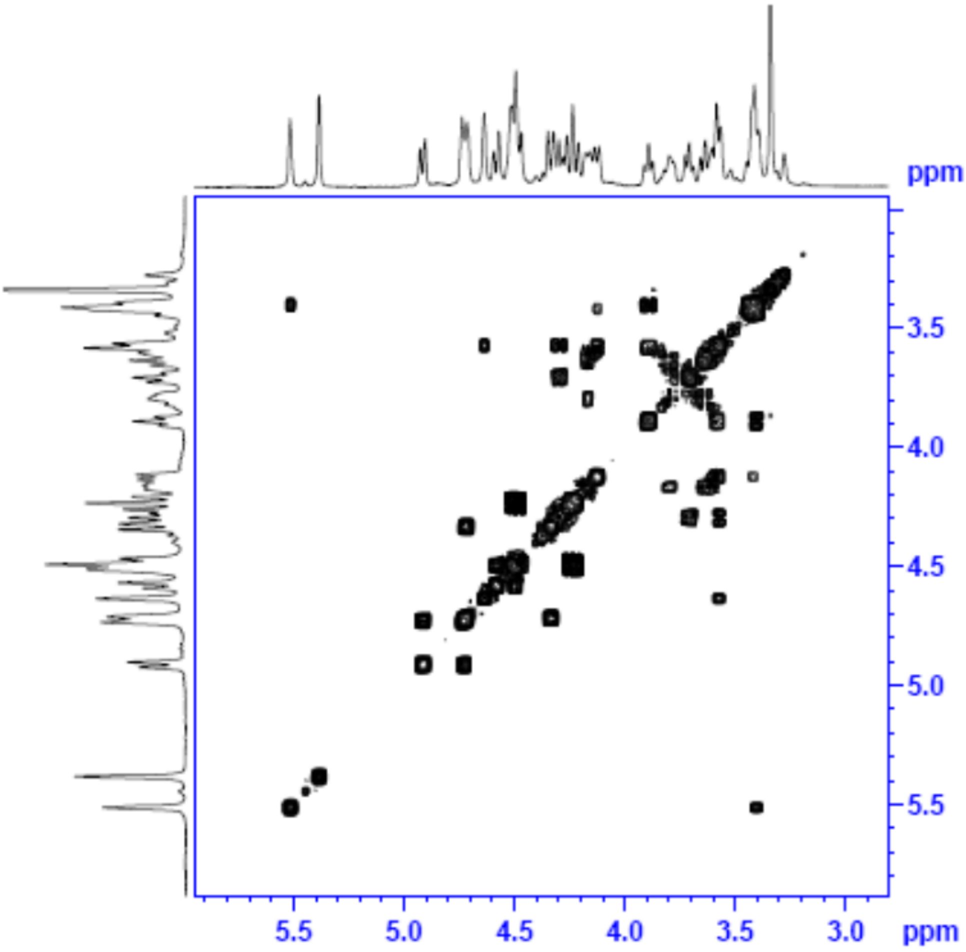

^{13}C NMR of 24α in $CDCl_3$

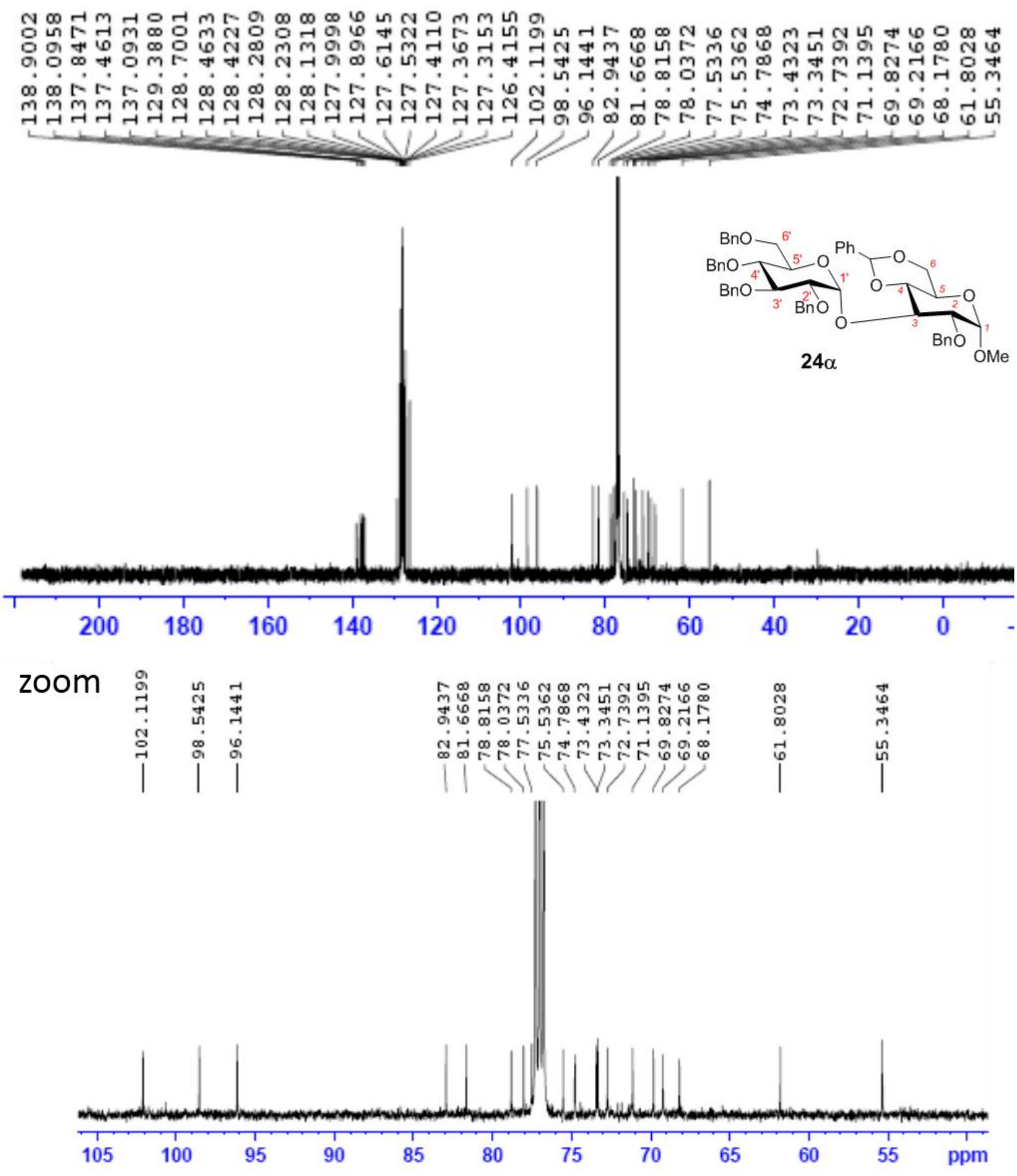

DEPT NMR of 24α in $CDCl_3$

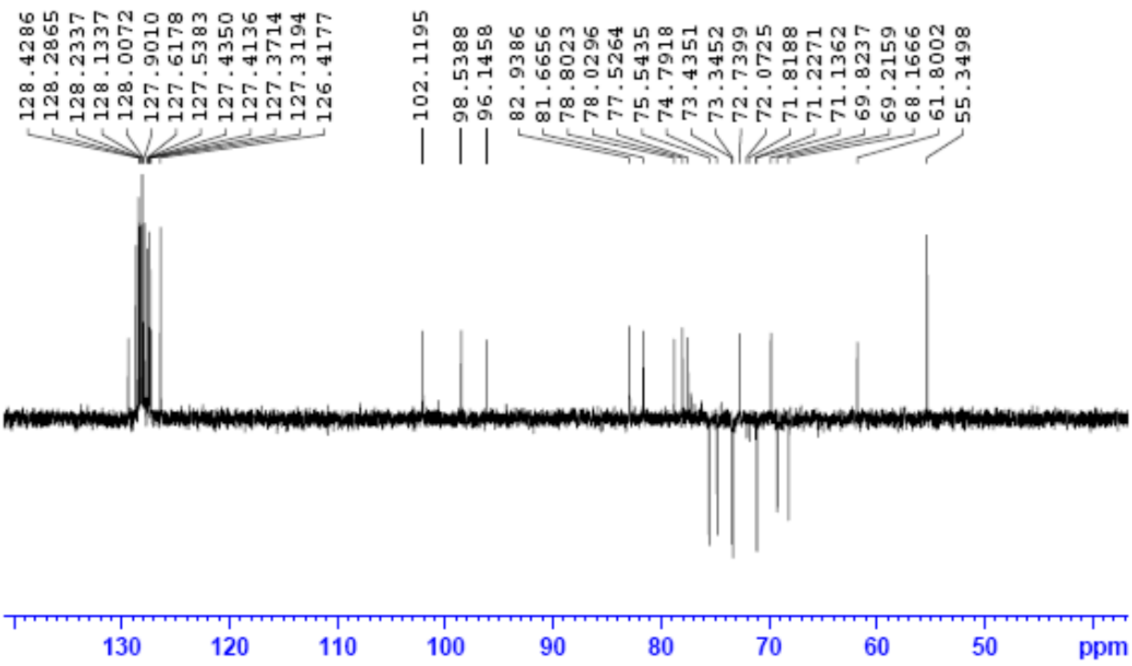

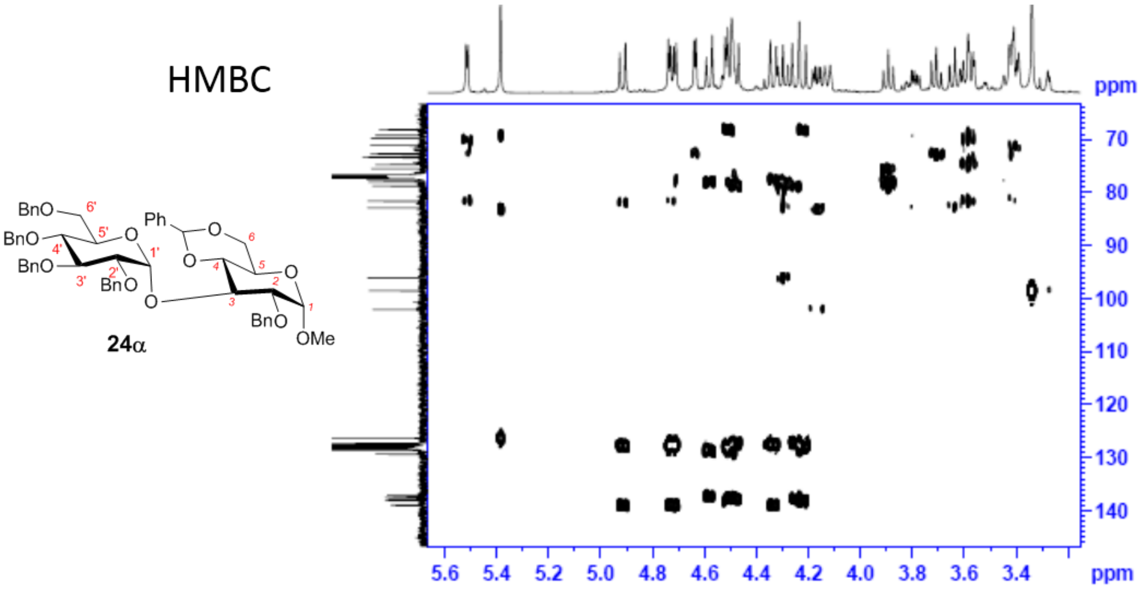

HMQC NMR of 24α in $CDCl_3$

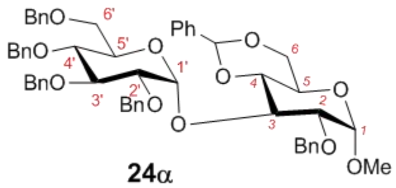

24α

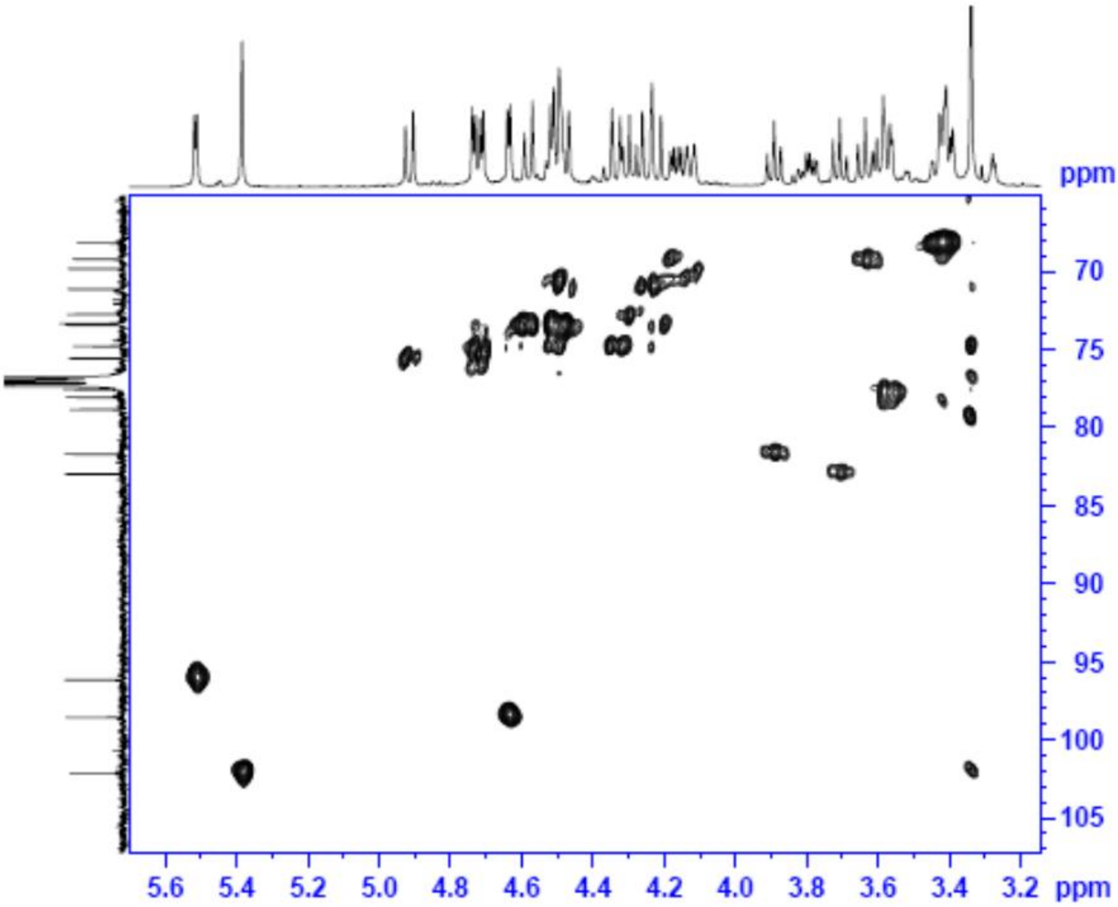

^{1}H NMR of 24β in $CDCl_3$

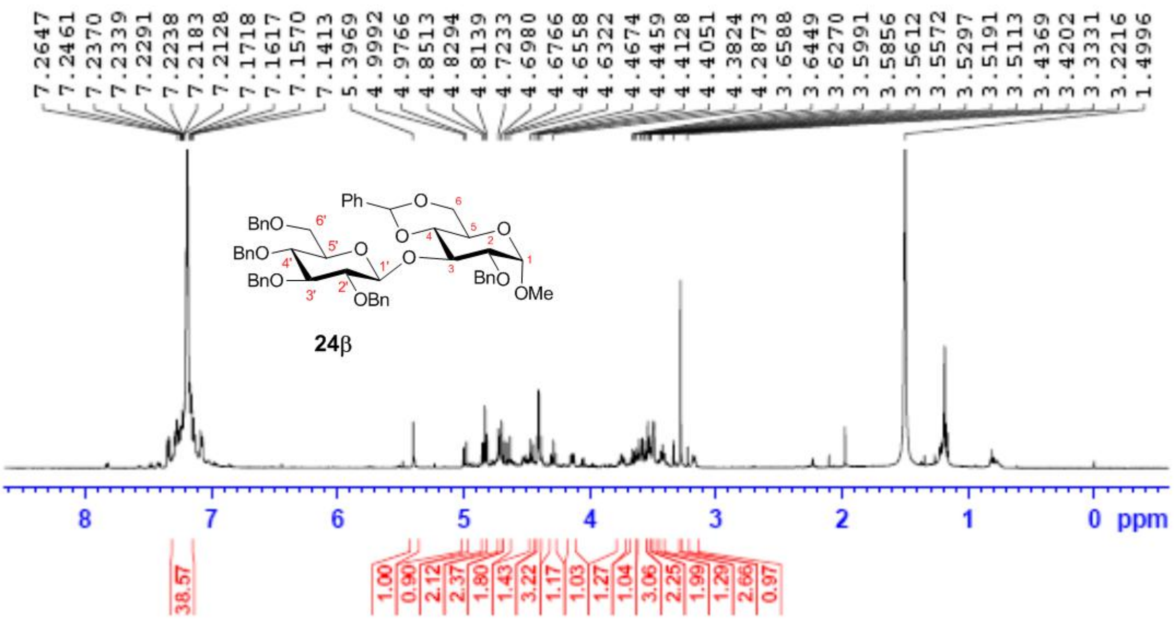

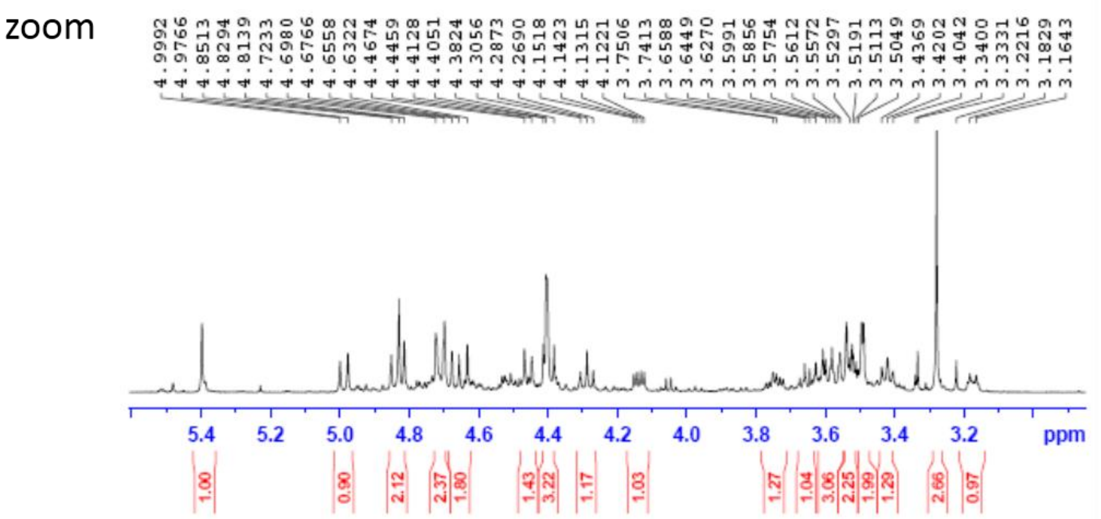

COSY NMR of 24β in $CDCl_3$

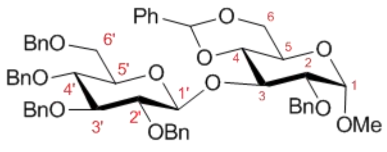

24β

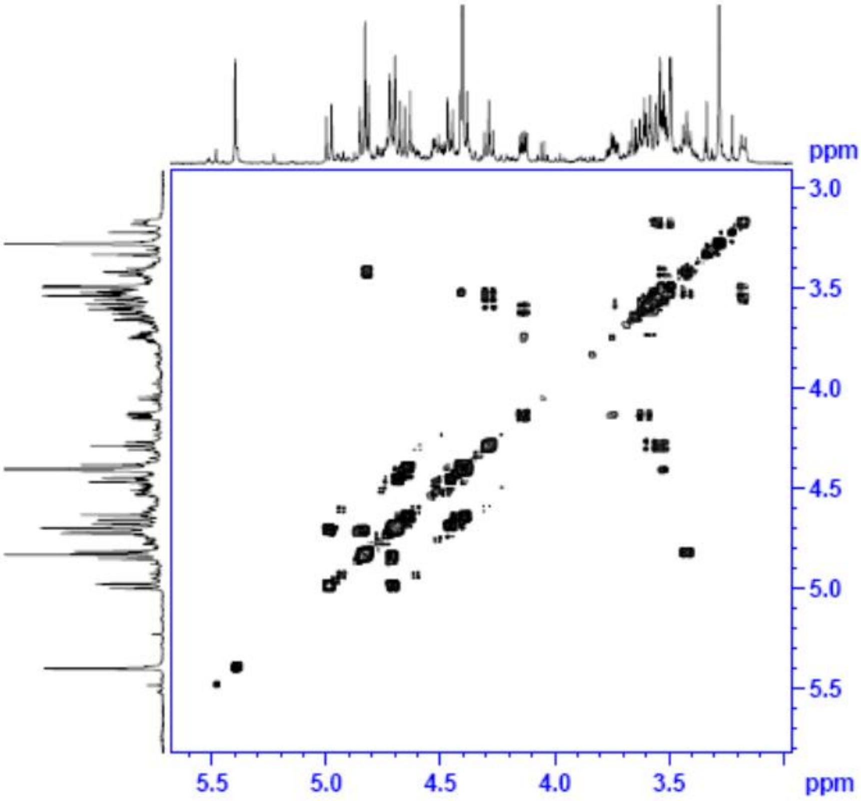

^{13}C NMR of 24β in $CDCl_3$

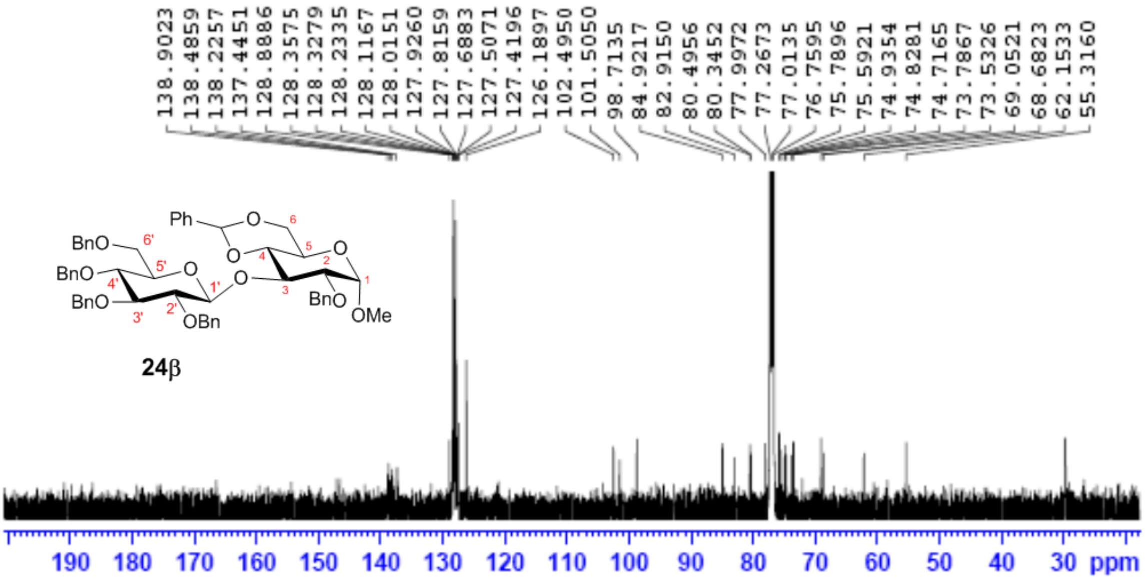

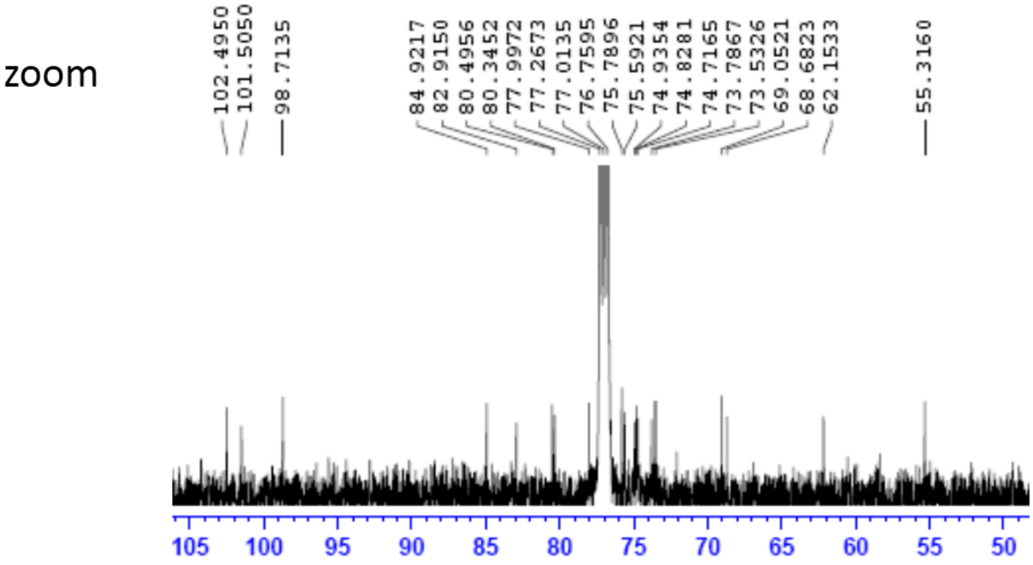

DEPT NMR of 24β in $CDCl_3$

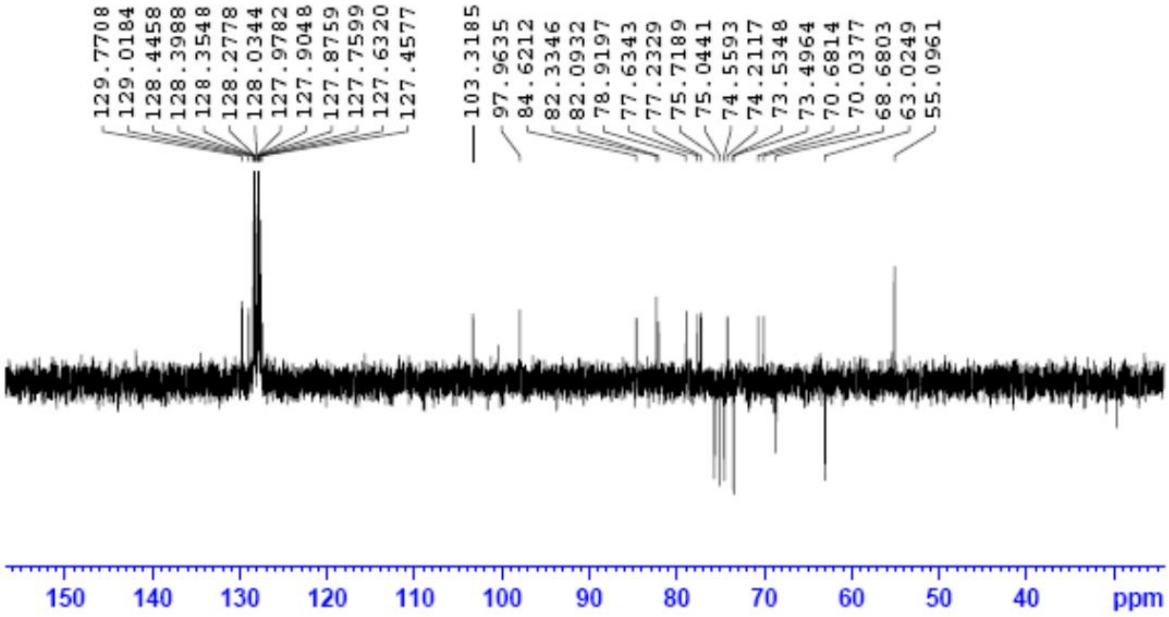

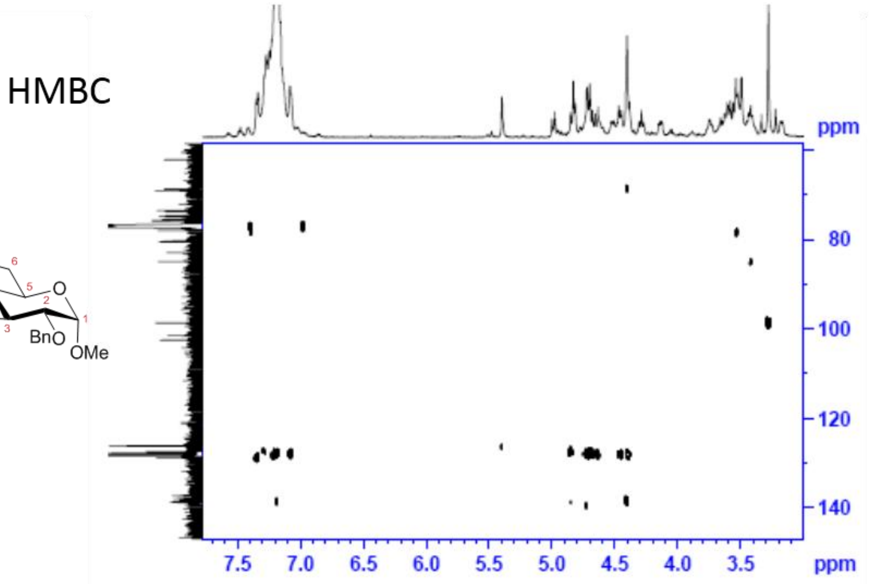

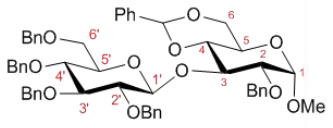

24β

HMQC NMR of 24β in $CDCl_3$

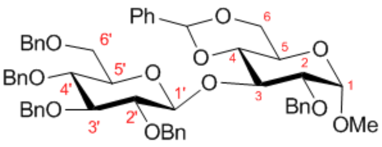

24β

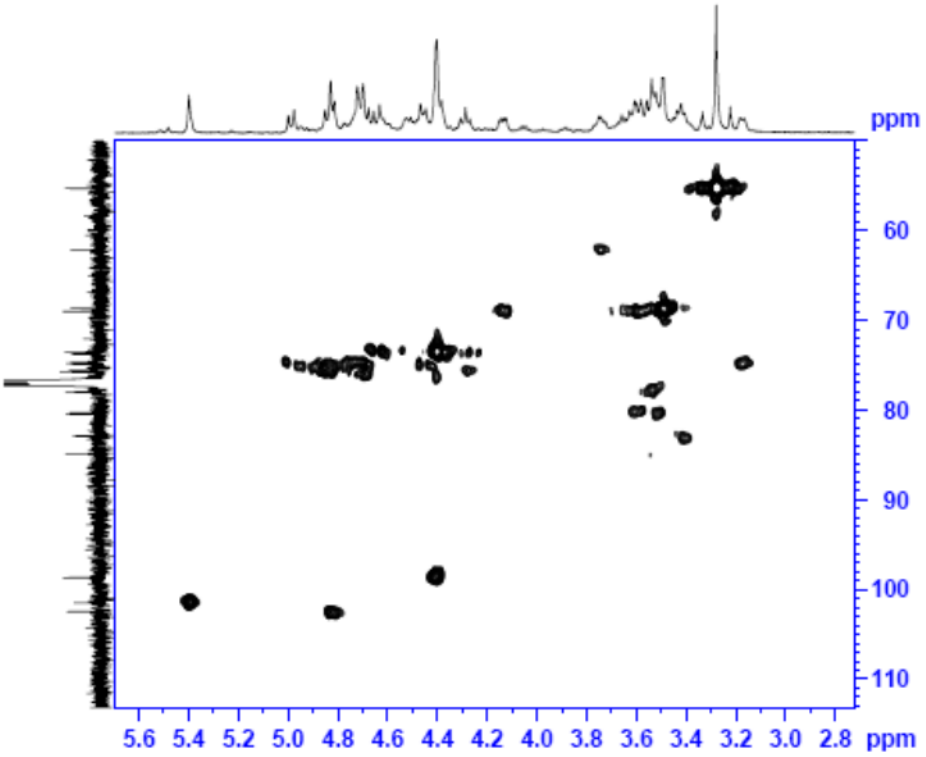

^1H NMR of 25αβ in $CDCl_3$

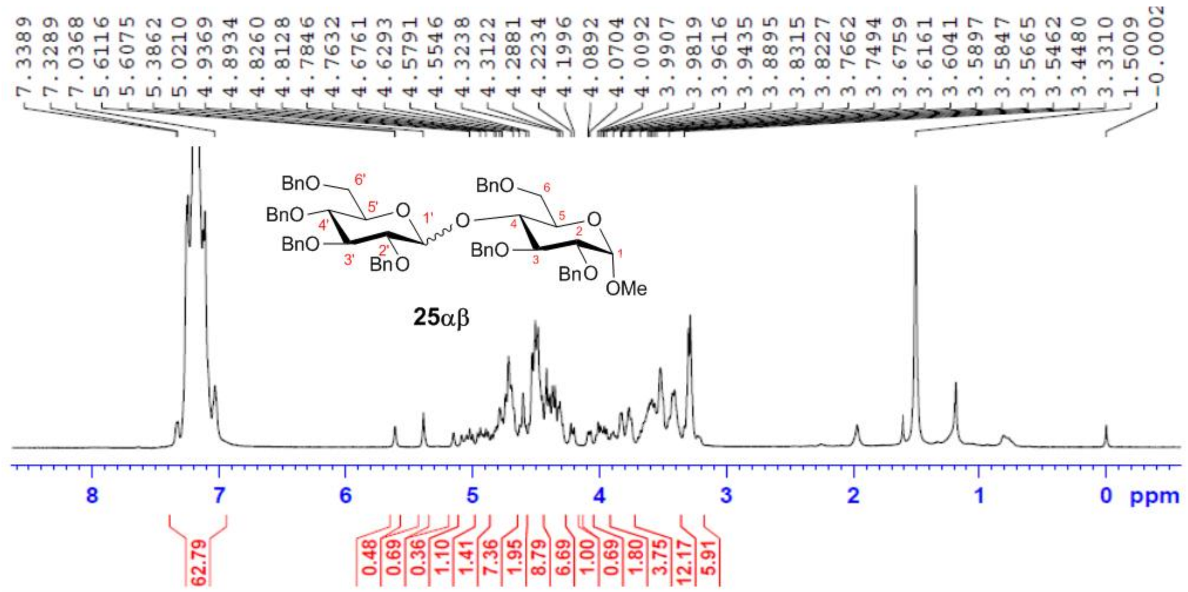

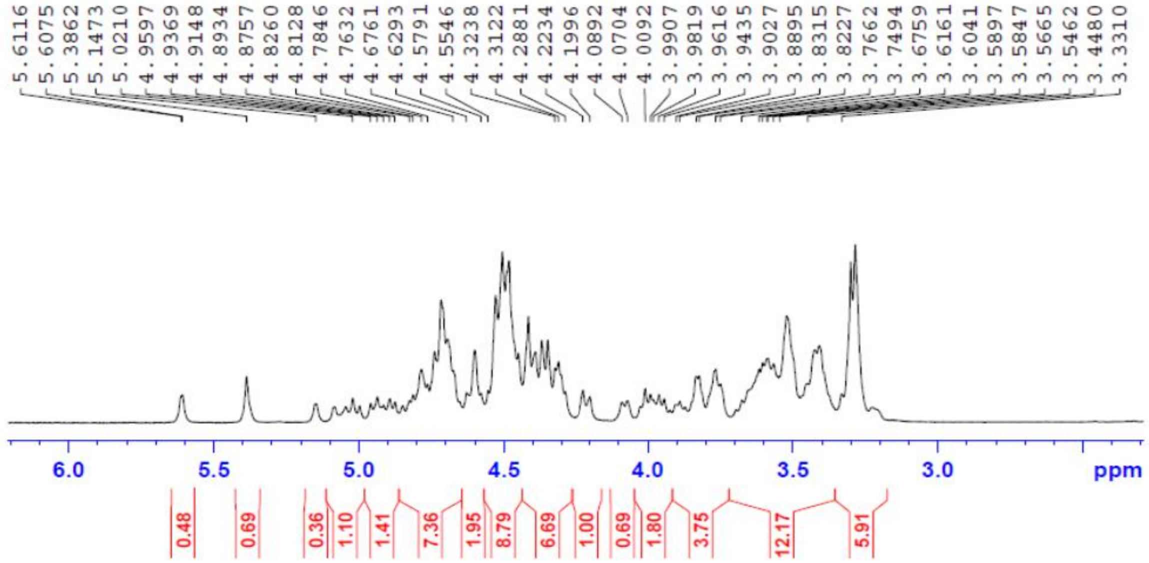

COSY NMR of 25αβ in $CDCl_3$

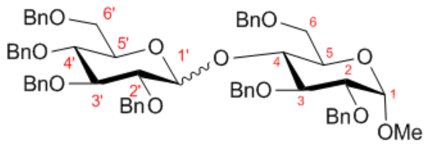

25αβ

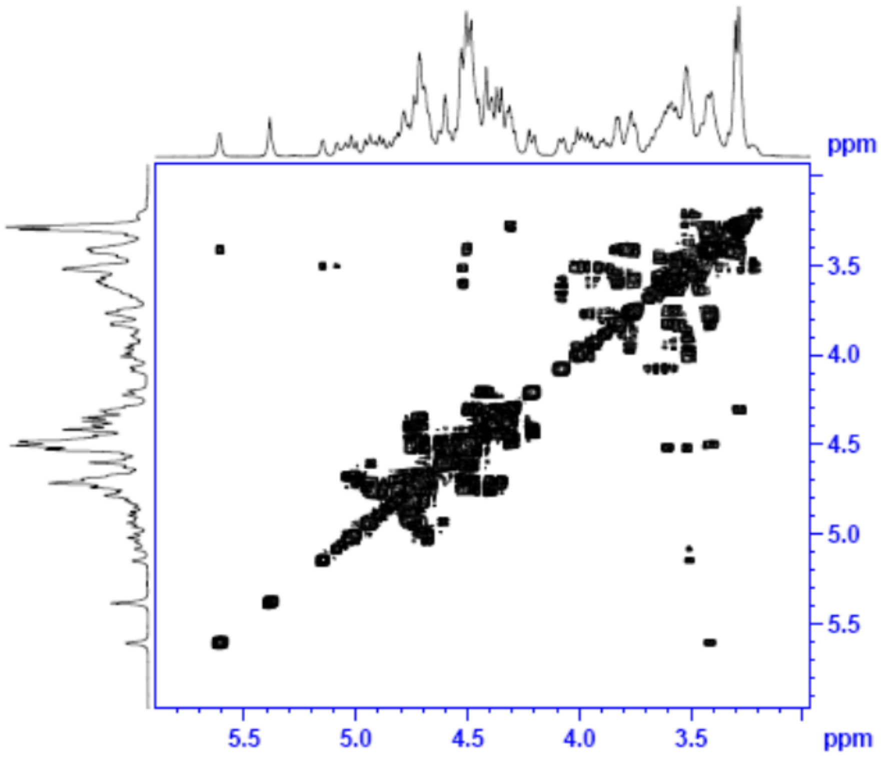

^{1}H NMR of 26αβ in $CDCl_3$

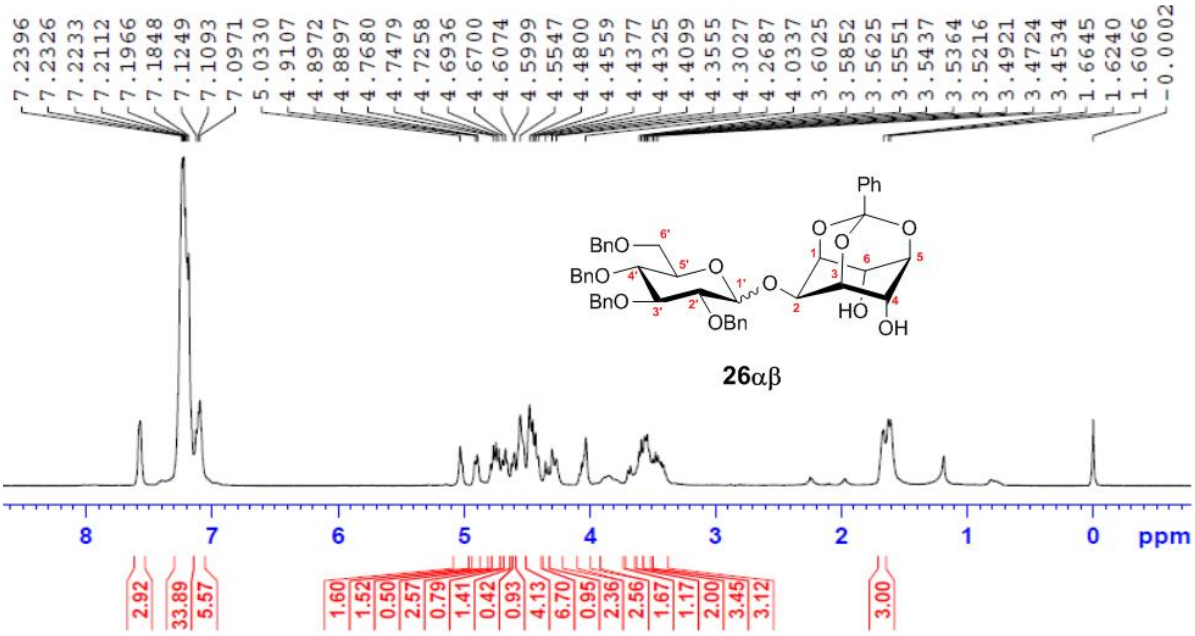

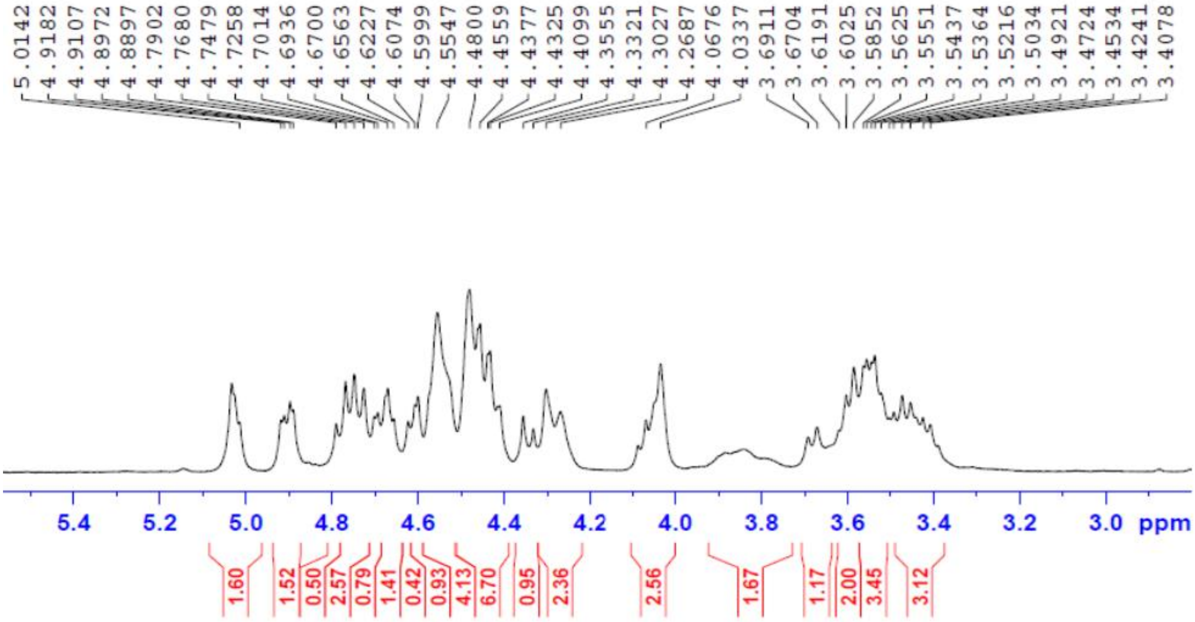

COSY NMR of 26αβ in $CDCl_3$

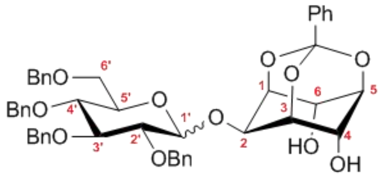

26αβ

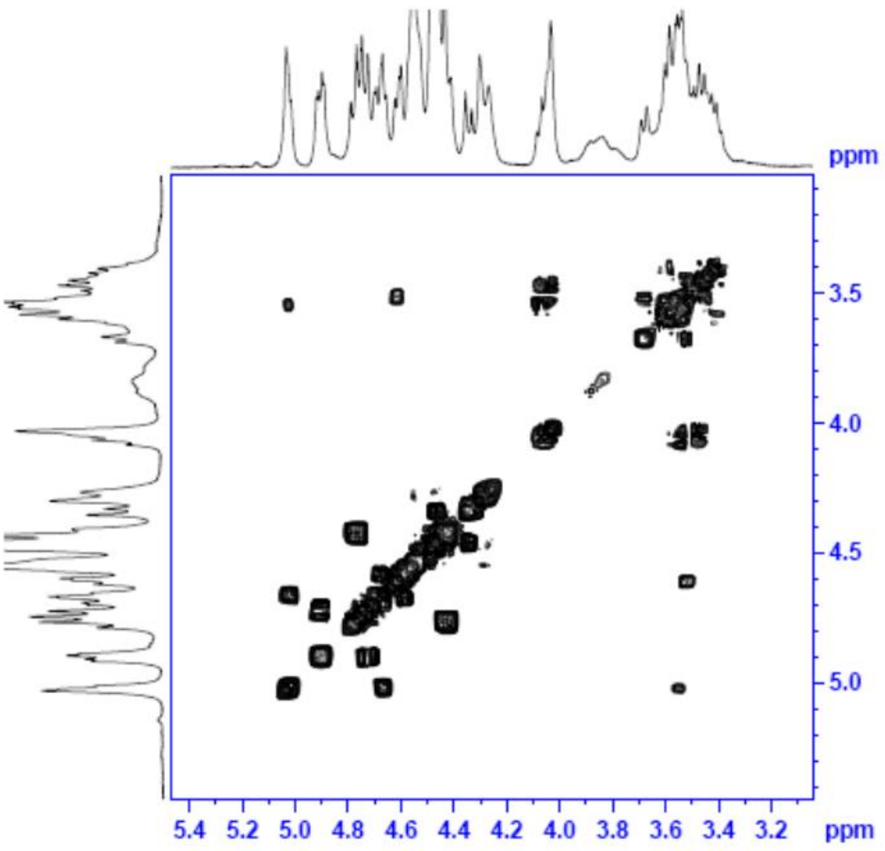

^{13}C NMR of 26αβ in $CDCl_3$

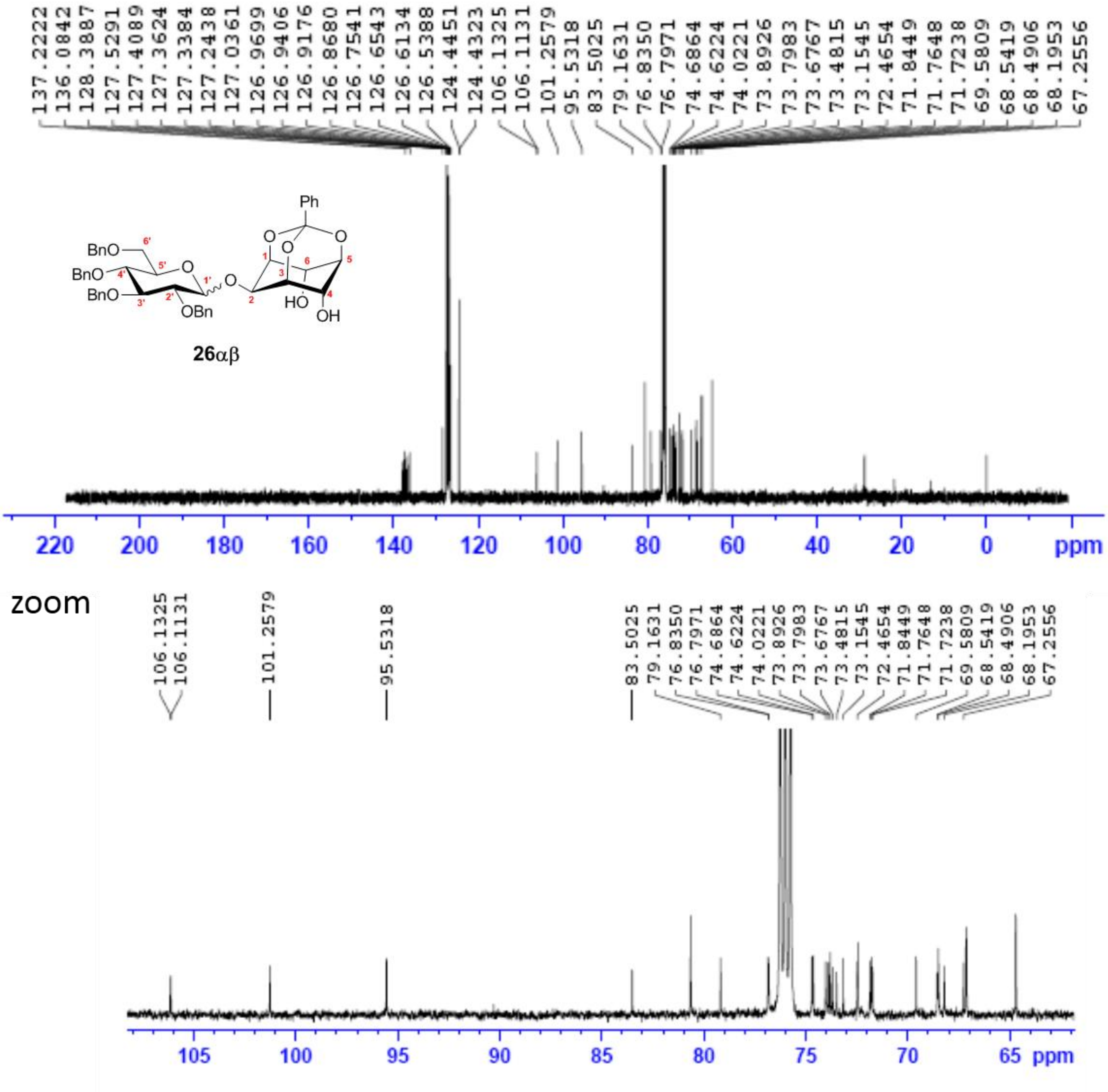

DEPT NMR of 26αβ in $CDCl_3$

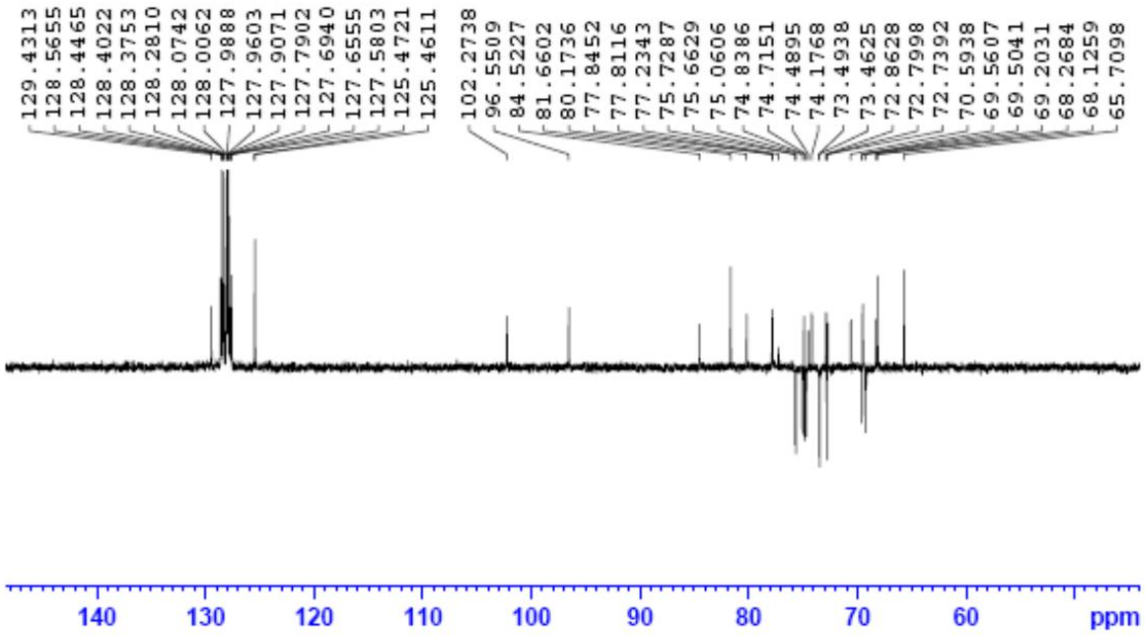

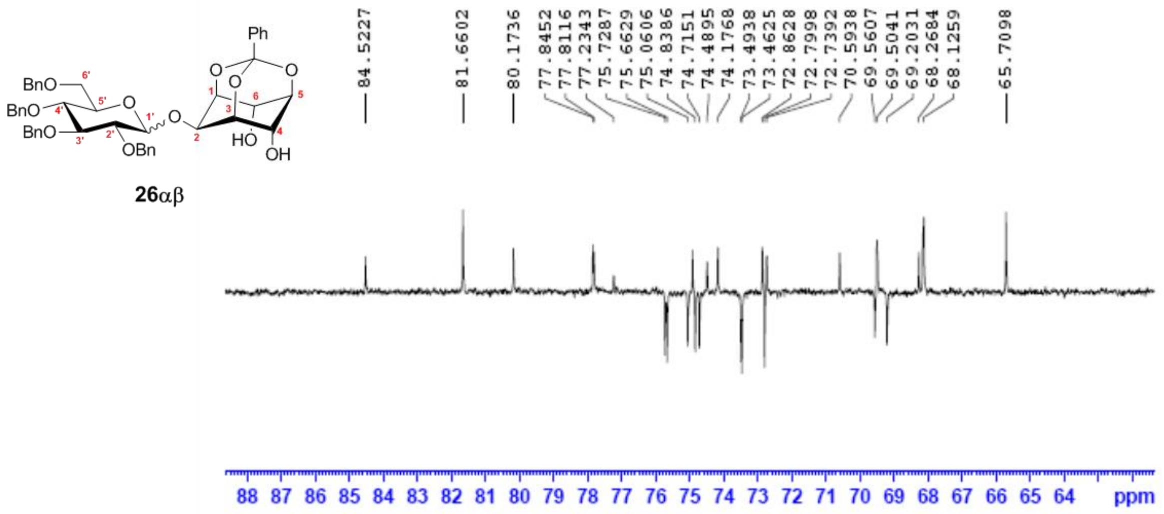

26αβ

HMBC NMR of 26αβ in $CDCl_3$

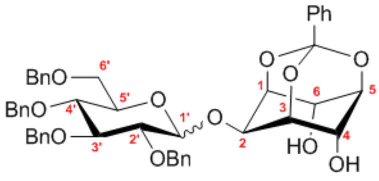

26αβ

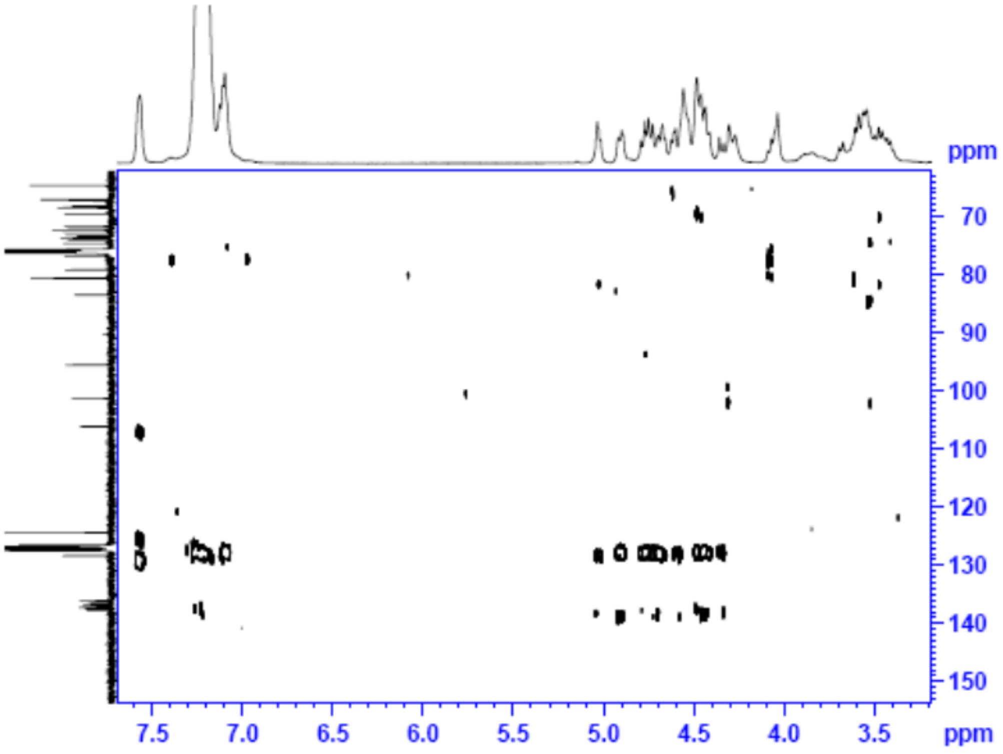

HMQC NMR of 26αβ in $CDCl_3$

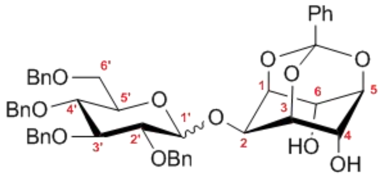

26αβ

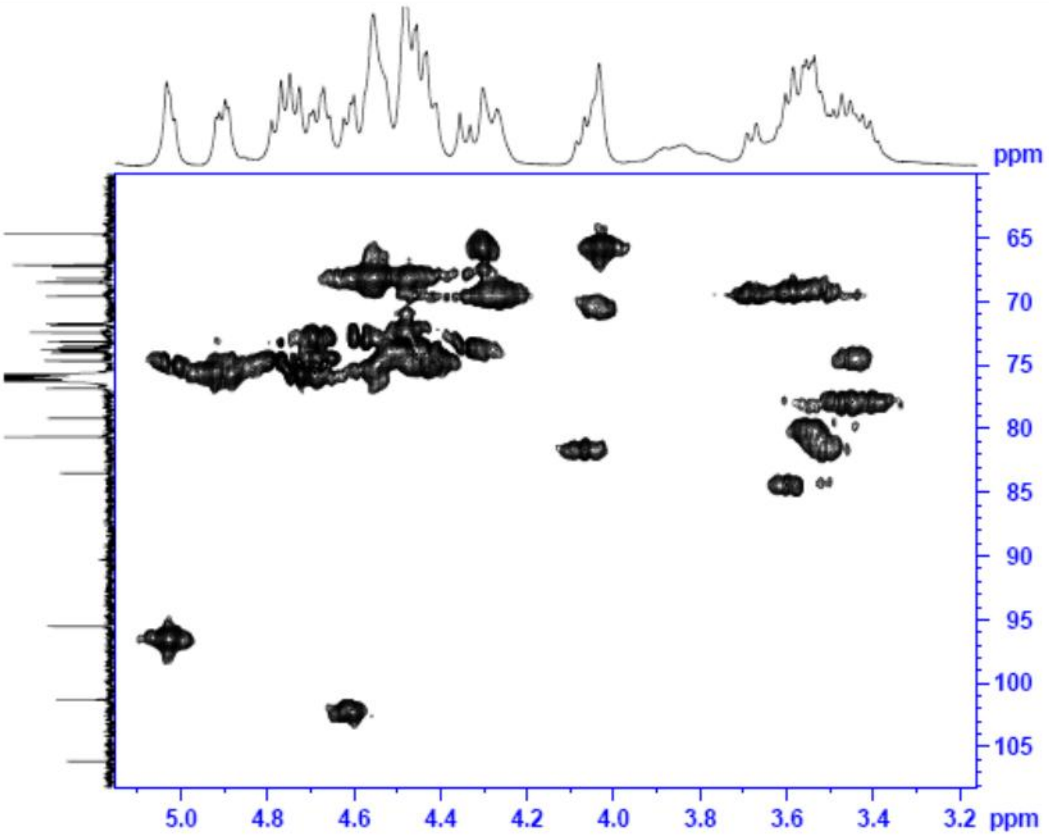

^{1}H NMR of D12 in $CDCl_3$

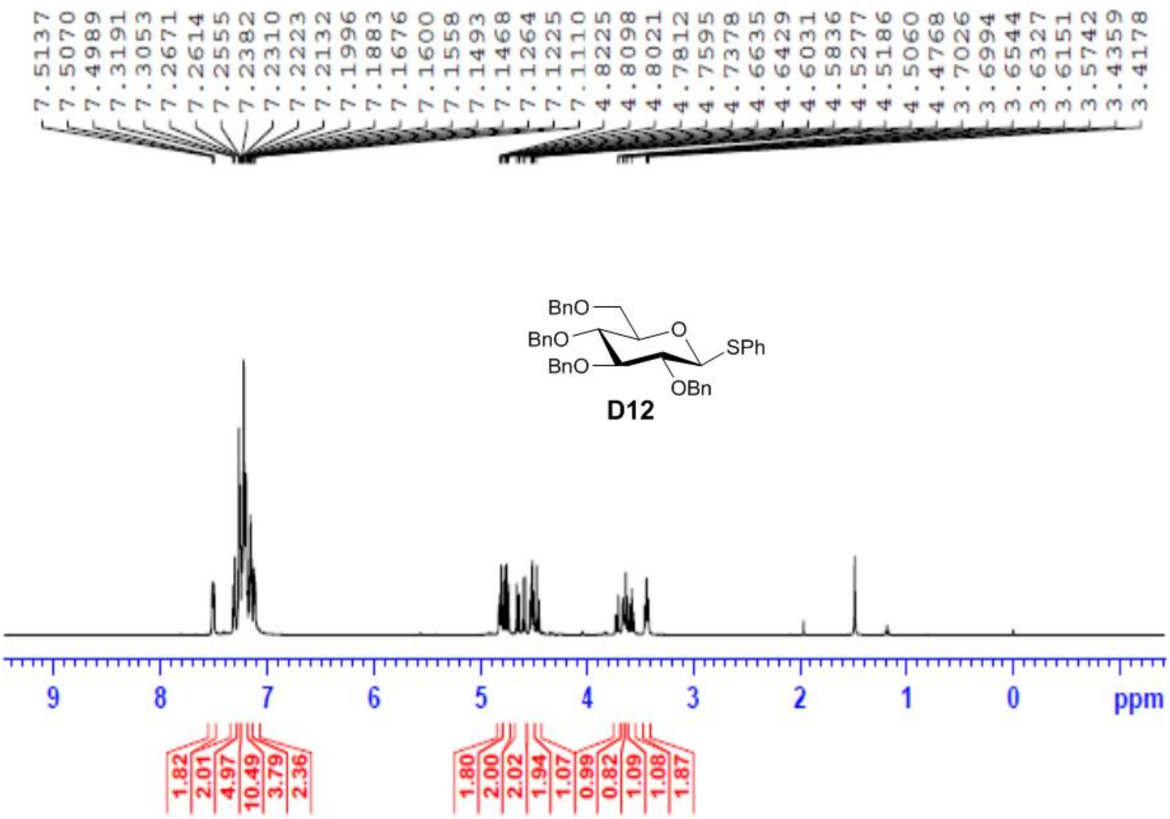

^{1}H NMR of D12 in $CDCl_3$ (zoom)

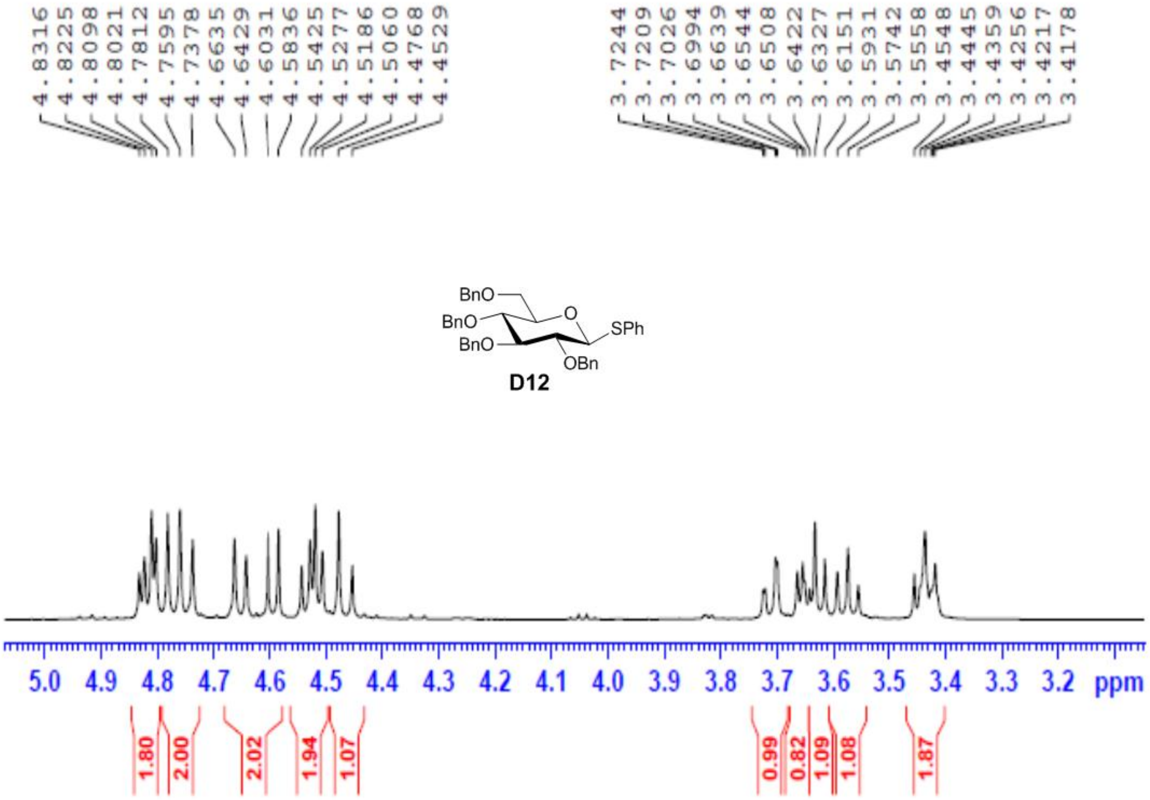

Cosy NMR of D12 in $CDCl_3$

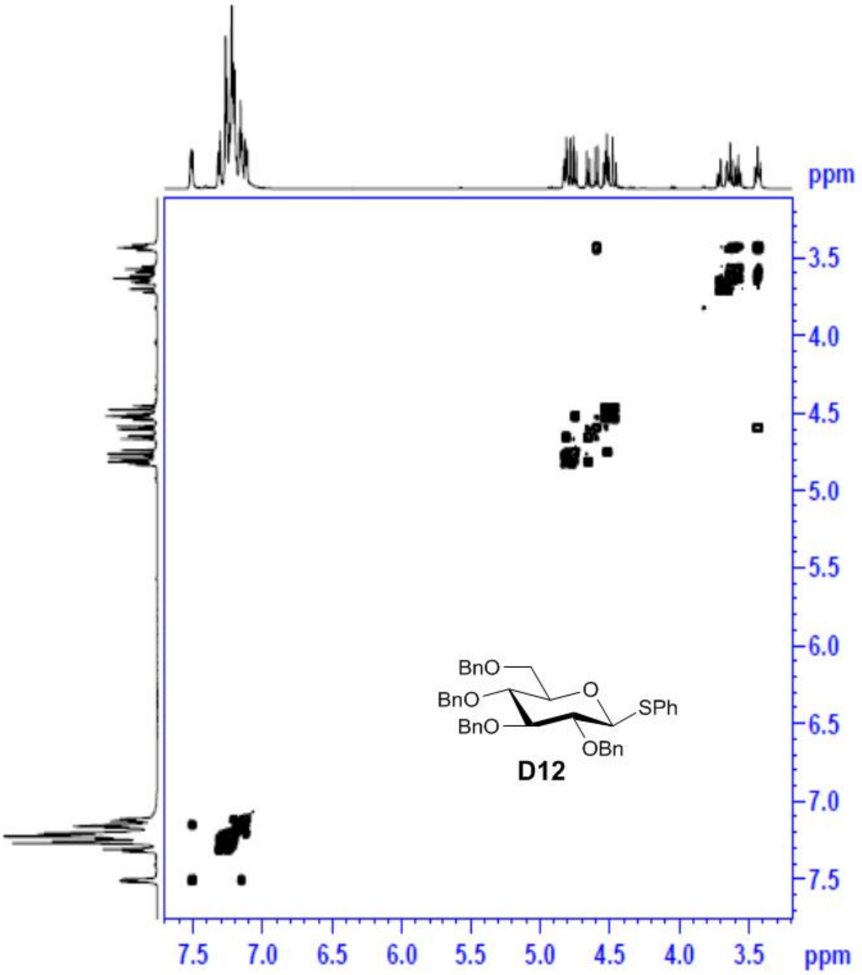

^{1}H NMR of 27αβ in $CDCl_3$

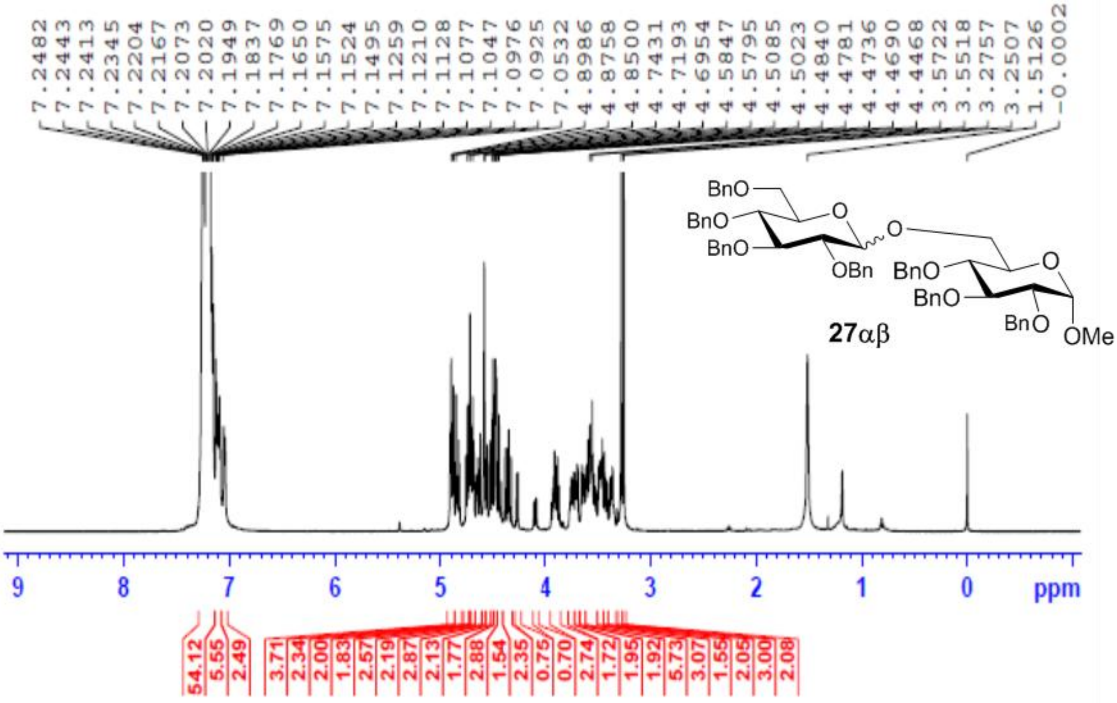

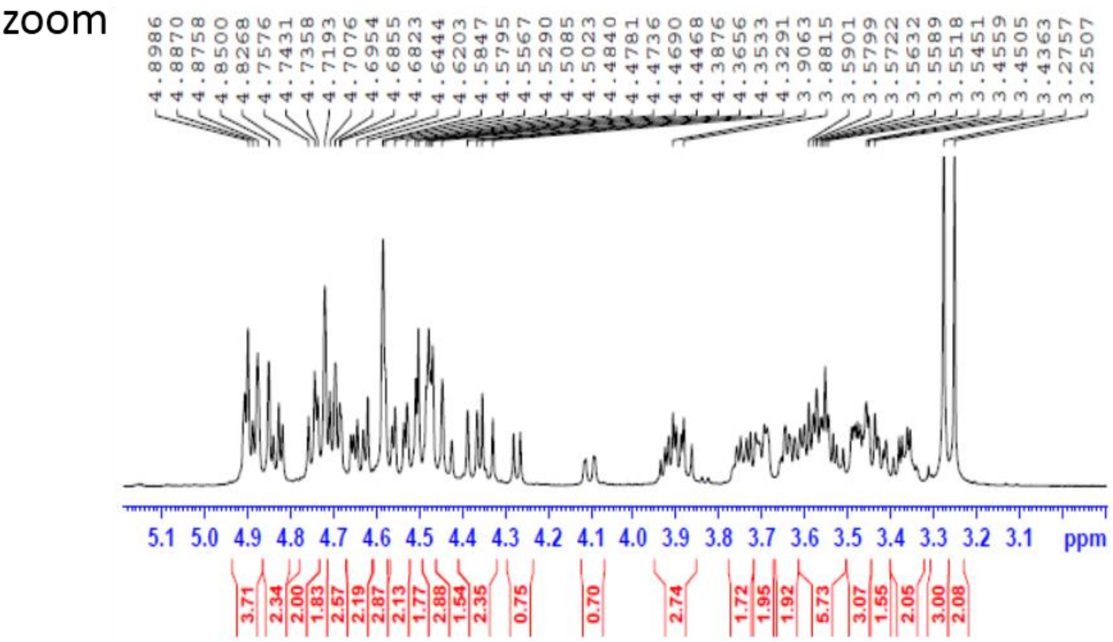

COSY NMR of 27αβ in $CDCl_3$

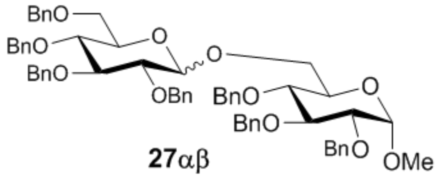

27αβ

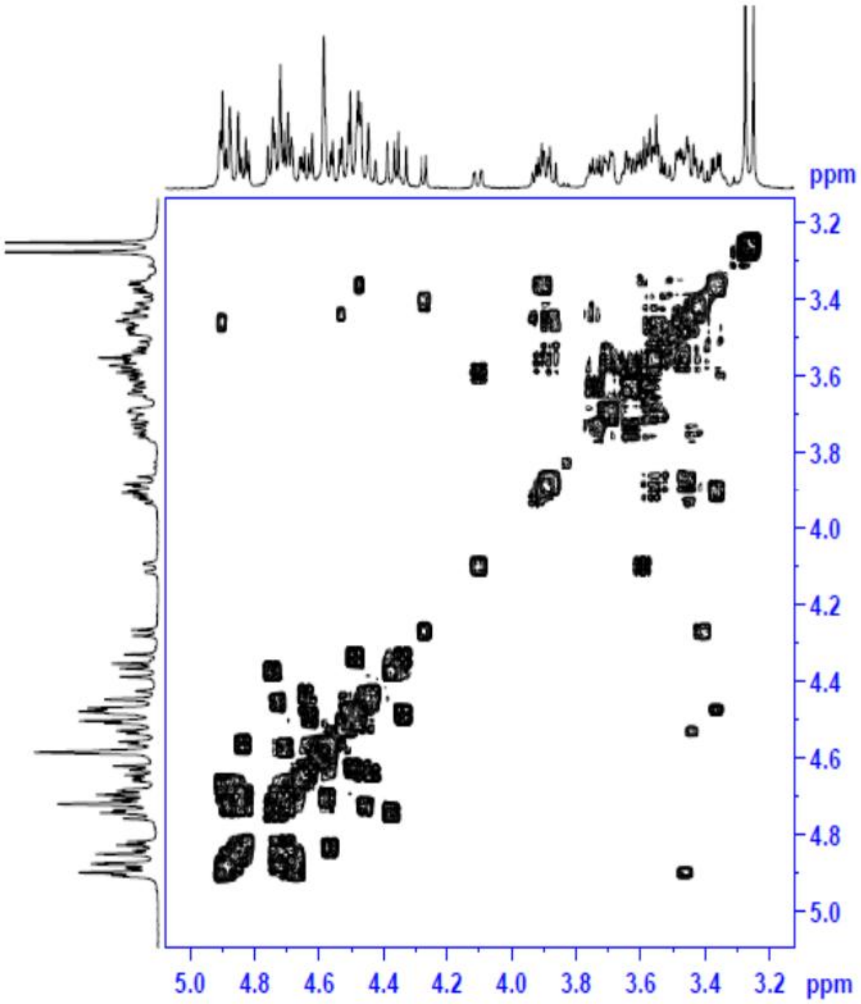

^{1}H NMR of 28α in $CDCl_3$

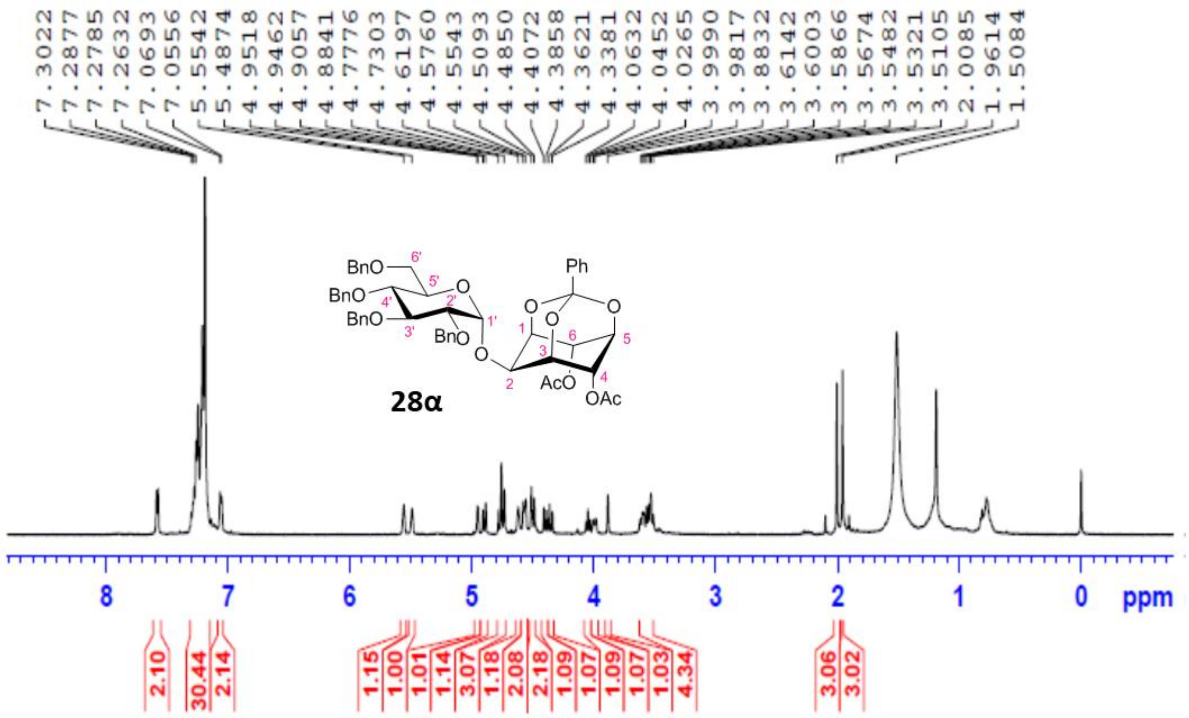

zoom

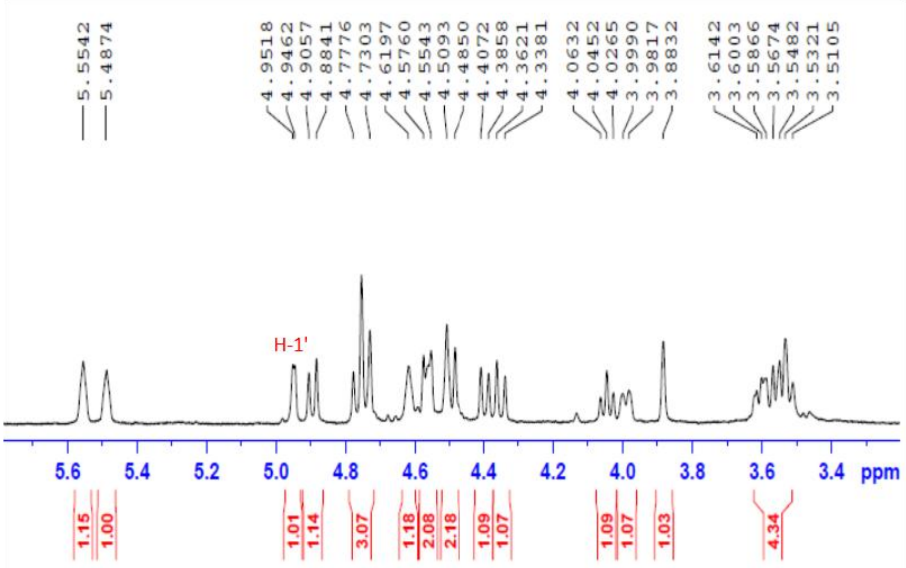

COSY NMR of 28α in $CDCl_3$

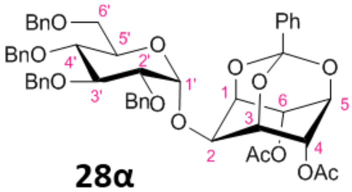

28α

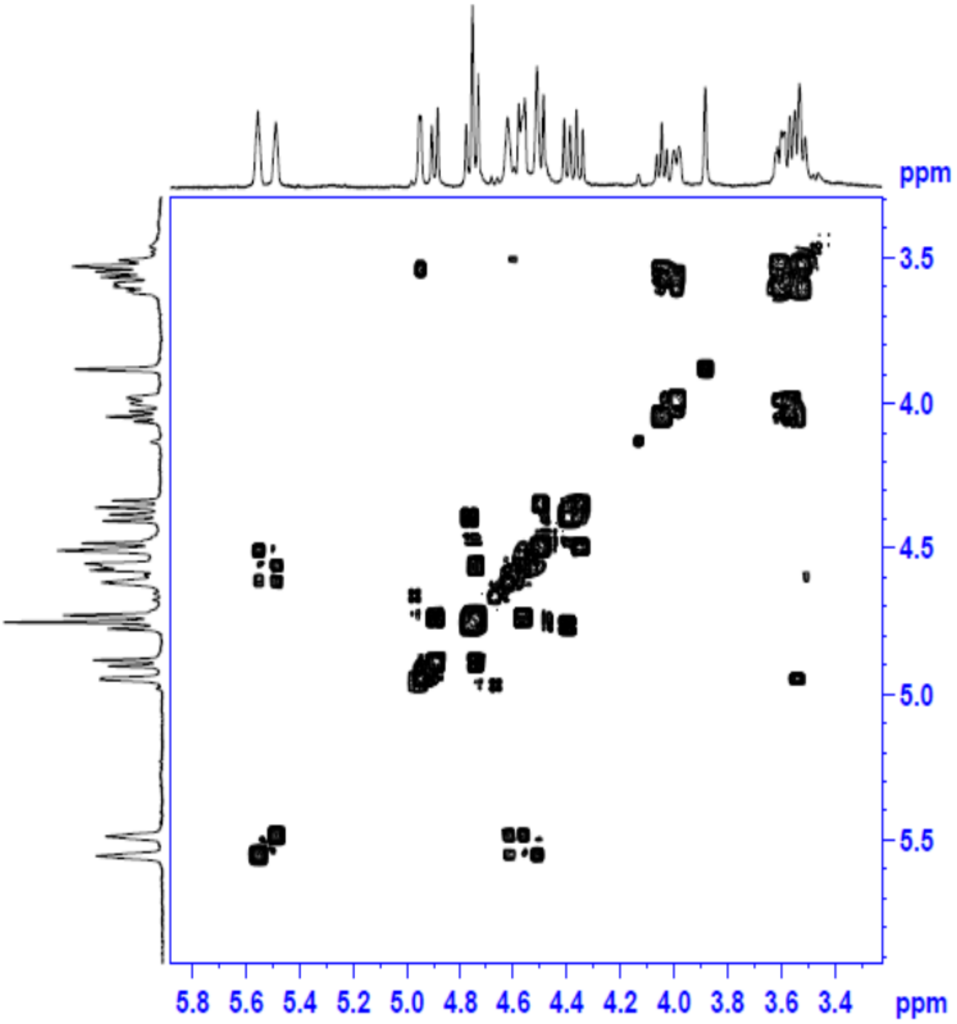

^{13}C NMR of 28α in CDCl$_3$

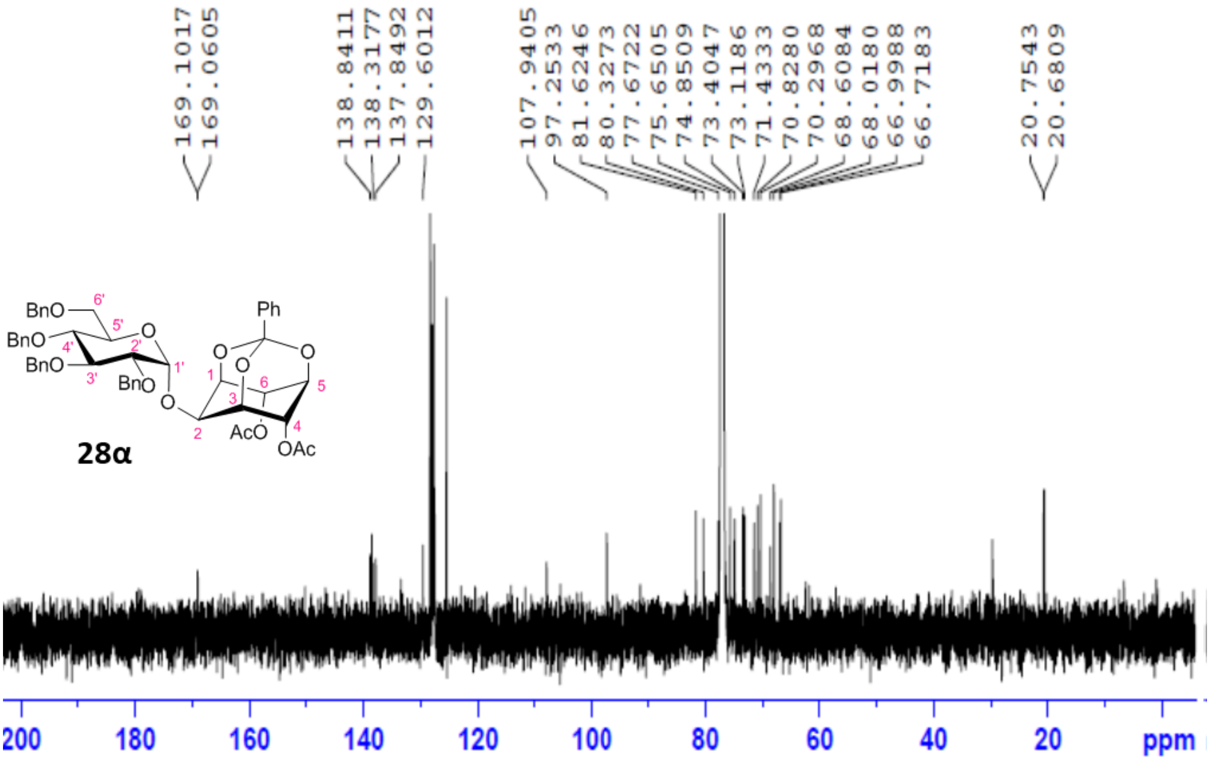

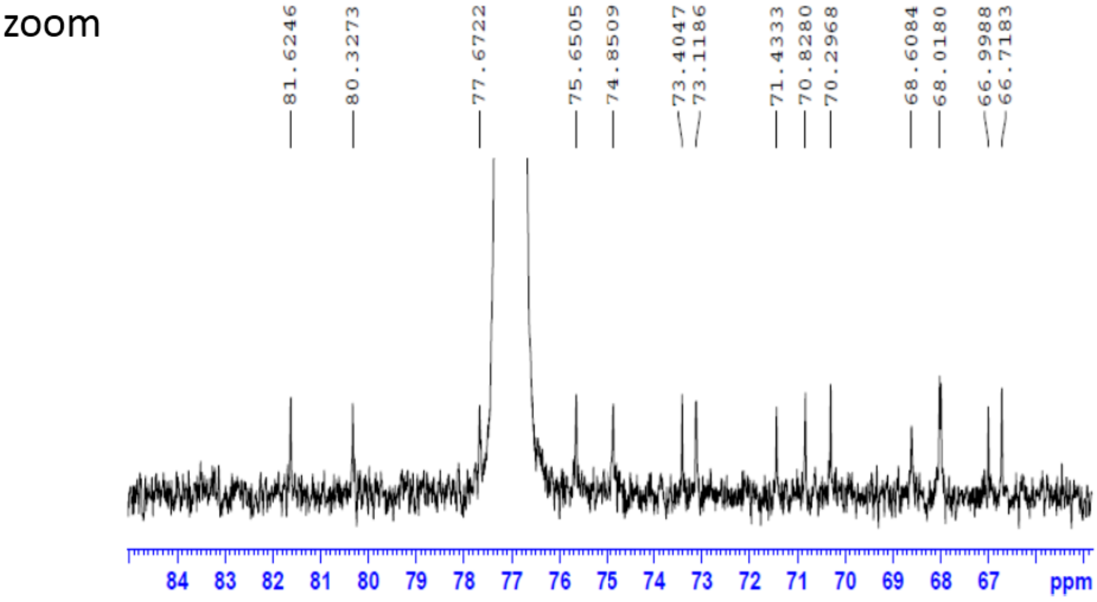

DEPT NMR of 28α in $CDCl_3$

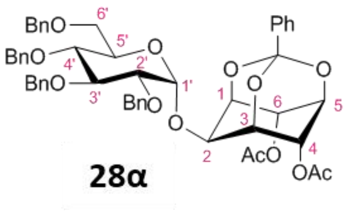

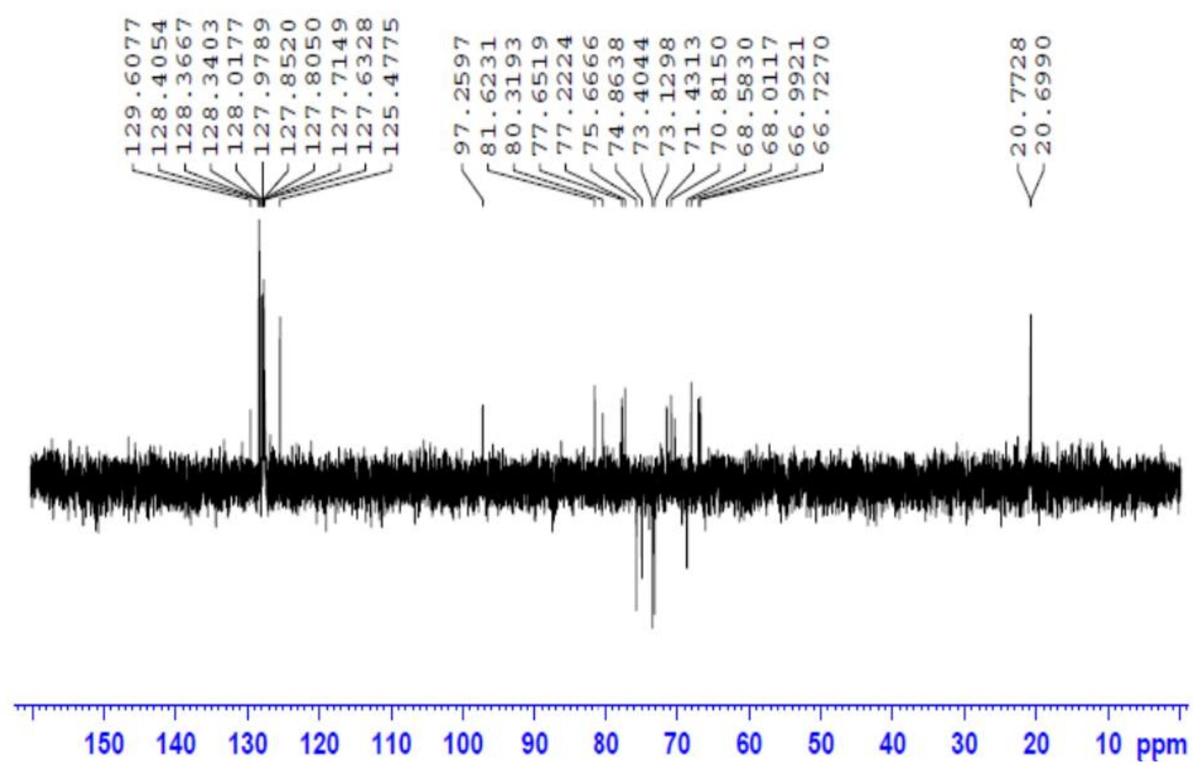

HMBC NMR of 28α in $CDCl_3$

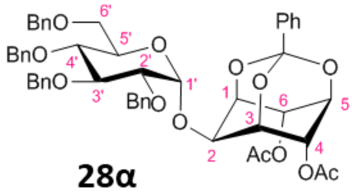

28α

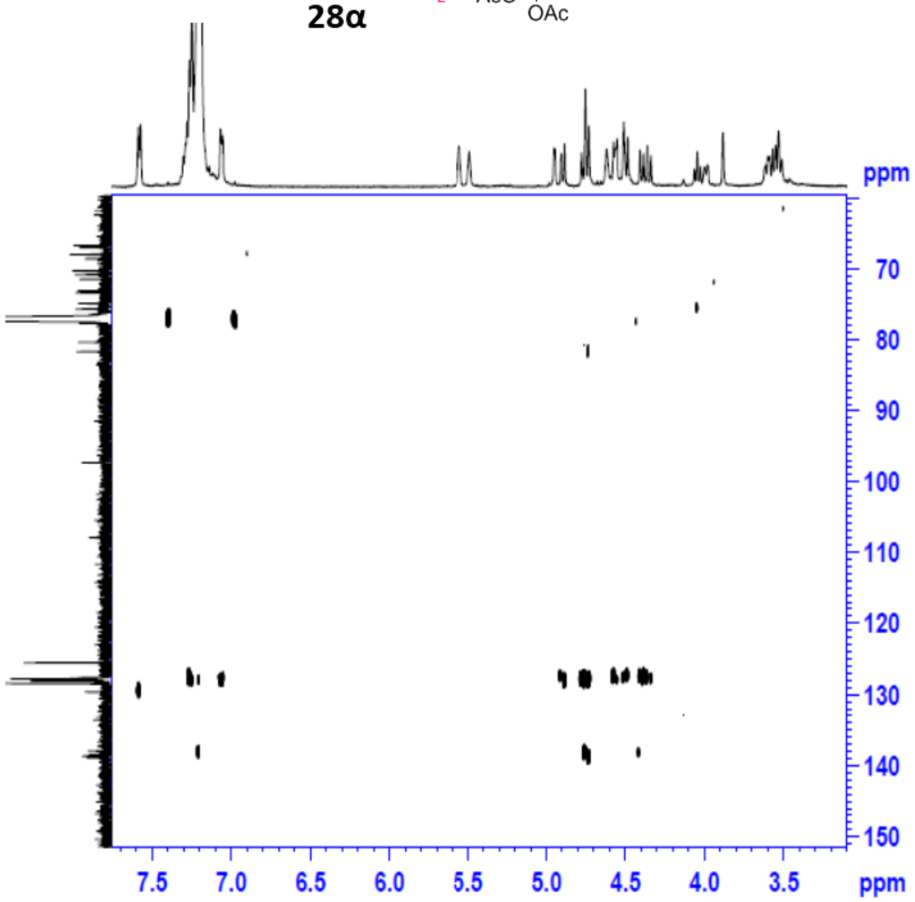

HMQC NMR of 28α in $CDCl_3$

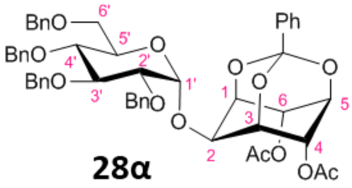

28α

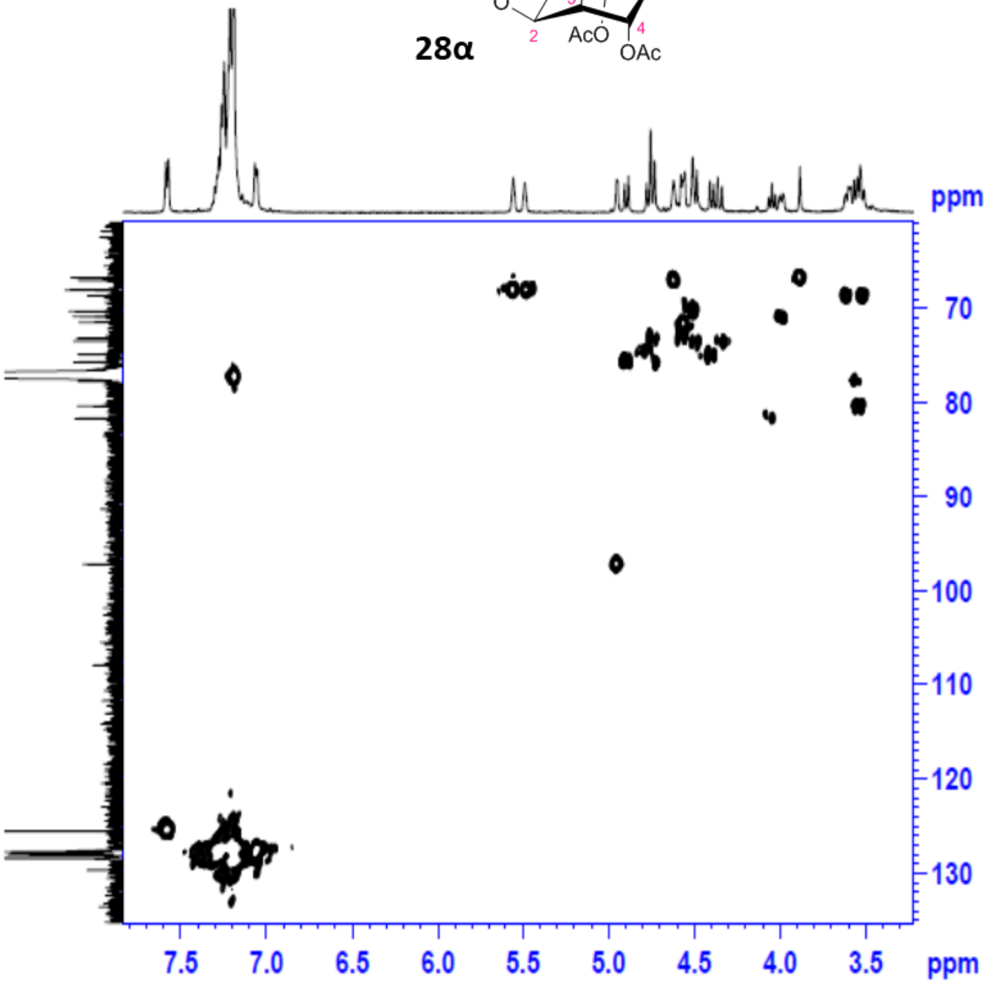

^{1}H NMR of 28β in $CDCl_3$

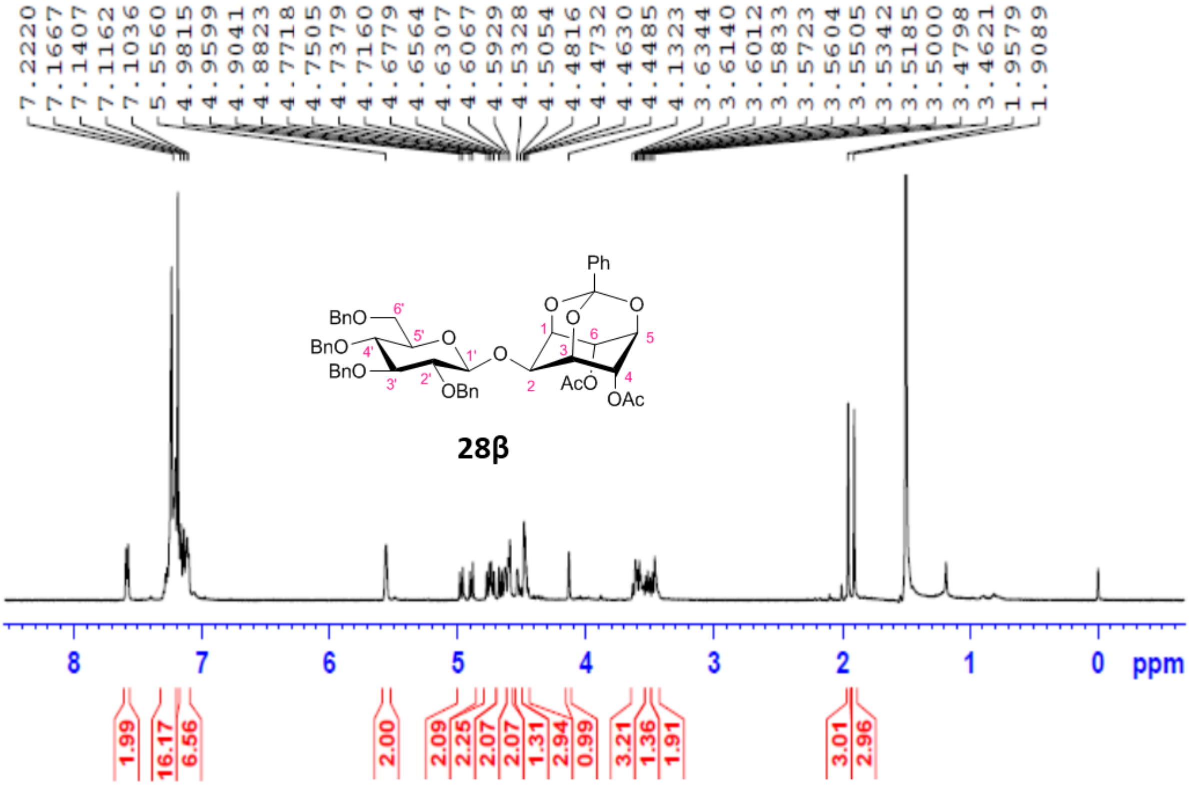

zoom

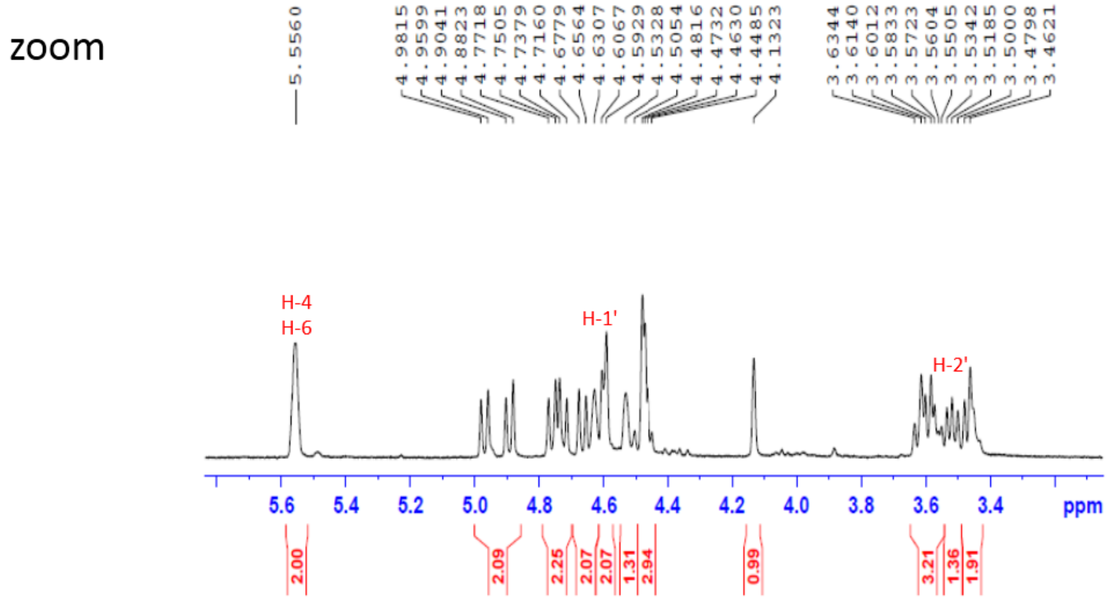

COSY NMR of 28β in $CDCl_3$

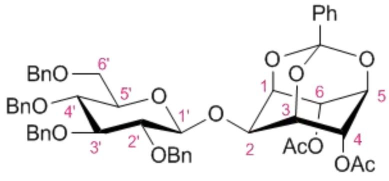

28β

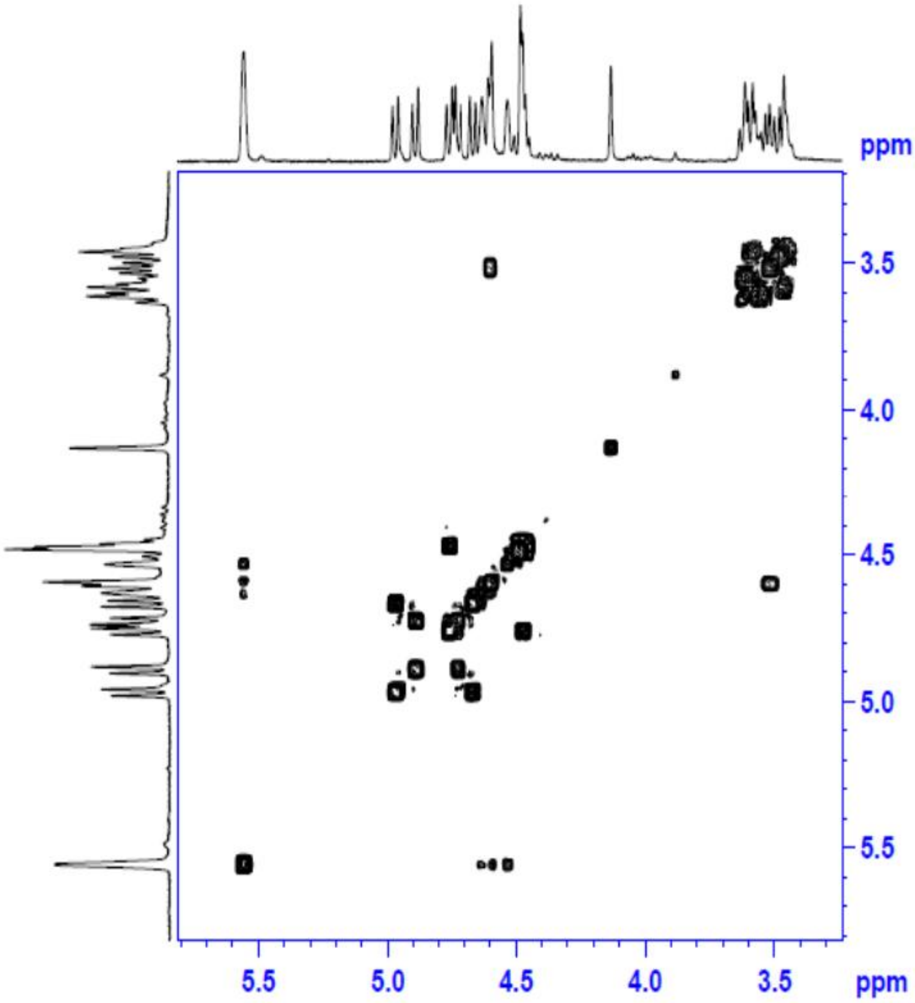

^{13}C NMR of 28β in CDCl$_3$

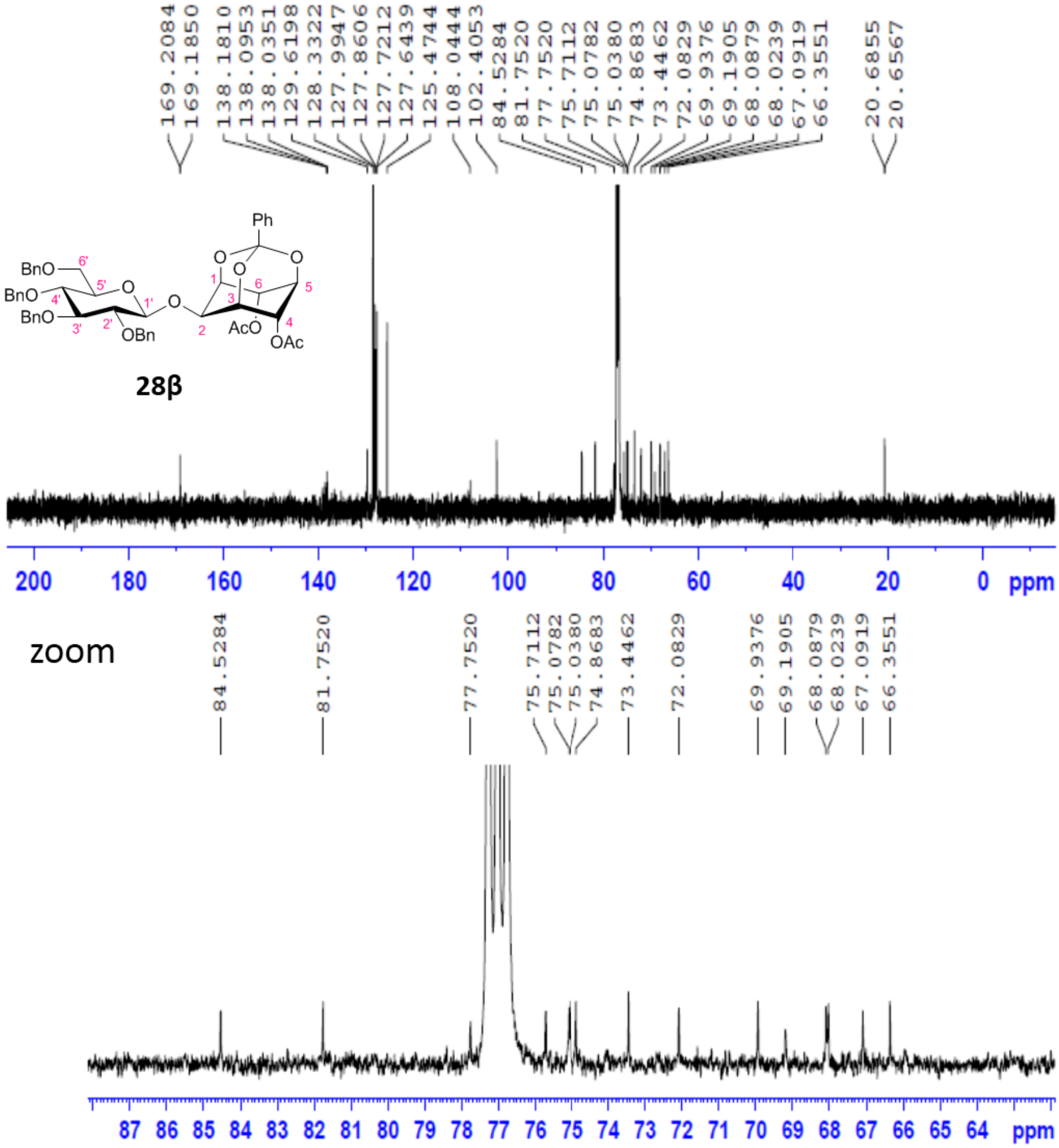

DEPT NMR of 28β in $CDCl_3$

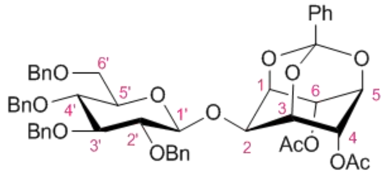

28β

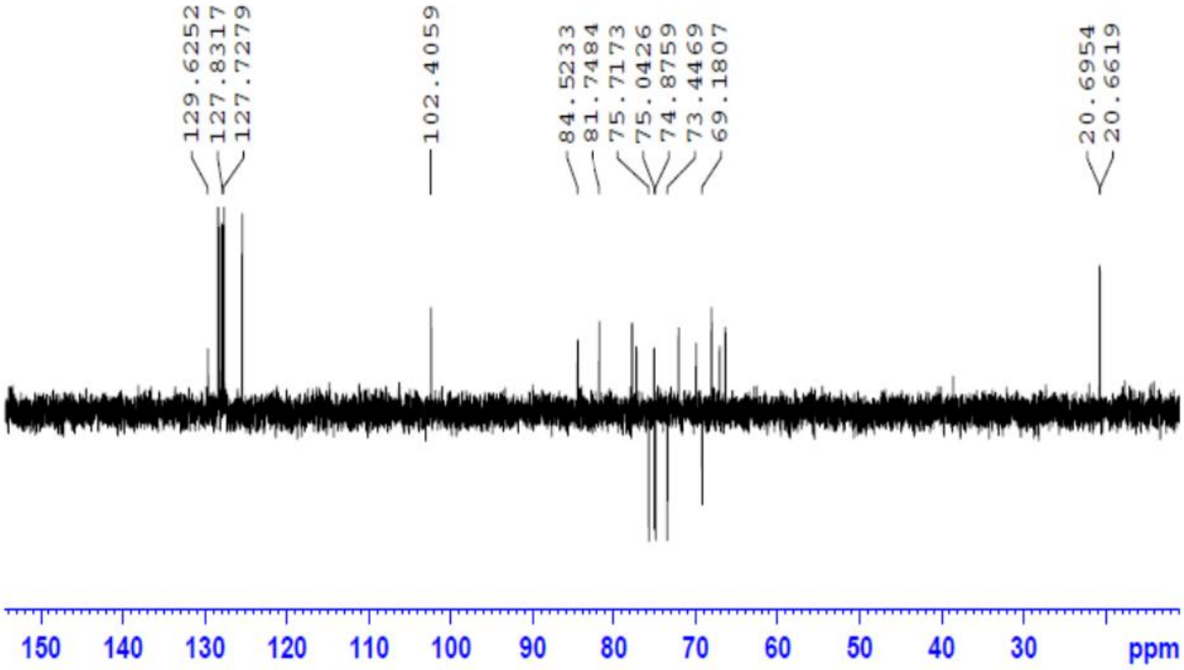

HMBC NMR of 28β in $CDCl_3$

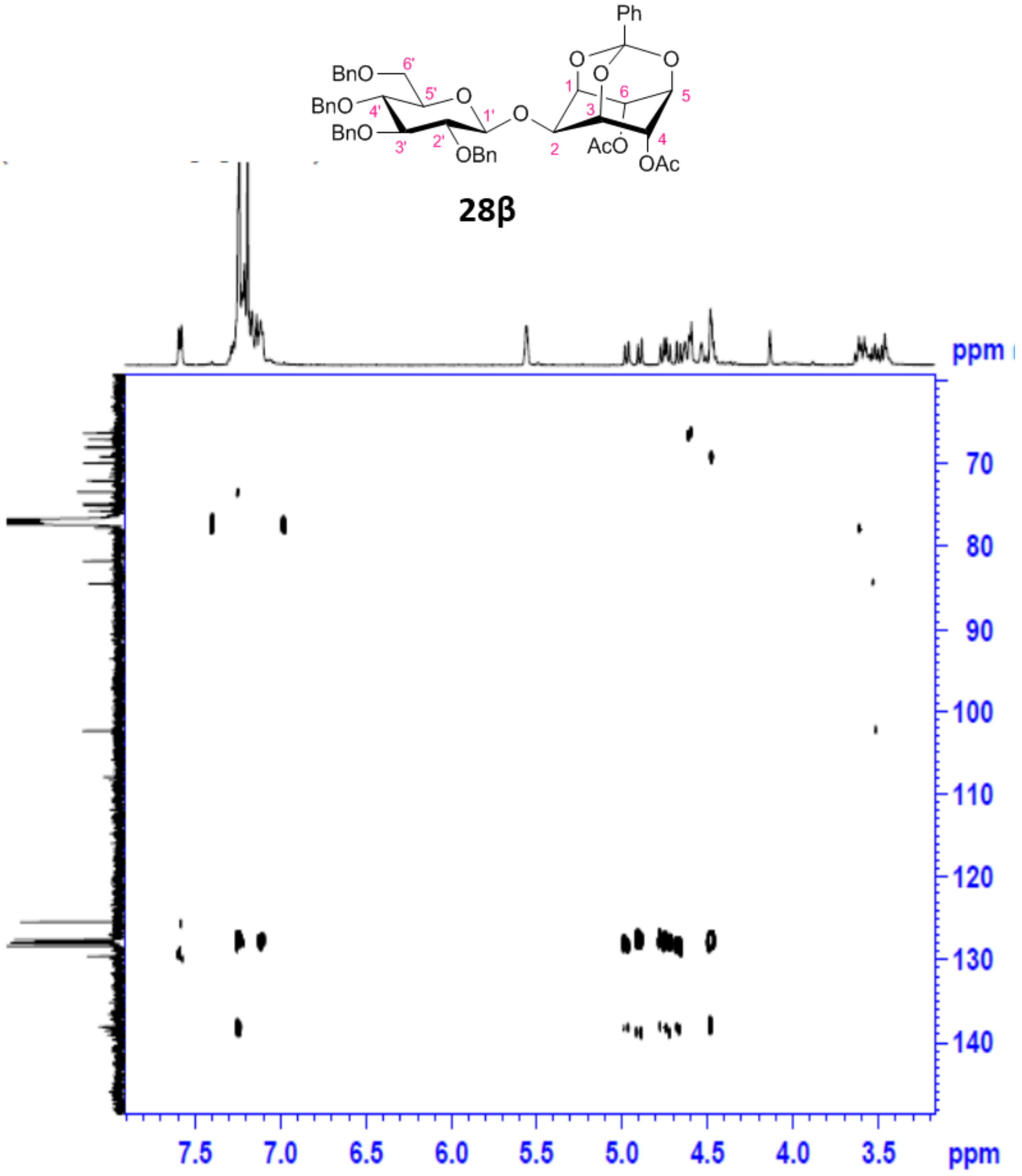

HMQC NMR of 28β in $CDCl_3$

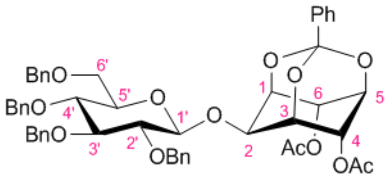

28β

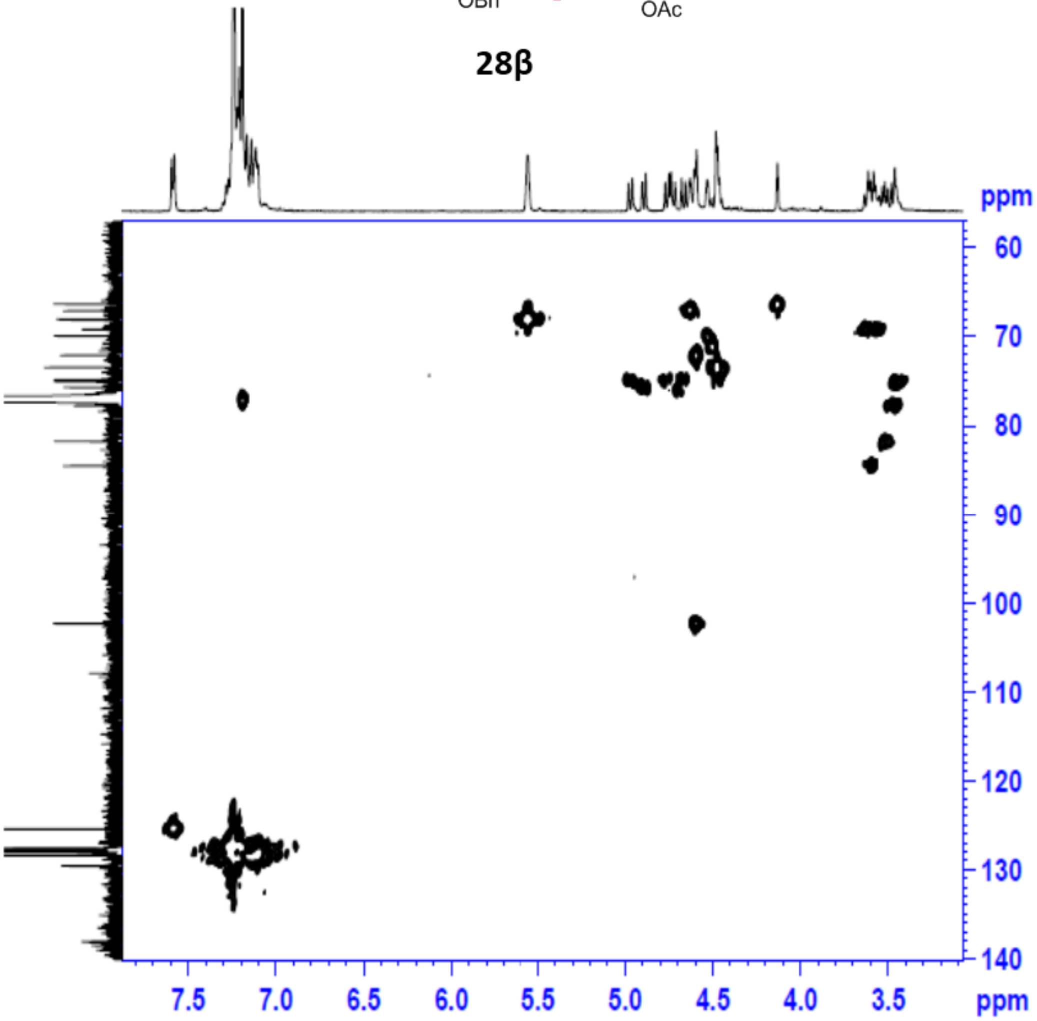

^{1}H NMR of D14 in $CDCl_3$

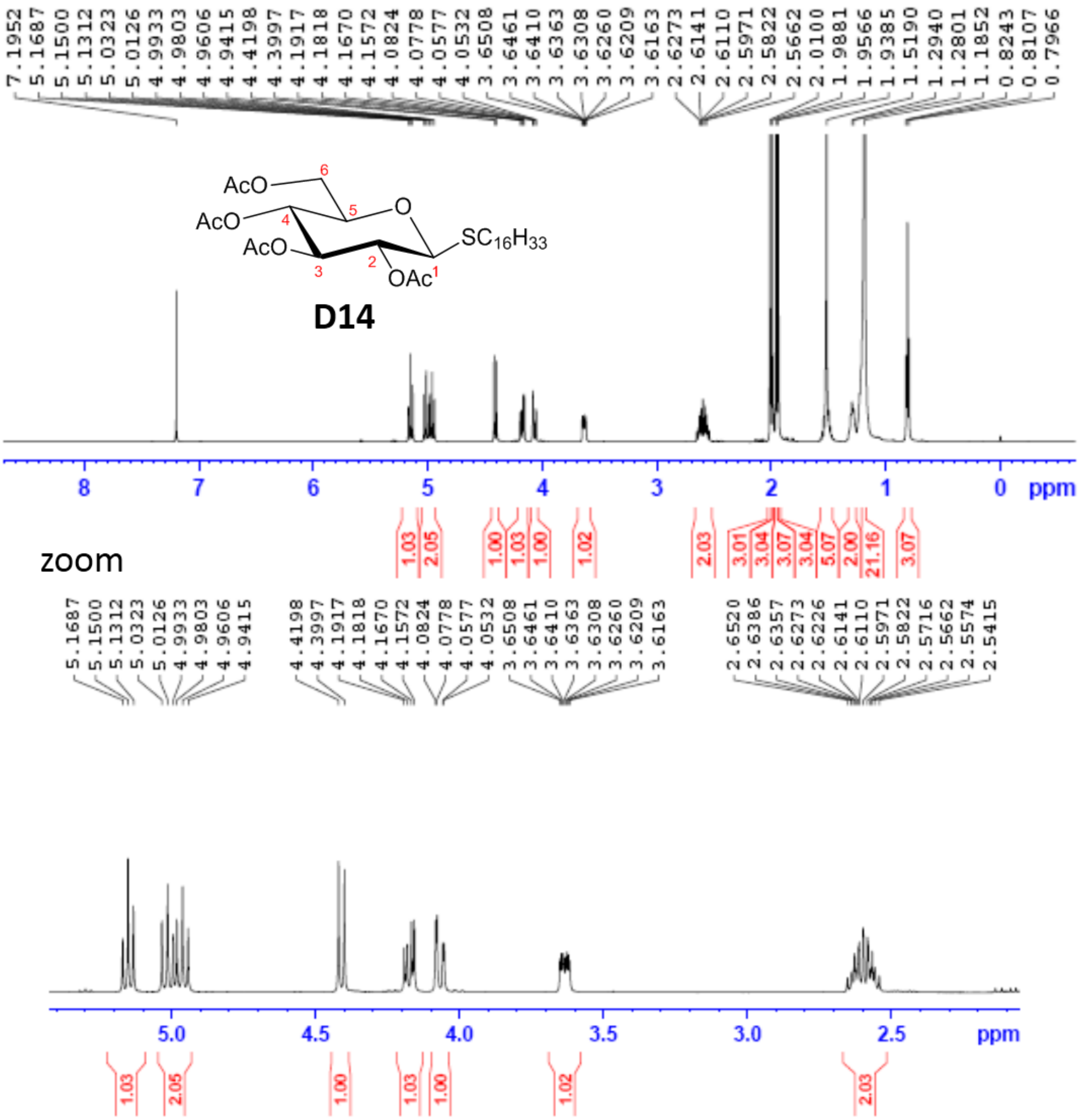

COSY NMR of D14 in $CDCl_3$

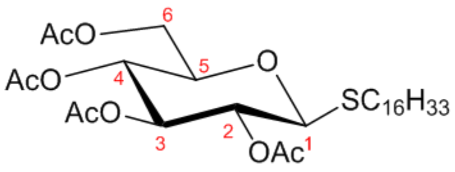

D14

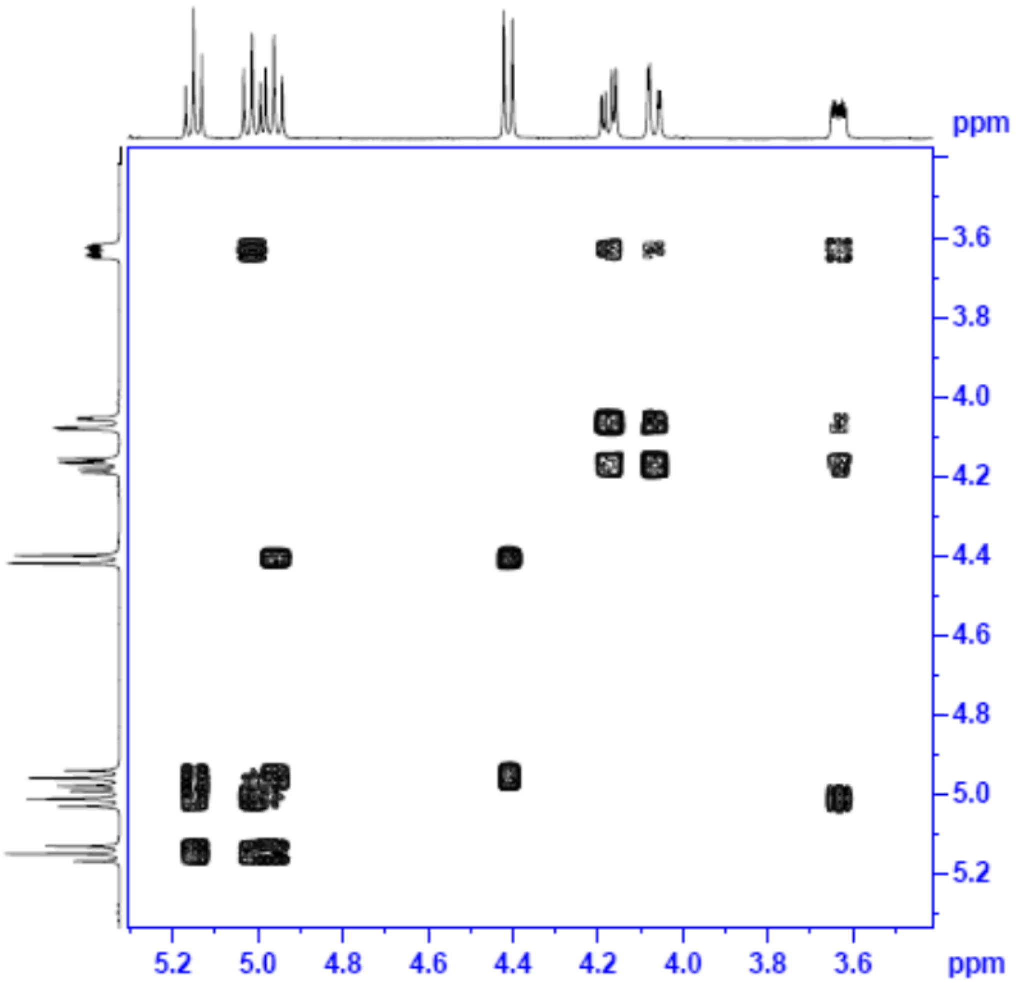

^{13}C NMR of D14 in $CDCl_3$

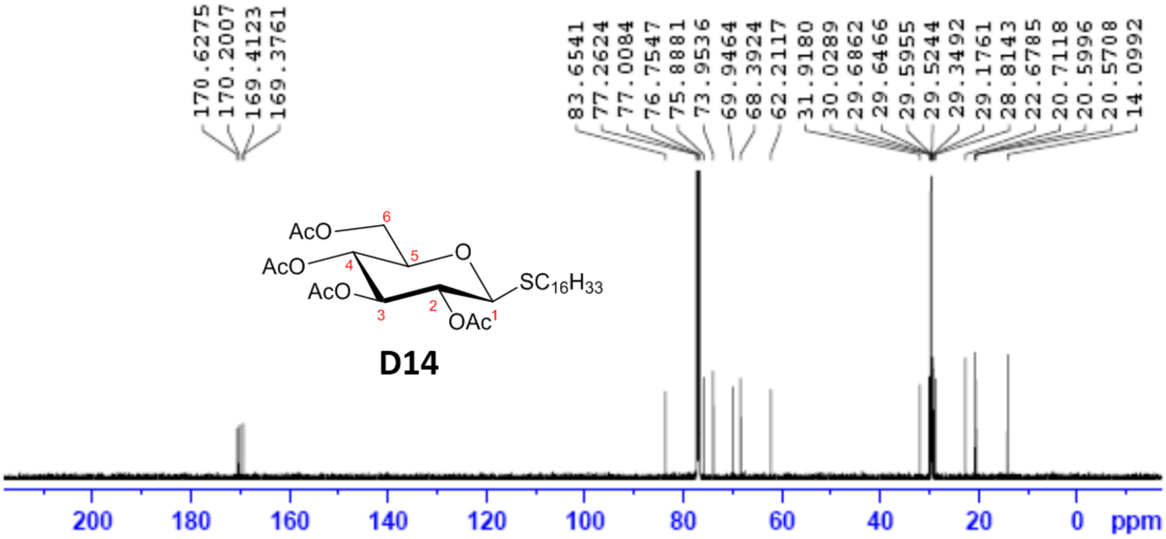

zoom

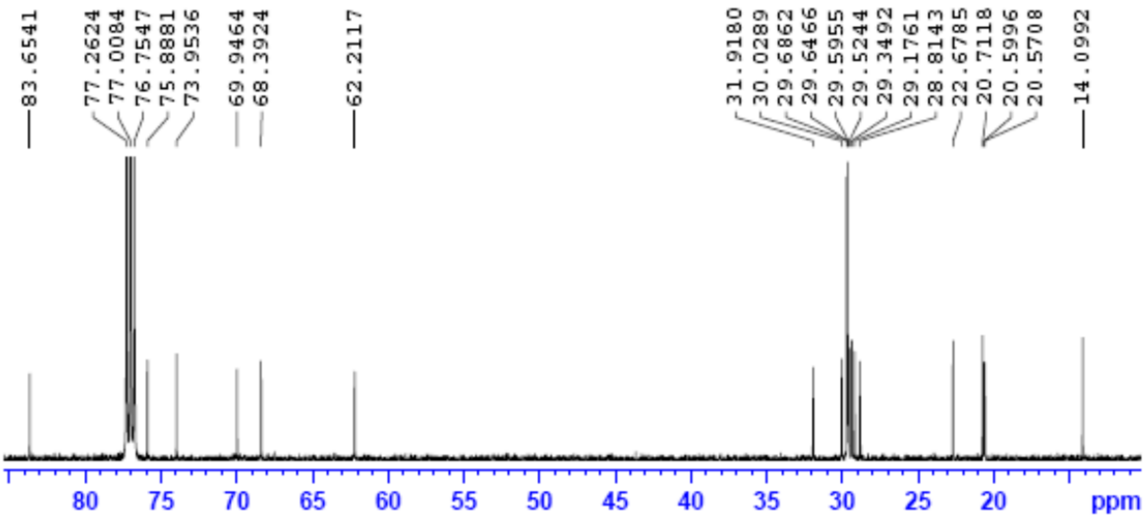

DEPT NMR of D14 in $CDCl_3$

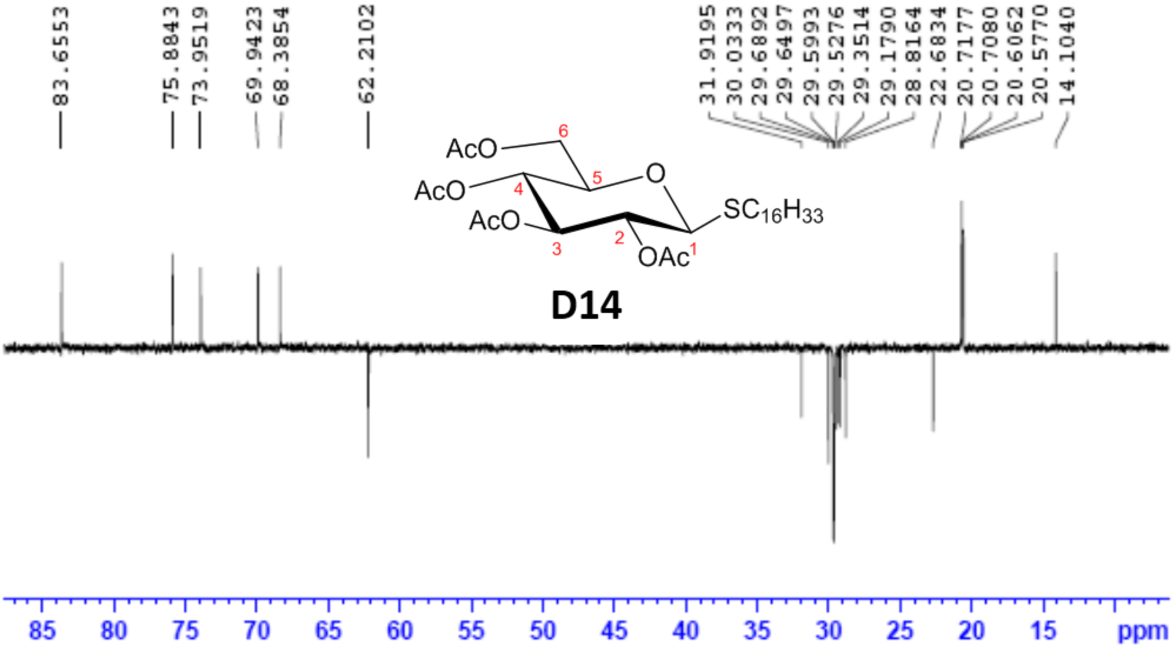

HMBC

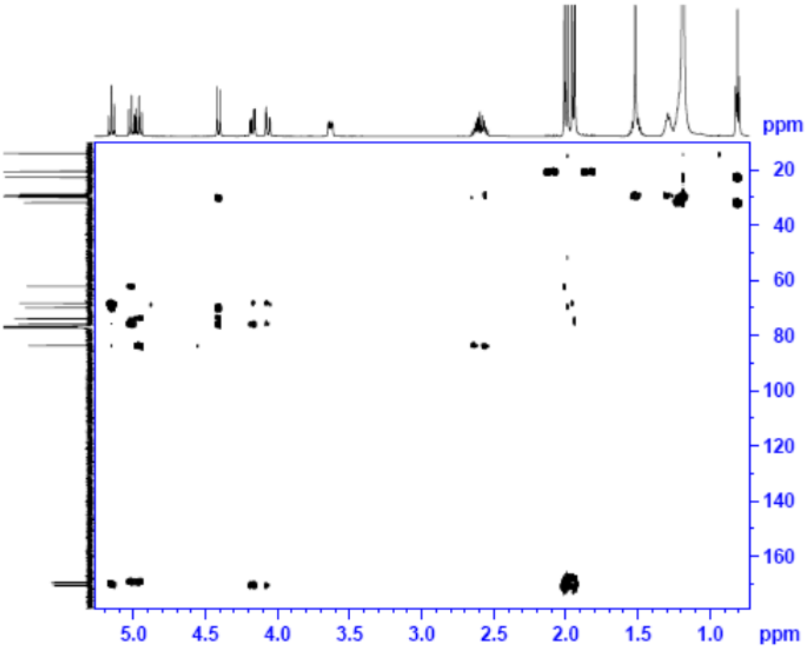

HMQC NMR of D14 in $CDCl_3$

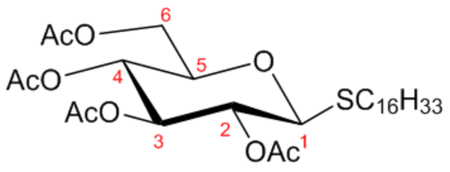

D14

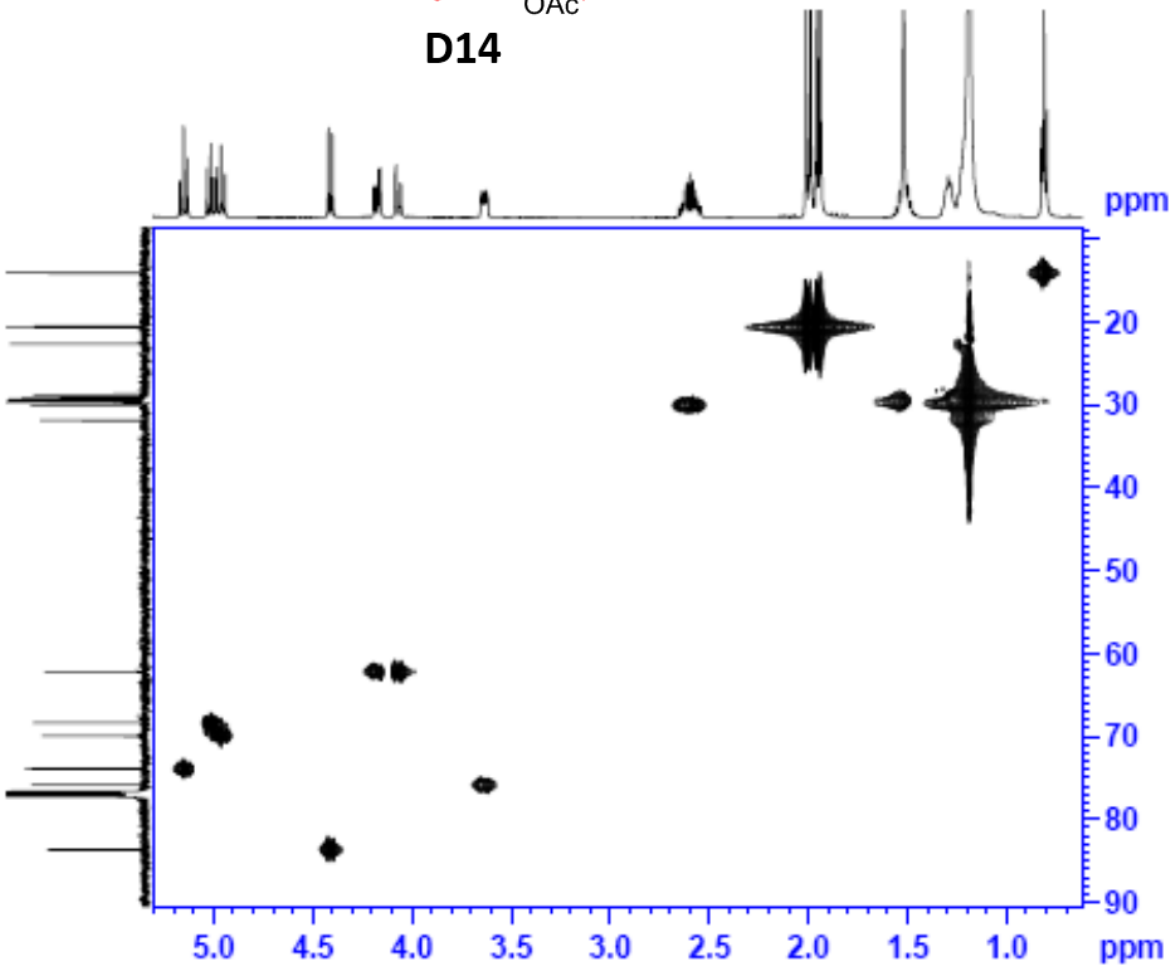

^{1}H NMR of D15 in $CDCl_3$

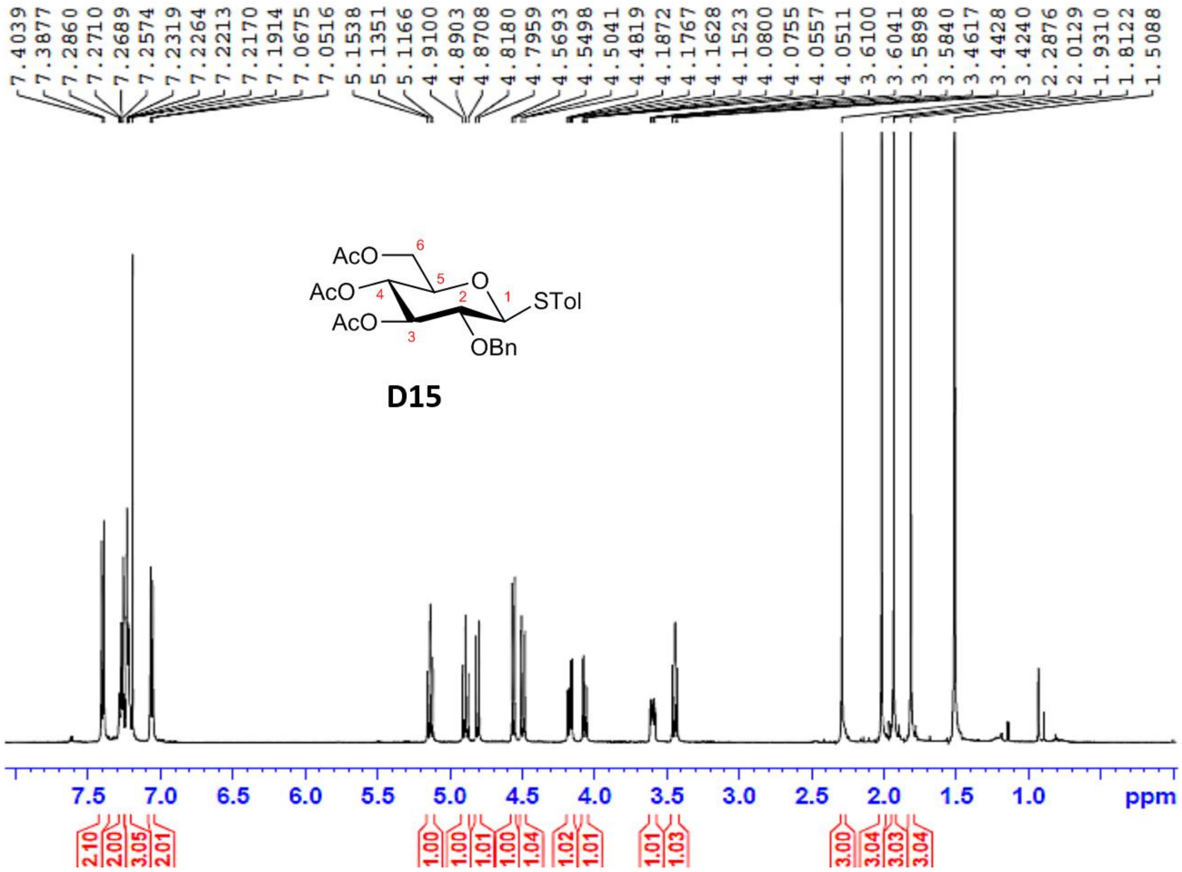

^{1}H NMR of D15 in $CDCl_3$ (ZOOM)

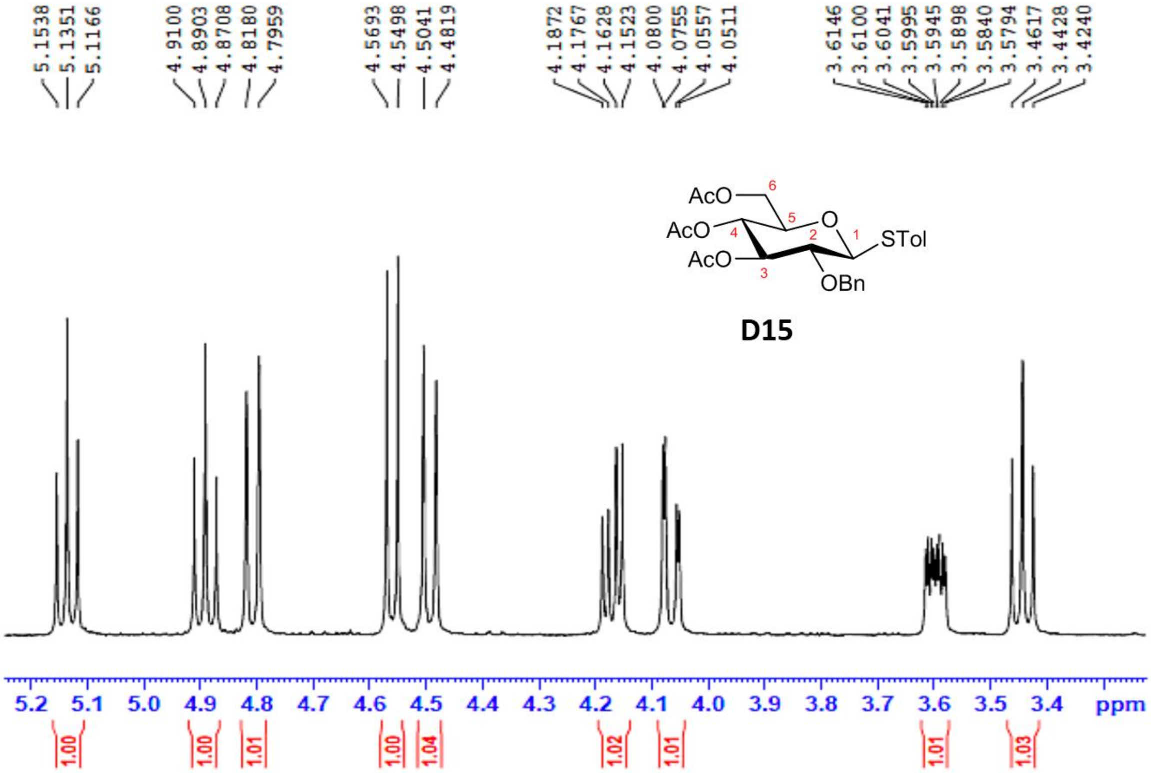

^{13}C NMR of D15 in $CDCl_3$

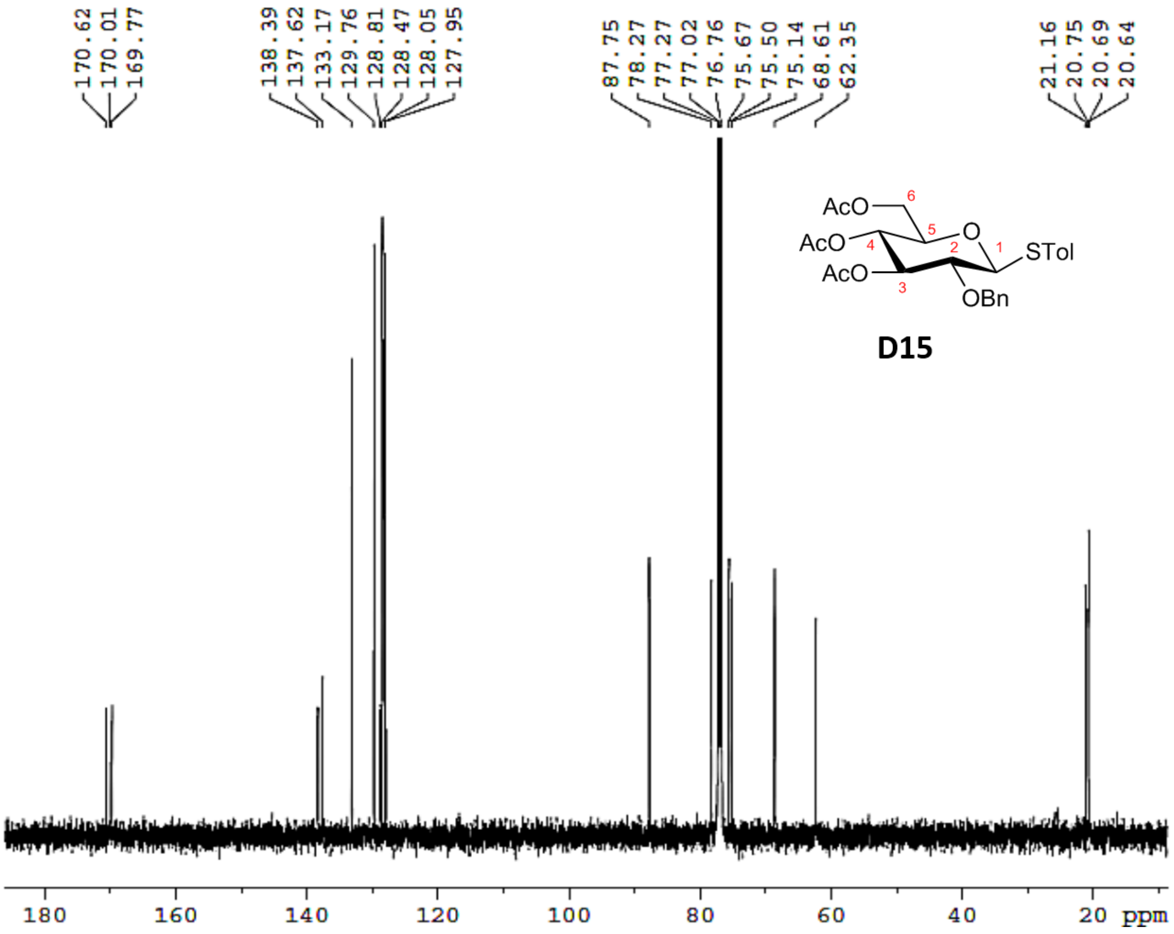

^{1}H NMR of 42 in CDCl$_3$

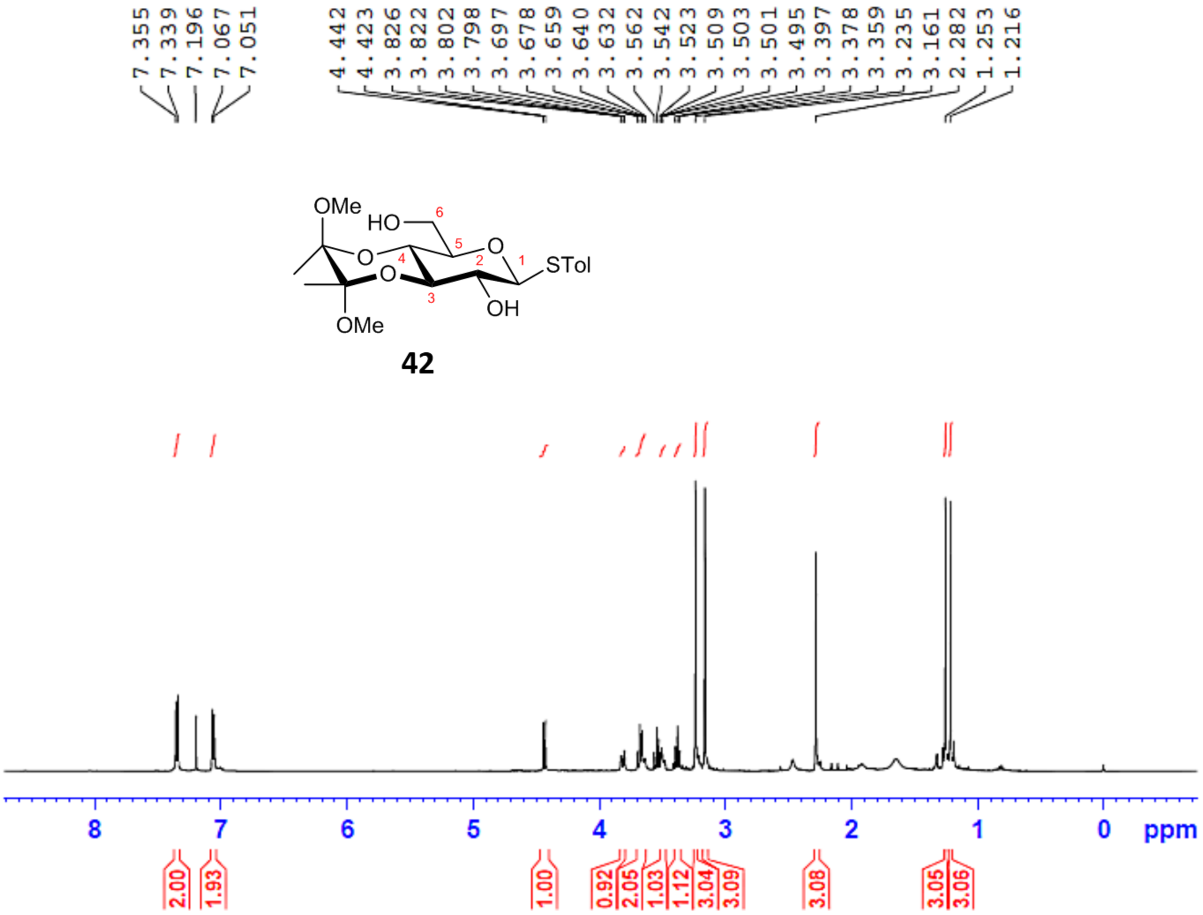

42

^{1}H NMR of 42 in $CDCl_3$ (ZOOM)

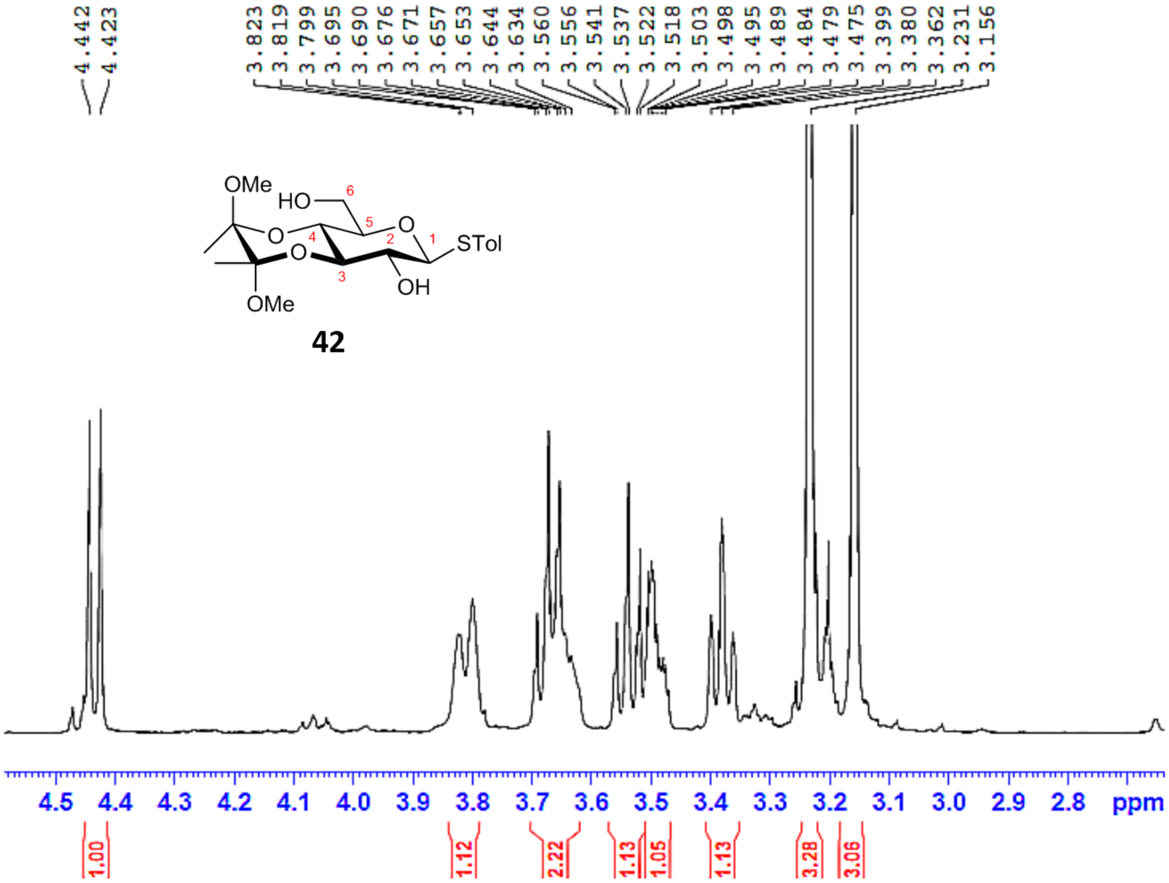

^{13}C NMR of 42 in CDCl$_3$

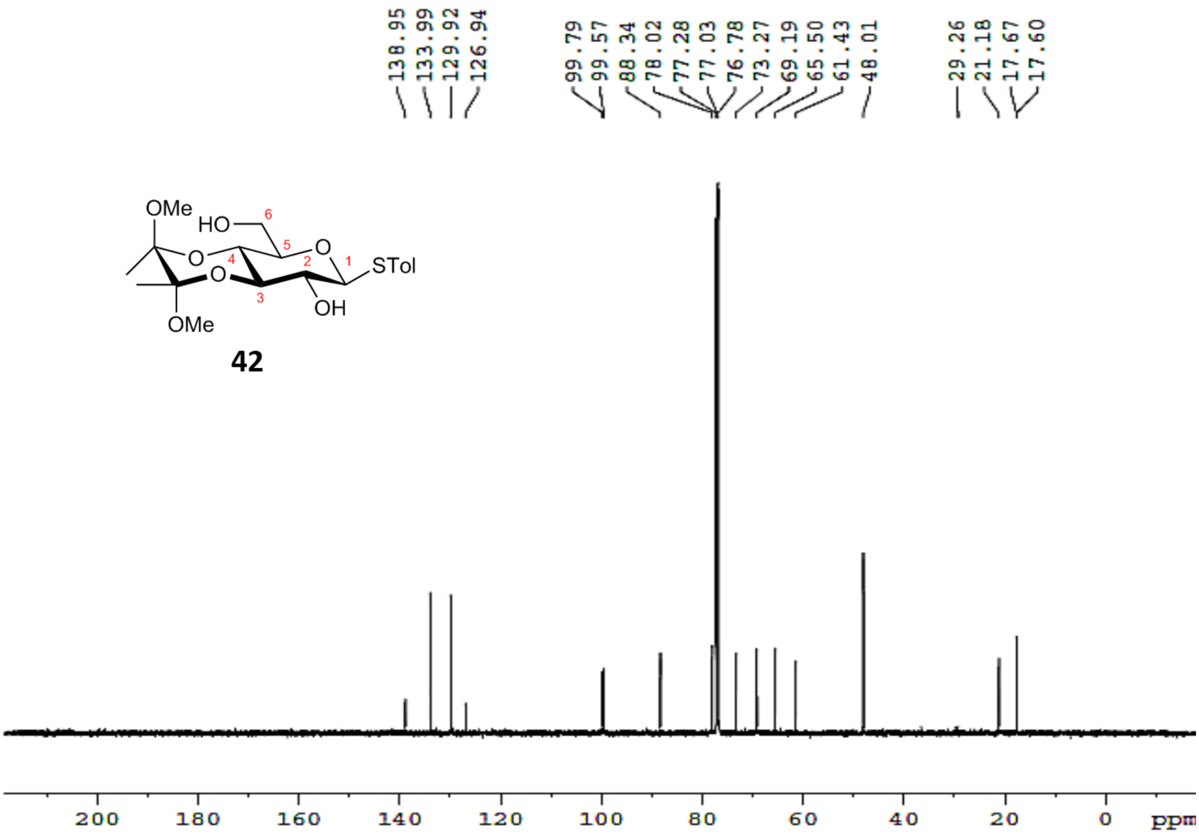

^{1}H NMR of 43 in CDCl$_3$

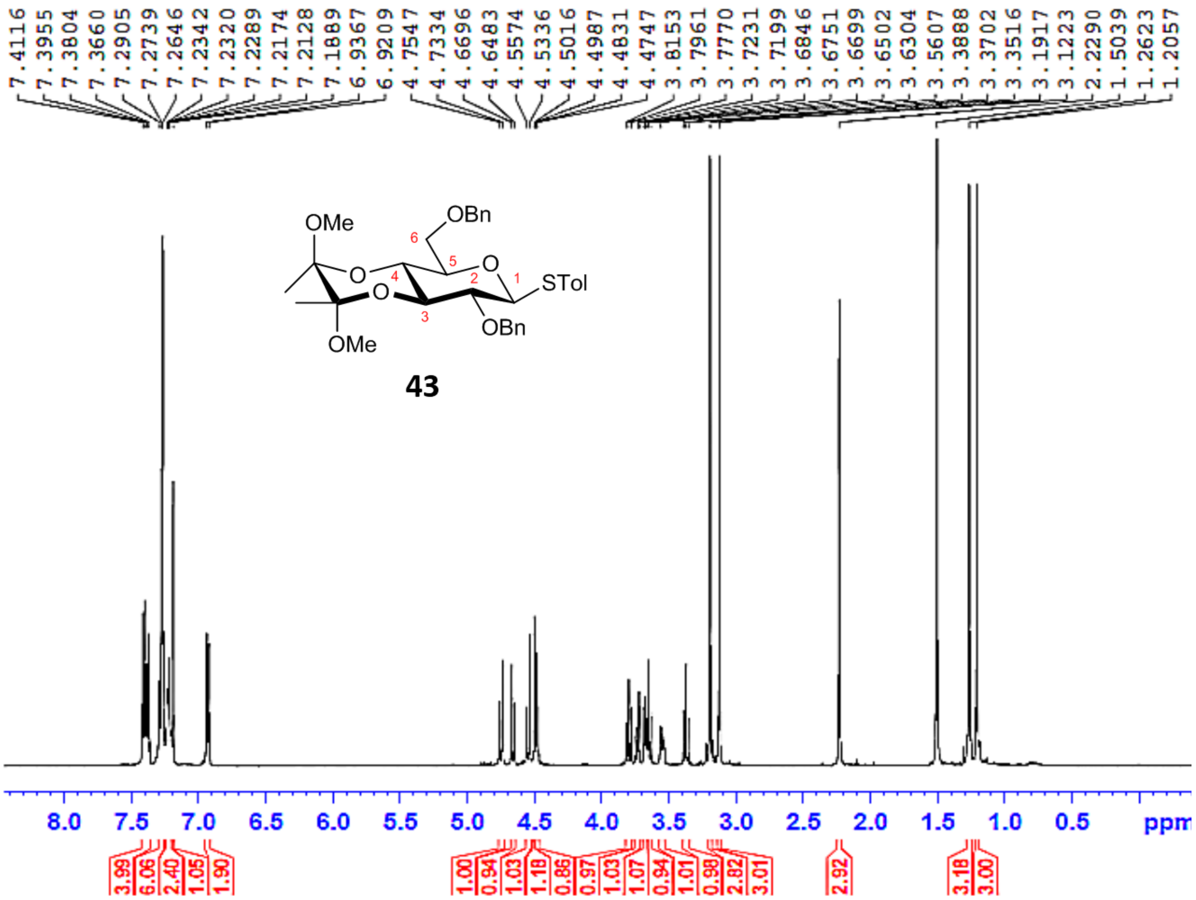

^{1}H NMR of 43 in CDCl$_3$ (ZOOM)

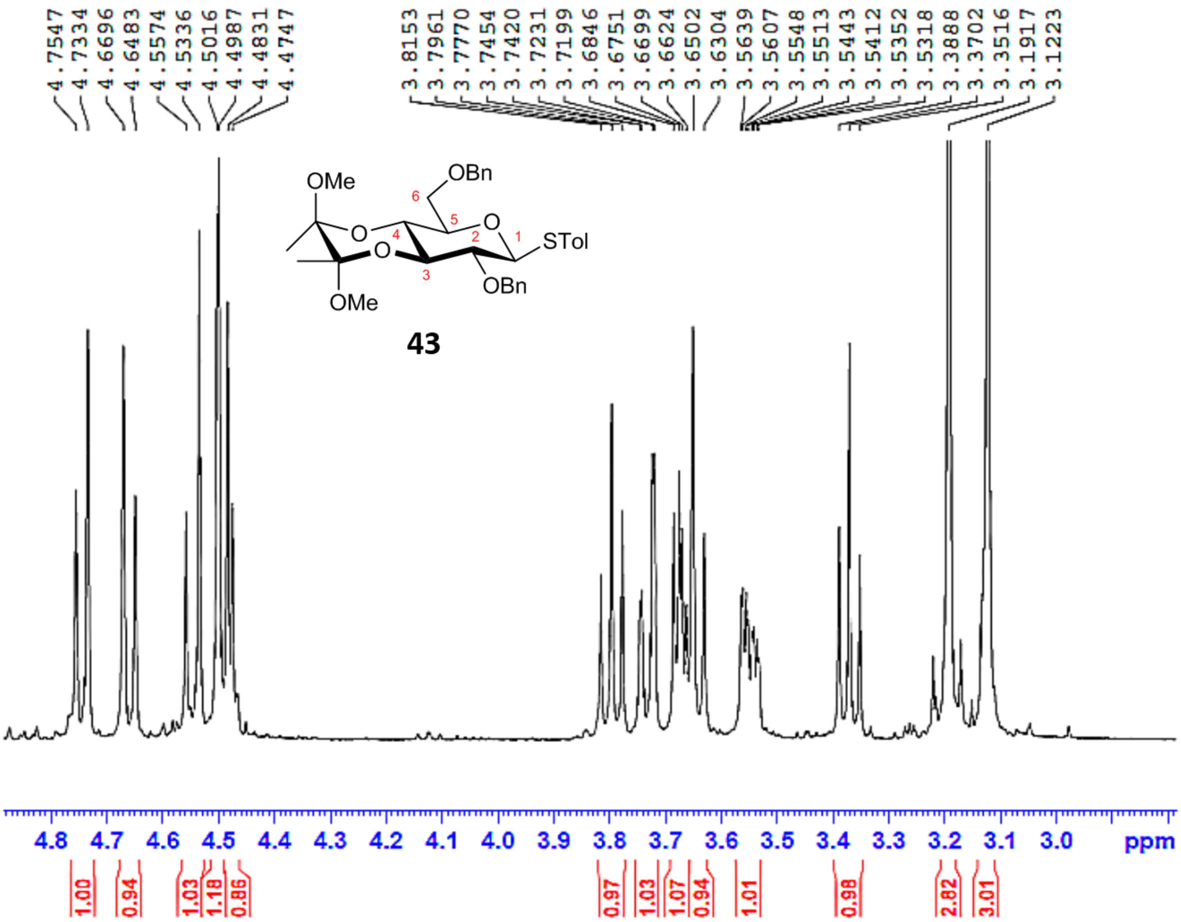

^{13}C NMR of 43 in $CDCl_3$

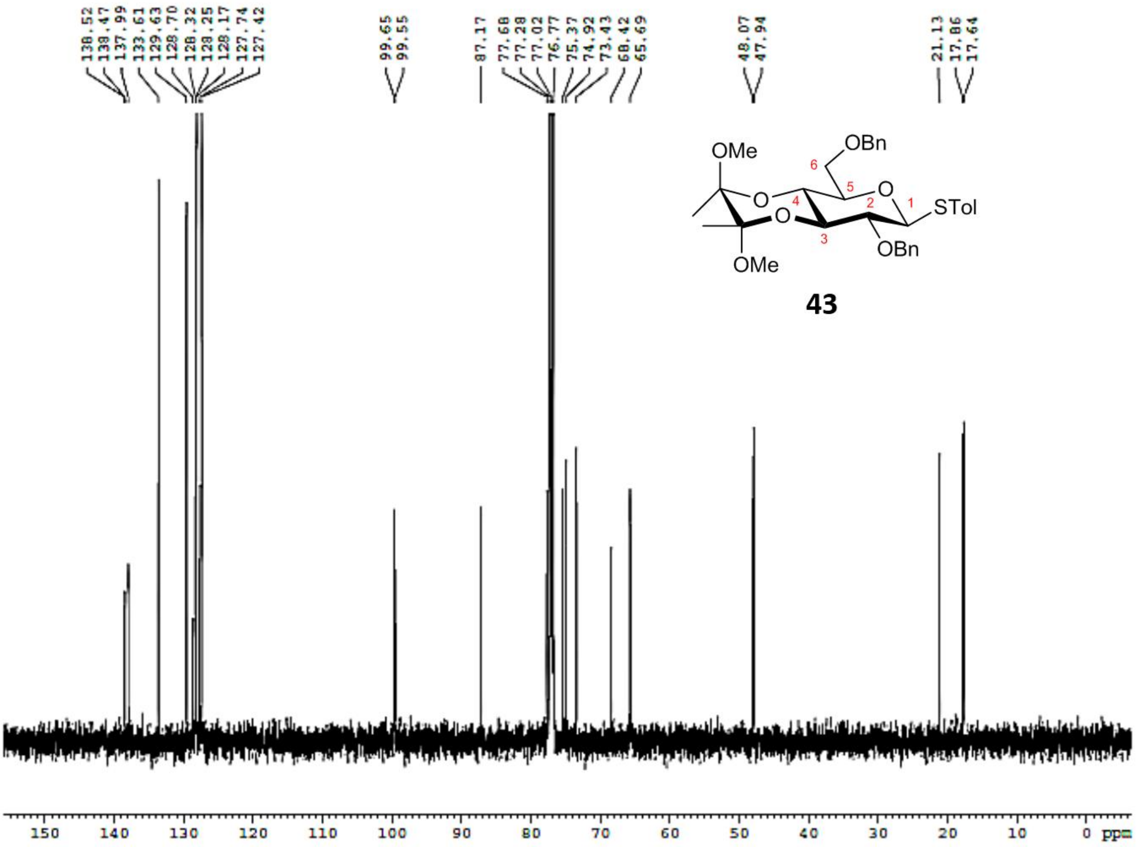

^{1}H NMR of D16 in $CDCl_3$

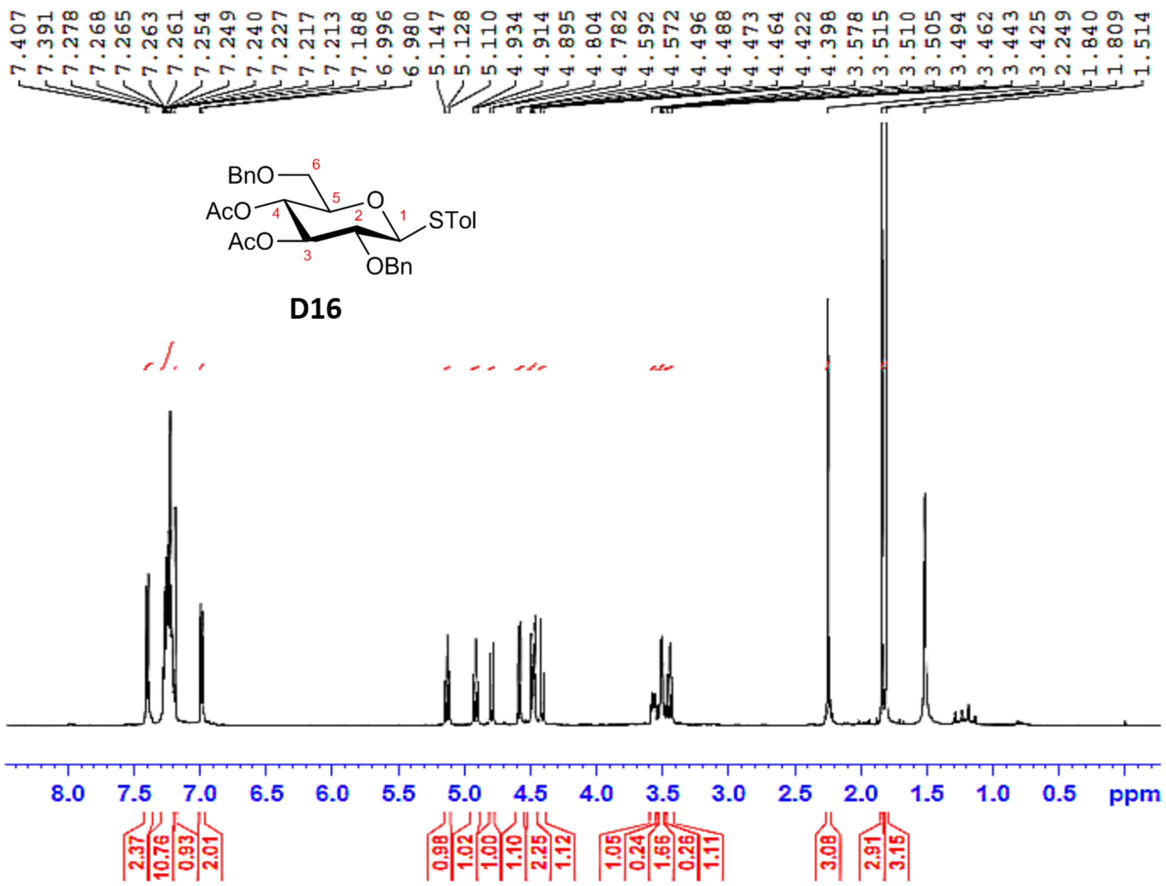

^{1}H NMR of D16 in $CDCl_3$ (ZOOM)

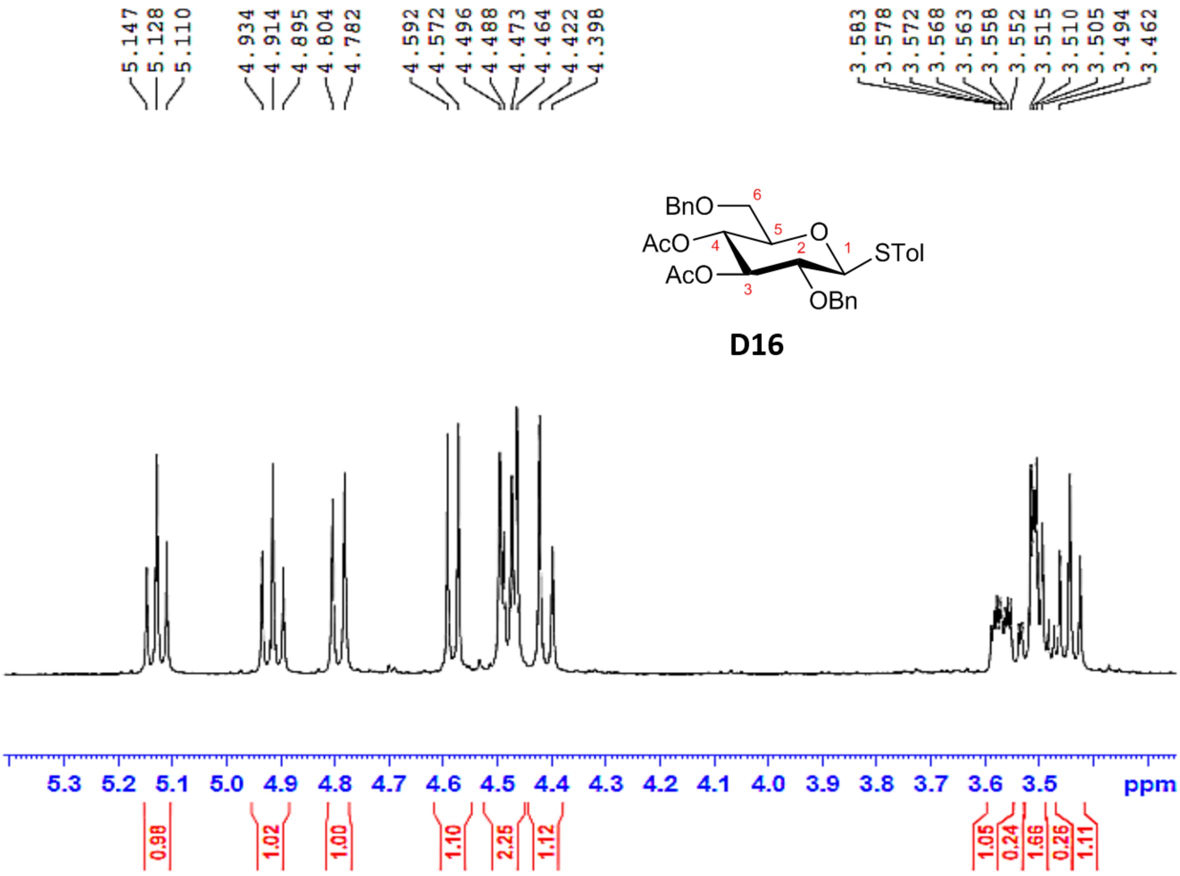

^{13}C NMR of D16 in $CDCl_3$

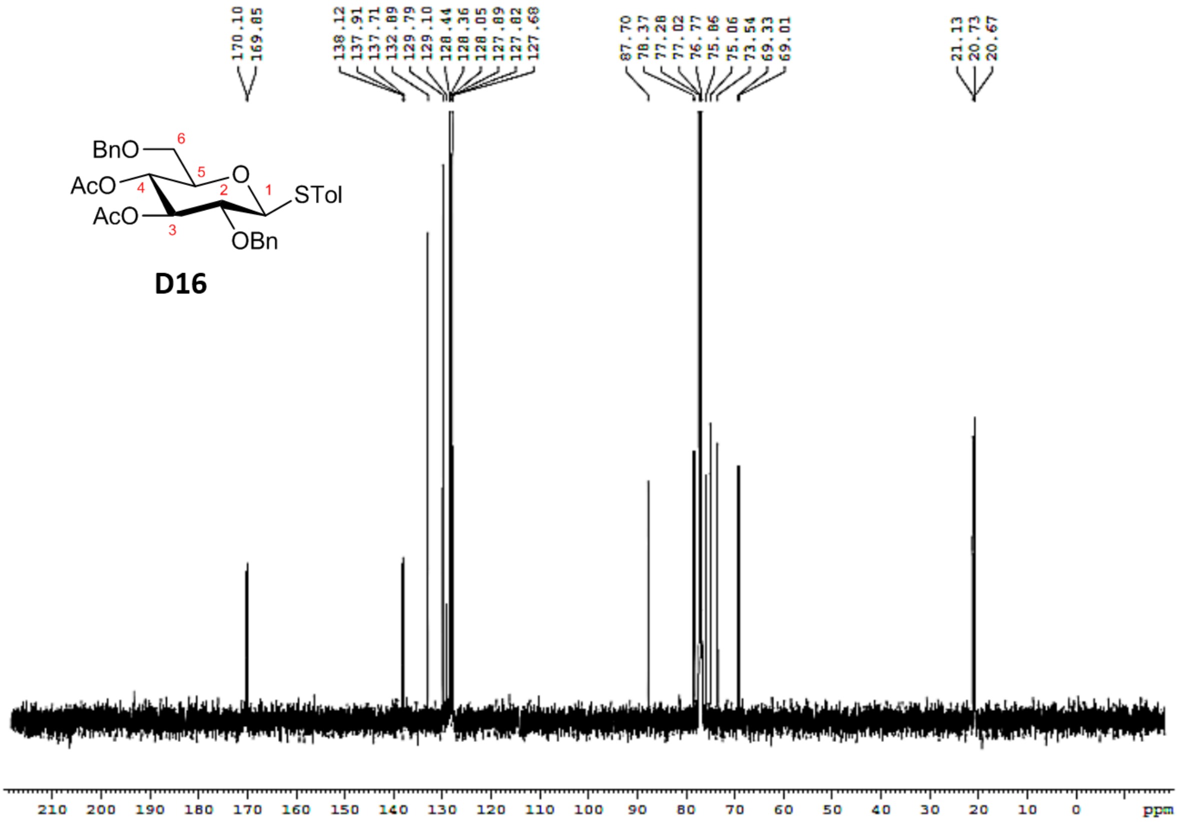

^1H NMR of 29αβ in $CDCl_3$

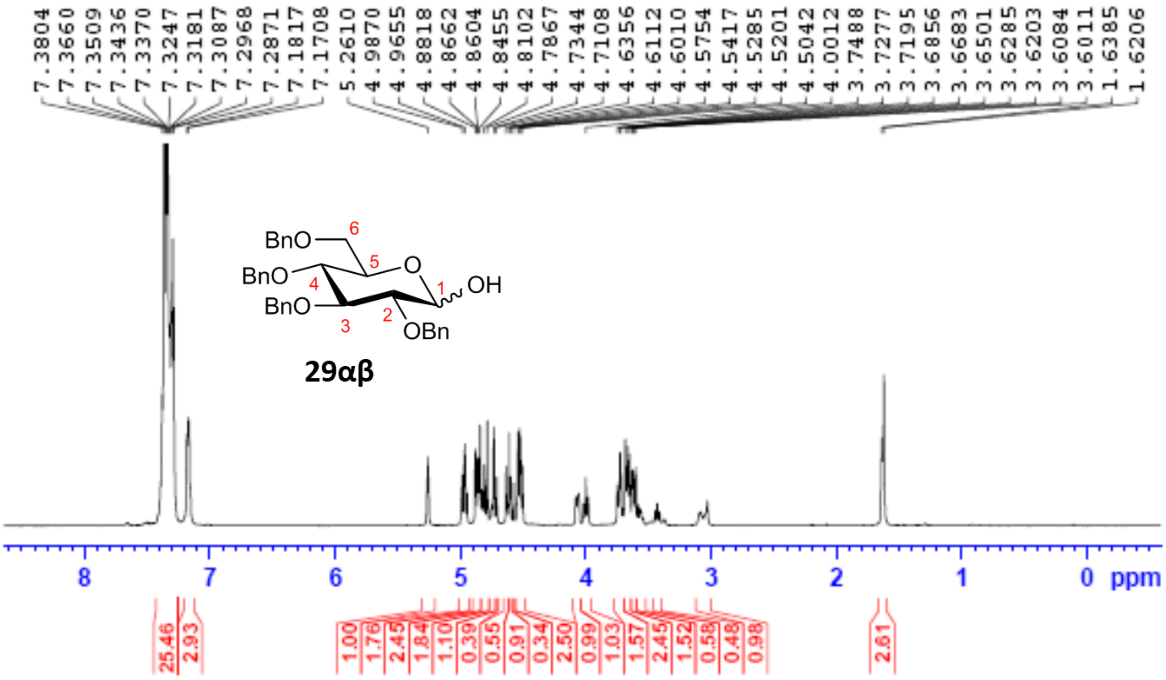

^{1}H NMR of 30αβ in $CDCl_3$

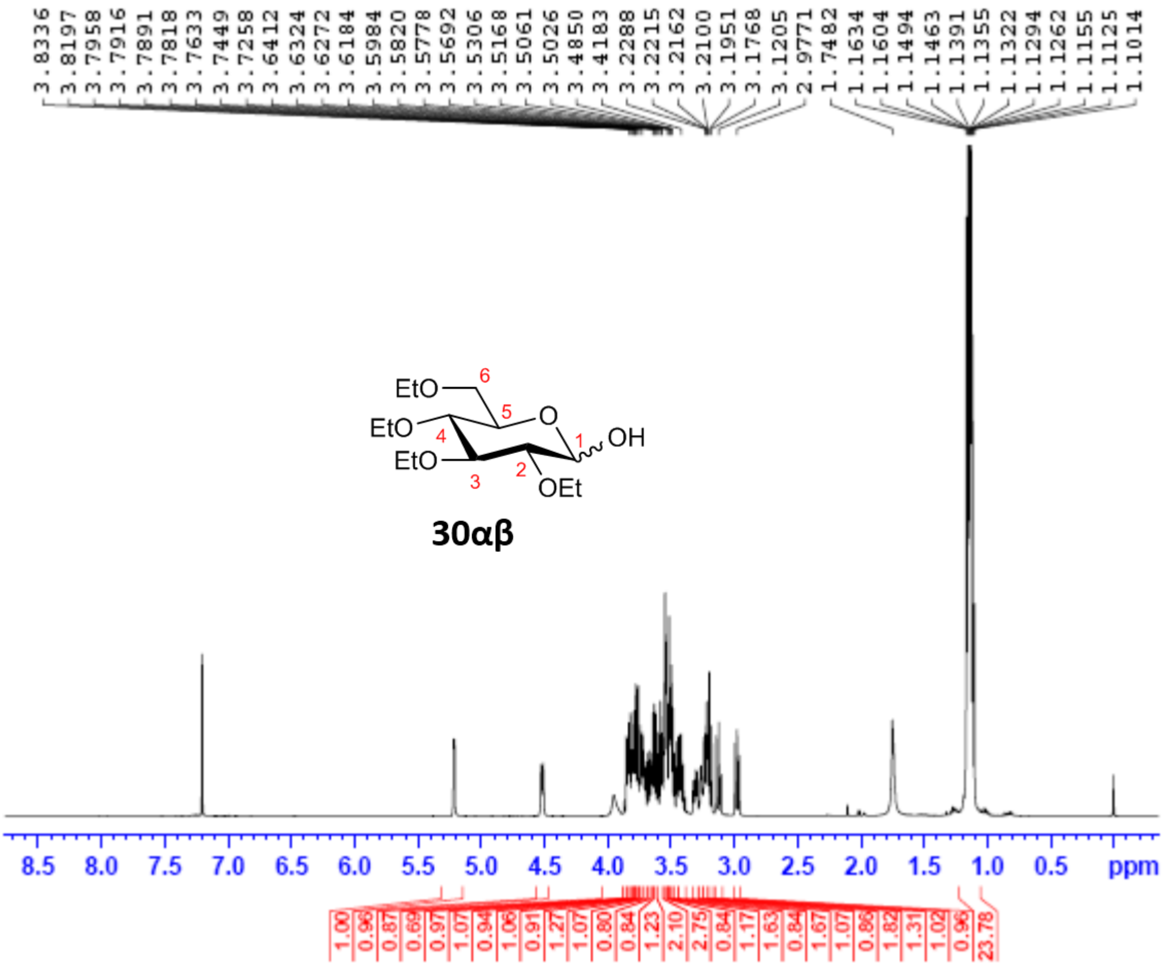

^{1}H NMR of 30αβ in $CDCl_3$ (ZOOM)

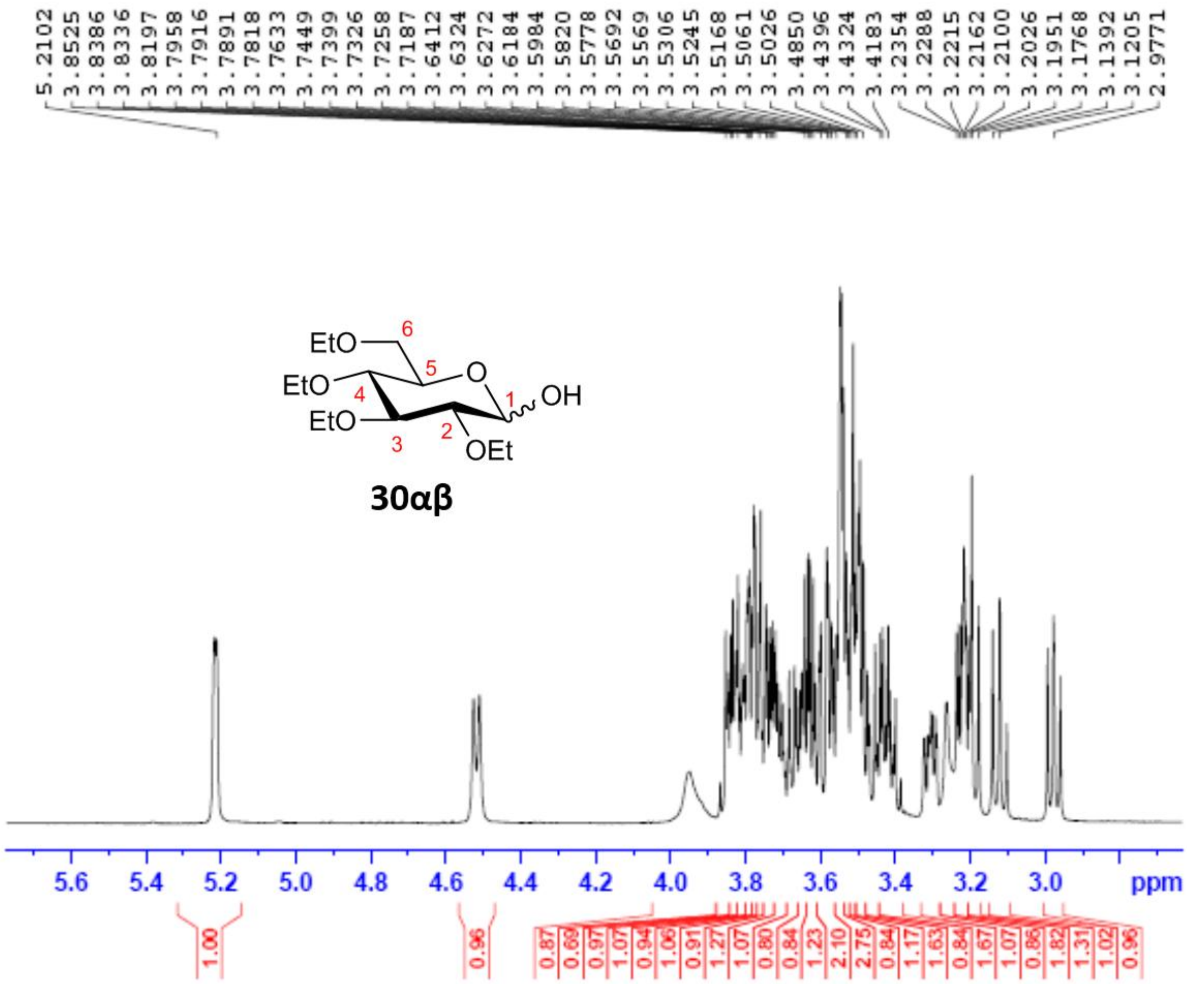

^{13}C NMR of 30αβ in $CDCl_3$

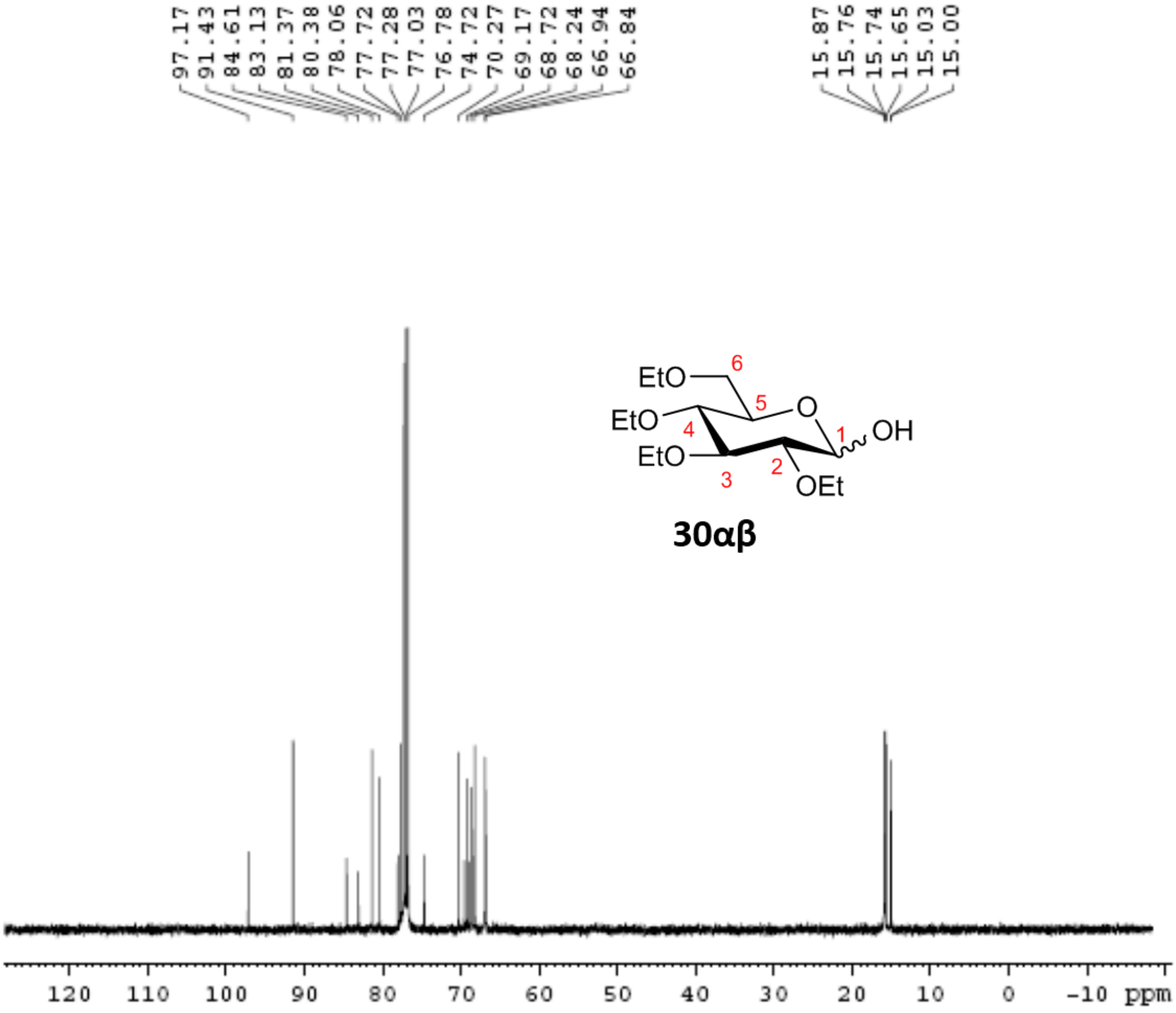

^{1}H NMR of 31αβ in CDCl$_3$

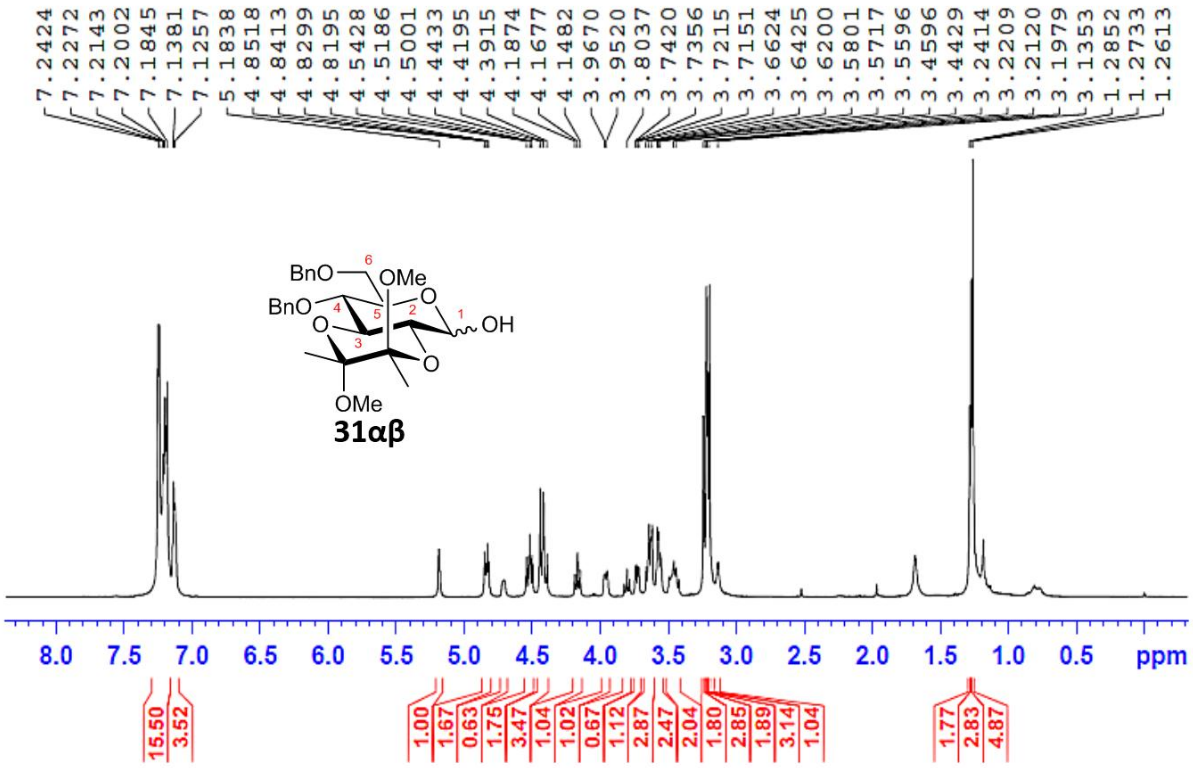

^{1}H NMR of 31αβ in $CDCl_3$ (ZOOM)

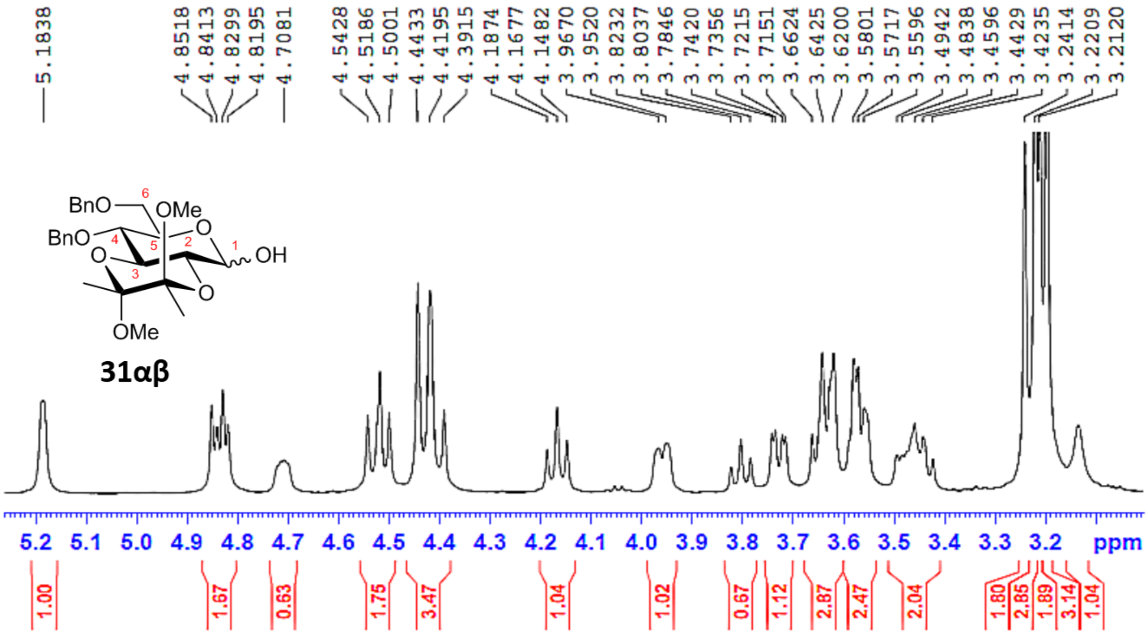

^{13}C NMR of 31αβ in CDCl$_3$

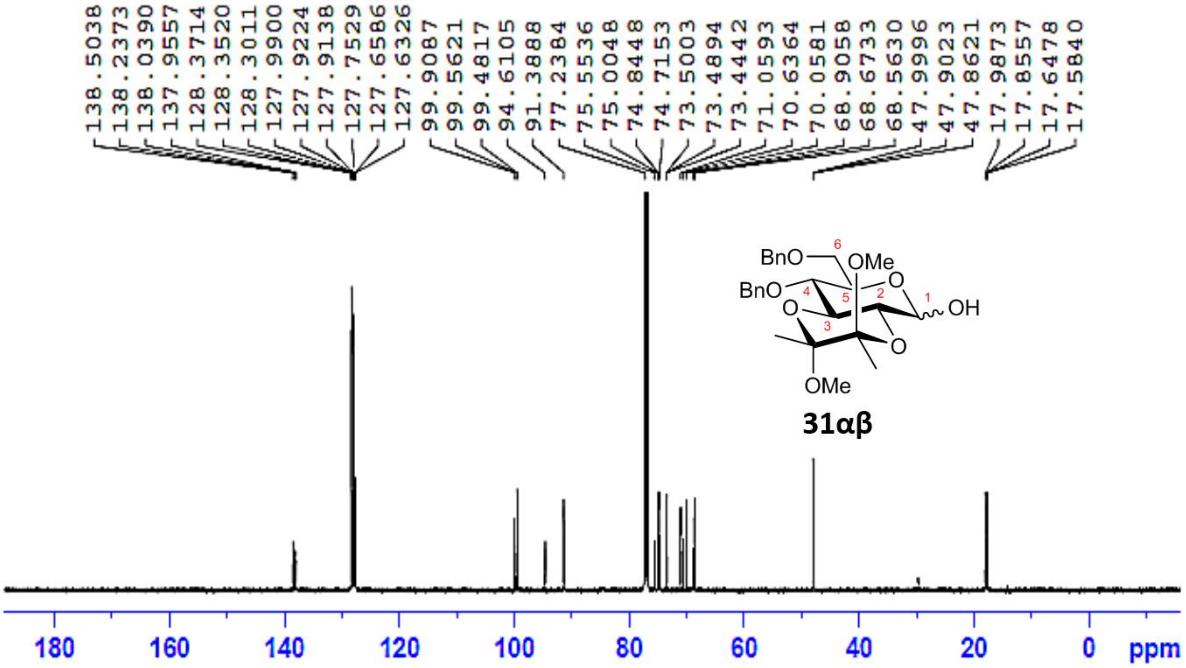

^{1}H NMR of 32αβ in CDCl$_3$

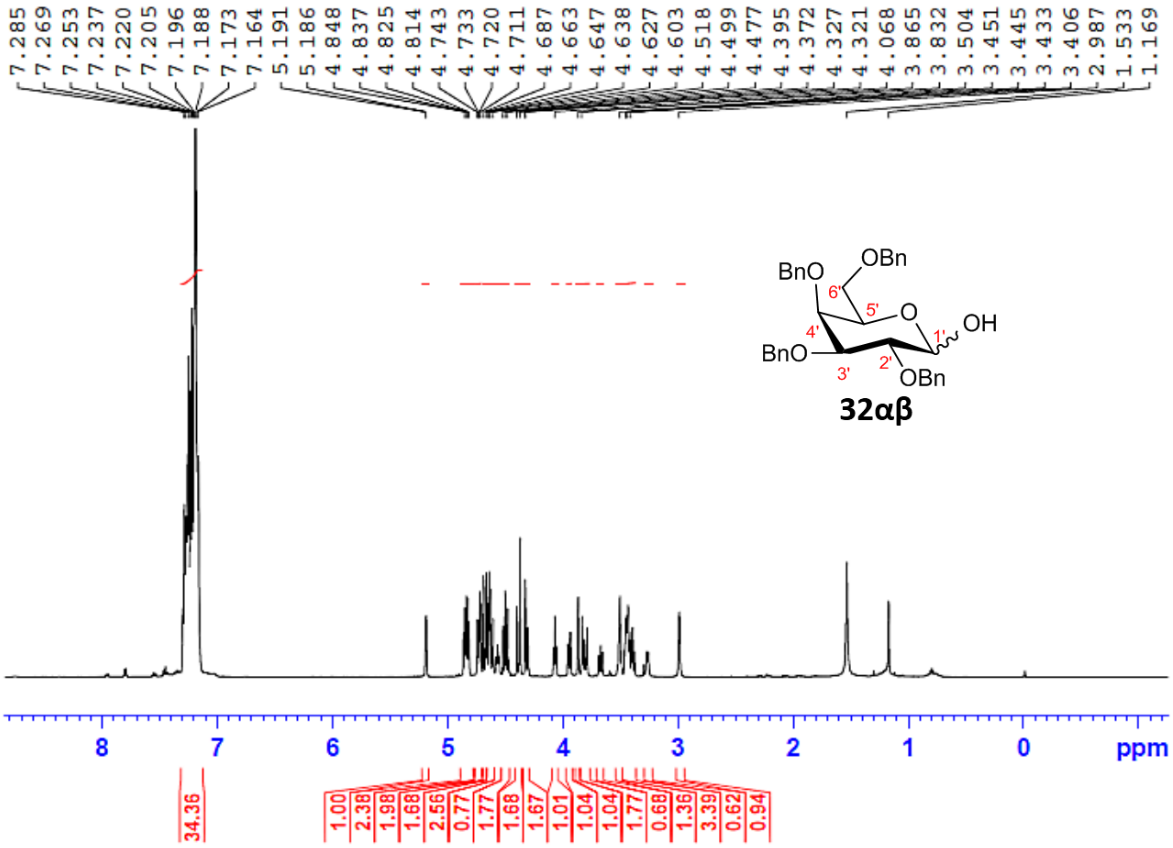
32αβ

^{1}H NMR of 33αβ in $CDCl_3$

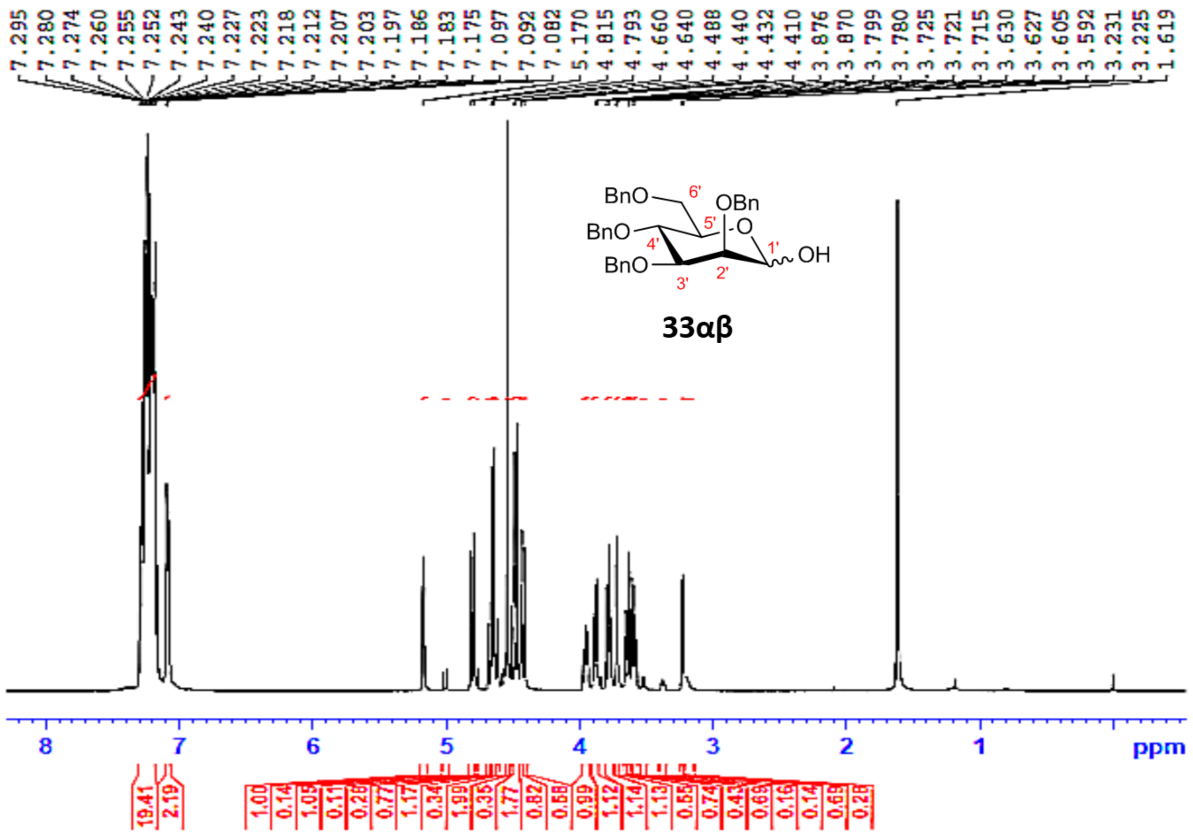

^{1}H NMR of 34αβ in $CDCl_3$

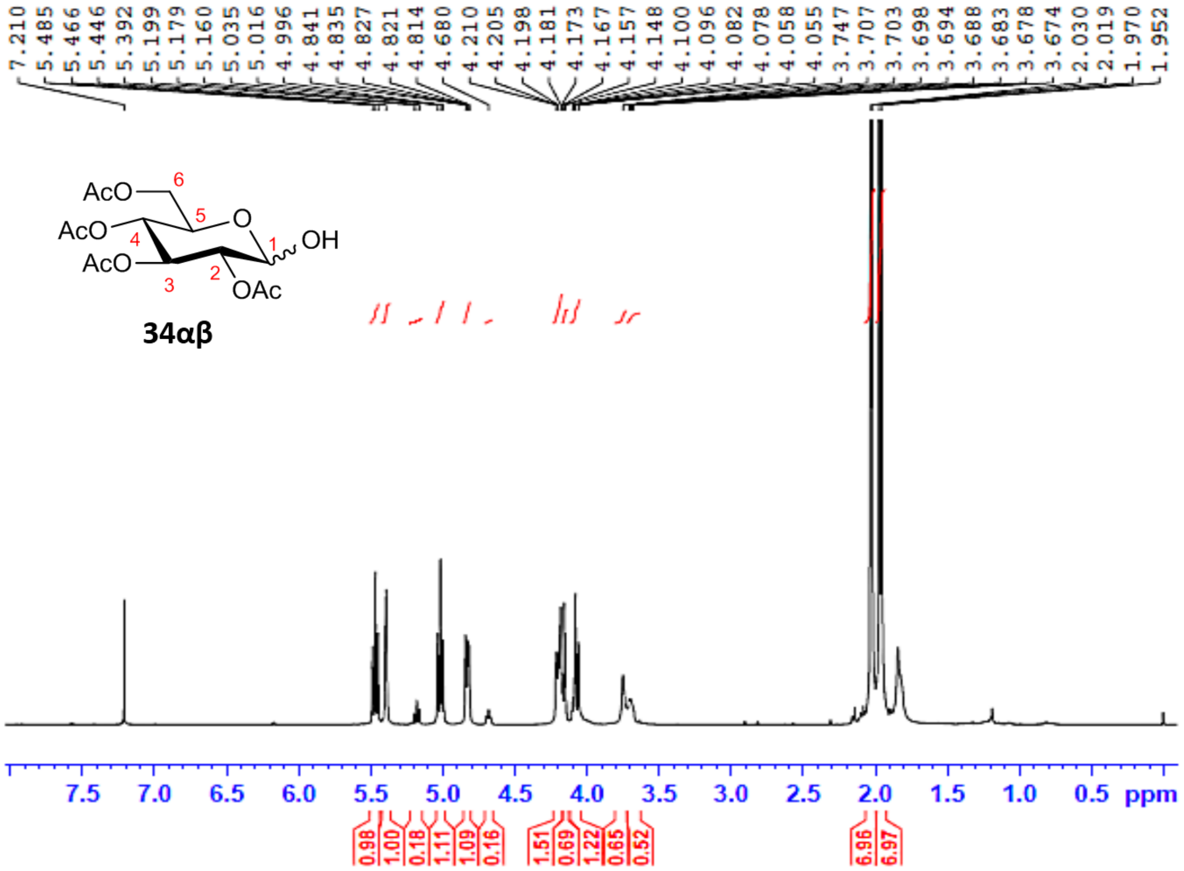

^{1}H NMR of 35αβ in $CDCl_3$

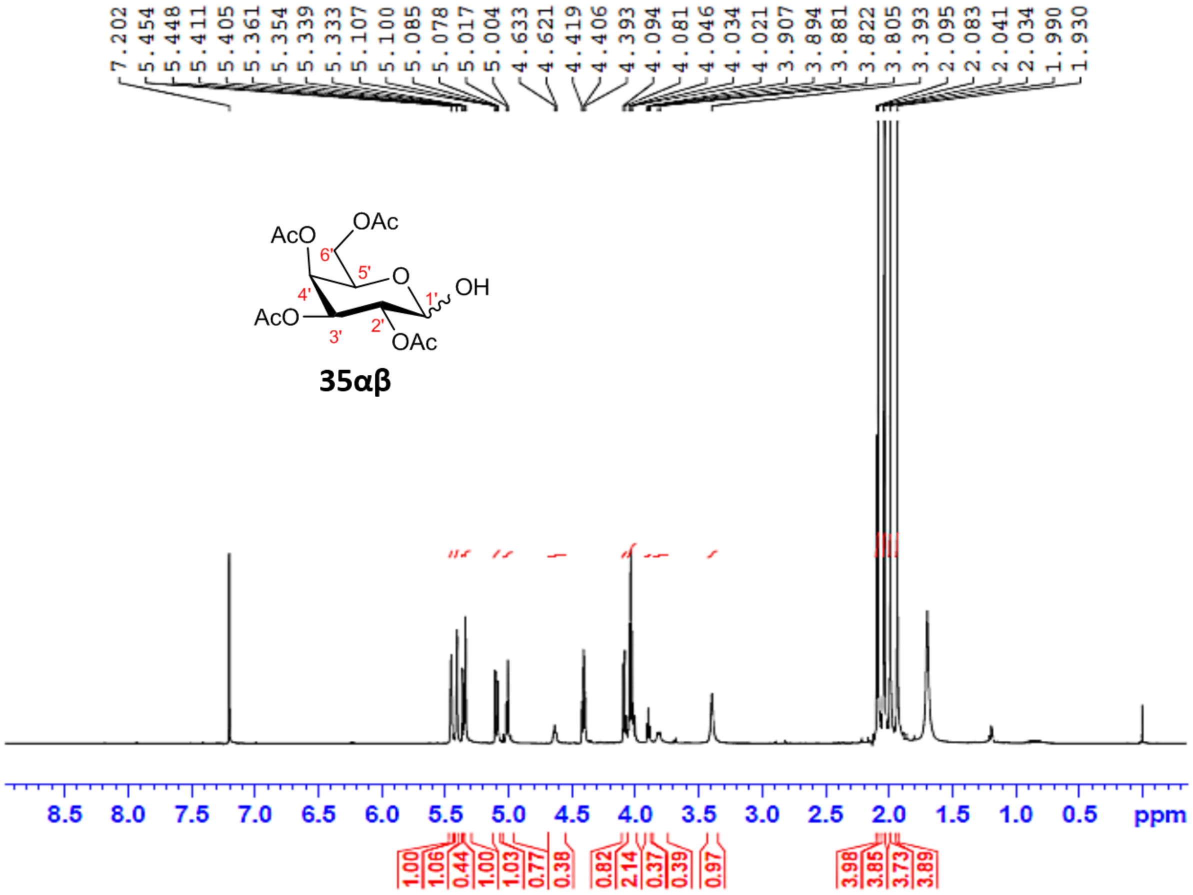

^{1}H NMR of 36αβ in CDCl$_3$

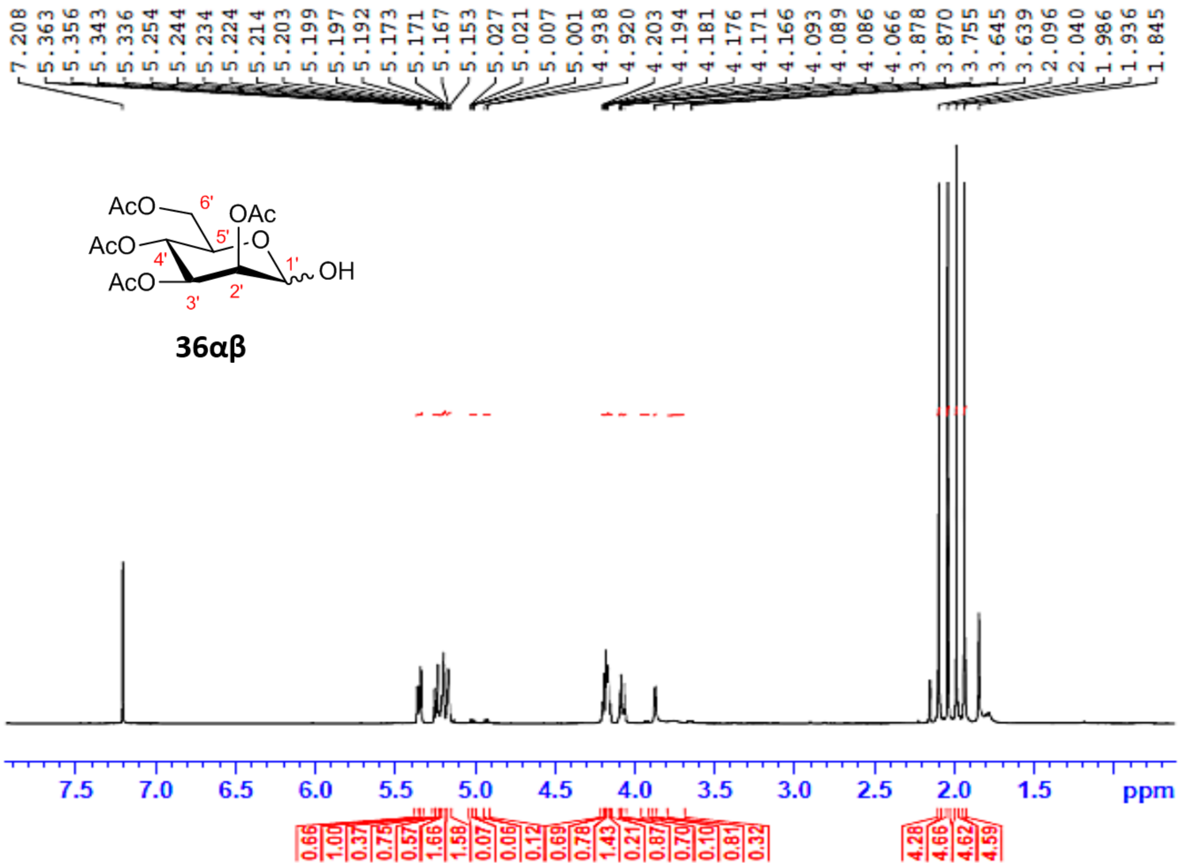

^{1}H NMR of 37αβ in $CDCl_3$

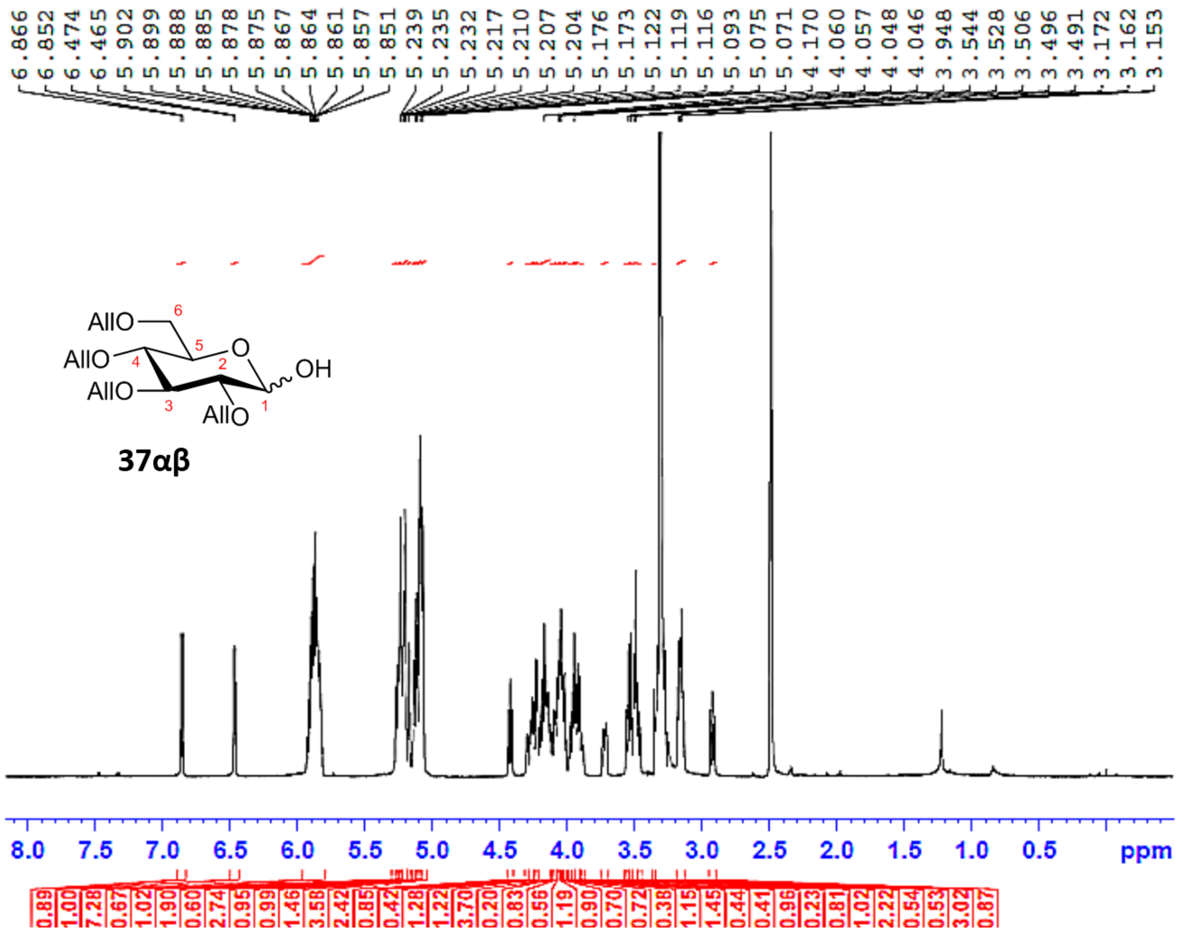

^{1}H NMR of 38αβ in CDCl$_3$

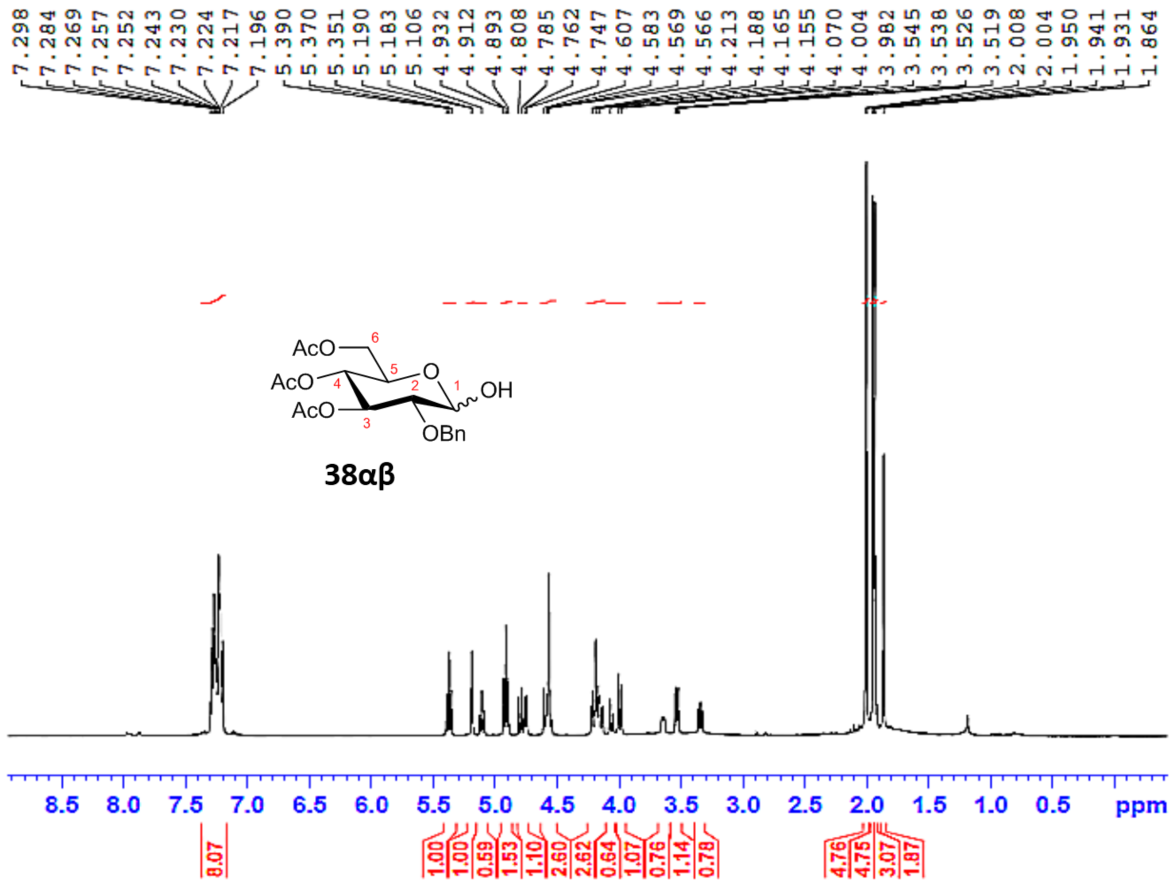

^{1}H NMR of 39αβ in $CDCl_3$

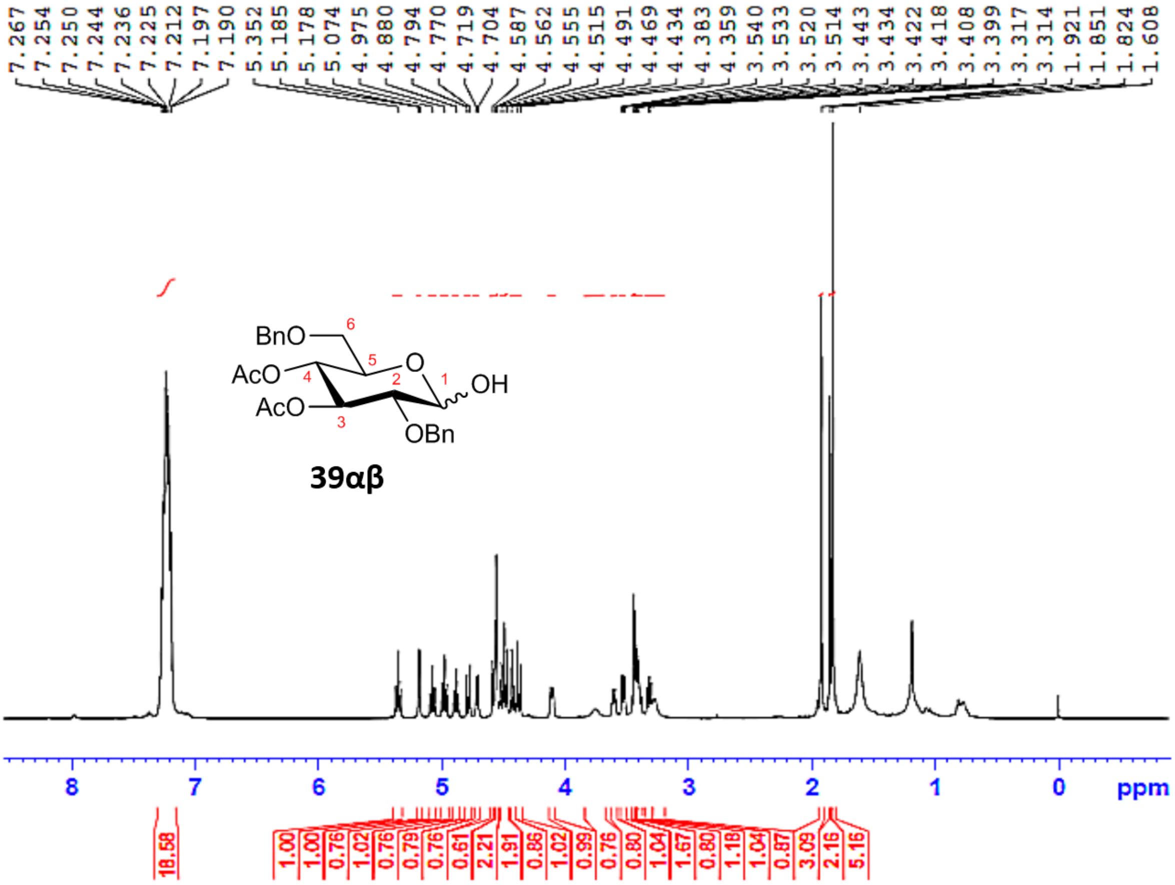

^{1}H NMR of diphenyl disulfide in $CDCl_3$

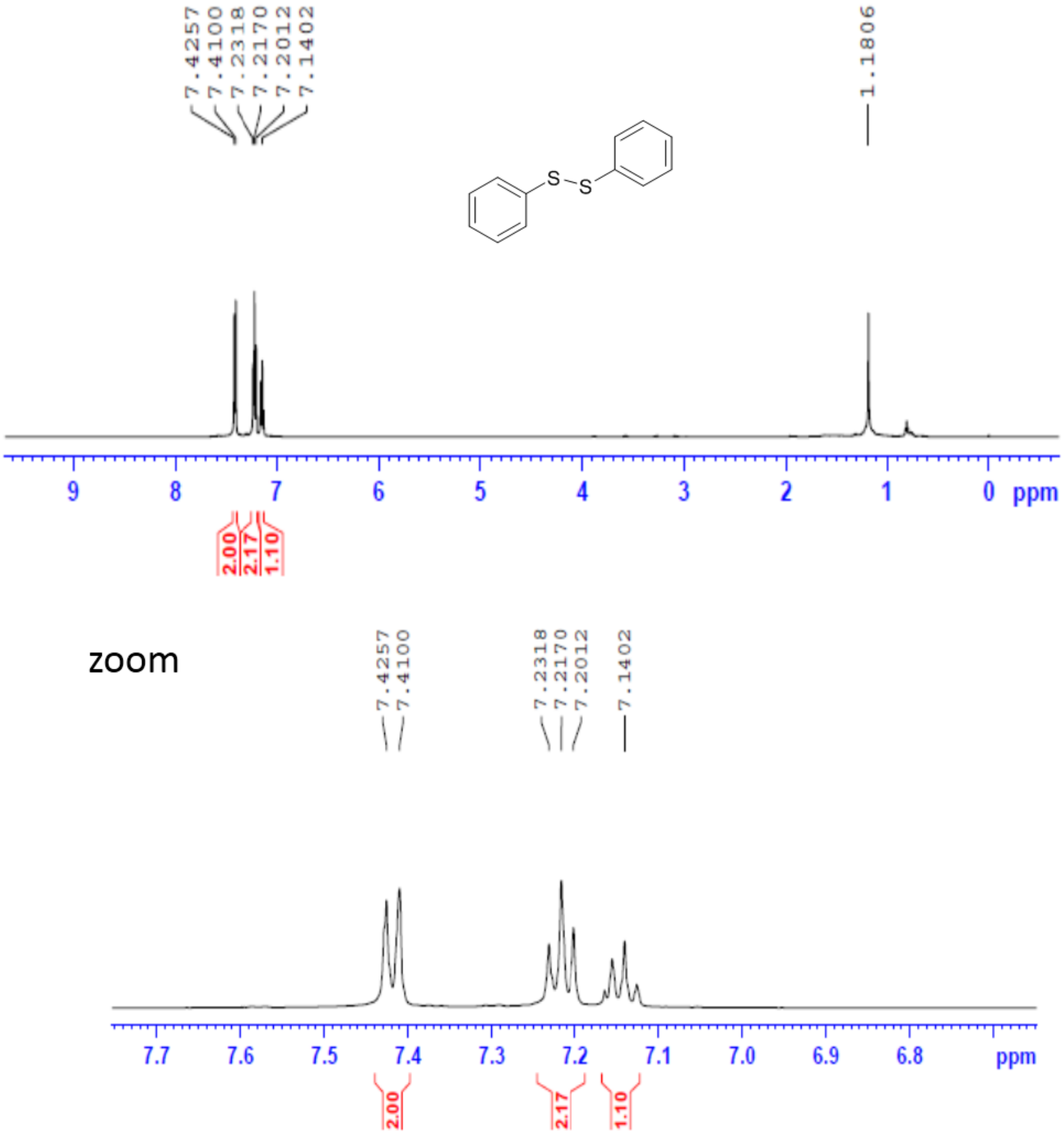

^{1}H NMR of thiophenol in $CDCl_3$

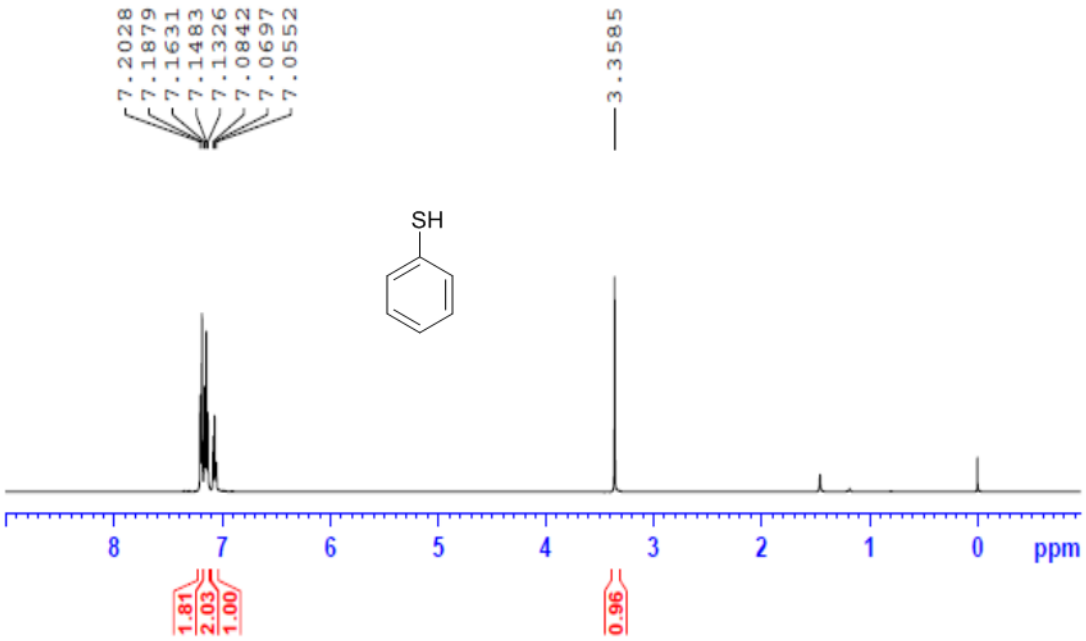

D$_2$O exchange

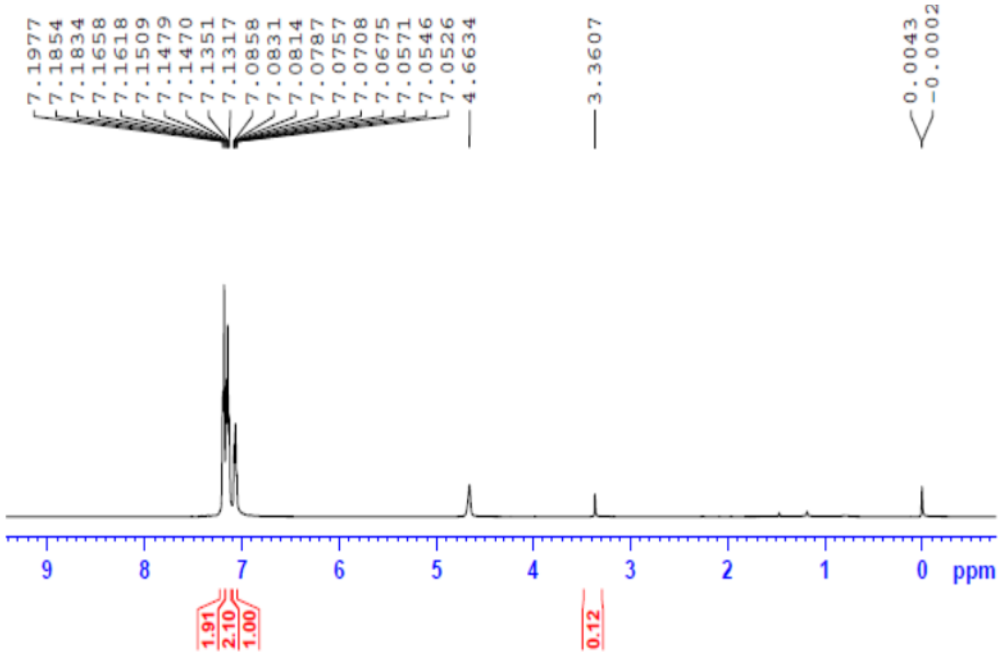

^{1}H NMR of *p*-tolyl disulfide in $CDCl_3$

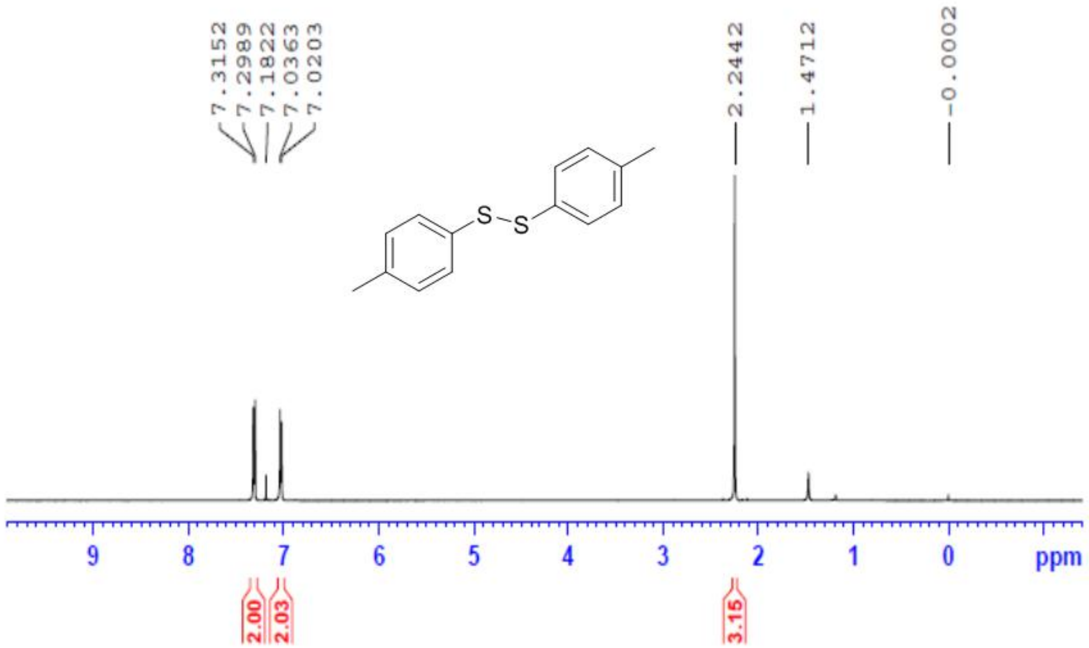

^{1}H NMR of 4-methylthiophenol in CDCl$_3$

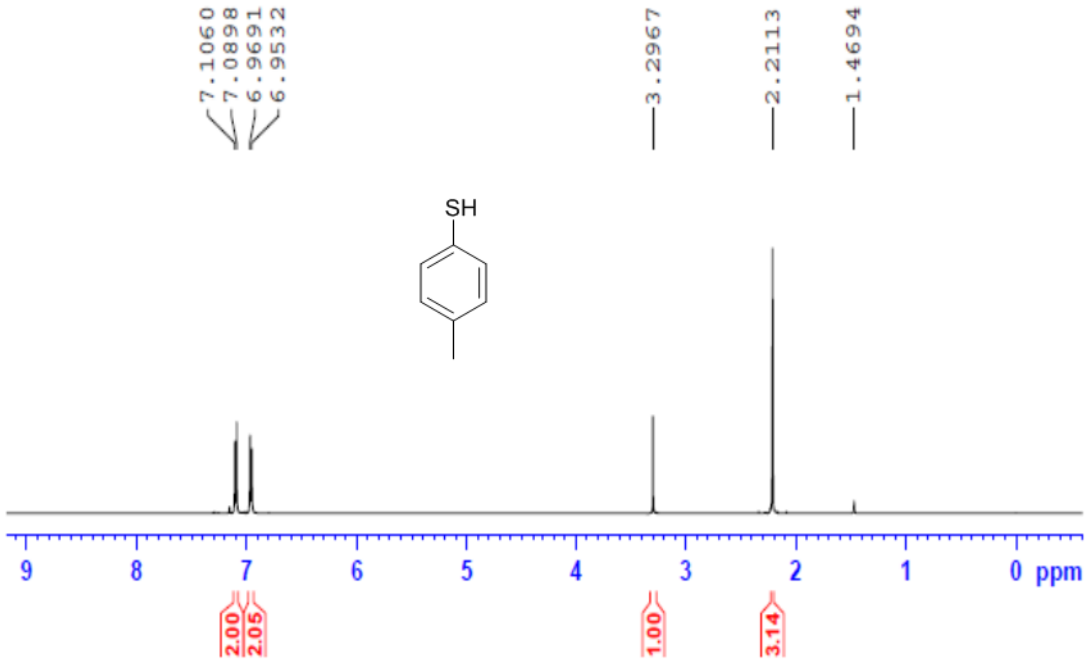

D$_2$O exchange

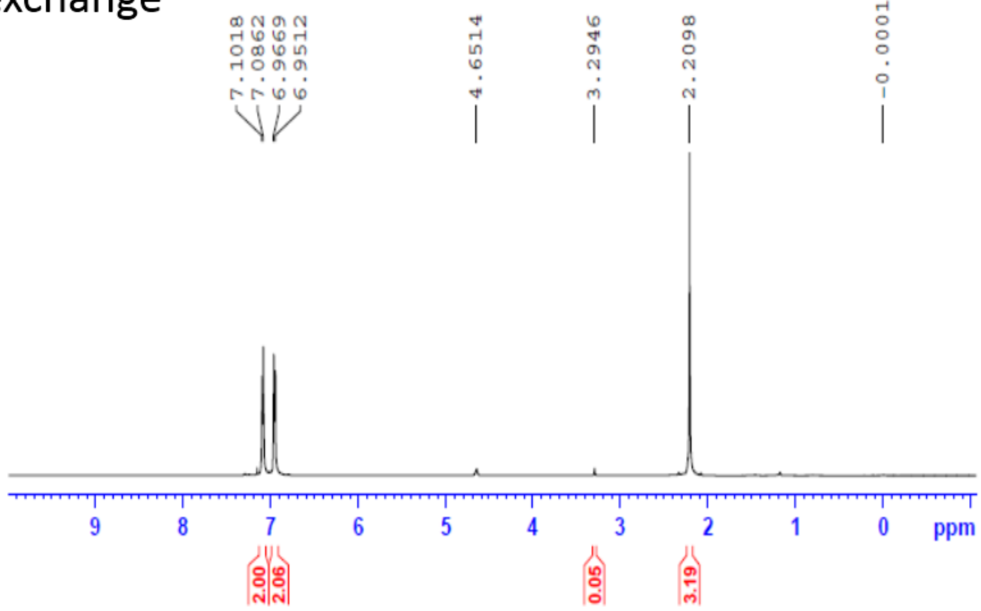

Supplement: SC-007-C6SC00633G-s001 [file SC-007-C6SC00633G-s001.pdf]
